# Supplementary material for: Kinetic resolution of indolines by asymmetric hydroxylamine formation
Source: Nat Commun. 2021 May 4;12:2512. doi: 10.1038/s41467-021-22658-3 (PMC8096955; doi:10.1038/s41467-021-22658-3)
Supplement: Supplementary file 1 — Supplementary Information [file 41467_2021_22658_MOESM1_ESM.pdf]

Supplementary Information

Kinetic Resolution of Indolines by Asymmetric Hydroxylamine Formation

Wang et al.

Supplementary Methods

General information

Proton (^{1}H NMR) and carbon (^{13}C NMR) nuclear magnetic resonance spectra were recorded at 500 MHz and 126 MHz, respectively. The chemical shifts are given in parts per million (ppm) on the delta (δ) scale. The solvent peak was used as a reference value, for ^{1}H NMR: $CDCl_3$ = 7.27 ppm, CD_3CN = 1.94 ppm; for ^{13}C NMR: $CDCl_3$ = 77.23 ppm, CD_3CN = 118.26 ppm. Analytical TLC was performed on precoated silica gel GF254 plates. Column chromatography was carried out on silica gel (200–300 mesh). IR spectra were recorded on an ALPHA-T spectrometer in the frequency range of 400-4000 cm^{-1}. HRMS were measured on an Orbitrap analyzer. Optical rotations were measured using a 2.5 mL cell with a 10 cm path length on Hanon P850 Automatic Polarimeter and concentrations (c) were reported in g×(100 mL)$^{-1}$. Enantiomeric excesses were determined by HPLC using a Daicel Chiralpak and Chiralcel column with hexane/*i*-PrOH as the eluent on Dionex instrument.

Synthesis of substrates and catalysts

Substrates **1v**, **6i and 6j** were known compounds and prepared following the established procedure.[1-3] Catalyst **C1** was prepared following established procedure.[4]

Substrate 1a-1u, 4a-4j, 6b and 6h were synthesized following a general method:

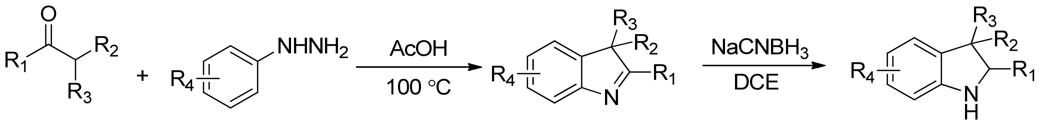

S1 **S2** **S3**

A mixture of arylhydrazine **S2** or its HCl salt (5.5 mmol) and **S1** (5 mmol) in AcOH (10 mL) was stirred at 100 °C for 1–6 h. The reaction was monitored by TLC. Upon completion, the reaction mixture was cooled with cold water and diluted with 1,2-dichloroethane (10 mL) followed by treatment with $NaCNBH_3$ (7.5 mmol, 1.5 equiv) in portions with cooling in cold water and was then stirred for 1 h at room temperature. The reaction was quenched with water, extracted with EtOAc and washed with sat. $NaHCO_3$. The organic layer was dried over $MgSO_4$, filtered, and concentrated. The residue was purified by chromatography with EtOAc/petroleum ether to provide the products.

Substrates 6a and 6c-6e were synthesized following a general method:

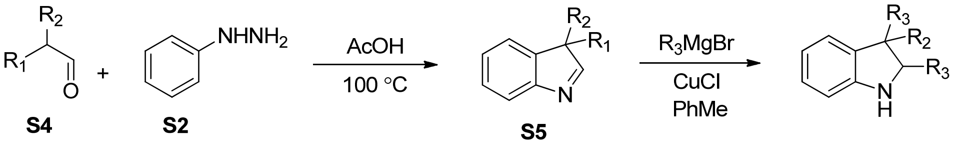

S4 **S2** **S5**

A mixture of **S4** (5.5 mmol)and **S2** (5 mmol) in AcOH (10 mL) was stirred at 100 °C for 2 h. The reaction was monitored by TLC. Upon completion, the reaction was

quenched with water, extracted with EtOAc and washed with sat. $NaHCO_3$. The organic layer was dried over $MgSO_4$, filtered, and concentrated. The residue was purified by chromatography with EtOAc/petroleum ether to provide **S5**.

A solution of **S5** (4 mmol) was added to a suspension of R_3MgBr (20 mmol) and CuCl (10 mg) in toluene (20 mL). The resulting mixture was refluxed for 18 h. The reaction was quenched with sat. NH_4Cl and extracted with EtOAc. The organic layer was dried over anhydrous $MgSO_4$, filtered, and concentrated. The residue was chromatographed on silica gel to afford the products.

Substrate 6f, 6g and 9 were synthesized following a general method:

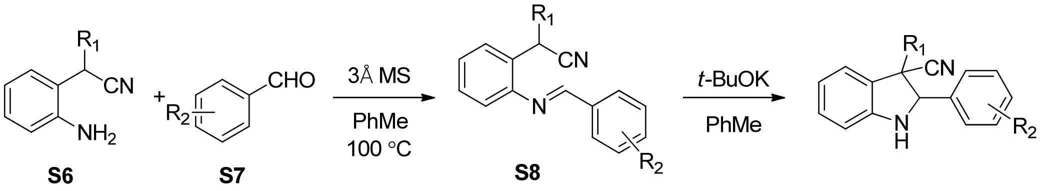

To a solution of **S7** (5.5 mmol) in toluene (25 mL) was added **S6** (5 mmol) and 3 Å MS (5.0 g). The reaction was stirred at 100 °C for 72 h before filtering through celite (EtOAc) and evaporating under reduced pressure. The residue was chromatographed on silica gel to afford **S8**.

KO*t*Bu (1.8 eq) was added to a solution of **S8** (1 eq, 2 mmol) in toluene (10 mL) and stirred at 0 °C until completion. Aqueous saturated NH_4Cl solution was added, and the mixture was extracted with EtOAc. The combined organic extracts were washed with brine, dried over $MgSO_4$, filtered, and concentrated. The residue was chromatographed on silica gel to afford the products.

Catalyst C2-C5 were synthesized following a general method

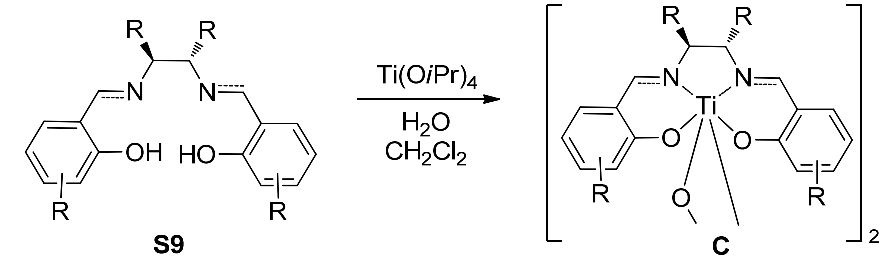

To a solution of salen or salanlen ligand **S9** (0.5 mmol) in dry CH_2Cl_2 (2.0 mL) was added $Ti(OiPr)_4$ (147 µL, 0.50 mmol) under nitrogen atmosphere, and the reaction mixture was stirred at room temperature for 3 h. Then water (27 mg, 1.5 mmol) was added and the resulting solution was stirred at room temperature for 3 h. The resulting precipitate was collected by filtration, washed with CH_2Cl_2 (0.5 mL x 2), and dried under vacuum to give the powder **C**.

Catalyst C6-C7 were synthesized following a known method[5]

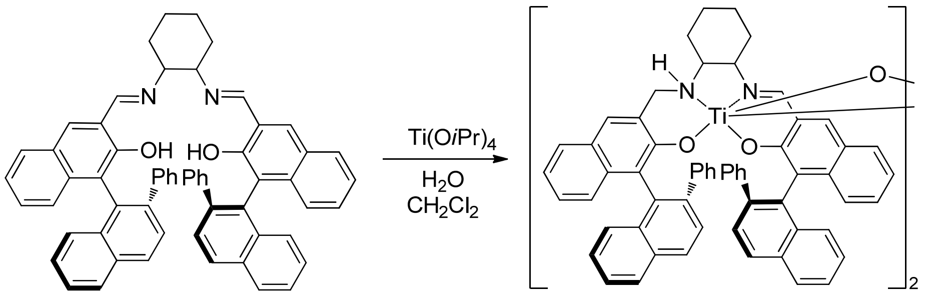

S10

C6 and **C7**

To a solution of salen ligand **S10** (418 mg, 0.5 mmol) in dry CH_2Cl_2 (2.0 mL) was added Ti(O*i*Pr)$_4$ (295 μL, 1.0 mmol) under nitrogen atmosphere, and the reaction mixture was stirred at room temperature for 3 days. Water (36 mg, 2.0 mmol) was added and the reaction mixture was stirred at the same temperature for 4 h. Then CH_2Cl_2 (10 mL) was added and the mixture was filtered and concentrated. The residue was recrystallized from diethyl ether and CH_2Cl_2 to give **C6** or **C7**. The catalyst **C6** and **C7** are pretty stable, and can be stored at ambient temperature for several months.

General procedure

To a solution of racemic substrate (0.1 mmol, 1.0 eq) in $CHCl_3$ (1.0 mL) was added 30% aqueous hydrogen peroxide (0.1 mmol, 10 μL, 1.0 eq) and **C6** (0.001 mmol, 1.8 mg, 1 mmol%) at room temperature. The reaction was vigorously stirred for 4-12 h. Then the mixture was diluted with CH_2Cl_2 (20 mL), washed with water (10 mL), dried over $MgSO_4$, filtered and concentrated. The residue was purified by silica gel chromatography (EtOAc/petroleum ether) to give the desired product.

Analytical data for products

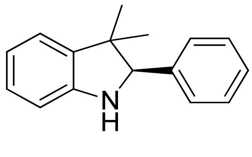

(*R*)-3,3-Dimethyl-2-phenylindoline (1a)
It was prepared following the general procedure and purified by silica gel flash chromatography using ethyl acetate/ petroleum ether (10:90) as eluent to afford **1a** (10.5 mg, 47% yield) and **2a** (10.8 mg, 45% yield). ^{1}H NMR (500 MHz, $CDCl_3$) δ 7.51 (d, *J* = 7.0 Hz, 2H), 7.42 (t, *J* = 7.4 Hz, 2H), 7.38–7.34 (m, 1H), 7.27–7.26 (m, 1H), 7.16–7.08 (m, 2H), 7.04 (td, *J* = 7.4, 1.0 Hz, 1H), 5.57 (s, 1H), 4.31 (s, 1H), 1.43 (s, 3H), 0.80 (s, 3H); ^{13}C NMR (126 MHz, $CDCl_3$) δ 151.3, 137.4, 137.3, 128.5, 128.2, 127.9, 127.8, 123.2, 122.2, 113.7, 85.4, 43.9, 29.9, 26.0, 25.3; HPLC: the ee value was determined by HPLC analysis (Chiralpak IB, *i*-PrOH/Hexane = 10/90, 1.0 mL/min, 247 nm), retention time: t_{major} = 5.647 min, t_{minor} = 11.103 min, ee = 92%; $[\alpha]_D^{20}$ = – 116.7 (c = 0.84, THF). HRMS *m*/*z* $[M + H]^+$ calculated for $C_{16}H_{18}N$: 224.1434, found 224.1433. The absolute configuration of **1a** was assigned to be *R* by comparing the optical rotation and HPLC analysis with reported data. [6,7]

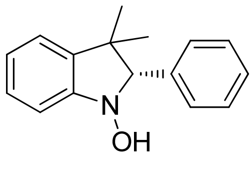

(*S*)-3,3-Dimethyl-2-phenylindolin-1-ol (2a)
^{1}H NMR (500 MHz, $CDCl_3$) δ 7.52 (d, *J* = 7.3 Hz, 2H), 7.43 (t, *J* = 7.4 Hz, 2H), 7.38 (t, *J* = 7.2 Hz, 1H), 7.28 (t, *J* = 7.6 Hz, 1H), 7.16 (d, *J* = 7.4 Hz, 1H), 7.10 (d, *J* = 7.8 Hz, 1H), 7.06 (dd, *J* = 7.9, 6.9 Hz, 1H), 5.72 (s, 1H), 4.34 (s, 1H), 1.45 (s, 3H), 0.82 (s, 3H); ^{13}C NMR (126 MHz, $CDCl_3$) δ 151.3, 137.4, 137.3, 128.5, 128.2, 127.9, 127.8, 123.2, 122.2, 113.7, 85.4, 43.9, 26.0, 25.3; HPLC: the ee value was determined by HPLC analysis (Chiralpak IB, *i*-PrOH/Hexane = 5/95, 1.0 mL/min, 247 nm), retention time: t_{major} = 6.847 min, t_{minor} = 10.427 min, ee = 92%; $[\alpha]_D^{20}$ = + 175.5 (c = 0.20, THF). HRMS *m*/*z* $[M + H]^+$ calculated for $C_{16}H_{18}NO$: 240.1383, found 240.1388.

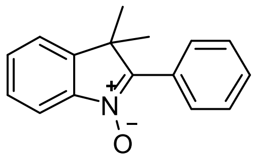

3,3-Dimethyl-2-phenyl-3*H*-indole 1-oxide (3)

^{1}H NMR (500 MHz, $CDCl_3$) δ 8.59–8.50 (m, 2H), 7.92–7.85 (m, 1H), 7.57–7.42 (m, 6H), 1.68 (s, 6H); ^{13}C NMR (126 MHz, $CDCl_3$) δ 149.9, 146.1, 142.4, 130.3, 129.3, 128.8, 128.7, 128.5, 128.1, 121.2, 115.5, 47.8, 25.5; HRMS *m*/*z* $[M + H]^+$ calculated for $C_{16}H_{16}NO$: 238.1226, found 238.1220.

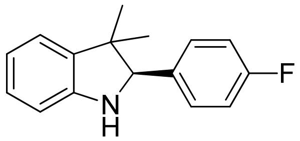

(*R*)-2-(4-Fluorophenyl)-3,3-dimethylindoline (1b)

It was prepared following the general procedure and purified by silica gel flash chromatography using ethyl acetate/ petroleum ether (15:85) as eluent to afford **1b** (10.8 mg, 45% yield) and **2b** (12.3 mg, 48% yield). ^{1}H NMR (500 MHz, $CDCl_3$) δ 7.47–7.41 (m, 2H), 7.17–7.00 (m, 4H), 6.81 (td, *J* = 7.4, 0.7 Hz, 1H), 6.74 (d, *J* = 7.7 Hz, 1H), 4.59 (s, 1H), 4.13 (brs, 1H), 1.42 (s, 3H), 0.73 (s, 3H); ^{13}C NMR (126 MHz, $CDCl_3$) δ 162.5 (d, *J* = 245.3 Hz), 149.2, 138.1, 135.7, 129.1 (d, *J* = 7.8 Hz), 127.7, 122.7, 119.4, 115.1 (d, *J* = 21.2 Hz), 109.5, 74.1, (Chiralpak IB, *i*-PrOH/Hexane = 10/90, 1.0 mL/min, 302 nm), retention time: t_{major} = 5.283 min, t_{minor} = 10.153 min, ee = 98%; $[\alpha]_D^{20}$ = – 145.1 (c = 0.48, THF). HRMS *m*/*z* $[M + H]^+$ calculated for $C_{16}H_{17}NF$: 242.1340, found 242.1344.

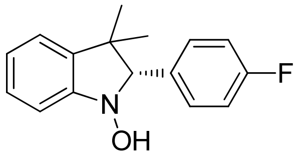

(*S*)-2-(4-Fluorophenyl)-3,3-dimethylindolin-1-ol (2b)

^{1}H NMR (500 MHz, $CDCl_3$) δ 7.50–7.44 (m, 2H), 7.27 (td, *J* = 7.7, 1.2 Hz, 1H), 7.20–7.08 (m, 4H), 7.06 (td, *J* = 7.4, 1.0 Hz, 1H), 5.67 (s, 1H), 4.29 (s, 1H), 1.41 (s, 3H), 0.79 (s, 3H); ^{13}C NMR (126 MHz, $CDCl_3$) δ 162.7 (d, *J* = 245.6 Hz), 151.1, 137.2, 133.0 (d, *J* = 3.1 Hz), 129.72 (d, *J* = 7.9 Hz), 127.9, 123.4, 122.2, 115.38 (d, *J* = 21.3 Hz), 113.7, 84.7, 43.8, 26.0, 25.2; HPLC: the ee value was determined by HPLC analysis (Chiralpak IB, *i*-PrOH/Hexane = 5/95, 1.0 mL/min, 249 nm), retention time: t_{major} = 6.473 min, t_{minor} = 9.497 min, ee = 92%; $[\alpha]_D^{20}$ = + 167.4 (c = 0.17, THF). HRMS *m*/*z* $[M + H]^+$ calculated for $C_{16}H_{17}FNO$: 258.1289, found 259.1297.

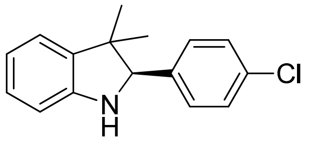

(*R*)-2-(4-Chlorophenyl)-3,3-dimethylindoline (1c)

It was prepared following the general procedure and purified by silica gel flash chromatography using ethyl acetate/ petroleum ether (15:85) as eluent to afford **1c** (12.1 mg, 47% yield) and **2c** (12.3 mg, 45% yield). ^{1}H NMR (500 MHz, $CDCl_3$) δ 7.40 (d, *J* = 8.4 Hz, 2H), 7.33 (d, *J* = 8.5 Hz, 2H), 7.15–7.02 (m, 2H), 6.80 (td, *J* = 7.4, 0.7 Hz, 1H), 6.73 (d, *J* = 7.7 Hz, 1H), 4.58 (s, 1H), 4.07 (s, 1H), 1.42 (s, 3H), 0.72 (s, 3H); ^{13}C NMR (126 MHz, $CDCl_3$) δ 149.3, 138.7, 138.0, 133.3, 128.9, 128.5, 127.7, 122.7, 119.4, 109.5, 74.1, 45.6, 26.6, 24.7; HPLC: the ee value was determined by HPLC analysis (Chiralpak IB, *i*-PrOH/Hexane = 10/90, 1.0 mL/min, 246 nm), retention time: t_{major} = 5.323 min, t_{minor} = 11.377 min, ee = 99%; $[\alpha]_D^{20}$ = – 154.7 (c = 0.48, THF). HRMS *m*/*z* $[M + H]^+$ calculated for $C_{16}H_{17}ClN$: 258.1044, found 258.1045.

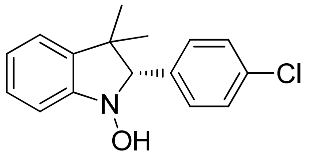

(*S*)-2-(4-Chlorophenyl)-3,3-dimethylindolin-1-ol (2c)

^{1}H NMR (500 MHz, $CDCl_3$) δ 7.44 (d, *J* = 8.4 Hz, 2H), 7.39 (d, *J* = 8.5 Hz, 2H), 7.27 (td, *J* = 7.7, 1.2 Hz, 1H), 7.13 (d, *J* = 6.9 Hz, 1H), 7.09 (d, *J* = 7.8 Hz, 1H), 7.05 (td, *J* = 7.4, 0.9 Hz, 1H), 5.67 (s, 1H), 4.28 (s, 1H), 1.42 (s, 3H), 0.79 (s, 3H); ^{13}C NMR (126 MHz, $CDCl_3$) δ 151.1, 137.1, 135.9, 133.7, 129.5, 128.7, 127.9, 123.4, 122.2, 113.7, 84.7, 43.9, 26.0, 25.2; HPLC: the ee value was determined by HPLC analysis (Chiralpak IB, *i*-PrOH/Hexane = 5/95, 1.0 mL/min, 247 nm), retention time: t_{major} = 8.133 min, t_{minor} = 11.520 min, ee = 93%; $[\alpha]_D^{20}$ = + 140.7 (c = 0.23, THF). HRMS *m*/*z* $[M + H]^+$ calculated for $C_{16}H_{17}ClN$: 274.0993, found 274.1001.

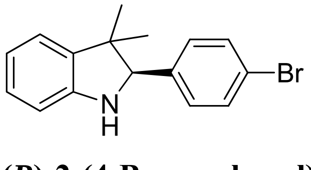

(*R*)-2-(4-Bromophenyl)-3,3-dimethylindoline (1d)

It was prepared following the general procedure and purified by silica gel flash chromatography using ethyl acetate/ petroleum ether (15:85) as eluent to afford **1d** (13.9 mg, 46% yield) and **2d** (14.6 mg, 46% yield). ^{1}H NMR (500 MHz, $CDCl_3$) δ 7.59–7.45 (m, 2H), 7.41–7.34 (m, 2H), 7.17–7.04 (m, 2H), 6.89–6.80 (m, 1H), 6.80–6.69 (m, 1H), 4.58 (s, 1H), 4.14 (brs, 1H), 1.44 (s, 3H), 0.74 (s, 3H); ^{13}C NMR (126 MHz, $CDCl_3$) δ 149.2, 139.2, 138.1, 131.4, 129.3, 127.7, 122.7, 121.5, 119.5, 109.6, 74.1, 45.6, 26.6, 24.7; HPLC: the ee value was determined by HPLC analysis (Chiralpak IB, *i*-PrOH/Hexane = 10/90, 1.0 mL/min, 303 nm), retention time: t_{major} = 5.677 min, t_{minor} = 12.473 min, ee = 99%; $[\alpha]_D^{20}$ = – 131.1 (c = 0.48, THF). HRMS

m/z $[M + H]^+$ calculated for $C_{16}H_{17}BrN$: 304.0524, found 304.0521.

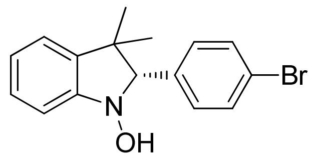

(*S*)-2-(4-Bromophenyl)-3,3-dimethylindolin-1-ol (2d)

^{1}H NMR (500 MHz, $CDCl_3$) δ 7.54 (d, *J* = 8.4 Hz, 2H), 7.38 (d, *J* = 8.4 Hz, 2H), 7.26 (td, *J* = 7.7, 1.1 Hz, 1H), 7.13 (d, *J* = 7.3 Hz, 1H), 7.09 (d, *J* = 7.8 Hz, 1H), 7.05 (td, *J* = 7.4, 0.9 Hz, 1H), 5.63 (s, 1H), 4.26 (s, 1H), 1.41 (s, 3H), 0.79 (s, 3H); ^{13}C NMR (126 MHz, $CDCl_3$) δ 151.1, 137.1, 136.4, 131.7, 129.9, 128.0, 123.4, 122.2, 121.8, 113.8, 84.8, 43.9, 26.0, 25.2; HPLC: the ee value was determined by HPLC analysis (Chiralpak IB, *i*-PrOH/Hexane = 5/95, 1.0 mL/min, 244 nm), retention time: t_{major} = 8.693 min, t_{minor} = 11.3103 min, ee = 91%; $[\alpha]_D^{20}$ = + 93.3 (c = 0.27, THF). HRMS m/z $[M + H]^+$ calculated for $C_{16}H_{17}BrNO$: 320.0473, found 320.0477.

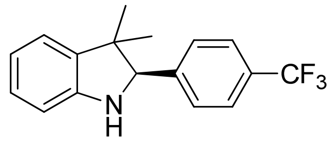

(*R*)-3,3-Dimethyl-2-(4-(trifluoromethyl)phenyl)indoline (1e)

It was prepared following the general procedure and purified by silica gel flash chromatography using ethyl acetate/ petroleum ether (20:80) as eluent to afford **1e** (12.5 mg, 43% yield) and **2e** (14.4 mg, 47% yield). ^{1}H NMR (500 MHz, $CDCl_3$) δ 7.66–7.56 (m, 4H), 7.17–7.02 (m, 2H), 6.84 (dt, *J* = 8.4, 4.2 Hz, 1H), 6.76 (d, *J* = 7.7 Hz, 1H), 4.68 (s, 1H), 4.19 (brs, 1H), 1.47 (s, 3H), 0.74 (s, 3H); ^{13}C NMR (126 MHz, $CDCl_3$) δ 149.1, 144.4, 137.9, 130.0 (q, *J* = 32.3 Hz), 127.9, 127.8, 125.3 (q, *J* = 3.7 Hz), 124.5 (q, *J* = 272.0 Hz), 122.7, 119.6, 109.6, 74.2, 45.8, 26.7, 24.8; HPLC: the ee value was determined by HPLC analysis (Chiralpak IB, *i*-PrOH/Hexane = 10/90, 1.0 mL/min, 302 nm), retention time: t_{major} = 5.487 min, t_{minor} = 13.090 min, ee = 99%; $[\alpha]_D^{20}$ = – 117.6 (c = 0.25, THF). HRMS m/z $[M + H]^+$ calculated for $C_{17}H_{17}F_3N$: 292.1308, found 292.1305.

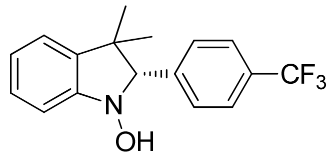

(*S*)-3,3-Dimethyl-2-(4-(trifluoromethyl)phenyl)indolin-1-ol (2e)

^{1}H NMR (500 MHz, $CDCl_3$) δ 7.68 (d, *J* = 8.2 Hz, 2H), 7.63 (d, *J* = 8.2 Hz, 2H), 7.28 (td, *J* = 7.6, 1.2 Hz, 1H), 7.15 (d, *J* = 7.3 Hz, 1H), 7.10 (d, *J* = 7.8 Hz, 1H), 7.07 (td, *J* = 7.4, 1.0 Hz, 1H), 5.70 (s, 1H), 4.37 (s, 1H), 1.45 (s, 3H), 0.79 (s, 3H); ^{13}C NMR (126 MHz, $CDCl_3$) δ 151.0, 141.7, 137.0, 130.2 (q, *J* = 32.4 Hz), 128.5, 128.0, 125.5 (q, *J* = 3.7 Hz), 124.4 (q, *J* = 272.0 Hz), 123.6, 122.2, 113.8, 84.9, 44.1, 26.0, 25.3; HPLC: the ee value was determined by HPLC analysis (Chiralpak IB, *i*-PrOH/Hexane

= 5/95, 1.0 mL/min, 214 nm), retention time: t_{major} = 6.373 min, t_{minor} = 10.997 min, ee = 92%; $[\alpha]_D^{20}$ = + 157.2 (c = 0.27, THF). HRMS *m*/*z* $[M + H]^+$ calculated for $C_{17}H_{17}F_3NO$: 308.1257, found 308.1252.

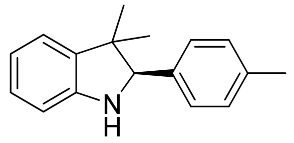

(*R*)-3,3-Dimethyl-2-(*p*-tolyl)indoline (1f)

It was prepared following the general procedure and purified by silica gel flash chromatography using ethyl acetate/ petroleum ether (10:90) as eluent to afford **1f** (10.9 mg, 46% yield) and **2f** (11.9 mg, 47% yield). ^1H NMR (500 MHz, $CDCl_3$) δ 7.39–7.32 (m, 2H), 7.24–7.14 (m, 2H), 7.13–7.01 (m, 2H), 6.85–6.76 (m, 1H), 6.73 (d, *J* = 7.7 Hz, 1H), 4.58 (s, 1H), 4.07 (brs, 1H), 2.37 (s, 3H), 1.43 (s, 3H), 0.75 (s, 3H); ^{13}C NMR (126 MHz, $CDCl_3$) δ 149.6, 138.4, 137.3, 137.1, 129.0, 127.6, 127.5, 122.7, 119.1, 109.3, 74.5, 45.5, 26.7, 24.7, 21.3; HPLC: the ee value was determined by HPLC analysis (Chiralpak IB, *i*-PrOH/Hexane = 10/90, 1.0 mL/min, 297 nm), retention time: t_{major} = 4.887 min, t_{minor} = 10.693 min, ee = 93%; $[\alpha]_D^{20}$ = – 133.6 (c = 0.22, THF). HRMS *m*/*z* $[M + H]^+$ calculated for $C_{17}H_{20}N$: 238.1590, found 238.1594.

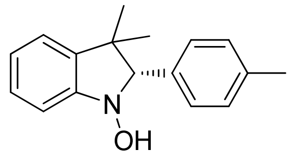

(*S*)-3,3-Dimethyl-2-(*p*-tolyl)indolin-1-ol (2f)

^1H NMR (500 MHz, $CDCl_3$) δ 7.40 (d, *J* = 7.9 Hz, 2H), 7.29–7.19 (m, 3H), 7.13 (d, *J* = 7.1 Hz, 1H), 7.09 (d, *J* = 7.7 Hz, 1H), 7.04 (t, *J* = 7.4 Hz, 1H), 5.63 (s, 1H), 4.28 (s, 1H), 2.40 (s, 3H), 1.42 (s, 3H), 0.81 (s, 3H); ^{13}C NMR (126 MHz, $CDCl_3$) δ 151.3, 137.6, 137.5, 134.2, 129.2, 128.2, 127.8, 123.1, 122.2, 113.6, 85.3, 43.8, 26.0, 25.2, 21.4; HPLC: the ee value was determined by HPLC analysis (Chiralpak IB, *i*-PrOH/Hexane = 5/95, 1.0 mL/min, 252 nm), retention time: t_{major} = 6.790 min, t_{minor} = 8.240 min, ee = 89%; $[\alpha]_D^{20}$ = + 14.6 (c = 0.19, THF). HRMS *m*/*z* $[M + H]^+$ calculated for $C_{17}H_{20}N$: 238.1590, found 238.1594. HRMS *m*/*z* $[M + H]^+$ calculated for $C_{17}H_{20}NO$: 254.1539, found 254,1533.

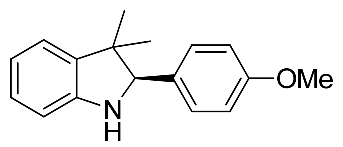

(*R*)-2-(4-Methoxyphenyl)-3,3-dimethylindoline (1g)

It was prepared following the general procedure and purified by silica gel flash chromatography using ethyl acetate/ petroleum ether (10:90) as eluent to afford **1g** (11.1 mg, 44% yield) and **2g** (12.7 mg, 47% yield). ^1H NMR (500 MHz, $CDCl_3$) δ 7.35–7.23 (m, 2H), 7.04–6.91 (m, 2H), 6.88–6.78 (m, 2H), 6.70 (t, *J* = 7.4 Hz, 1H), 6.63 (d, *J* = 7.7 Hz, 1H), 4.46 (s, 1H), 3.97 (brs, 1H), 3.74 (s, 3H), 1.32 (s, 3H), 0.65

(s, 3H); ^{13}C NMR (126 MHz, $CDCl_3$) δ 159.3, 149.5, 138.4, 132.1, 128.7, 127.5, 122.7, 119.1, 113.6, 109.3, 74.3, 55.5, 45.4, 26.6, 24.6; HPLC: the ee value was determined by HPLC analysis (Chiralpak IB, *i*-PrOH/Hexane = 5/95, 1.0 mL/min, 300 nm), retention time: t_{major} = 7.020 min, t_{minor} = 13.177 min, ee = 92%; $[\alpha]_D^{20}$ = – 115.1 (c = 0.22, THF). HRMS *m*/*z* $[M + H]^+$ calculated for $C_{17}H_{20}NO$: 254.1539, found 254.1538.

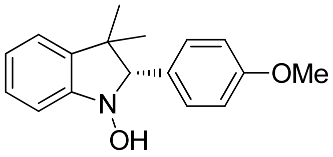

(*S*)-2-(4-Methoxyphenyl)-3,3-dimethylindolin-1-ol (2g)

^{1}H NMR (500 MHz, $CDCl_3$) δ 7.36–7.30 (m, 2H), 7.18–7.14 (m, 1H), 7.04 (d, *J* = 7.1 Hz, 1H), 6.99 (d, *J* = 7.7 Hz, 1H), 6.94 (td, *J* = 7.4, 1.0 Hz, 1H), 6.90–6.79 (m, 2H), 5.79–5.42 (m, 1H), 4.16 (s, 1H), 3.76 (s, 3H), 1.30 (s, 3H), 0.71 (s, 3H); ^{13}C NMR (126 MHz, $CDCl_3$) δ 159.4, 151.3, 137.5, 129.4, 129.2, 127.8, 123.1, 122.2, 113.9, 113.6, 85.0, 55.5, 43.8, 26.0, 25.2; HPLC: the ee value was determined by HPLC analysis (Chiralcel AD-H, *i*-PrOH/Hexane = 30/70, 1.0 mL/min, 224 nm), retention time: t_{major} = 6.073 min, t_{minor} = 11.020 min, ee = 90%; $[\alpha]_D^{20}$ = + 183.1 (c = 0.16, THF). HRMS *m*/*z* $[M + H]^+$ calculated for $C_{17}H_{20}NO_2$: 270.1489, found 270.1495.

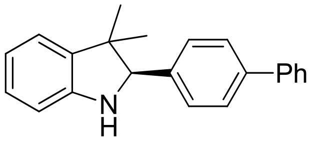

(*R*)-2-([1,1'-Biphenyl]-4-yl)-3,3-dimethylindoline (1h)

It was prepared following the general procedure and purified by silica gel flash chromatography using ethyl acetate/ petroleum ether (10:90) as eluent to afford **1h** (13.5 mg, 45% yield) and **2h** (13.9 mg, 44% yield). ^{1}H NMR (500 MHz, $CDCl_3$) δ 7.67–7.58 (m, 4H), 7.54 (d, *J* = 8.2 Hz, 2H), 7.46 (t, *J* = 7.7 Hz, 2H), 7.37 (t, *J* = 7.4 Hz, 1H), 7.15–7.06 (m, 2H), 6.82 (t, *J* = 7.4 Hz, 1H), 6.76 (d, *J* = 7.7 Hz, 1H), 4.66 (s, 1H), 4.18 (brs, 1H), 1.48 (s, 3H), 0.81 (s, 3H); ^{13}C NMR (126 MHz, $CDCl_3$) δ 149.5, 141.1, 140.6, 139.2, 138.3, 129.0, 128.1, 127.6, 127.4, 127.2, 127.0, 122.7, 119.3, 109.4, 74.5, 45.7, 26.8, 24.8; HPLC: the ee value was determined by HPLC analysis (Chiralpak IB, *i*-PrOH/Hexane = 10/90, 1.0 mL/min, 254 nm), retention time: t_{major} = 6.343 min, t_{minor} = 11.227 min, ee = 98%; $[\alpha]_D^{20}$ = – 112.1 (c = 0.22, THF). HRMS *m*/*z* $[M + H]^+$ calculated for $C_{22}H_{22}N$: 300.1747, found 300.1749.

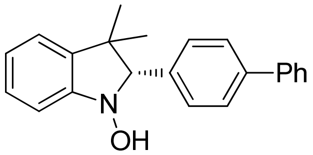

(*S*)-2-([1,1'-Biphenyl]-4-yl)-3,3-dimethylindolin-1-ol (2h)

^{1}H NMR (500 MHz, $CDCl_3$) δ 7.69–7.64 (m, 4H), 7.59 (d, *J* = 8.2 Hz, 2H), 7.49 (t, *J*

= 7.7 Hz, 2H), 7.42–7.36 (m, 1H), 7.29 (td, *J* = 7.7, 1.2 Hz, 1H), 7.17 (d, *J* = 6.9 Hz, 1H), 7.13 (d, *J* = 7.7 Hz, 1H), 7.07 (td, *J* = 7.4, 0.9 Hz, 1H), 5.76 (s, 1H), 4.38 (s, 1H), 1.48 (s, 3H), 0.87 (s, 3H); ^{13}C NMR (126 MHz, $CDCl_3$) δ 151.3, 141.1, 140.8, 137.4, 136.4, 129.0, 128.7, 127.9, 127.5, 127.3, 127.2, 123.2, 122.2, 113.7, 85.2, 44.0, 26.0, 25.3; HPLC: the ee value was determined by HPLC analysis (Chiralpak IB, *i*-PrOH/Hexane = 5/95, 1.0 mL/min, 252 nm), retention time: t_{major} = 11.540 min, t_{minor} = 12.867 min, ee = 94%; $[\alpha]_D^{20}$ = + 150.1 (c = 0.24, THF). HRMS *m*/*z* $[M + H]^+$ calculated for $C_{22}H_{22}NO$: 316.1696, found 316.1692

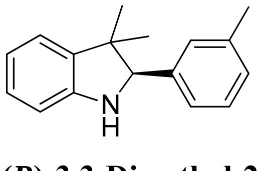

(*R*)-3,3-Dimethyl-2-(*m*-tolyl)indoline (1i)

It was prepared following the general procedure and purified by silica gel flash chromatography using ethyl acetate/ petroleum ether (10:90) as eluent to afford **1i** (10.2 mg, 43% yield) and **2i** (10.6 mg, 42% yield). ^{1}H NMR (500 MHz, $CDCl_3$) δ 7.31–7.24 (m, 3H), 7.17–7.01 (m, 3H), 6.80 (td, *J* = 7.4, 0.9 Hz, 1H), 6.73 (d, *J* = 7.7 Hz, 1H), 4.58 (s, 1H), 4.16 (brs, 1H), 2.38 (s, 3H), 1.44 (s, 3H), 0.75 (s, 3H); ^{13}C NMR (126 MHz, $CDCl_3$) δ 149.4, 140.0, 138.4, 137.9, 128.4, 128.3, 128.2, 127.6, 124.7, 122.7, 119.2, 109.4, 74.7, 45.5, 26.8, 24.7, 21.7; HPLC: the ee value was determined by HPLC analysis (Chiralpak IB, *i*-PrOH/Hexane = 10/90, 1.0 mL/min, 301 nm), retention time: t_{major} = 4.593 min, t_{minor} = 6.703 min, ee = 95%; $[\alpha]_D^{20}$ = – 155.5 (c = 0.20, THF). HRMS *m*/*z* $[M + H]^+$ calculated for $C_{17}H_{20}N$: 238.1590, found 238.1488.

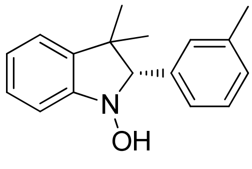

(*S*)-3,3-Dimethyl-2-(*m*-tolyl)indolin-1-ol (2i)

^{1}H NMR (500 MHz, $CDCl_3$) δ 7.36–7.28 (m, 3H), 7.27–7.24 (m, 1H), 7.20–7.15 (m, 1H), 7.16–7.12 (m, 1H), 7.10 (d, *J* = 7.7 Hz, 1H), 7.04 (td, *J* = 7.4, 1.0 Hz, 1H), 5.57 (s, 1H), 4.28 (s, 1H), 2.41 (s, 3H), 1.43 (s, 3H), 0.81 (s, 3H); ^{13}C NMR (126 MHz, $CDCl_3$) δ 151.3, 138.1, 137.5, 137.2, 128.9, 128.7, 128.4, 127.8, 125.3, 123.2, 122.2, 113.6, 85.4, 43.8, 26.1, 25.3, 21.8; HPLC: the ee value was determined by HPLC analysis (Chiralpak IB, *i*-PrOH/Hexane = 5/95, 1.0 mL/min, 247 nm), retention time: t_{major} = 5.910 min, t_{minor} = 8.060 min, ee = 85%; $[\alpha]_D^{20}$ = + 161.7 (c = 0.20, THF). HRMS *m*/*z* $[M + H]^+$ calculated for $C_{17}H_{20}NO$: 254.1539, found 254.1547.

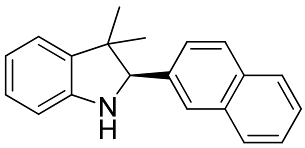

(*R*)-3,3-Dimethyl-2-(naphthalen-2-yl)indoline (1j)

It was prepared following the general procedure and purified by silica gel flash chromatography using ethyl acetate/ petroleum ether (10:90) as eluent to afford **1j** (12.3 mg, 45% yield) and **2j** (13.3 mg, 46% yield). ^{1}H NMR (500 MHz, $CDCl_3$) δ 7.95 (s, 1H), 7.89–7.76 (m, 3H), 7.58 (d, *J* = 8.4 Hz, 1H), 7.54–7.43 (m, 2H), 7.18–7.06 (m, 2H), 6.83 (t, *J* = 7.3 Hz, 1H), 6.79 (d, *J* = 7.7 Hz, 1H), 4.79 (s, 1H), 4.33 (brs, 1H), 1.51 (s, 3H), 0.78 (s, 3H); ^{13}C NMR (126 MHz, $CDCl_3$) δ 149.4, 138.3, 137.8, 133.5, 133.3, 128.1, 127.9, 127.8, 127.7, 126.3, 126.3, 126.0, 125.9, 122.7, 119.3, 109.5, 74.8, 45.8, 27.0, 24.9; HPLC: the ee value was determined by HPLC analysis (Chiralpak IB, *i*-PrOH/Hexane = 10/90, 1.0 mL/min, 301 nm), retention time: t_{major} = 6.303 min, t_{minor} = 15.667 min, ee = 93%; $[\alpha]_D^{20}$ = – 139.0 (c = 0.27, THF). HRMS *m*/*z* $[M + H]^+$ calculated for $C_{20}H_{20}N$: 274.1590, found 274.1593.

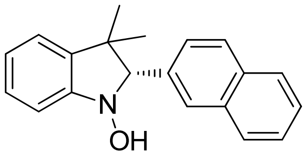

(*S*)-3,3-Dimethyl-2-(naphthalen-2-yl)indolin-1-ol (2j)

^{1}H NMR (500 MHz, $CDCl_3$) δ 7.99 (s, 1H), 7.93–7.86 (m, 3H), 7.66 (dd, *J* = 8.5, 1.6 Hz, 1H), 7.55–7.49 (m, 2H), 7.29 (td, *J* = 7.7, 1.2 Hz, 1H), 7.21–7.12 (m, 2H), 7.07 (td, *J* = 7.4, 1.0 Hz, 1H), 5.73 (s, 1H), 4.50 (s, 1H), 1.50 (s, 3H), 0.85 (s, 3H); ^{13}C NMR (126 MHz, $CDCl_3$) δ 151.3, 137.4, 135.0, 133.5, 133.4, 128.1, 128.1, 127.9, 127.9, 127.2, 126.3, 126.0, 123.3, 122.3, 113.7, 85.6, 44.2, 26.2, 25.4; HPLC: the ee value was determined by HPLC analysis (Chiralcel OD-H, *i*-PrOH/Hexane = 15/85, 1.0 mL/min, 224 nm), retention time: t_{minor} = 12.000 min, t_{major} = 112.873 min, ee = 91%; $[\alpha]_D^{20}$ = + 122.1 (c = 0.20, THF). HRMS *m*/*z* $[M + H]^+$ calculated for $C_{20}H_{20}NO$: 290.1539, found 290.1535.

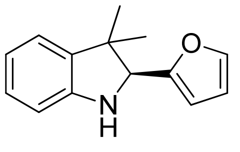

(*S*)-2-(Furan-2-yl)-3,3-dimethylindoline (1k)

It was prepared following the general procedure and purified by silica gel flash chromatography using ethyl acetate/ petroleum ether (15:85) as eluent to afford **1k** (8.9 mg, 42% yield) and **2k** (10.0 mg, 44% yield). ^{1}H NMR (500 MHz, $CDCl_3$) δ 7.44–7.37 (m, 1H), 7.12–7.03 (m, 2H), 6.81 (t, *J* = 7.4 Hz, 1H), 6.73 (d, *J* = 7.7 Hz, 1H), 6.44–6.36 (m, 1H), 6.34 (d, *J* = 3.2 Hz, 1H), 4.63 (s, 1H), 1.49 (s, 3H), 0.92 (s, 3H); ^{13}C NMR (126 MHz, $CDCl_3$) δ 154.8, 148.9, 142.0, 138.2, 127.6, 122.6, 119.6, 110.3, 109.8, 107.0, 68.6, 46.0, 27.1, 24.3; HPLC: the ee value was determined by

HPLC analysis (Chiralpak IB, *i*-PrOH/Hexane = 5/95, 1.0 mL/min, 230 nm), retention time: t_{major} = 5.167 min, t_{minor} = 6.017 min, ee = 93%; $[\alpha]_D^{20}$ = – 35.1 (c = 0.22, THF). HRMS *m*/*z* $[M + H]^+$ calculated for $C_{14}H_{16}NO$: 214.1226, found 214.1225.

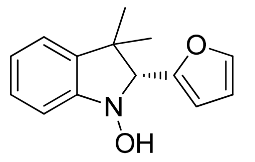

(*R*)-2-(Furan-2-yl)-3,3-dimethylindolin-1-ol (2k)

^{1}H NMR (500 MHz, $CDCl_3$) δ 7.49–7.43 (m, 1H), 7.25 (td, *J* = 7.7, 1.2 Hz, 1H), 7.14–7.10 (m, 1H), 7.09–7.01 (m, 2H), 6.45–6.42 (m, 2H), 6.05 (s, 1H), 4.35 (s, 1H), 1.47 (s, 3H), 0.96 (s, 3H); ^{13}C NMR (126 MHz, $CDCl_3$) δ 152.1, 150.8, 142.5, 137.3, 127.9, 123.6, 122.1, 114.1, 110.5, 108.4, 79.5, 44.2, 26.3, 25.4; HPLC: the ee value was determined by HPLC analysis (Chiralpak IB, *i*-PrOH/Hexane = 5/95, 1.0 mL/min, 245 nm), retention time: t_{major} = 5.703 min, t_{minor} = 6.957 min, ee = 84%; $[\alpha]_D^{20}$ = + 80.1 (c = 0.14, THF). HRMS *m*/*z* $[M + H]^+$ calculated for $C_{14}H_{16}NO_2$: 230.1176, found 230.1171.

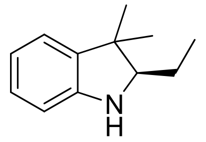

(*R*)-2-Ethyl-3,3-dimethylindoline (1l)

It was prepared following the general procedure and purified by silica gel flash chromatography using ethyl acetate/ petroleum ether (5:95) as eluent to afford **1l** (8.1 mg, 46% yield) and **2l** (9.0 mg, 47% yield). ^{1}H NMR (500 MHz, $CDCl_3$) δ 7.04–6.96 (m, 2H), 6.73 (td, *J* = 7.4, 0.9 Hz, 1H), 6.67–6.60 (m, 1H), 3.92 (brs, 1H), 3.26 (dd, *J* = 9.9, 3.7 Hz, 1H), 1.63–1.50 (m, 2H), 1.30 (s, 3H), 1.07–0.99 (m, 6H); ^{13}C NMR (126 MHz, $CDCl_3$) δ 149.0, 139.8, 127.4, 122.3, 119.3, 109.8, 72.1, 43.6, 26.6, 23.1, 23.0, 12.3; HPLC: the ee value was determined by HPLC analysis (Chiralpak IB, *i*-PrOH/Hexane = 2/98, 1.0 mL/min, 244 nm), retention time: t_{major} = 4.507 min, t_{minor} = 5.417 min, ee = 89%; $[\alpha]_D^{25}$ = + 11.5 (c = 0.55, THF). HRMS *m*/*z* $[M + H]^+$ calculated for $C_{12}H_{18}N$: 176.1434, found 176.1436. The absolute configuration of recovered **5n** was assigned to be *R* by optical rotation data with reported data.[7]

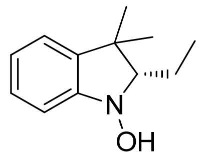

(*S*)-2-Ethyl-3,3-dimethylindolin-1-ol (2l)

^{1}H NMR (500 MHz, CD_3CN) δ 7.18–7.06 (m, 3H), 6.91 (td, *J* = 7.4, 1.0 Hz, 1H), 6.85 (dd, *J* = 7.8, 0.5 Hz, 1H), 2.92 (td, *J* = 6.7, 1.8 Hz, 1H), 1.91–1.83 (m, 1H), 1.77–1.68 (m, 1H), 1.35 (s, 3H), 1.12 (t, *J* = 7.6 Hz, 3H), 1.03 (s, 3H); ^{13}C NMR (126 MHz, CD_3CN) δ 153.4, 139.1, 128.1, 123.1, 122.5, 113.8, 82.6, 42.8, 27.2, 23.7, 22.5, 12.4;

HPLC: the ee value was determined by HPLC analysis (Chiralcel OD-H, *i*-PrOH/Hexane = 3/97, 1.0 mL/min, 248 nm), retention time: t_{minor} = 7.007 min, t_{major} = 7.433 min, ee = 92%; $[\alpha]_D^{25}$ = + 8.8 (c = 0.20, THF). HRMS *m*/*z* $[M + H]^+$ calculated for $C_{12}H_{18}NO$: 192.1383, found 192.1388.

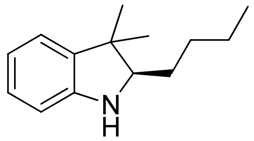

(*R*)-2-Butyl-3,3-dimethylindoline (1m)

It was prepared following the general procedure and purified by silica gel flash chromatography using ethyl acetate/ petroleum ether (5:95) as eluent to afford **1m** (9.1 mg, 45% yield) and **2m** (9.6 mg, 44% yield). ^{1}H NMR (500 MHz, $CDCl_3$) δ 7.10–6.99 (m, 2H), 6.78 (td, *J* = 7.4, 0.9 Hz, 1H), 6.69 (d, *J* = 7.5 Hz, 1H), 3.38–3.34 (m, 1H), 1.61–1.54 (m, 2H), 1.53–1.35 (m, 4H), 1.33 (s, 3H), 1.07 (s, 3H), 0.97 (t, *J* = 7.1 Hz, 3H); ^{13}C NMR (126 MHz, $CDCl_3$) δ 148.9, 139.9, 127.4, 122.3, 119.5, 110.0, 70.4, 43.6, 30.2, 29.7, 26.4, 23.2, 23.1, 14.3; HPLC: the ee value was determined by HPLC analysis (Chiralcel OD-H, *i*-PrOH/Hexane = 2/98, 1.0 mL/min, 249 nm), retention time: t_{major} = 4.927 min, t_{minor} = 6.603 min, ee = 92%; $[\alpha]_D^{25}$ = + 3.6 (c = 0.30, THF). HRMS *m*/*z* $[M + H]^+$ calculated for $C_{14}H_{22}N$: 204.1747, found 204.1742.

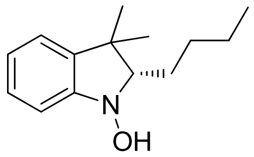

(*S*)-2-Butyl-3,3-dimethylindolin-1-ol (2m)

^{1}H NMR (500 MHz, CD_3CN) δ 7.17–7.05 (m, 2H), 7.02 (brs, 1H), 6.91 (td, *J* = 7.4, 1.1 Hz, 1H), 6.85 (d, *J* = 7.8 Hz, 1H), 2.98 (t, *J* = 6.5 Hz, 1H), 1.87–1.80 (m, 1H), 1.71–1.65 (m, 1H), 1.62–1.49 (m, 2H), 1.43–1.38 (m, 2H), 1.34 (s, 3H), 1.03 (s, 3H), 0.96 (t, *J* = 7.3 Hz, 3H); ^{13}C NMR (126 MHz, CD_3CN) δ 153.4, 139.1, 128.1, 123.1, 122.5, 113.8, 81.0, 42.9, 30.4, 29.4, 27.1, 24.0, 23.8, 14.3; HPLC: the ee value was determined by HPLC analysis (Chiralcel OD-H, *i*-PrOH/Hexane = 2/98, 1.0 mL/min, 253 nm), retention time: t_{major} = 9.633 min, t_{minor} = 10.970 min, ee = 91%; $[\alpha]_D^{25}$ = + 5.1 (c = 0.91, THF). HRMS *m*/*z* $[M + H]^+$ calculated for $C_{14}H_{22}NO$: 220.1696, found 220.1699.

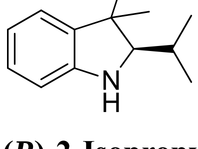

(*R*)-2-Isopropyl-3,3-dimethylindoline (1n)

It was prepared following the general procedure and purified by silica gel flash chromatography using ethyl acetate/ petroleum ether (5:95) as eluent to afford **1n** (8.1 mg, 43% yield) and **2n** (9.4 mg, 46% yield). ^{1}H NMR (500 MHz, $CDCl_3$) δ 7.07–6.96

(m, 2H), 6.77 (t, *J* = 7.2 Hz, 1H), 6.68 (d, *J* = 7.8 Hz, 1H), 3.10 (d, *J* = 9.1 Hz, 1H), 2.07–1.97 (m, 1H), 1.44 (s, 3H), 1.16 (s, 3H), 1.06 (d, *J* = 6.6 Hz, 6H); ^{13}C NMR (126 MHz, $CDCl_3$) δ 148.3, 140.5, 127.4, 122.1, 119.4, 109.7, 76.7, 43.8, 29.6, 28.2, 22.9, 21.7, 20.7; HPLC: the ee value was determined by HPLC analysis (Chiralcel OD-H, *i*-PrOH/Hexane = 3/97, 1.0 mL/min, 303 nm), retention time: t_{major} = 4.520 min, t_{minor} = 8.983 min, ee = 80%; $[\alpha]_D^{25}$ = + 31.2 (c = 0.45, THF). HRMS *m*/*z* $[M + H]^+$ calculated for $C_{13}H_{20}N$: 190.1590, found 190.1591.

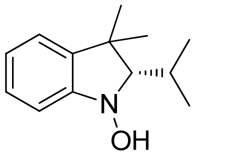

(*S*)-2-Isopropyl-3,3-dimethylindolin-1-ol (2n)

^1H NMR (500 MHz, CD_3CN) δ 7.14 (td, *J* = 7.6, 1.2 Hz, 1H), 7.08 (d, *J* = 7.4 Hz, 1H), 7.01–6.96 (m, 1H), 6.92 (td, *J* = 7.4, 1.0 Hz, 1H), 6.87 (dd, *J* = 7.8, 0.4 Hz, 1H), 2.87 (d, *J* = 5.3 Hz, 1H), 2.13–2.06 (m, 1H), 1.37 (s, 3H), 1.18 (d, *J* = 6.8 Hz, 3H), 1.13 (d, *J* = 7.0 Hz, 3H), 1.08 (s, 3H); ^{13}C NMR (126 MHz, CD_3CN) δ 153.7, 139.3, 128.1, 123.2, 122.5, 114.4, 85.7, 43.7, 28.4, 28.2, 24.9, 23.3, 20.7; HPLC: the ee value was determined by HPLC analysis (Chiralpak IG, *i*-PrOH/Hexane = 3/97, 1.0 mL/min, 250 nm), retention time: t_{minor} = 7.783 min, t_{major} = 8.957 min, ee = 82%; $[\alpha]_D^{25}$ = – 12.9 (c = 0.95, THF). HRMS *m*/*z* $[M + H]^+$ calculated for $C_{13}H_{20}NO$: 206.1539, found 206.1534.

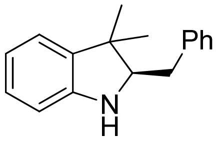

(*R*)-2-Benzyl-3,3-dimethylindoline (1o)

It was prepared following the general procedure and purified by silica gel flash chromatography using ethyl acetate/ petroleum ether (5:95) as eluent to afford **1o** (10.7 mg, 45% yield) and **2o** (11.6 mg, 46% yield). ^1H NMR (500 MHz, $CDCl_3$) δ 7.36 (t, *J* = 7.5 Hz, 2H), 7.33–7.26 (m, 3H), 7.07 (d, *J* = 7.3 Hz, 1H), 7.03 (t, *J* = 7.6 Hz, 1H), 6.78 (t, *J* = 7.4 Hz, 1H), 6.61 (d, *J* = 7.6 Hz, 1H), 3.62 (dd, *J* = 10.9, 3.1 Hz, 1H), 2.93 (dd, *J* = 13.2, 3.0 Hz, 1H), 2.81 – 2.73 (m, 1H), 1.39 (s, 3H), 1.22 (s, 3H); ^{13}C NMR (126 MHz, $CDCl_3$) δ 148.5, 140.0, 139.4, 129.3, 128.9, 127.5, 126.6, 122.3, 119.5, 110.0, 71.6, 43. 7, 36.5, 26.5, 23.1; HPLC: the ee value was determined by HPLC analysis (Chiralcel OD-H, *i*-PrOH/Hexane = 5/95, 1.0 mL/min, 251 nm), retention time: t_{minor} = 5.100 min, t_{major} = 5.640 min, ee = 94%; $[\alpha]_D^{25}$ = + 24.1 (c = 0.55, THF). HRMS *m*/*z* $[M + H]^+$ calculated for $C_{17}H_{20}N$: 238.1590, found 238.1587.

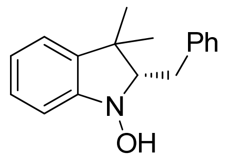

(*S*)-2-Benzyl-3,3-dimethylindolin-1-ol (2o)

^{1}H NMR (500 MHz, CD_3CN) δ 7.39–7.34 (m, *J* = 7.3 Hz, 2H), 7.31 (t, *J* = 7.6 Hz, 2H), 7.22 (t, *J* = 7.3 Hz, 1H), 7.16–7.11 (m, 1H), 7.06 (d, *J* = 6.9 Hz, 1H), 7.02–6.96 (m, 1H), 6.91 (td, *J* = 7.4, 1.0 Hz, 1H), 6.87 (d, *J* = 7.8 Hz, 1H), 3.41–3.33 (m, 1H), 3.27 (dd, *J* = 14.0, 5.5 Hz, 1H), 2.97 (dd, *J* = 13.9, 8.5 Hz, 1H), 1.14 (s, 3H), 1.00 (s, 3H); ^{13}C NMR (126 MHz, CD_3CN) δ 153.0, 140.8, 139.1, 130.3, 129.2, 128.2, 126.9, 123.3, 122.4, 113.9, 82.5, 43.0, 35.3, 26.7, 24.1; HPLC: the ee value was determined by HPLC analysis (Chiralpak AS-H, *i*-PrOH/Hexane = 5/95, 1.0 mL/min, 252 nm), retention time: t_{minor} = 6.863 min, t_{major} = 7.787 min, ee = 91%; $[\alpha]_D^{25}$ = – 17.1 (c = 0.45, THF). HRMS *m*/*z* $[M + H]^+$ calculated for $C_{17}H_{20}NO$: 254.1539, found 254.1530.

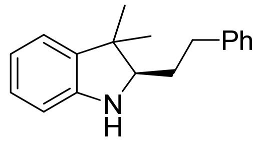

(*R*)-3,3-Dimethyl-2-phenethylindoline (1p)

It was prepared following the general procedure and purified by silica gel flash chromatography using ethyl acetate/ petroleum ether (5:95) as eluent to afford **1p** (10.5 mg, 42% yield) and **2p** (13.1 mg, 49% yield). ^{1}H NMR (500 MHz, $CDCl_3$) δ 7.33 (t, *J* = 7.5 Hz, 2H), 7.29–7.22 (m, 3H), 7.09–6.98 (m, 2H), 6.78 (t, *J* = 7.2 Hz, 1H), 6.67 (d, *J* = 7.4 Hz, 1H), 3.42 (dd, *J* = 8.6, 4.8 Hz, 1H), 2.91–2.82 (m, 1H), 2.73–2.66 (m, 1H), 1.95–1.87 (m, 2H), 1.33 (s, 3H), 1.07 (s, 3H); ^{13}C NMR (126 MHz, $CDCl_3$) δ 148.9, 142.1, 139.6, 128.7, 128.6, 127.4, 126.3, 122.3, 119.5, 110.0, 70.0, 43.8, 34.5, 32.1, 26.3, 23.2; HPLC: the ee value was determined by HPLC analysis (Chiralpak AD-H, *i*-PrOH/Hexane = 5/95, 1.0 mL/min, 250 nm), retention time: t_{major} = 6.303 min, t_{minor} = 8.397 min, ee = 96%; $[\alpha]_D^{20}$ = + 9.8 (c = 1.0, THF). HRMS *m*/*z* $[M + H]^+$ calculated for $C_{18}H_{22}N$: 252.1747, found 252.1751.

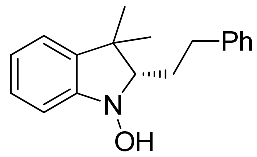

(*S*)-3,3-Dimethyl-2-phenethylindolin-1-ol (2p)

^{1}H NMR (500 MHz, CD_3CN) δ 7.38–7.25 (m, 5H), 7.24–7.17 (m, 1H), 7.14 (t, *J* = 7.6 Hz, 1H), 7.10 (d, *J* = 7.4 Hz, 1H), 6.96–6.90 (m, 1H), 6.90–6.84 (m, 1H), 3.11–3.03 (m, 1H), 3.02–2.94 (m, 1H), 2.87–2.80 (m, 1H), 2.17–2.07 (m, 1H), 2.04–1.95 (m, 1H), 1.34 (s, 3H), 1.06 (s, 3H); ^{13}C NMR (126 MHz, CD_3CN) δ 153.3, 143.9, 138.9, 129.3, 129.3, 128.1, 126.6, 123.2, 122.5, 113.9, 80.4, 42.9, 34.4, 32.0, 26.9, 23.9; HPLC: the ee value was determined by HPLC analysis (Chiralpak AD-H,

i-PrOH/Hexane = 15/85, 1.0 mL/min, 251 nm), retention time: t_{major} = 8.457 min, t_{minor} = 9.777 min, ee = 88%; $[\alpha]_D^{20}$ = – 14.2 (c = 0.5, THF). HRMS *m*/*z* $[M + H]^+$ calculated for $C_{18}H_{22}NO$: 268.1696, found 268.1699.

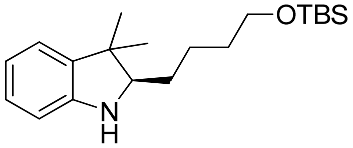

(*R*)-2-(4-((*Tert*-butyldimethylsilyl)oxy)butyl)-3,3-dimethylindoline (1q)

It was prepared following the general procedure and purified by silica gel flash chromatography using ethyl acetate/ petroleum ether (5:95) as eluent to afford **1q** (14.7 mg, 44% yield) and **2q** (17.5 mg, 50% yield). ^1H NMR (500 MHz, $CDCl_3$) δ 7.10–6.99 (m, 2H), 6.77 (t, *J* = 7.4 Hz, 1H), 6.67 (d, *J* = 7.6 Hz, 1H), 3.71–3.65 (m, *J* = 6.0, 2.7 Hz, 2H), 3.40–3.35 (m, 1H), 1.68–1.54 (m, 5H), 1.47–1.40 (m, 1H), 1.33 (s, 3H), 1.06 (s, 3H), 0.93 (d, *J* = 1.2 Hz, 9H), 0.06 (d, *J* = 21.0 Hz, 6H); ^{13}C NMR (126 MHz, $CDCl_3$) δ 149.0, 139.8, 127.4, 122.3, 119.3, 109.9, 70.3, 63.2, 43.6, 33.2, 29.8, 26.5, 26.2, 24.2, 23.1, 18.6, -5.0, -5.0; HPLC: the ee value was determined by HPLC analysis (Chiralpak IB, *i*-PrOH/Hexane = 1/99, 1.0 mL/min, 296 nm), retention time: t_{major} = 5.030 min, t_{minor} = 6.407 min, ee = 95%; $[\alpha]_D^{20}$ = + 15.0 (c = 0.2, THF). HRMS *m*/*z* $[M + H]^+$ calculated for $C_{20}H_{36}NOSi$: 334.2561, found 334.2556.

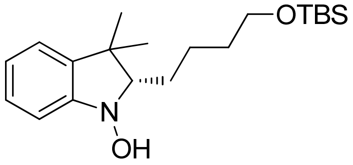

(*S*)-2-(4-((*Tert*-butyldimethylsilyl)oxy)butyl)-3,3-dimethylindolin-1-ol (2q)

^1H NMR (500 MHz, CD_3CN) δ 7.13 (td, *J* = 7.6, 1.2 Hz, 1H), 7.09 (d, *J* = 7.4 Hz, 1H), 7.04–6.99 (m, 1H), 6.91 (td, *J* = 7.4, 1.0 Hz, 1H), 6.85 (d, *J* = 7.8 Hz, 1H), 3.68 (t, *J* = 6.0 Hz, 2H), 2.98 (t, *J* = 6.5 Hz, 1H), 1.88–1.79 (m, 1H), 1.73–1.65 (m, 1H), 1.64–1.55 (m, 4H), 1.34 (s, 3H), 1.03 (s, 3H), 0.91 (s, 9H), 0.07 (s, 6H); ^{13}C NMR (126 MHz, CD_3CN) δ 153.4, 139.1, 128.2, 123.1, 122.5, 113.8, 81.0, 63.6, 42.9, 34.2, 29.5, 27.1, 26.3, 24.5, 23.8, 18.9, -5.1; HPLC: the ee value was determined by HPLC analysis (Chiralpak IG, *i*-PrOH/Hexane = 1/99, 1.0 mL/min, 248 nm), retention time: t_{minor} = 8.460 min, t_{major} = 8.913 min, ee = 87%; $[\alpha]_D^{20}$ = – 1.5 (c = 0.1, THF). HRMS *m*/*z* $[M + H]^+$ calculated for $C_{20}H_{36}NO_2Si$: 350.2510, found 350.2508.

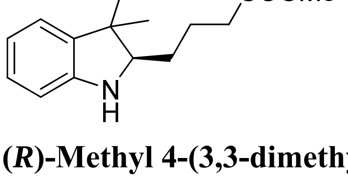

(*R*)-Methyl 4-(3,3-dimethylindolin-2-yl)butanoate (1r)

It was prepared following the general procedure and purified by silica gel flash chromatography using ethyl acetate/ petroleum ether (15:85) as eluent to afford **1r** (9.9 mg, 40% yield) and **2r** (11.8 mg, 45% yield). ^1H NMR (500 MHz, $CDCl_3$) δ 7.03

(t, *J* = 6.8 Hz, 2H), 6.76 (td, *J* = 7.4, 0.8 Hz, 1H), 6.65 (d, *J* = 7.8 Hz, 1H), 4.04 (brs, 1H), 3.71 (s, 3H), 3.42–3.31 (m, 1H), 2.44–2.39 (m, 2H), 1.89–1.80 (m, 1H), 1.77–1.69 (m, 1H), 1.64–1.55 (m, 2H), 1.33 (s, 3H), 1.06 (s, 3H); ^{13}C NMR (126 MHz, $CDCl_3$) δ 174.0, 149.1, 139.4, 127.4, 122.2, 119.2, 109.7, 69.8, 51.7, 43.6, 34.3, 29.7, 26.4, 23.2, 23.1; HPLC: the ee value was determined by HPLC analysis (Chiralpak AD, *i*-PrOH/Hexane = 5/95, 1.0 mL/min, 295 nm), retention time: t_{major} = 9.200 min, t_{minor} = 10.873 min, ee = 91%; $[\alpha]_D^{20}$ = + 2.8 (c = 0.5, THF). HRMS *m*/*z* $[M + H]^+$ calculated for $C_{15}H_{22}NO_2$: 248.1645, found 248.1655.

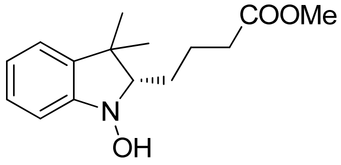

(*S*)-Methyl 4-(1-hydroxy-3,3-dimethylindolin-2-yl)butanoate (2r)

^{1}H NMR (500 MHz, CD_3CN) δ 7.13 (td, *J* = 7.6, 1.2 Hz, 1H), 7.11–7.05 (m, 2H), 6.91 (td, *J* = 7.4, 0.9 Hz, 1H), 6.85 (d, *J* = 7.8 Hz, 1H), 3.64 (s, 3H), 2.99 (t, *J* = 6.0 Hz, 1H), 2.40 (t, *J* = 7.1 Hz, 2H), 1.92–1.85 (m, 1H), 1.85–1.77 (m, 2H), 1.74–1.66 (m, 1H), 1.34 (s, 3H), 1.01 (s, 3H); ^{13}C NMR (126 MHz, CD_3CN) δ 174.7, 153.4, 139.0, 128.2, 123.1, 122.5, 113.8, 80.5, 51.9, 42.9, 34.9, 29.1, 26.9, 23.8, 23.7; HPLC: the ee value was determined by HPLC analysis (Chiralpak IC, *i*-PrOH/Hexane = 10/90, 1.0 mL/min, 288 nm), retention time: t_{minor} = 7.517 min, t_{major} = 8.510 min, ee = 88%; $[\alpha]_D^{20}$ = – 0.8 (c = 0.3, THF). HRMS *m*/*z* $[M + H]^+$ calculated for $C_{15}H_{22}NO_3$: 264.1594, found 264.1599.

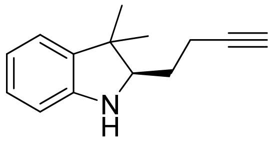

(*R*)-2-(But-3-yn-1-yl)-3,3-dimethylindoline (1s)

It was prepared following the general procedure and purified by silica gel flash chromatography using ethyl acetate/ petroleum ether (5:95) as eluent to afford **1s** (9.4 mg, 47% yield) and **2s** (9.7 mg, 45% yield). ^{1}H NMR (500 MHz, $CDCl_3$) δ 7.05 (t, *J* = 6.9 Hz, 2H), 6.78 (t, *J* = 7.3 Hz, 1H), 6.68 (d, *J* = 7.8 Hz, 1H), 4.23 (brs, 1H), 3.61–3.49 (m, 1H), 2.48–2.32 (m, 2H), 2.04 (t, *J* = 2.6 Hz, 1H), 1.86–1.71 (m, 2H), 1.35 (s, 3H), 1.07 (s, 3H); ^{13}C NMR (126 MHz, $CDCl_3$) δ 149.1, 139.3, 127.5, 122.3, 119.4, 109.9, 83.9, 69.5, 69.1, 43.7, 28.6, 26.3, 23.2, 17.0; HPLC: the ee value was determined by HPLC analysis (Chiralpak IB, *i*-PrOH/Hexane = 5/95, 1.0 mL/min, 297 nm), retention time: t_{major} = 6.540 min, t_{minor} = 9.193 min, ee = 95%; $[\alpha]_D^{20}$ = + 5.7 (c = 1.0, THF). HRMS *m*/*z* $[M + H]^+$ calculated for $C_{14}H_{18}N$: 200.1434, found 200.1430.

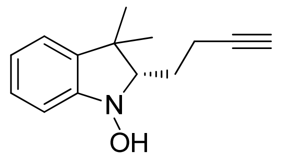

(*S*)-2-(But-3-yn-1-yl)-3,3-dimethylindolin-1-ol (2s)

^{1}H NMR (500 MHz, CD_3CN) δ 7.14 (td, *J* = 7.6, 1.0 Hz, 2H), 7.10 (d, *J* = 7.4 Hz, 1H), 6.93 (td, *J* = 7.4, 1.0 Hz, 1H), 6.86 (d, *J* = 7.8 Hz, 1H), 3.13–3.05 (m, 1H), 2.57–2.43 (m, 2H), 2.07–1.98 (m, 1H), 1.92–1.84 (m, 1H), 1.34 (s, 3H), 1.02 (s, 3H); ^{13}C NMR (126 MHz, CD_3CN) δ 153.3, 138.8, 128.2, 123.3, 122.5, 113.9, 85.4, 79.4, 69.9, 42.8, 29.0, 26.6, 23.9, 17.3; HPLC: the ee value was determined by HPLC analysis (Chiralpak IG, *i*-PrOH/Hexane = 5/95, 1.0 mL/min, 300 nm), retention time: t_{minor} = 13.833 min, t_{major} = 14.353 min, ee = 90%; $[\alpha]_D^{20}$ = – 4.1 (c = 0.5, THF). HRMS *m*/*z* $[M + H]^+$ calculated for $C_{14}H_{18}NO$: 216.1383, found 216.1374.

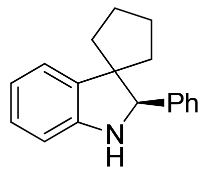

(*R*)-2'-Phenylspiro[cyclopentane-1,3'-indoline] (1t)

It was prepared following the general procedure and purified by silica gel flash chromatography using ethyl acetate/ petroleum ether (10:90) as eluent to afford **1t** (11.0 mg, 44% yield) and **2t** (11.1 mg, 42% yield). ^{1}H NMR (500 MHz, $CDCl_3$) δ 7.39 (d, *J* = 7.6 Hz, 2H), 7.37–7.28 (m, 3H), 7.14–6.99 (m, 2H), 6.80 (t, *J* = 7.4 Hz, 1H), 6.71 (d, *J* = 7.6 Hz, 1H), 4.66 (s, 1H), 4.18 (brs, 1H), 2.07–1.98 (m, 2H), 1.89–1.79 (m, 1H), 1.73–1.62 (m, 2H), 1.49–1.43 (m, 2H), 1.29–1.20 (m, 1H); ^{13}C NMR (126 MHz, $CDCl_3$) δ 150.0, 141.3, 138.2, 128.4, 127.8, 127.5, 123.0, 119.2, 108.9, 73.9, 57.5, 39.9, 35.1, 24.8, 24.8; HPLC: the ee value was determined by HPLC analysis (Chiralpak IB, *i*-PrOH/Hexane = 10/90, 1.0 mL/min, 304 nm), retention time: t_{major} = 6.177 min, t_{minor} = 9.530 min, ee = 85%; $[\alpha]_D^{20}$ = – 7.2 (c = 0.18, THF). HRMS *m*/*z* $[M + H]^+$ calculated for $C_{18}H_{20}N$: 250.1590, found 250.1488.

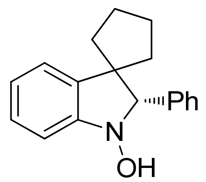

(*S*)-2'-Phenylspiro[cyclopentane-1,3'-indolin]-1'-ol (2t)

^{1}H NMR (500 MHz, $CDCl_3$) δ 7.53 (d, *J* = 7.1 Hz, 2H), 7.47–7.39 (m, 2H), 7.39–7.34 (m, 1H), 7.28–7.24 (m, 1H), 7.15 (d, *J* = 7.4 Hz, 1H), 7.08 (d, *J* = 7.7 Hz, 1H), 7.04 (t, *J* = 7.4 Hz, 1H), 5.64 (d, *J* = 15.4 Hz, 1H), 4.49 (s, 1H), 2.23–2.17 (m, 1H), 1.94 (ddd, *J* = 13.5, 9.4, 7.5 Hz, 1H), 1.85–1.76 (m, 1H), 1.64 – 1.56 (m, 2H), 1.36–1.32 (m, 1H), 1.19–1.12 (m, 1H), 0.93 – 0.83 (m, 1H); ^{13}C NMR (126 MHz, $CDCl_3$) δ 151.4, 138.1, 137.5, 128.8, 128.5, 128.0, 127.6, 123.2, 122.6, 113.3, 84.3, 54.6, 37.3, 35.5, 25.0,

24.5; HPLC: the ee value was determined by HPLC analysis (Chiralpak IB, *i*-PrOH/Hexane = 5/95, 1.0 mL/min, 247 nm), retention time: t_{major} = 6.987 min, t_{minor} = 12.137 min, ee = 84%; $[\alpha]_D^{20}$ = + 11.2 (c = 0.30, THF). HRMS *m*/*z* $[M + H]^+$ calculated for $C_{18}H_{20}NO$: 266.1539, found 266.1536.

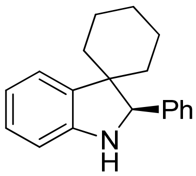

(*R*)-2'-Phenylspiro[cyclohexane-1,3'-indoline] (1u)

It was prepared following the general procedure and purified by silica gel flash chromatography using ethyl acetate/ petroleum ether (10:90) as eluent to afford **1u** (10.8 mg, 41% yield) and **2u** (12.3 mg, 44% yield). ^{1}H NMR (500 MHz, $CDCl_3$) δ 7.31–7.14 (m, 6H), 6.99 (td, *J* = 7.6, 1.2 Hz, 1H), 6.67 (td, *J* = 7.4, 0.9 Hz, 1H), 6.57 (d, *J* = 7.7 Hz, 1H), 4.47 (s, 1H), 3.82 (brs, 1H), 1.80–1.70 (m, 2H), 1.67–1.57 (m, 2H), 1.51–1.44 (m, 1H), 1.44–1.28 (m, 3H), 1.15–1.00 (m, 2H); ^{13}C NMR (126 MHz, $CDCl_3$) δ 150.2, 141.4, 137.3, 128.3, 128.1, 127.8, 127.6, 124.5, 118.6, 108.9, 73.2, 49.4, 37.5, 32.0, 25.9, 23.2, 22.4; HPLC: the ee value was determined by HPLC analysis (Chiralpak IB, *i*-PrOH/Hexane = 10/90, 1.0 mL/min, 248 nm), retention time: t_{major} = 5.393 min, t_{minor} = 6.890 min, ee = 87%; $[\alpha]_D^{20}$ = + 118.7 (c = 0.11, THF). HRMS *m*/*z* $[M + H]^+$ calculated for $C_{19}H_{22}N$: 264.1747, found 264.1748.

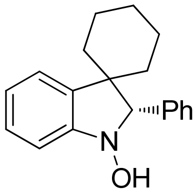

(*S*)-2'-Phenylspiro[cyclohexane-1,3'-indolin]-1'-ol (2u)

^{1}H NMR (500 MHz, $CDCl_3$) δ 7.55 (d, *J* = 7.5 Hz, 1H), 7.49 (d, *J* = 7.0 Hz, 2H), 7.48–7.34 (m, 3H), 7.29 (t, *J* = 7.6 Hz, 1H), 7.11 (d, *J* = 7.4 Hz, 1H), 7.03 (td, *J* = 7.5, 1.0 Hz, 1H), 5.64 (s, 1H), 4.24 (s, 1H), 2.00 (dd, *J* = 18.3, 7.8 Hz, 2H), 1.80 – 1.65 (m, 3H), 1.54–1.42 (m, 2H), 1.39–1.32 (m, 1H), 1.15 – 0.97 (m, 2H); ^{13}C NMR (126 MHz, $CDCl_3$) δ 152.0, 137.3, 136.5, 129.2, 128.4, 128.0, 127.7, 125.3, 122.5, 113.6, 86.3, 47.1, 35.3, 33.0, 25.7, 23.5, 21.5; HPLC: the ee value was determined by HPLC analysis (Chiralpak IB, *i*-PrOH/Hexane = 10/90, 1.0 mL/min, 250 nm), retention time: t_{major} = 5.180 min, t_{minor} = 7.763 min, ee = 79%; $[\alpha]_D^{20}$ = – 85.5 (c = 0.10, THF). HRMS *m*/*z* $[M + H]^+$ calculated for $C_{19}H_{22}N$: 280.1696, found 280.1691.

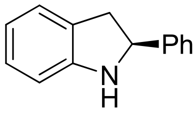

(*S*)-2-Phenylindoline (1v)

It was prepared following the general procedure and purified by silica gel flash

chromatography using ethyl acetate/ petroleum ether (10:90) as eluent to afford **1v** (8.6 mg, 44% yield). ^{1}H NMR (500 MHz, $CDCl_3$) δ 7.60–7.50 (m, 2H), 7.47–7.36 (m, 3H), 7.24 (t, *J* = 7.6 Hz, 1H), 7.16 (d, *J* = 7.3 Hz, 1H), 7.01 (t, *J* = 7.4 Hz, 1H), 6.91 (d, *J* = 7.7 Hz, 1H), 6.03 (brs, 1H), 4.56 (dd, *J* = 11.7, 8.2 Hz, 1H), 3.34 (dd, *J* = 15.2, 8.2 Hz, 1H), 2.94 (dd, *J* = 15.0, 11.9 Hz, 1H); ^{13}C NMR (126 MHz, $CDCl_3$) δ 152.9, 140.7, 128.8, 128.1, 128.0, 127.9, 127.3, 124.4, 123.1, 113.7, 76.5, 37.3; HPLC: the ee value was determined by HPLC analysis (Chiralpak IB, *i*-PrOH/Hexane = 10/90, 1.0 mL/min, 248 nm), retention time: t_{major} = 7.933 min, t_{minor} = 11.813 min, ee = 86%; $[\alpha]_D^{20}$ = + 45.4 (c = 0.12, THF). HRMS *m*/*z* $[M + H]^+$ calculated for $C_{14}H_{14}N$: 196.1121, found 196.1127.

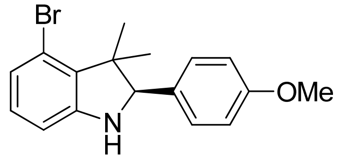

(*R*)-4-bromo-2-(4-methoxyphenyl)-3,3-dimethylindoline (4a)

It was prepared following the general procedure and purified by silica gel flash chromatography using ethyl acetate/ petroleum ether (15:85) as eluent to afford **4a** (15.9 mg, 48% yield) and **5a** (15.3 mg, 44% yield). ^{1}H NMR (500 MHz, $CDCl_3$) δ 7.38 (d, *J* = 8.6 Hz, 2H), 6.98–6.80 (m, 4H), 6.62 (dd, *J* = 7.2, 1.2 Hz, 1H), 4.53 (s, 1H), 4.16 (brs, 1H), 3.84 (s, 3H), 1.58 (s, 3H), 0.86 (s, 3H); ^{13}C NMR (126 MHz, $CDCl_3$) δ 159.5, 151.7, 134.6, 131.1, 129.2, 129.1, 123.8, 119.6, 113.7, 108.3, 74.0, 55.5, 47.8, 25.7, 21.7; HPLC: the ee value was determined by HPLC analysis (Chiralpak IB, *i*-PrOH/Hexane = 10/90, 1.0 mL/min, 255 nm), retention time: t_{major} = 6.800 min, t_{minor} = 11.923 min, ee = 89%; $[\alpha]_D^{20}$ = – 53.6 (c = 0.24, THF). HRMS *m*/*z* $[M + H]^+$ calculated for $C_{17}H_{18}BrNO$: 334.0630, found 334.0635.

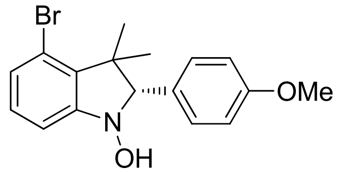

(*S*)-4-bromo-2-(4-methoxyphenyl)-3,3-dimethylindolin-1-ol (5a)

^{1}H NMR (500 MHz, $CDCl_3$) δ 7.41 (d, *J* = 8.6 Hz, 2H), 7.14 (dd, *J* = 7.9, 1.1 Hz, 1H), 7.09 (t, *J* = 7.8 Hz, 1H), 7.03 (dd, *J* = 7.6, 1.1 Hz, 1H), 6.96 (d, *J* = 8.7 Hz, 2H), 5.62 (s, 1H), 4.23 (s, 1H), 3.85 (s, 3H), 1.58 (s, 3H), 0.91 (s, 3H); ^{13}C NMR (126 MHz, $CDCl_3$) δ 159.7, 153.6, 134.0, 129.8, 129.2, 128.4, 127.8, 118.6, 113.9, 112.7, 84.1, 55.5, 45.8, 25.4, 21.7; HPLC: the ee value was determined by HPLC analysis (Chiralpak IB, *i*-PrOH/Hexane = 10/90, 1.0 mL/min, 252 nm), retention time: t_{minor} = 8.283 min, t_{major} = 9.547 min, ee = 96%; $[\alpha]_D^{20}$ = + 137.5 (c = 0.18, THF). HRMS *m*/*z* $[M + H]^+$ calculated for $C_{17}H_{18}BrNO_2$: 350.0579, found 350.0572.

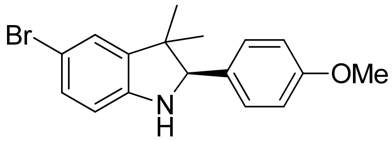

(*R*)-5-bromo-2-(4-methoxyphenyl)-3,3-dimethylindoline (4b)

It was prepared following the general procedure and purified by silica gel flash chromatography using ethyl acetate/ petroleum ether (15:85) as eluent to afford **4b** (14.6 mg, 44% yield) and **5b** (16.4 mg, 47% yield). ^{1}H NMR (500 MHz, $CDCl_3$) δ 7.34 (d, *J* = 8.7 Hz, 2H), 7.16 (dd, *J* = 8.2, 2.0 Hz, 1H), 7.12 (d, *J* = 2.0 Hz, 1H), 6.93–6.86 (m, 2H), 6.58 (d, *J* = 8.2 Hz, 1H), 4.55 (s, 1H), 4.10 (brs, 1H), 3.83 (s, 3H), 1.39 (s, 3H), 0.74 (s, 3H); ^{13}C NMR (126 MHz, $CDCl_3$) δ 159.4, 148.5, 140.8, 131.5, 130.1, 128.6, 125.9, 113.7, 110.7, 110.6, 74.5, 55.5, 45.8, 26.6, 24.5; HPLC: the ee value was determined by HPLC analysis (Chiralpak IB, *i*-PrOH/Hexane = 10/90, 1.0 mL/min, 257 nm), retention time: t_{major} = 6.077 min, t_{minor} = 17.343 min, ee = 97%; $[\alpha]_D^{20}$ = – 50.4 (c = 0.27, THF). HRMS *m*/*z* $[M + H]^+$ calculated for $C_{17}H_{18}BrNO$: 334.0630, found 334.0638.

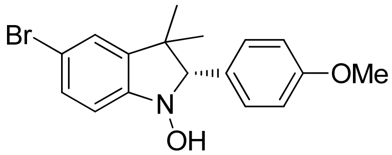

(*S*)-5-bromo-2-(4-methoxyphenyl)-3,3-dimethylindolin-1-ol (5b)

^{1}H NMR (500 MHz, $CDCl_3$) δ 7.41–7.36 (m, 2H), 7.34 (dd, *J* = 8.3, 1.9 Hz, 1H), 7.21 (d, *J* = 1.9 Hz, 1H), 6.99–6.91 (m, 3H), 5.63 (s, 1H), 4.25 (s, 1H), 3.85 (s, 3H), 1.37 (s, 3H), 0.80 (s, 3H); ^{13}C NMR (126 MHz, $CDCl_3$) δ 159.6, 150.4, 139.7, 130.6, 129.3, 128.6, 125.5, 115.5, 115.2, 114.0, 85.0, 55.5, 43.9, 25.8, 25.1; HPLC: the ee value was determined by HPLC analysis (Chiralpak IB, *i*-PrOH/Hexane = 10/90, 1.0 mL/min, 257 nm), retention time: t_{minor} = 8.290 min, t_{major} = 8.877 min, ee = 93%; $[\alpha]_D^{20}$ = + 143.0 (c = 0.36, THF). HRMS *m*/*z* $[M + H]^+$ calculated for $C_{17}H_{18}BrNO_2$: 350.0579, found 350.0583.

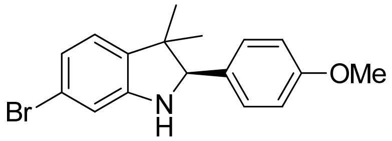

(*R*)-6-bromo-2-(4-methoxyphenyl)-3,3-dimethylindoline (4c)

It was prepared following the general procedure and purified by silica gel flash chromatography using ethyl acetate/ petroleum ether 15:85) as eluent to afford **4c** (13.9 mg, 42% yield) and **5c** (16.7 mg, 48% yield). ^{1}H NMR (500 MHz, $CDCl_3$) δ 7.33 (d, *J* = 8.6 Hz, 2H), 6.97–6.86 (m, 4H), 6.83 (s, 1H), 4.55 (s, 1H), 4.12 (s, 1H), 3.83 (s, 3H), 1.38 (s, 3H), 0.72 (s, 3H); ^{13}C NMR (126 MHz, $CDCl_3$) δ 159.4, 151.0, 137.4, 131.5, 128.5, 124.0, 121.7, 120.9, 113.7, 112.2, 74.4, 55.5, 45.2, 26.7, 24.5; HPLC: the ee value was determined by HPLC analysis (Chiralpak IB, *i*-PrOH/Hexane = 10/90, 1.0 mL/min, 255 nm), retention time: t_{major} = 5.913 min, t_{minor} = 11.130 min,

ee = 99%; $[\alpha]_D^{20}$ = – 146.2 (c = 0.26, THF). HRMS *m*/*z* $[M + H]^+$ calculated for $C_{17}H_{18}BrNO$: 334.0630, found 334.0624.

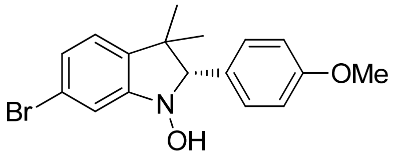

(*S*)-6-bromo-2-(4-methoxyphenyl)-3,3-dimethylindolin-1-ol (5c)

^{1}H NMR (500 MHz, $CDCl_3$) δ 7.38 (d, *J* = 8.6 Hz, 2H), 7.21 (d, *J* = 1.7 Hz, 1H), 7.14 (dd, *J* = 7.9, 1.8 Hz, 1H), 7.01–6.90 (m, 3H), 5.67 (s, 1H), 4.26 (s, 1H), 3.85 (s, 3H), 1.37 (s, 3H), 0.79 (s, 3H); ^{13}C NMR (126 MHz, $CDCl_3$) δ 159.6, 152.7, 136.5, 129.3, 128.6, 126.0, 123.7, 121.2, 116.8, 114.0, 84.9, 55.5, 43.6, 25.8, 25.0; HPLC: the ee value was determined by HPLC analysis (Chiralpak IB, *i*-PrOH/Hexane = 10/90, 1.0 mL/min, 228 nm), retention time: t_{minor} = 7.647 min, t_{major} = 11.443 min, ee = 88%; $[\alpha]_D^{20}$ = + 171.2 (c = 0.36, THF). HRMS *m*/*z* $[M + H]^+$ calculated for $C_{17}H_{18}BrNO_2$: 350.0579, found 350.0586.

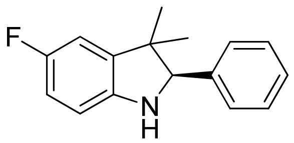

(*R*)-5-fluoro-3,3-dimethyl-2-phenylindoline (4d)

It was prepared following the general procedure and purified by silica gel flash chromatography using ethyl acetate/ petroleum ether (15:85) as eluent to afford **4d** (10.4 mg, 43% yield) and **5d** (12.1 mg, 47% yield). ^{1}H NMR (500 MHz, $CDCl_3$) δ 7.47–7.44 (m, 2H), 7.41–7.28 (m, 3H), 6.96–6.66 (m, 2H), 6.64 (dd, *J* = 9.1, 4.3 Hz, 1H), 4.62 (s, 1H), 4.16 (brs, 1H), 1.43 (s, 3H), 0.75 (s, 3H); ^{13}C NMR (126 MHz, $CDCl_3$) δ 157.6 (d, *J* = 235.6 Hz), 140.1 (d, *J* = 7.0 Hz), 145.2, 139.7, 128.3, 127.8, 127.6, 113.5 (d, *J* = 23.2 Hz), 110.2 (d, *J* = 23.6 Hz), 75.3, 45.9, 26.5, 24.4; HPLC: the ee value was determined by HPLC analysis (Chiralpak IB, *i*-PrOH/Hexane = 10/90, 1.0 mL/min, 308 nm), retention time: t_{major} = 6.133 min, t_{minor} = 16.110 min, ee = 99%; $[\alpha]_D^{20}$ = – 160.3 (c = 0.10, THF). HRMS *m*/*z* $[M + H]^+$ calculated for $C_{16}H_{17}FN$: 242.1340, found 242.1345.

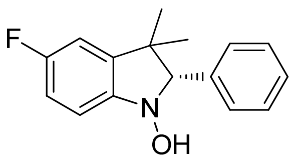

(*S*)-5-fluoro-3,3-dimethyl-2-phenylindolin-1-ol (5d)

^{1}H NMR (500 MHz, $CDCl_3$) δ 7.49 (d, *J* = 7.1 Hz, 2H), 7.42 (t, *J* = 7.3 Hz, 2H), 7.39–7.35 (m, 1H), 7.01 (dd, *J* = 8.5, 4.6 Hz, 1H), 6.94 (td, *J* = 8.9, 2.5 Hz, 1H), 6.83 (dd, *J* = 8.3, 2.5 Hz, 1H), 5.71–5.63 (m, 1H), 4.31 (s, 1H), 1.41 (s, 3H), 0.79 (s, 3H); ^{13}C NMR (126 MHz, $CDCl_3$) δ 159.9 (d, *J* = 240.0 Hz), 147.2, 139.3 (d, *J* = 7.4 Hz), 136.9, 128.5, 128.2, 128.1, 114.7 (d, *J* = 8.6 Hz), 114.3 (d, *J* = 23.7 Hz), 109.6 (d, *J* =

23.7 Hz), 85.8, 43.9, 25.8, 25.1; HPLC: the ee value was determined by HPLC analysis (Chiralpak IB, *i*-PrOH/Hexane = 10/90, 1.0 mL/min, 302 nm), retention time: t_{major} = 4.940 min, t_{minor} = 6.307 min, ee = 90%; $[\alpha]_D^{20}$ = + 92.2 (c = 0.19, THF). HRMS *m*/*z* $[M + H]^+$ calculated for $C_{16}H_{17}FNO$: 258.1289, found 258.1291.

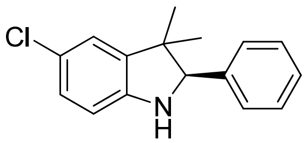

(*R*)-5-chloro-3,3-dimethyl-2-phenylindoline (4e)

It was prepared following the general procedure and purified by silica gel flash chromatography using ethyl acetate/ petroleum ether (15:85) as eluent to afford **4e** (11.1 mg, 43% yield) and **5e** (12.6 mg, 46% yield). ^{1}H NMR (500 MHz, $CDCl_3$) δ 7.45–7.41 (m, 2H), 7.39–7.31 (m, 3H), 7.04 (dd, *J* = 8.2, 2.2 Hz, 1H), 7.00 (d, *J* = 2.1 Hz, 1H), 6.64 (d, *J* = 8.2 Hz, 1H), 4.61 (s, 1H), 4.21 (brs, 1H), 1.42 (s, 3H), 0.74 (s, 3H); ^{13}C NMR (126 MHz, $CDCl_3$) δ 148.0, 140.3, 139.5, 128.4, 127.9, 127.6, 127.3, 123.7, 123.1, 110.2, 75.0, 45.9, 26.7, 24.6; HPLC: the ee value was determined by HPLC analysis (Chiralpak IB, *i*-PrOH/Hexane = 25/75, 1.0 mL/min, 253 nm), retention time: t_{major} = 5.440 min, t_{minor} = 13.793 min, ee = 99%; $[\alpha]_D^{25}$ = – 134.4 (c = 0.34, THF). HRMS *m*/*z* $[M + H]^+$ calculated for $C_{16}H_{17}ClN$: 258.1044, found 258.1049.

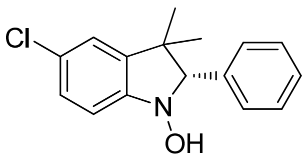

(*S*)-5-chloro-3,3-dimethyl-2-phenylindolin-1-ol (5e)

^{1}H NMR (500 MHz, $CDCl_3$) δ 7.51–7.46 (m, 2H), 7.43–7.39 (m, 2H), 7.39–7.35 (m, 1H), 7.25–7.17 (m, 1H), 7.09 (d, *J* = 2.0 Hz, 1H), 6.99 (d, *J* = 8.3 Hz, 1H), 5.74 (brs, 1H), 4.32 (s, 1H), 1.41 (s, 3H), 0.79 (s, 3H); ^{13}C NMR (126 MHz, $CDCl_3$) δ 149.9, 139.2, 136.7, 128.6, 128.2, 128.1, 127.8, 122.6, 114.8, 85.4, 44.0, 25.8, 25.1; HPLC: the ee value was determined by HPLC analysis (Chiralpak IB, *i*-PrOH/Hexane = 10/90, 1.0 mL/min, 252 nm), retention time: t_{major} = 5.093 min, t_{minor} = 6.563 min, ee = 92%; $[\alpha]_D^{25}$ = + 122.2 (c = 0.33, THF). HRMS *m*/*z* $[M + H]^+$ calculated for $C_{16}H_{17}ClNO$: 274.0993, found 274.0998.

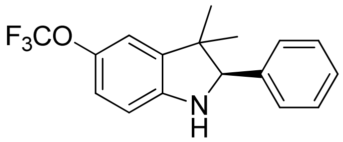

(*R*)-3,3-dimethyl-2-phenyl-5-(trifluoromethoxy)indoline (4f)

It was prepared following the general procedure and purified by silica gel flash chromatography using ethyl acetate/ petroleum ether (10:90) as eluent to afford **4f** (12.9 mg, 42% yield) and **5f** (14.9 mg, 46% yield). ^{1}H NMR (500 MHz, $CDCl_3$) δ

7.51–7.31 (m, 5H), 7.16–6.80 (m, 2H), 6.67 (d, J = 8.3 Hz, 1H), 4.67 (s, 1H), 4.12 (brs, 1H), 1.45 (s, 3H), 0.76 (s, 3H); ^{13}C NMR (126 MHz, $CDCl_3$) δ 148.0 (d, J = 26.6 Hz), 142.3, 139.8 (d, J = 5.8 Hz), 139.4 (d, J = 16.8 Hz), 128.4, 128.0, 127.6, 121.0 (q, J = 255.1 Hz), 120.6, 116.6, 109.3 (d, J = 15.5 Hz), 75.2 (d, J = 3.0 Hz), 45.8, 26.6, 24.6; HPLC: the ee value was determined by HPLC analysis (Chiralpak IB, *i*-PrOH/Hexane = 20/80, 1.0 mL/min, 305 nm), retention time: t_{major} = 5.630 min, t_{minor} = 14.627 min, ee = 97%; $[\alpha]_D^{20}$ = – 68.6 (c = 0.26, THF). HRMS *m*/*z* $[M + H]^+$ calculated for $C_{17}H_{17}F_3N$: 308.1257, found 308.1265.

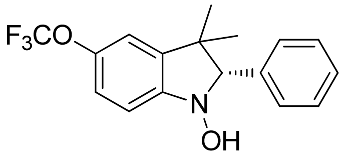

(*S*)-3,3-dimethyl-2-phenyl-5-(trifluoromethoxy)indolin-1-ol (5f)

^{1}H NMR (500 MHz, $CDCl_3$) δ 7.49 (d, J = 7.1 Hz, 2H), 7.47–7.30 (m, 3H), 7.17–7.08 (m, 1H), 7.04 (d, J = 8.5 Hz, 1H), 6.98 (s, 1H), 5.86 (s, 1H), 4.35 (s, 1H), 1.42 (s, 3H), 0.81 (s, 3H); ^{13}C NMR (126 MHz, $CDCl_3$) δ 149.9, 145.3, 139.0, 136.7, 128.6, 128.2, 128.2, 120.9, 120.83 (q, J = 256.0 Hz), 115.8, 114.3, 85.6, 43.9, 25.8, 25.1; HPLC: the ee value was determined by HPLC analysis (Chiralpak IB, *i*-PrOH/Hexane = 10/90, 1.0 mL/min, 252 nm), retention time: t_{major} = 4.67 min, t_{minor} = 6.193 min, ee = 89%; $[\alpha]_D^{20}$ = + 168.5 (c = 0.50, THF). HRMS *m*/*z* $[M + H]^+$ calculated for $C_{17}H_{17}F_3NO_2$: 324.1206, found 324.1211.

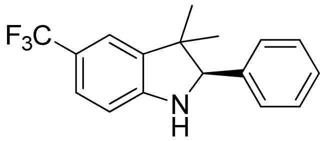

(*R*)-3,3-dimethyl-2-phenyl-5-(trifluoromethyl)indoline (4g)

It was prepared following the general procedure and purified by silica gel flash chromatography using ethyl acetate/ petroleum ether (20:80) as eluent to afford **4g** (14.3 mg, 49% yield) and **5g** (12.3 mg, 40% yield). ^{1}H NMR (500 MHz, $CDCl_3$) δ 7.44–7.40 (m, 2H), 7.40–7.31 (m, 4H), 7.25 (s, 1H), 6.73 (d, J = 8.1 Hz, 1H), 4.68 (s, 1H), 1.46 (s, 3H), 0.77 (s, 3H); ^{13}C NMR (126 MHz, $CDCl_3$) δ 152.3, 139.4, 138.4, 128.5, 128.0, 127.4, 125.59 (q, J = 4.0 Hz), 125.32 (q, J = 270.5 Hz), 120.90 (q, J = 32.1 Hz), 119.88 (q, J = 3.7 Hz), 108.3, 74.8, 45.5, 27.0, 24.7; HPLC: the ee value was determined by HPLC analysis (Chiralpak IB, *i*-PrOH/Hexane = 35/65, 1.0 mL/min, 253 nm), retention time: t_{major} = 5.283 min, t_{minor} = 12.807 min, ee = 88%; $[\alpha]_D^{20}$ = – 152.5 (c = 0.25, THF). HRMS *m*/*z* $[M + H]^+$ calculated for $C_{17}H_{17}F_3N$: 292.1308, found 292.1316.

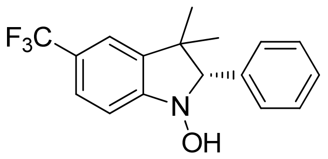

(*S*)-3,3-dimethyl-2-phenyl-5-(trifluoromethyl)indolin-1-ol (5g)
^{1}H NMR (500 MHz, $CDCl_3$) δ 7.52 (dd, *J* = 8.2, 0.8 Hz, 1H), 7.50–7.46 (m, 2H), 7.46–7.41 (m, 2H), 7.41–7.37 (m, 1H), 7.36 (s, 1H), 7.12 (d, *J* = 8.2 Hz, 1H), 5.65 (brs, 1H), 4.40 (s, 1H), 1.45 (s, 3H), 0.82 (s, 3H); ^{13}C NMR (126 MHz, $CDCl_3$) δ 154.1, 137.7, 136.4, 128.7, 128.3, 128.1, 125.5 (q, *J* = 3.9 Hz), 125.2 (q, *J* = 32.0 Hz), 124.9 (q, *J* = 271.4 Hz), 119.5 (q, *J* = 3.7 Hz), 113.1, 85.2, 43.9, 25.9, 25.1; HPLC: the ee value was determined by HPLC analysis (Chiralpak IB, *i*-PrOH/Hexane = 10/90, 1.0 mL/min, 256 nm), retention time: t_{major} = 5.033 min, t_{minor} = 6.337 min, ee = 96%; $[\alpha]_D^{20}$ = + 132.4 (c = 0.30, THF). HRMS *m*/*z* $[M + H]^+$ calculated for $C_{17}H_{17}F_3NO$: 308.1257, found 308.1263.

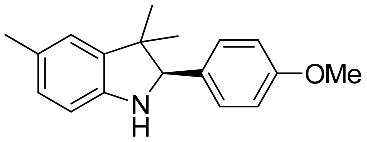

(*R*)-2-(4-methoxyphenyl)-3,3,5-trimethylindoline (4h)
It was prepared following the general procedure and purified by silica gel flash chromatography using ethyl acetate/ petroleum ether (10:90) as eluent to afford **4h** (10.9 mg, 41% yield) and **5h** (12.2 mg, 43% yield). ^{1}H NMR (500 MHz, $CDCl_3$) δ 7.41 (d, *J* = 8.3 Hz, 2H), 6.93 (d, *J* = 9.8 Hz, 4H), 6.67 (d, *J* = 7.6 Hz, 1H), 4.55 (s, 1H), 3.85 (s, 3H), 2.34 (s, 3H), 1.43 (s, 3H), 0.78 (s, 3H); ^{13}C NMR (126 MHz, $CDCl_3$) δ 159.2, 147.0, 138.6, 132.1, 128.7, 128.4, 127.8, 123.4, 113.6, 109.3, 74.5, 55.4, 45.4, 26.5, 24.6, 21.1; HPLC: the ee value was determined by HPLC analysis (Chiralpak IB, *i*-PrOH/Hexane = 10/90, 1.0 mL/min, 248 nm), retention time: t_{major} = 5.020 min, t_{minor} = 7.603 min, ee = 94%; $[\alpha]_D^{20}$ = – 96.4 (c = 0.20, THF). HRMS *m*/*z* $[M + H]^+$ calculated for $C_{18}H_{22}NO$: 268.1696, found 268.1613.

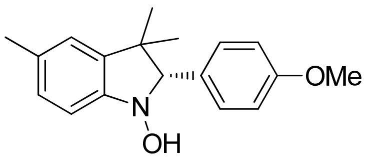

(*S*)-2-(4-methoxyphenyl)-3,3,5-trimethylindolin-1-ol (5h)
^{1}H NMR (500 MHz, CD_3CN) δ 7.45–7.36 (m, 2H), 7.10–6.98 (m, 2H), 6.99–6.90 (m, 3H), 6.85 (d, *J* = 7.9 Hz, 1H), 4.11 (s, 1H), 3.81 (s, 3H), 2.30 (s, 3H), 1.34 (s, 3H), 0.69 (s, 3H); ^{13}C NMR (126 MHz, CD_3CN) δ 160.1, 150.7, 138.3, 132.8, 130.5, 130.2, 128.8, 123.6, 114.3, 113.8, 85.4, 55.8, 44.0, 26.1, 25.3, 21.1; HPLC: the ee value was determined by HPLC analysis (Chiralpak AS-H, *i*-PrOH/Hexane = 10/90, 1.0 mL/min, 251 nm), retention time: t_{major} = 7.850 min, t_{minor} = 10.213 min, ee = 87%; $[\alpha]_D^{20}$ = + 72.2 (c = 0.19, THF). HRMS *m*/*z* $[M + H]^+$ calculated for $C_{18}H_{22}NO_2$: 284.1645, found 284.1642.

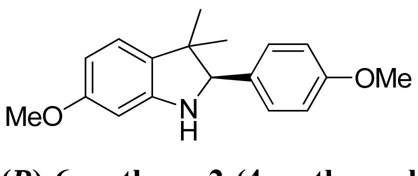

(*R*)-6-methoxy-2-(4-methoxyphenyl)-3,3-dimethylindoline (4i)

It was prepared following the general procedure and purified by silica gel flash chromatography using ethyl acetate/ petroleum ether (10:90) as eluent to afford **4i** (12.5 mg, 44% yield) and **5i** (12.0 mg, 40% yield). ^{1}H NMR (500 MHz, $CDCl_3$) δ 7.37 (d, *J* = 8.6 Hz, 2H), 6.99–6.87 (m, 3H), 6.35 (d, *J* = 7.8 Hz, 2H), 4.56 (s, 1H), 3.83 (s, 3H), 3.79 (s, 3H), 1.39 (s, 3H), 0.73 (s, 3H); ^{13}C NMR (126 MHz, $CDCl_3$) δ 160.1, 159.3, 150.5, 132.0, 131.0, 128.6, 123.0, 113.7, 104.0, 96.5, 74.7, 55.6, 55.5, 44.8, 27.0, 24.8; HPLC: the ee value was determined by HPLC analysis (Chiralpak IB, *i*-PrOH/Hexane = 10/90, 1.0 mL/min, 234 nm), retention time: t_{major} = 7.370 min, t_{minor} = 8.770 min, ee = 93%; $[\alpha]_D^{20}$ = – 135.7 (c = 0.20, THF). HRMS *m*/*z* $[M + H]^+$ calculated for $C_{18}H_{22}NO_2$: 284.1645, found 284.1651.

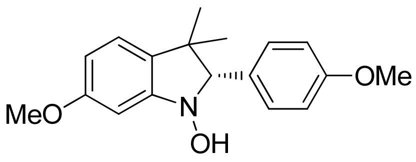

(*S*)-6-methoxy-2-(4-methoxyphenyl)-3,3-dimethylindolin-1-ol (5i)

^{1}H NMR (500 MHz, $CDCl_3$) δ 7.41 (d, *J* = 8.6 Hz, 2H), 7.01 (d, *J* = 8.2 Hz, 1H), 6.95 (d, *J* = 8.7 Hz, 2H), 6.68 (d, *J* = 2.3 Hz, 1H), 6.57 (dd, *J* = 8.2, 2.4 Hz, 1H), 5.57 (s, 1H), 4.25 (s, 1H), 3.84 (d, *J* = 8.1 Hz, 6H), 1.37 (s, 3H), 0.78 (s, 3H); ^{13}C NMR (126 MHz, $CDCl_3$) δ 160.1, 159.4, 152.5, 129.6, 129.3, 122.8, 113.9, 109.0, 99.6, 85.3, 55.7, 55.5, 43.2, 26.2, 25.3; HPLC: the ee value was determined by HPLC analysis (Chiralpak IB, *i*-PrOH/Hexane = 20/80, 1.0 mL/min, 251 nm), retention time: t_{major} = 6.213 min, t_{minor} = 7.373 min, ee = 88%; $[\alpha]_D^{20}$ = + 97.8 (c = 0.80, THF). HRMS *m*/*z* $[M + H]^+$ calculated for $C_{18}H_{22}NO_3$: 300.1594, found 300.1597.

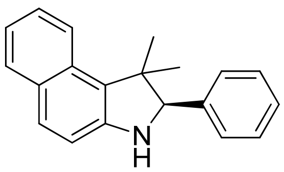

(*R*)-1,1-dimethyl-2-phenyl-2,3-dihydro-1*H*-benzo[*e*]indole (4j)

It was prepared following the general procedure and purified by silica gel flash chromatography using ethyl acetate/ petroleum ether (10:90) as eluent to afford **4j** (11.7 mg, 43% yield) and **5j** (11.8 mg, 41% yield). ^{1}H NMR (500 MHz, $CDCl_3$) δ 7.97 (d, *J* = 8.3 Hz, 1H), 7.79 (d, *J* = 8.2 Hz, 1H), 7.65 (d, *J* = 8.5 Hz, 1H), 7.60–7.51 (m, 2H), 7.50–7.28 (m, 4H), 7.26–7.19 (m, 1H), 7.09 (d, *J* = 8.5 Hz, 1H), 4.73 (s, 1H), 1.77 (s, 3H), 1.02 (s, 3H); ^{13}C NMR (126 MHz, $CDCl_3$) δ 147.1, 139.7, 131.2, 129.9, 129.7, 129.1, 128.3, 128.3, 127.9, 126.9, 126.4, 121.8, 113.1, 75.4, 47.4, 27.6, 23.2; HPLC: the ee value was determined by HPLC analysis (Chiralpak IB, *i*-PrOH/Hexane

= 20/80, 1.0 mL/min, 301 nm), retention time: t_{major} = 6.027 min, t_{minor} = 10.643 min, ee = 93%; $[\alpha]_D^{20}$ = – 143.3 (c = 0.42, THF). HRMS *m*/*z* $[M + H]^+$ calculated for $C_{20}H_{20}N$: 274.1590, found 274.1594.

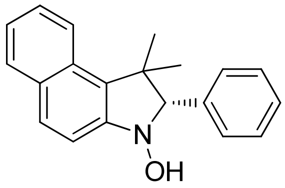

(*S*)-1,1-dimethyl-2-phenyl-1*H*-benzo[e]indol-3(2*H*)-ol (5j)

^{1}H NMR (500 MHz, $CDCl_3$) δ 8.05 (d, *J* = 8.5 Hz, 1H), 7.87 (d, *J* = 8.2 Hz, 1H), 7.80 (d, *J* = 8.6 Hz, 1H), 7.58 (d, *J* = 7.1 Hz, 2H), 7.53–7.32 (m, 6H), 4.43 (s, 1H), 1.78 (s, 3H), 1.06 (s, 3H); ^{13}C NMR (126 MHz, $CDCl_3$) δ 149.1, 137.1, 131.9, 130.3, 129.7, 129.2, 128.9, 128.5, 128.1, 127.9, 126.4, 123.5, 122.9, 115.3, 85.7, 45.2, 27.4, 23.7; HPLC: the ee value was determined by HPLC analysis (Chiralpak IB, *i*-PrOH/Hexane = 5/95, 1.0 mL/min, 249 nm), retention time: t_{major} = 7.933 min, t_{minor} = 10.843 min, ee = 91%; $[\alpha]_D^{20}$ = + 102.1 (c = 0.25, THF). HRMS *m*/*z* $[M + H]^+$ calculated for $C_{20}H_{20}NO$: 290.1539, found 290.1533.

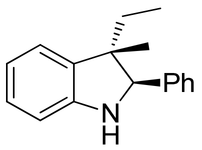

(2*R*,3*S*)-3-Ethyl-3-methyl-2-phenylindoline (6a)

It was prepared following the general procedure and purified by silica gel flash chromatography using ethyl acetate/ petroleum ether (10:90) as eluent to afford **6a** (10.9 mg, 46% yield) and **7a** (11.4 mg, 45% yield). ^{1}H NMR (500 MHz, $CDCl_3$) δ 7.42–7.28 (m, 5H), 7.12 (td, *J* = 7.6, 1.2 Hz, 1H), 7.07–6.96 (m, 1H), 6.81 (td, *J* = 7.4, 0.7 Hz, 1H), 6.74 (d, *J* = 7.7 Hz, 1H), 4.75 (s, 1H), 4.10 (brs, 1H), 1.94–1.70 (m, 2H), 1.03 (t, *J* = 7.5 Hz, 3H), 0.80 (s, 3H); ^{13}C NMR (126 MHz, $CDCl_3$) δ 150.1, 141.3, 135.9, 128.3, 127.6, 127.5, 123.5, 118.9, 109.0, 70.3, 49.7, 32.4, 23.7, 9.6; HPLC: the ee value was determined by HPLC analysis (Chiralpak IB, *i*-PrOH/Hexane = 10/90, 1.0 mL/min, 246 nm), retention time: t_{major} = 5.837 min, t_{minor} = 11.297 min, ee = 88%; $[\alpha]_D^{20}$ = – 119.6 (c = 0.16, THF). HRMS *m*/*z* $[M + H]^+$ calculated for $C_{17}H_{20}N$: 238.1590, found 238.1593.

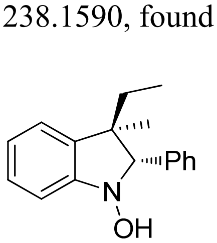

(2*S*,3*R*)-3-Ethyl-3-methyl-2-phenylindolin-1-ol (7a)

^{1}H NMR (500 MHz, $CDCl_3$) δ 7.49 (d, *J* = 7.2 Hz, 2H), 7.42 (t, *J* = 7.4 Hz, 2H), 7.39–7.31 (m, 1H), 7.29–7.24 (m, 1H), 7.20–6.99 (m, 3H), 5.69 (s, 1H), 4.53 (s, 1H), 1.92–1.81 (m, 1H), 1.71–1.65 (m, 1H), 1.02 (t, *J* = 7.5 Hz, 3H), 0.81 (s, 3H); ^{13}C NMR

(126 MHz, $CDCl_3$) δ 151.8, 137.7, 135.0, 128.5, 128.3, 127.8, 127.7, 123.2, 122.7, 113.8, 80.0, 47.8, 29.7, 25.5, 9.7; HPLC: the ee value was determined by HPLC analysis (Chiralpak IB, *i*-PrOH/Hexane = 5/95, 1.0 mL/min, 247 nm), retention time: t_{major} = 6.233 min, t_{minor} = 8.083 min, ee = 93%; $[\alpha]_D^{20}$ = + 183.0 (c = 0.24, THF). HRMS *m*/*z* $[M + H]^+$ calculated for $C_{17}H_{20}NO$: 254.1359, found 254.1355.

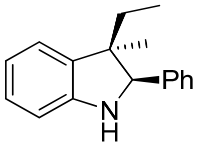

(2*R*,3*R*)-3-Ethyl-3-methyl-2-phenylindoline (6b)

It was prepared following the general procedure and purified by silica gel flash chromatography using ethyl acetate/ petroleum ether (10:90) as eluent to afford **6b** (10.4 mg, 44% yield) and **7b** (11.6 mg, 46% yield). ^1H NMR (500 MHz, C_6D_6) δ 7.40–7.31 (m, 2H), 7.22–7.17 (m, 2H), 7.15–7.13 (m, 1H), 7.10 (td, *J* = 7.6, 1.3 Hz, 1H), 6.97 (dd, *J* = 7.3, 0.8 Hz, 1H), 6.82 (td, *J* = 7.4, 0.9 Hz, 1H), 6.50 (d, *J* = 7.7 Hz, 1H), 4.30 (s, 1H), 3.16 (s, 1H), 1.50–1.41 (m, 1H), 1.24 (s, 3H), 0.81–0.72 (m, 1H), 0.60 (t, *J* = 7.5 Hz, 3H); ^{13}C NMR (126 MHz, C_6D_6) δ 150.4, 139.9, 136.1, 128.4, 127.6, 127.6, 124.3, 118.8, 109.8, 76.3, 48.5, 28.3, 22.5, 8.5; HPLC: the ee value was determined by HPLC analysis (Chiralpak IB, *i*-PrOH/Hexane = 10/90, 1.0 mL/min, 304 nm), retention time: t_{major} = 5.730 min, t_{minor} = 12.590 min, ee = 91%; $[\alpha]_D^{20}$ = –76.1 (c = 0.28, THF). HRMS *m*/*z* $[M + H]^+$ calculated for $C_{17}H_{20}N$: 238.1590, found 238.1586.

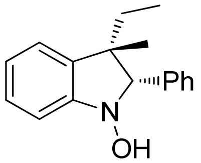

(2*S*,3*S*)-3-Ethyl-3-methyl-2-phenylindolin-1-ol (7b)

^1H NMR (500 MHz, $CDCl_3$) δ 7.53 (d, *J* = 7.3 Hz, 2H), 7.43 (t, *J* = 7.4 Hz, 2H), 7.37 (t, *J* = 7.3 Hz, 1H), 7.30–7.27 (m, 1H), 7.14 (dd, *J* = 13.8, 7.5 Hz, 2H), 7.05 (t, *J* = 7.4 Hz, 1H), 5.64 (s, 1H), 4.41 (s, 1H), 1.43 (s, 3H), 1.40–1.34 (m, 1H), 0.94–0.89 (m, 1H), 0.69 (t, *J* = 7.5 Hz, 3H); ^{13}C NMR (126 MHz, $CDCl_3$) δ 151.9, 137.1, 135.3, 128.5, 128.3, 127.8, 127.8, 124.0, 122.6, 113.7, 86.9, 47.4, 29.1, 22.6, 8.7; HPLC: the ee value was determined by HPLC analysis (Chiralpak IB, *i*-PrOH/Hexane = 10/90, 1.0 mL/min, 247 nm), retention time: t_{major} = 5.067 min, t_{minor} = 6.693 min, ee = 87%; $[\alpha]_D^{20}$ = + 148.9 (c = 0.26, THF). HRMS *m*/*z* $[M + H]^+$ calculated for $C_{17}H_{20}NO$: 254.1359, found 254.1366.

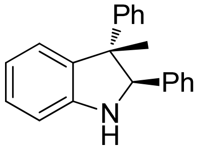

(2*R*,3*S*)-3-Methyl-2,3-diphenylindoline (6c)

It was prepared following the general procedure and purified by silica gel flash

chromatography using ethyl acetate/ petroleum ether (10:90) as eluent to afford **6c** (12.3 mg, 43% yield) and **7c** (13.5 mg, 45% yield). ^{1}H NMR (500 MHz, $CDCl_3$) δ 7.41–7.26 (m, 8H), 7.25–7.13 (m, 3H), 6.99–6.69 (m, 3H), 5.06 (s, 1H), 4.24 (brs, 1H), 1.18 (s, 3H); ^{13}C NMR (126 MHz, $CDCl_3$) δ 149.9, 147.0, 139.1, 137.8, 128.3, 128.2, 128.1, 127.9, 127.7, 127.6, 126.6, 124.8, 119.6, 109.6, 77.0, 54.1, 21.7; HPLC: the ee value was determined by HPLC analysis (Chiralpak IB, *i*-PrOH/Hexane = 10/90, 1.0 mL/min, 208 nm), retention time: t_{major} = 5.103 min, t_{minor} = 6.490 min, ee = 94%; $[\alpha]_D^{20}$ = – 101.2 (c = 0.30, THF). HRMS *m*/*z* $[M + H]^+$ calculated for $C_{21}H_{20}N$: 286.1590, found 286.1597.

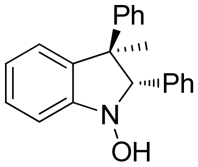

(2*S*,3*R*)-3-Methyl-2,3-diphenylindolin-1-ol (7c)

^{1}H NMR (500 MHz, $CDCl_3$) δ 7.38–7.25 (m, 9H), 7.19 (d, *J* = 7.8 Hz, 1H), 7.17–7.08 (m, 2H), 7.03 (t, *J* = 7.4 Hz, 1H), 6.87 (d, *J* = 7.4 Hz, 1H), 4.73 (s, 1H), 2.69 (s, 1H), 1.21 (s, 3H); ^{13}C NMR (126 MHz, $CDCl_3$) δ 151.5, 145.3, 137.1, 136.3, 128.5, 128.3, 128.1, 128.0, 127.9, 126.8, 124.3, 123.6, 114.0, 87.5, 52.0, 21.9; HPLC: the ee value was determined by HPLC analysis (Chiralpak IB, *i*-PrOH/Hexane = 5/95, 1.0 mL/min, 250 nm), retention time: t_{major} = 5.903 min, t_{minor} = 7.263 min, ee = 87%; $[\alpha]_D^{20}$ = + 120.5 (c = 0.25, THF). HRMS *m*/*z* $[M + H]^+$ calculated for $C_{21}H_{20}NO$: 302.1539, found 302.1542.

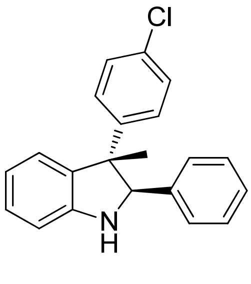

(2*R*,3*S*)-3-(4-Chlorophenyl)-3-methyl-2-phenylindoline (6d)

It was prepared following the general procedure and purified by silica gel flash chromatography using ethyl acetate/ petroleum ether (10:90) as eluent to afford **6d** (13.4 mg, 42% yield) and **7d** (14.7 mg, 44% yield). ^{1}H NMR (500 MHz, $CDCl_3$) δ 7.40–7.23 (m, 7H), 7.21–7.12 (m, 3H), 6.92–6.73 (m, 3H), 4.98 (s, 1H), 4.25 (brs, 1H), 1.14 (s, 3H); ^{13}C NMR (126 MHz, $CDCl_3$) δ 149.81, 145.6, 138.6, 137.3, 132.5, 129.7, 128.4, 128.2, 128.1, 127.9, 127.5, 124.6, 119.8, 109.8, 77.0, 53.8, 21.7; HPLC: the ee value was determined by HPLC analysis (Chiralpak IB, *i*-PrOH/Hexane = 10/90, 1.0 mL/min, 298 nm), retention time: t_{major} = 4.587 min, t_{minor} = 6.707 min, ee = 91%; $[\alpha]_D^{20}$ = – 60.1 (c = 0.36, THF). HRMS *m*/*z* $[M + H]^+$ calculated for $C_{21}H_{19}ClN$: 320.1201, found 320.1205.

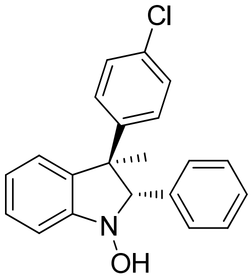

(2*S*,3*R*)-3-(4-Chlorophenyl)-3-methyl-2-phenylindolin-1-ol (7d)

^{1}H NMR (500 MHz, $CDCl_3$) δ 7.43–7.28 (m, 6H), 7.22–7.16 (m, 3H), 7.15–7.09 (m, 2H), 7.04 (t, *J* = 7.4 Hz, 1H), 6.84 (d, *J* = 7.4 Hz, 1H), 5.72 (d, *J* = 3.0 Hz, 1H), 4.67 (s, 1H), 1.18 (s, 3H); ^{13}C NMR (126 MHz, $CDCl_3$) δ 151.5, 143.9, 136.5, 136.0, 132.8, 129.9, 128.4, 128.4, 128.3, 128.1, 128.0, 124.1, 123.7, 114.2, 87.4, 51.7, 21.9; HPLC: the ee value was determined by HPLC analysis (Chiralcel AD-H, *i*-PrOH/Hexane = 20/80, 1.0 mL/min, 211 nm), retention time: t_{major} = 7.720 min, t_{minor} = 13.773 min, ee = 88%; $[\alpha]_D^{20}$ = + 67.5 (c = 0.33, THF). HRMS *m*/*z* $[M + H]^+$ calculated for $C_{21}H_{19}ClNO$: 336.1150, found 336.1153.

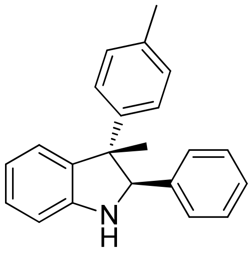

(2*R*,3*S*)-3-Methyl-2-phenyl-3-(p-tolyl)indoline (6e)

It was prepared following the general procedure and purified by silica gel flash chromatography using ethyl acetate/ petroleum ether (10:90) as eluent to afford **6e** (13.2 mg, 44% yield) and **7e** (14.2 mg, 45% yield). ^{1}H NMR (500 MHz, $CDCl_3$) δ 7.37–7.27 (m, 3H), 7.26–7.08 (m, 7H), 6.94–6.74 (m, 3H), 5.03 (s, 1H), 4.22 (brs, 1H), 2.41 (s, 3H), 1.15 (s, 3H); ^{13}C NMR (126 MHz, $CDCl_3$) δ 149.9, 144.0, 139.2, 138.0, 136.1, 128.9, 128.1, 128.1, 127.8, 127.6, 127.6, 124.8, 119.6, 109.6, 76.9, 53.8, 21.8, 21.2; HPLC: the ee value was determined by HPLC analysis (Chiralpak IB, *i*-PrOH/Hexane = 5/95, 1.0 mL/min, 248 nm), retention time: t_{major} = 5.543 min, t_{minor} = 8.353 min, ee = 87%; $[\alpha]_D^{20}$ = – 84.6 (c = 0.36, THF). HRMS *m*/*z* $[M + H]^+$ calculated for $C_{22}H_{22}N$: 300.1747, found 300.1744.

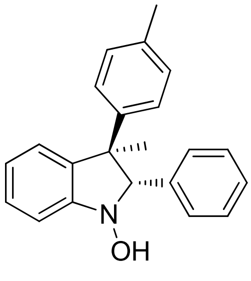

(2*S*,3*R*)-3-Methyl-2-phenyl-3-(p-tolyl)indolin-1-ol (7e)

^{1}H NMR (500 MHz, $CDCl_3$) δ 7.41–7.28 (m, 4H), 7.20 (d, *J* = 7.8 Hz, 1H), 7.19–7.06

(m, 6H), 7.03 (t, *J* = 7.4 Hz, 1H), 6.87 (d, *J* = 7.5 Hz, 1H), 5.69 (s, 1H), 4.72 (s, 1H), 2.39 (s, 3H), 1.18 (s, 3H); ^{13}C NMR (126 MHz, $CDCl_3$) δ 151.5, 142.3, 137.3, 136.4, 136.4, 129.0, 128.4, 128.2, 128.0, 128.0, 127.8, 124.3, 123.6, 114.0, 87.5, 51.7, 22.0, 21.2; HPLC: the ee value was determined by HPLC analysis (Chiralpak IB, *i*-PrOH/Hexane = 5/95, 1.0 mL/min, 248 nm), retention time: t_{major} = 5.983 min, t_{minor} = 7.183 min, ee = 89%; $[\alpha]_D^{20}$ = + 90.0 (c = 0.26, THF). HRMS *m*/*z* $[M + H]^+$ calculated for $C_{22}H_{22}NO$: 316.1696, found 316.1691.

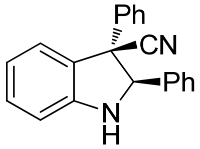

(2*R*,3*R*)-2,3-Diphenylindoline-3-carbonitrile (6f)

It was prepared following the general procedure and purified by silica gel flash chromatography using ethyl acetate/ petroleum ether (20:80) as eluent to afford **6f** (13.6 mg, 46% yield) and **7f** (14.0 mg, 45% yield). ^{1}H NMR (500 MHz, $CDCl_3$) δ 7.44–7.32 (m, 8H), 7.29–7.23 (m, 3H), 7.04 (d, *J* = 7.1 Hz, 1H), 6.88 (t, *J* = 7.4 Hz, 2H), 5.03 (s, 1H), 4.24 (brs, 1H); ^{13}C NMR (126 MHz, $CDCl_3$) δ 150.4, 137.8, 136.5, 130.5, 129.3, 129.0, 128.7, 128.6, 127.8, 127.4, 125.9, 120.8, 119.1, 110.8, 77.9, 58.6; HPLC: the ee value was determined by HPLC analysis (Chiralpak IB, *i*-PrOH/Hexane = 20/80, 1.0 mL/min, 308 nm), retention time: t_{major} = 6.923 min, t_{minor} = 7.707 min, ee = 94%; $[\alpha]_D^{20}$ = – 15.2 (c = 0.27, THF). HRMS *m*/*z* $[M + H]^+$ calculated for $C_{21}H_{17}N_2$: 297.1386, found 297.1394. These NMR data are consistent with reported literature values.[8]

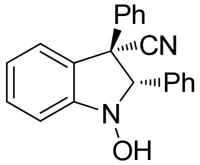

(2*S*,3*S*)-1-Hydroxy-2,3-diphenylindoline-3-carbonitrile (7f)

^{1}H NMR (500 MHz, $CDCl_3$) δ 7.70–7.30 (m, 9H), 7.29–7.25 (m, 3H), 7.15 (td, *J* = 7.6, 0.9 Hz, 1H), 7.08 (d, *J* = 7.2 Hz, 1H), 6.01 (s, 1H), 4.72 (s, 1H); ^{13}C NMR (126 MHz, $CDCl_3$) δ 152.6, 136.1, 133.5, 130.7, 129.4, 129.1, 128.9, 128.7, 128.5, 128.1, 126.2, 125.3, 124.6, 118.6, 114.8, 87.5, 55.9; HPLC: the ee value was determined by HPLC analysis (Chiralcel OD-H, *i*-PrOH/Hexane = 20/80, 1.0 mL/min, 248 nm), retention time: t_{major} = 4.360 min, t_{minor} = 6.133 min, ee = 92%; $[\alpha]_D^{20}$ = + 67.1 (c = 0.52, THF). HRMS *m*/*z* $[M + H]^+$ calculated for $C_{21}H_{17}N_2O$: 213.1335, found 313.1338.

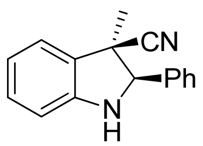

(2*R*,3*R*)-3-Methyl-2-phenylindoline-3-carbonitrile (6g)

It was prepared following the general procedure and purified by silica gel flash chromatography using ethyl acetate/ petroleum ether (15:85) as eluent to afford **6g** (9.8 mg, 42% yield) and **7g** (11.5 mg, 46% yield). ^{1}H NMR (500 MHz, $CDCl_3$) δ 7.59 (d, *J* = 7.0 Hz, 2H), 7.48–7.37 (m, 3H), 7.33 (d, *J* = 7.5 Hz, 1H), 7.21 (td, *J* = 7.7, 1.0 Hz, 1H), 6.90 (t, *J* = 7.5 Hz, 1H), 6.80 (d, *J* = 7.8 Hz, 1H), 5.30 (s, 1H), 4.18 (brs, 1H), 1.09 (s, 3H); ^{13}C NMR (126 MHz, $CDCl_3$) δ 148.6, 136.3, 129.9, 129.8, 128.9, 127.5, 124.0, 123.0, 120.4, 110.5, 71.5, 44.9, 21.7; HPLC: the ee value was determined by HPLC analysis (Chiralpak IB, *i*-PrOH/Hexane = 25/75, 1.0 mL/min, 306 nm), retention time: t_{major} = 5.993 min, t_{minor} = 12.287 min, ee = 95%; $[\alpha]_D^{20}$ = – 76.9 (c = 0.30, THF). HRMS *m*/*z* $[M + H]^+$ calculated for $C_{16}H_{15}N_2$: 235.1230, found 235.1226.

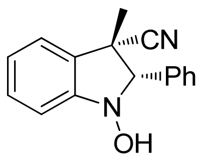

(2*S*,3*S*)-1-Hydroxy-3-methyl-2-phenylindoline-3-carbonitrile (7g)

^{1}H NMR (500 MHz, $CDCl_3$) δ 7.65–7.57 (m, 2H), 7.56–7.28 (m, 5H), 7.22–7.07 (m, 2H), 5.91 (s, 1H), 4.95 (s, 1H), 1.16 (s, 3H); ^{13}C NMR (126 MHz, $CDCl_3$) δ 150.6, 134.2, 130.0, 129.1, 129.1, 128.8, 127.9, 124.2, 123.4, 121.6, 114.6, 81.0, 43.0, 22.5; HPLC: the ee value was determined by HPLC analysis (Chiralpak IB, *i*-PrOH/Hexane = 15/85, 1.0 mL/min, 245 nm), retention time: t_{major} = 11.363 min, t_{minor} = 12.457 min, ee = 91%; $[\alpha]_D^{20}$ = + 90.0 (c = 1.0, THF). HRMS *m*/*z* $[M + H]^+$ calculated for $C_{16}H_{15}N_2O$: 251.1179, found 251.1180.

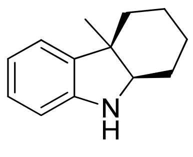

(4a*R*,9a*R*)-4a-Methyl-2,3,4,4a,9,9a-hexahydro-1*H*-carbazole (6h)

It was prepared following the general procedure and purified by silica gel flash chromatography using ethyl acetate/ petroleum ether (5:95) as eluent to afford **6h** (8.4 mg, 45% yield) and **7h** (8.7 mg, 43% yield). ^{1}H NMR (500 MHz, $CDCl_3$) δ 7.11–7.00 (m, 2H), 6.78 (td, *J* = 7.4, 0.8 Hz, 1H), 6.72 (d, *J* = 7.6 Hz, 1H), 3.59 (brs, 1H), 3.44 (t, *J* = 4.4 Hz, 1H), 1.74–1.59 (m, 4H), 1.53–1.39 (m, 4H), 1.33 (s, 3H); ^{13}C NMR (126 MHz, $CDCl_3$) δ 149.5, 139.6, 127.2, 121.7, 119.1, 110.4, 66.1, 42.9, 35.2, 27.7, 23.8, 21.68, 21.3; HPLC: the ee value was determined by HPLC analysis (Chiralpak IB, *i*-PrOH/Hexane = 3/97, 1.0 mL/min, 298 nm), retention time: t_{major} = 4.770 min, t_{minor} = 5.143 min, ee = 86%; $[\alpha]_D^{25}$ = + 7.4 (c = 1.0, THF). HRMS *m*/*z* $[M + H]^+$ calculated for $C_{13}H_{18}N$: 188.1434, found 188.1438.

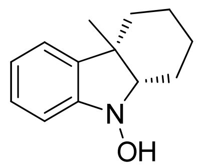

(4a*S*,9a*S*)-4a-Methyl-2,3,4,4a-tetrahydro-1*H*-carbazol-9(9aH)-ol (7h)
^{1}H NMR (500 MHz, CD_3CN) δ 7.13 (td, *J* = 7.6, 1.2 Hz, 1H), 7.09 (d, *J* = 7.4 Hz, 1H), 7.04 (s, 1H), 6.91 (td, *J* = 7.4, 1.0 Hz, 1H), 6.85 (d, *J* = 7.8 Hz, 1H), 2.91 (t, *J* = 6.6 Hz, 1H), 1.92–1.82 (m, 1H), 1.77–1.68 (m, 1H), 1.36 (s, 3H), 1.12 (t, *J* = 7.6 Hz, 3H), 1.03 (s, 3H); ^{13}C NMR (126 MHz, CD_3CN) δ 153.4, 139.1, 128.1, 123.0, 122.5, 113.7, 82.6, 42.8, 27.2, 23.7, 23.1, 22.5, 12.4; HPLC: the ee value was determined by HPLC analysis (Chiralcel AD-H, *i*-PrOH/Hexane = 10/90, 1.0 mL/min, 255 nm), retention time: t_{minor} = 6.287 min, t_{major} = 6.650 min, ee = 84%; $[\alpha]_D^{25}$ = + 2.1 (c = 0.35, THF). HRMS *m*/*z* $[M + H]^+$ calculated for $C_{13}H_{18}NO$: 204.1383, found 204.1389.

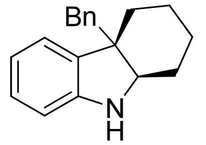

(4a*S*,9a*R*)-4a-Benzyl-2,3,4,4a,9,9a-hexahydro-1*H*-carbazole (6i)
It was prepared following the general procedure and purified by silica gel flash chromatography using ethyl acetate/ petroleum ether (5:95) as eluent to afford **6i** (12.4 mg, 47% yield) and **7i** (12.3 mg, 44% yield). ^{1}H NMR (500 MHz, $CDCl_3$) δ 7.26–7.15 (m, 3H), 7.08 (td, *J* = 7.6, 1.1 Hz, 1H), 7.02–6.92 (m, 2H), 6.80 (d, *J* = 6.8 Hz, 1H), 6.77–6.64 (m, 2H), 3.47 (dd, *J* = 7.7, 5.5 Hz, 1H), 2.95 (d, *J* = 13.2 Hz, 1H), 2.85 (d, *J* = 13.2 Hz, 1H), 1.93–1.85 (m, 1H), 1.80–1.72 (m, 1H), 1.64 (ddd, *J* = 13.7, 10.2, 3.5 Hz, 1H), 1.61–1.52 (m, 2H), 1.41–1.32 (m, 1H), 1.30–1.20 (m, 2H); ^{13}C NMR (126 MHz, $CDCl_3$) δ 149.8, 138.5, 135.0, 131.1, 127.7, 127.5, 126.2, 123.7, 118.7, 110.9, 63.4, 48.6, 45.0, 32.0, 29.8, 22.0, 21.9; HPLC: the ee value was determined by HPLC analysis (Chiralcel OD-H, *i*-PrOH/Hexane = 3/97, 1.0 mL/min, 296 nm), retention time: t_{minor} = 6.677 min, t_{major} = 7.100 min, ee = 86%; $[\alpha]_D^{25}$ = + 78.8 (c = 1.0, THF). HRMS *m*/*z* $[M + H]^+$ calculated for $C_{19}H_{22}N$: 264.1747, found 264.1745. The absolute configuration of recovered **6i** was assigned to be *R* by comparing the HPLC analysis and optical rotation data with reported data.[7]

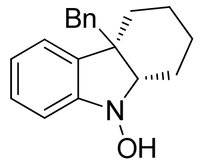

(4a*R*,9a*S*)-4a-Benzyl-2,3,4,4a-tetrahydro-1*H*-carbazol-9(9aH)-ol (7i)
^{1}H NMR (500 MHz, $CDCl_3$) δ 7.25–6.93 (m, 9H), 6.11 (brs, 1H), 3.24–3.12 (m, 2H), 3.08 (d, *J* = 14.0 Hz, 1H), 2.02–1.94 (m, 1H), 1.75–1.44 (m, 7H); ^{13}C NMR (126 MHz, $CDCl_3$) δ 151.5, 138.2, 136.2, 130.8, 129.6, 128.2, 127.6, 126.5, 122.6, 114.3, 72.4, 45.9, 41.0, 35.7, 23.3, 21.9, 21.0; HPLC: the ee value was determined by HPLC analysis (Chiralpak IG, *i*-PrOH/Hexane = 5/95, 1.0 mL/min, 255 nm), retention time:

t_{minor} = 10.340 min, t_{major} =12.700 min, ee = 90%; $[\alpha]_D^{25}$ = + 65.1 (c = 0.50, THF). HRMS *m/z* $[M + H]^+$ calculated for $C_{19}H_{22}NO$: 280.1696, found 280.1699.

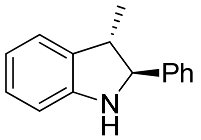

(2*S*,3*S*)-3-Methyl-2-phenylindoline (6j)

It was prepared following the general procedure and purified by silica gel flash chromatography using ethyl acetate/ petroleum ether (10:90) as eluent to afford **6j** (8.8 mg, 42% yield). ^{1}H NMR (500 MHz, $CDCl_3$) δ 7.55–7.48 (m, 2H), 7.42–7.30 (m, 3H), 7.16–7.04 (m, 2H), 6.86–6.75 (m, 1H), 6.70 (d, *J* = 7.7 Hz, 1H), 4.43 (d, *J* = 9.8 Hz, 1H), 3.23–3.15 (m, 1H), 1.41–1.36 (m, 3H); ^{13}C NMR (126 MHz, $CDCl_3$) δ 150.5, 143.6, 133.3, 128.7, 127.9, 127.8, 127.2, 123.4, 119.1, 109.1, 73.1, 46.7, 17.1; HPLC: the ee value was determined by HPLC analysis (Chiralcel OD-H, *i*-PrOH/Hexane = 20/80, 1.0 mL/min, 302 nm), retention time: t_{major} = 8.217 min, t_{minor} = 13.220 min, ee = 84%. HRMS *m/z* $[M + H]^+$ calculated for $C_{15}H_{16}N$: 210.1277, found 210.1272.

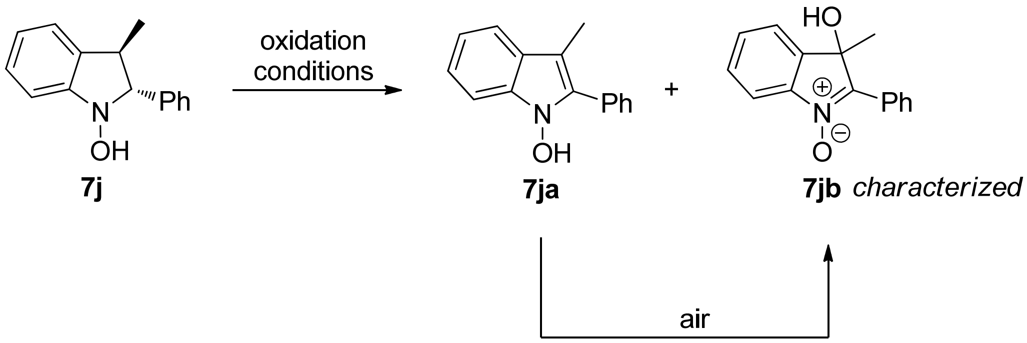

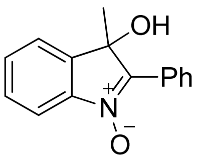

3-Hydroxy-3-methyl-2-phenyl-3*H*-indole 1-oxide (7jb)

Under the oxidation conditions, the oxidized hydroxylamine **7j** was further oxidized to **7ja** and **7jb.** During the characterization of **7ja**, **7ja** was further oxidized to **7jb** in air. ^{1}H NMR (500 MHz, $CDCl_3$) δ 8.53 (d, *J* = 7.5 Hz, 2H), 7.46 (d, *J* = 7.3 Hz, 1H), 7.32 (t, *J* = 7.2 Hz, 1H), 7.29–7.26 (m, 2H), 7.20 (t, *J* = 7.4 Hz, 1H), 7.07 (d, *J* = 7.8 Hz, 1H), 6.89 (t, *J* = 7.6 Hz, 1H), 1.60 (s, 3H); ^{13}C NMR (126 MHz, $CDCl_3$) δ 148.9, 143.8, 139.0, 131.0, 129.5, 129.4, 128.8, 128.3, 127.0, 121.5, 114.6, 79.0, 25.7. HRMS *m/z* $[M + H]^+$ calculated for $C_{15}H_{14}NO_2$: 240.1019, found 240.1011.

Synthetic Applications

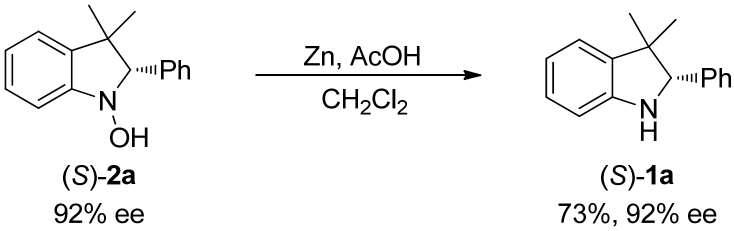

To a solution of (*S*)-**2a** (12 mg, 0.05 mmol) in CH_2Cl_2 (1.0 mL) was added AcOH (0.18 mL, 10 mmol) and Zn dust (24 mg, 0.375 mmol) at 0 °C. The mixture was stirred vigorously at 30 °C. When the reaction was completed, the reaction mixture was treated with saturated aqueous solution of ammonium chloride, and extracted with dichloromethane. The combined organic layer was washed with brine, dried over anhydrous sodium sulfate, and concentrated under vacuum. The crude residue was purified by flash column chromatography (EtOAc/petroleum ether 95:5) on silica gel to afford (*S*)-**1a** (8.1 mg, 73 yield, 92% ee). HPLC: the ee value was determined by HPLC analysis (Chiralpak IB, *i*-PrOH/Hexane = 10/90, 1.0 mL/min, 247 nm), retention time: t_{minor} = 5.763 min, t_{major} = 11.113 min, ee = 92%; $[\alpha]_D^{25}$ = + 125.0 (c = 0.20, THF).

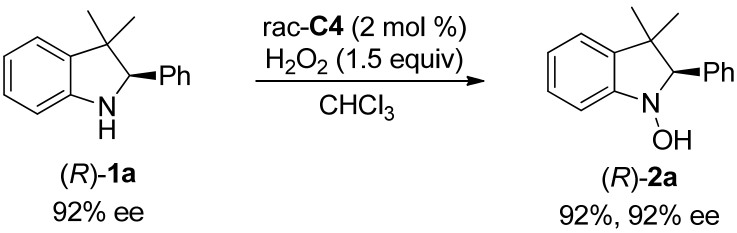

To a solution of (*R*)-**1a** (22.3 mg, 0.1mmol, 1.0 eq) in $CHCl_3$ (1.0 mL) was added 30% aqueous hydrogen peroxide (15 μL, 0.15 mmol, 1.5 eq) and rac-**C4** (2.1 mg, 0.002 mmol, 2 mmol%) at room temperature. The reaction was vigorously stirred for 3 h. Then the mixture was diluted with CH_2Cl_2 (20 mL), washed with water (10 mL), dried over $MgSO_4$, filtered and concentrated. The residue was purified by silica gel chromatography (EtOAc/petroleum ether 90:10) to give the desired product (*R*)-**2a** (22.0 mg, 92% yield). HPLC: the ee value was determined by HPLC analysis (Chiralpak IB, *i*-PrOH/Hexane = 5/95, 1.0 mL/min, 247 nm), retention time: t_{minor} = 6.493 min, t_{major} = 9.773 min, ee = 92%; $[\alpha]_D^{25}$ = – 165.5 (c = 0.25, THF).

Gram-scale experiment

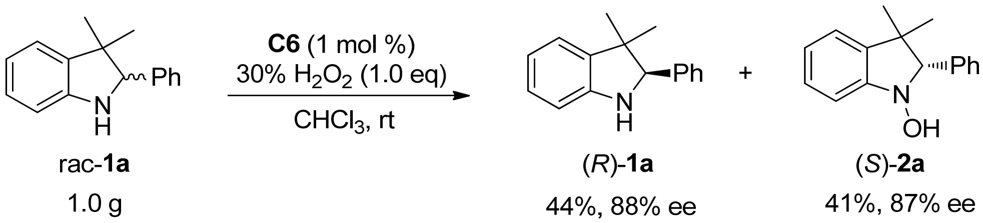

To a solution of (*R*)-**1a** (1.0 g, 4.48 mmol) in $CHCl_3$ (45 mL) was added 30% aqueous hydrogen peroxide (0.45 mL, 4.48 mmol) and **C6** (80 mg, 0.045 mmol) at room temperature. The reaction was vigorously stirred for 10 h. Then the mixture was diluted with CH_2Cl_2 (200 mL), washed with sat. Na_2SO_3 (100 mL), dried over $MgSO_4$, filtered and concentrated. The residue was purified by silica gel chromatography (EtOAc/petroleum ether 90:10) to give the desired product (*R*)-**1a** (440 mg, 92% yield, 88% ee) and (*S*)-**2a** (439 mg, 41% yield, 87% ee).

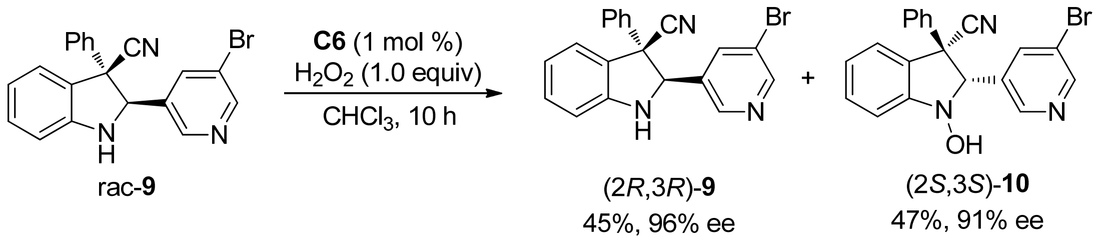

To a solution of rac-**9** (36.4 mg. 0.1mmol, 1.0 eq) in $CHCl_3$ (1.0 mL) was added 30% aqueous hydrogen peroxide (0.1 mmol, 10 μL, 1.0 eq) and **C6** (1.8 mg, 1 mmol%) at room temperature. The reaction was vigorously stirred for 10 h. Then the mixture was diluted with CH_2Cl_2 (20 mL), washed with water (10 mL), dried over $MgSO_4$, filtered and concentrated. The residue was purified by silica gel chromatography (EtOAc/petroleum ether 70:30) to give product (2*R*,3*R*)-**9** (16.9 mg, 45% yield) and (2*S*,3*R*)-**10** (18.4 mg, 47% yield).

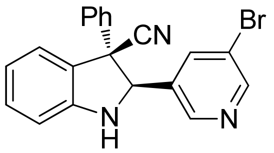

(2*R*,3*R*)-2-(5-Bromopyridin-3-yl)-3-phenylindoline-3-carbonitrile (9)
^{1}H NMR (500 MHz, $CDCl_3$) δ 8.69 (s, 1H), 8.14 (s, 1H), 8.07 (s, 1H), 7.59–7.42 (m, 3H), 7.42–7.34 (m, 2H), 7.30 (td, *J* = 7.8, 1.2 Hz, 1H), 7.05 (dd, *J* = 7.5, 0.6 Hz, 1H), 6.97–6.87 (m, *J* = 10.6, 5.7, 1.9 Hz, 2H), 5.04 (s, 1H), 4.38 (brs, 1H); ^{13}C NMR (126 MHz, $CDCl_3$) δ 151.6, 150.0, 147.1, 138.4, 136.5, 134.3, 130.8, 129.4, 129.3, 127.8, 126.8, 126.0, 121.5, 121.1, 118.4, 111.2, 74.9, 58.5; HPLC: the ee value was determined by HPLC analysis (Chiralcel OD-H, *i*-PrOH/Hexane = 20/80, 1.0 mL/min, 246 nm), retention time: t_{major} = 8.997 min, t_{minor} = 10.580 min, ee = 96%; $[\alpha]_D^{25}$ = –8.5 (c = 0.40, THF). HRMS *m*/*z* $[M + H]^+$ calculated for $C_{20}H_{15}BrN_3$: 378.0429, found 378.0422. These data are consistent with reported literature values.[9]

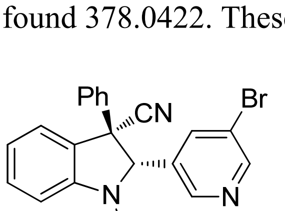

(2*S*,3*S*)-2-(5-Bromopyridin-3-yl)-1-hydroxy-3-phenylindoline-3-carbonitrile (10)
^{1}H NMR (500 MHz, DMSO) δ 9.91 (s, 1H), 8.76 (d, *J* = 2.2 Hz, 1H), 8.17 (d, *J* = 1.7 Hz, 1H), 7.94 (t, *J* = 1.9 Hz, 1H), 7.62–7.39 (m, 4H), 7.39–7.28 (m, 2H), 7.21 (d, *J* =

7.8 Hz, 1H), 7.12 (td, J = 7.5, 0.9 Hz, 1H), 7.00 (d, J = 7.2 Hz, 1H), 4.90 (s, 1H); ^{13}C NMR (126 MHz, DMSO) δ 152.8, 150.8, 147.7, 138.3, 134.0, 131.9, 130.7, 129.3, 129.2, 127.5, 125.1, 124.4, 123.8, 119.8, 118.2, 113.9, 82.19, 55.19; HPLC: the ee value was determined by HPLC analysis (Chiralpak IB, *i*-PrOH/Hexane = 10/90, 1.0 mL/min, 245 nm), retention time: t_{major} = 6.093 min, t_{minor} = 8.353 min, ee = 91%; $[\alpha]_D^{20}$ = + 70.5 (c = 0.2, THF). HRMS *m/z* $[M + H]^+$ calculated for $C_{20}H_{15}BrN_3O$: 394.0378, found 394.0374.

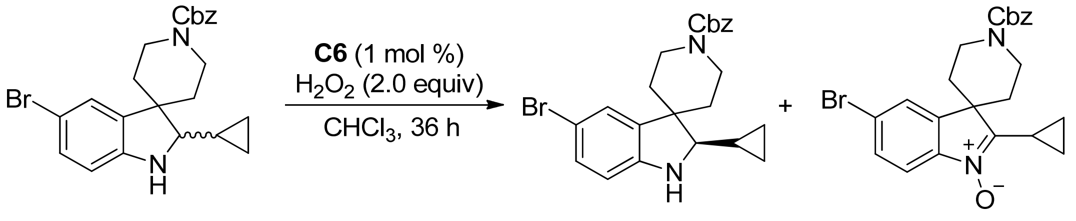

rac-**12** (*R*)-**12** 49%, 83% ee **13** 45%

To a solution of rac-**12** (44.1 mg, 0.1mmol, 1.0 eq)[10] in $CHCl_3$ (1.0 mL) was added 30% aqueous hydrogen peroxide (20 μL, 0.2 mmol, 2.0 eq) and **C6** (1.8 mg, 1 mmol%) at room temperature. The reaction was vigorously stirred for 36 h. Then the mixture was diluted with CH_2Cl_2 (20 mL), washed with water (10 mL), dried over $MgSO_4$, filtered and concentrated. The residue was purified by silica gel chromatography (EtOAc/petroleum ether 95:5) to give product (*R*)-**12** (21.6 mg, 49% yield) and **13** (20.5 mg, 45% yield).

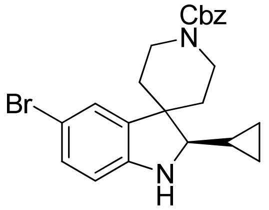

(*R*)-Benzyl 5-bromo-2-cyclopropylspiro[indoline-3,4'-piperidine]-1'-carboxylate (12)

^{1}H NMR (500 MHz, $CDCl_3$) δ 7.17–7.08 (m, 5H), 7.00 (d, J = 2.0 Hz, 1H), 6.92 (dd, J = 8.2, 2.0 Hz, 1H), 6.38–6.24 (m, 1H), 4.95 (s, 2H), 3.72–3.57 (m, 2H), 3.29–3.14 (m, 2H), 2.55 (d, J = 9.6 Hz, 1H), 1.92–1.83 (m, 1H), 1.73–1.53 (m, 2H), 1.44–1.35 (m, 1H), 0.80–0.71 (m, 1H), 0.51–0.37 (m, 1H), 0.35–0.27 (m, 1H), 0.17–0.08 (m, 1H), 0.05–-0.07 (m, 1H); ^{13}C NMR (126 MHz, $CDCl_3$) δ 155.6, 148.1, 138.3, 137.0, 130.7, 128.7, 128.2, 128.1, 126.8, 111.3, 110.6, 73.39, 67.38, 47.07, 41.71, 41.11, 35.93, 30.50, 12.76, 4.75, 2.5; HPLC: the ee value was determined by HPLC analysis (Chiralcel AD-H, *i*-PrOH/Hexane = 20/80, 1.0 mL/min, 316 nm), retention time: t_{minor} = 10.993 min, t_{major} = 11.627 min, ee = 83%; $[\alpha]_D^{25}$ = + 85.6 (c = 0.5, THF). HRMS *m/z* $[M + H]^+$ calculated for $C_{23}H_{26}BrN_2O_2$: 443.1157, found 443.1165.

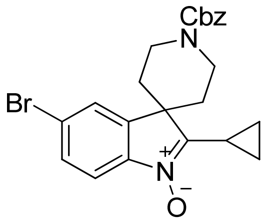

1'-((Benzyloxy)carbonyl)-5-bromo-2-cyclopropylspiro[indole-3,4'-piperidine] 1-oxide (13)

^{1}H NMR (500 MHz, CD_3CN) δ 8.05 (s, 1H), 7.71–7.62 (m, 1H), 7.50 (d, J = 8.3 Hz, 1H), 7.49–7.35 (m, 4H), 7.35–7.24 (m, 1H), 5.16 (s, 2H), 4.21 (d, J = 14.0 Hz, 2H), 3.42 (brs, 2H), 2.15 (td, J = 13.1, 4.6 Hz, 2H), 2.03 (dt, J = 6.2, 3.1 Hz, 2H), 1.66–1.60 (m, 1H), 1.49 (d, J = 12.6 Hz, 2H), 1.02–0.94 (m, 2H); ^{13}C NMR (126 MHz, CD_3CN) δ 156.2, 155.5, 141.4, 138.3, 132.5, 129.4, 128.9, 128.7, 127.9, 121.8, 116.5, 67.7, 50.9, 41.0, 30.9, 9.2, 5.4; HRMS m/z $[M + H]^+$ calculated for $C_{23}H_{24}BrN_2O_3$: 457.0950, found 457.0958.

Mechanism studies

Kinetic studies

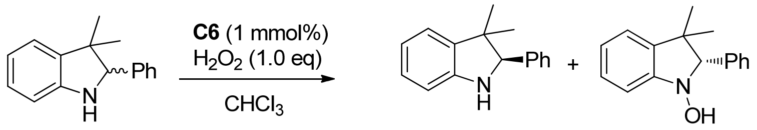

rac-**1a** (*R*)-**1a** (*S*)-**2a**

To a solution of rac-**1a** (0.1mmol, 22.3 mg) in $CHCl_3$ (1.0 mL) was added 30% aqueous hydrogen peroxide (0.1 mmol, 10 μL) and **C6** (0.001 mmol, 1.8 mg) at room temperature. Then, 5 μL aliquots of reaction mixture were periodically taken (every 10\20\30\60 min) and the $ee_{\mathbf{1a}}$ and $ee_{\mathbf{2a}}$ were measured by chiral HPLC (Chiralpak IB, *i*-PrOH/Hexane = 10/90, 1.0 mL/min, 247 nm; Chiralpak IB, *i*-PrOH/Hexane = 5/95, 1.0 mL/min, 247 nm).

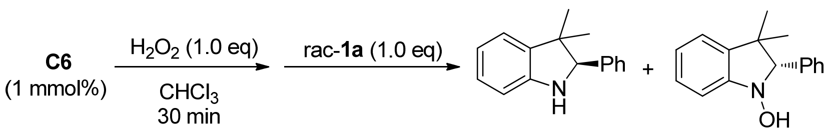

(*R*)-**1a** (*S*)-**2a**

To a solution of **C6** (0.001 mmol, 1.8 mg) in $CHCl_3$ (1.0 mL) was added 30% aqueous hydrogen peroxide (0.1 mmol, 10 μL) at room temperature. The mixture was vigorously stirred for 30 min and **1a** (0.1 mmol, 22.3 mg) was added. Then, 5 μL aliquots of reaction mixture were periodically taken (every 10\20\30\60 min) and the $ee_{\mathbf{1a}}$ and $ee_{\mathbf{2a}}$ were measured by chiral HPLC (Chiralpak IB, *i*-PrOH/Hexane = 10/90, 1.0 mL/min, 247 nm; Chiralpak IB, *i*-PrOH/Hexane = 5/95, 1.0 mL/min, 247 nm). Kinetic analysis revealed apparent pseudo first order dependence of the reaction rate on the concentration of (*S*)-**1a** (0-40% conversion).

$$\ln([S_S]/[S_S]_0) = -0.0118t - 0.14$$

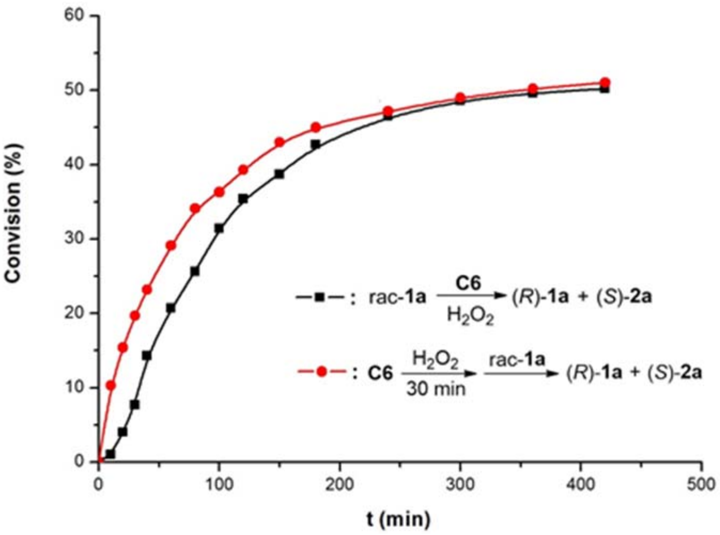

Supplementary figure 1. Kinetic plots for the oxygenation of rac-**1a** with **C6**/H_2O_2. Conversion = ($ee_{\mathbf{1a}}$)/ ($ee_{\mathbf{1a}}$ + $ee_{\mathbf{2a}}$).

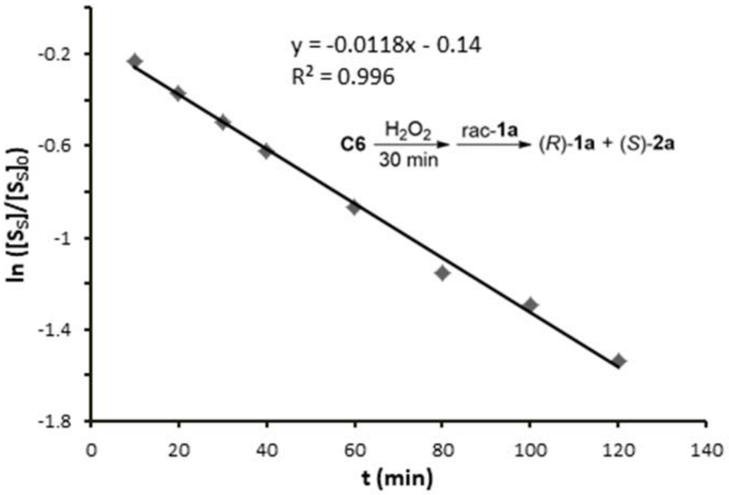

Supplementary figure 2. ln($[S_S]/[S_S]_0$) versus time dependences for the oxygenation of (*S*)-**1a.** $[S_S]_0$ = [(*S*)-**1a**]$_0$ = 0.05 M, $[S_S]$ = [(*S*)-**1a**] ≈ [0.5 – C][rac-**1a**]$_0$, [rac-**1a**]$_0$ = 0.1 M, C = ($ee_{\mathbf{1a}}$)/ ($ee_{\mathbf{1a}}$ + $ee_{\mathbf{2a}}$).

Correlation of the enantiomeric excess of C6 and 2a

The $CHCl_3$ solutions of **C6** and ent-**C6** (0.001 M, respectively) were prepared and mixed to regulate each complex solution (0% ee, 10%ee, 20% ee, 40% ee, 60% ee, 80% ee and 100% ee, 0.001 M, respectively) in an appropriate manner. To the solutions, **1a** (0.1 mmol, 22.3 mg) and 30% aqueous hydrogen peroxide (0.1 mmol, 10 μL) were added. After stirring for 6 h at room temperature (50% conversion of **1a**), the reaction mixture was diluted with CH_2Cl_2 (20 mL), washed with water (10 mL), dried over $MgSO_4$, filtered and concentrated. The residue was purified by silica gel flash chromatography using EtOAc/petroleum ether (10:90) as eluent. The ee values of **2a** were determined by HPLC analysis on chiral phase column (Chiralpak IB, *i*-PrOH/Hexane = 5/95, 1.0 mL/min, 247 nm).

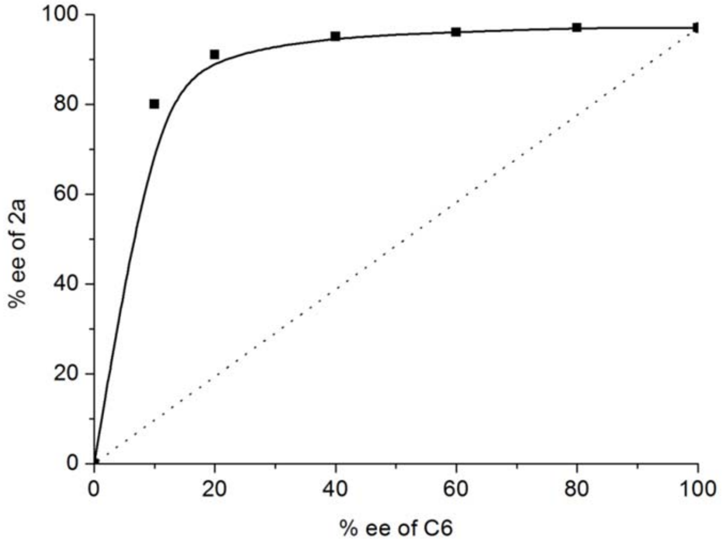

Supplementary figure 3. Plot of the enantiomeric excess of **2a** versus the enantiomeric excess of **C6** at 50% conversion. The dotted line symbolizes the linear correlation.

.

Control Experiments

ESI-MS analysis of the mixture of C6 and H_2O_2

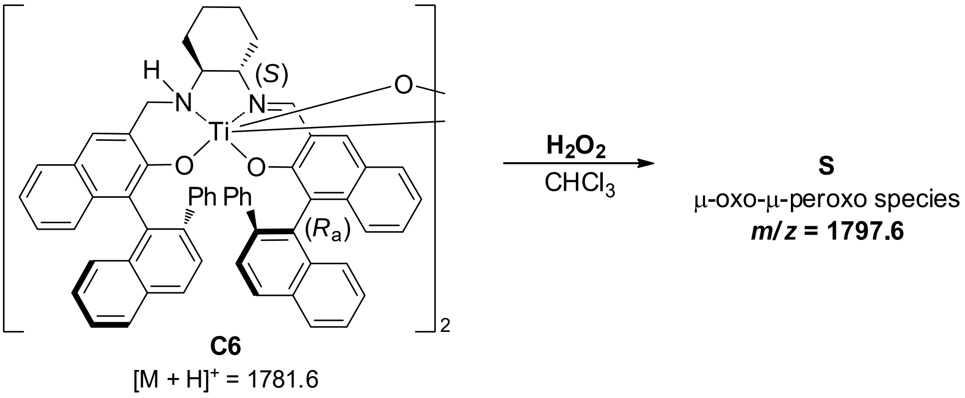

To a solution of **C6** (0.01 mmol, 18 mg) in $CHCl_3$ (10 mL) was added 30% aqueous hydrogen peroxide (1 mmol, 100 μL) at room temperature. The mixture was vigorously stirred for 30 min. Analysis by ESI mass-spectrometry indicated a μ-oxo-μ-peroxo species **S** ($[M+H+O]^+$: *m*/*z*= 1797.6. Due to the isotope effect, a cluster of peaks appeared). After 1.5 h, solvent was removed under vacuum and the residue was purified by flash chromatography on silica gel using ethyl acetate/ CH_2Cl_2 (2:98) as eluent to give **S** (13.0 mg, 72% yield) with the same ion peak (*m*/*z*= 1797.6) in the mass spectrum. IR (KBr): 3435, 3228, 3051, 2924, 2853, 1634, 1617, 1586, 1493, 1452, 1423, 1350, 1260, 1114, 1015, 910, 791, 760, 701, 578, 507 cm^{-1}.

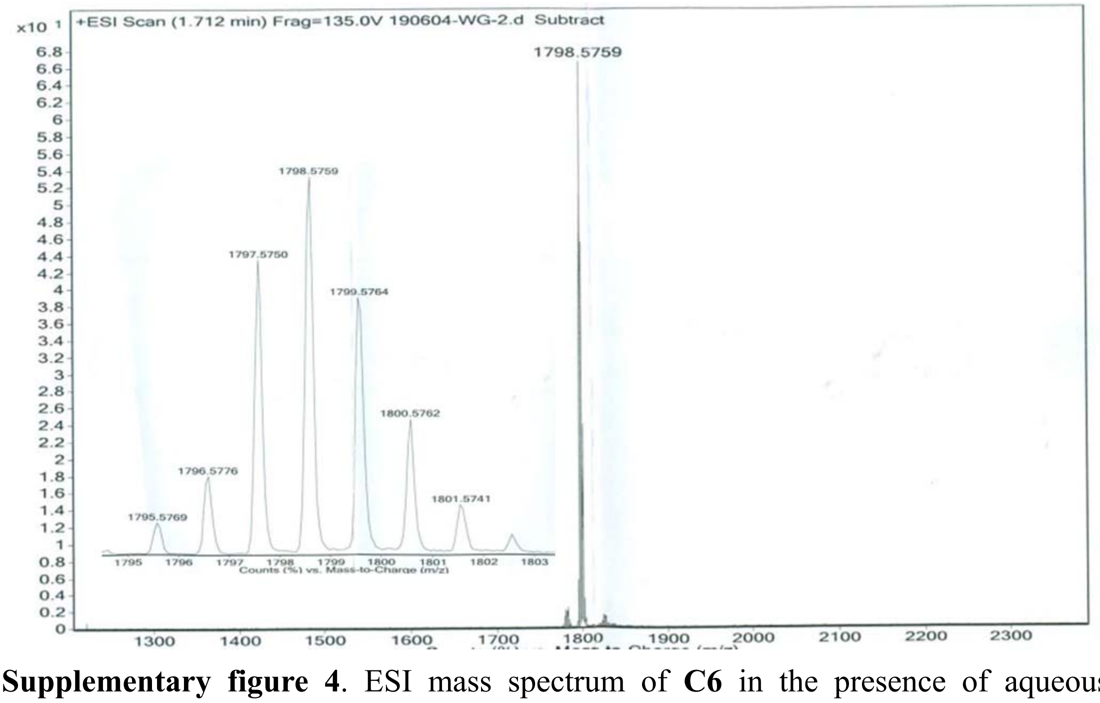

Supplementary figure 4. ESI mass spectrum of **C6** in the presence of aqueous hydrogen peroxide in $CHCl_3$.

The oxidation reactivity of stoichiometric S without H_2O_2

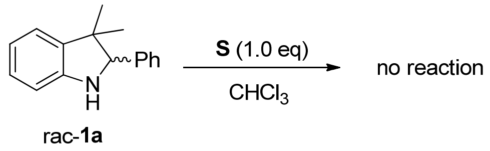

To a solution of rac-**1a** (0.05 mmol, 11.2 mg) in $CHCl_3$ (0.5 mL) was added **S** (0.05 mmol, 90 mg) at room temperature. The mixture was vigorously stirred for 24 h. No reaction occurred.

The oxidation catalysis reactivity of S with H_2O_2

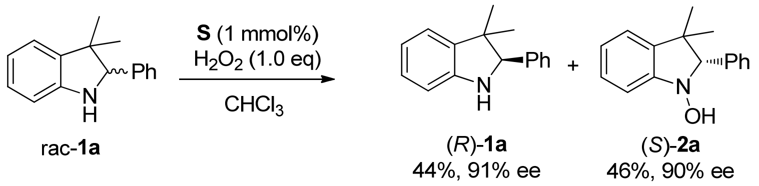

To a solution of rac-**1a** (22.3 mg, 0.1mmol, 1.0 eq) in $CHCl_3$ (1.0 mL) was added 30% aqueous hydrogen peroxide (10 µL, 0.1 mmol, 1.0 eq) and **S** (1.8 mg, 1 mmol%) at room temperature. The reaction was vigorously stirred for 5 h. Then the mixture was diluted with CH_2Cl_2 (20 mL), washed with water (10 mL), dried over $MgSO_4$, filtered and concentrated. The residue was purified by silica gel chromatography (EtOAc/petroleum ether 90:10) to give product (*R*)-**1a** (9.8 mg, 44% yield, 91% ee) and (*S*)-**2a** (11.0 mg, 46% yield, 90% ee).

ESI-MS analysis of the mixture of C4, C6 and H_2O_2

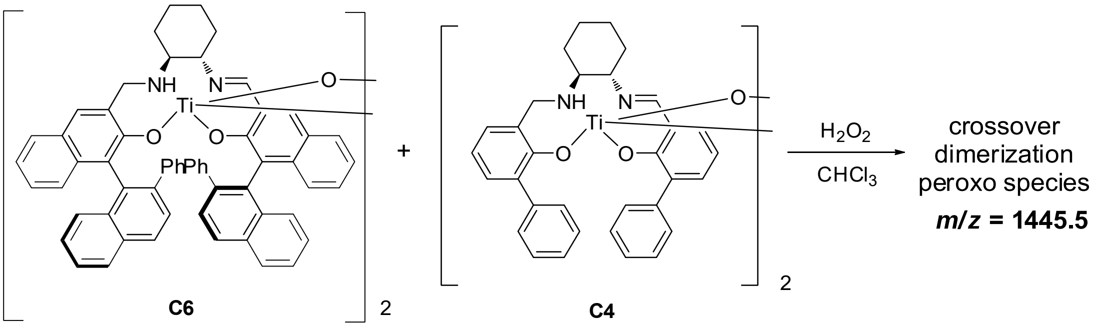

To a solution of **C6** (0.001 mmol, 1.8 mg) and **C4** (0.001 mmol, 1.1 mg) in $CHCl_3$ (1.0 mL) was added 30% aqueous hydrogen peroxide (0.1 mmol, 10 μL) at room temperature. The mixture was vigorously stirred for 1 h. Analysis by ESI mass-spectrometry indicated the generation of a crossover dimerization peroxo complex ($[M_{C6}/2+M_{C2}/2+H+O]^+$: *m*/*z* = 1445.5).

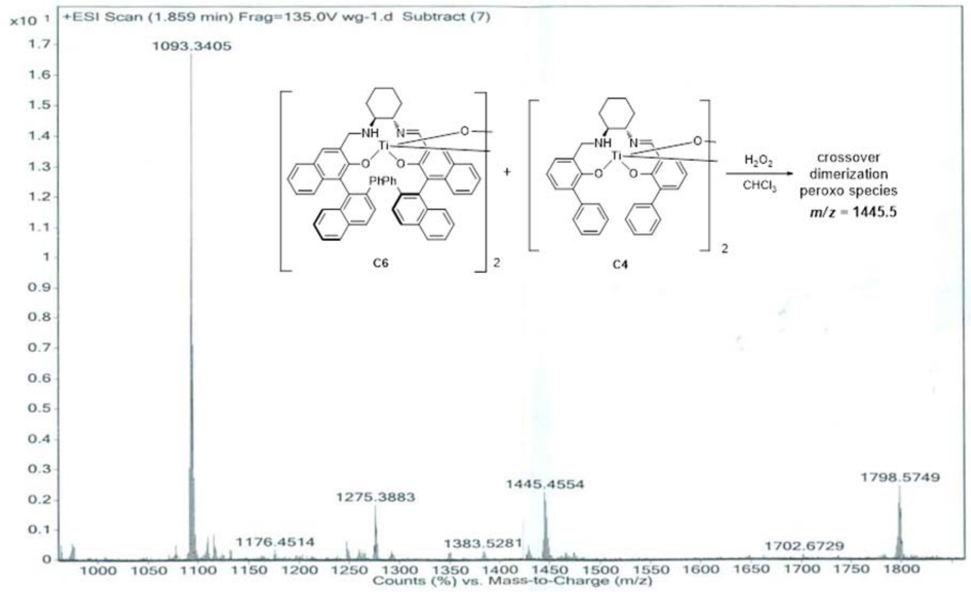

Supplementary figure 5. ESI mass spectrum of **C6** and **C4** in the presence of H_2O_2 in $CHCl_3$.

ESI-MS analysis of the mixture of C4 and C6 without H_2O_2

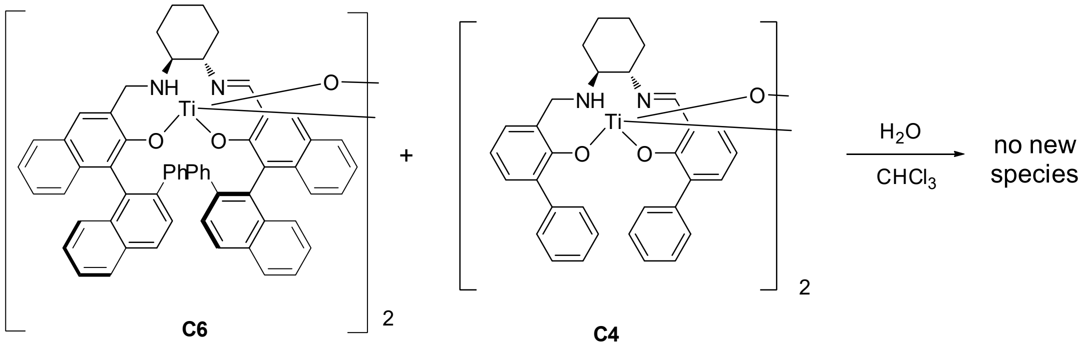

To a solution of **C6** (0.001 mmol, 1.8 mg) and **C4** (0.001 mmol, 1.1 mg) in $CHCl_3$ (1.0 mL) was added water (10 μL) at room temperature. The mixture was vigorously stirred for 1 h. No new species was detected by ESI mass-spectrometry.

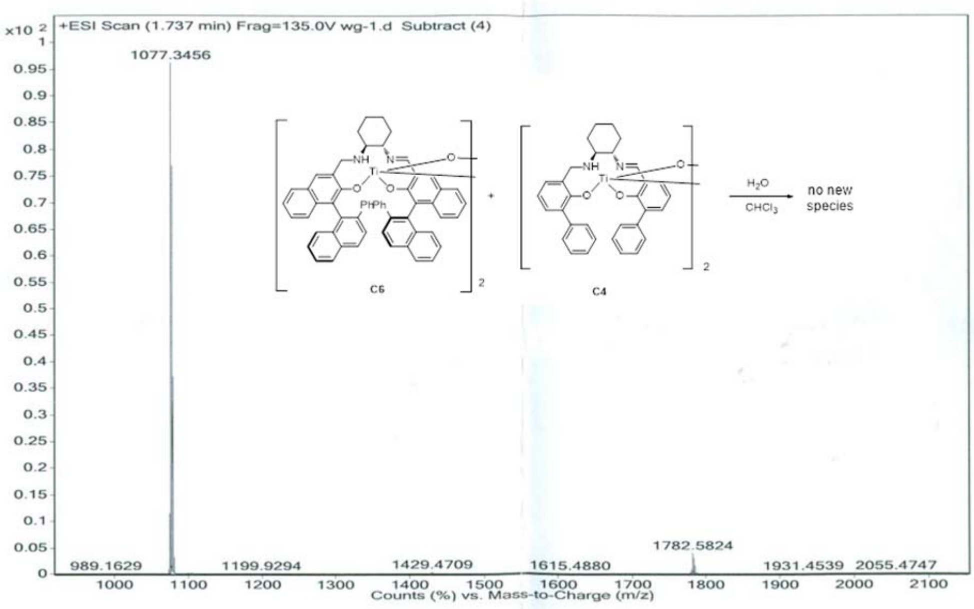

Supplementary figure 6. ESI mass spectrum of **C6** and **C4** in $CHCl_3$ without H_2O_2.

Hammett plot for the competitive oxidation

A mixture two different *p*-substituted **1** (**1a** and **4d**; **1a** and **4e**; **1a** and **4j**; **1a** and **4k**; 0.1 mmol each) was dissolved in $CHCl_3$ (1.0 mL). Then 30% aqueous hydrogen peroxide (0.1 mmol, 10 µL, 1.0 eq) and **C6** (1.8 mg, 1 mmol%) were added. After 1.5 h, the reaction was quenched with water (10 mL) at 15-35% conversion and the mixture was extracted with CH_2Cl_2 (20 mL). The solvent was removed and the residue was purified by silica gel chromatography to give the desired product. The results were summarized as follows:

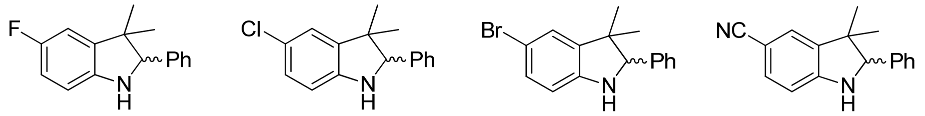

4d **4e** **4j** **4k**

entry	*p*-substituted X	$\log(k_X/k_H)$ [a]	σ [b]	σ^{+} [b]
1	F	0.204	0.15	-0.07
2	Cl	0.122	0.24	0.11
3	Br	0.0766	0.26	0.15
4	CN	-0.522	0.70	0.66

[a]Average of three experiments at 15-35% conversion.

[b]Data from: Anslyn, E. V.; Dougherty, D. A. (2006). Modern Physical Organic Chemistry, University science books.

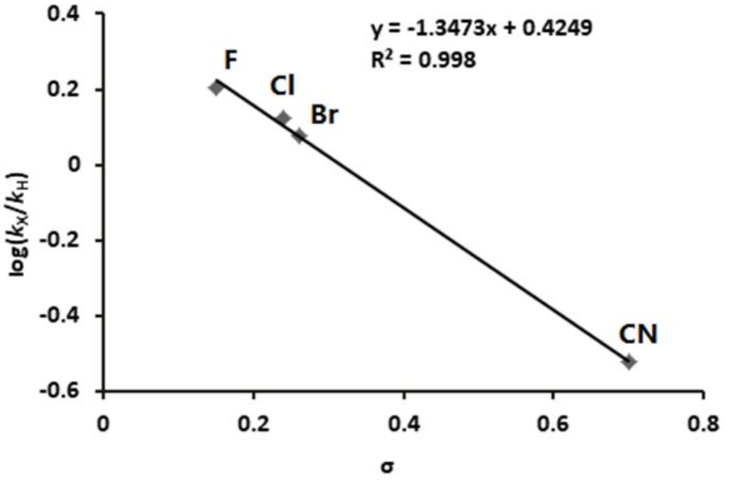

Supplementary figure 7. Hammett Plot of log(k_X/k_H) vs σ for the competition oxidation of C_5-substituted indolines by **C6**/H_2O_2.

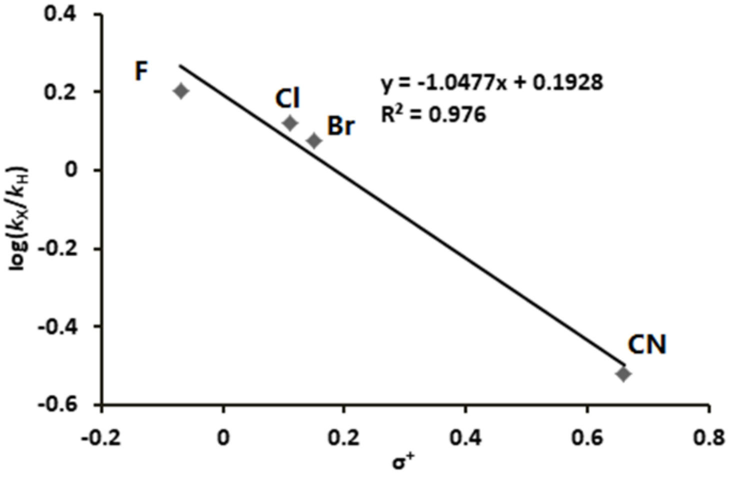

Supplementary figure 8. Hammett Plot of log(k_X/k_H) vs σ^+ for the competition oxidation of C_5-substituted indolines by **C6**/H_2O_2.

Supplementary date

NMR spectra

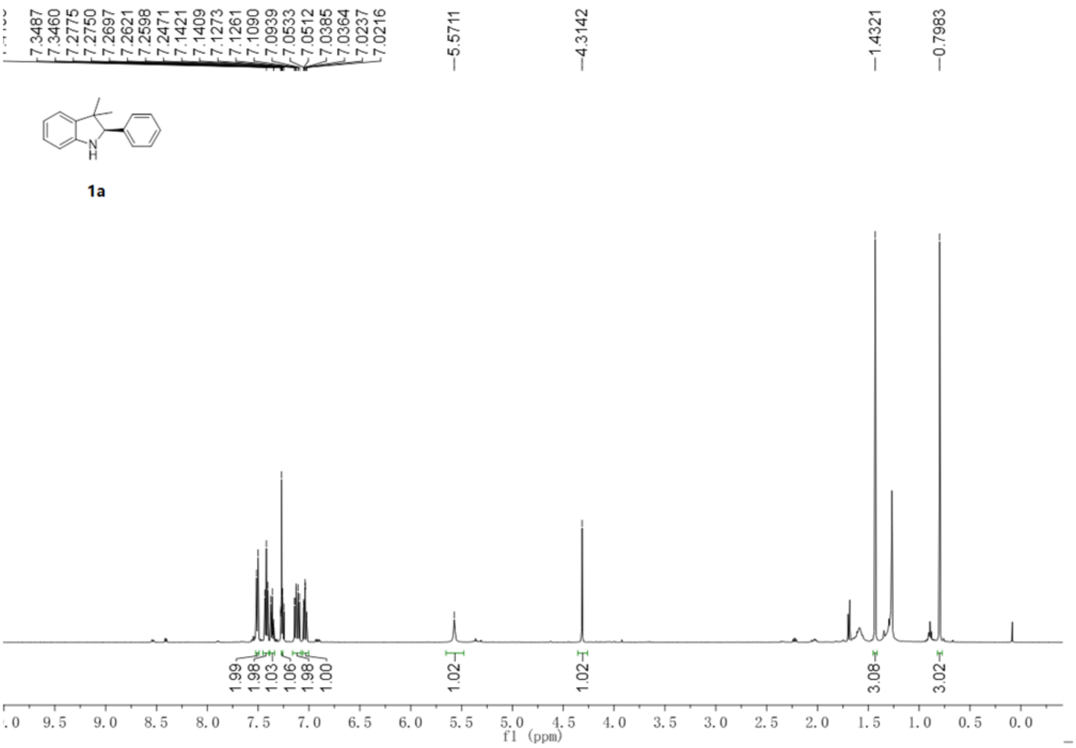

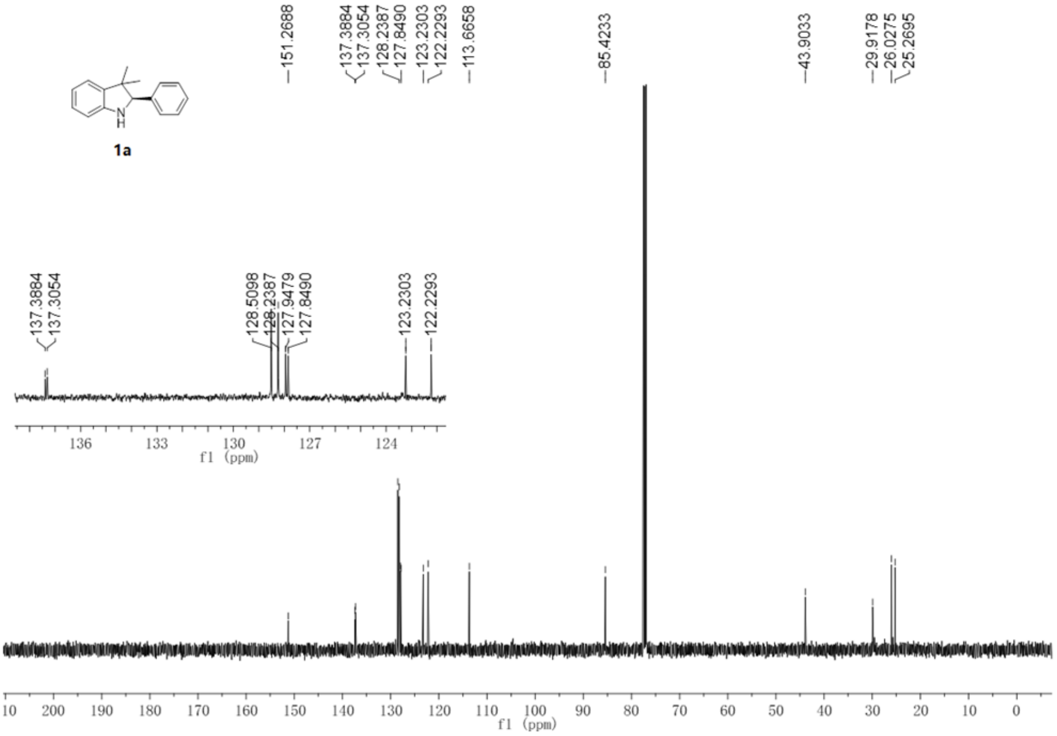

Supplementary figure 9. ^{1}H & ^{13}C NMR spectra of **1a**.

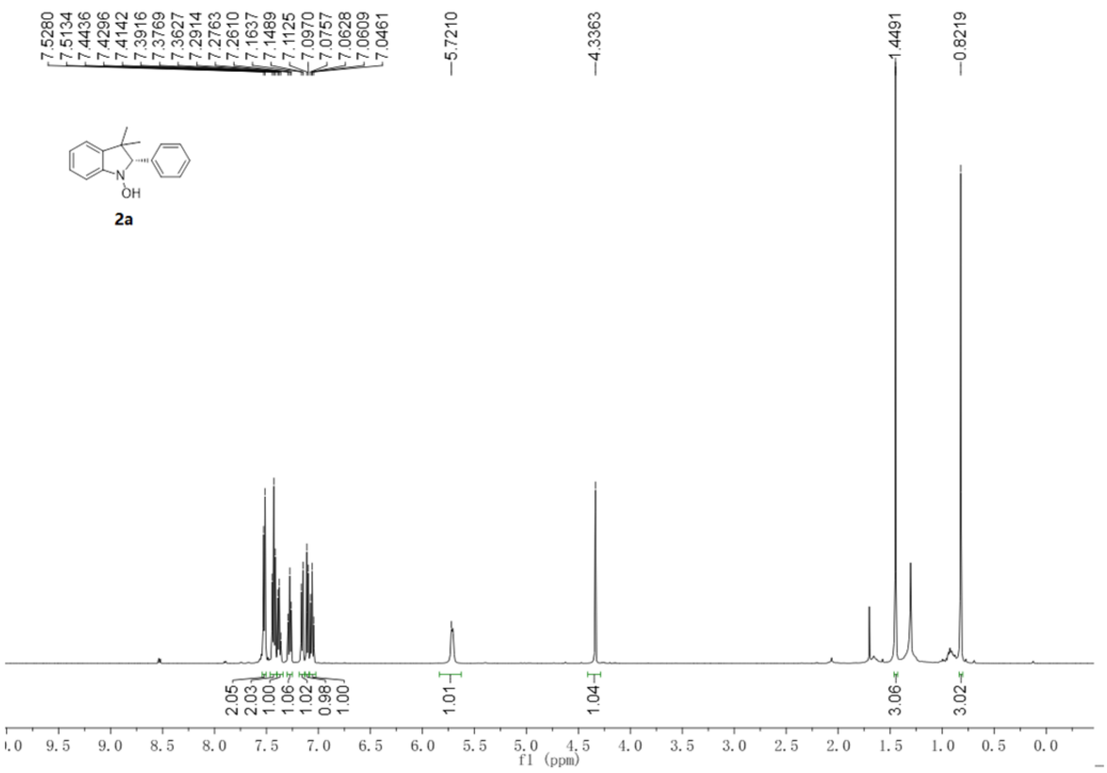

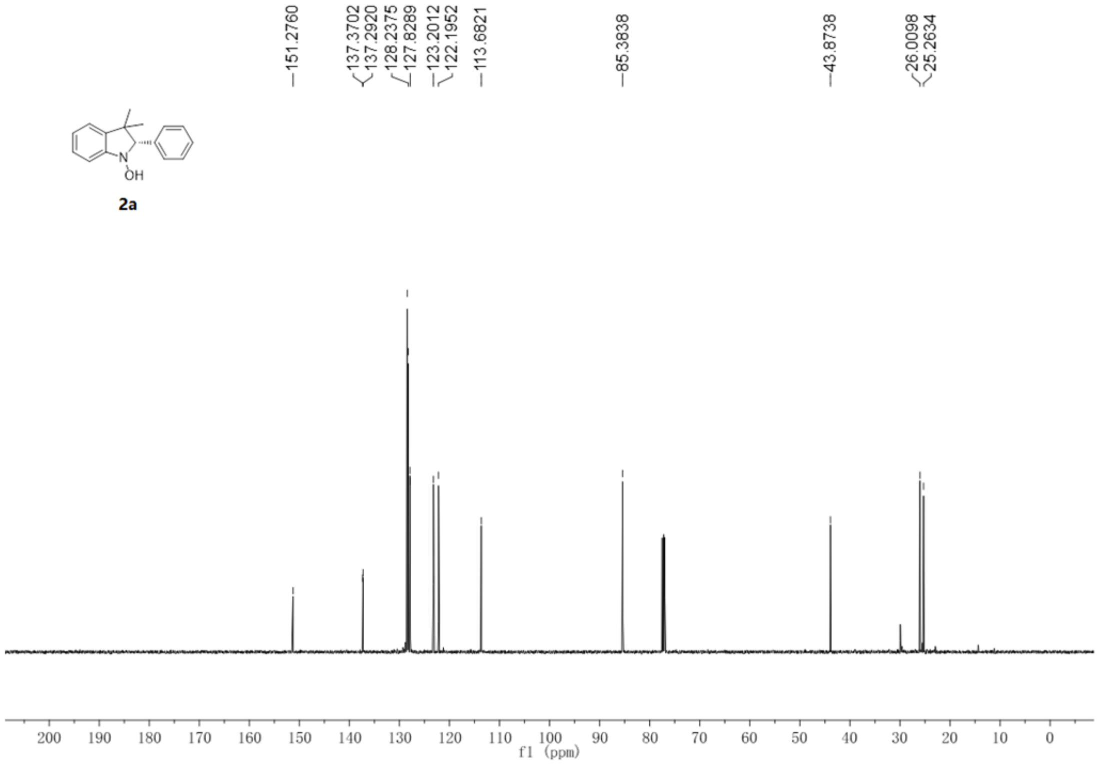

Supplementary figure 10. ^{1}H & ^{13}C NMR spectra of **2a**.

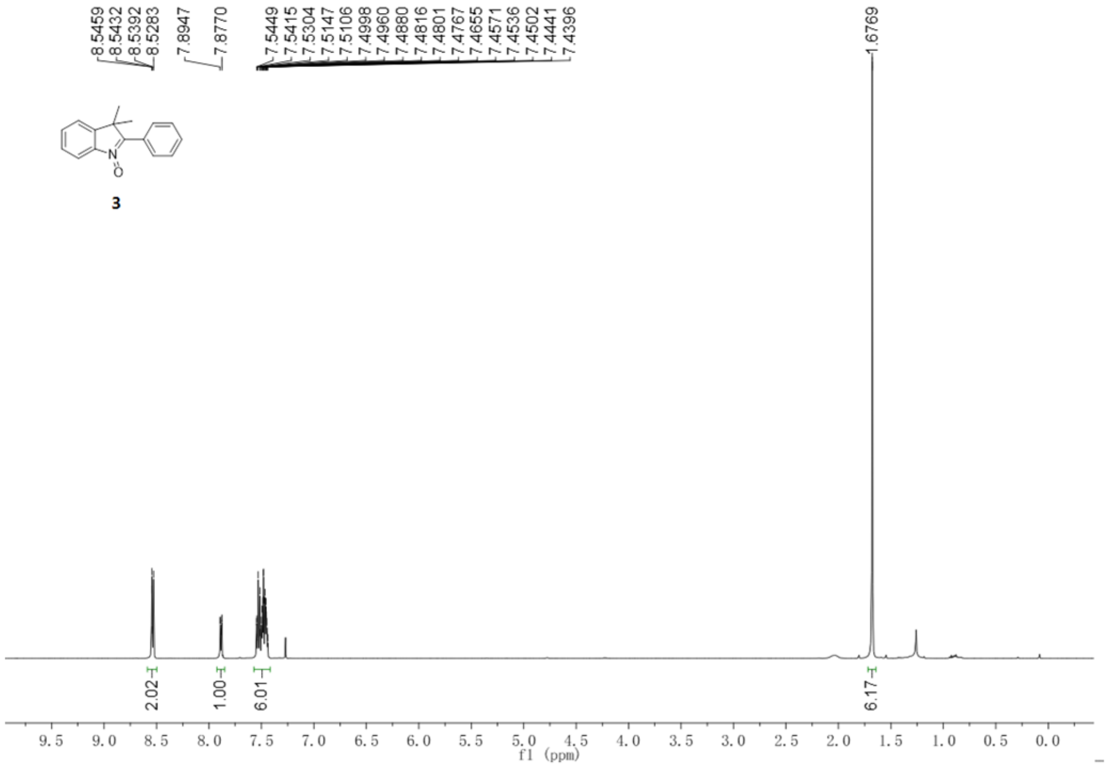

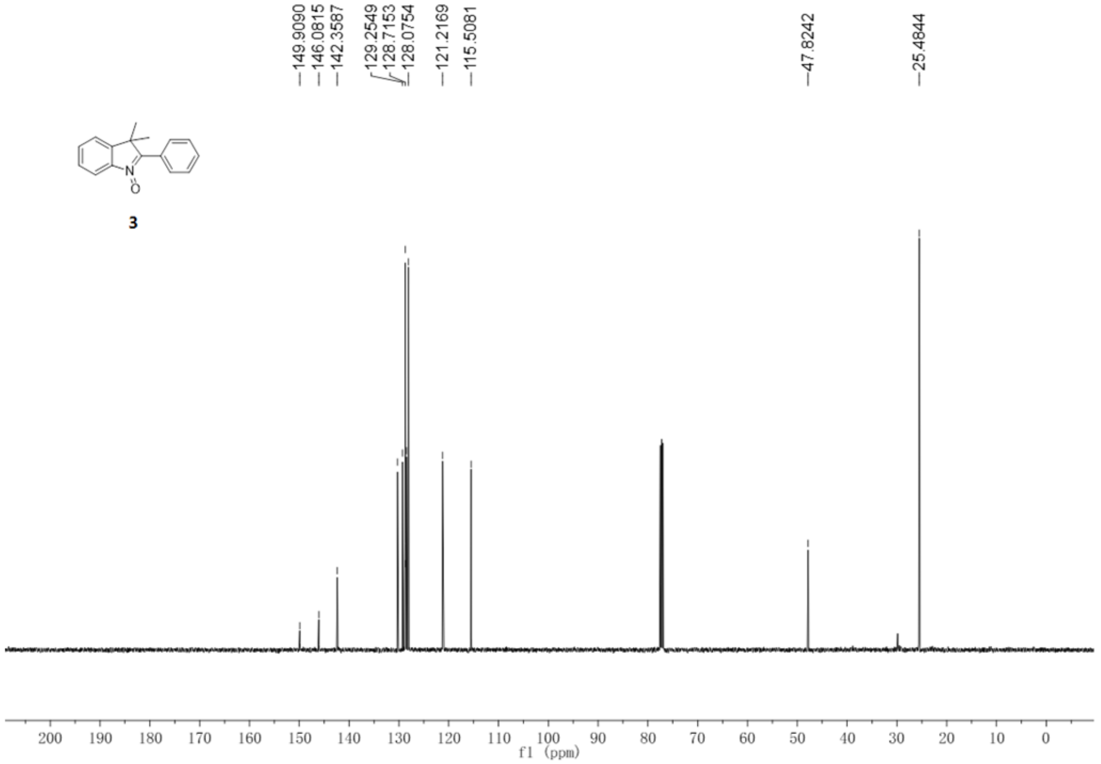

Supplementary figure 11. ^{1}H & ^{13}C NMR spectra of **3**.

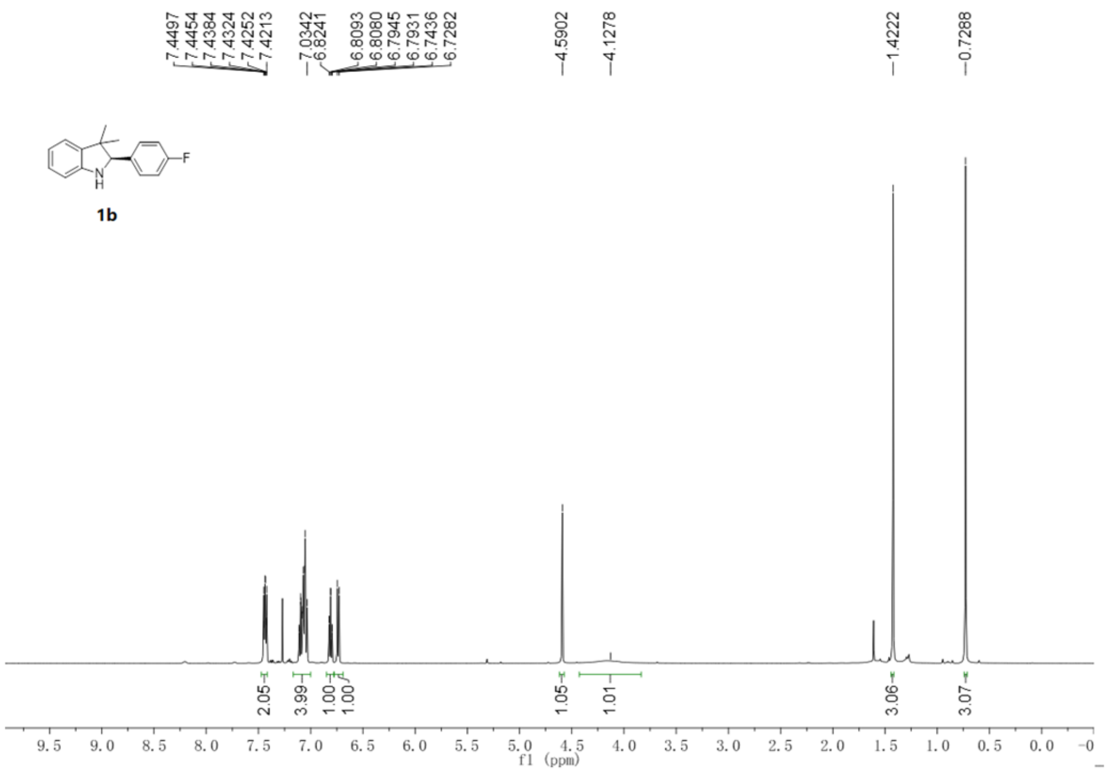

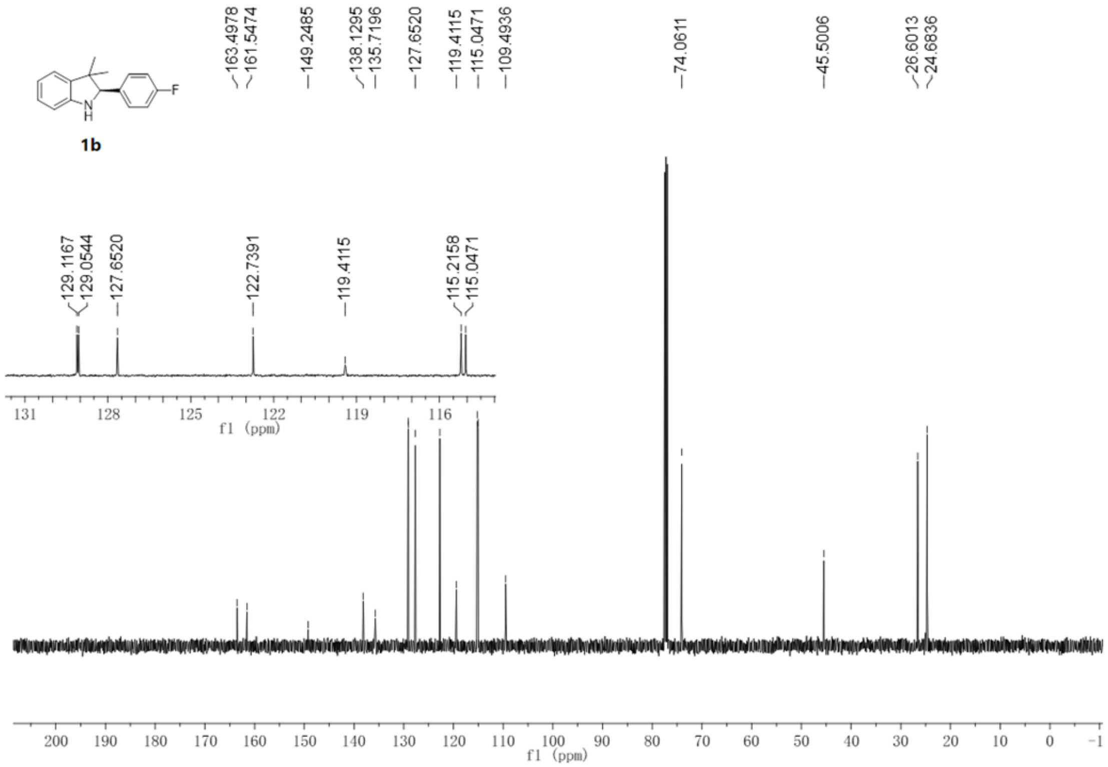

Supplementary figure 12. ^{1}H & ^{13}C NMR spectra of **1b**.

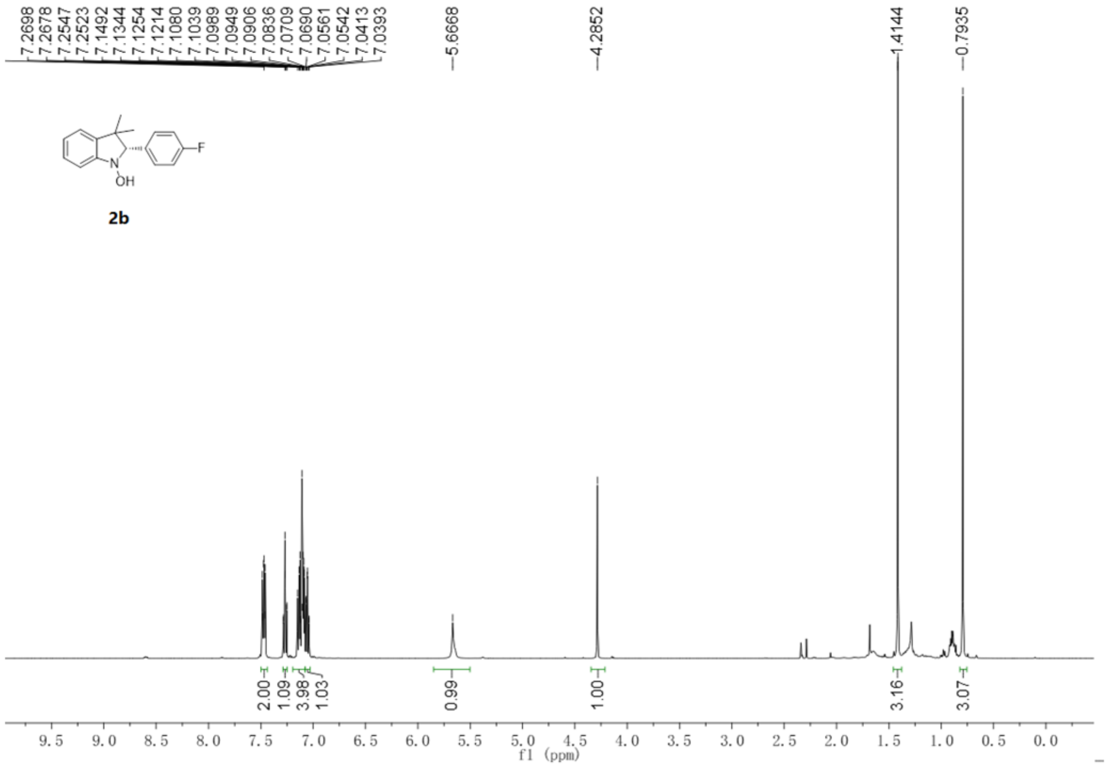

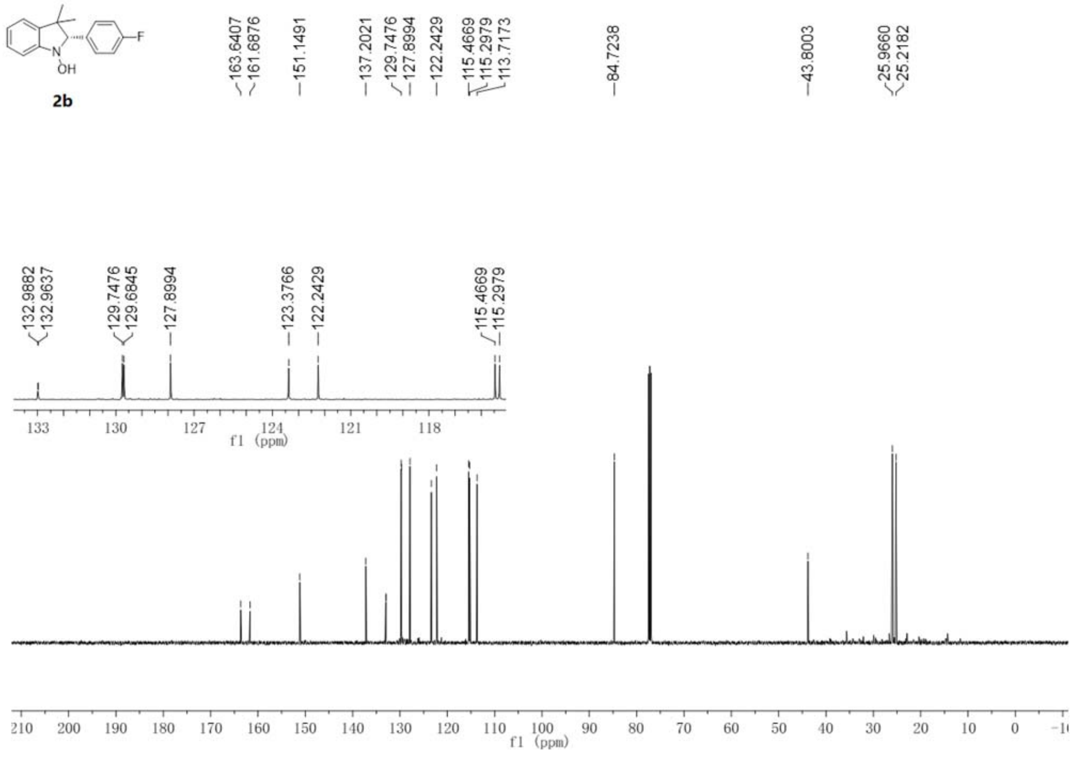

Supplementary figure 13. ^{1}H & ^{13}C NMR spectra of **2b**.

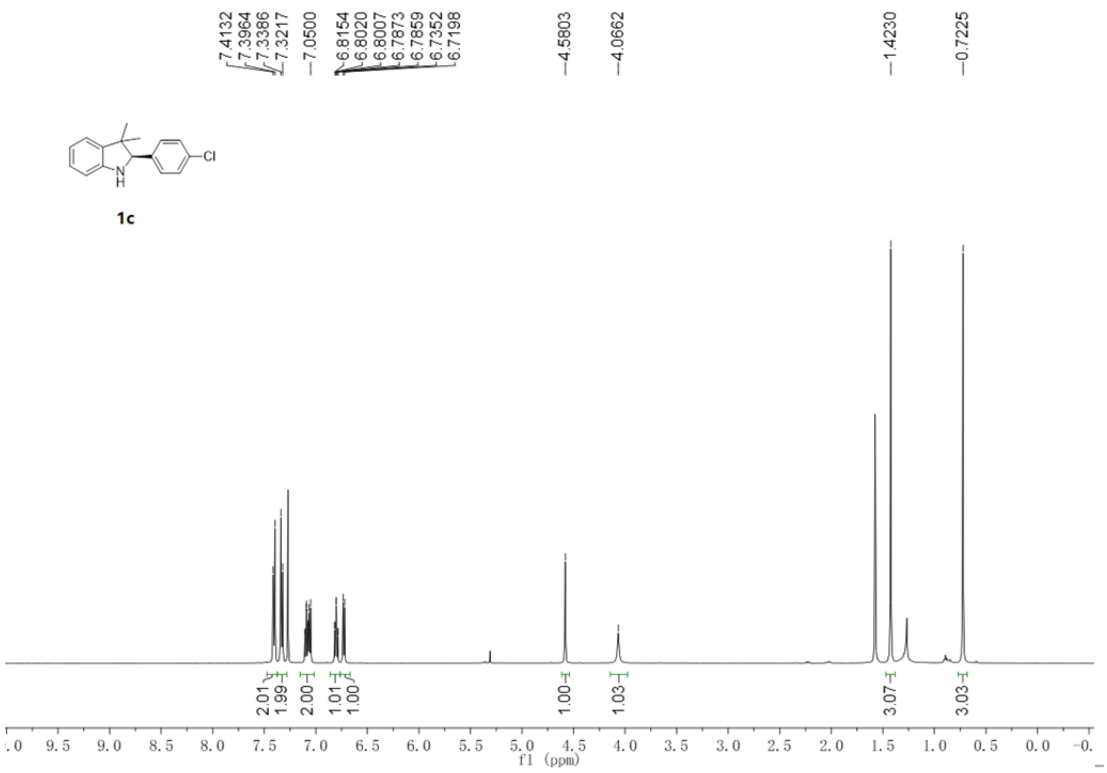

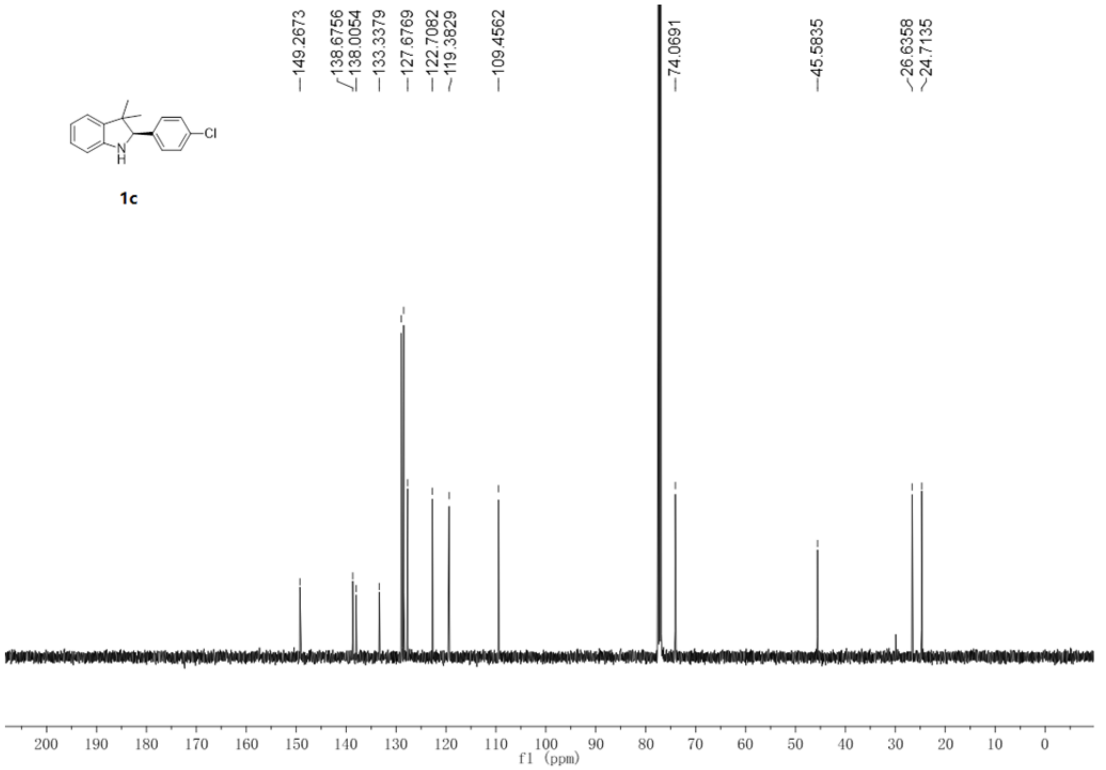

Supplementary figure 14. ^{1}H & ^{13}C NMR spectra of **1c**.

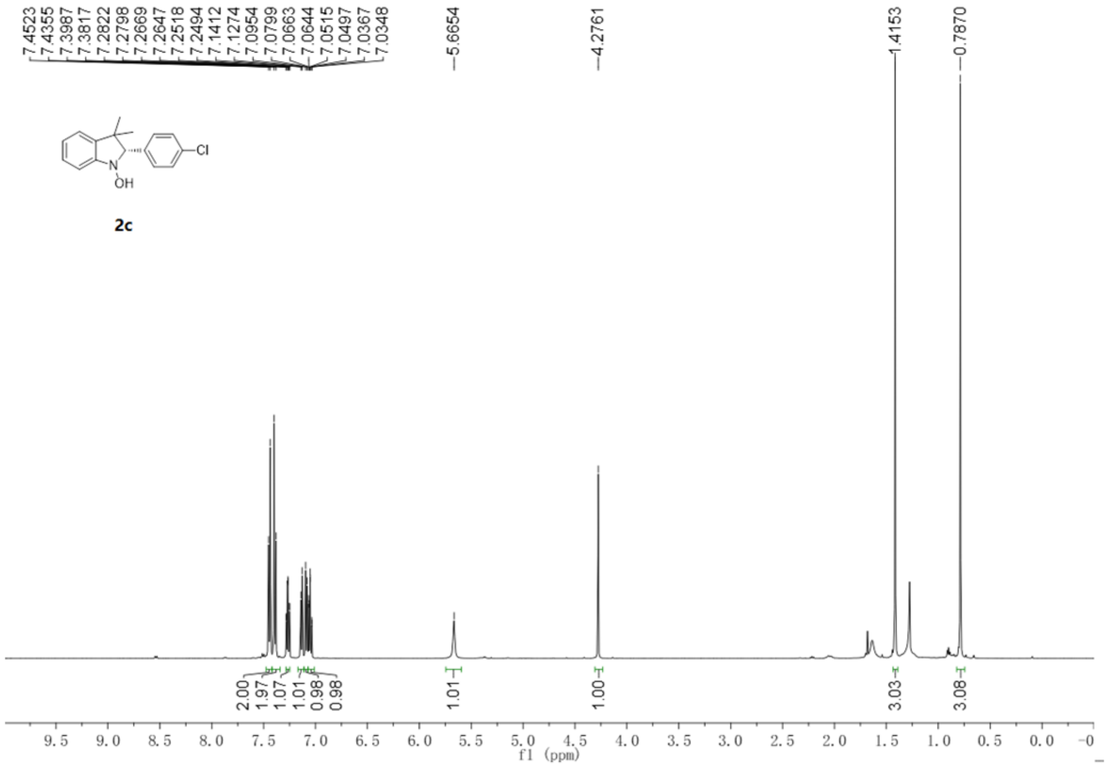

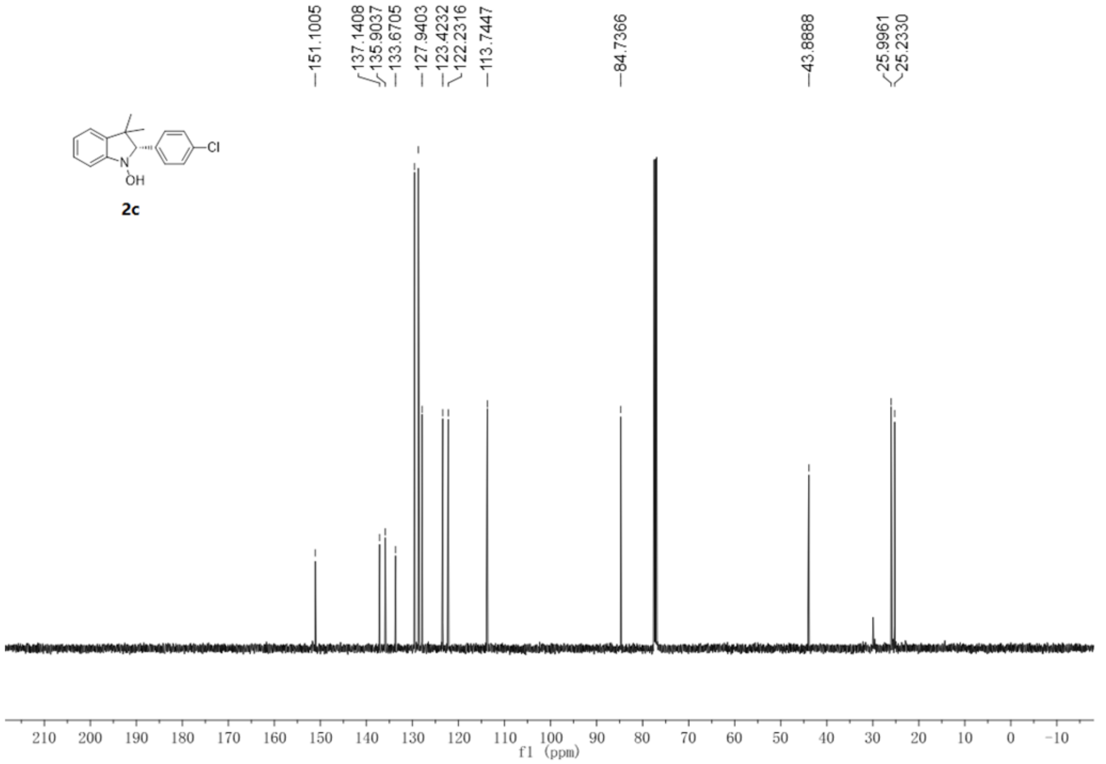

Supplementary figure 15. ^{1}H & ^{13}C NMR spectra of **2c**.

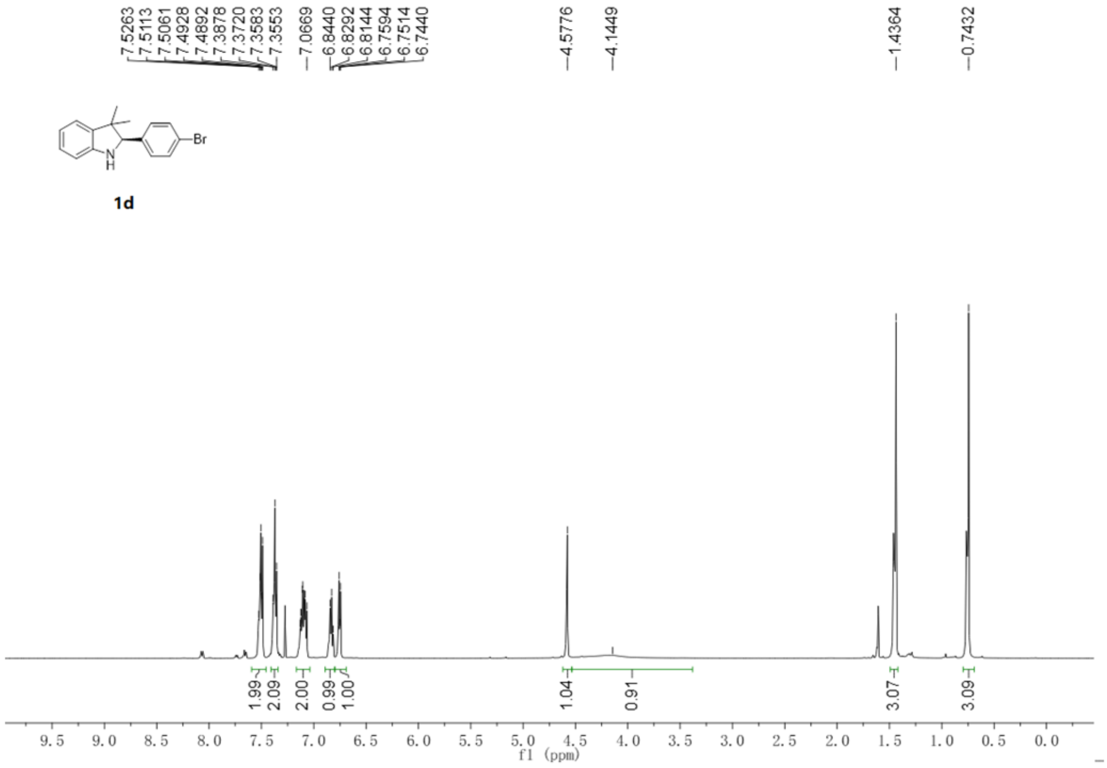

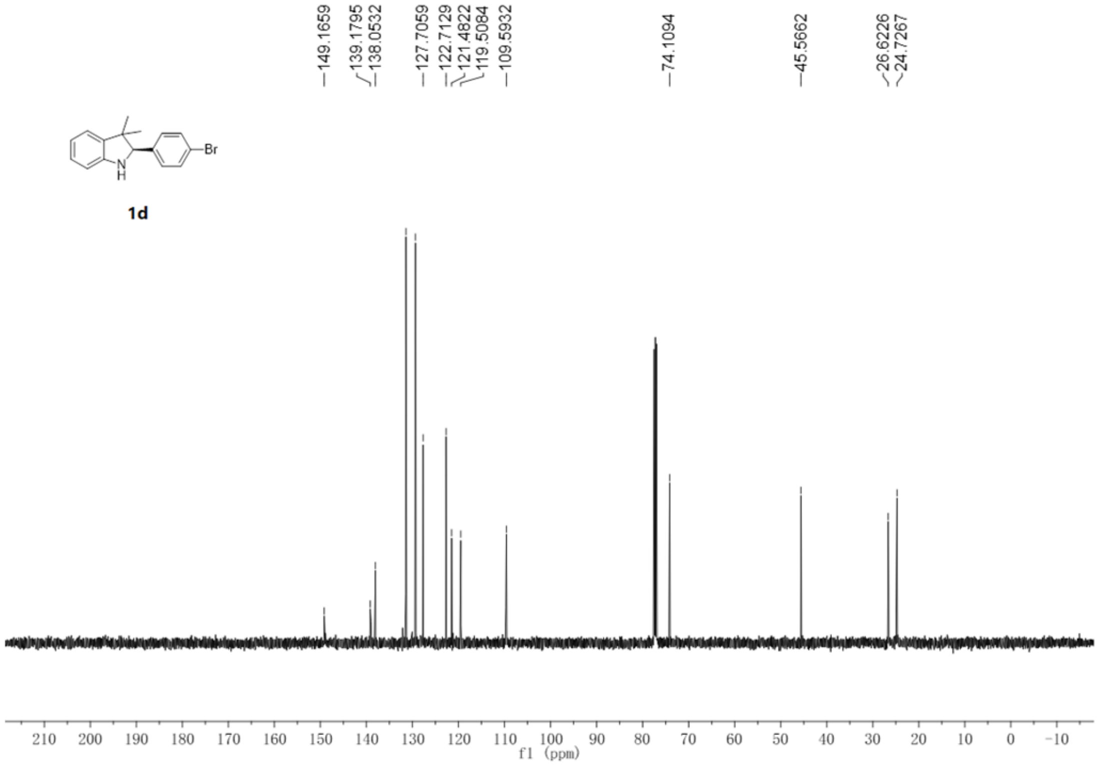

Supplementary figure 16. ^{1}H & ^{13}C NMR spectra of **1d**.

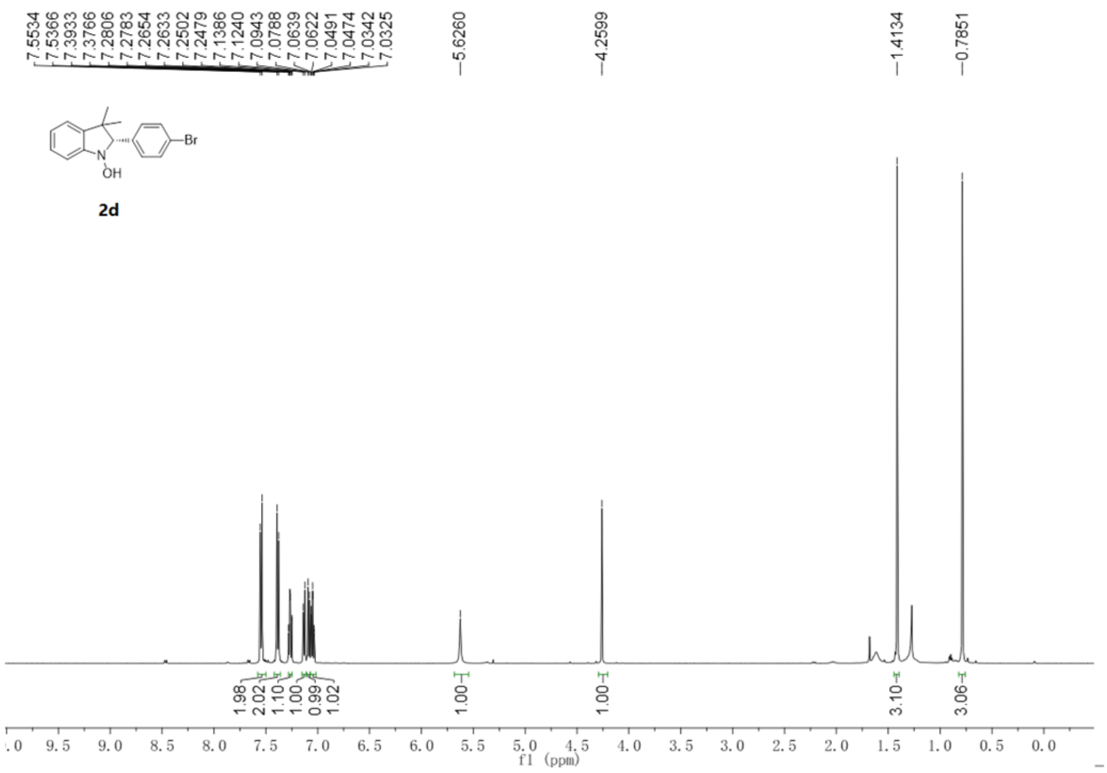

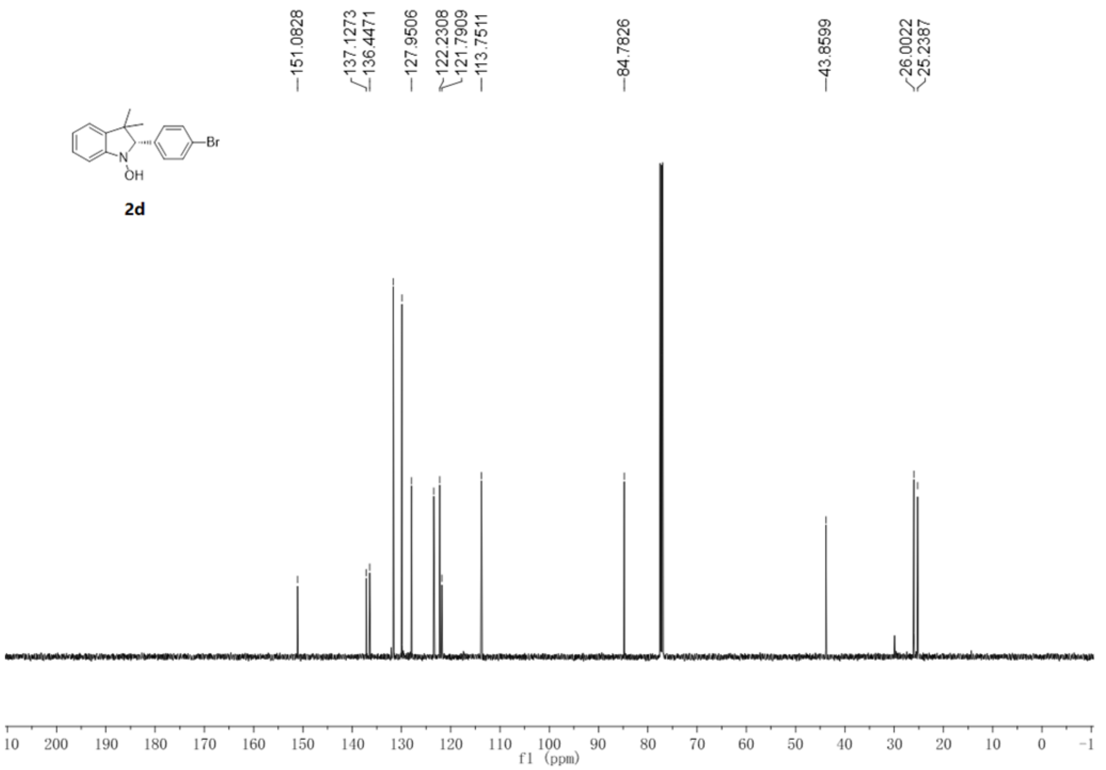

Supplementary figure 17. ^{1}H & ^{13}C NMR spectra of **2d**.

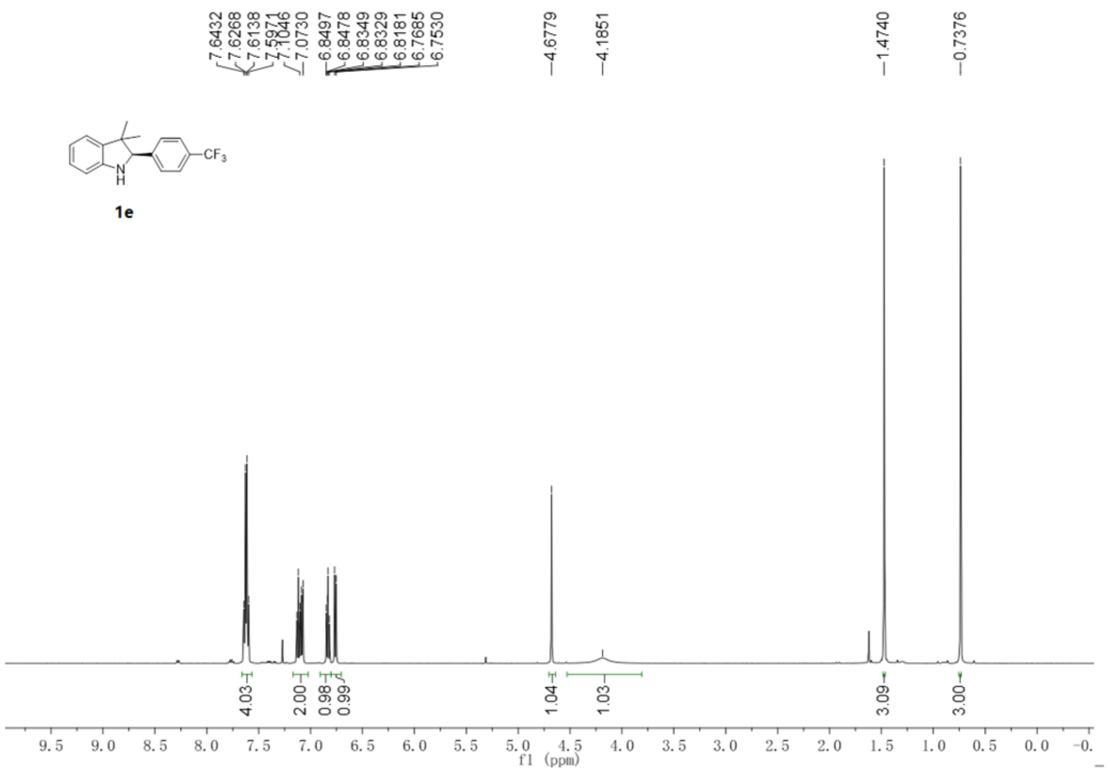

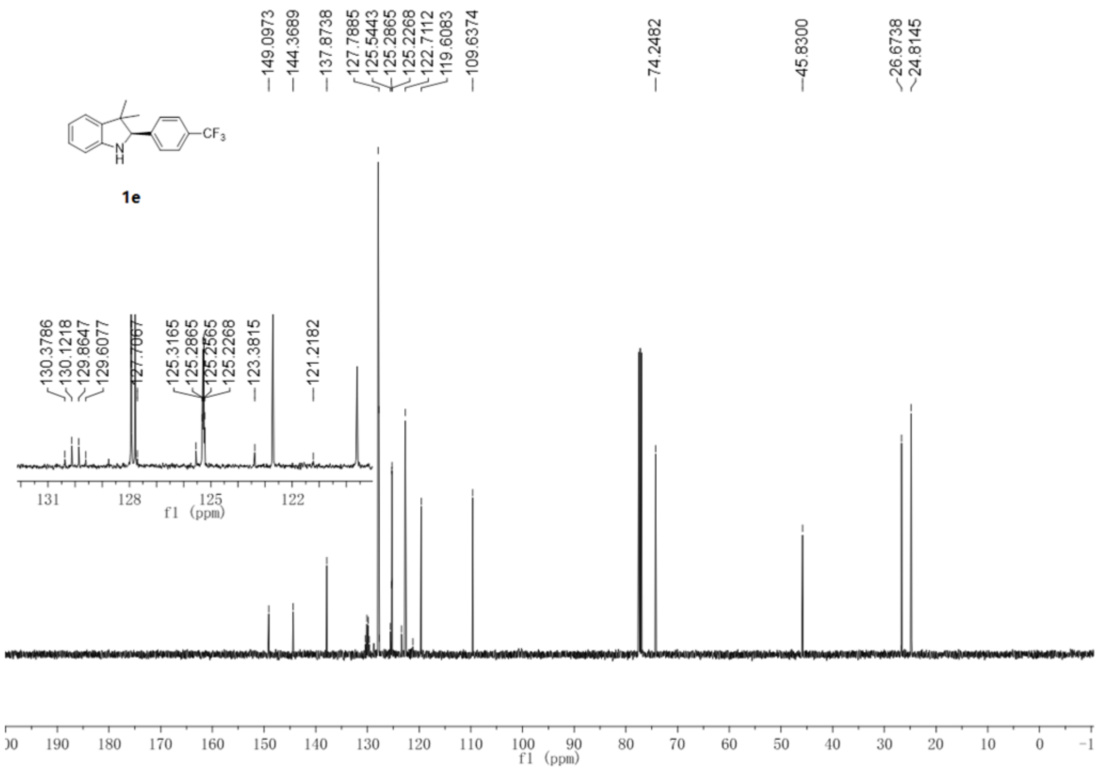

Supplementary figure 18. ^{1}H & ^{13}C NMR spectra of **1e**.

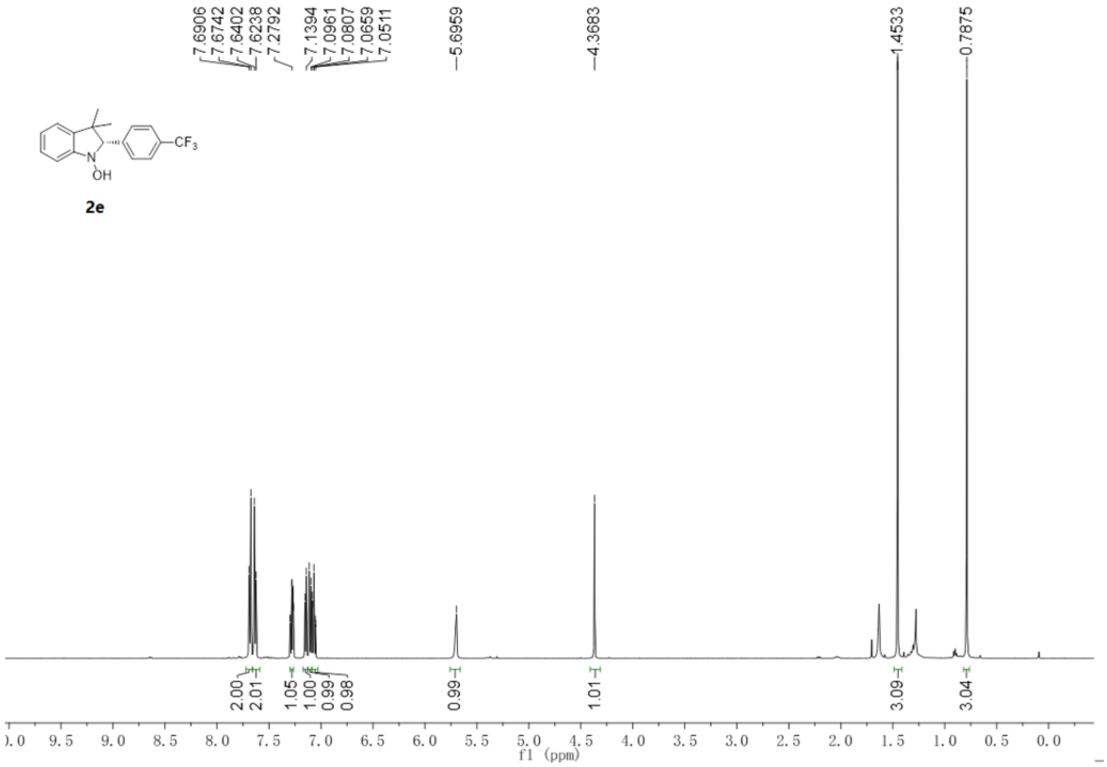

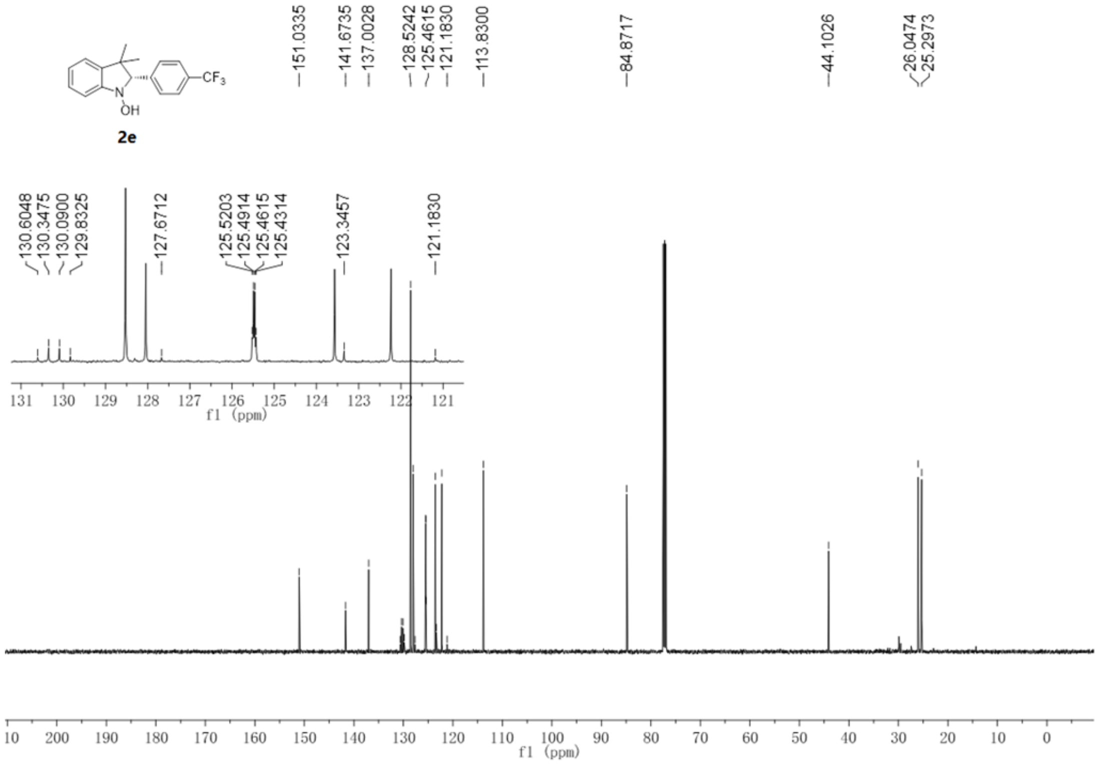

Supplementary figure 19. ^{1}H & ^{13}C NMR spectra of **2e**.

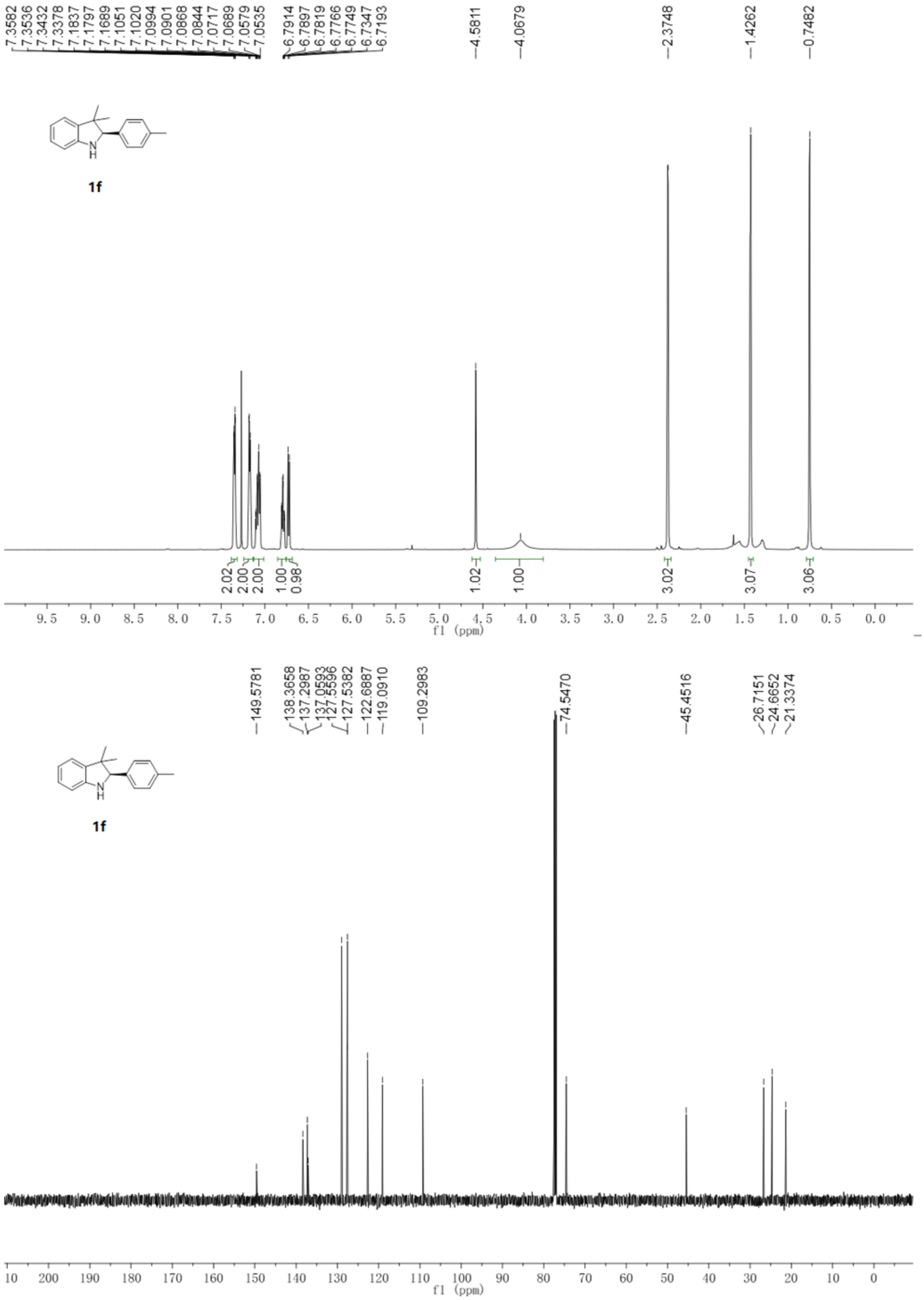

Supplementary figure 20. ^{1}H & ^{13}C NMR spectra of **1f**.

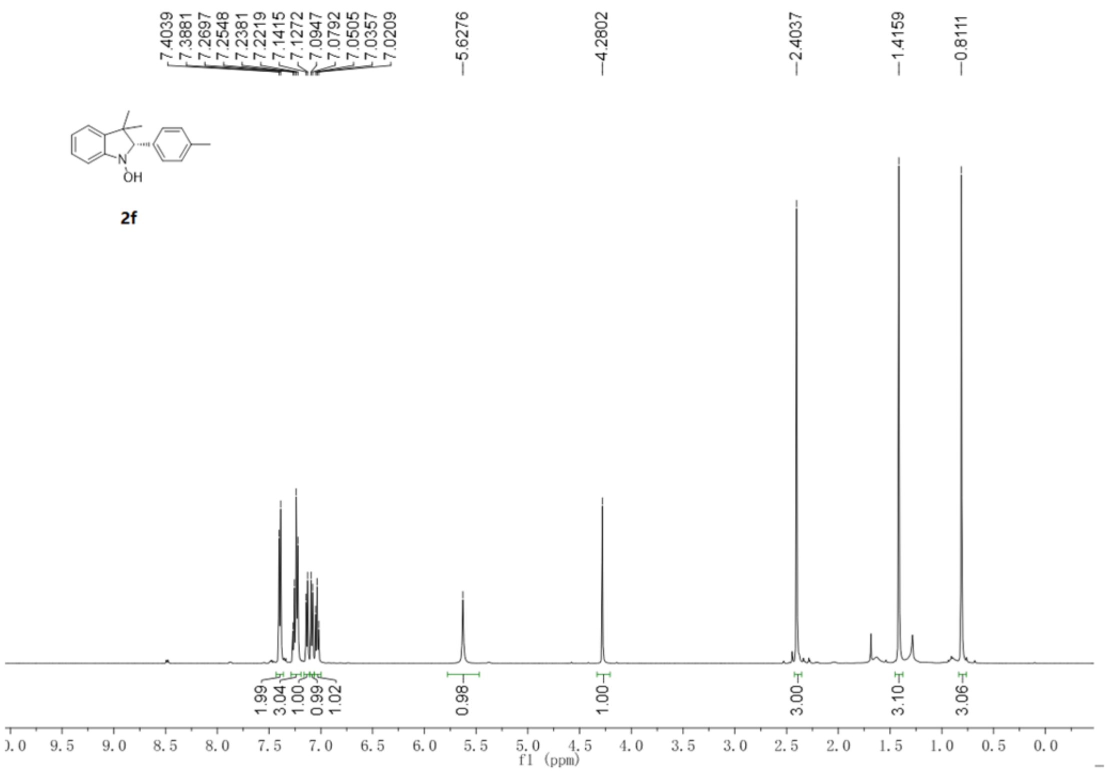

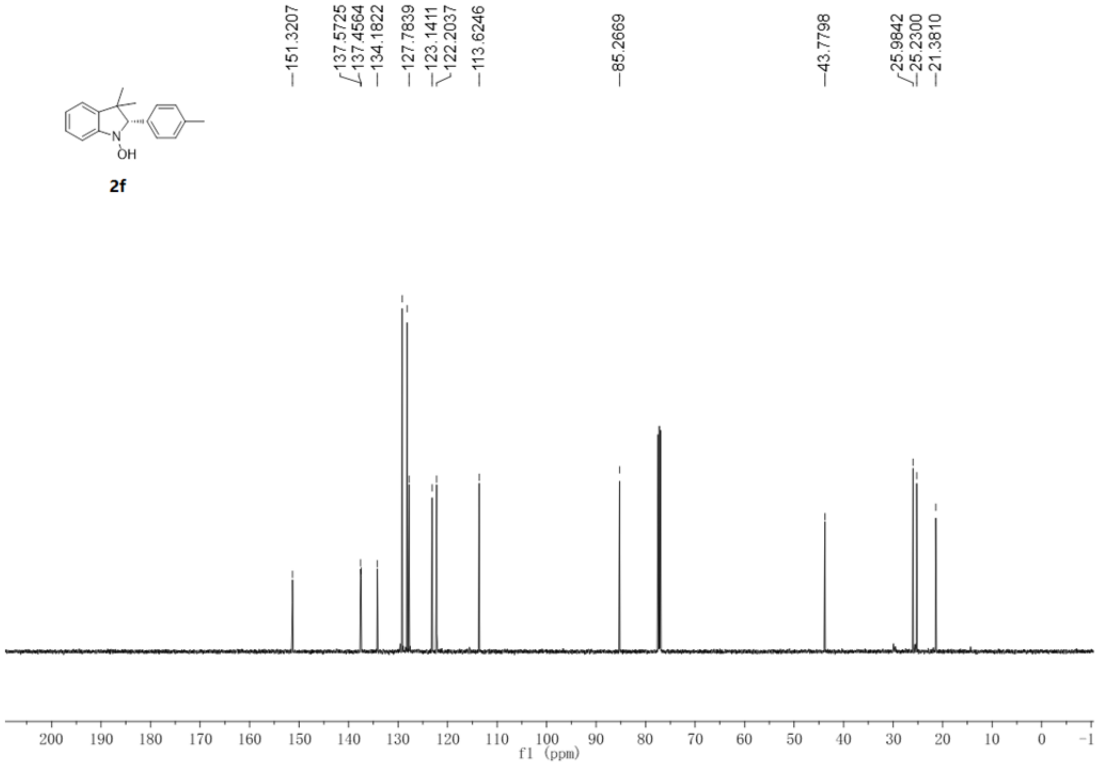

Supplementary figure 21. ^{1}H & ^{13}C NMR spectra of **2f**.

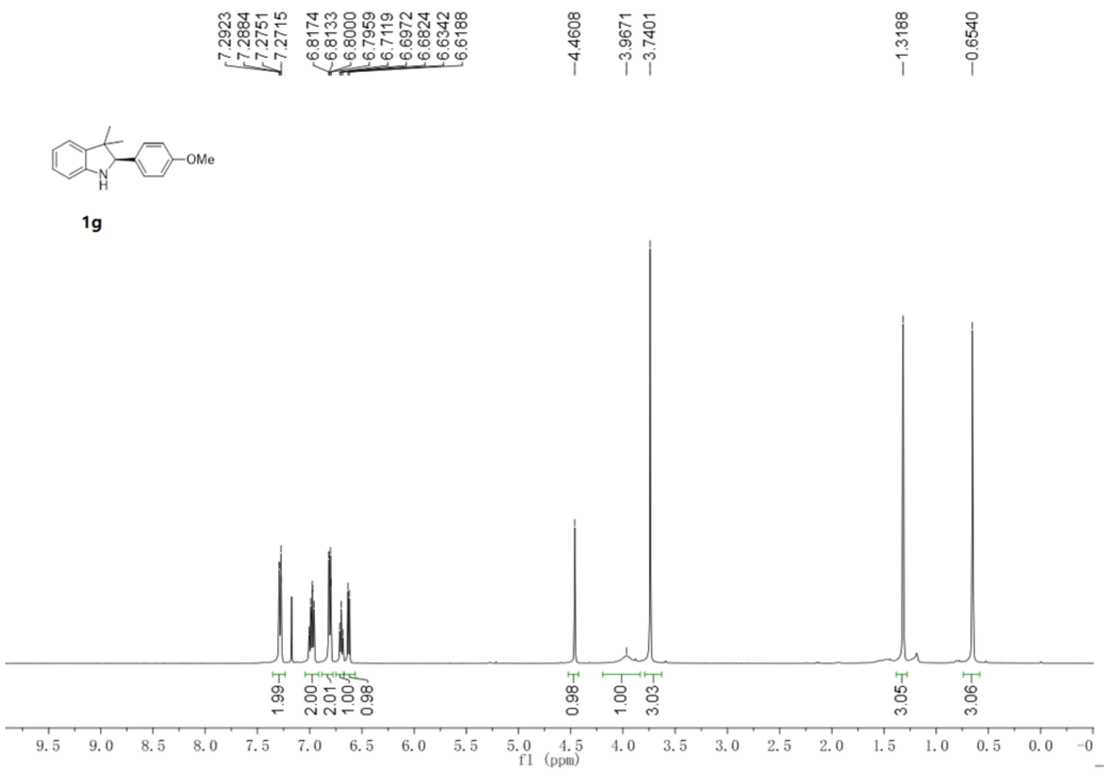

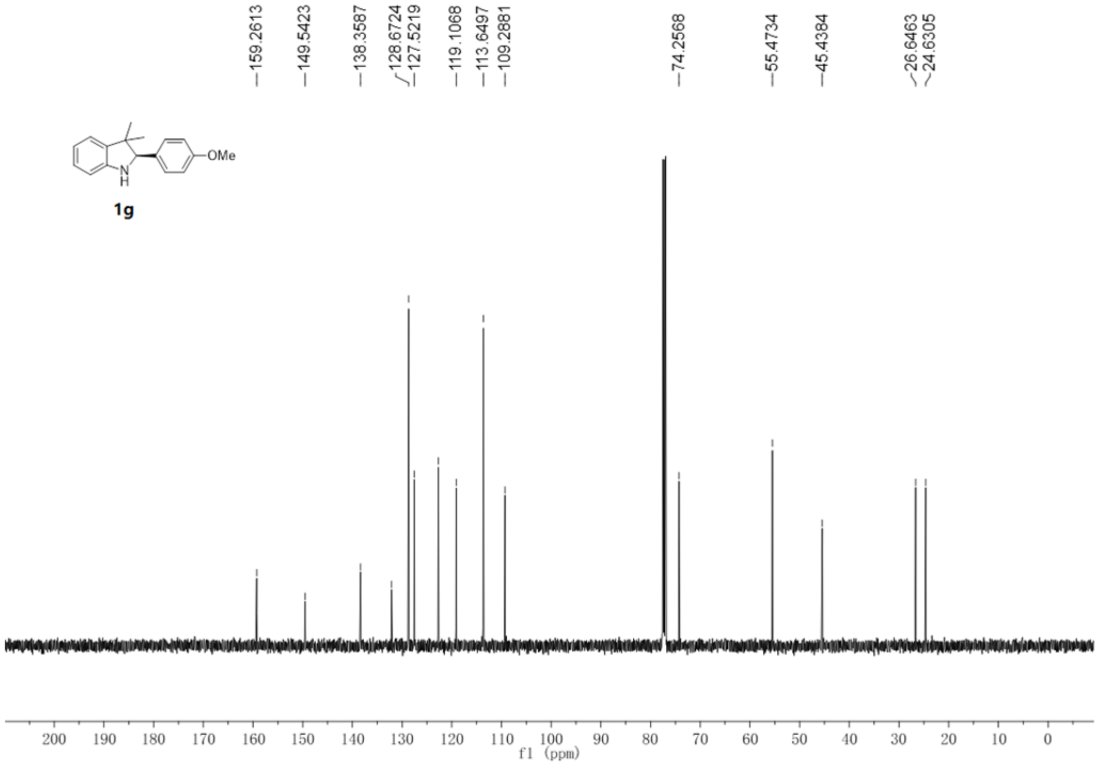

Supplementary figure 22. ^{1}H & ^{13}C NMR spectra of **1g**.

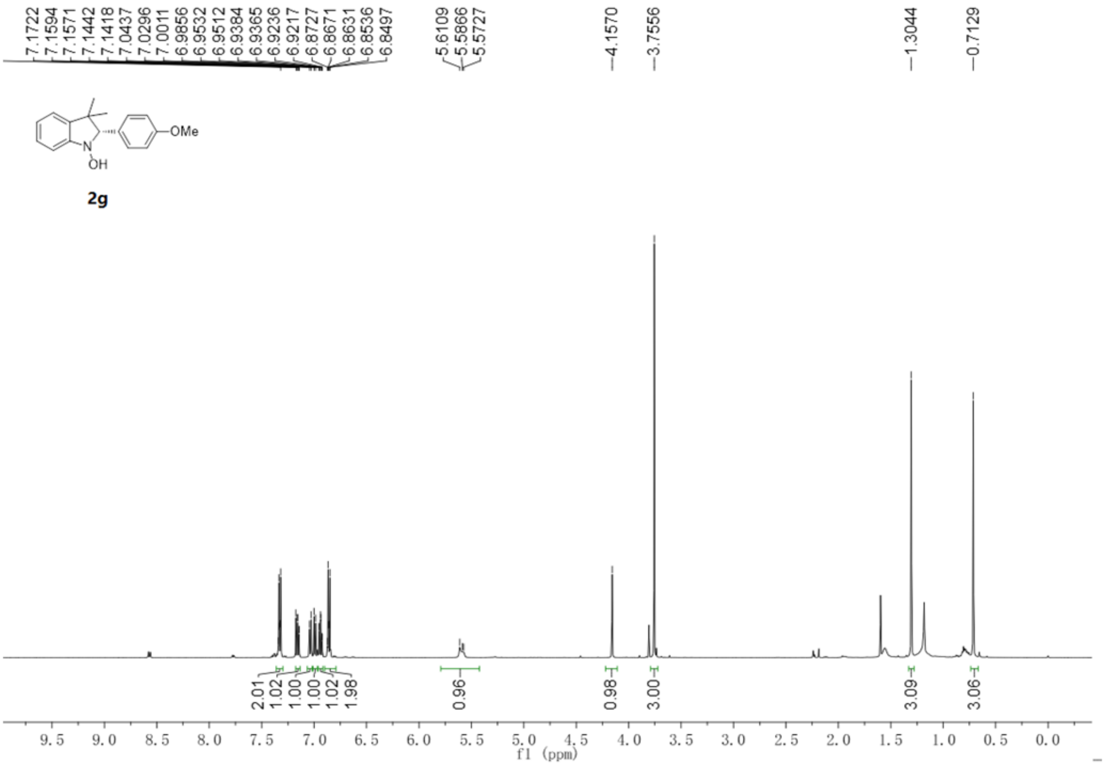

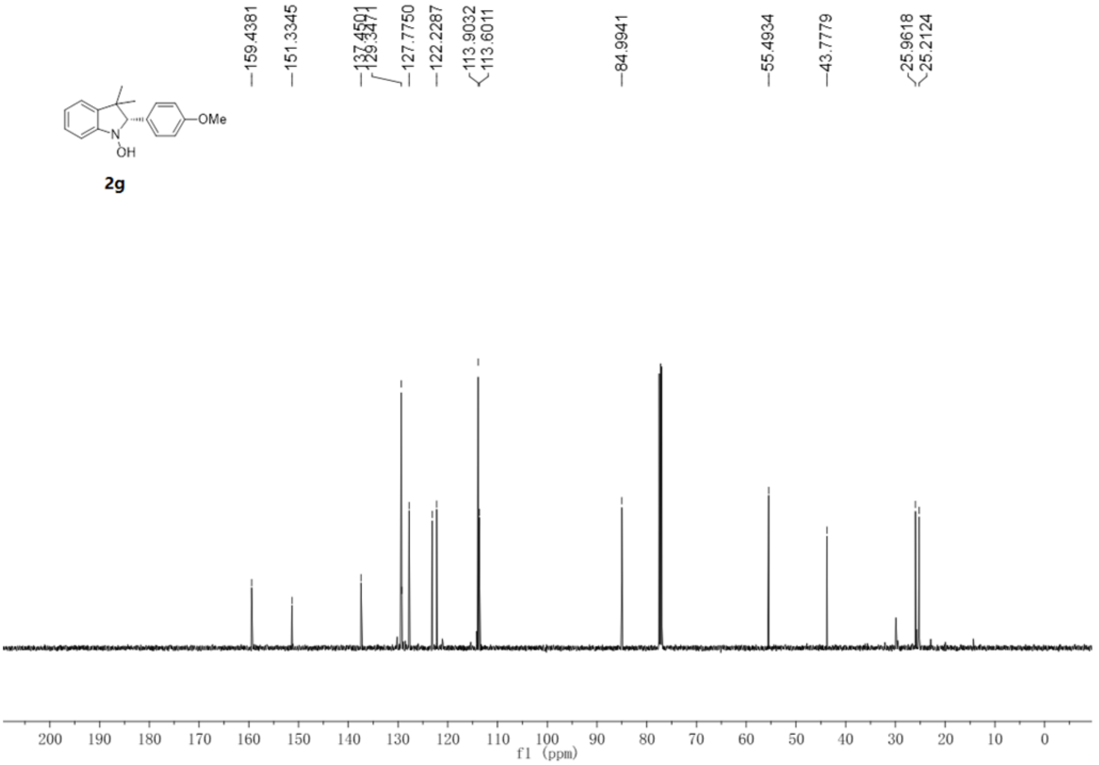

Supplementary figure 23. ^{1}H & ^{13}C NMR spectra of **2g**.

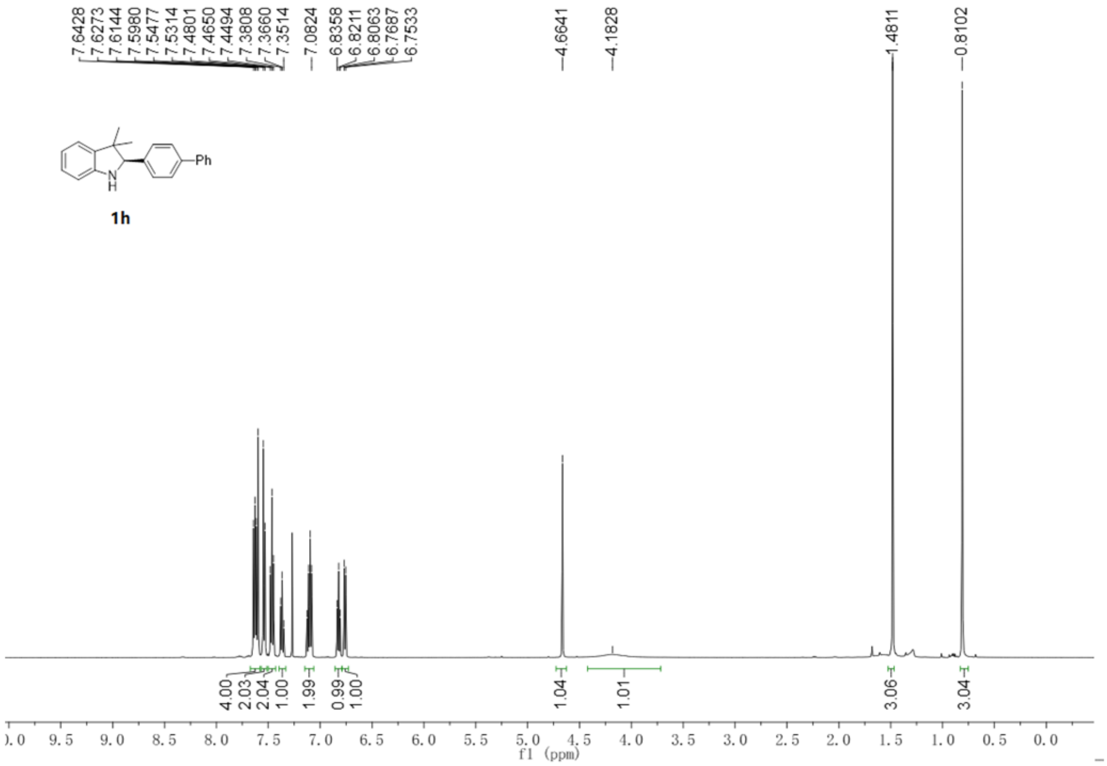

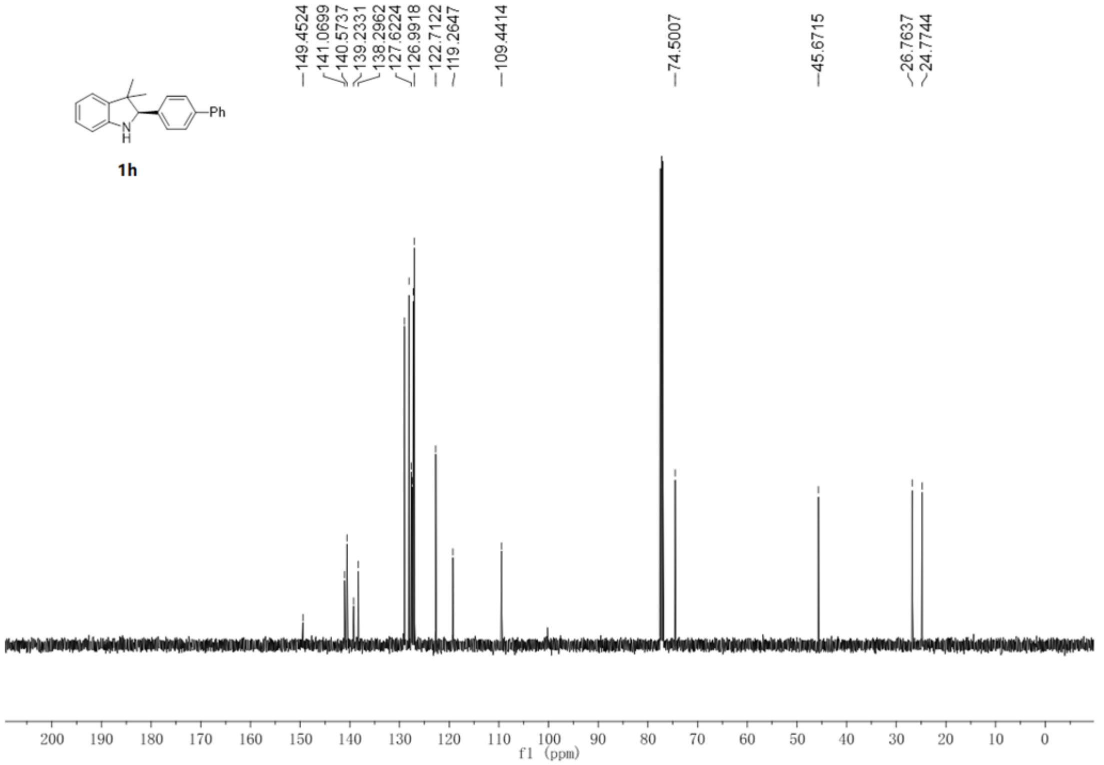

Supplementary figure 24. ^{1}H & ^{13}C NMR spectra of **1h**.

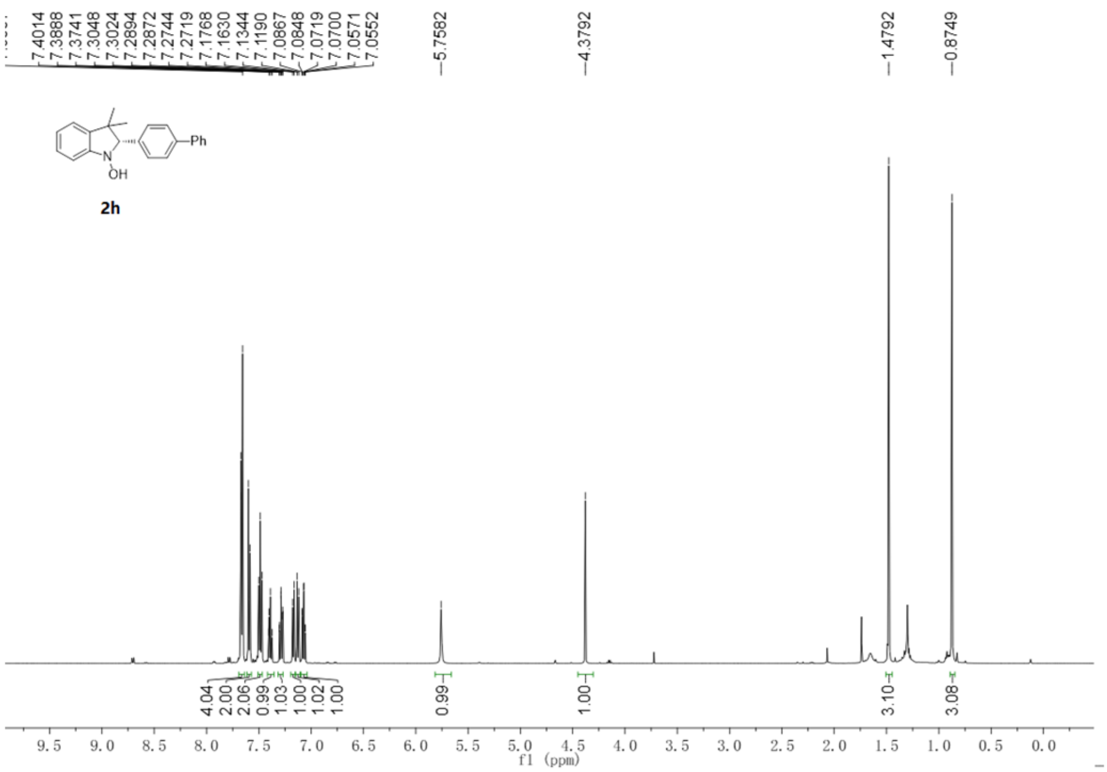

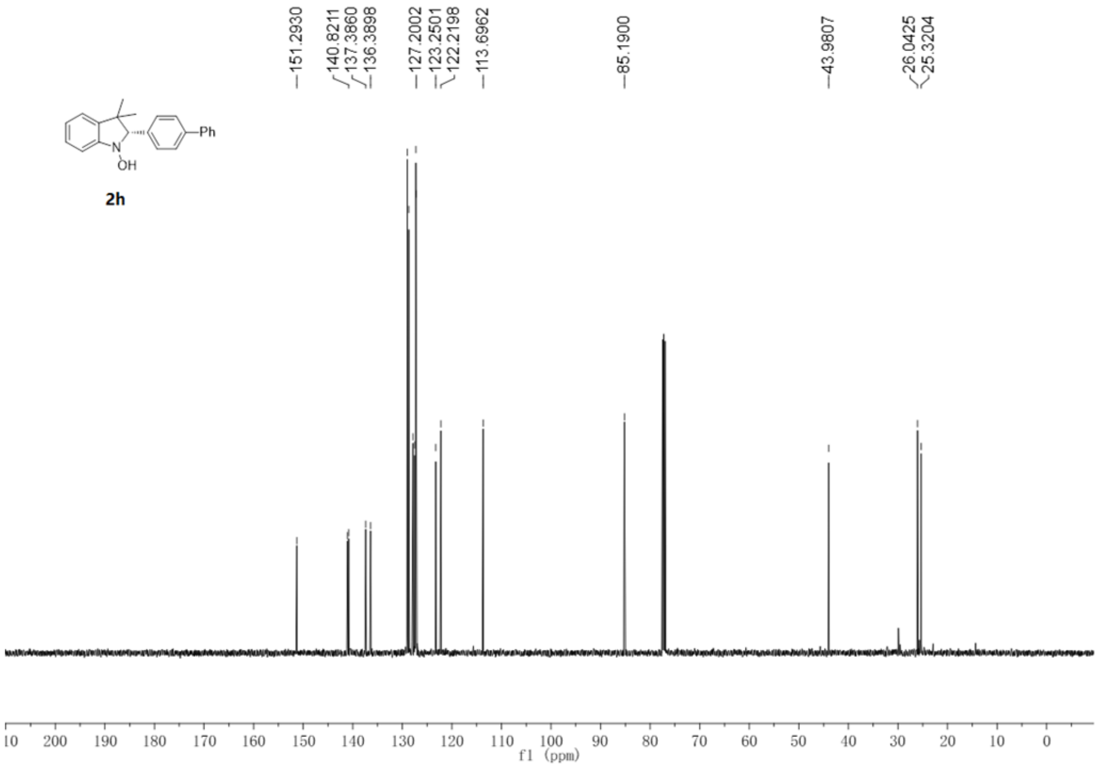

Supplementary figure 25. ^{1}H & ^{13}C NMR spectra of **2h**.

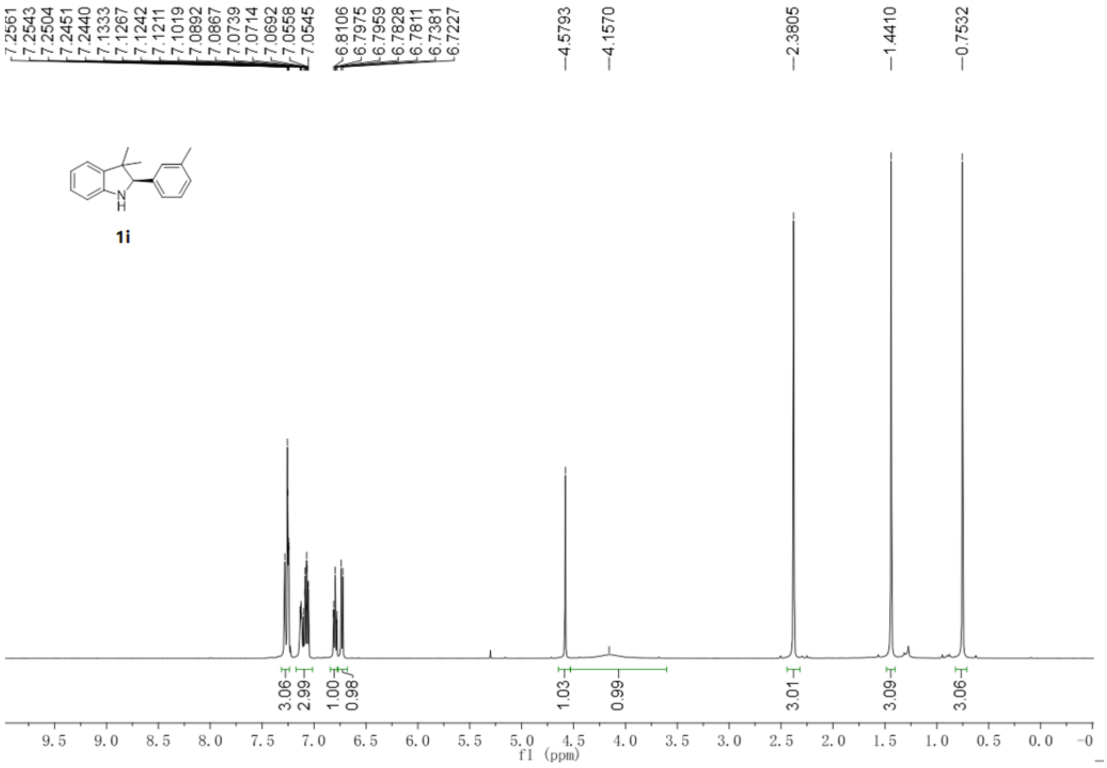

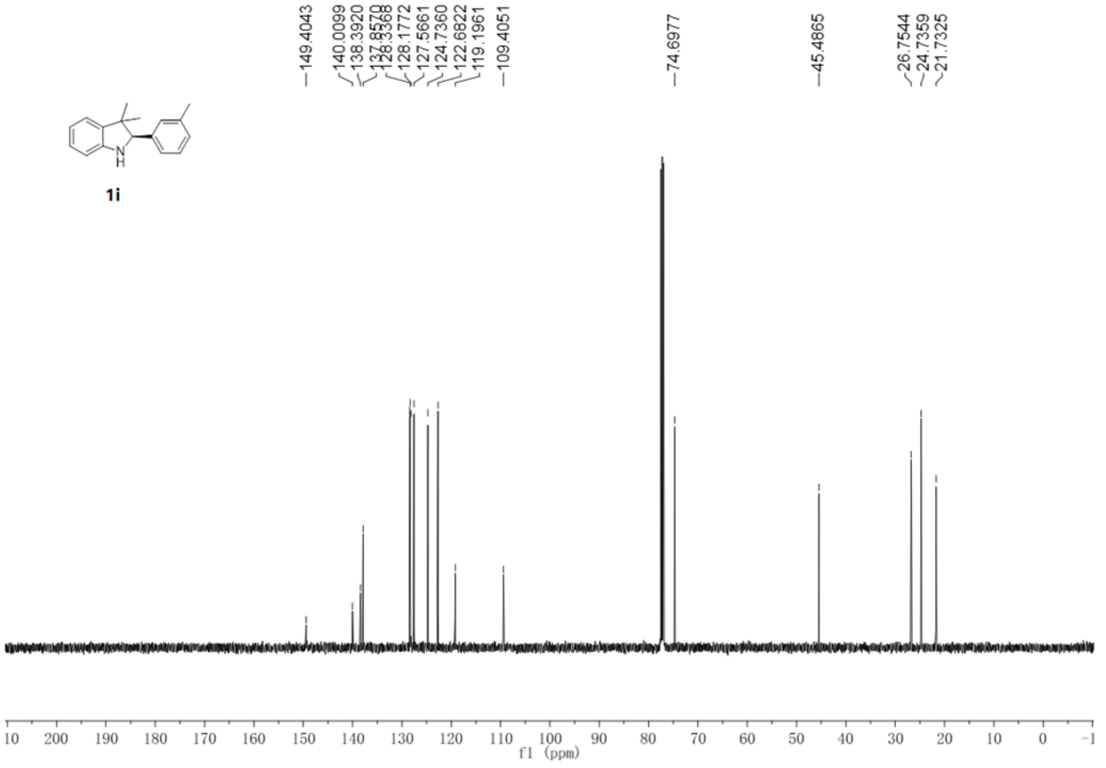

Supplementary figure 26. ^{1}H & ^{13}C NMR spectra of **1i**.

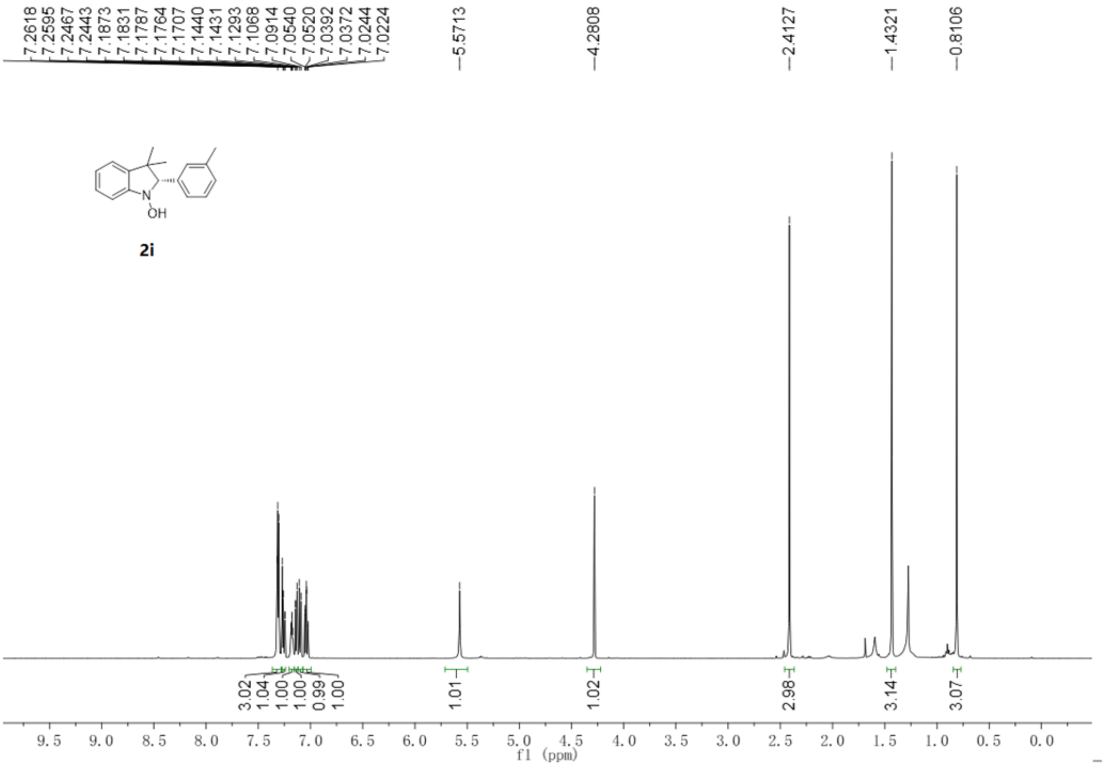

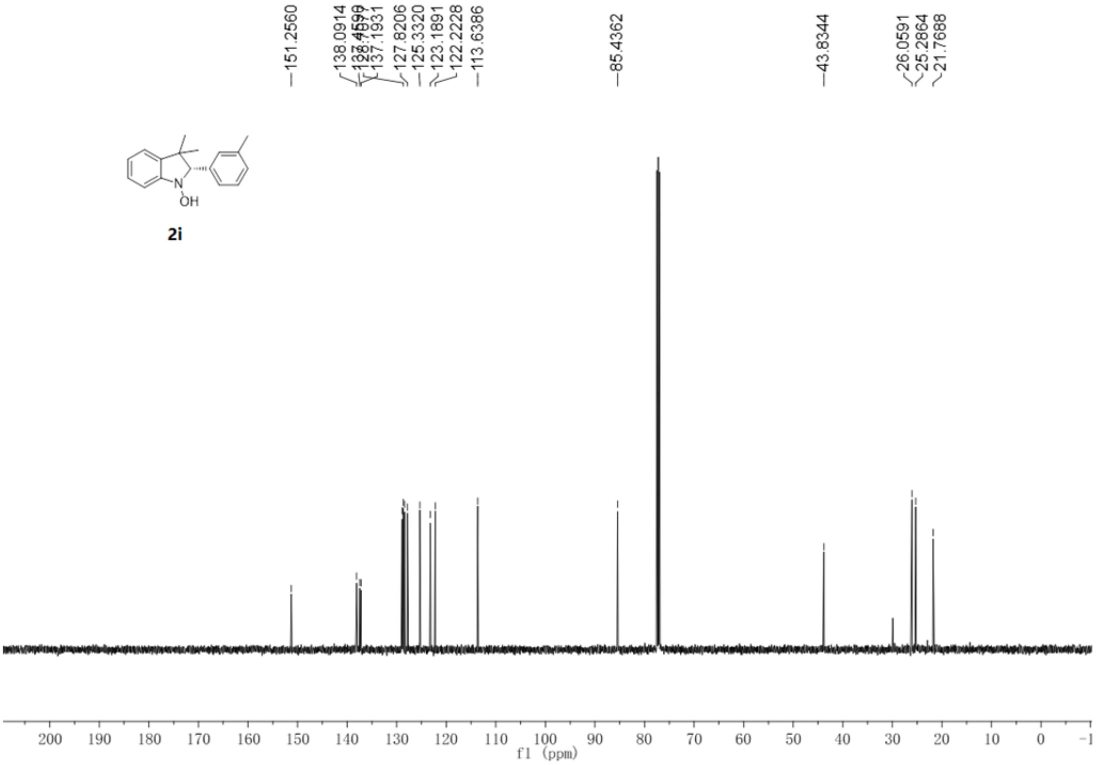

Supplementary figure 27. ^{1}H & ^{13}C NMR spectra of **2i**.

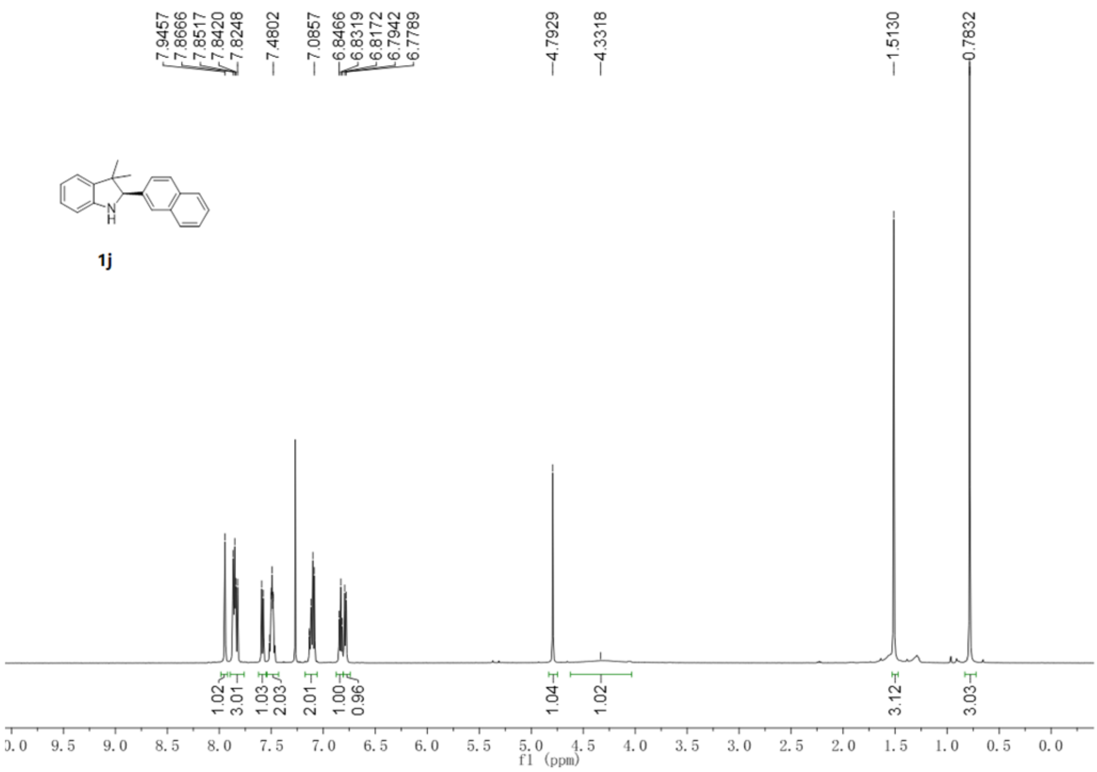

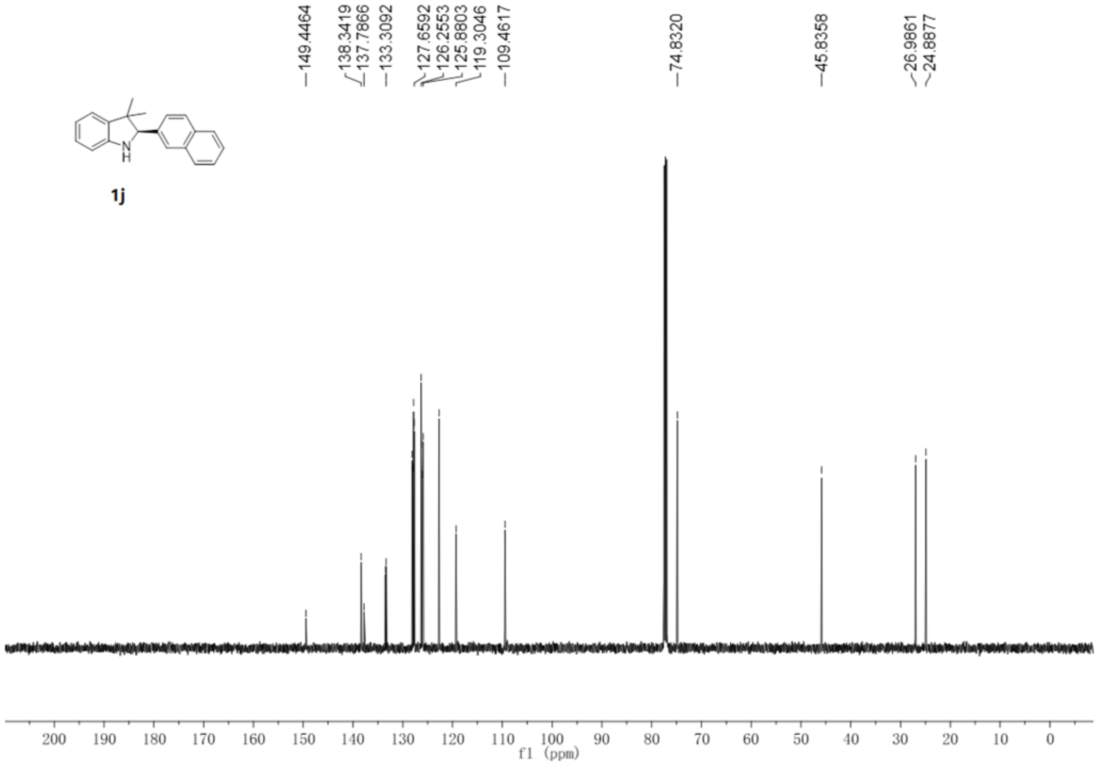

Supplementary figure 28. ^{1}H & ^{13}C NMR spectra of **1j**.

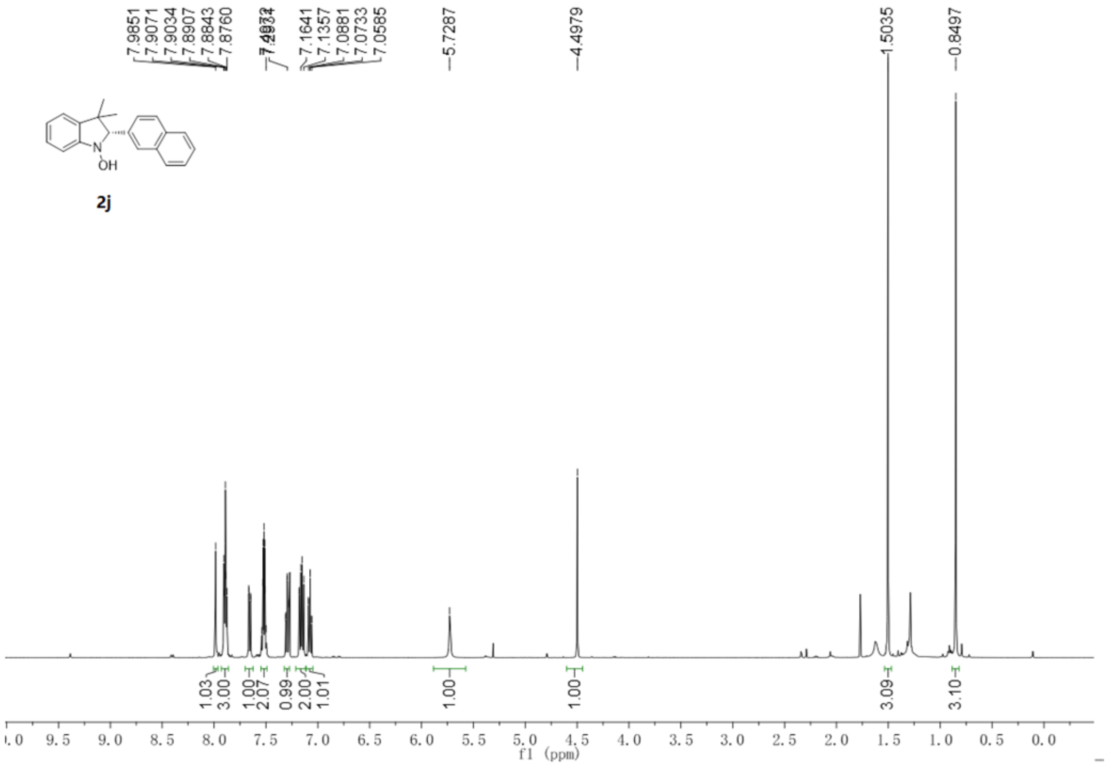

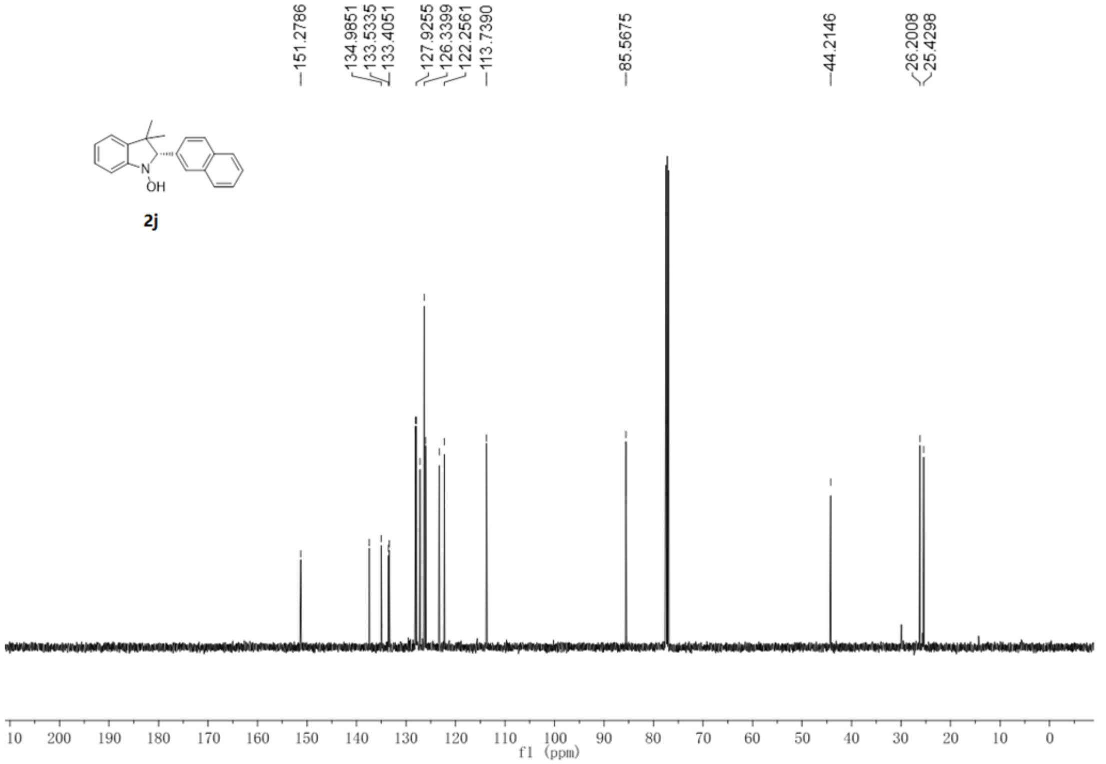

Supplementary figure 29. ^{1}H & ^{13}C NMR spectra of **2j**.

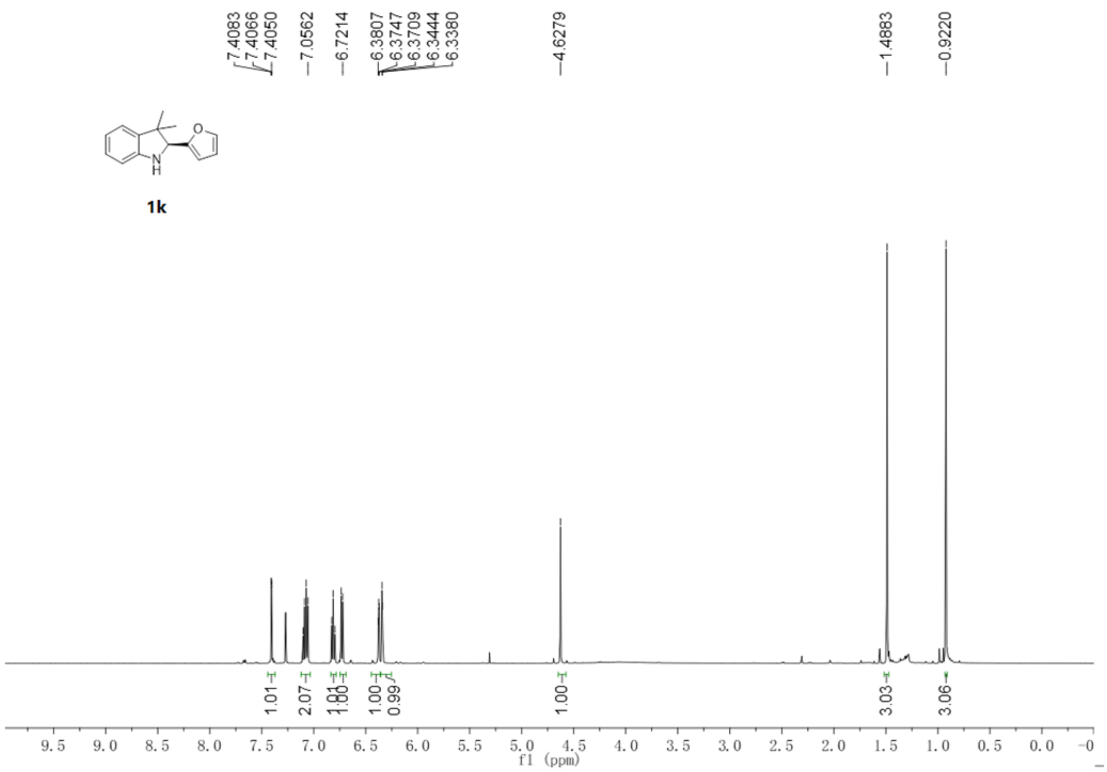

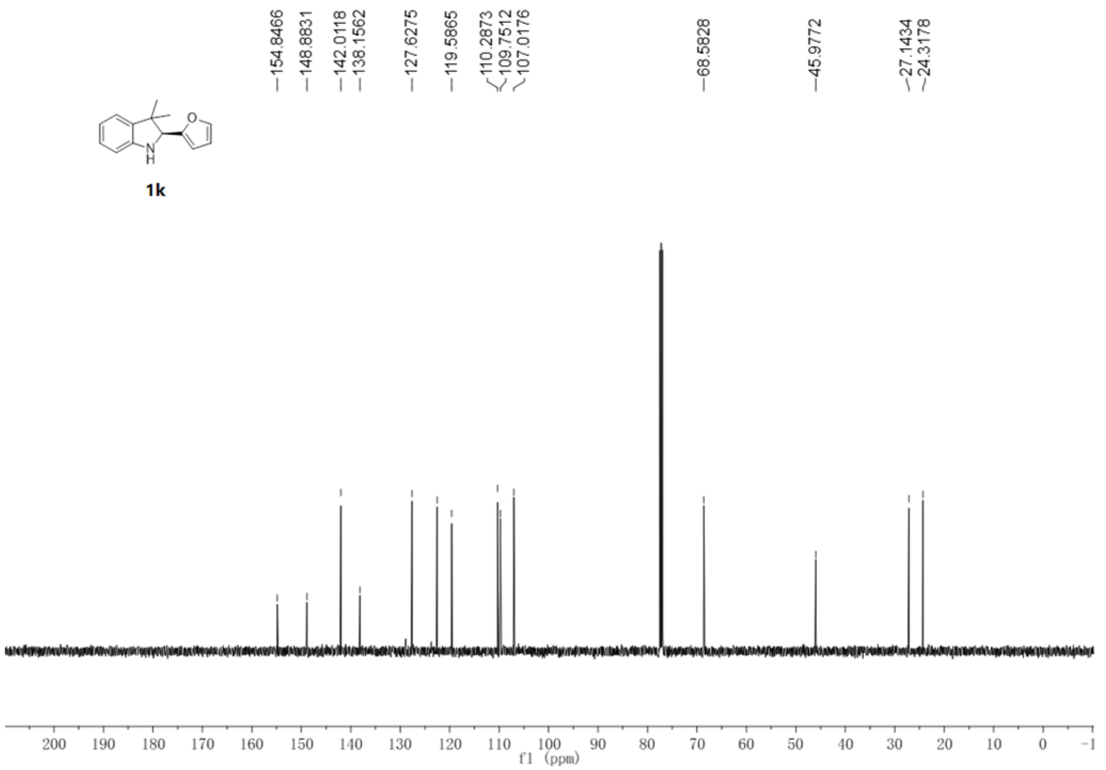

Supplementary figure 30. ^{1}H & ^{13}C NMR spectra of **1k**.

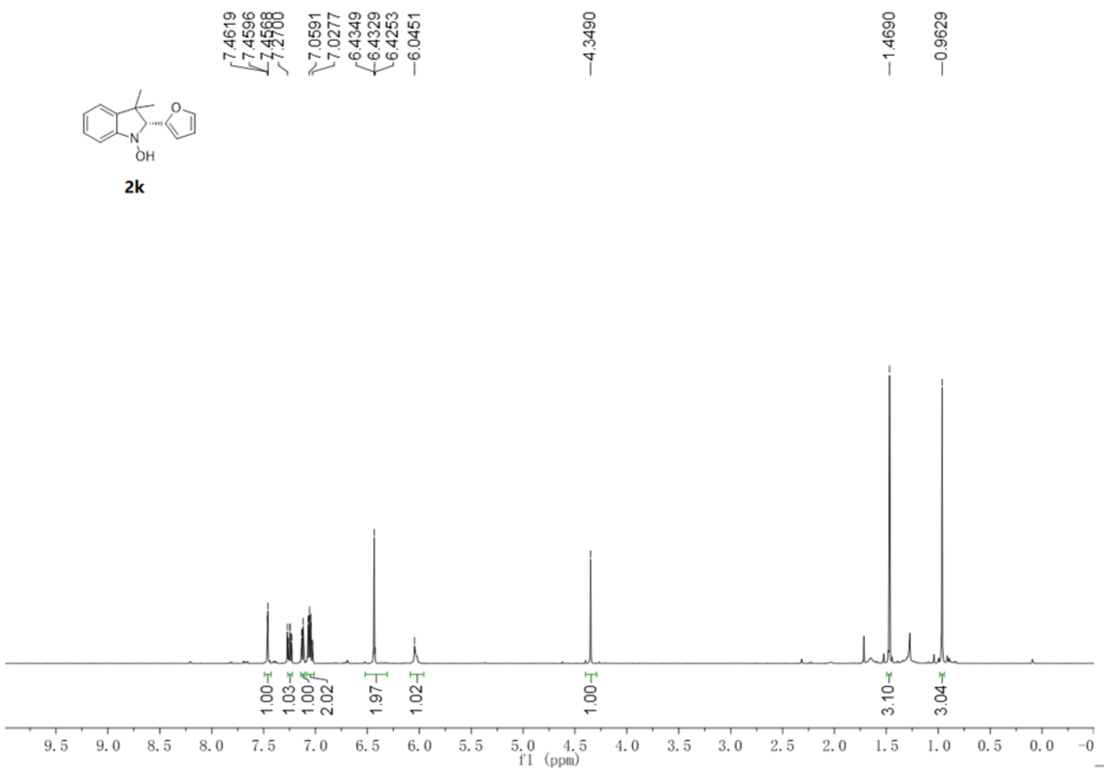

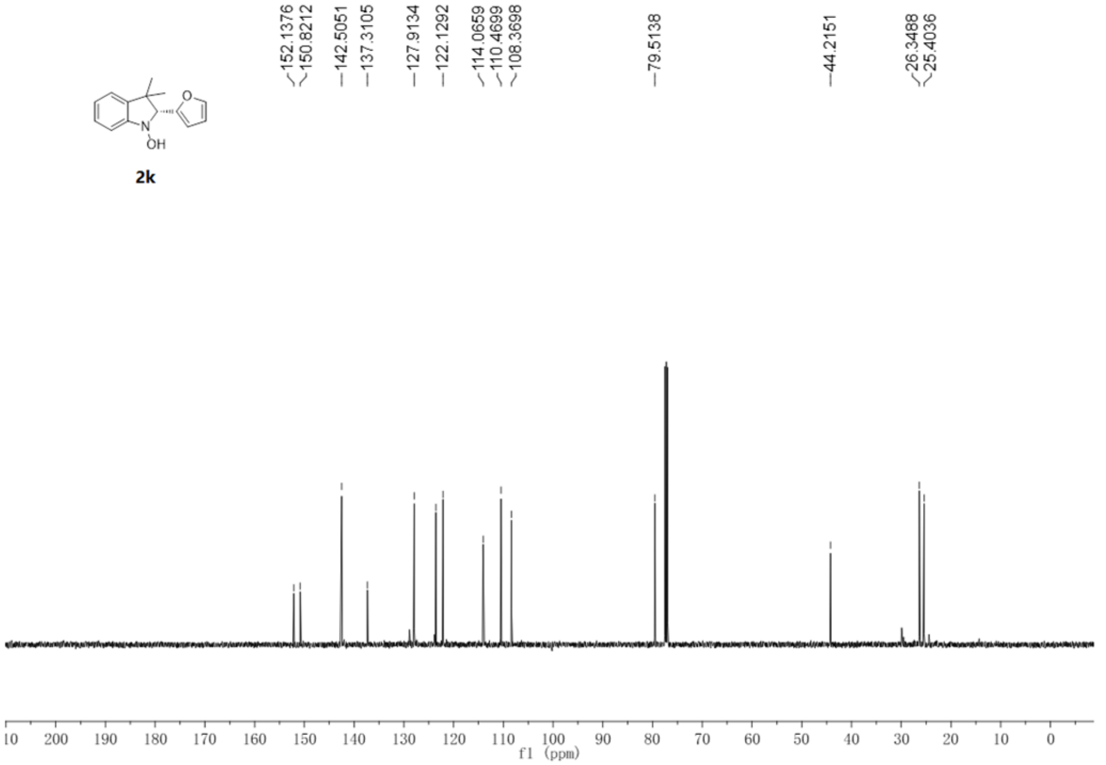

Supplementary figure 31. ^{1}H & ^{13}C NMR spectra of **2k**.

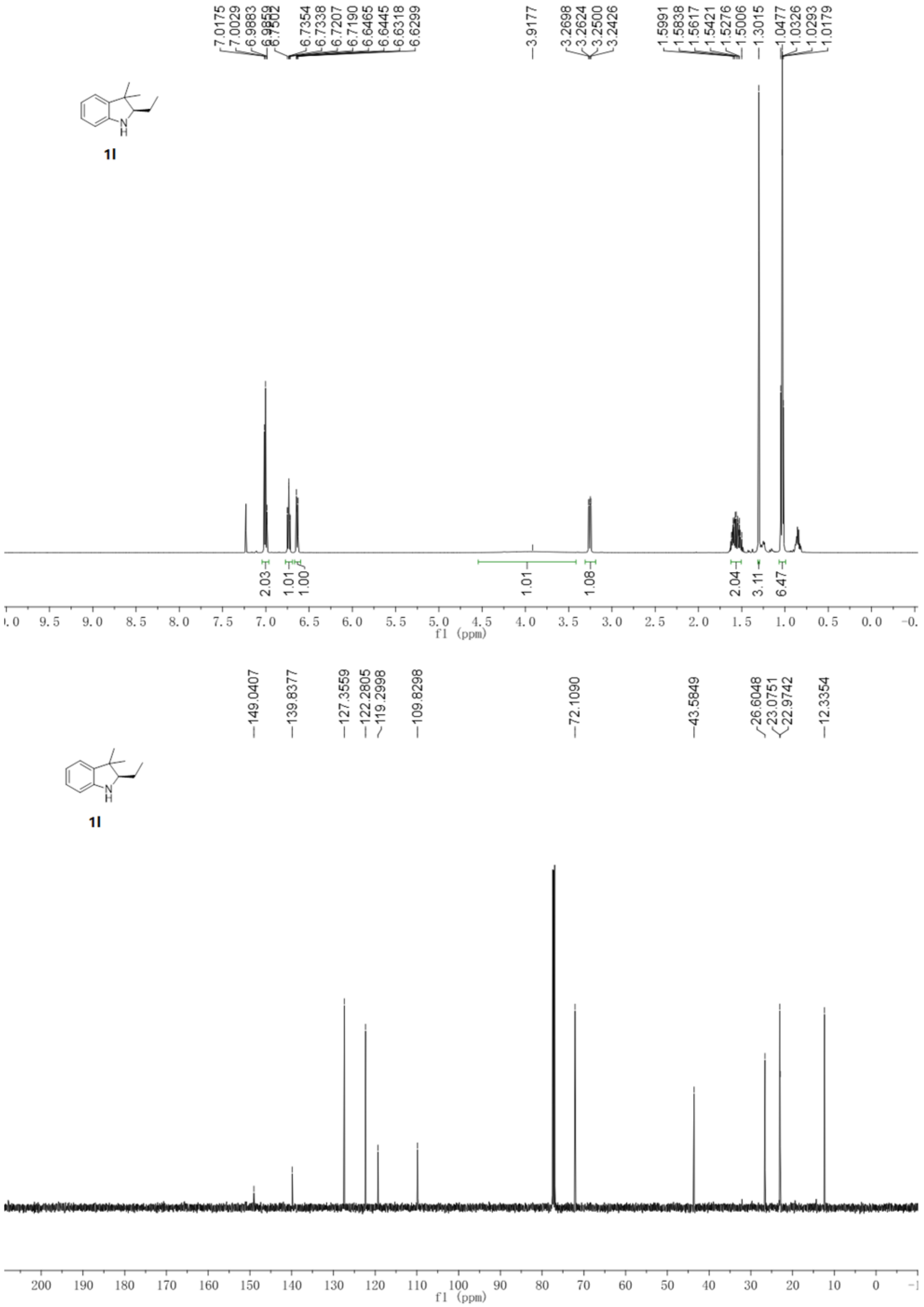

Supplementary figure 32. ^{1}H & ^{13}C NMR spectra of **1l**.

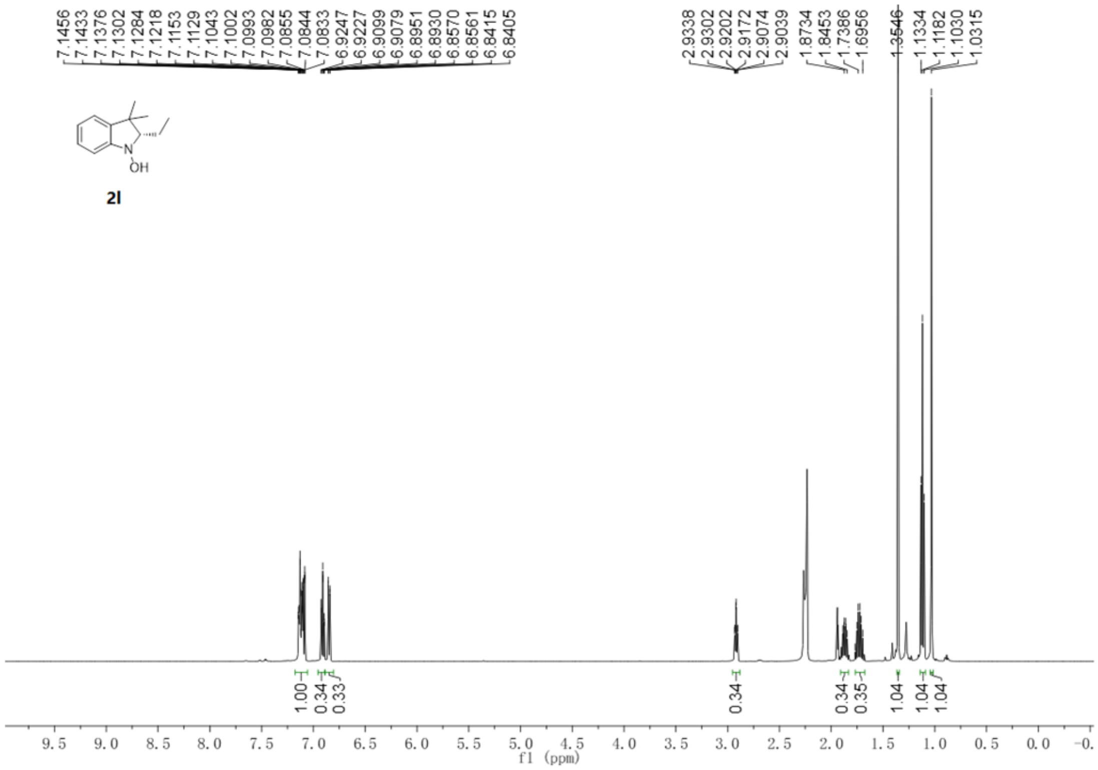

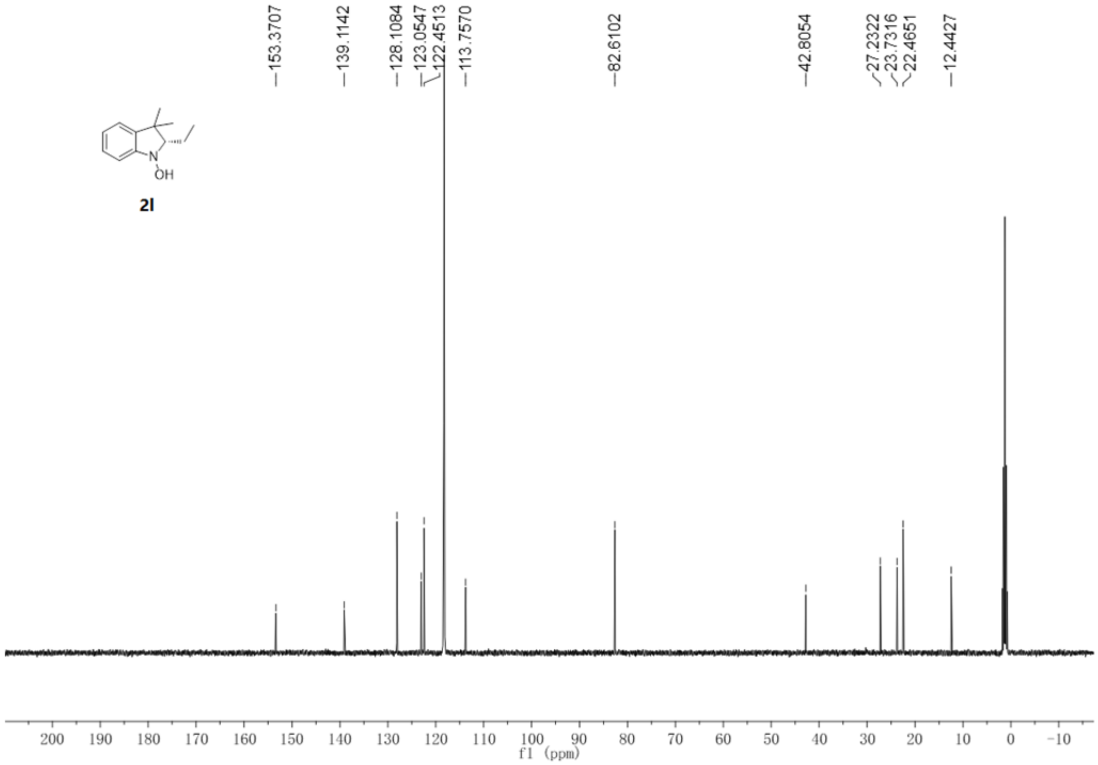

Supplementary figure 33. ^{1}H & ^{13}C NMR spectra of **2l**.

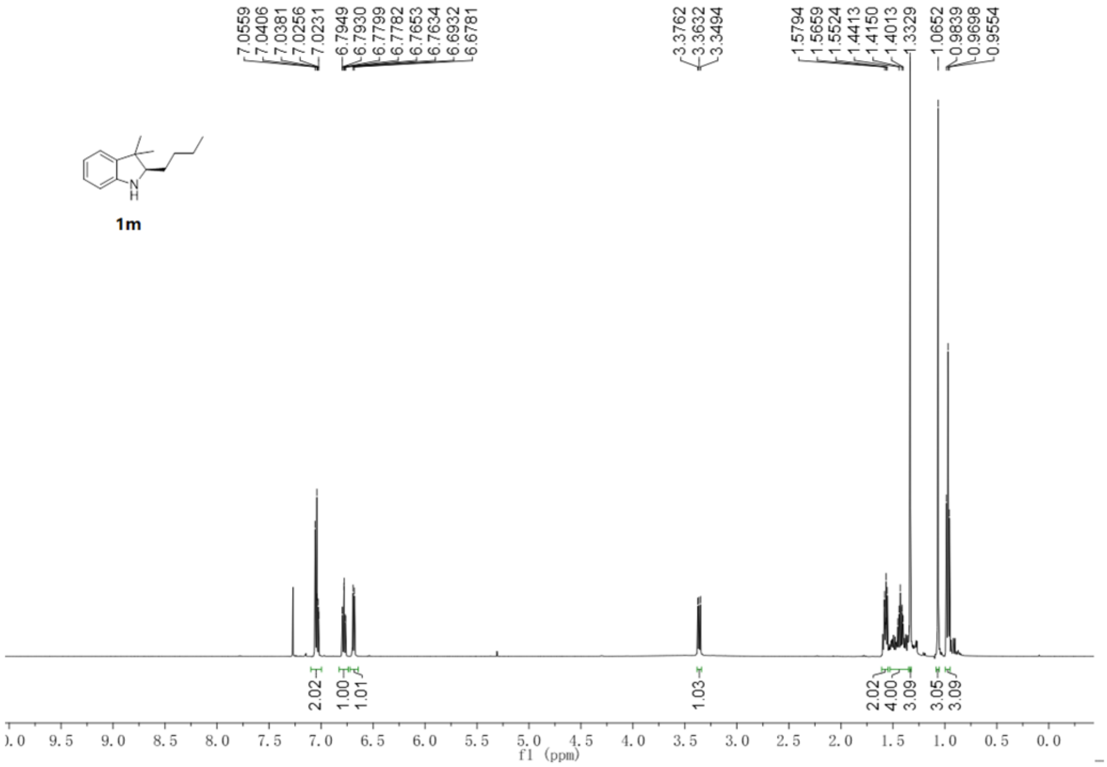

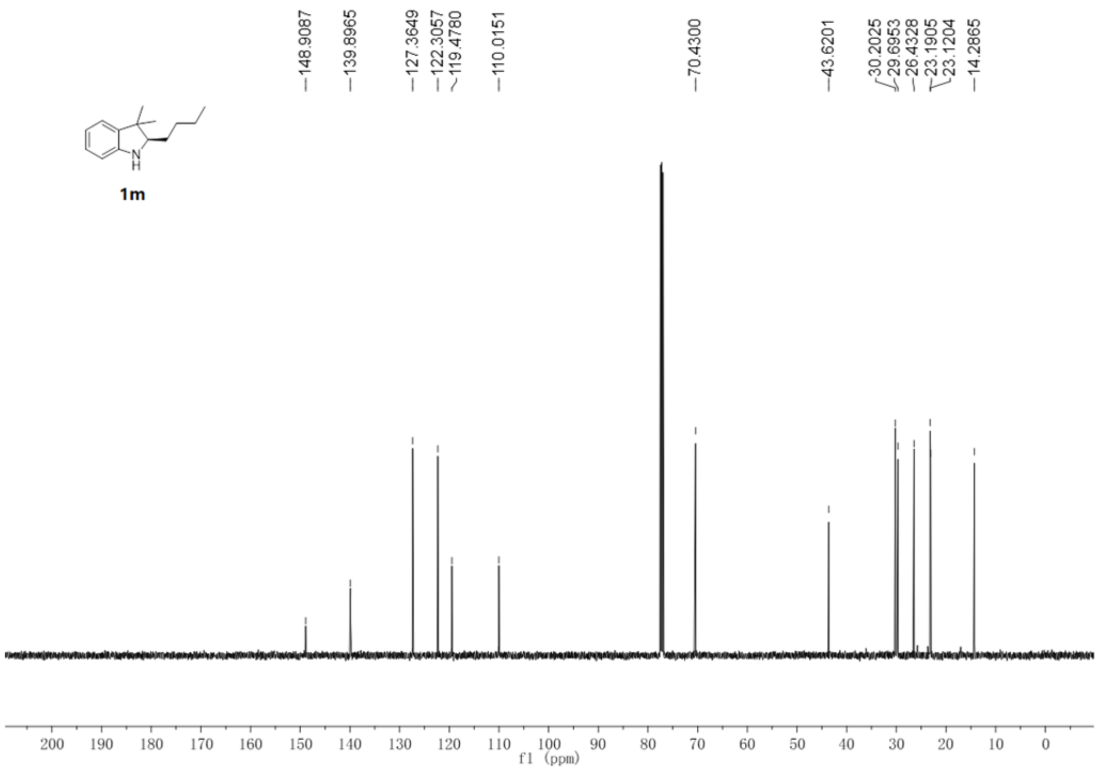

Supplementary figure 34. ^{1}H & ^{13}C NMR spectra of **1m**.

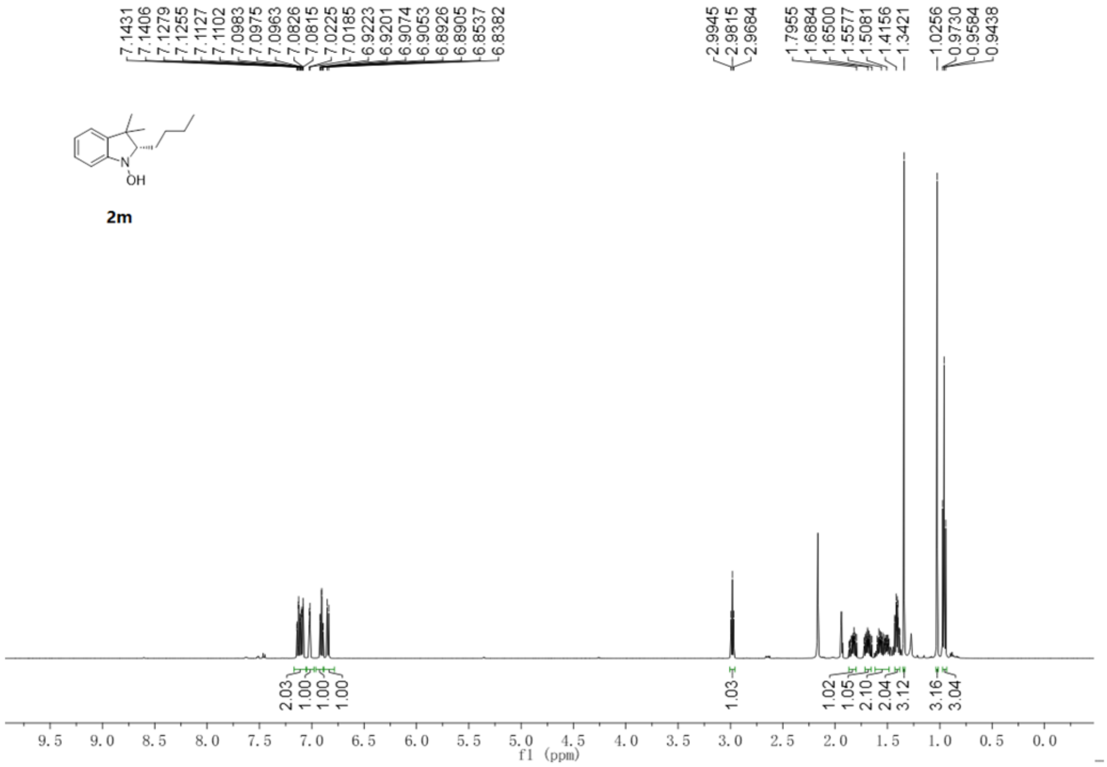

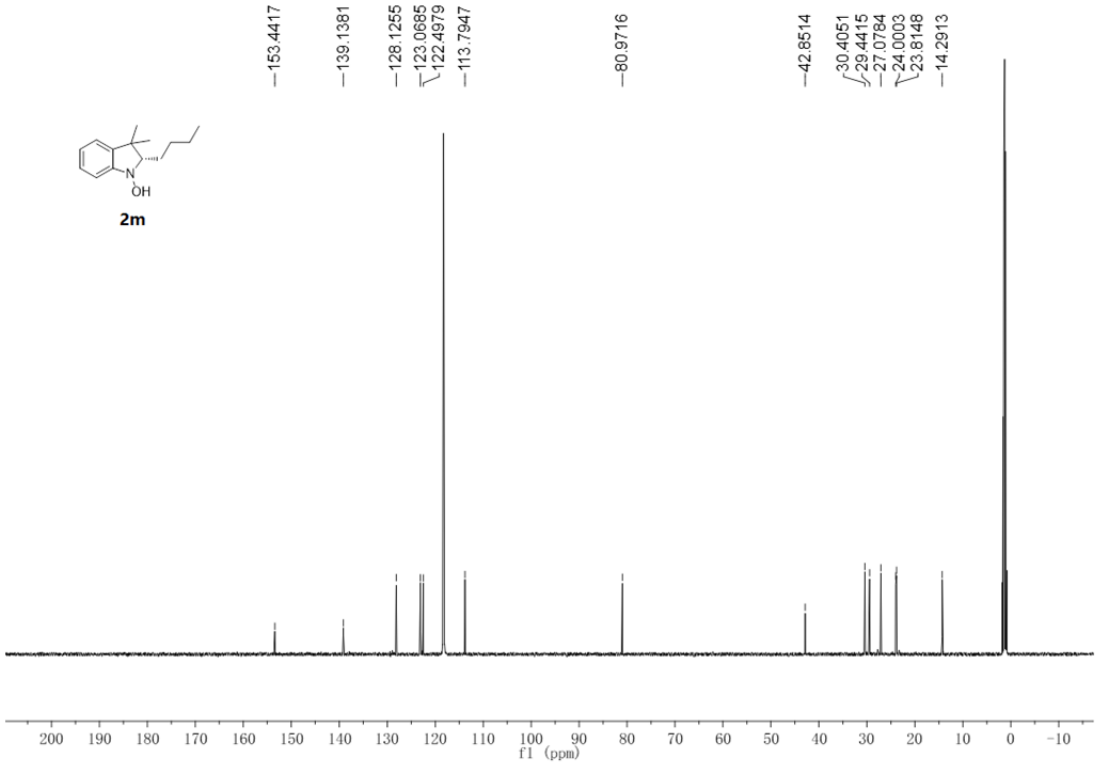

Supplementary figure 35. ^{1}H & ^{13}C NMR spectra of **2m**.

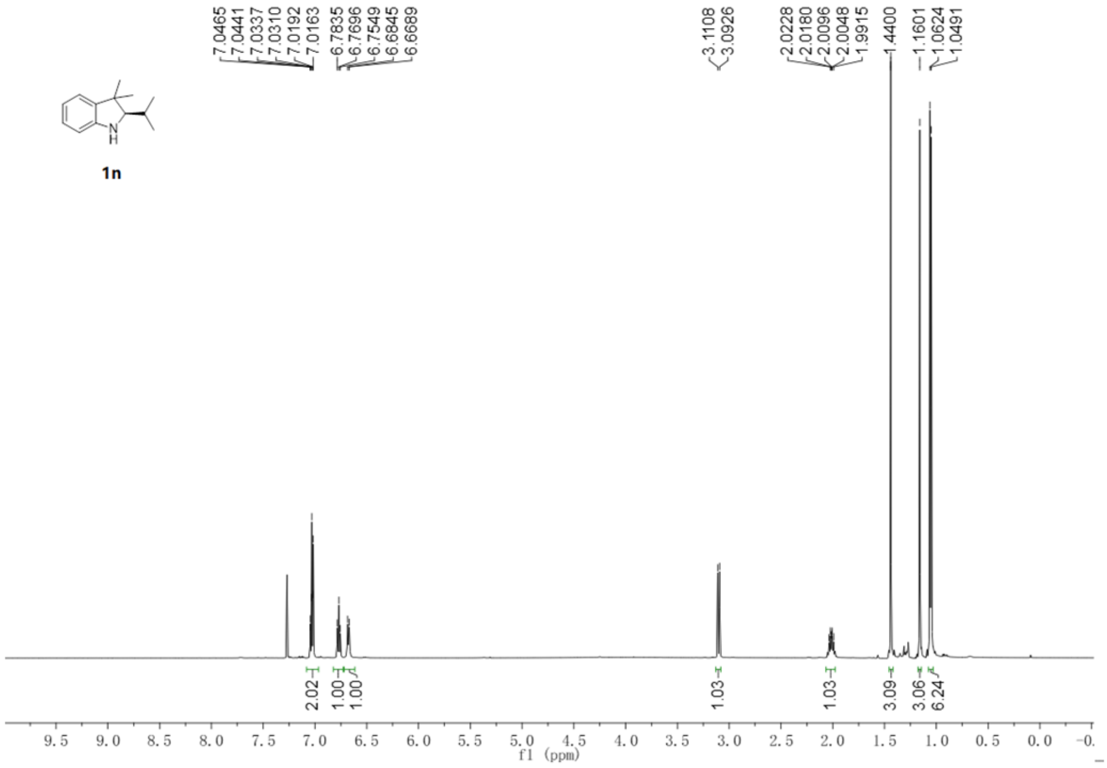

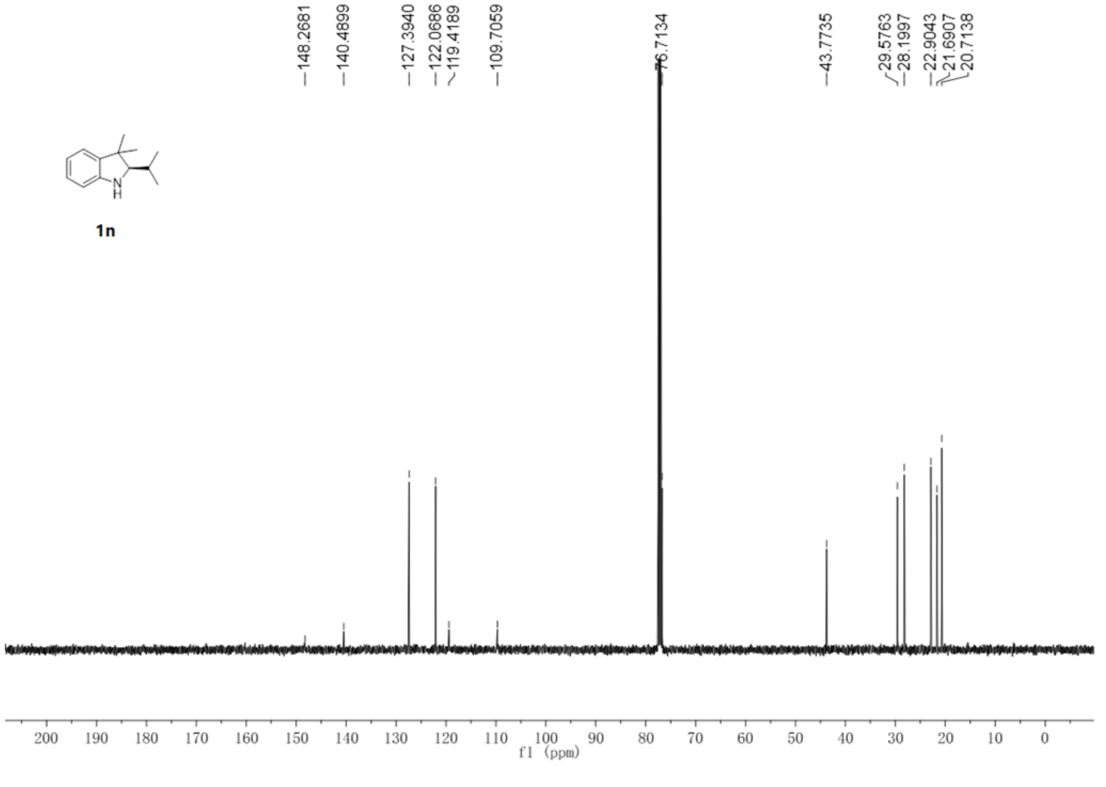

Supplementary figure 36. ^{1}H & ^{13}C NMR spectra of **1n**.

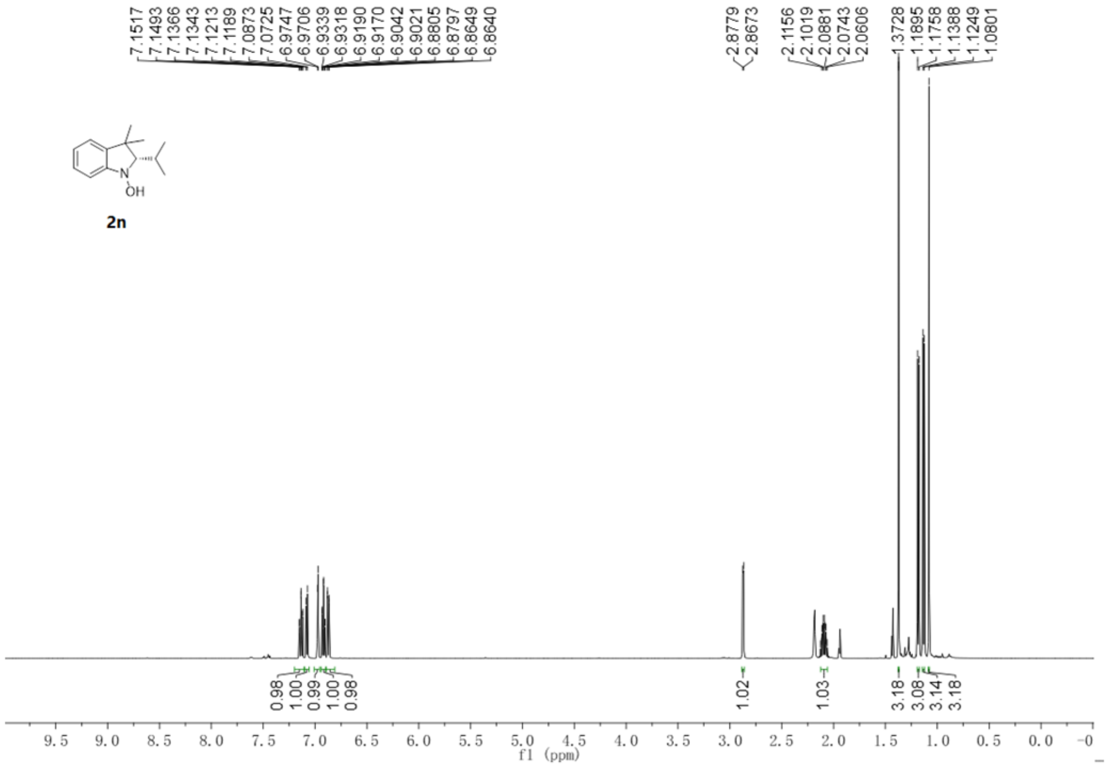

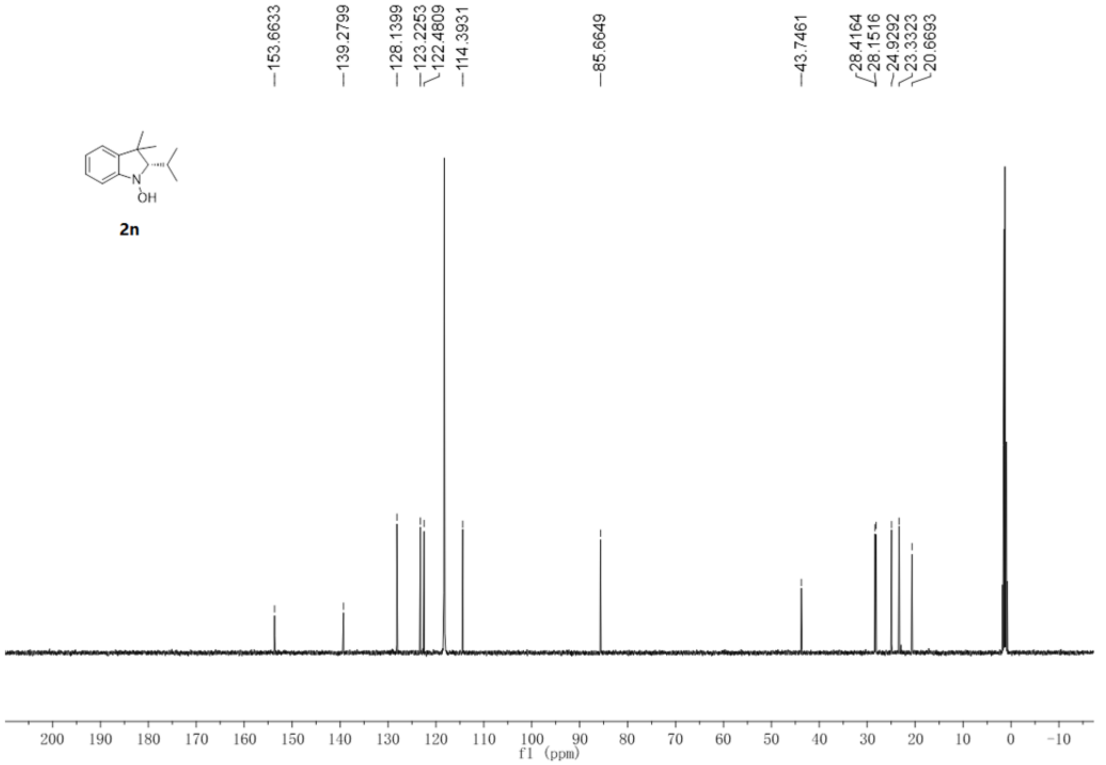

Supplementary figure 37. ^{1}H & ^{13}C NMR spectra of **2n**.

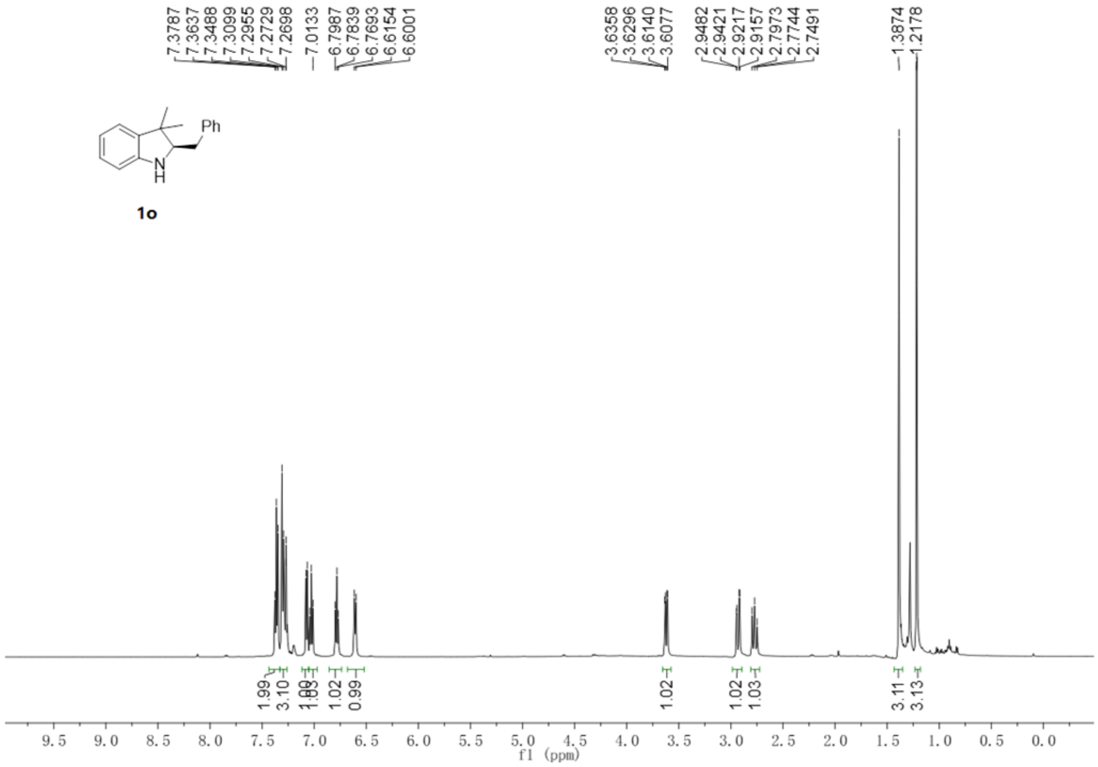

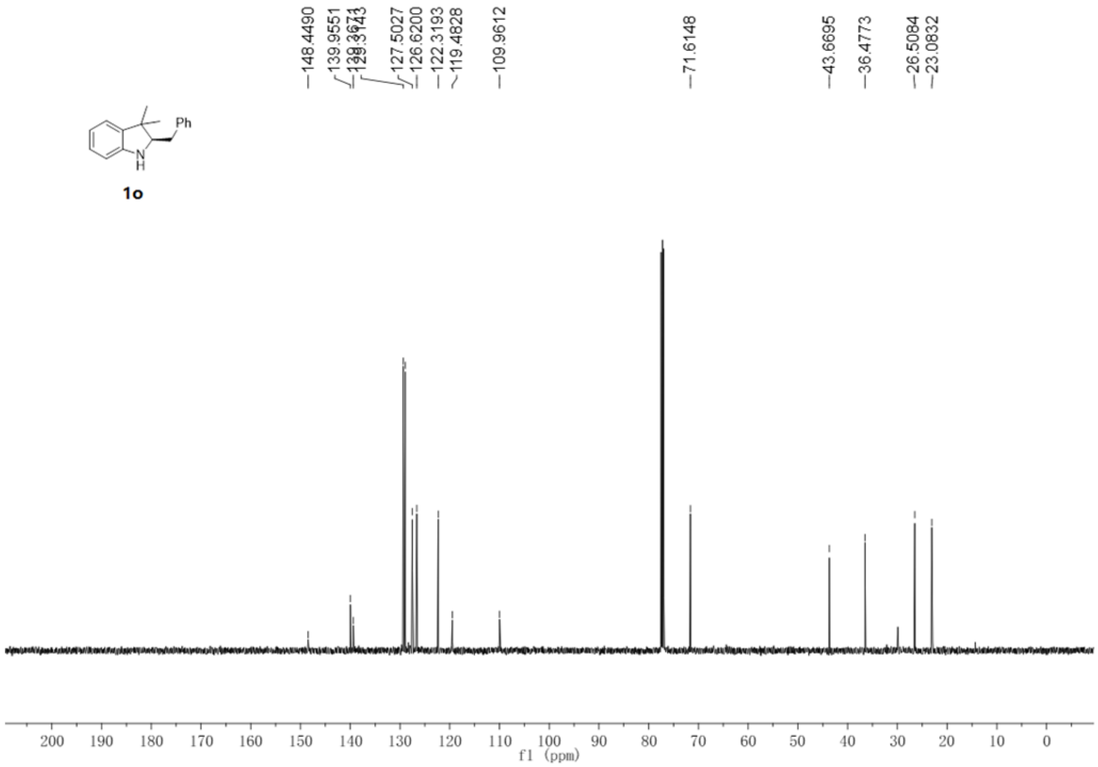

Supplementary figure 38. ^{1}H & ^{13}C NMR spectra of **1o**.

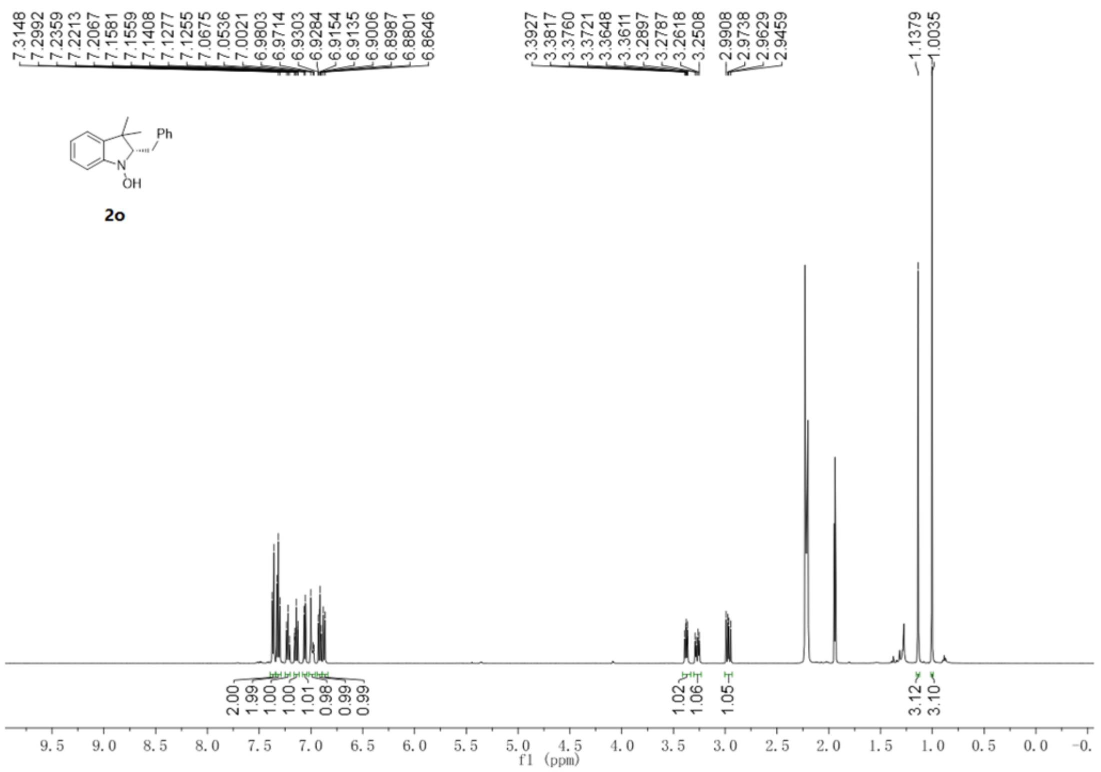

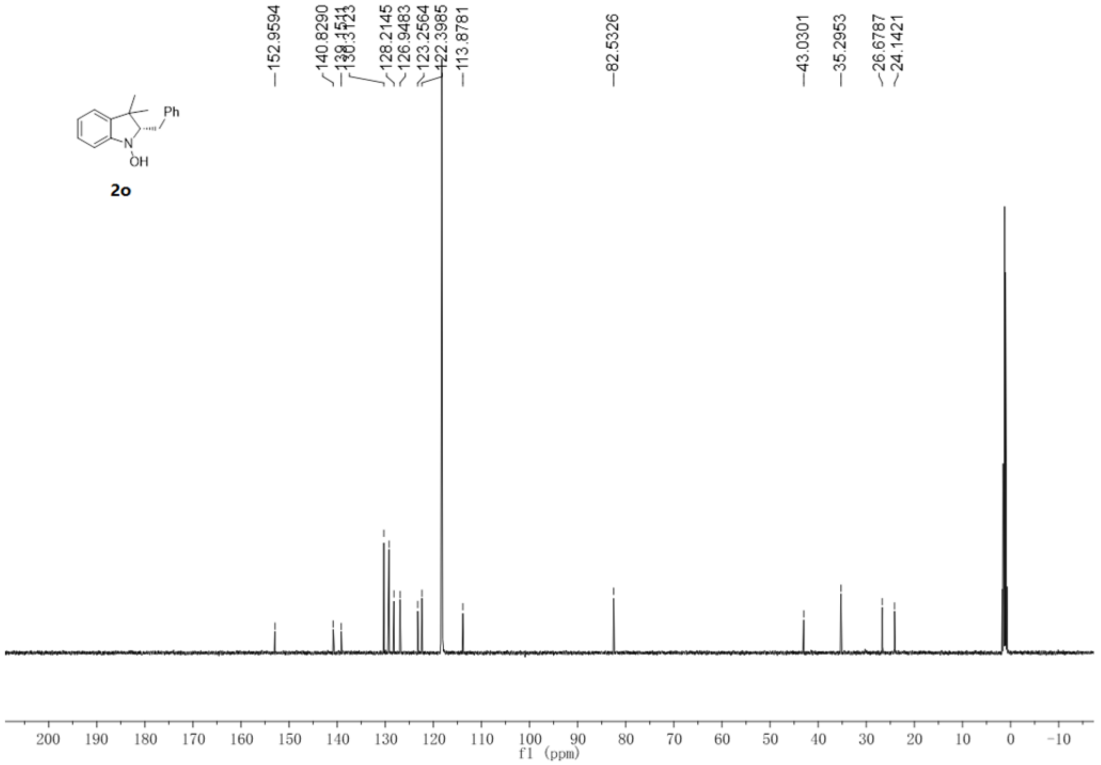

Supplementary figure 39. ^{1}H & ^{13}C NMR spectra of **2o**.

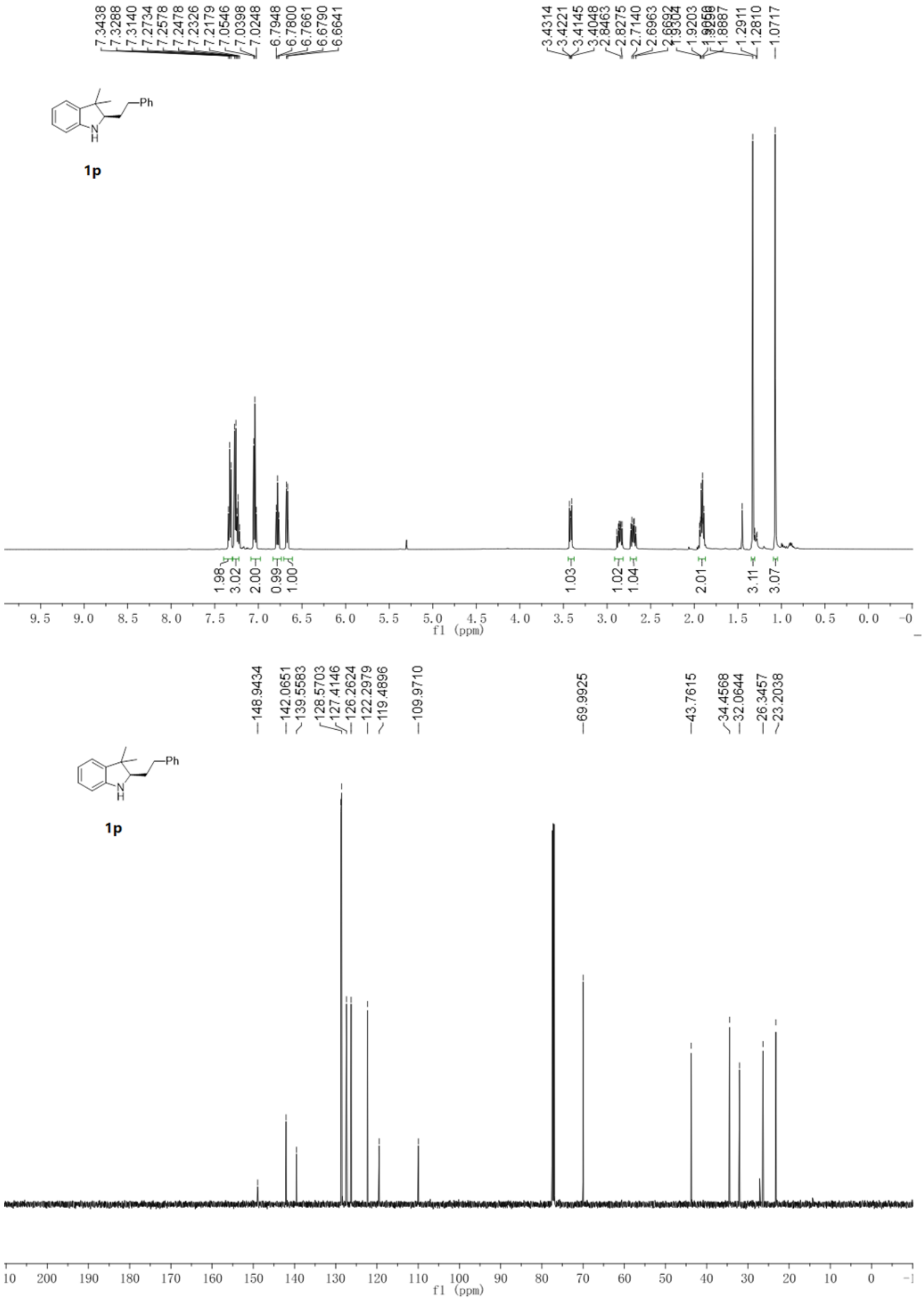

Supplementary figure 40. ^{1}H & ^{13}C NMR spectra of **1p**.

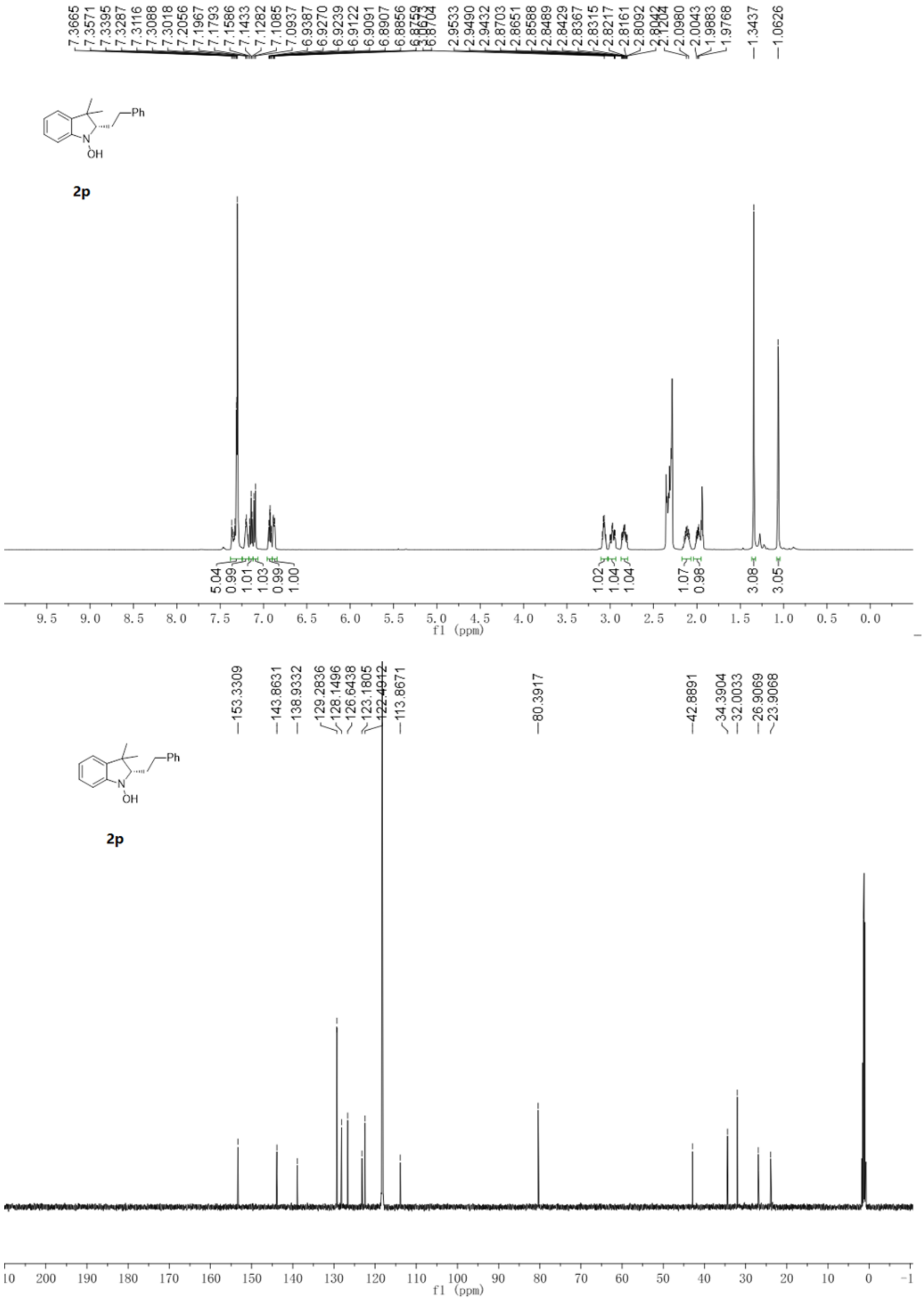

Supplementary figure 41. ^{1}H & ^{13}C NMR spectra of **2p**.

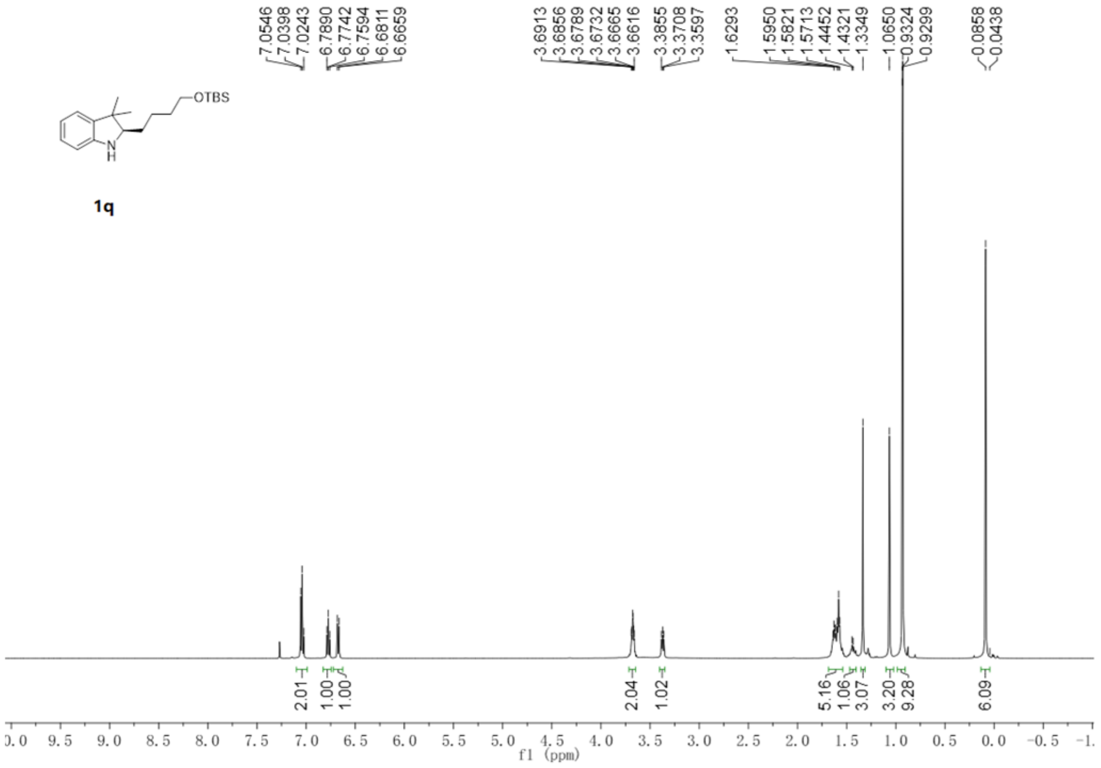

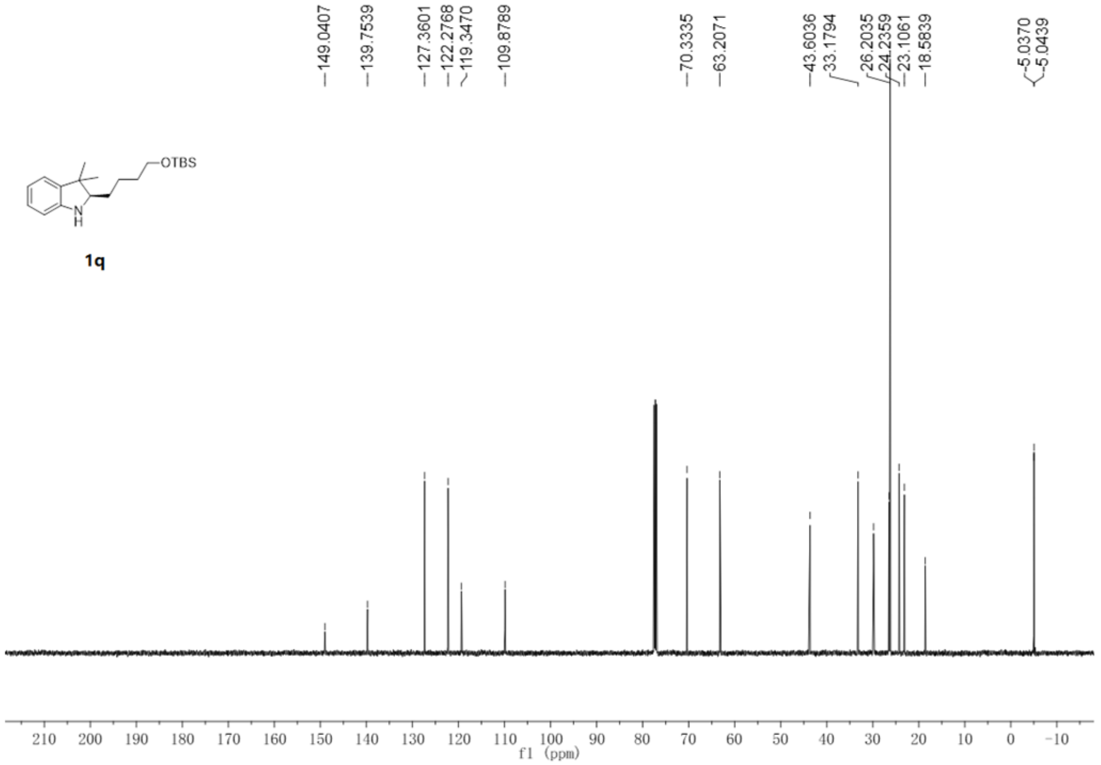

Supplementary figure 42. ^{1}H & ^{13}C NMR spectra of **1q**.

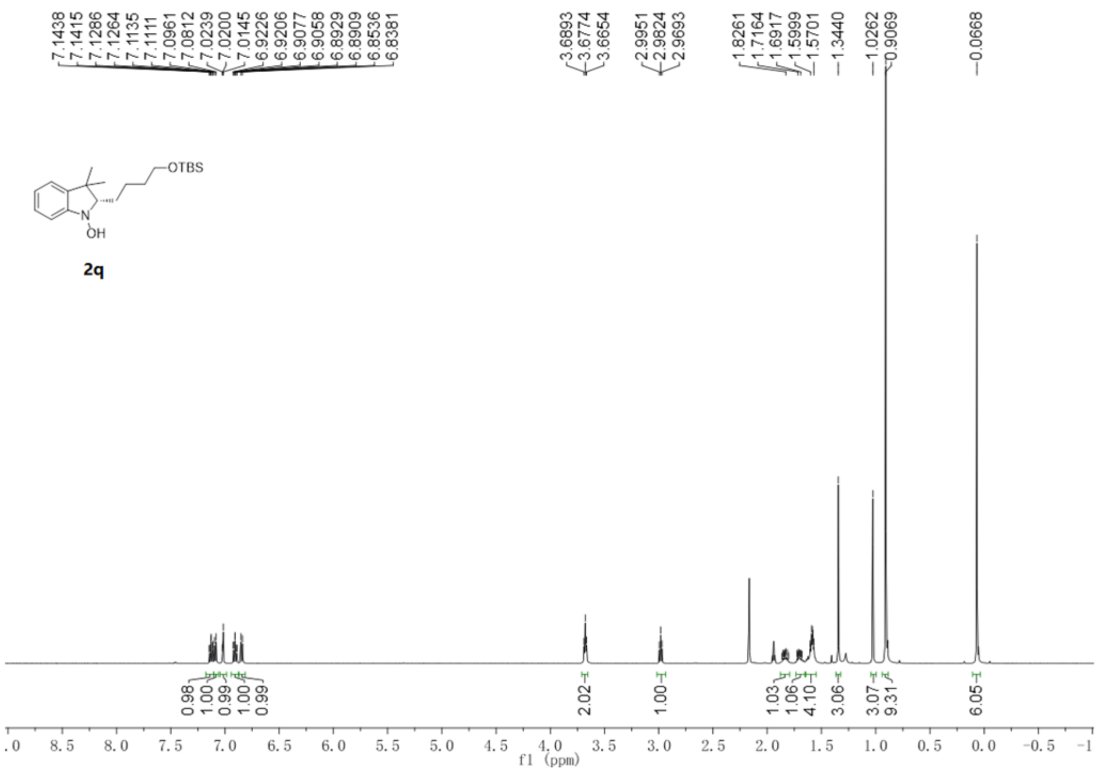

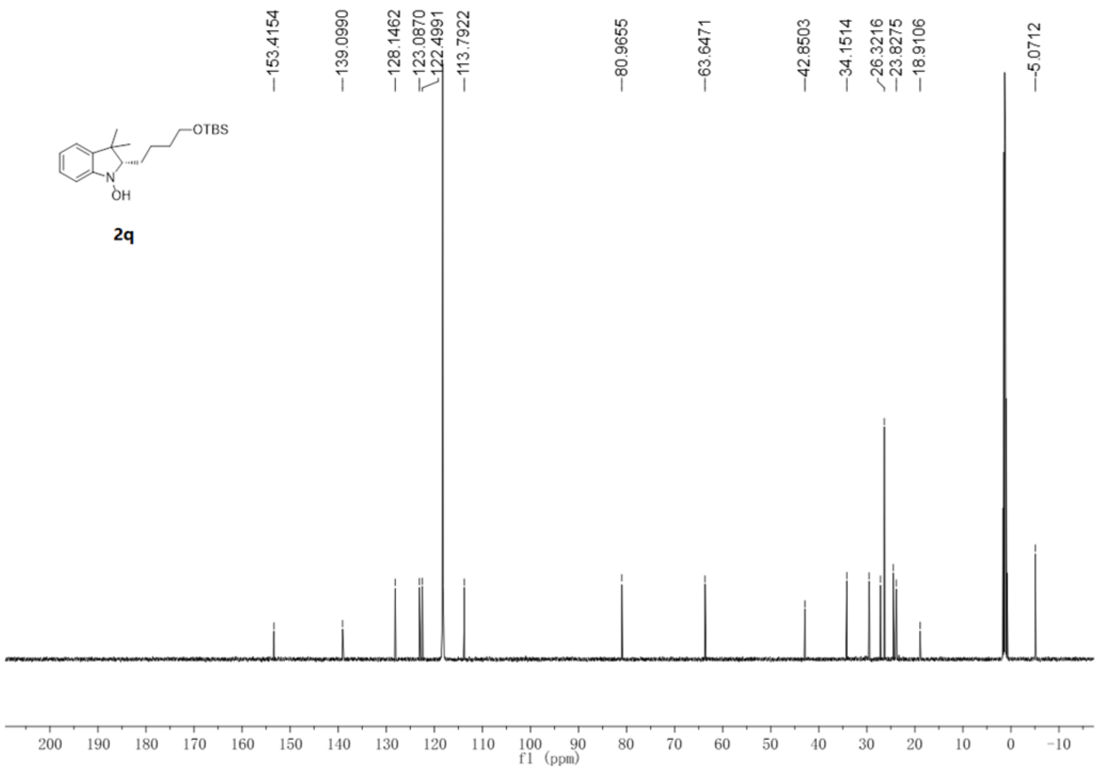

Supplementary figure 43. ^{1}H & ^{13}C NMR spectra of **2q**.

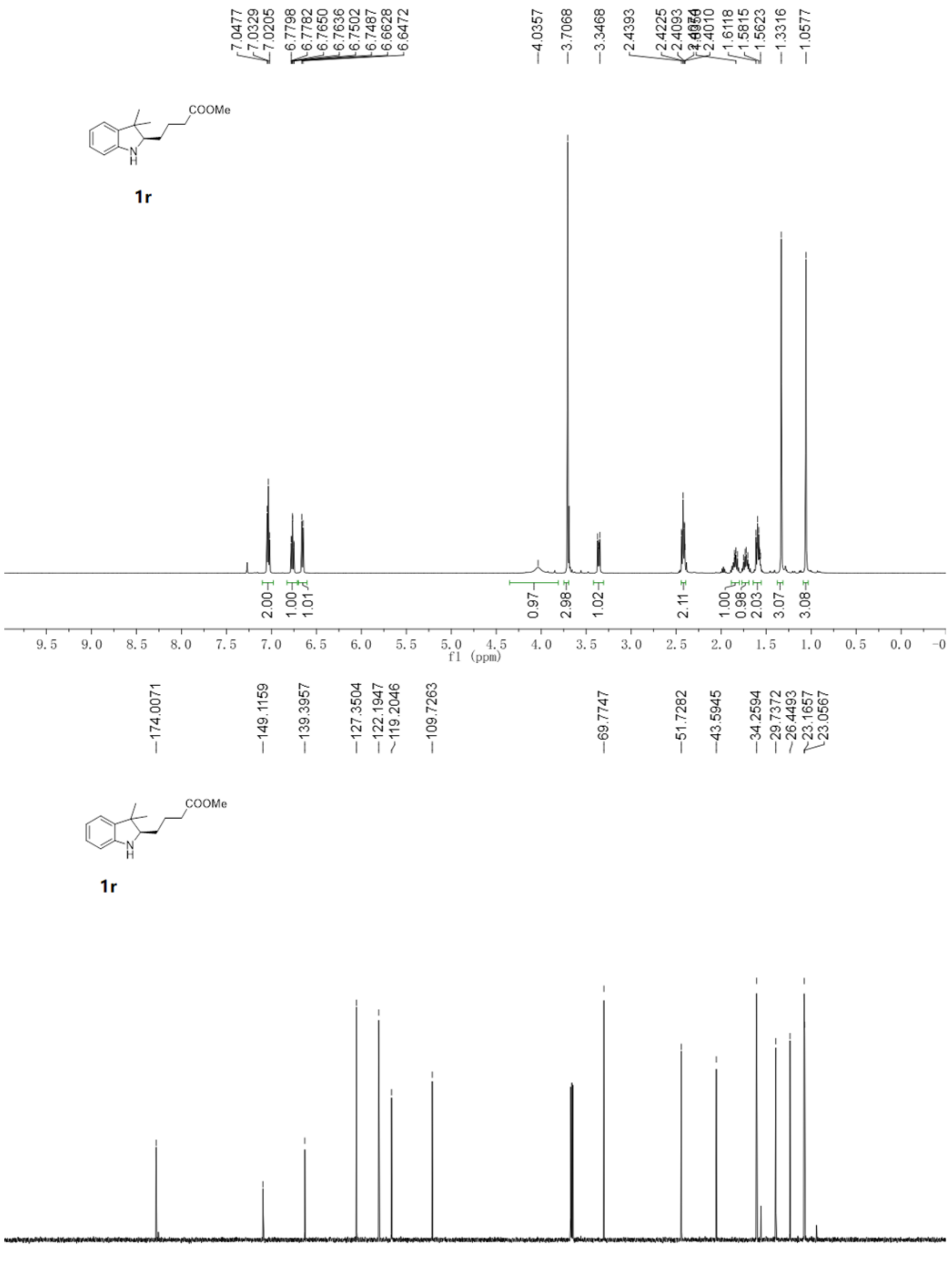

Supplementary figure 44. ^{1}H & ^{13}C NMR spectra of **1r**.

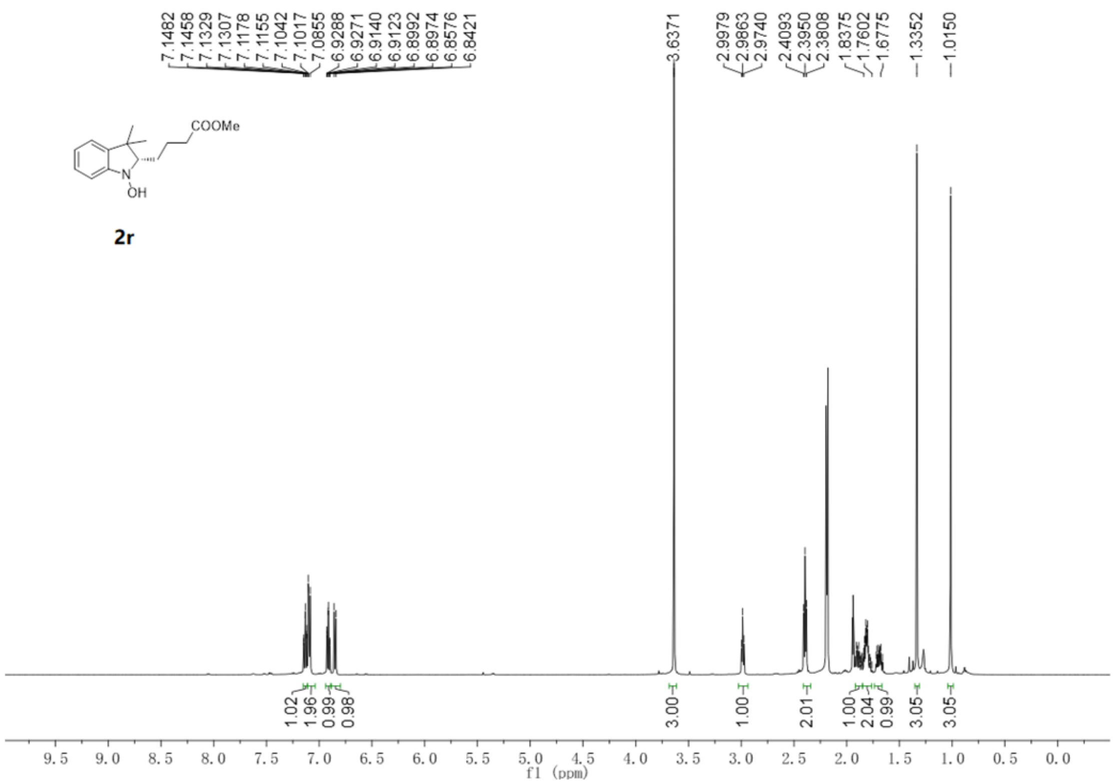

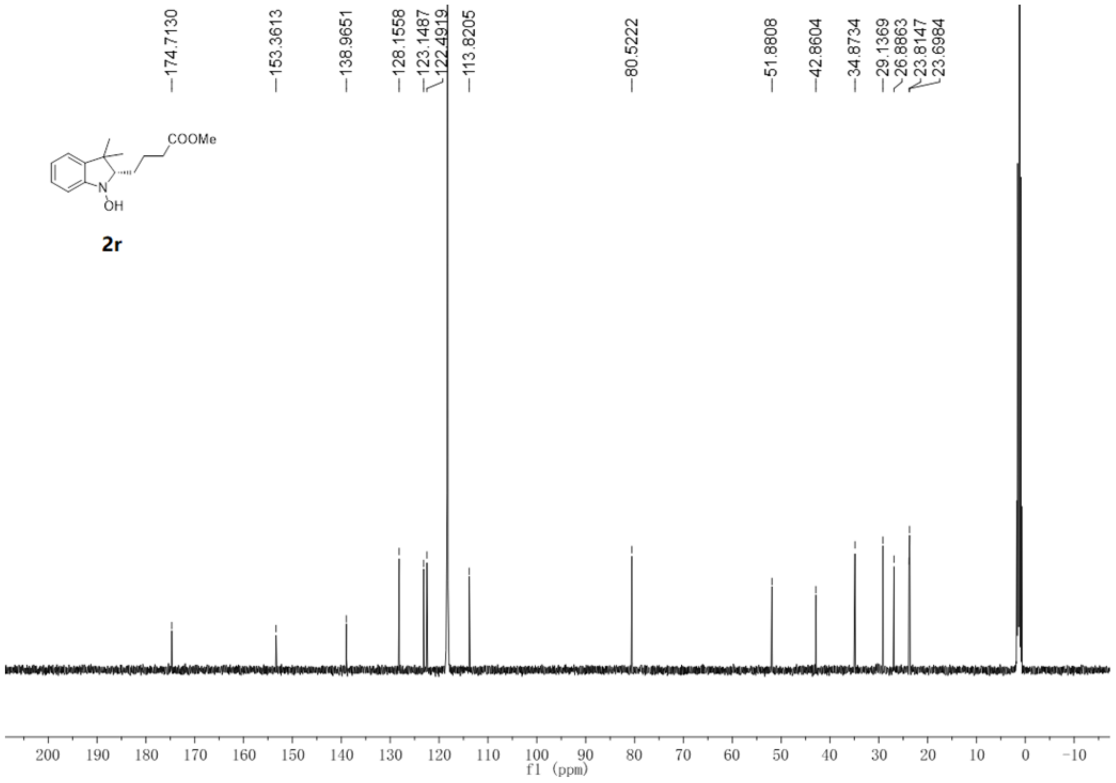

Supplementary figure 45. ^{1}H & ^{13}C NMR spectra of **2r**.

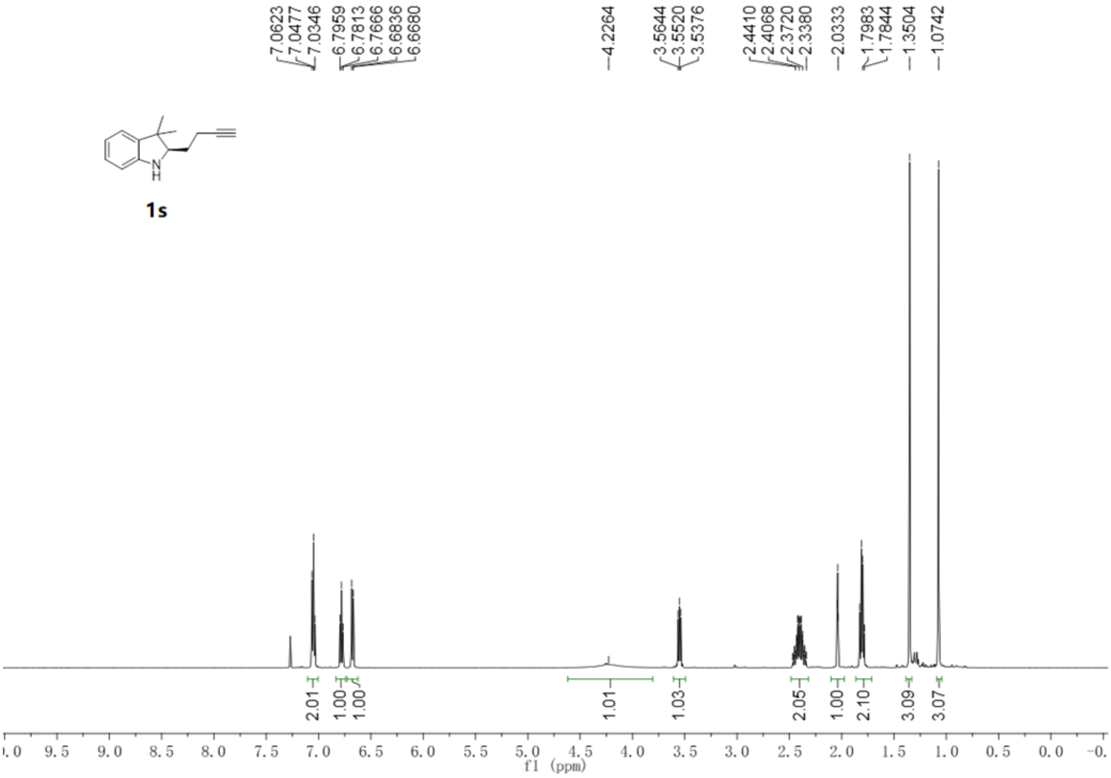

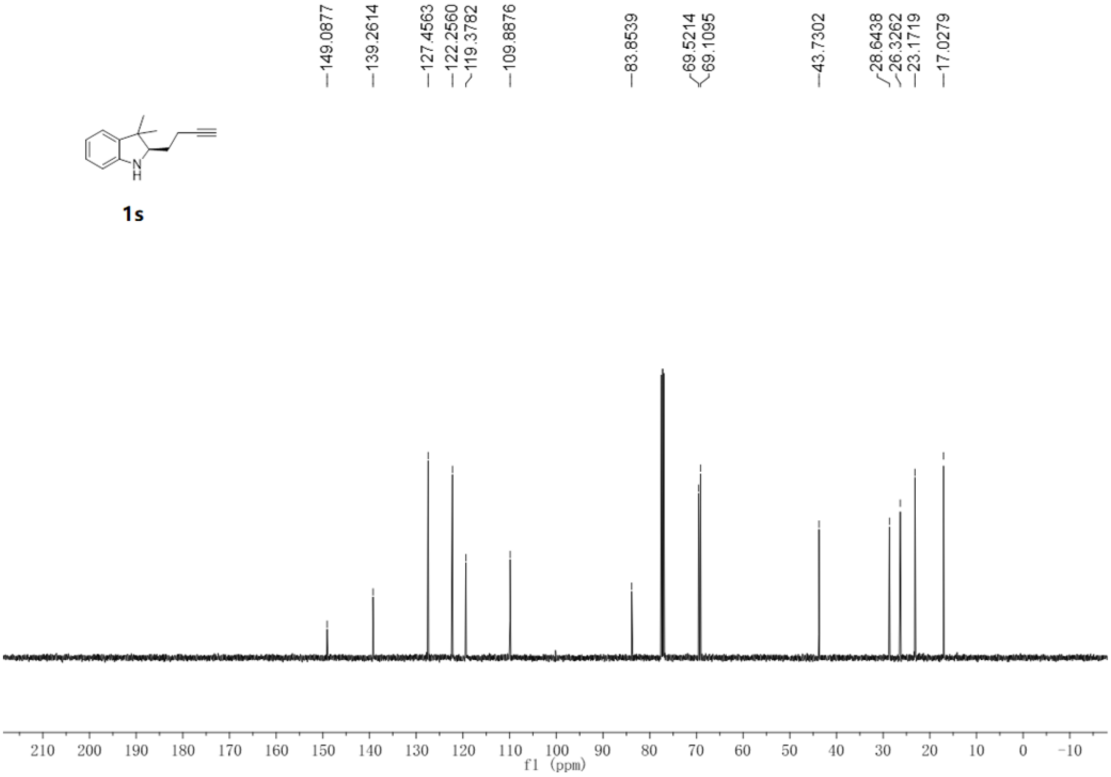

Supplementary figure 46. ^{1}H & ^{13}C NMR spectra of **1s**.

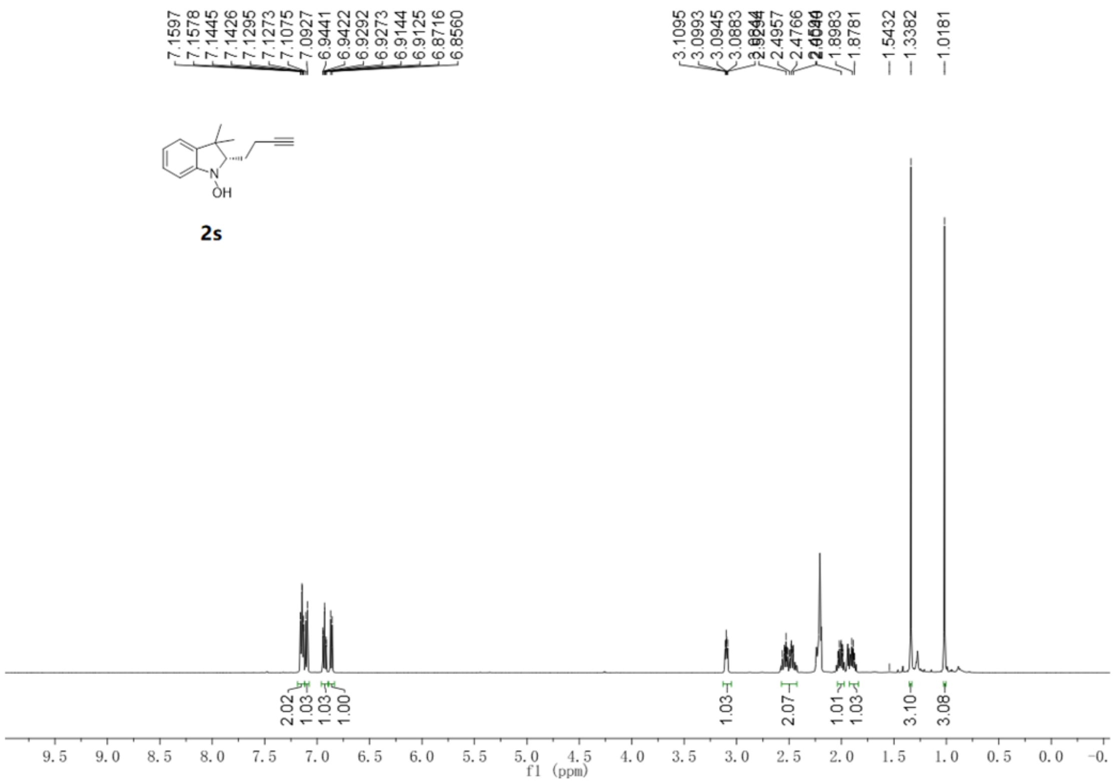

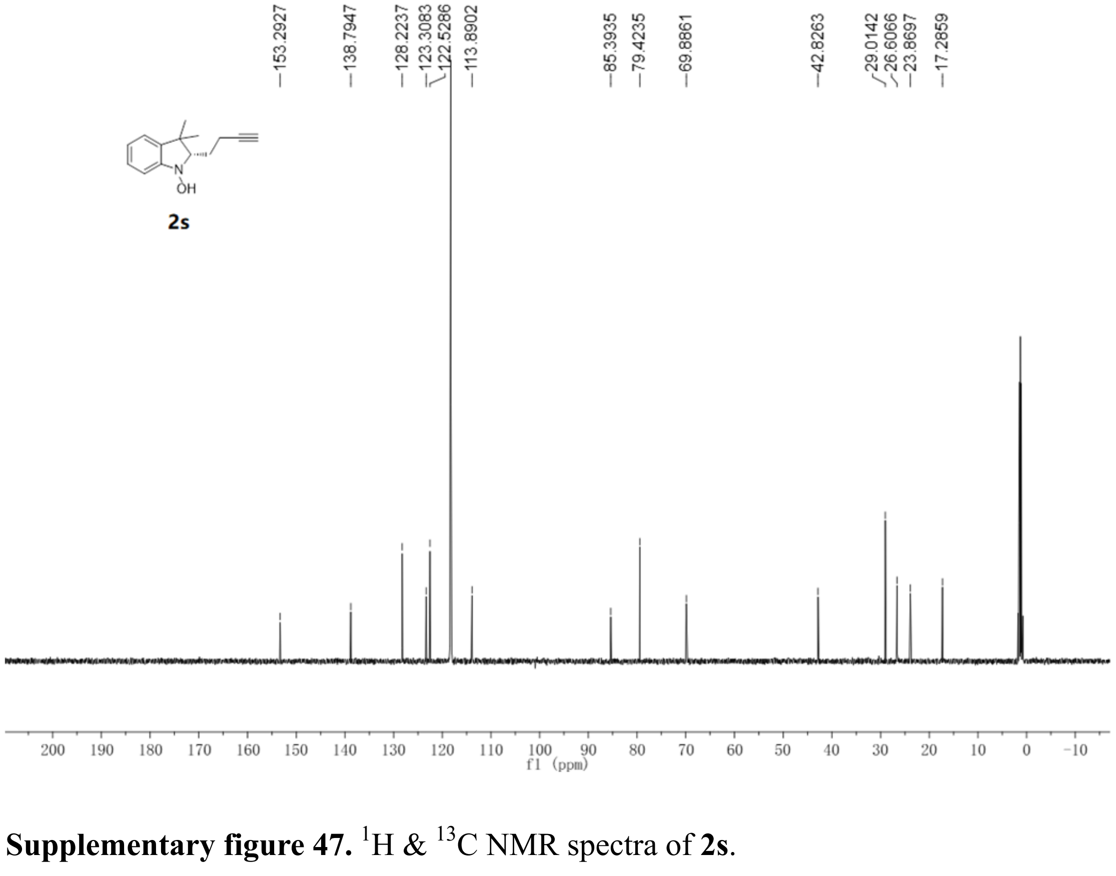

Supplementary figure 47. ^{1}H & ^{13}C NMR spectra of **2s**.

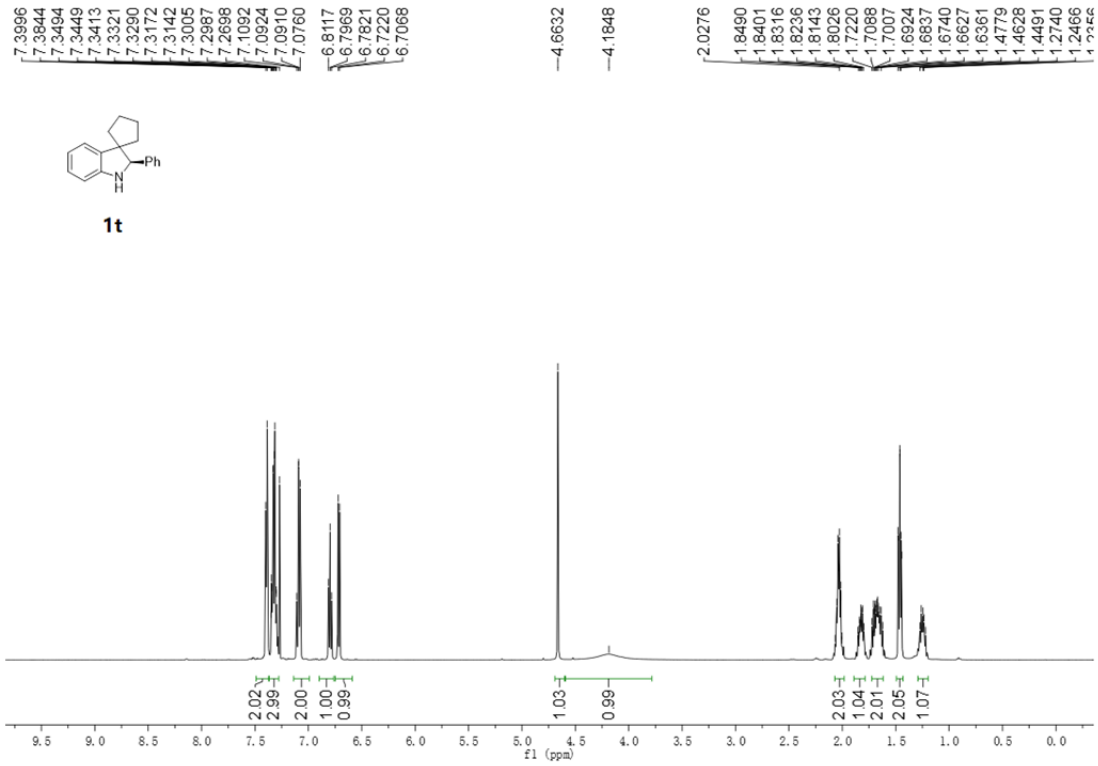

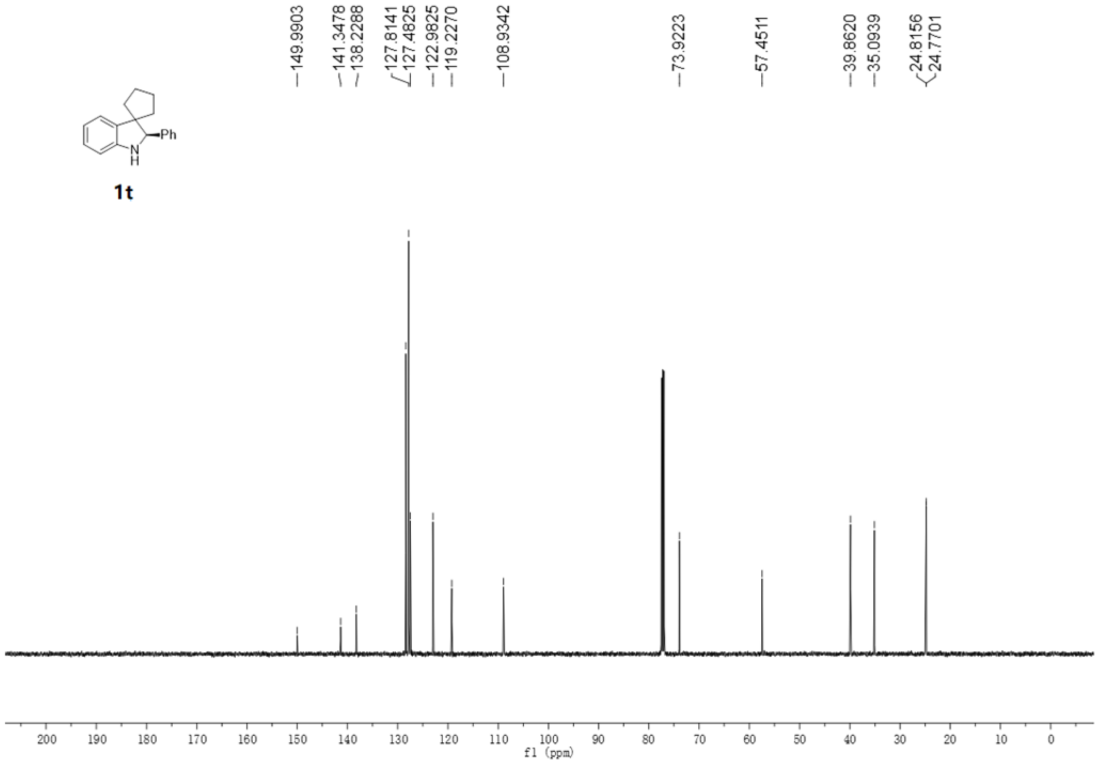

Supplementary figure 48. ^{1}H & ^{13}C NMR spectra of **1t**.

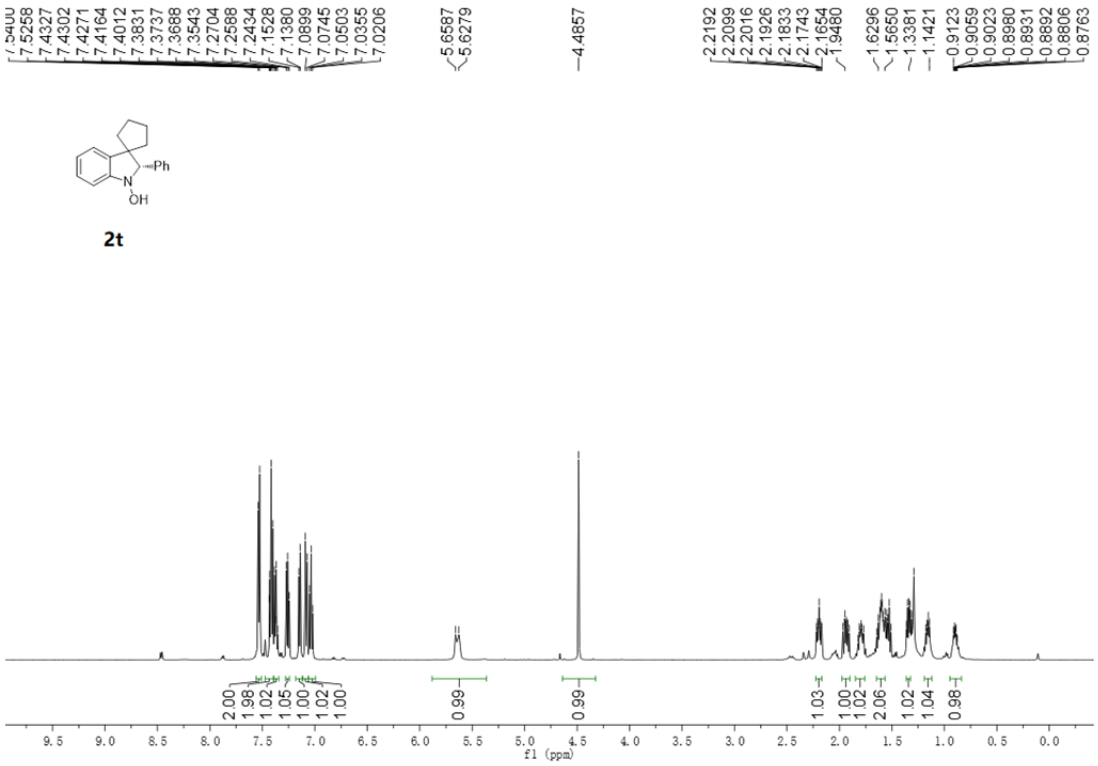

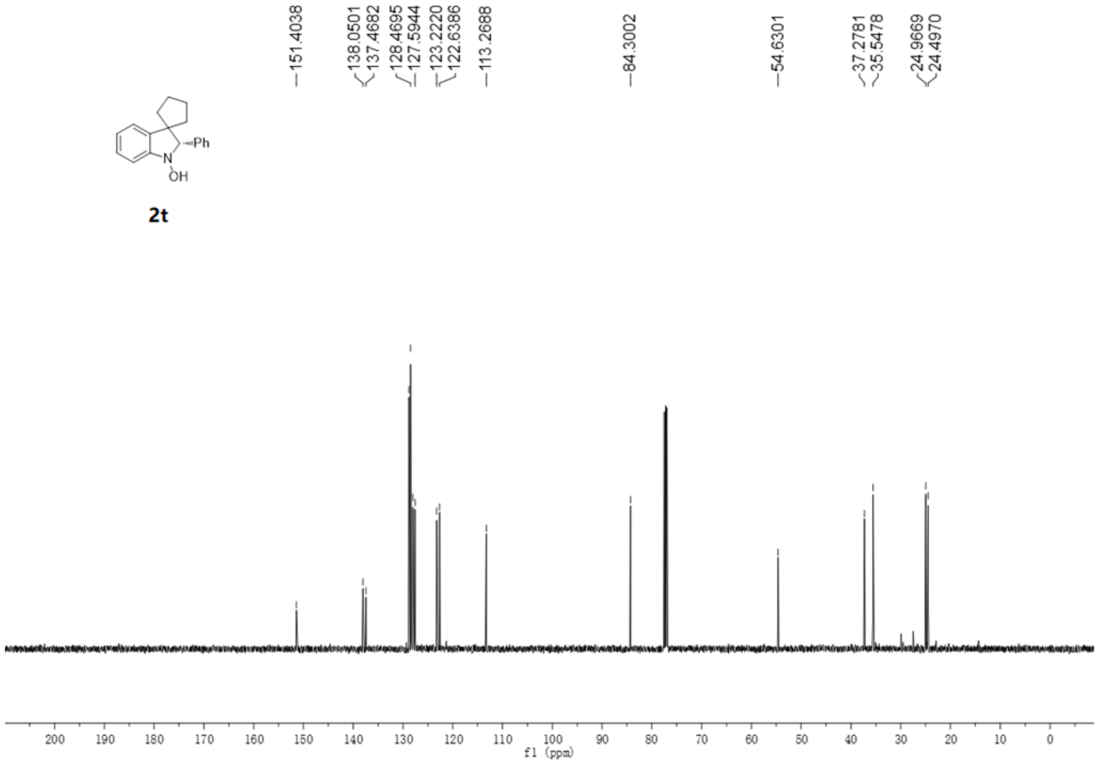

Supplementary figure 49. ^{1}H & ^{13}C NMR spectra of **2t**.

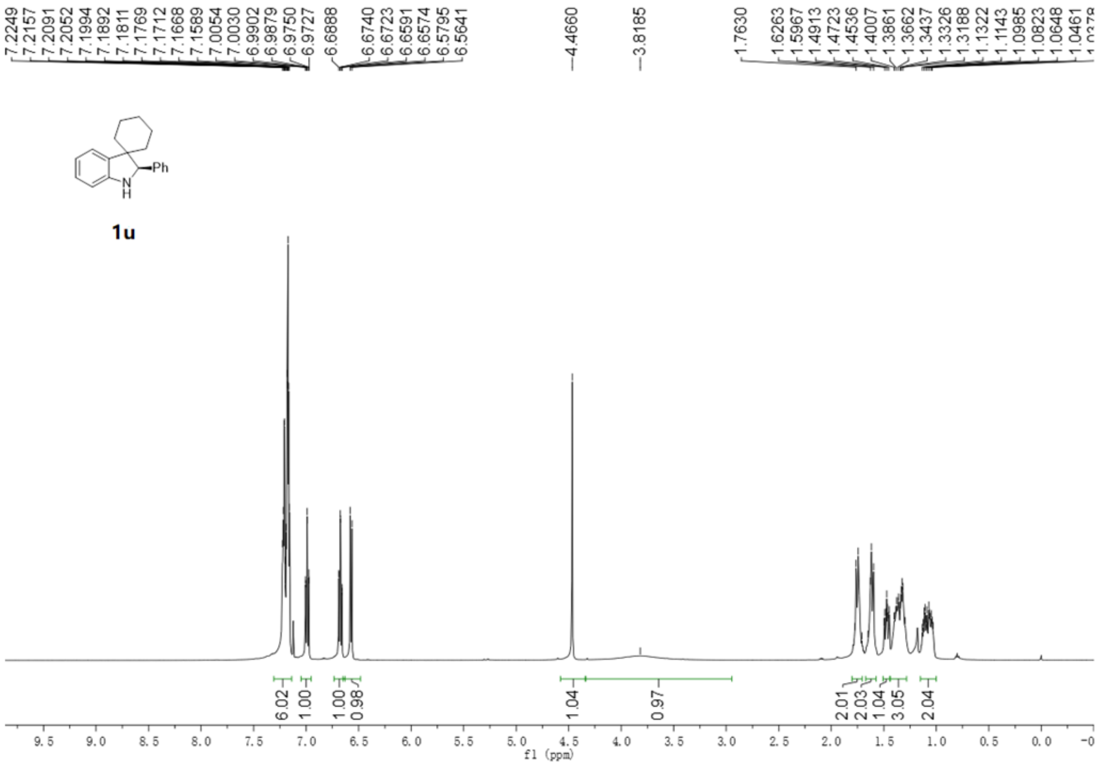

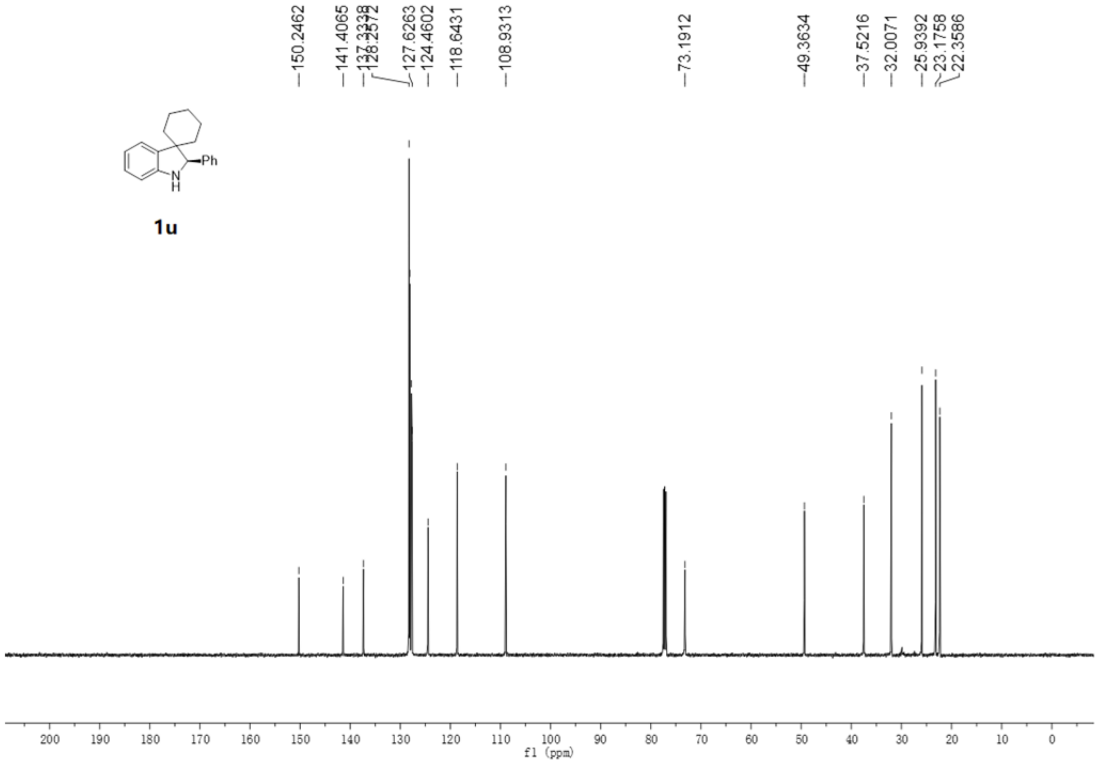

Supplementary figure 50 ^{1}H & ^{13}C NMR spectra of **1u**.

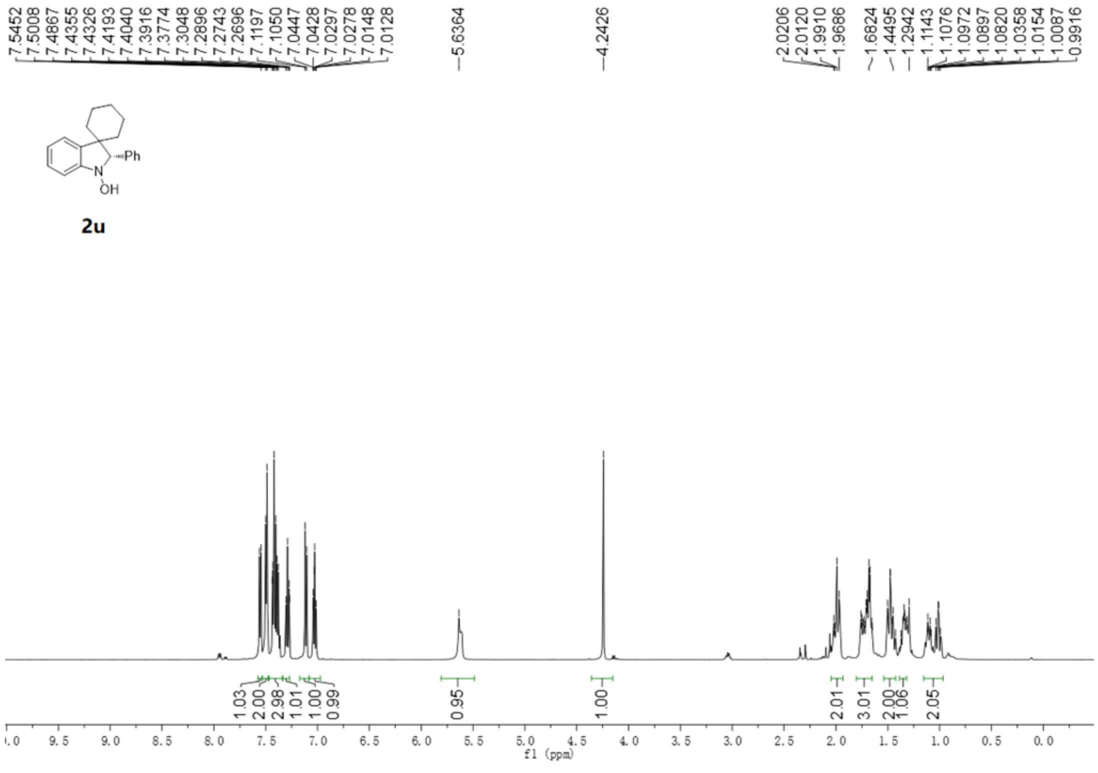

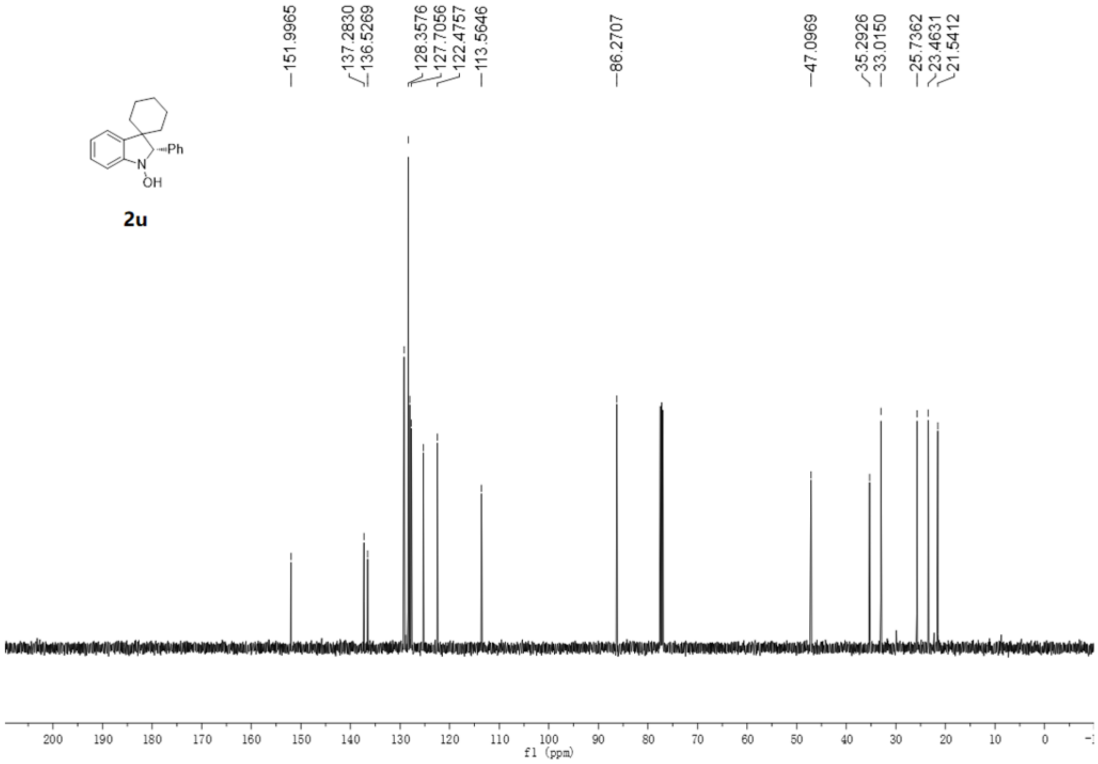

Supplementary figure 51 ^{1}H & ^{13}C NMR spectra of **2u**.

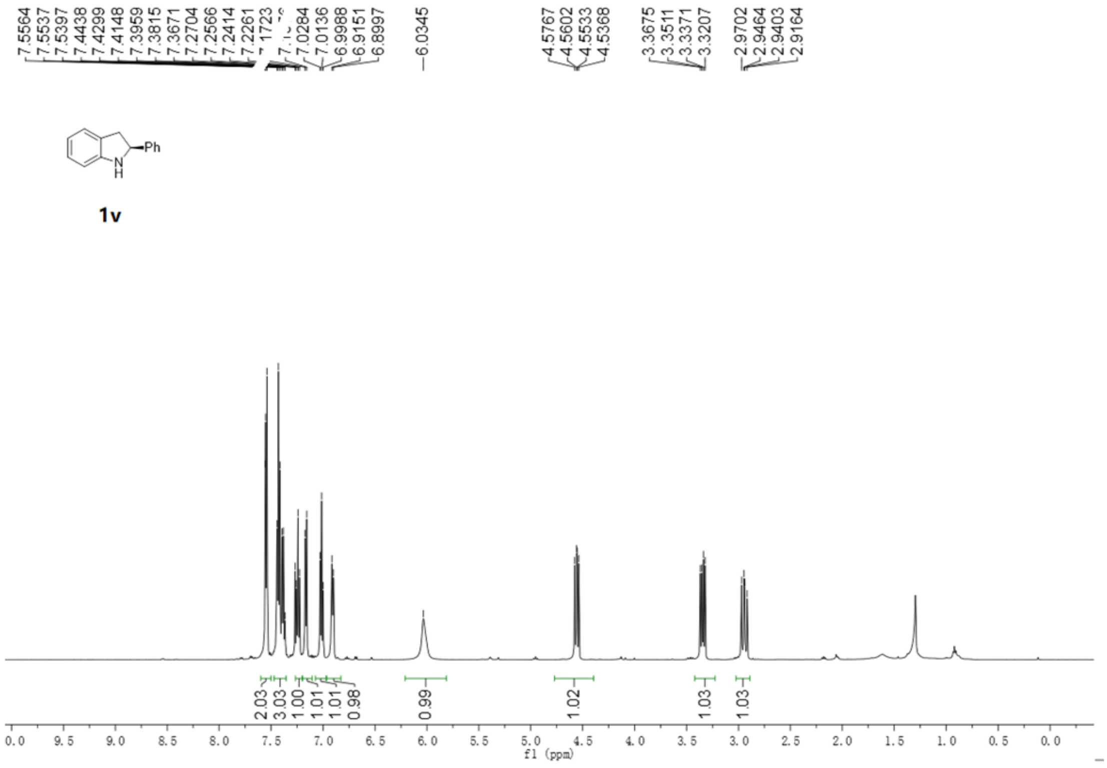

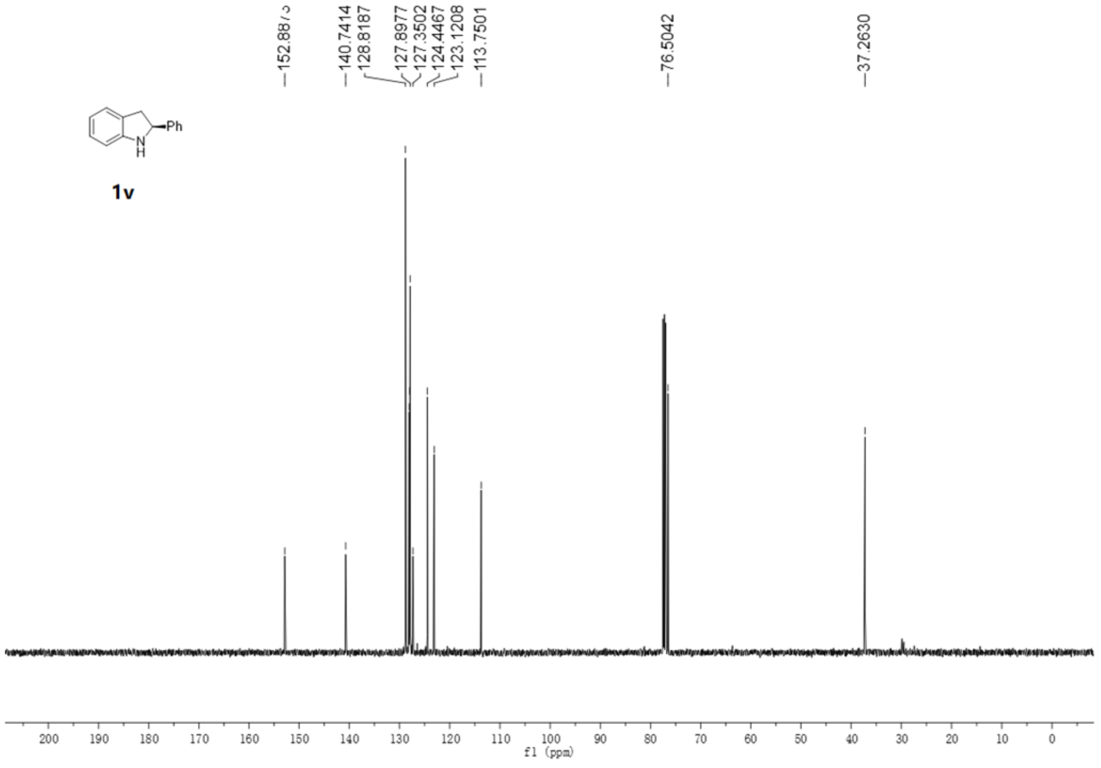

Supplementary figure 52^{1}H & ^{13}C NMR spectra of **1v**.

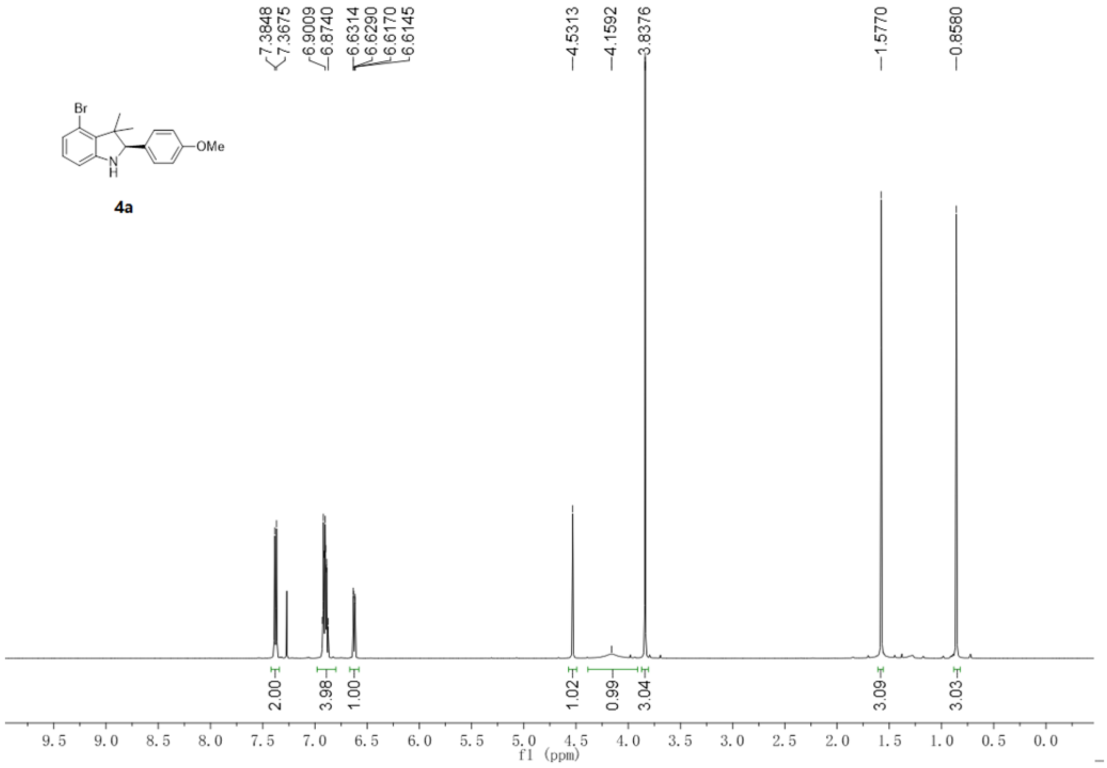

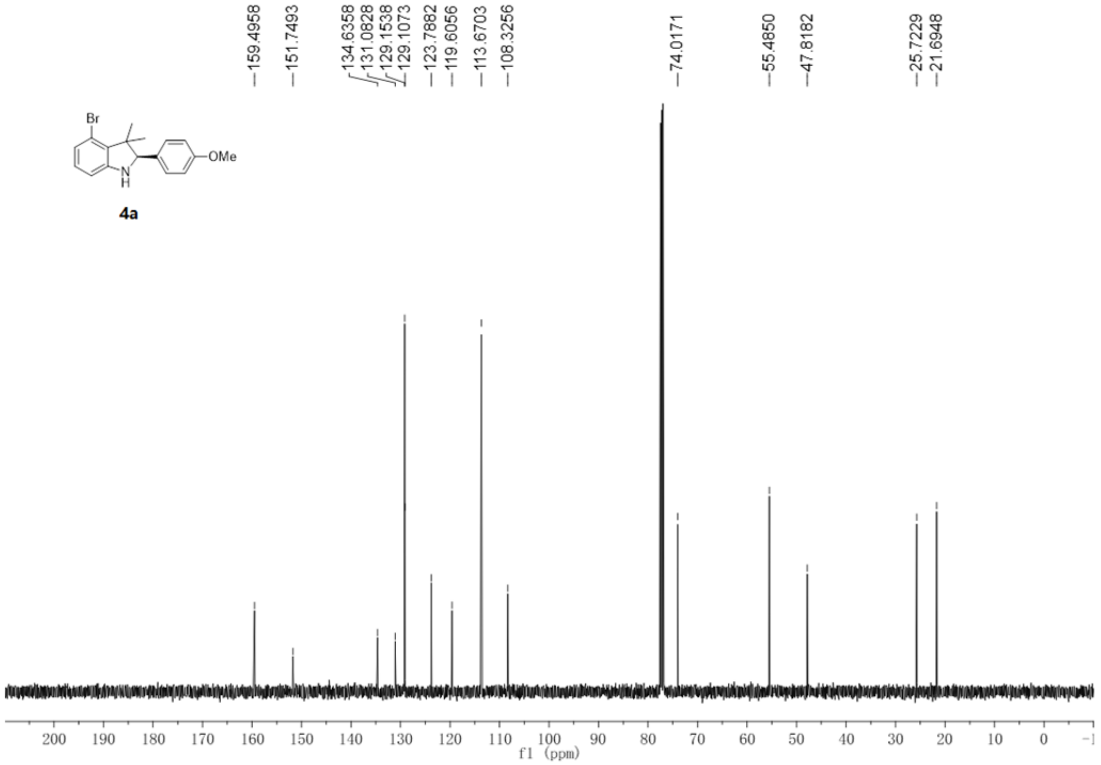

Supplementary figure 53 ^{1}H & ^{13}C NMR spectra of **4a**.

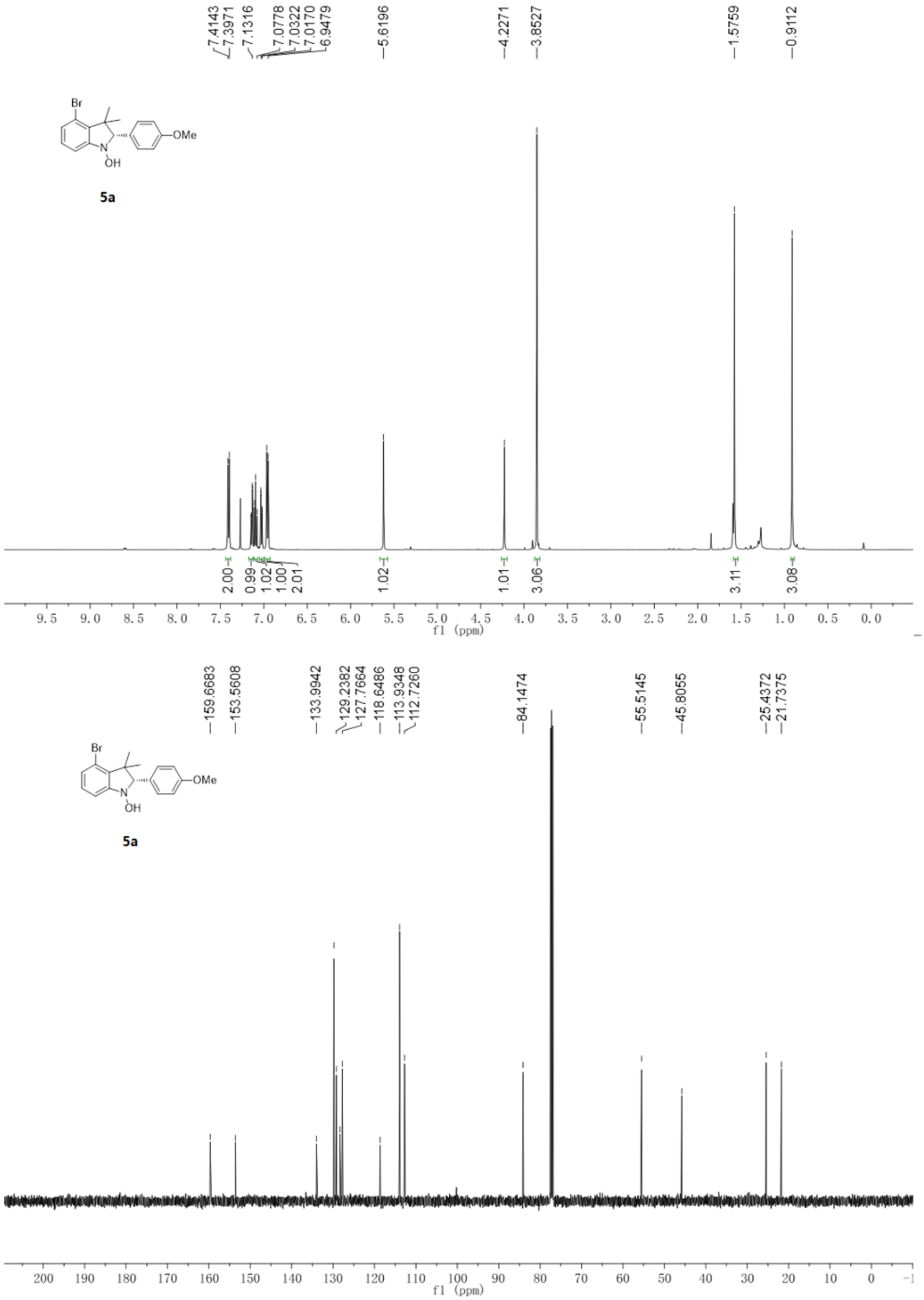

Supplementary figure 54 ^{1}H & ^{13}C NMR spectra of **5a**.

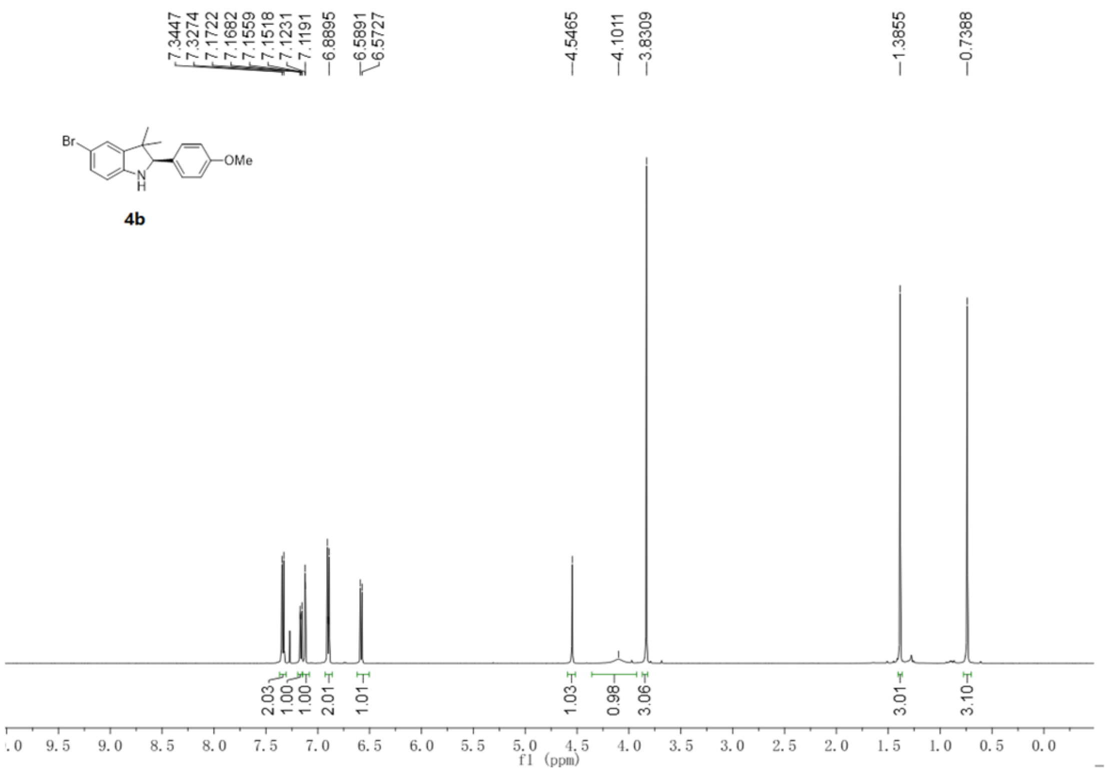

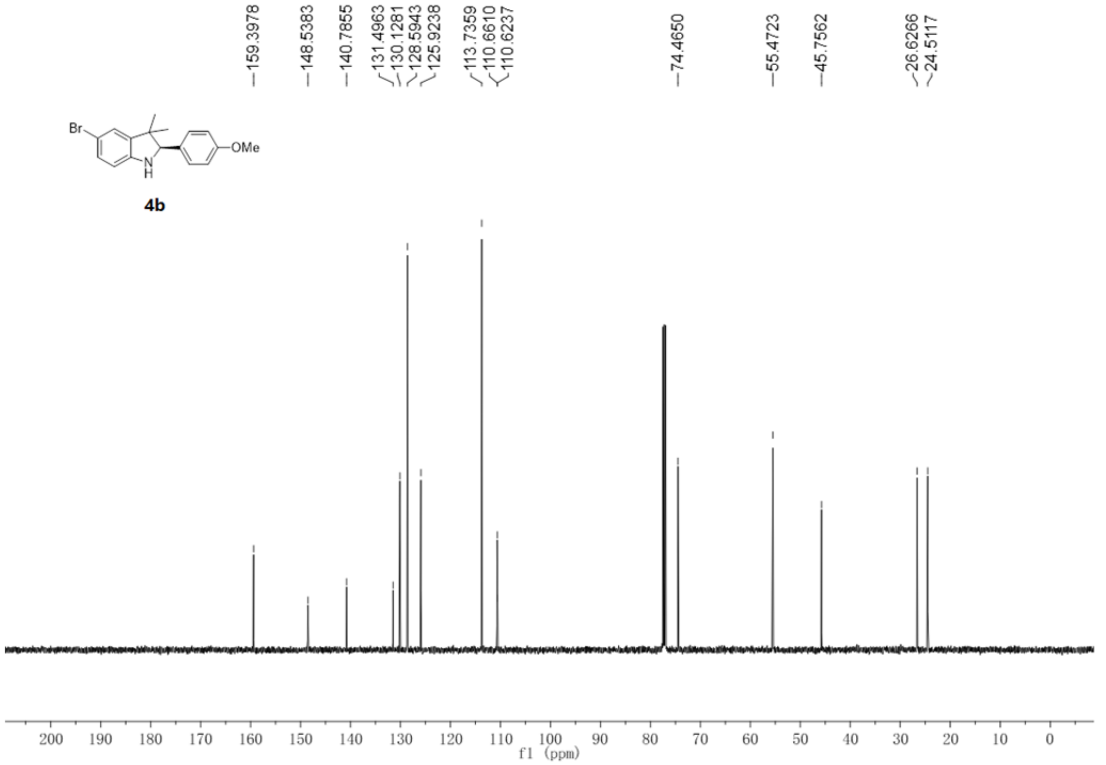

Supplementary figure 55 ^{1}H & ^{13}C NMR spectra of **4b**.

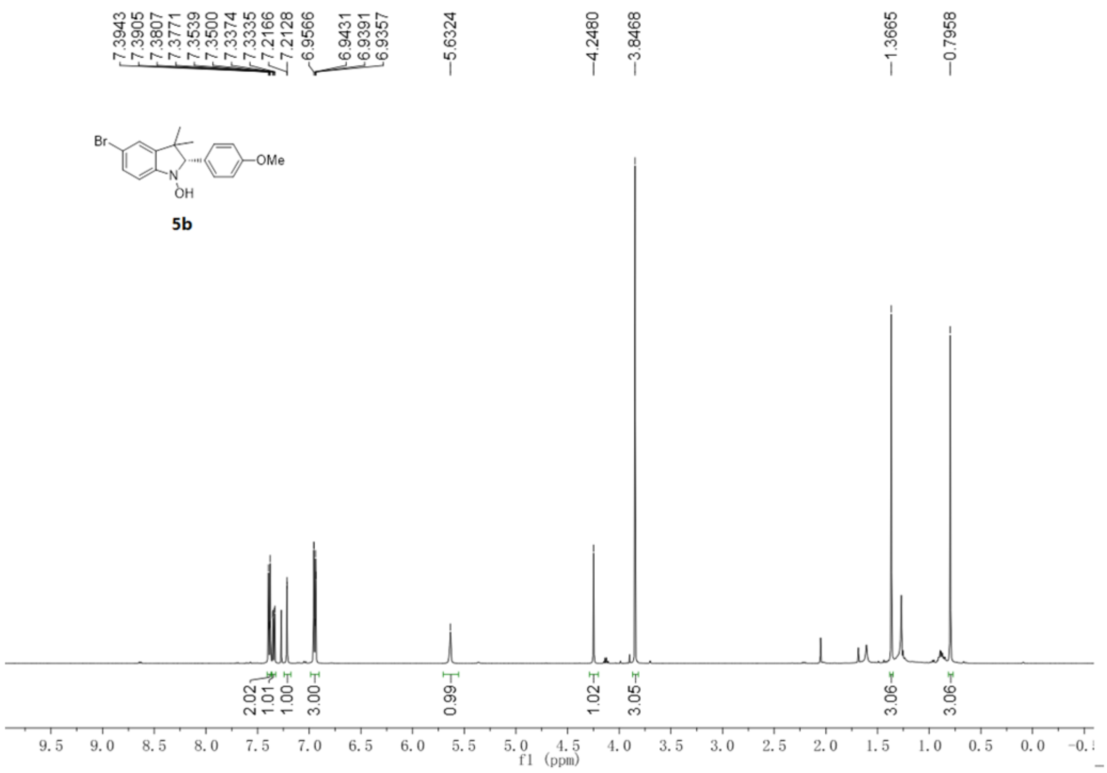

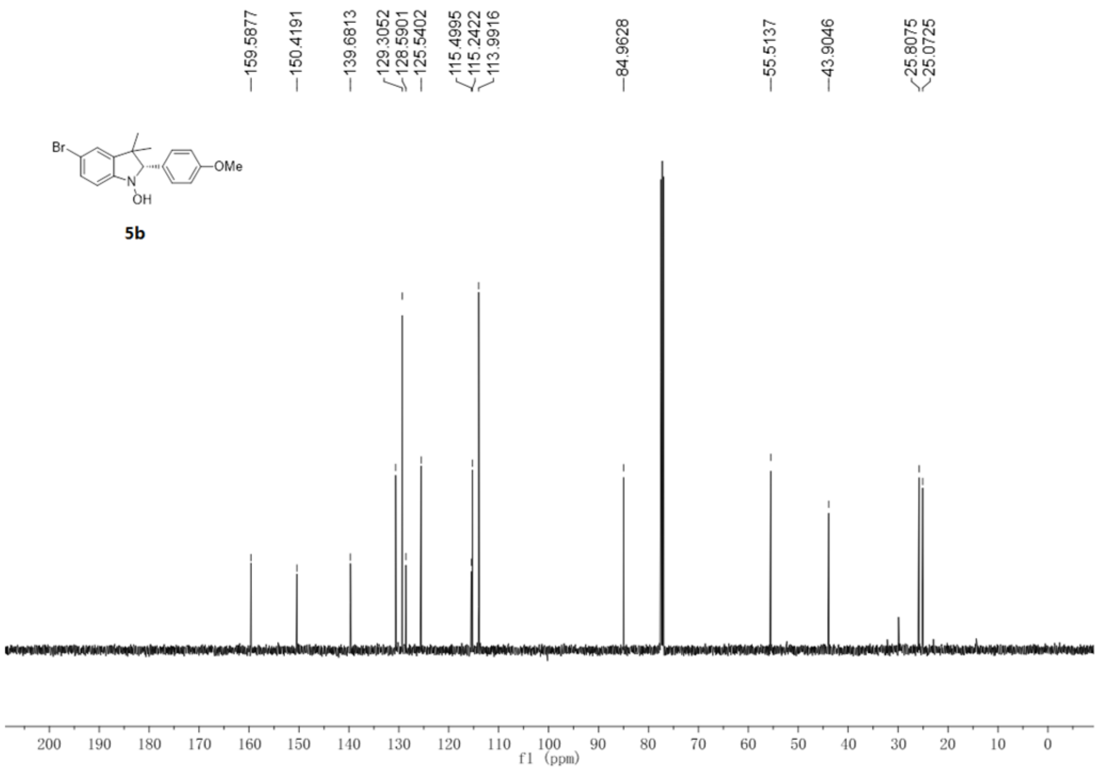

Supplementary figure 56 ^{1}H & ^{13}C NMR spectra of **5b**.

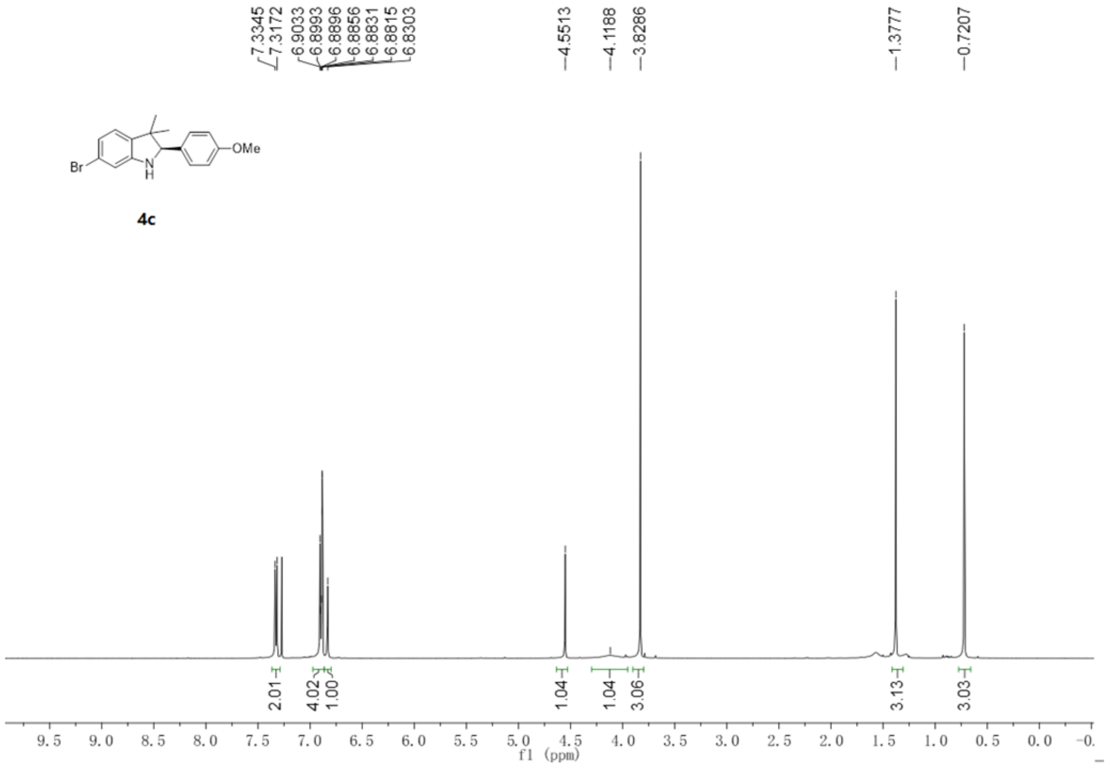

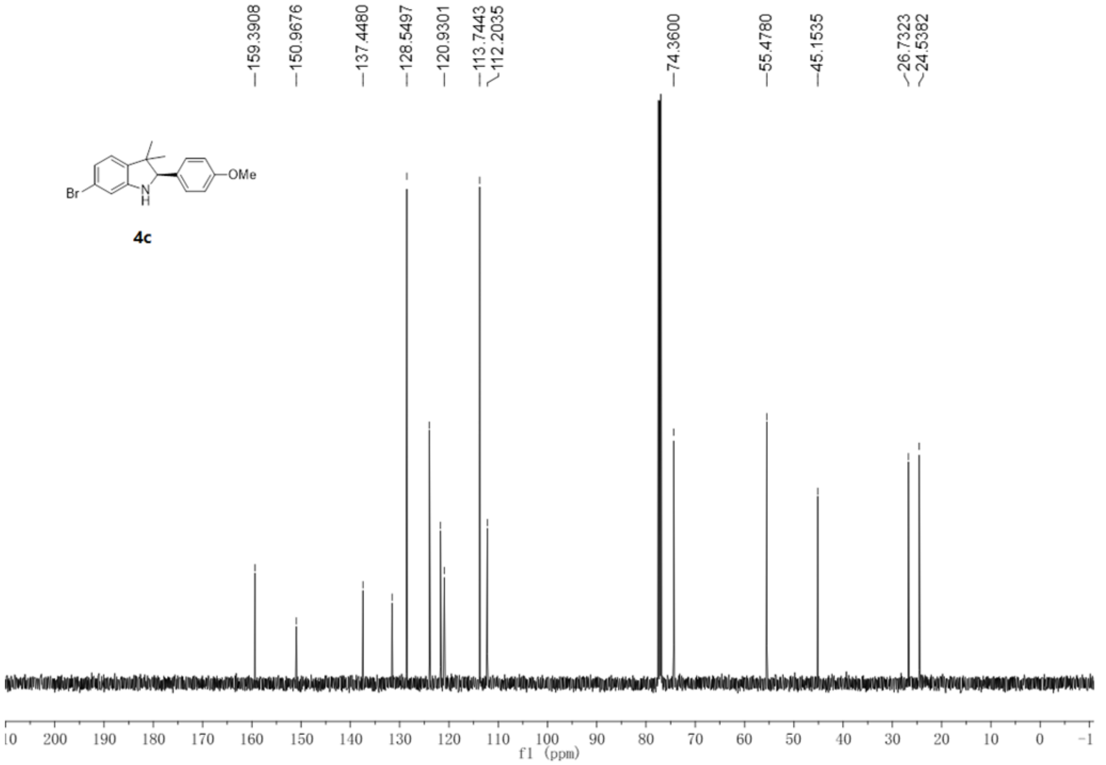

Supplementary figure 57 ^{1}H & ^{13}C NMR spectra of **4c**.

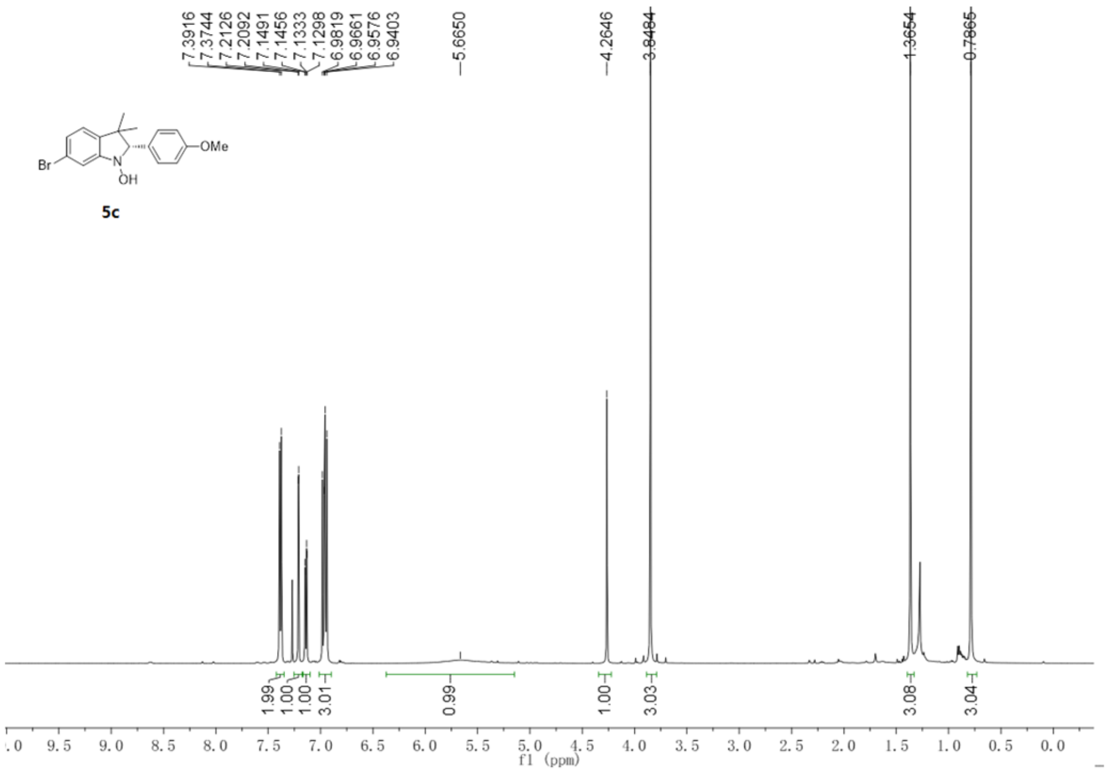

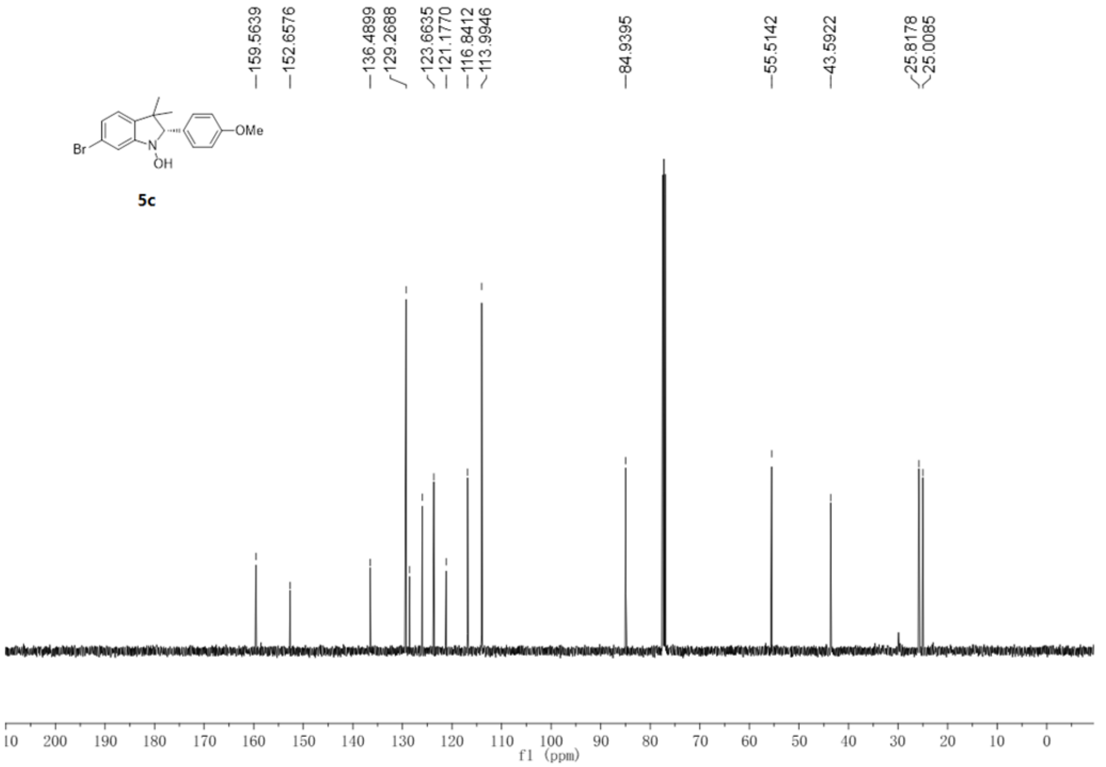

Supplementary figure 58^{1}H & ^{13}C NMR spectra of **5c**.

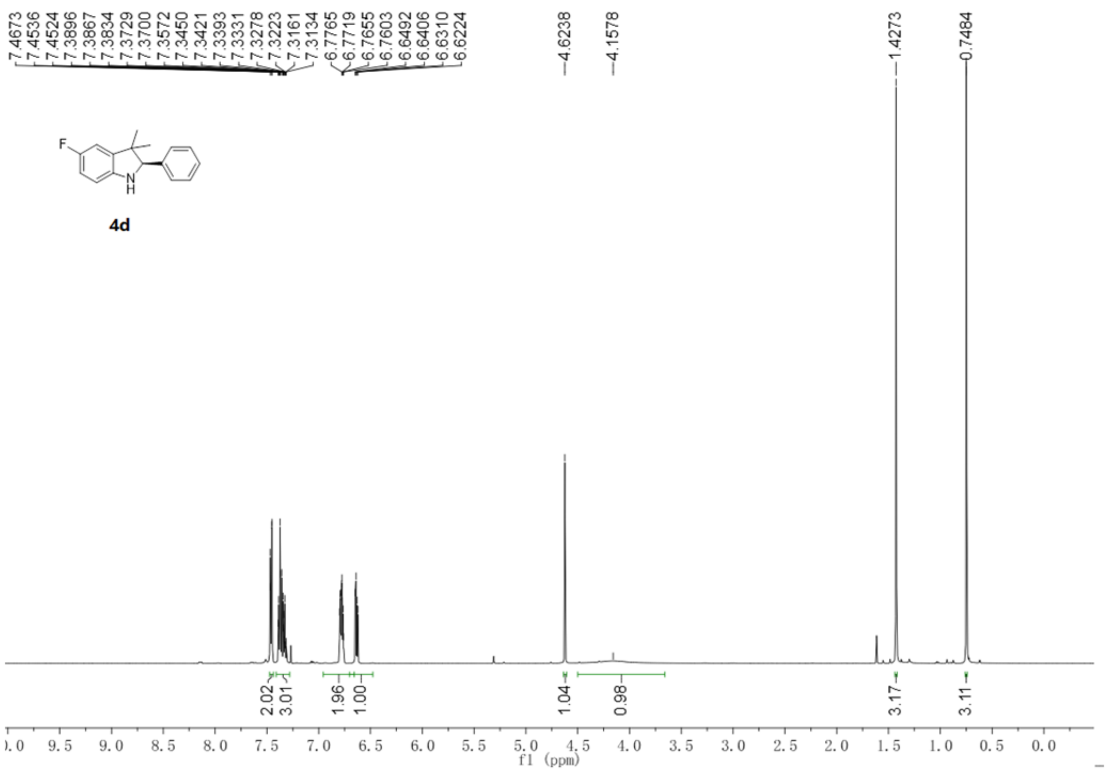

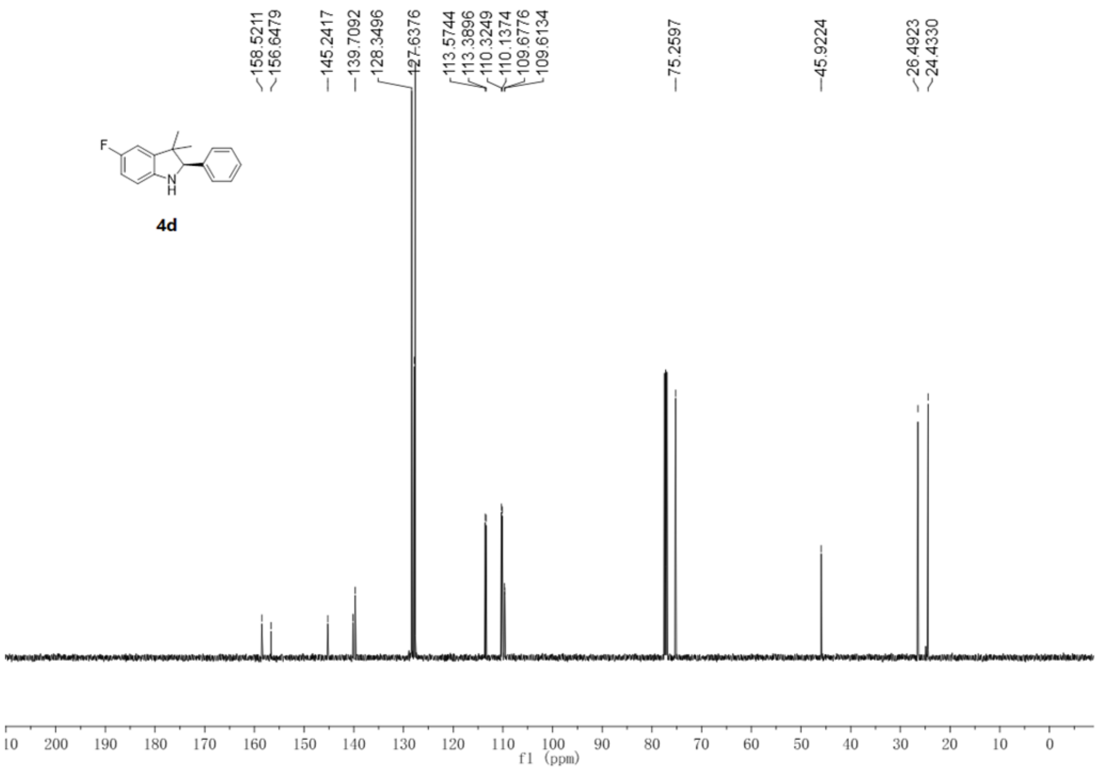

Supplementary figure 59 ^{1}H & ^{13}C NMR spectra of **4d**.

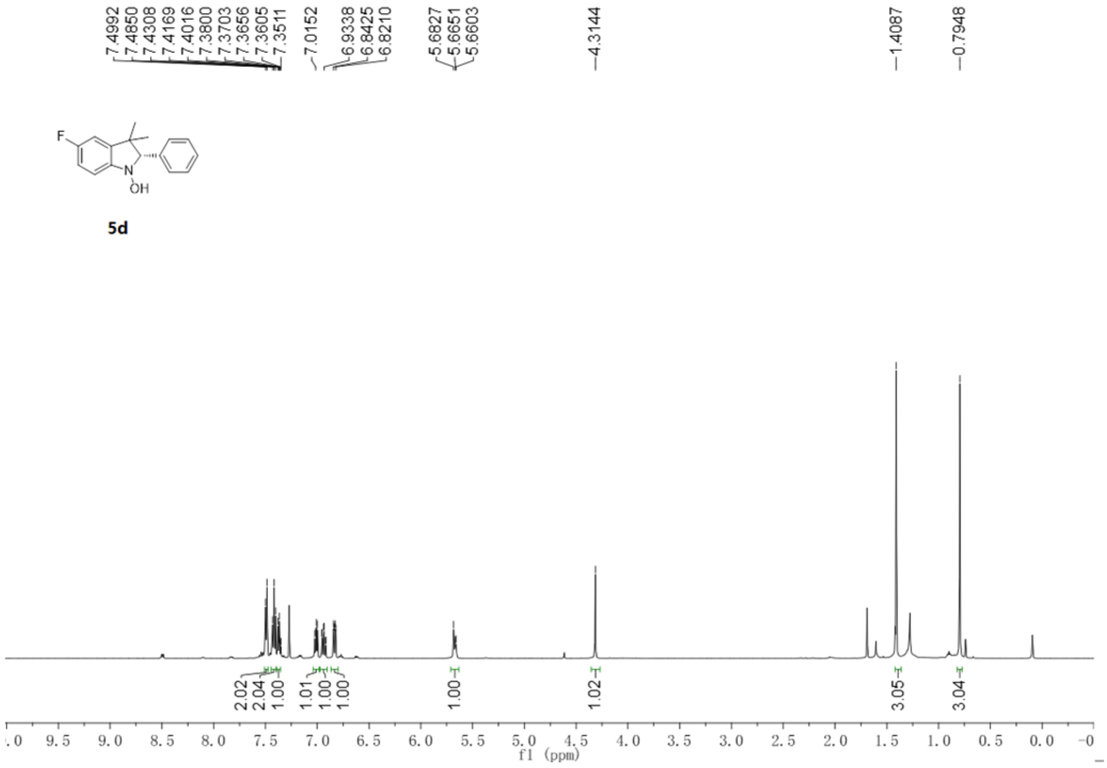

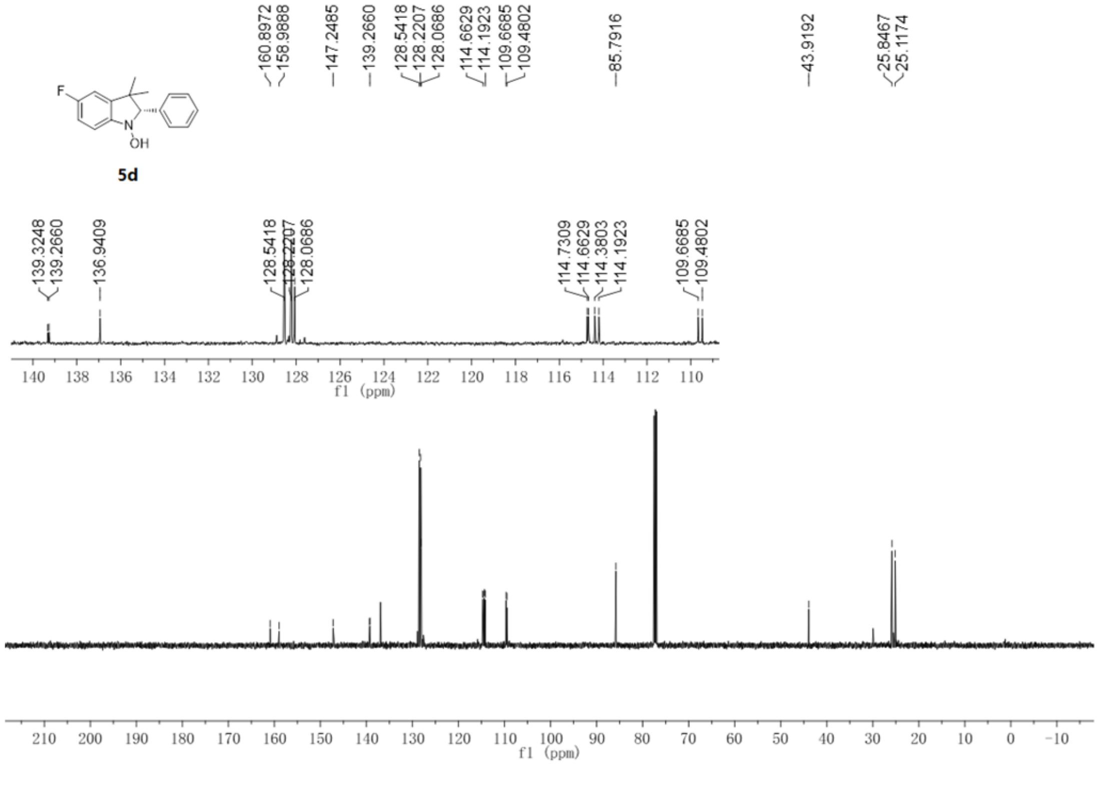

Supplementary figure 60 ^{1}H & ^{13}C NMR spectra of **5d**.

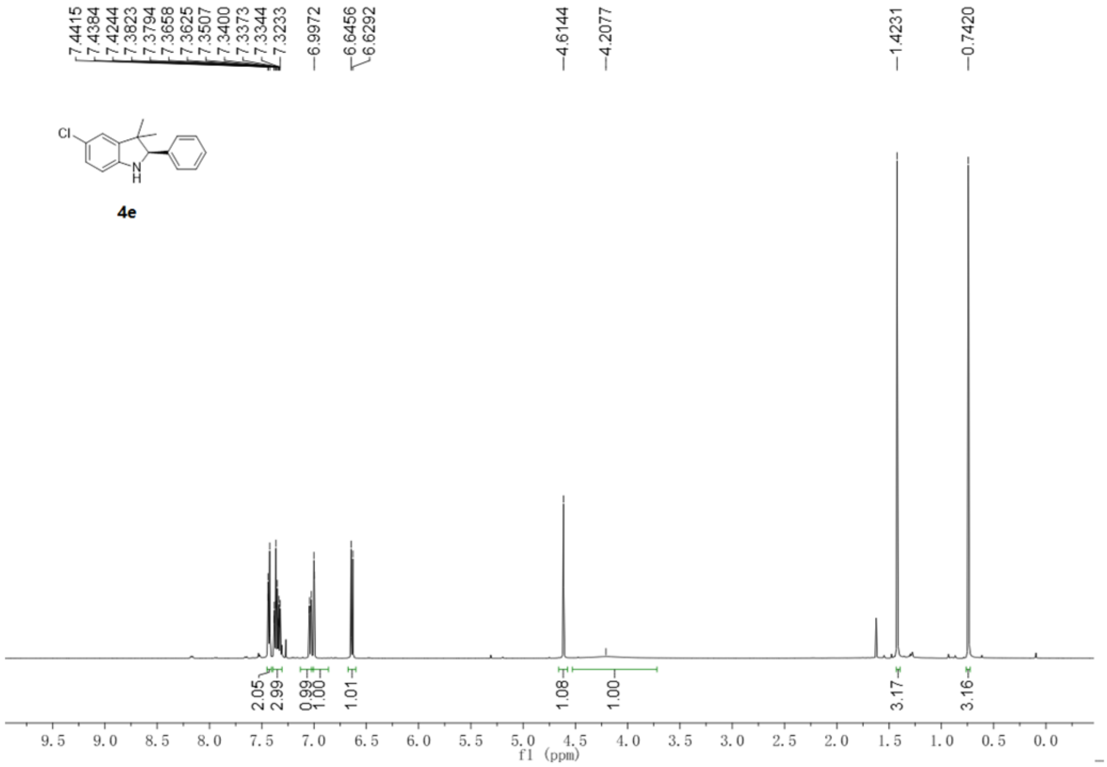

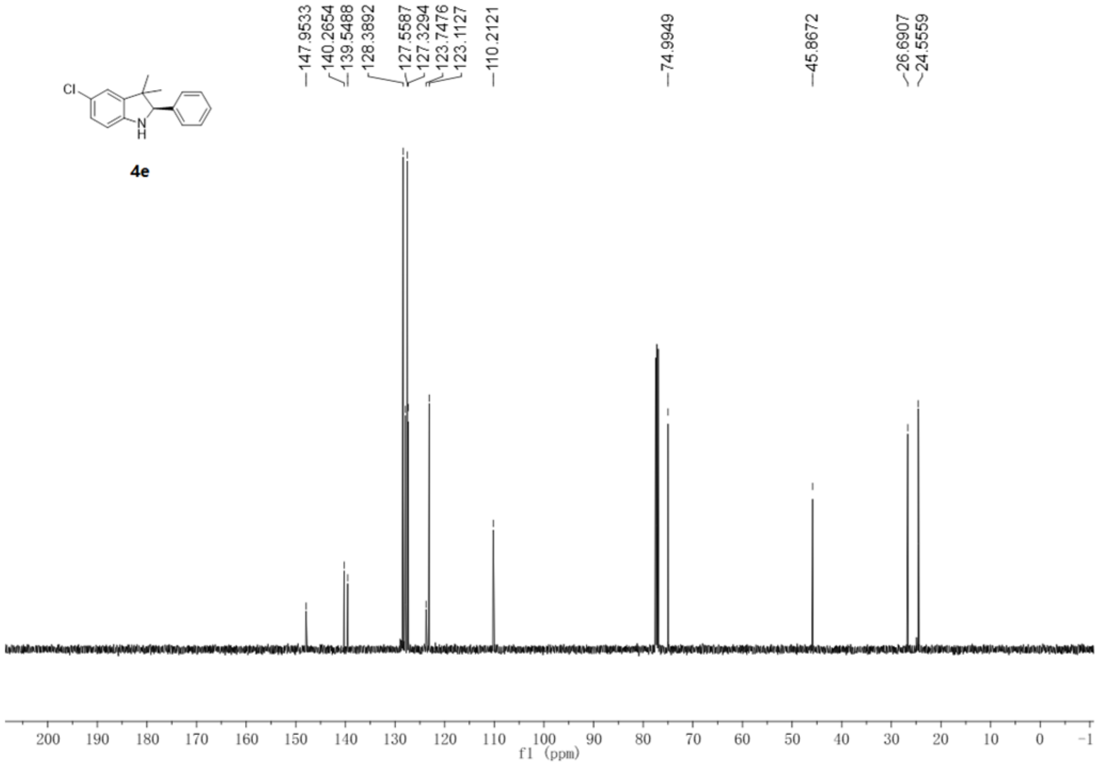

Supplementary figure 61 ^{1}H & ^{13}C NMR spectra of **4e**.

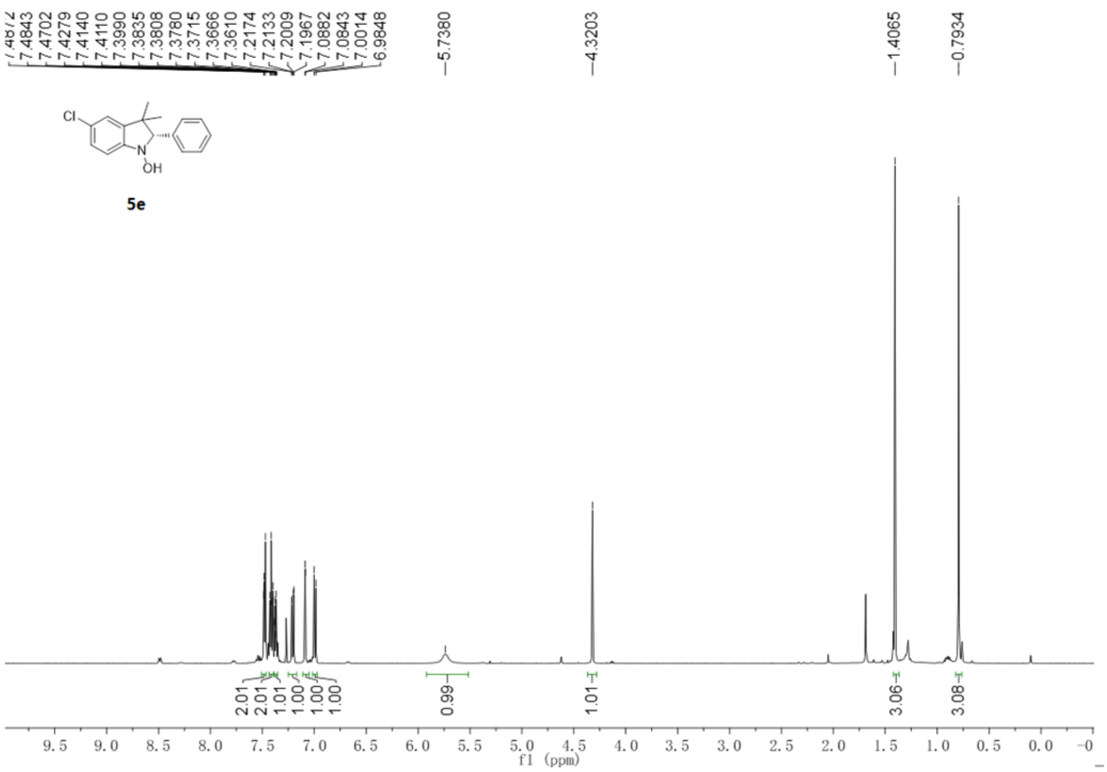

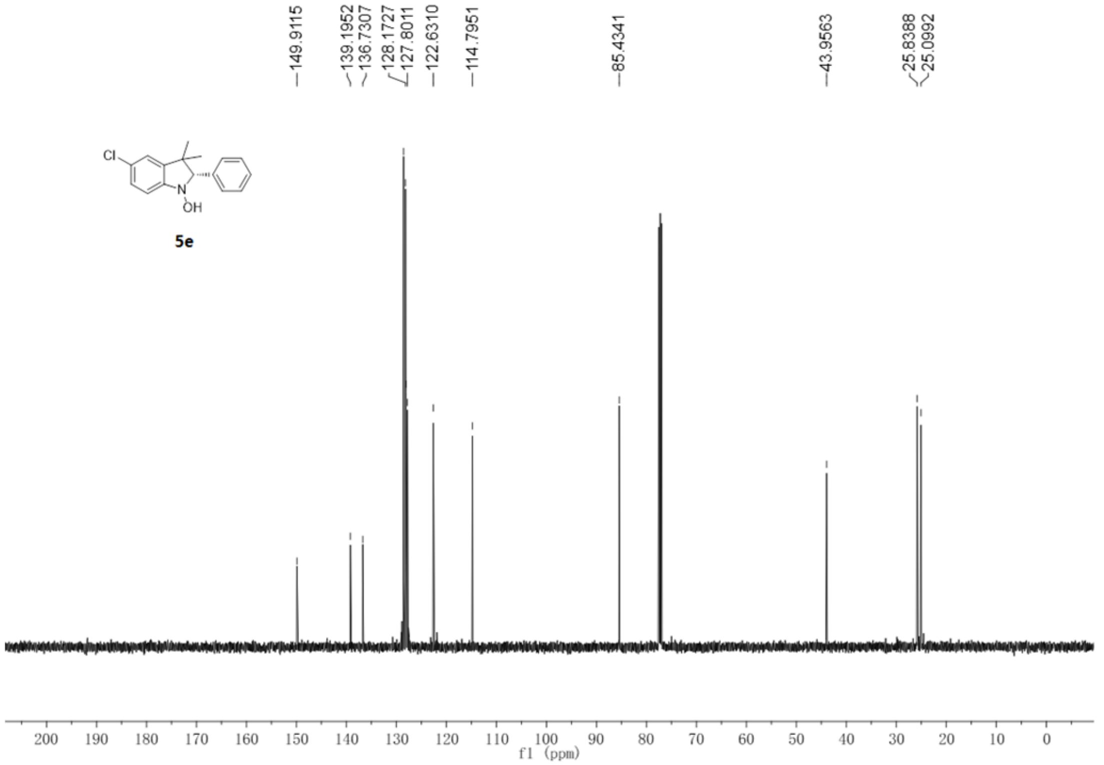

Supplementary figure 62 ^{1}H & ^{13}C NMR spectra of **5e**.

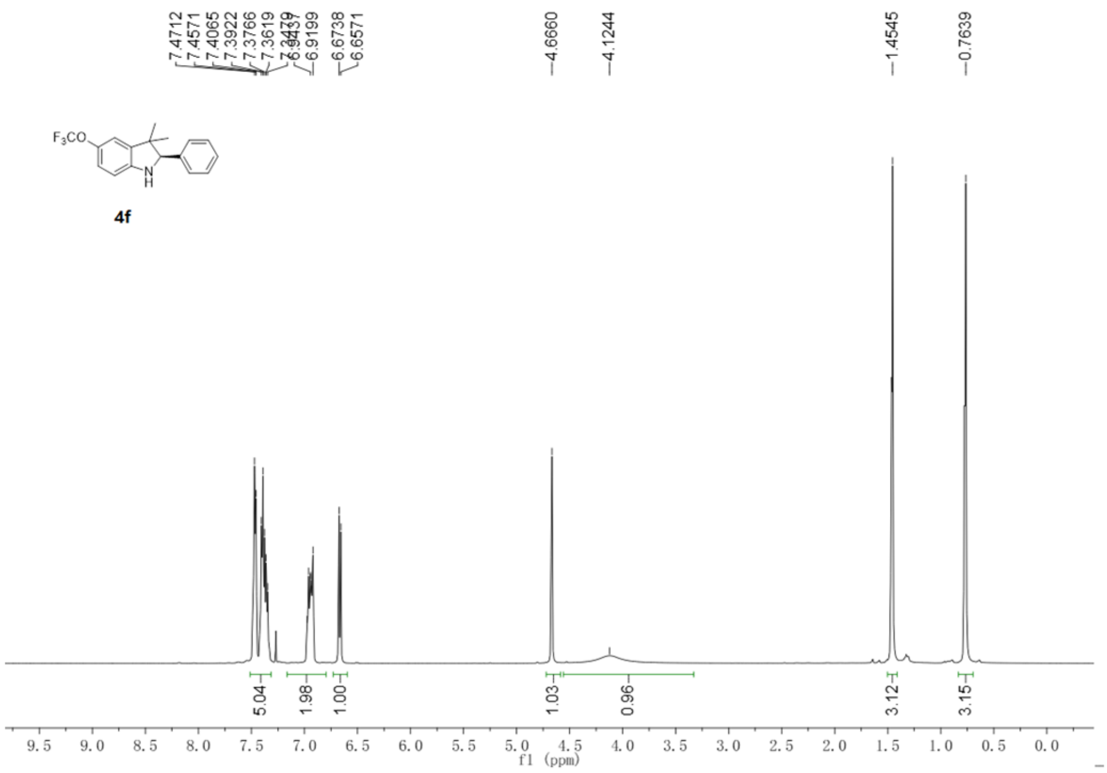

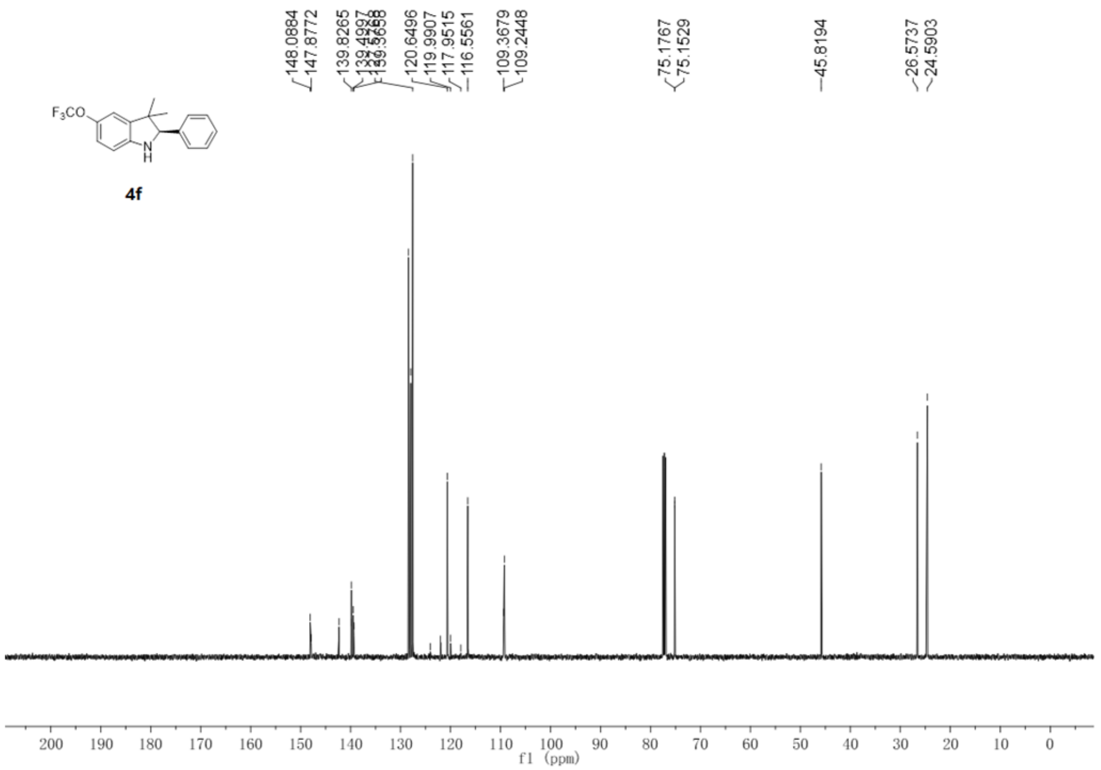

Supplementary figure 63^{1}H & ^{13}C NMR spectra of **4f**.

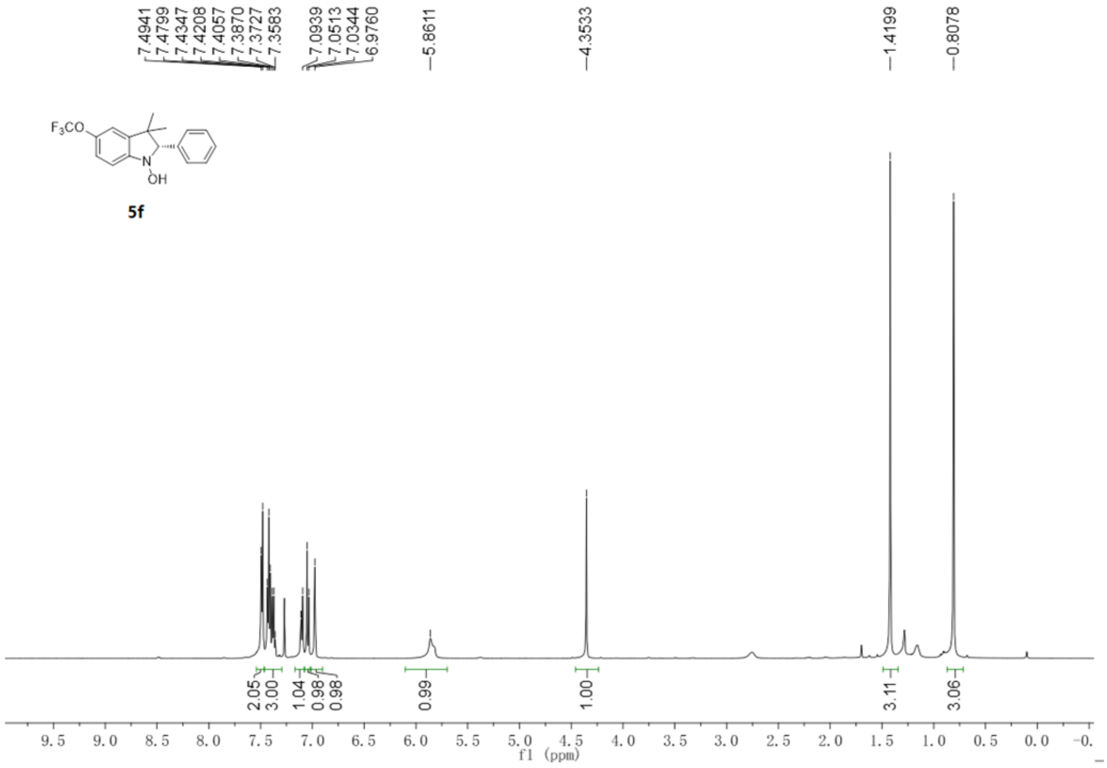

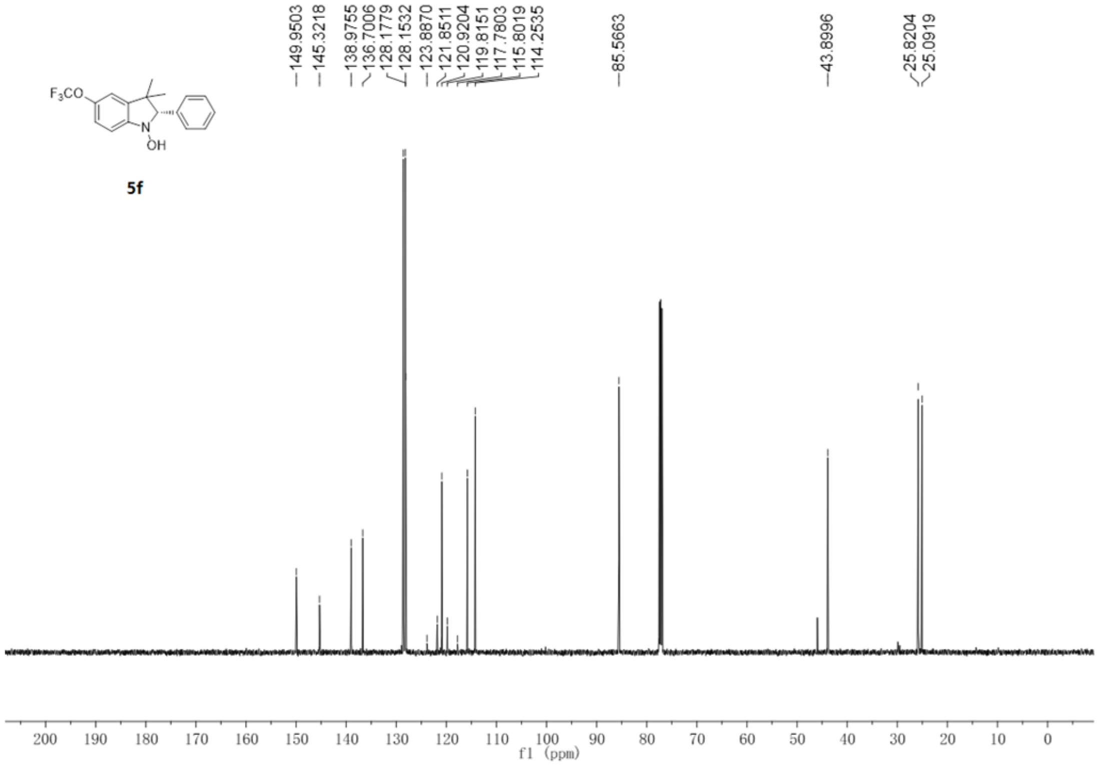

Supplementary figure 64 ^{1}H & ^{13}C NMR spectra of **5f**.

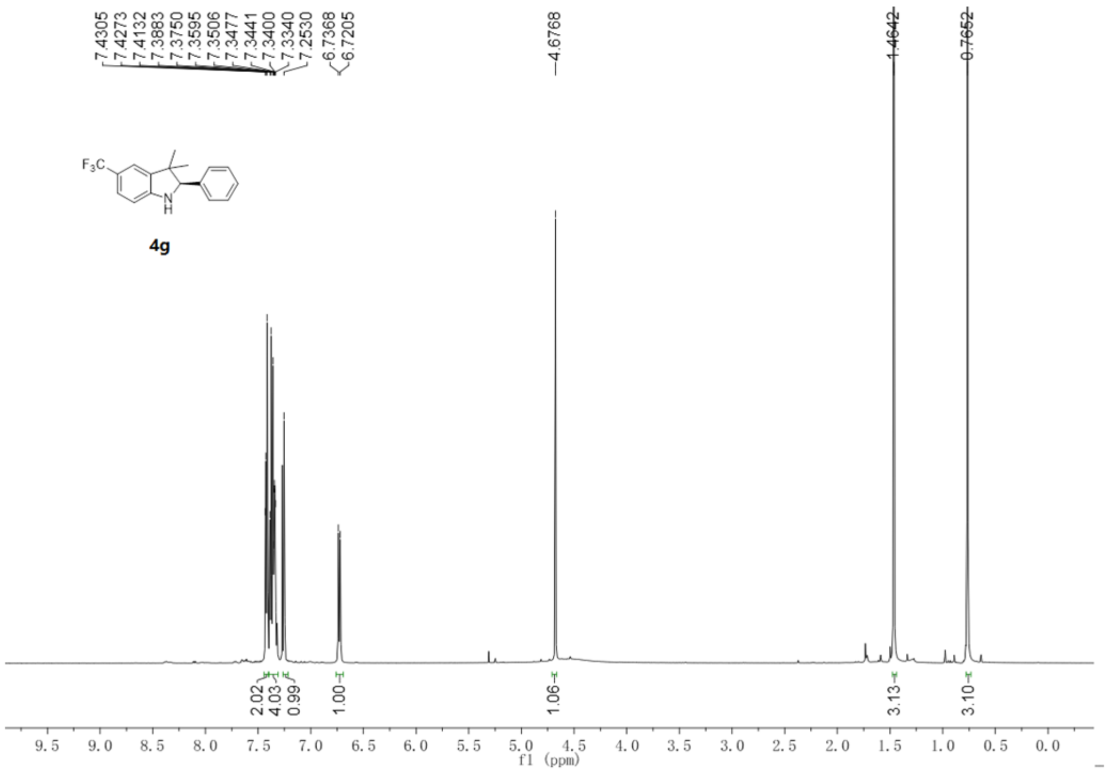

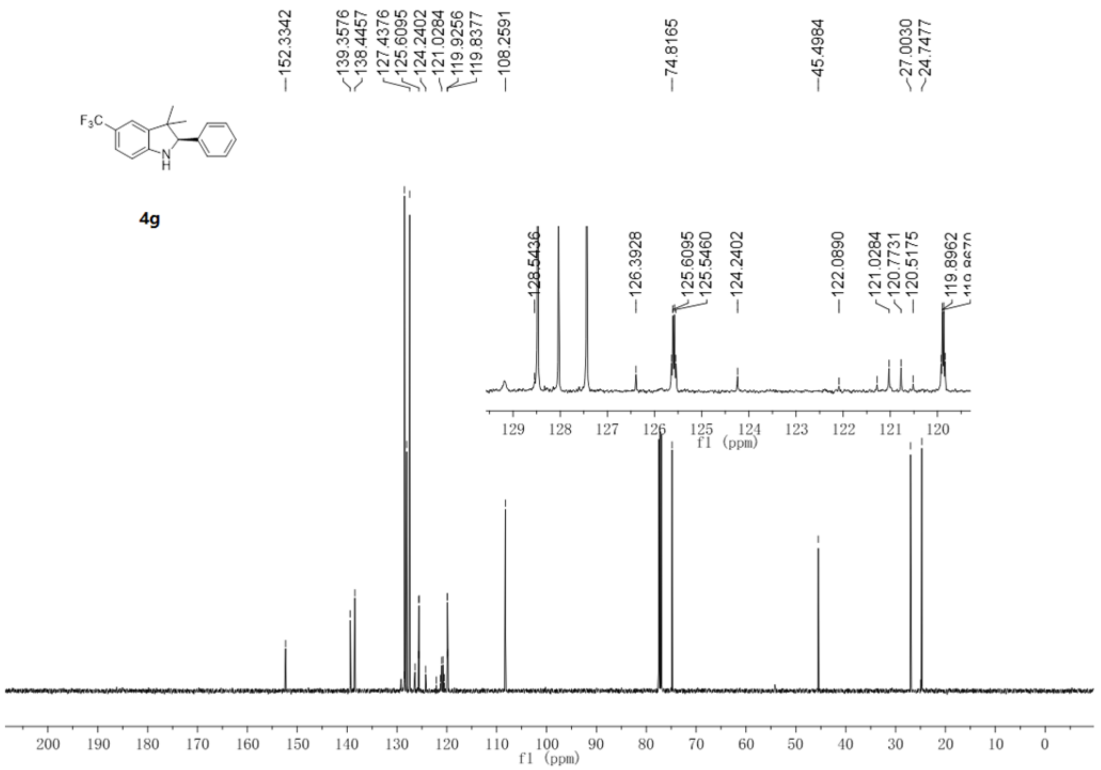

Supplementary figure 65^{1}H & ^{13}C NMR spectra of **4g**.

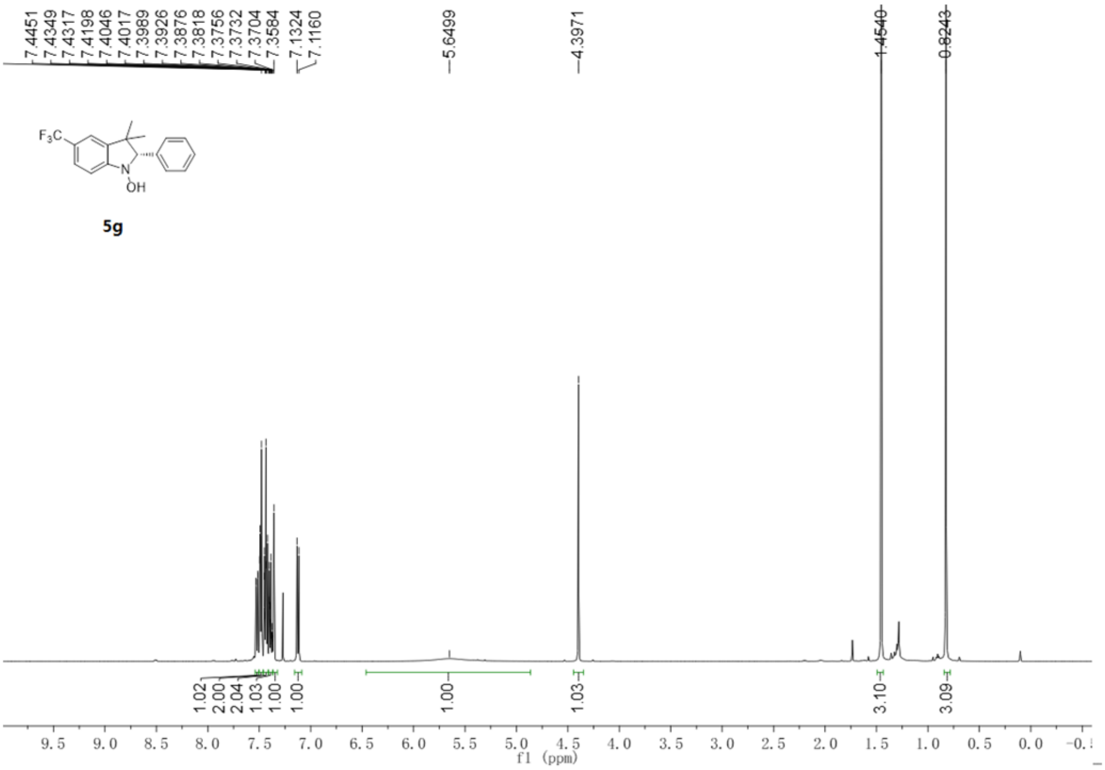

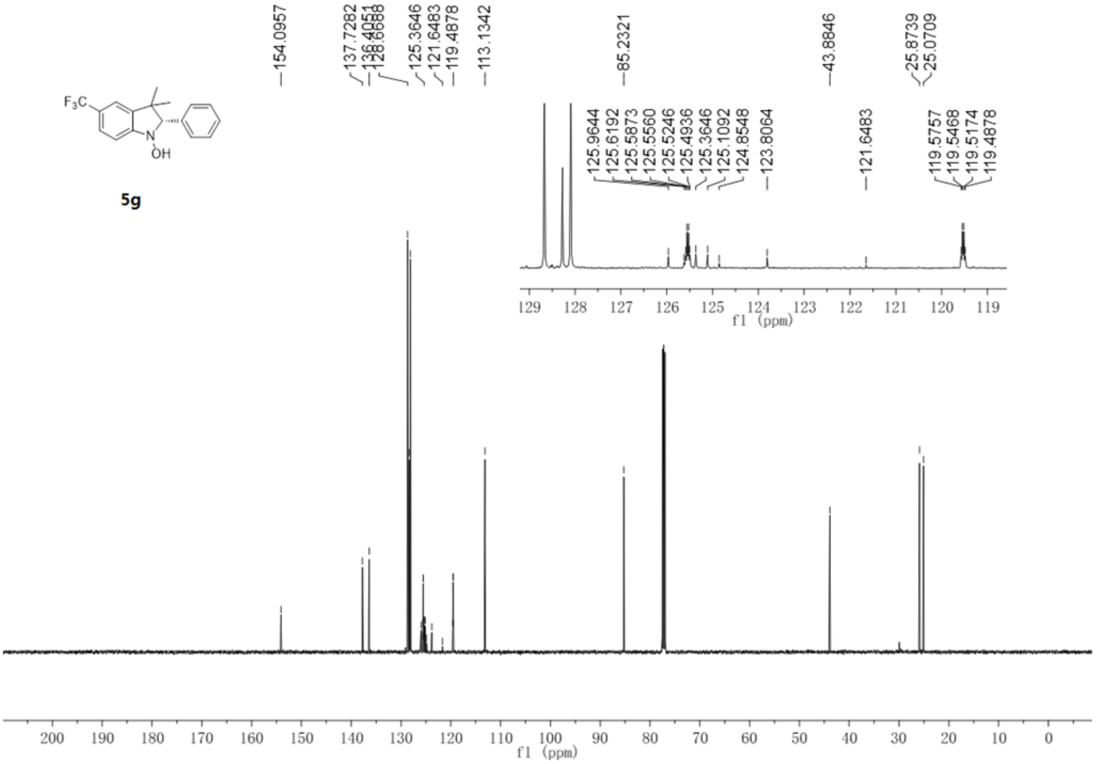

Supplementary figure 66 ^{1}H & ^{13}C NMR spectra of **5g**.

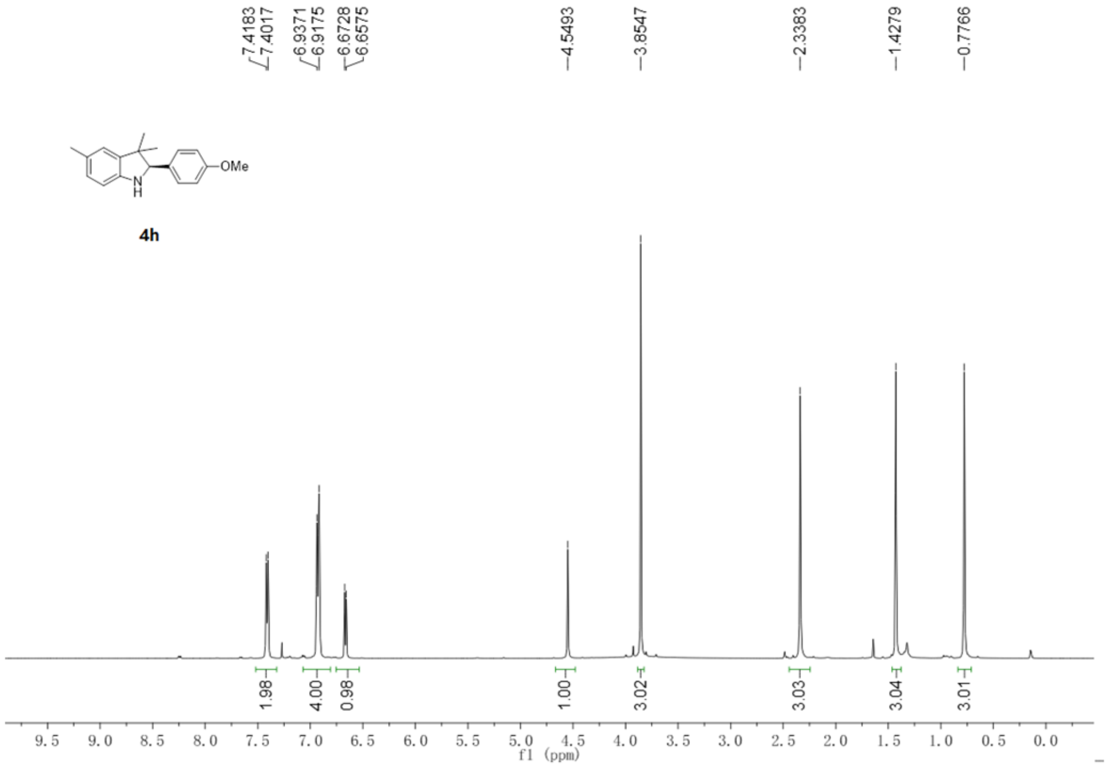

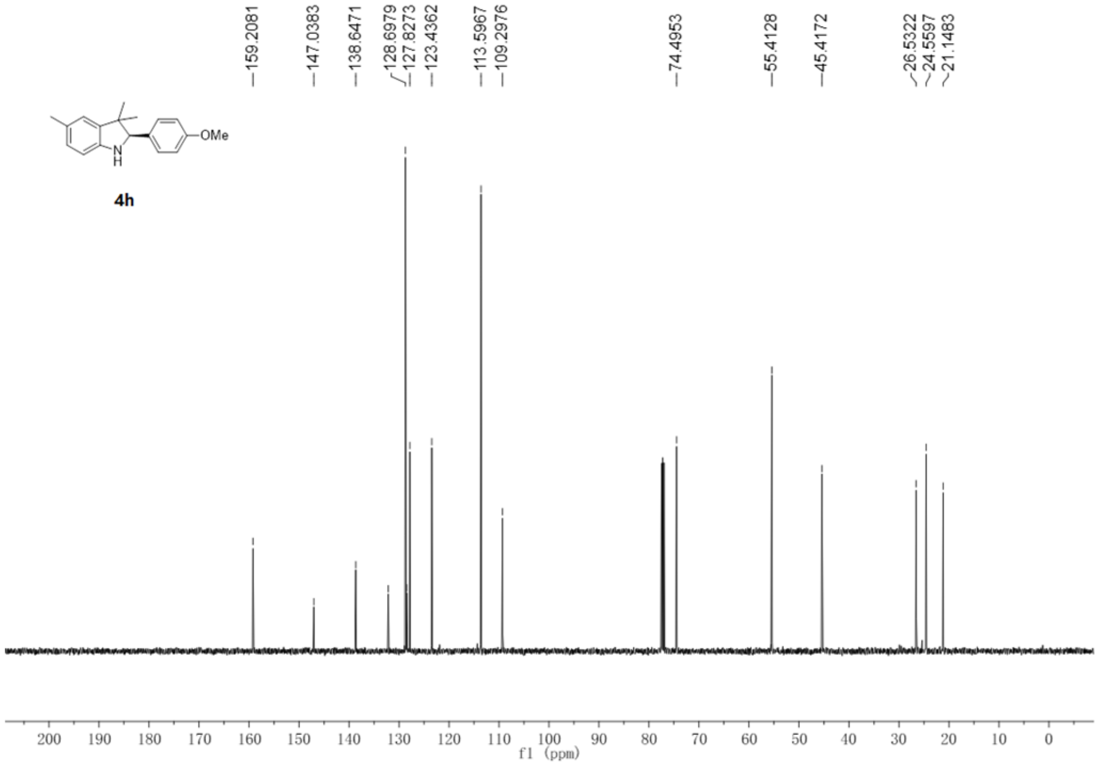

Supplementary figure 67 ^{1}H & ^{13}C NMR spectra of **4h**.

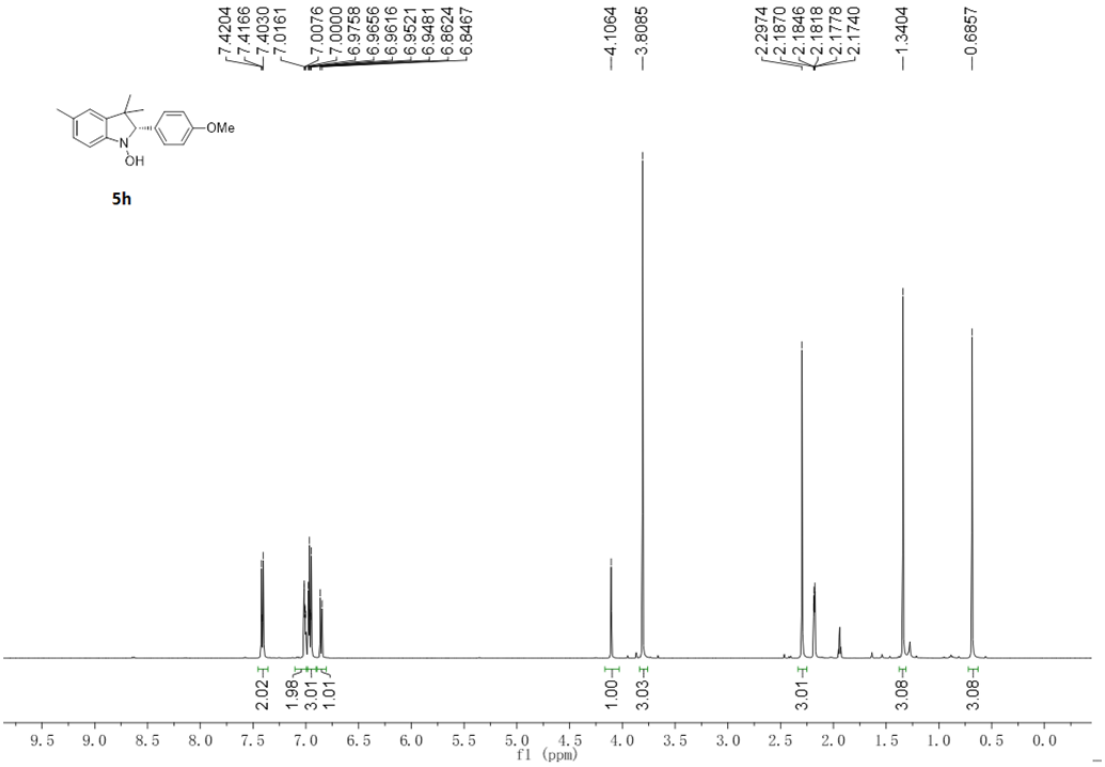

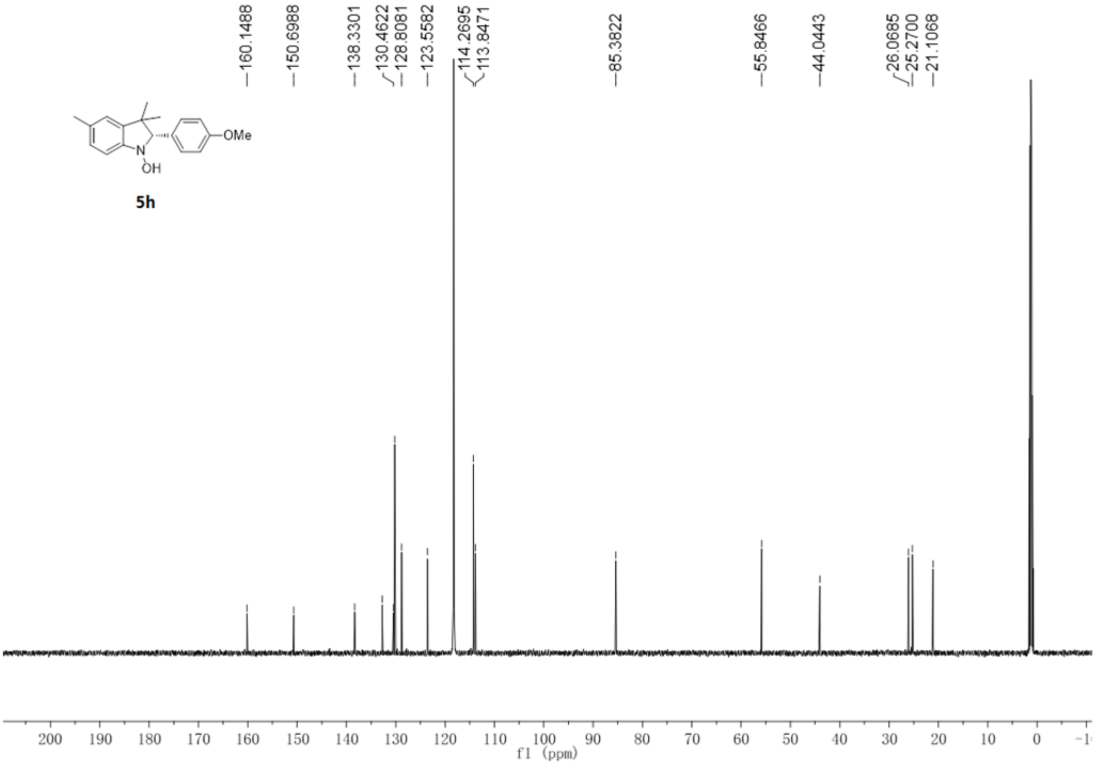

Supplementary figure 68 ^{1}H & ^{13}C NMR spectra of **5h**.

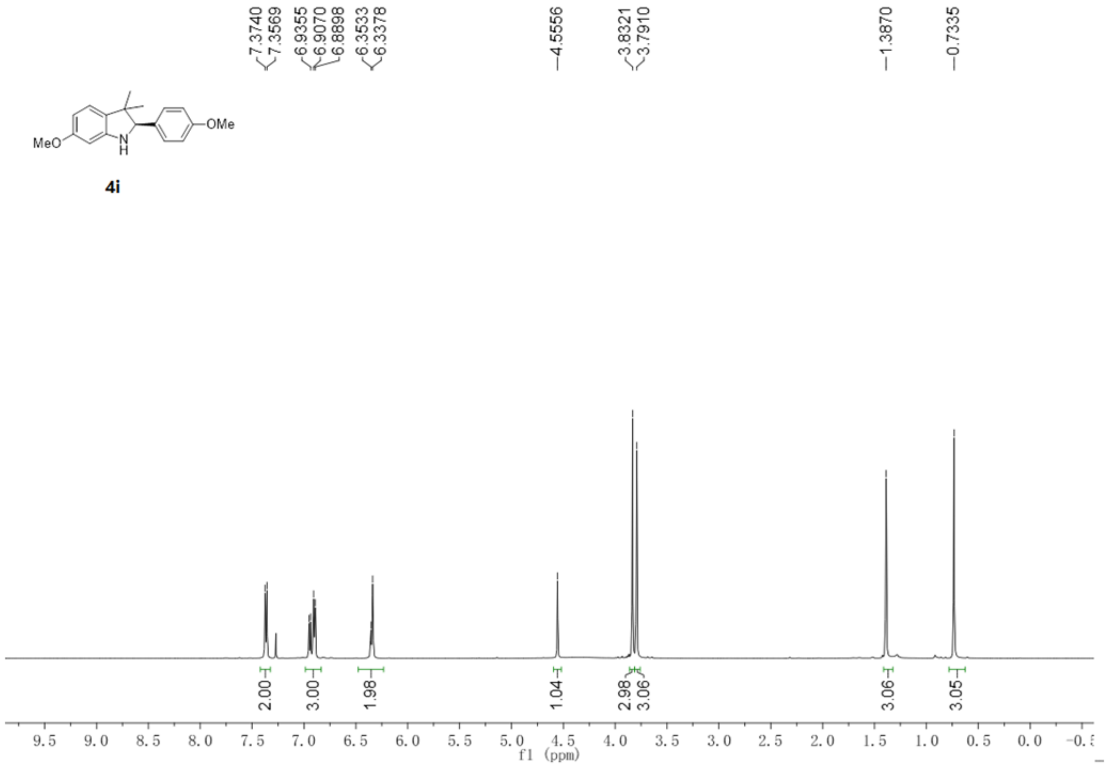

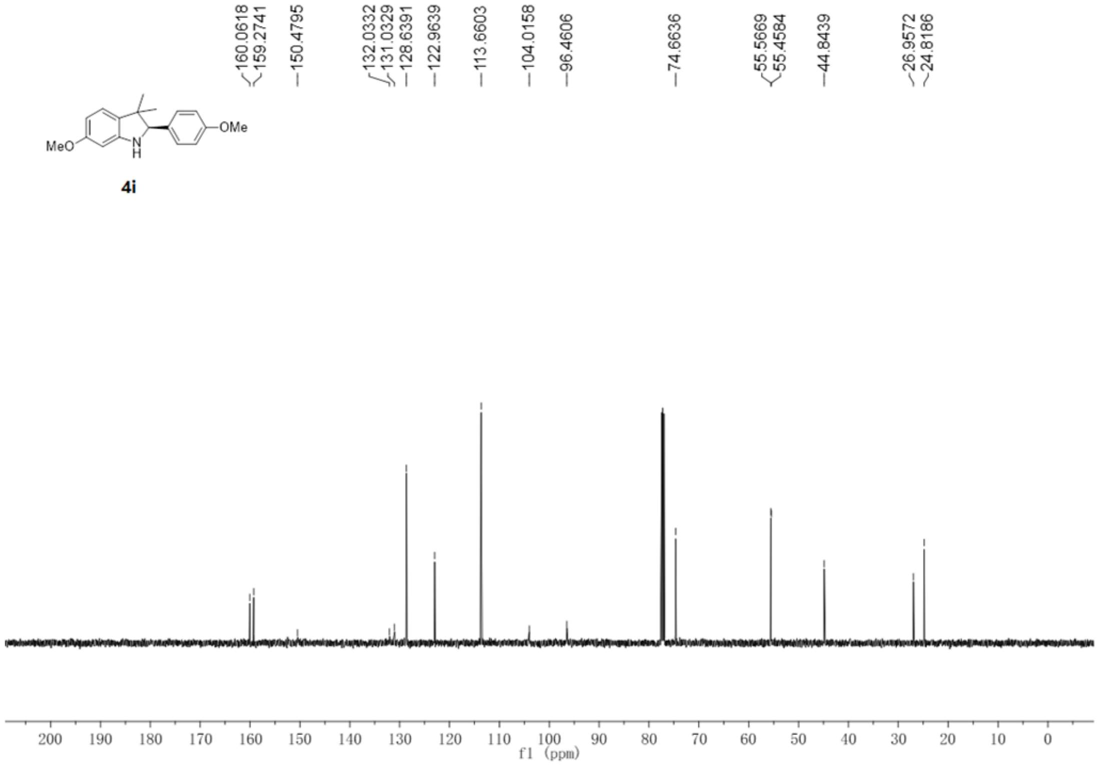

Supplementary figure 69 ^{1}H & ^{13}C NMR spectra of **4i**.

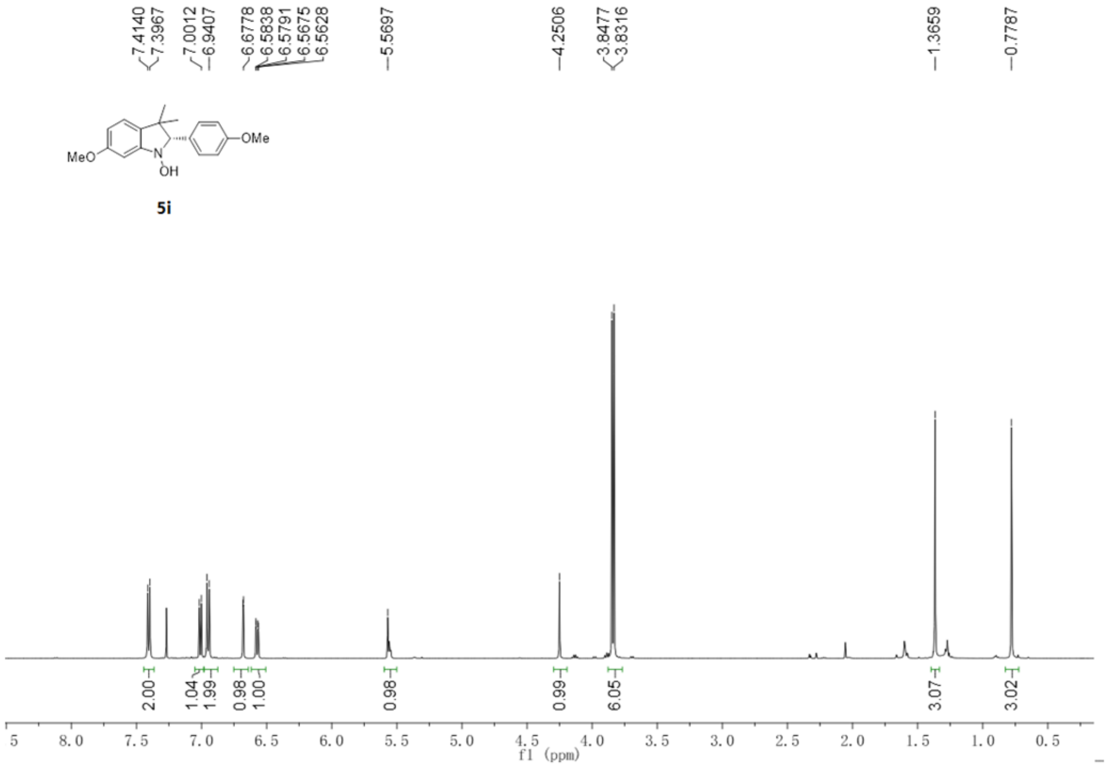

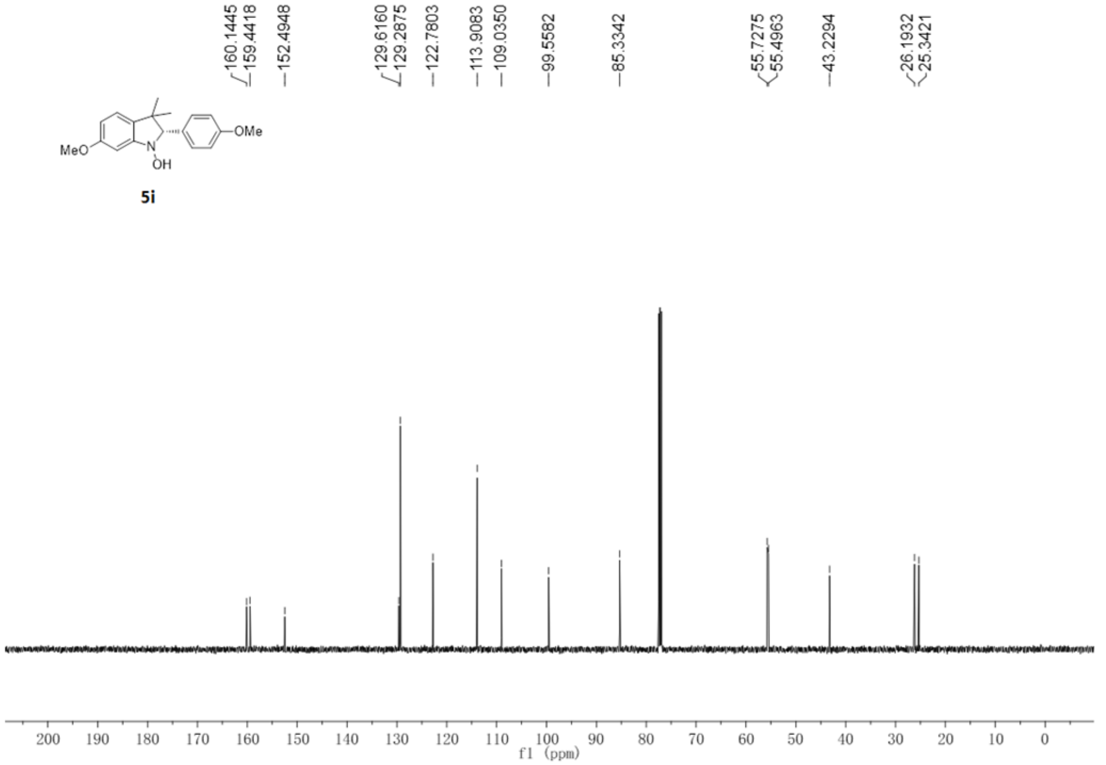

Supplementary figure 70 ^{1}H & ^{13}C NMR spectra of **5i**.

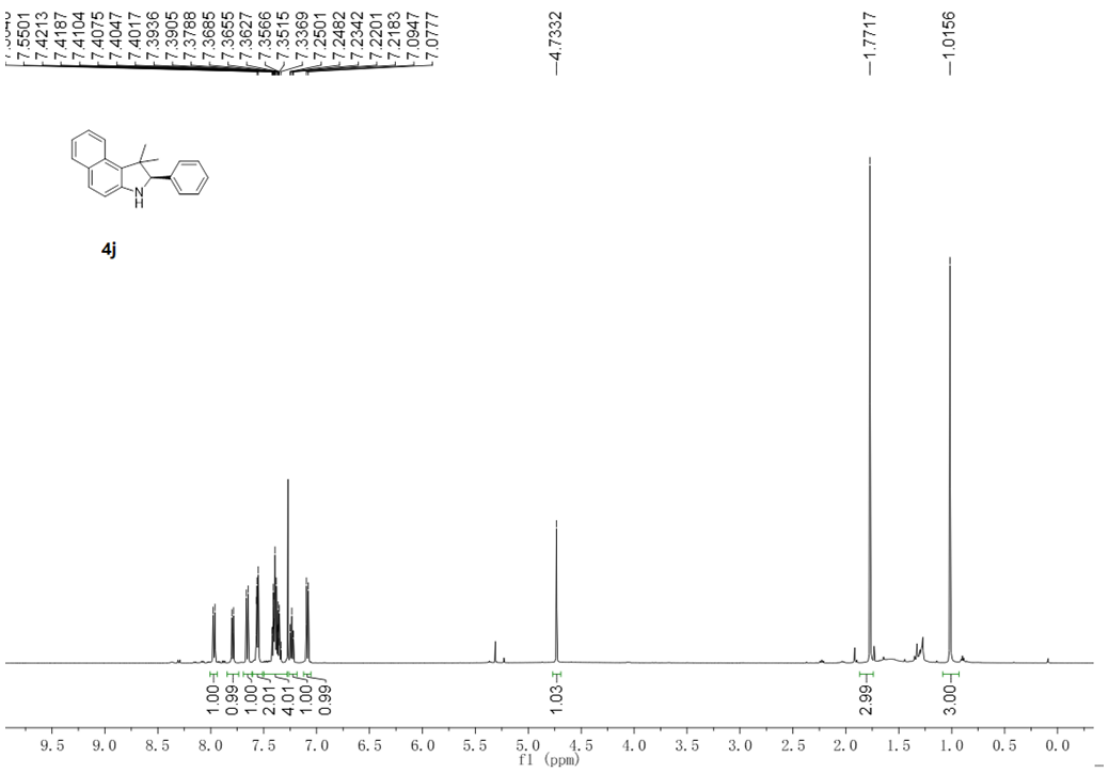

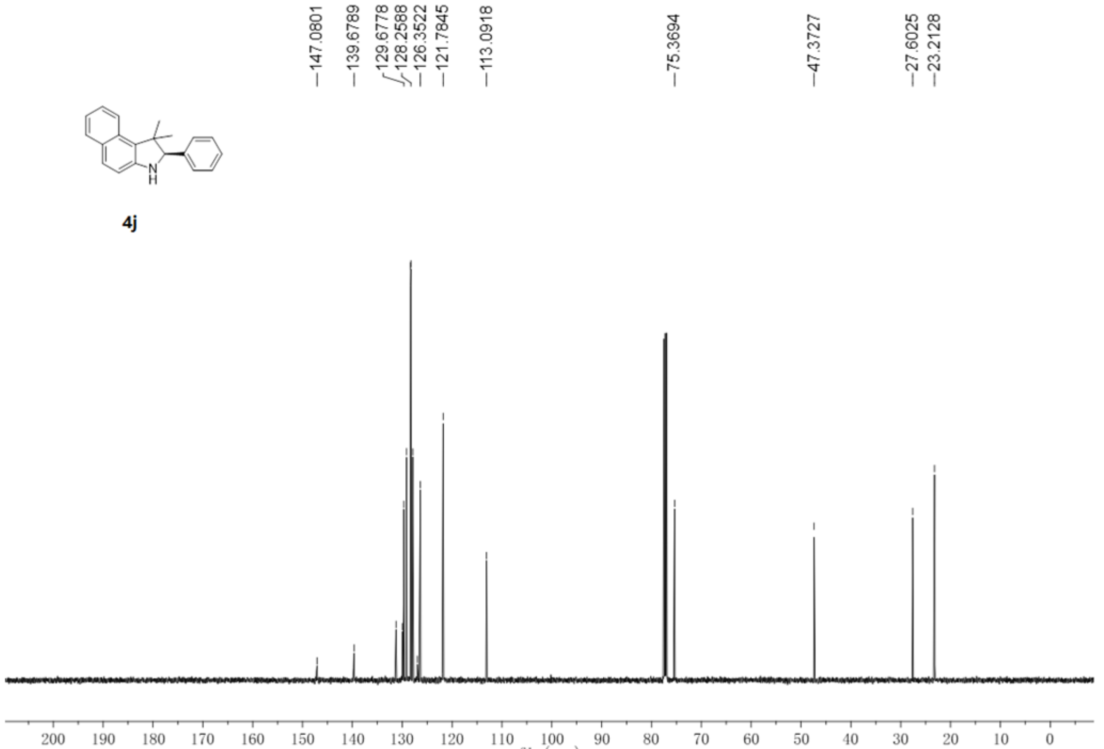

Supplementary figure 71^{1}H & ^{13}C NMR spectra of **4j**.

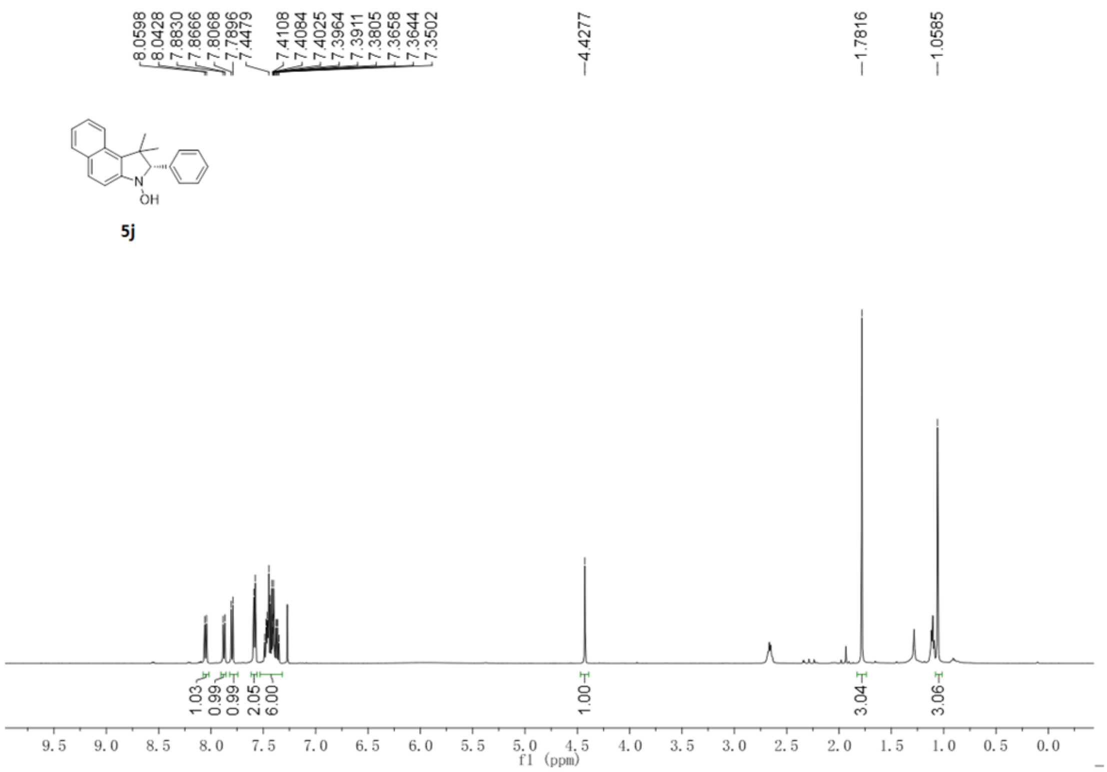

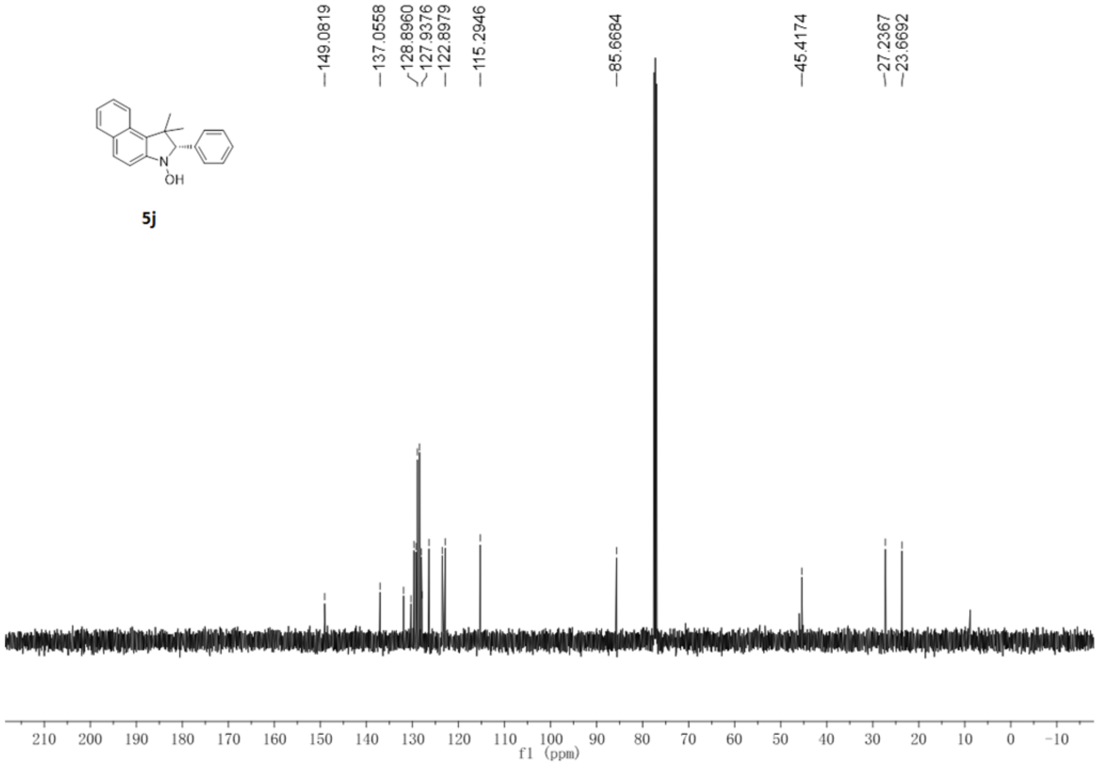

Supplementary figure 72^{1}H & ^{13}C NMR spectra of **5j**.

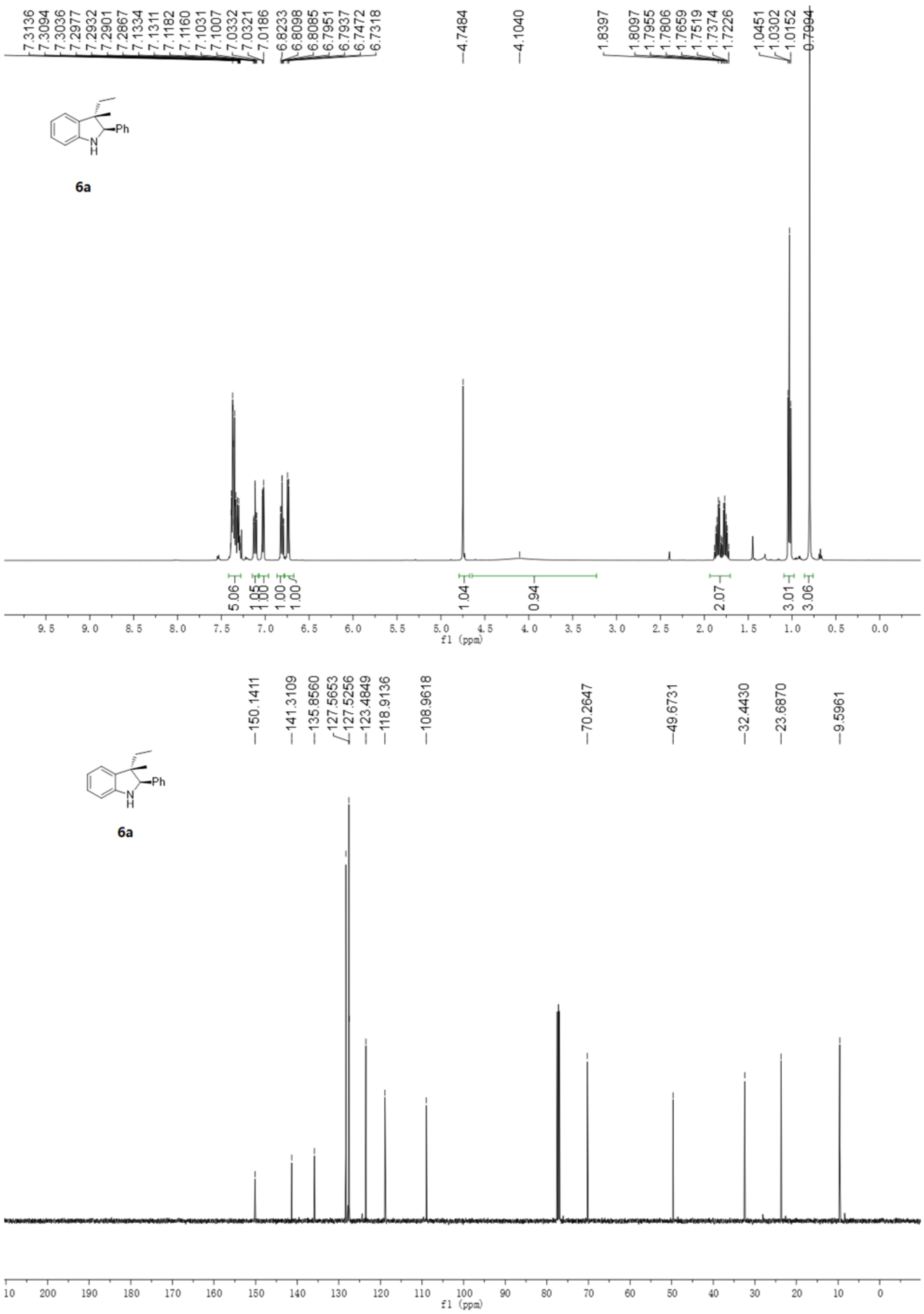

Supplementary figure 73 ^{1}H & ^{13}C NMR spectra of **6a**.

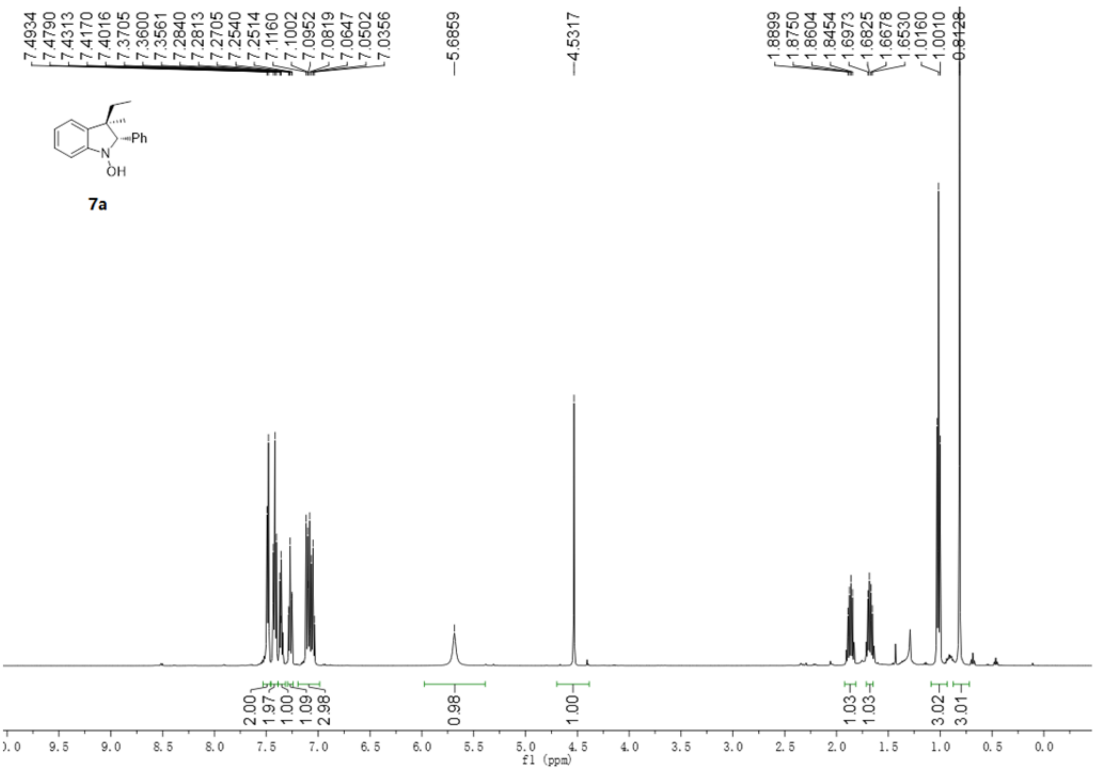

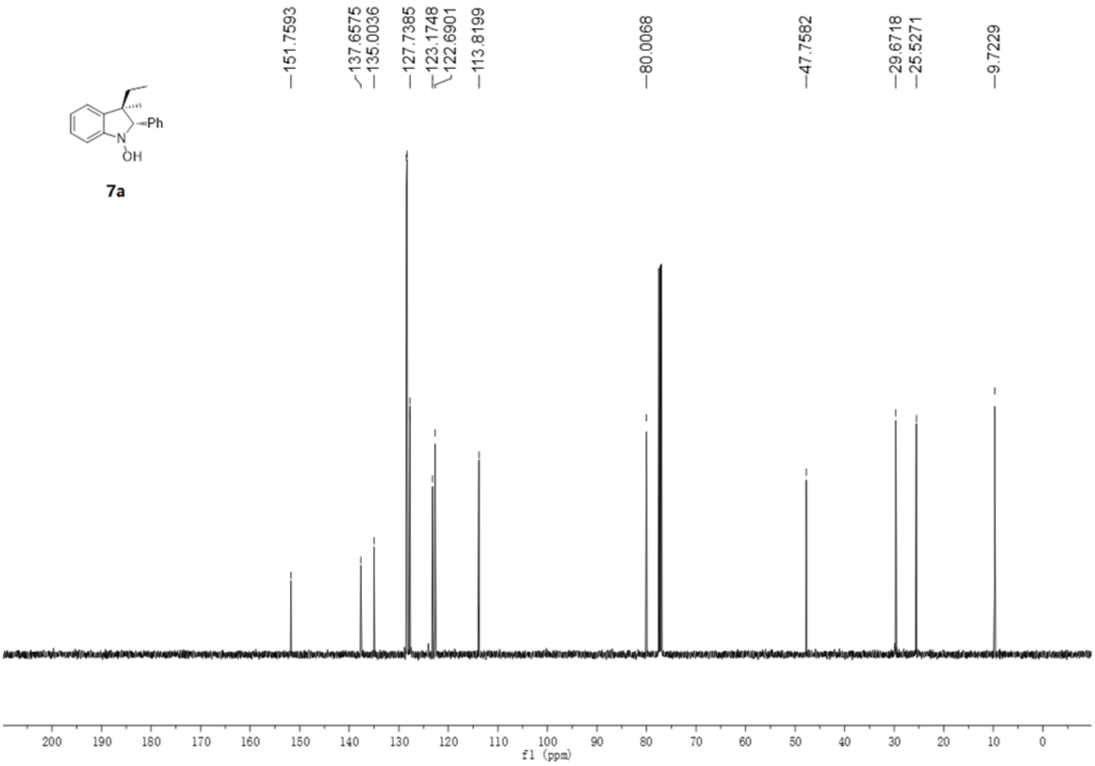

Supplementary figure 74 ^{1}H & ^{13}C NMR spectra of **7a**.

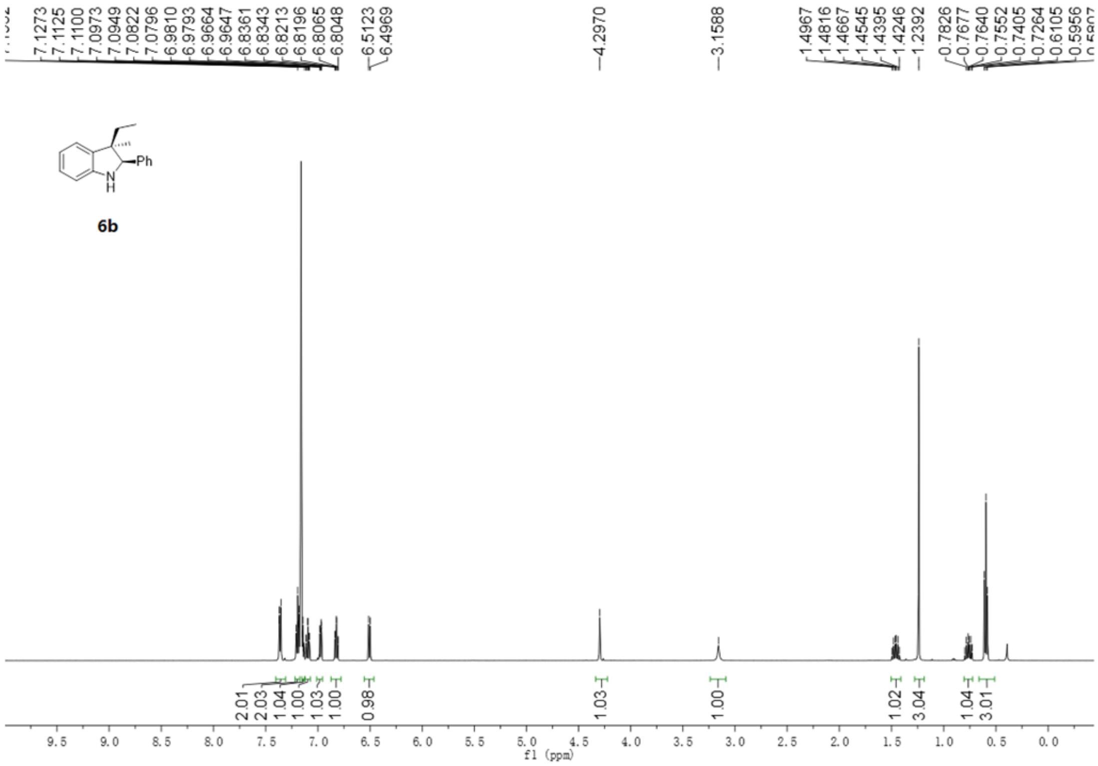

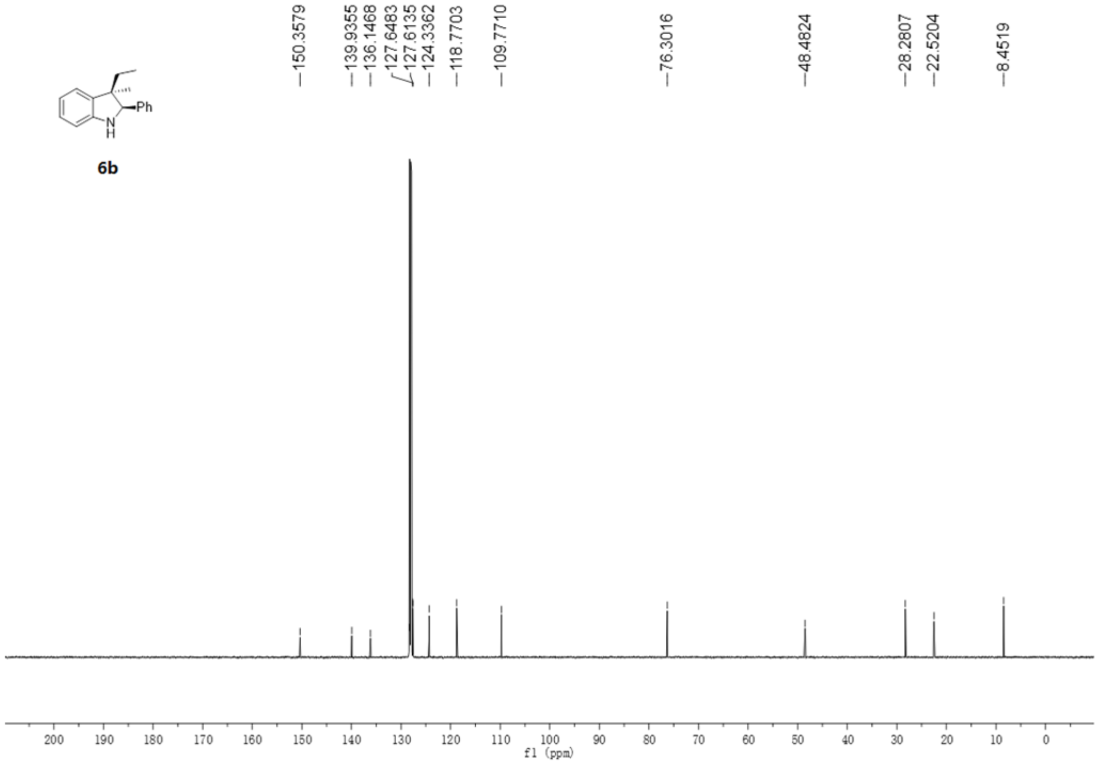

Supplementary figure 75 ^{1}H & ^{13}C NMR spectra of **6b**.

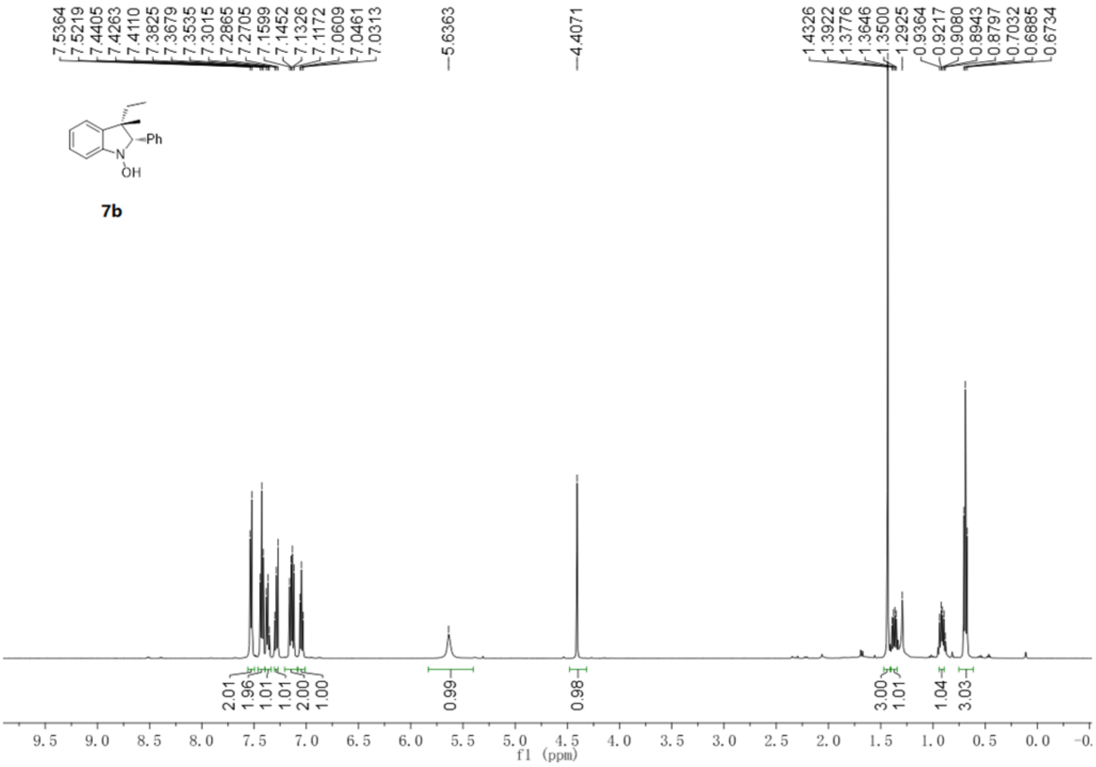

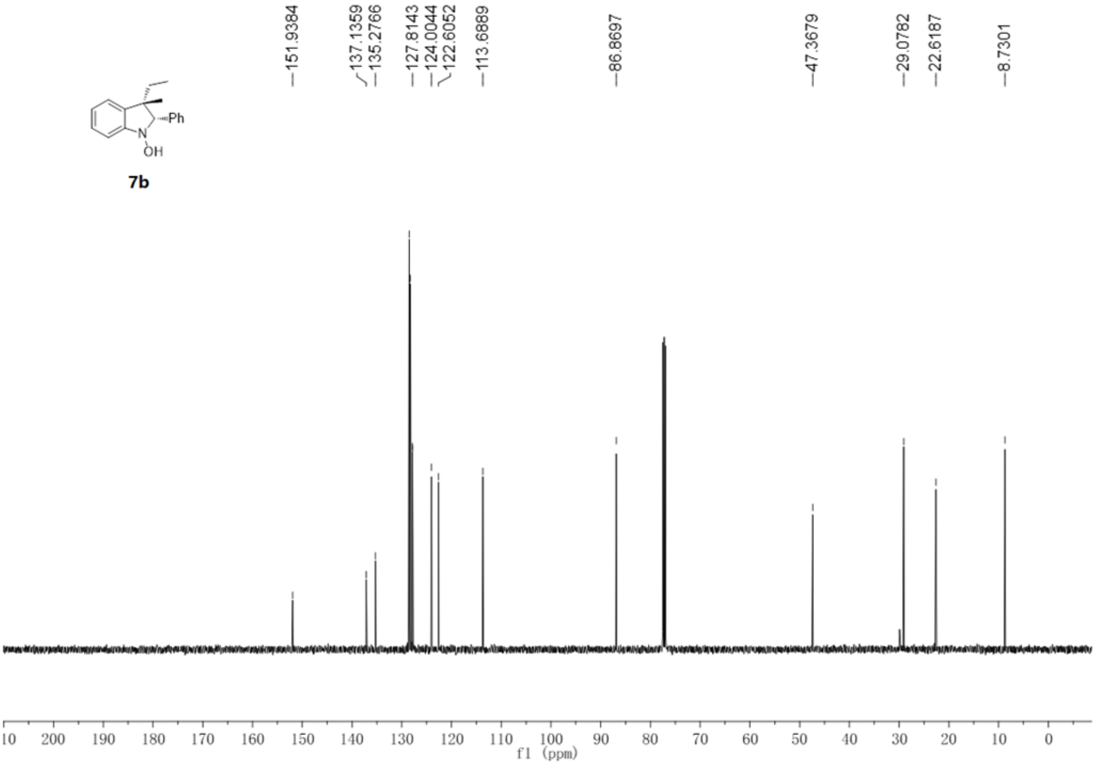

Supplementary figure 76 ^{1}H & ^{13}C NMR spectra of **7b**.

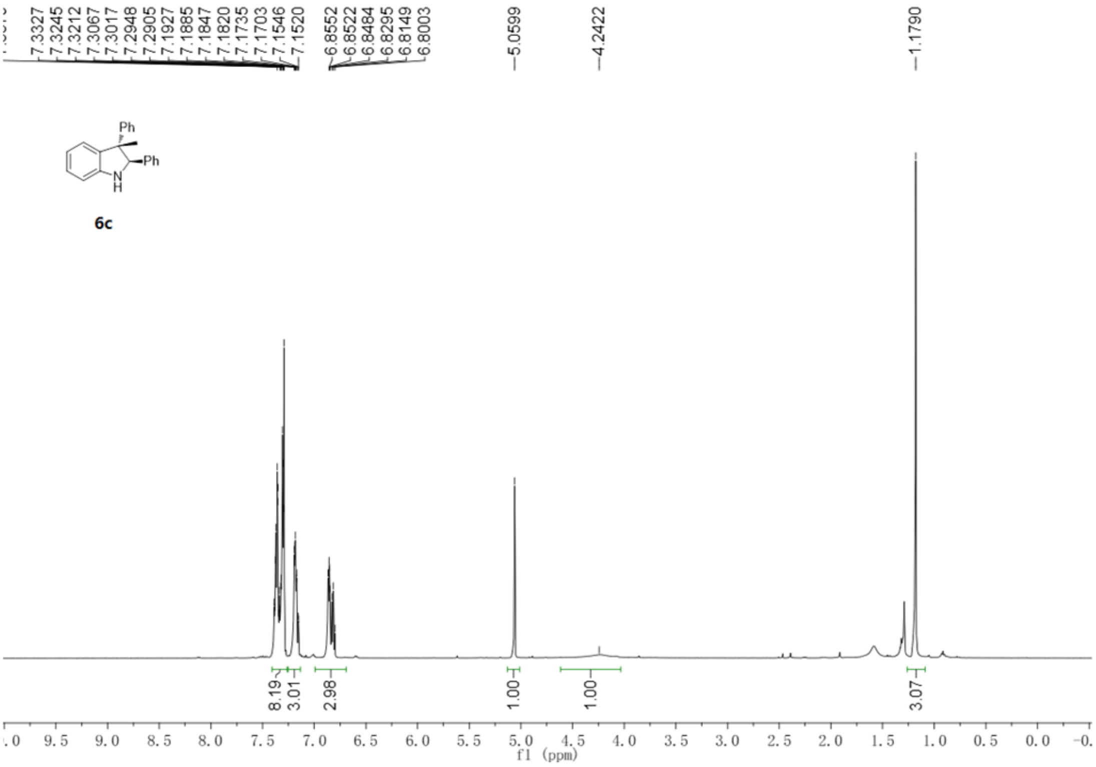

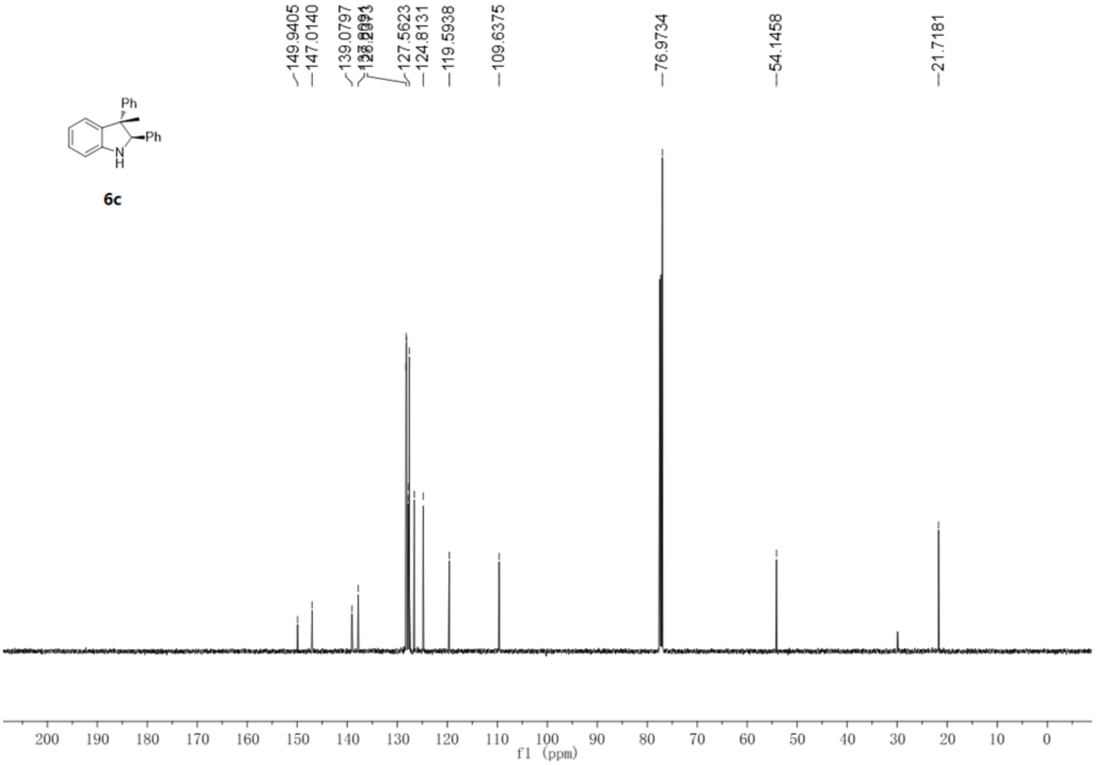

Supplementary figure 77 ^{1}H & ^{13}C NMR spectra of **6c**.

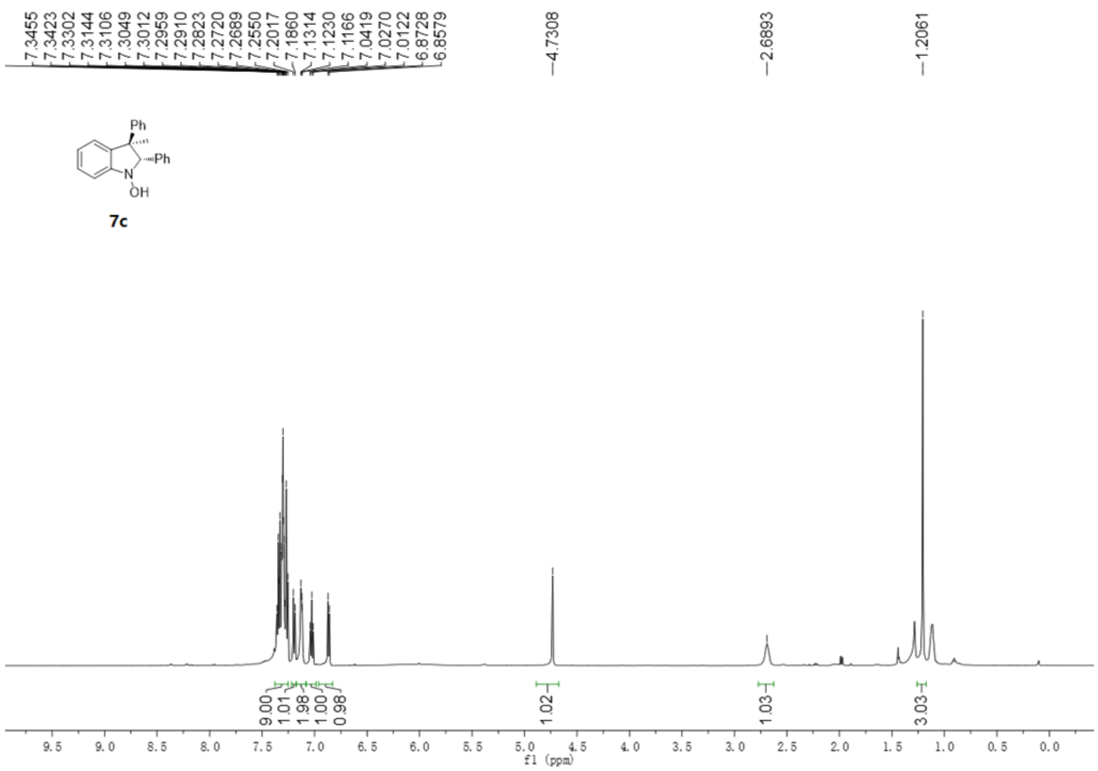

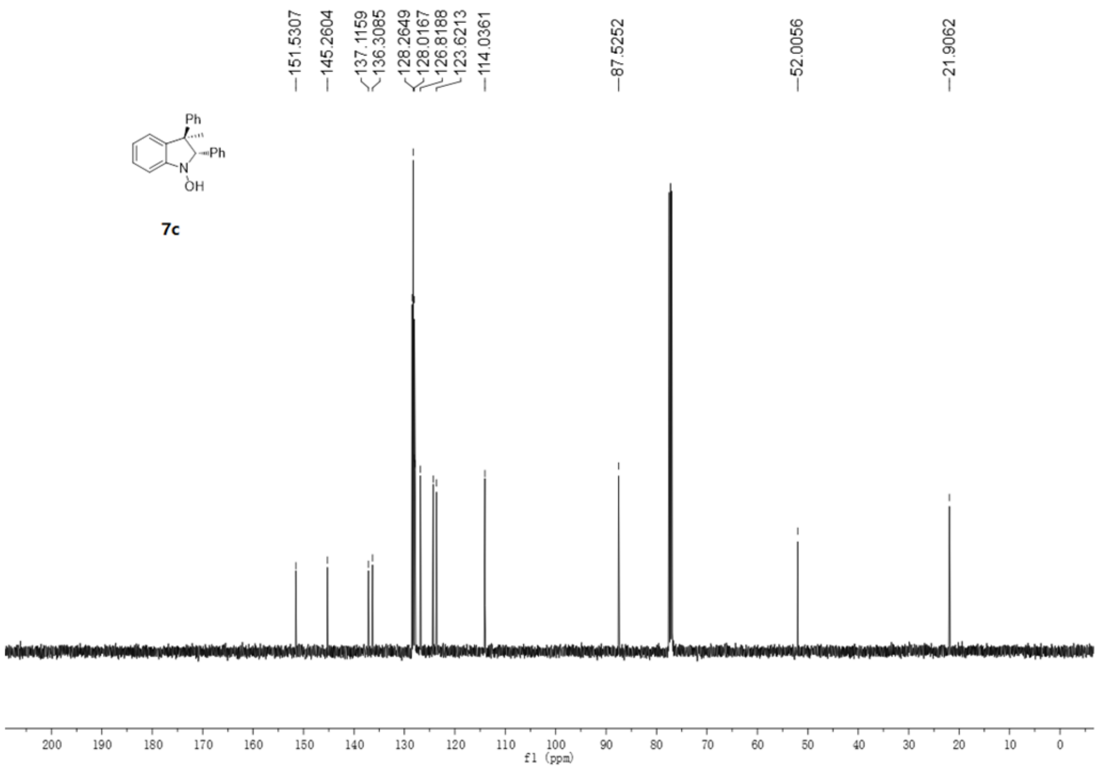

Supplementary figure 78 ^{1}H & ^{13}C NMR spectra of **7c**.

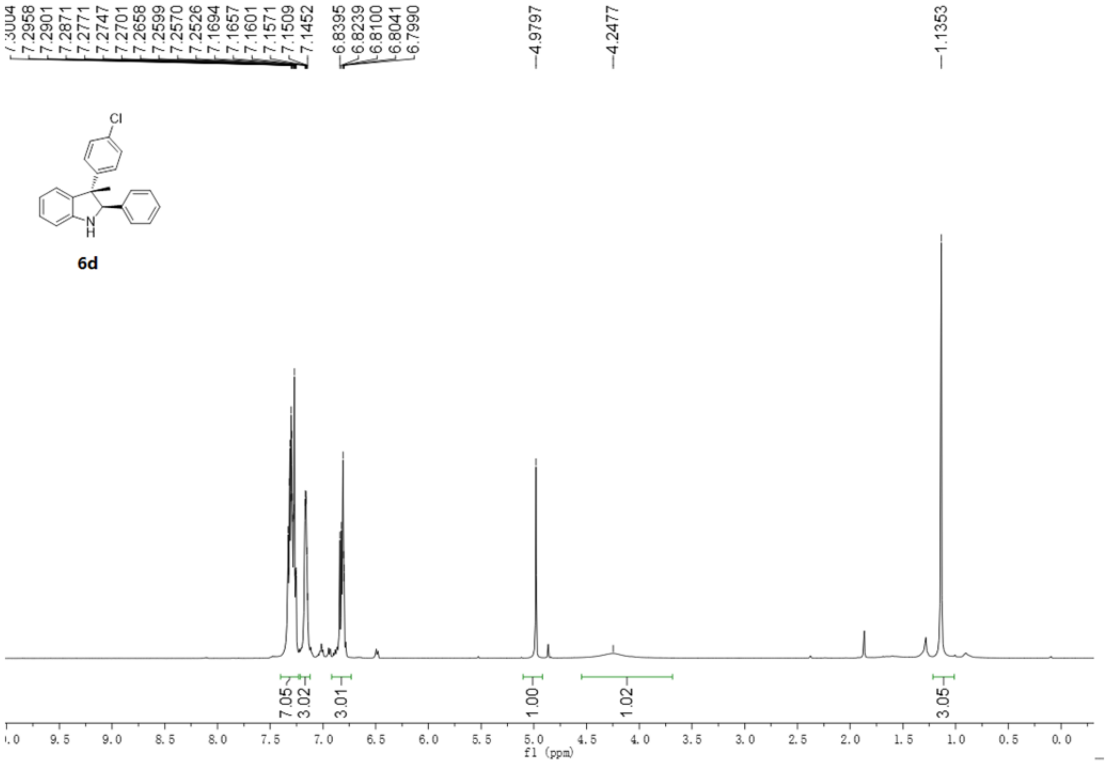

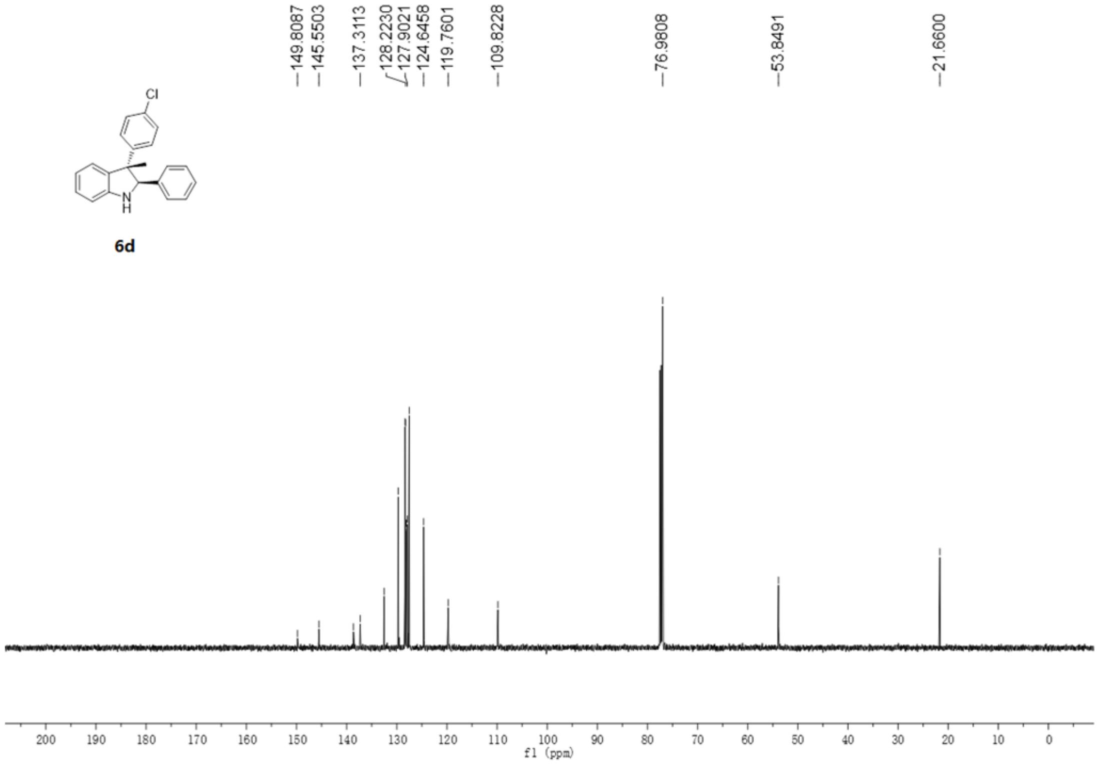

Supplementary figure 79 ^{1}H & ^{13}C NMR spectra of **6d**.

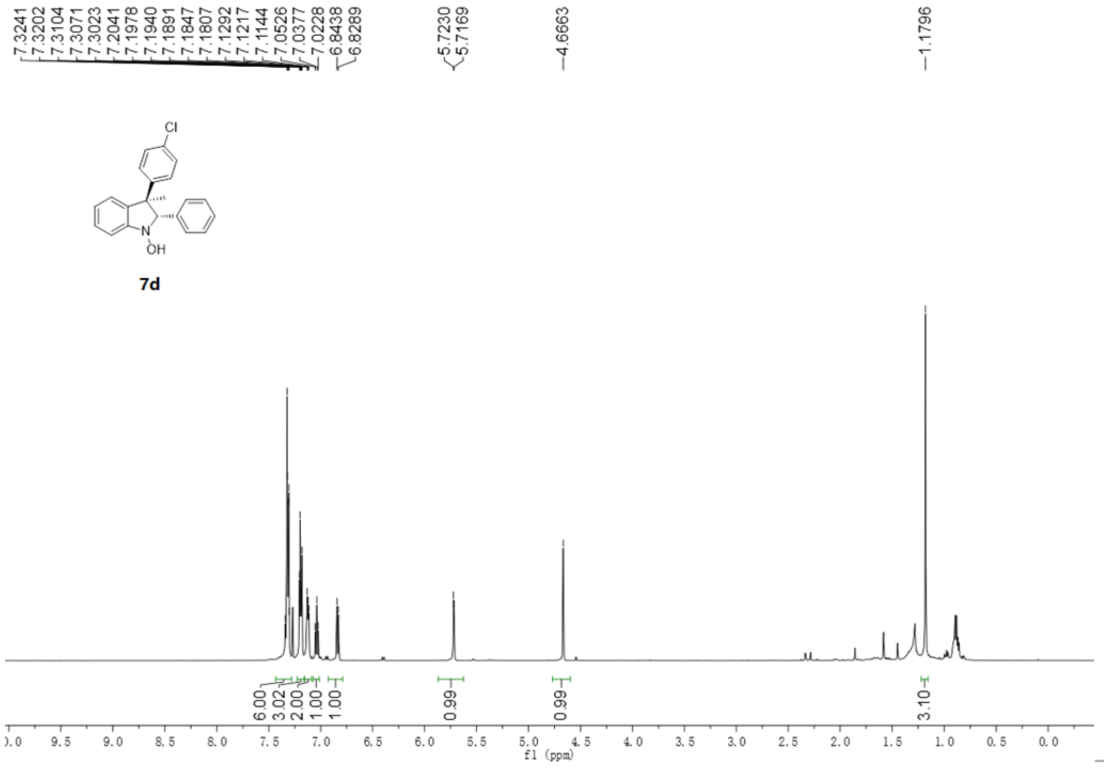

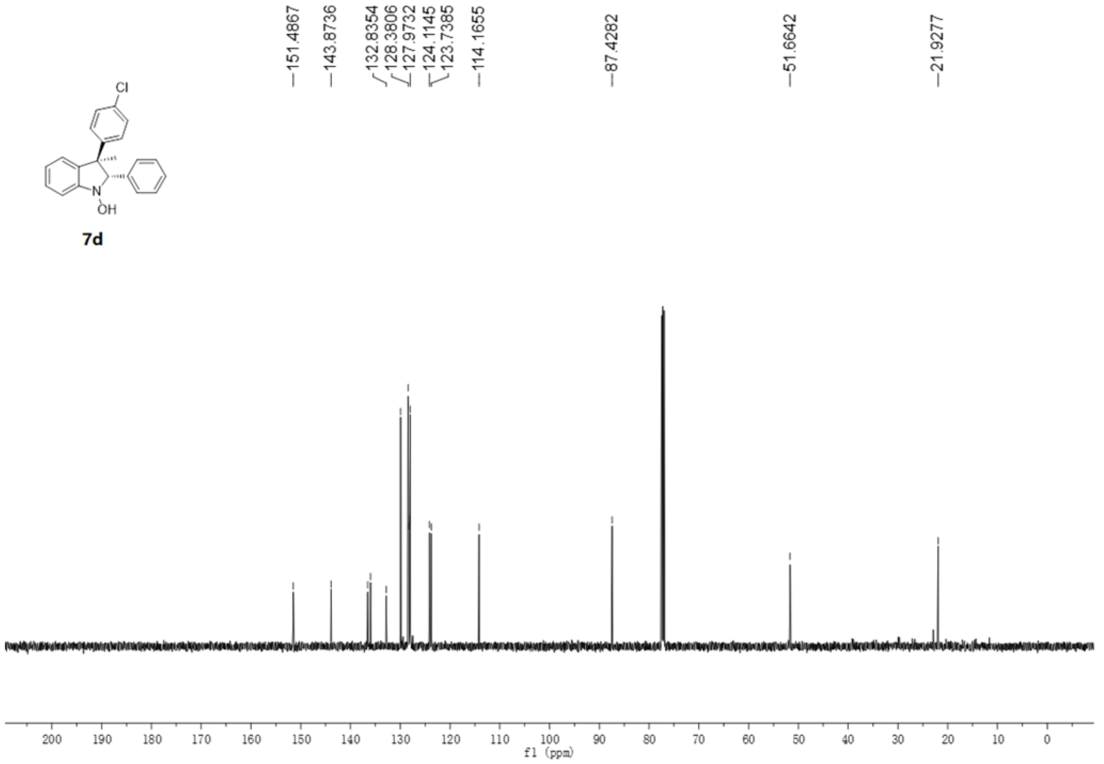

Supplementary figure 80H & ^{13}C NMR spectra of **7d**.

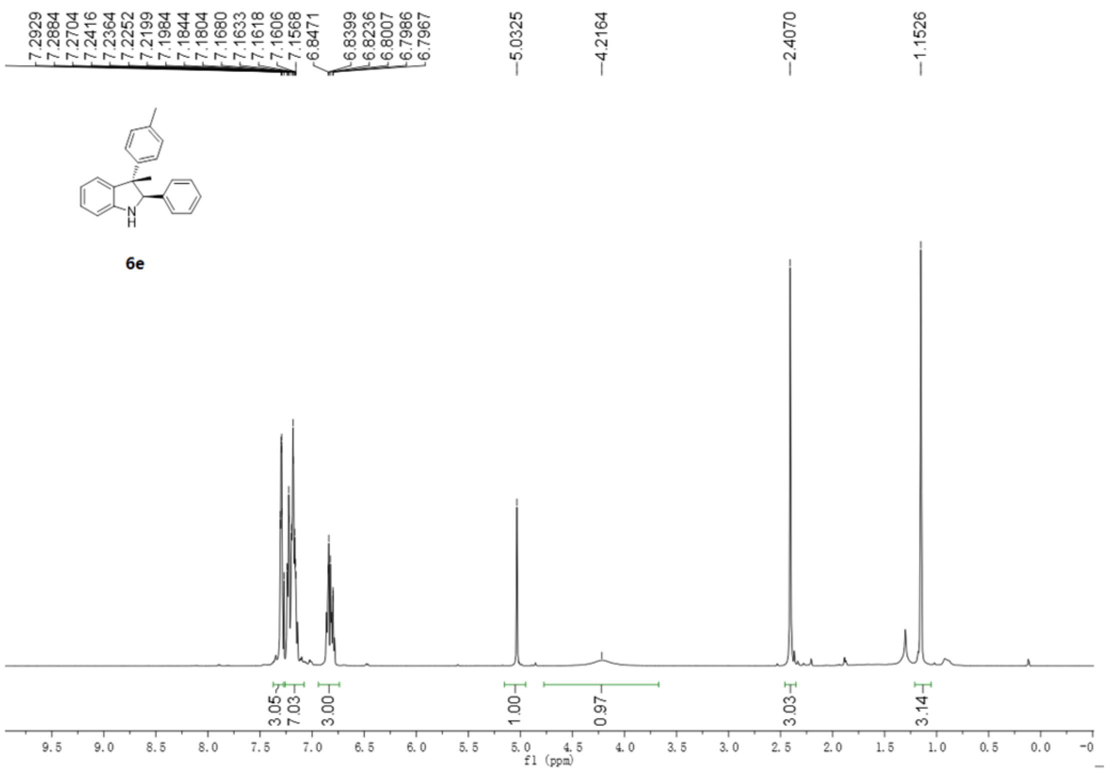

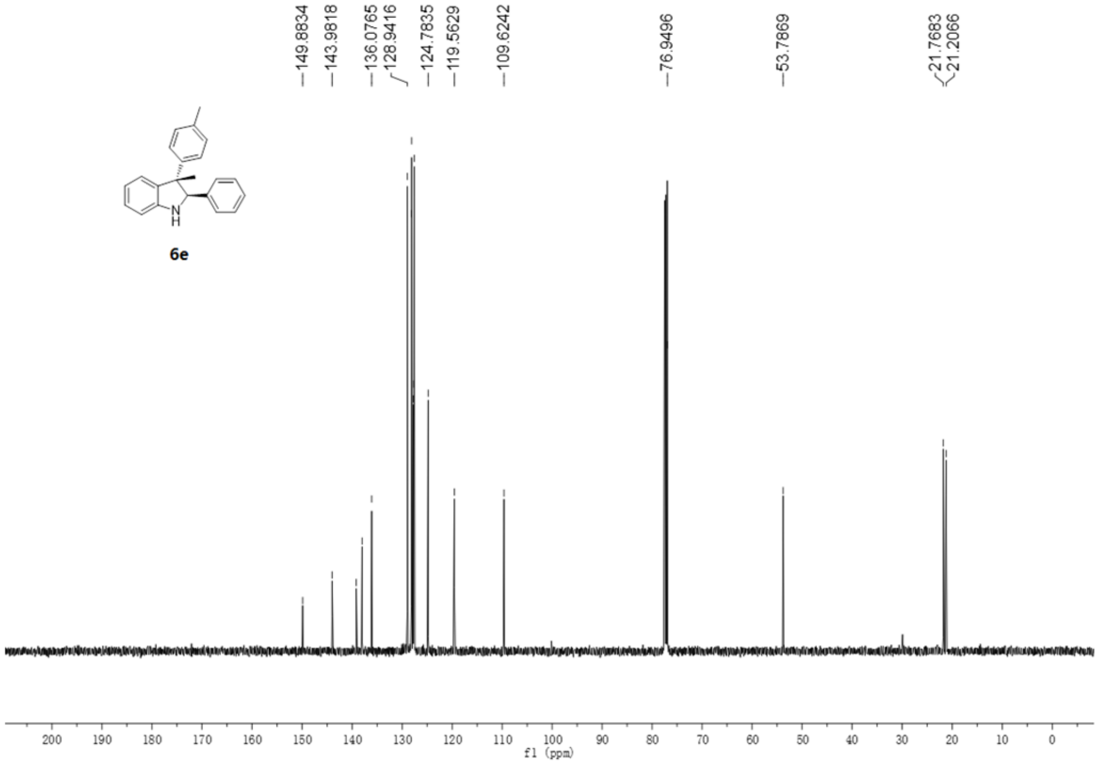

Supplementary figure 81^{1}H & ^{13}C NMR spectra of **6e**.

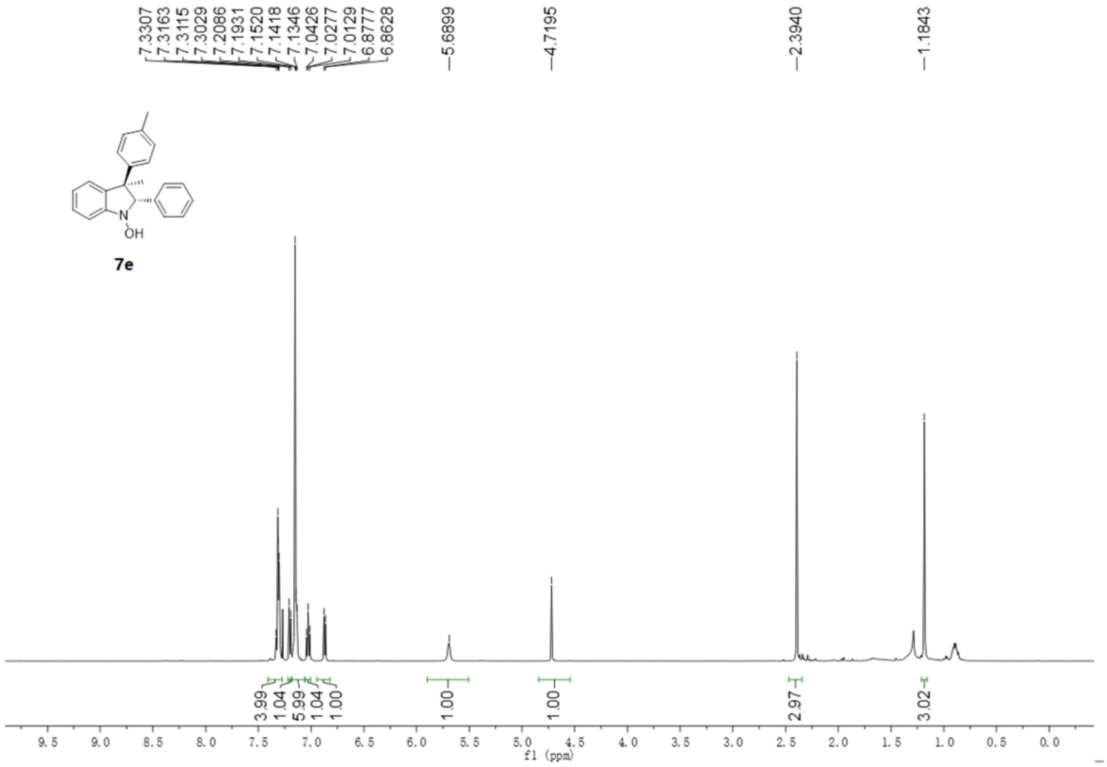

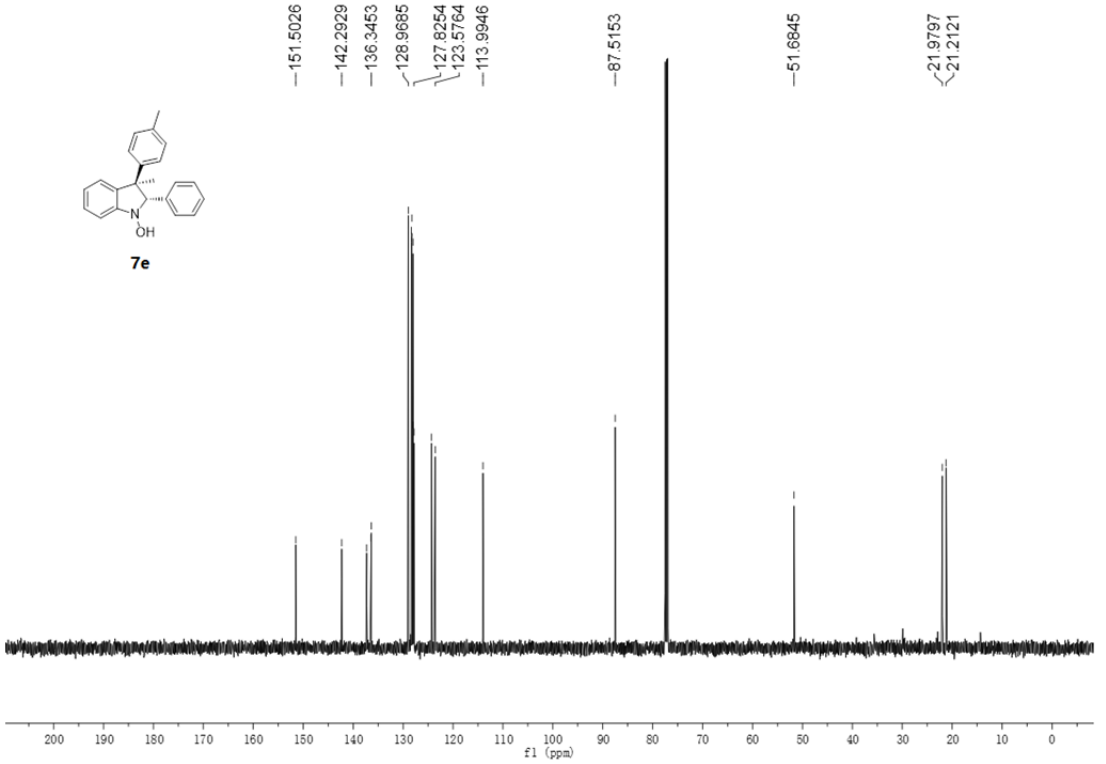

Supplementary figure 82 ^{1}H & ^{13}C NMR spectra of **7e**.

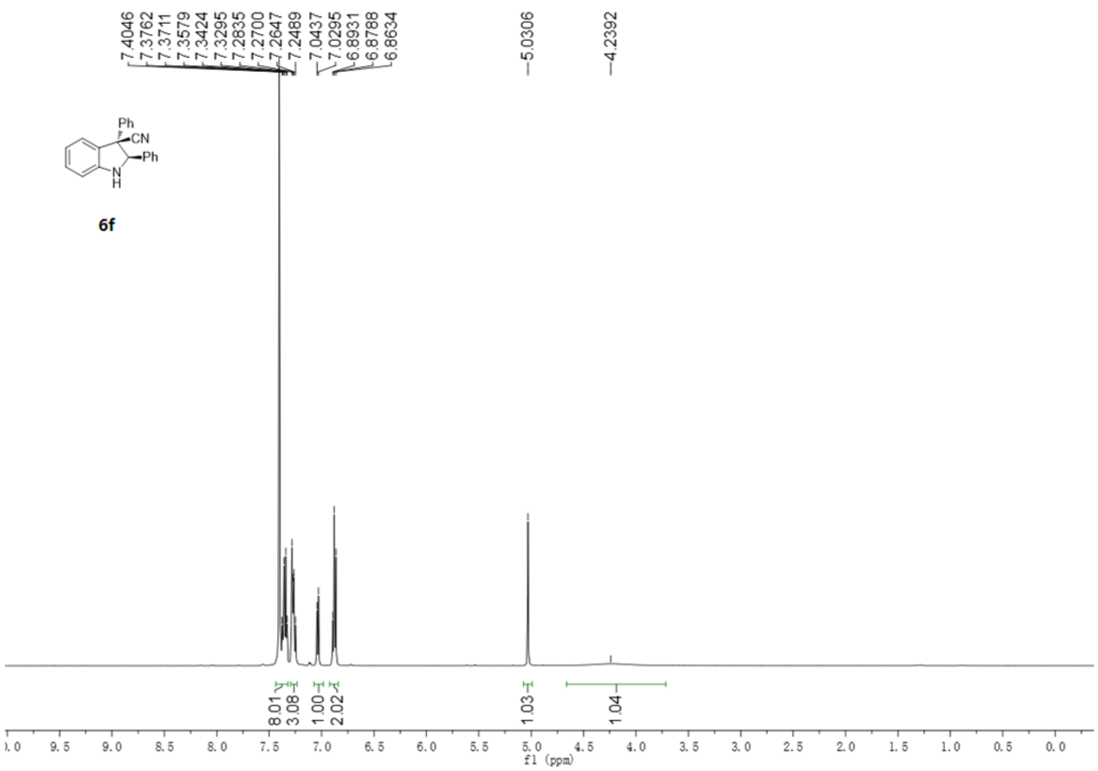

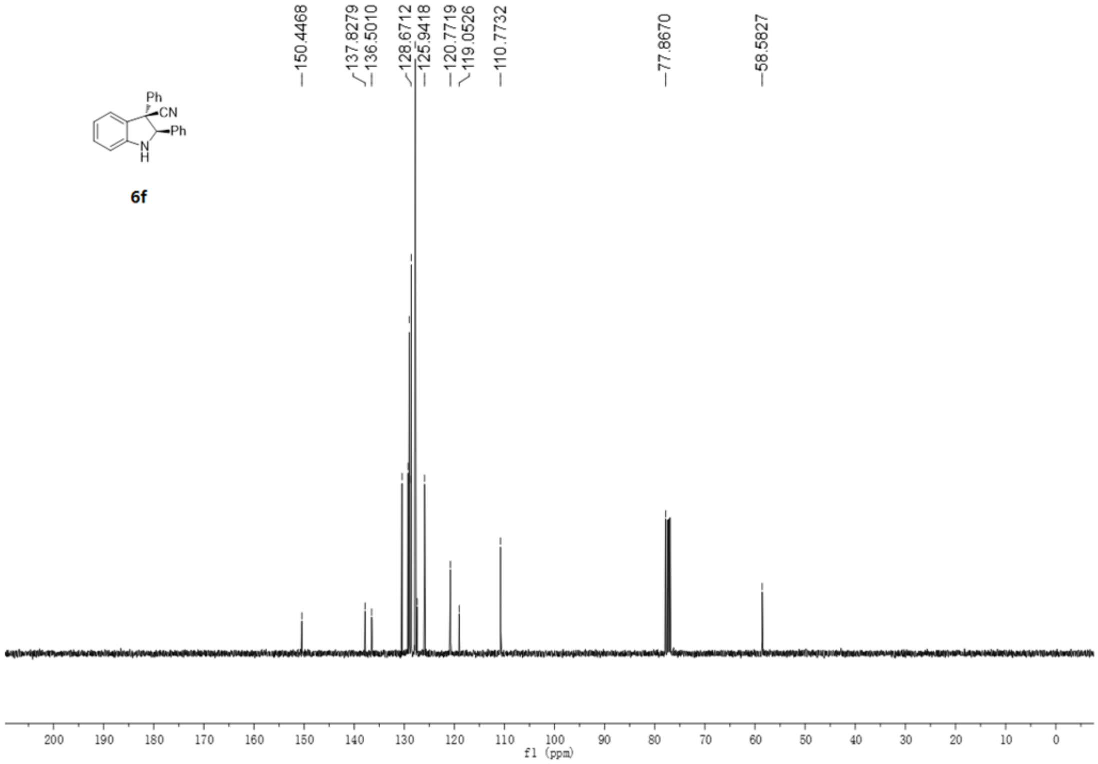

Supplementary figure 83 ^{1}H & ^{13}C NMR spectra of **6f**.

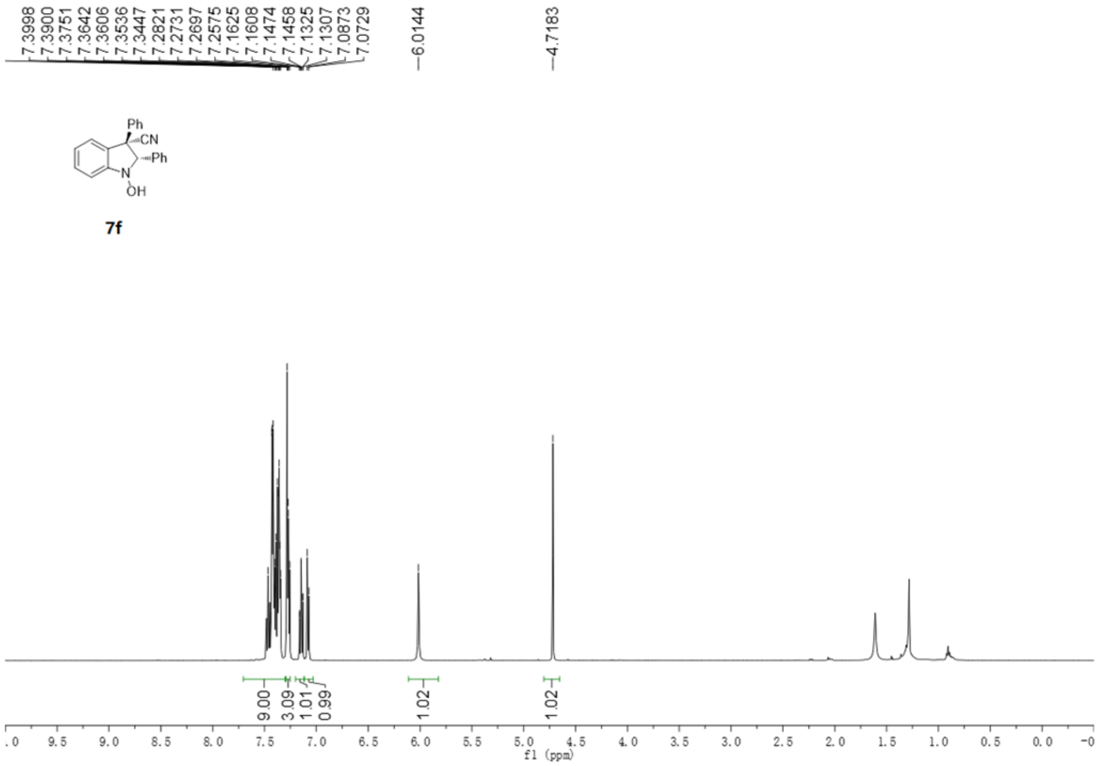

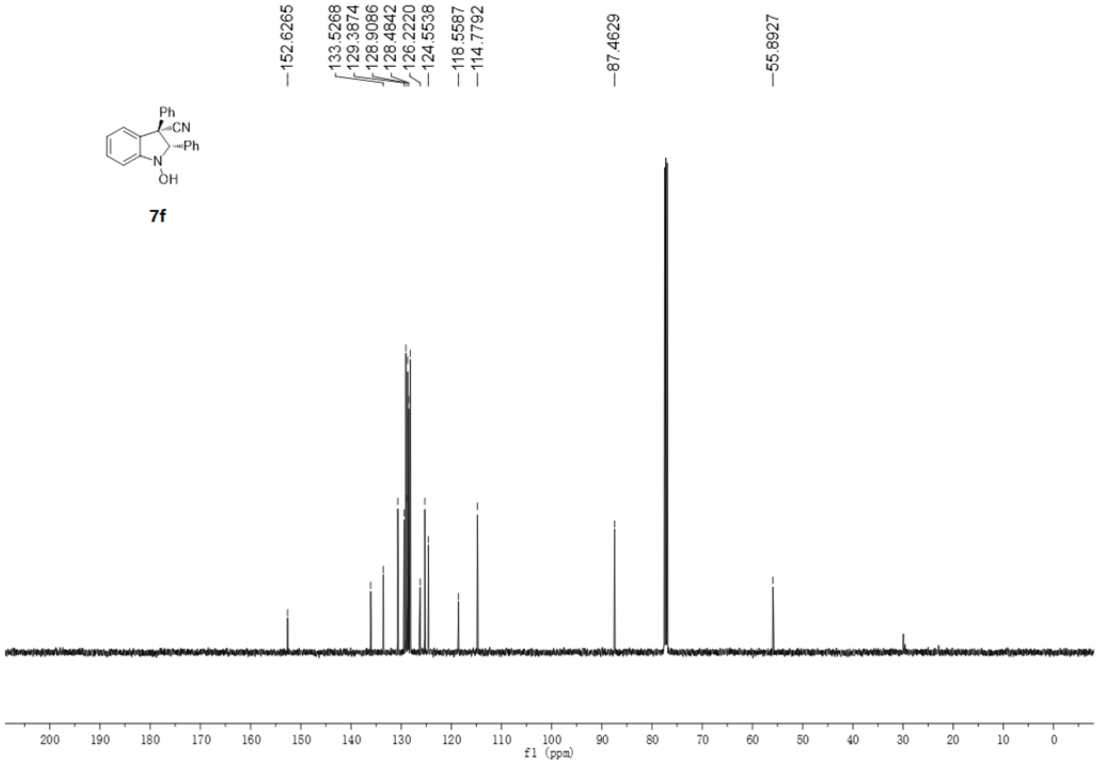

Supplementary figure 84^{1}H & ^{13}C NMR spectra of **7f**.

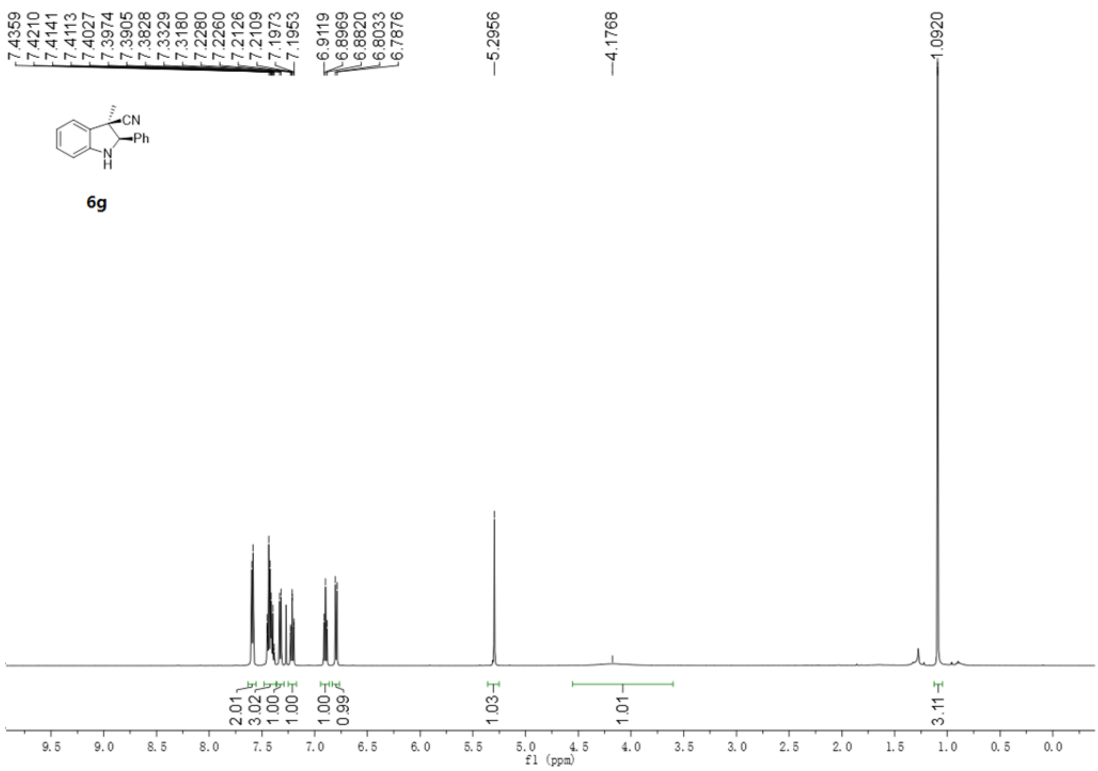

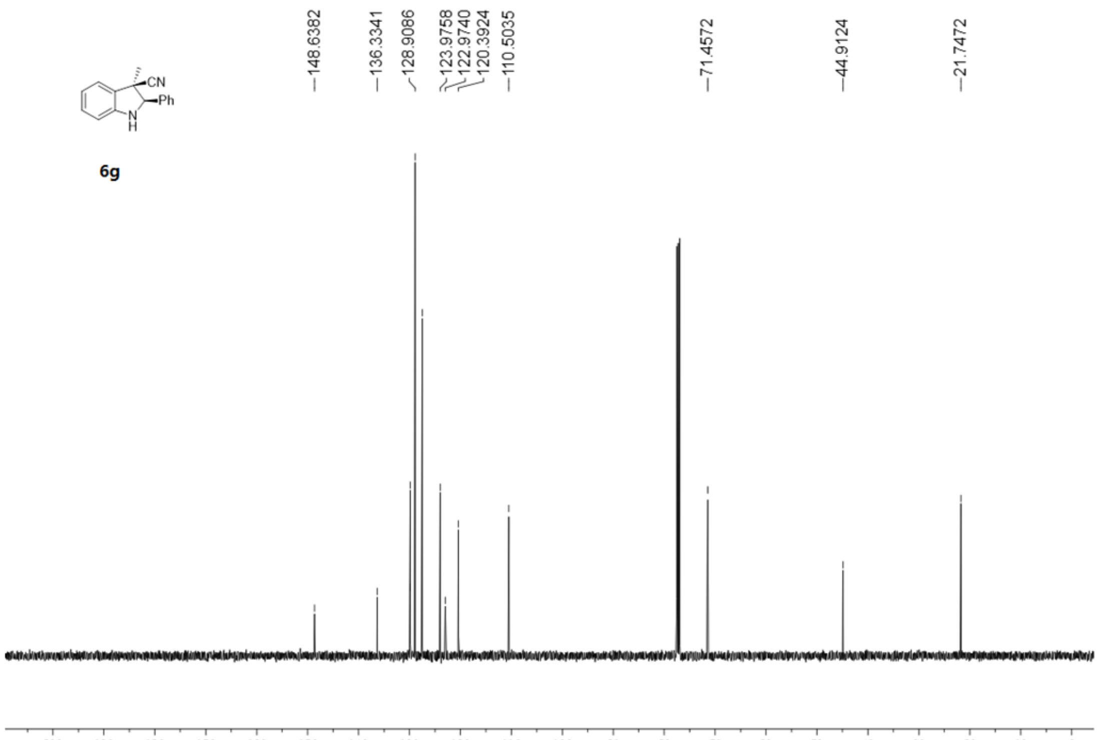

Supplementary figure 85 ^{1}H & ^{13}C NMR spectra of **6g**.

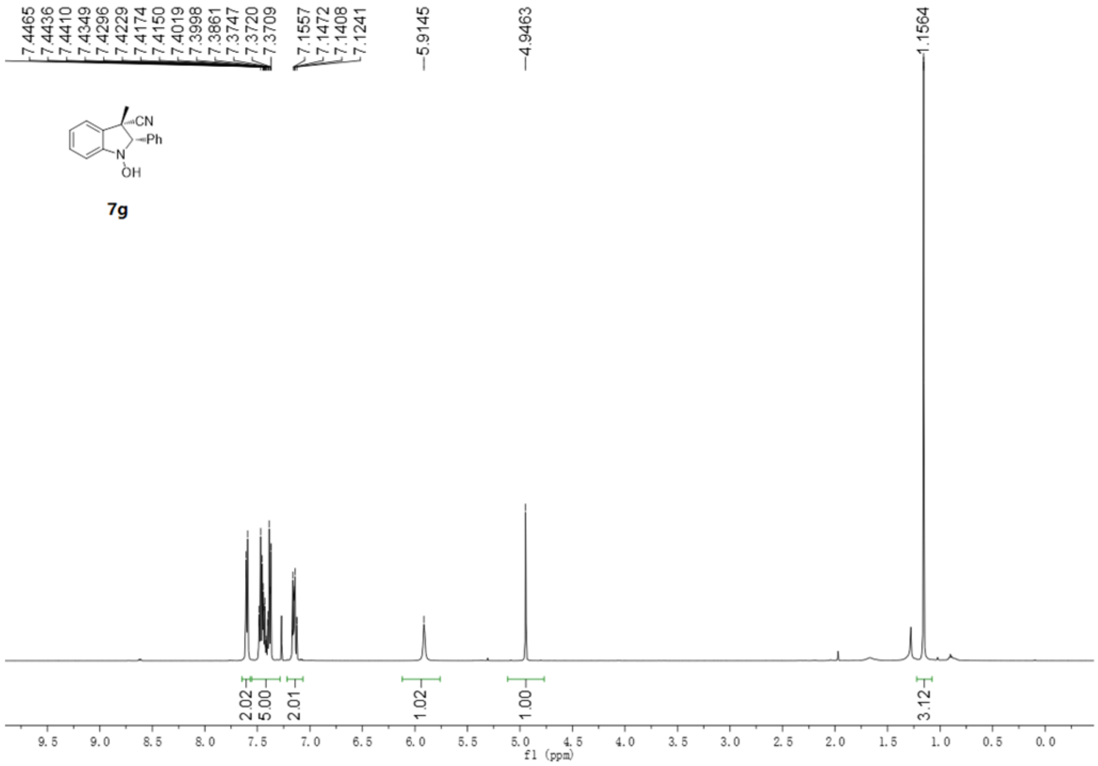

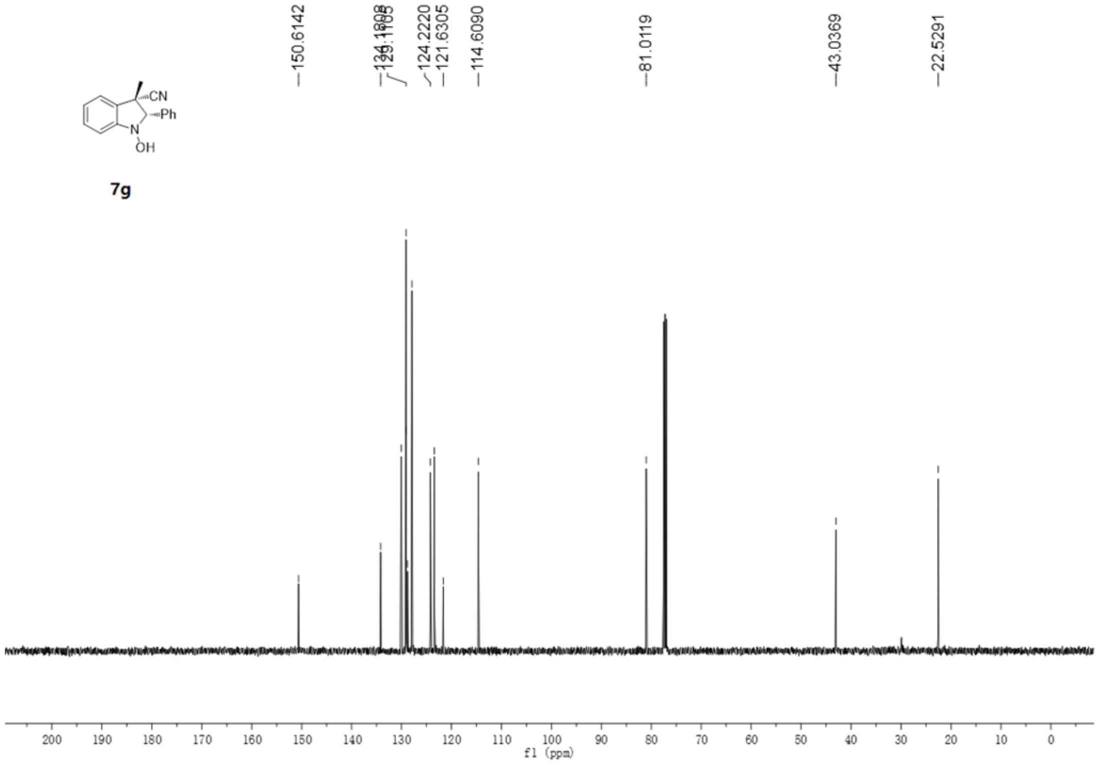

Supplementary figure 86 ^{1}H & ^{13}C NMR spectra of **7g**.

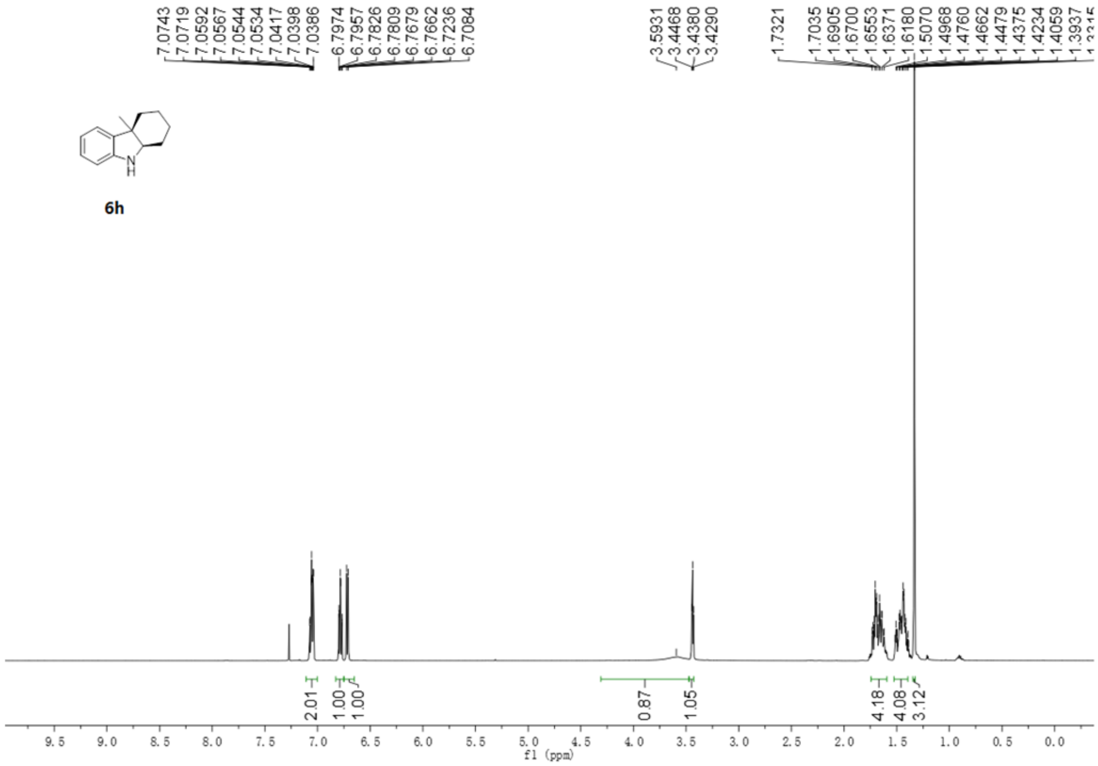

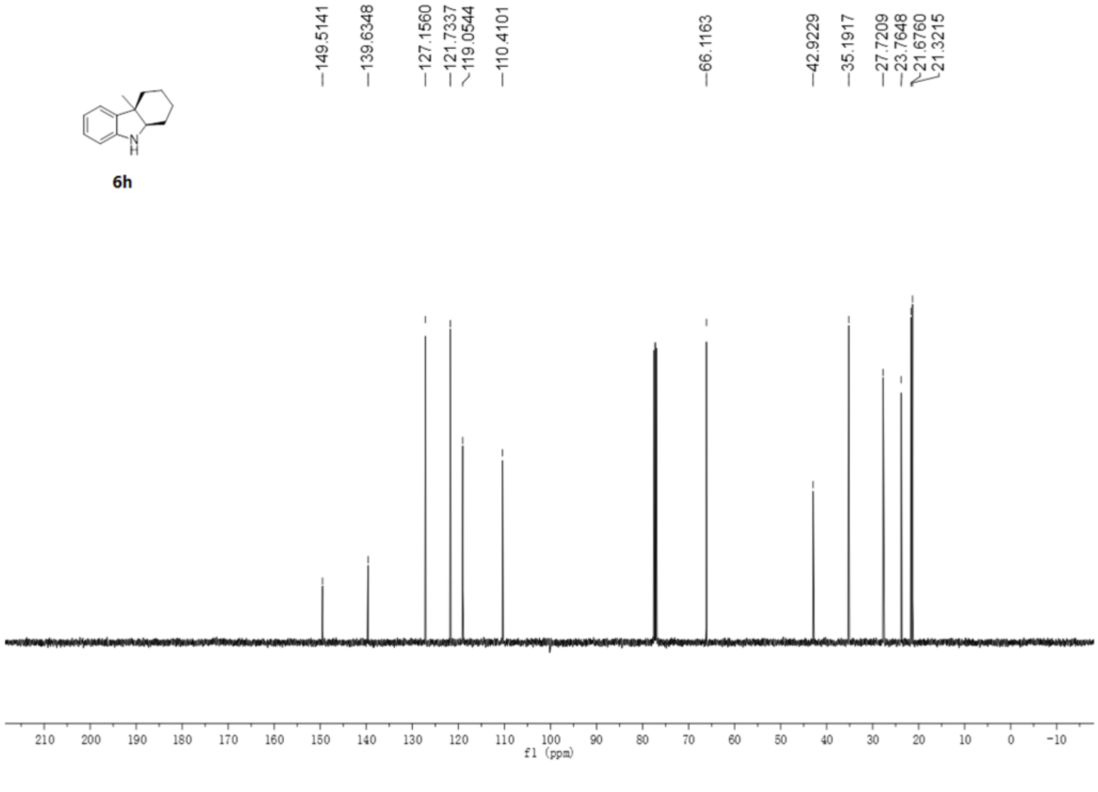

Supplementary figure 87 ^{1}H & ^{13}C NMR spectra of **6h**.

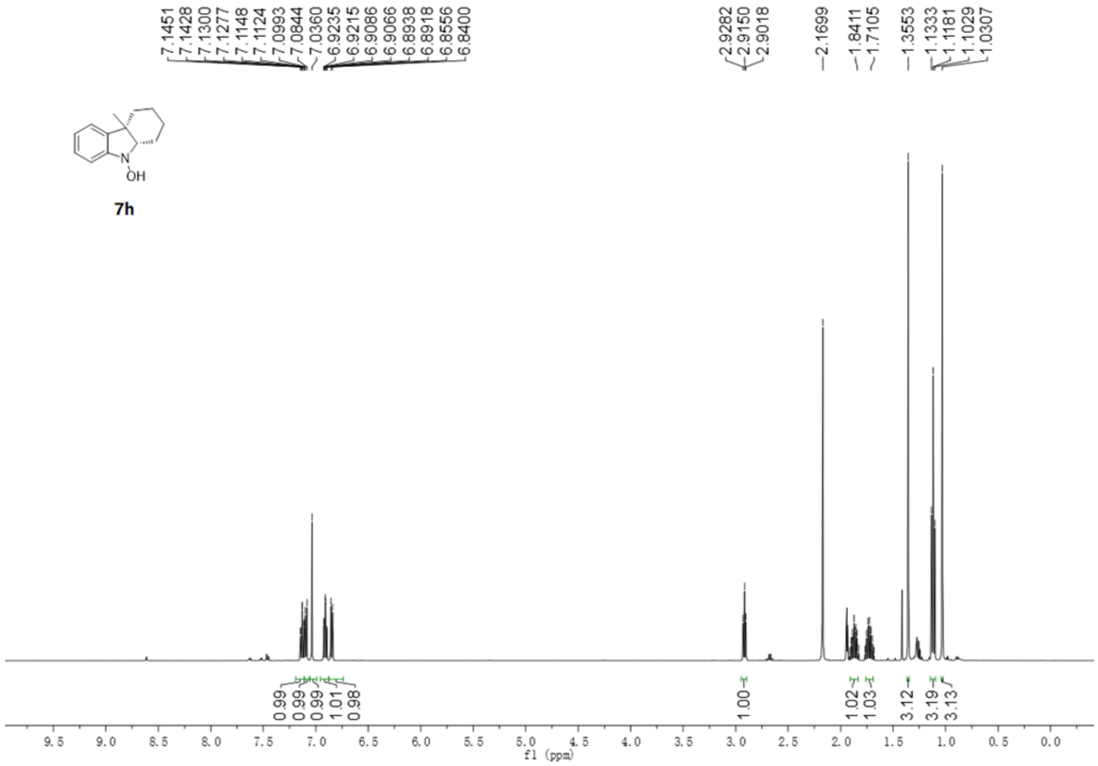

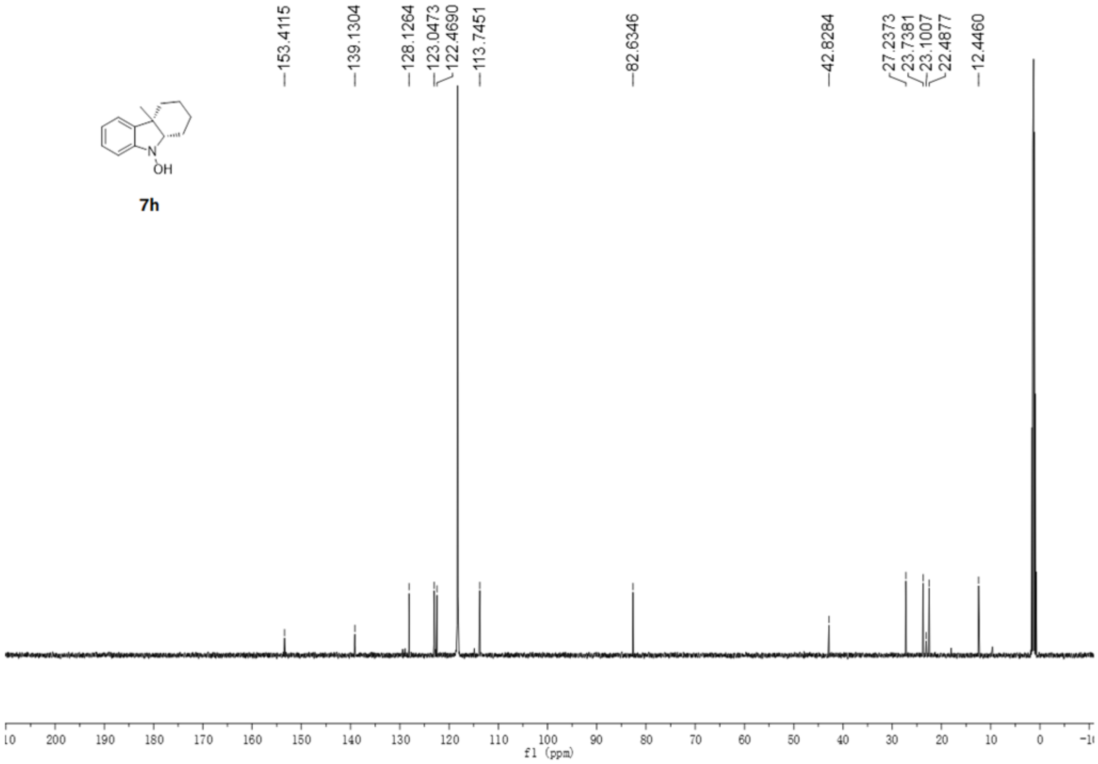

Supplementary figure 88 ^{1}H & ^{13}C NMR spectra of **7h**.

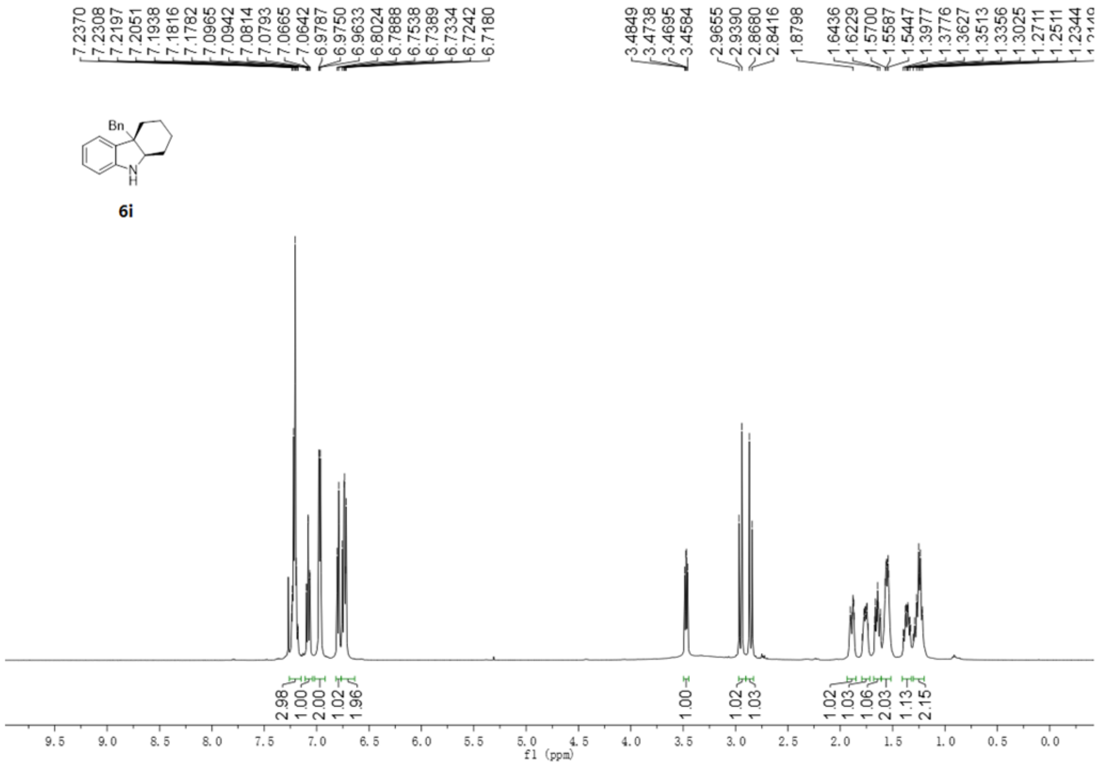

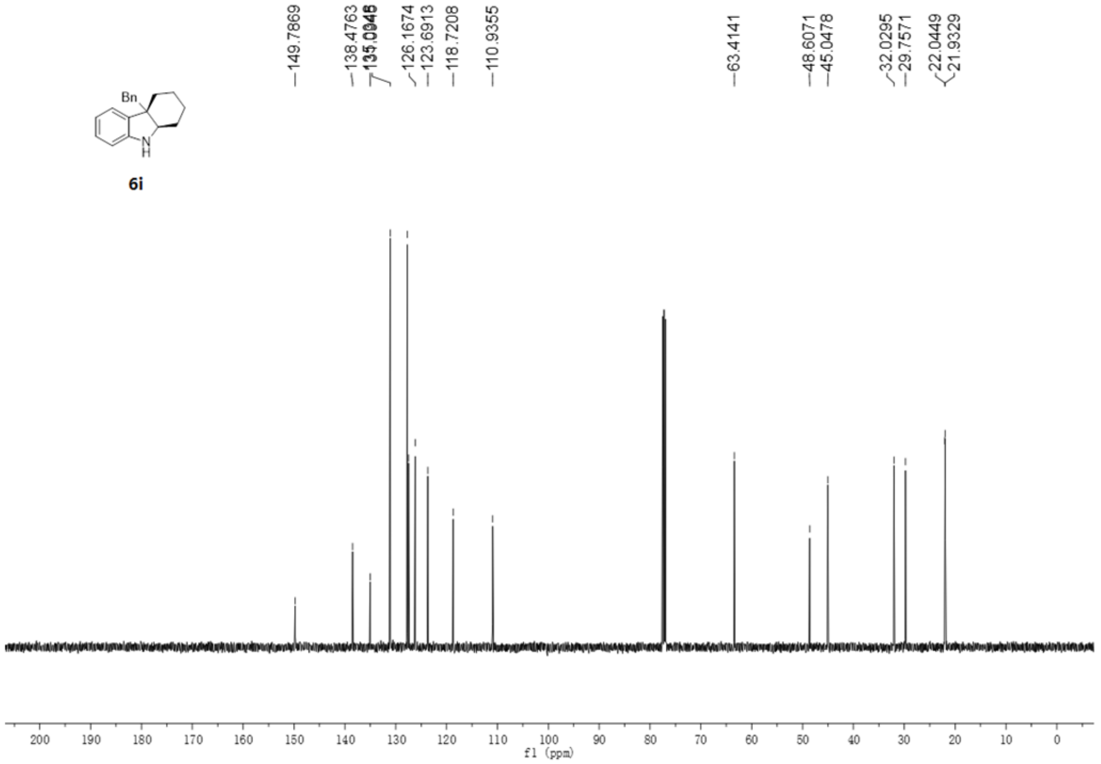

Supplementary figure 89 ^{1}H & ^{13}C NMR spectra of **6i**.

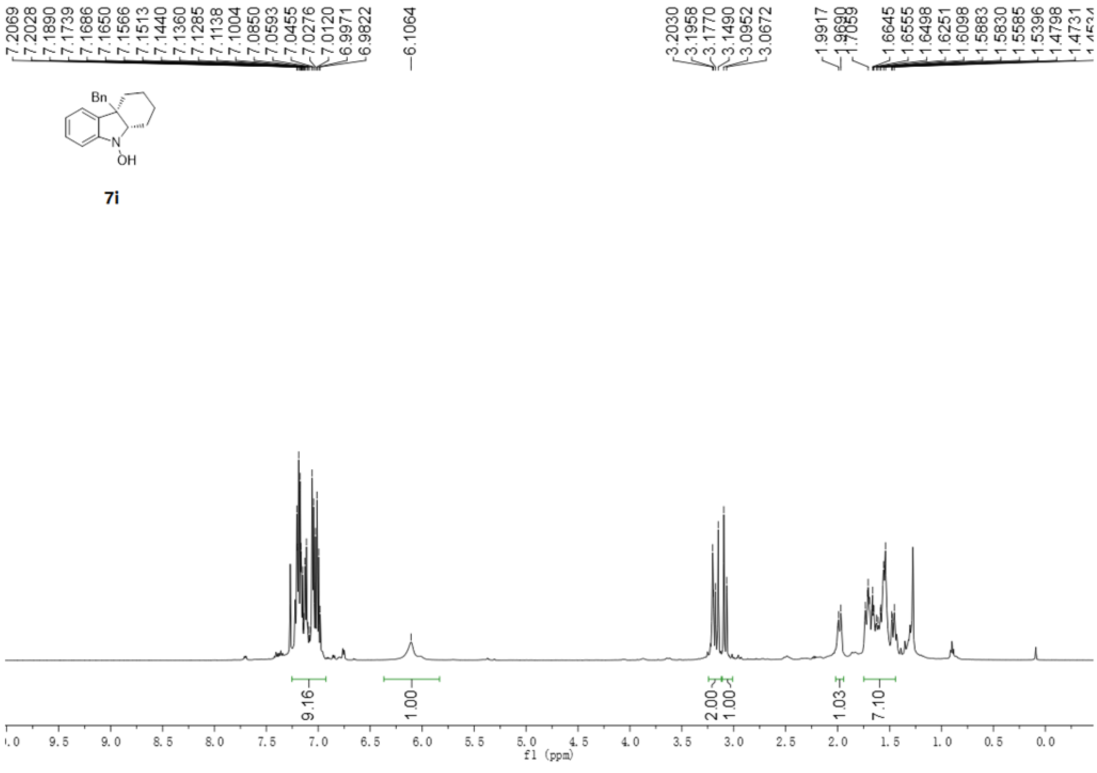

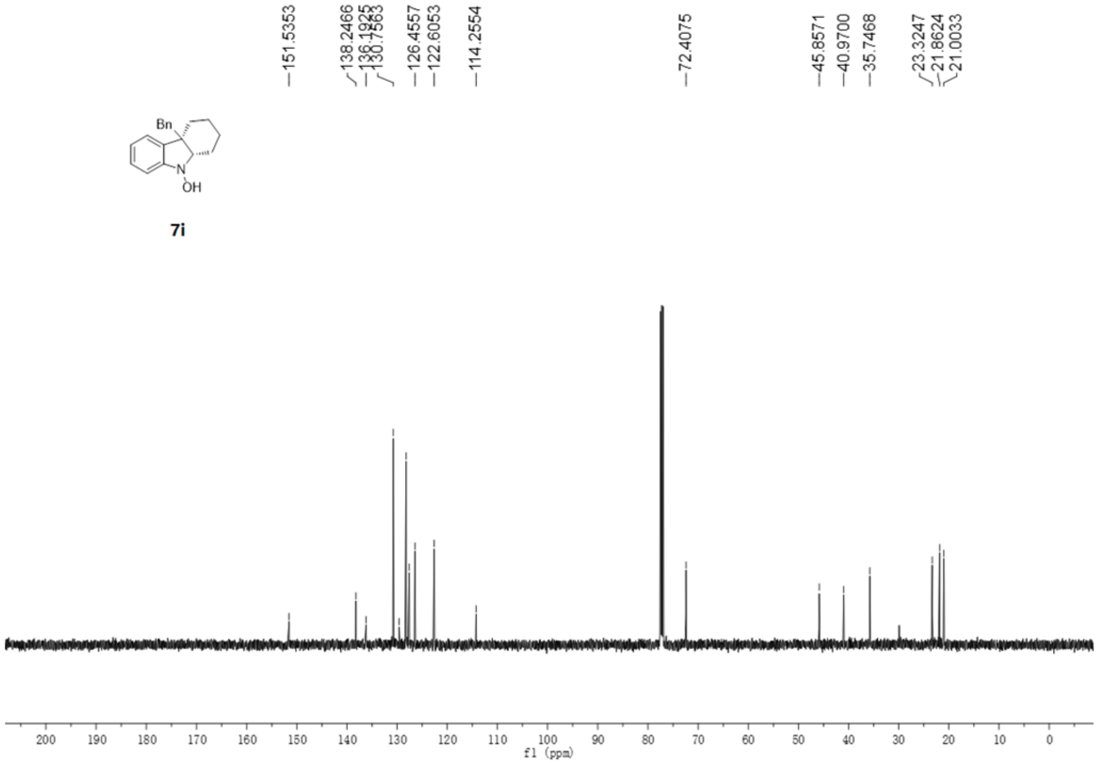

Supplementary figure 90^{1}H & ^{13}C NMR spectra of **7i**.

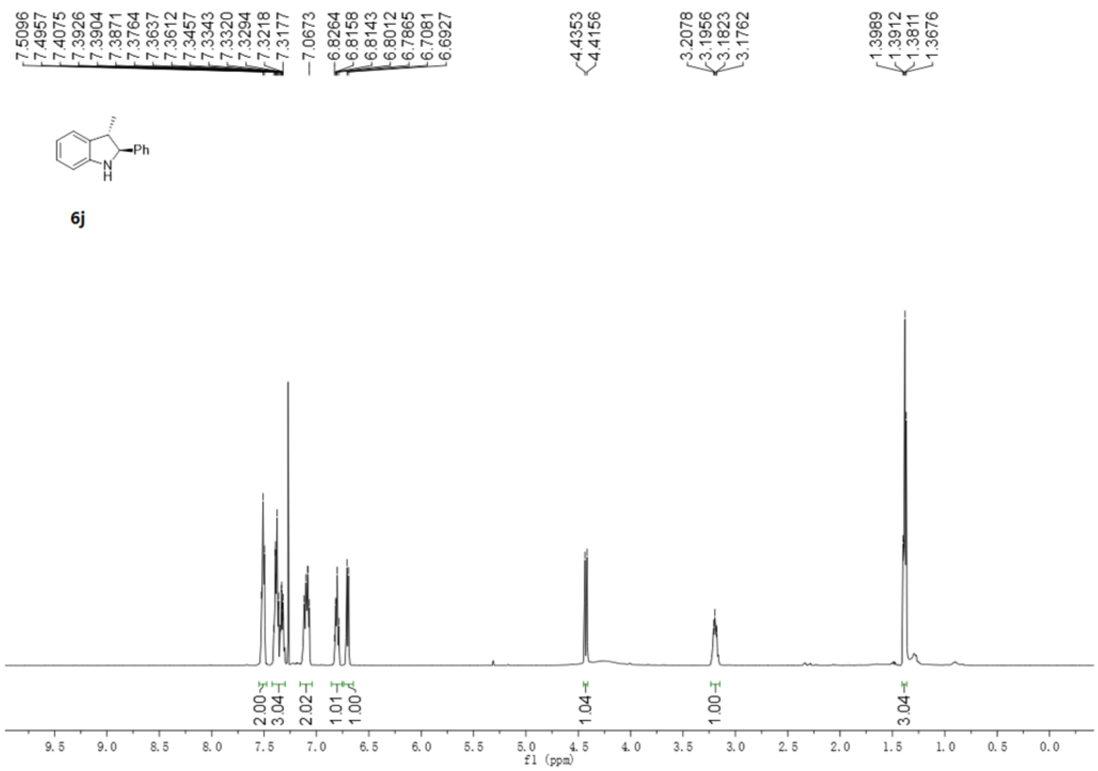

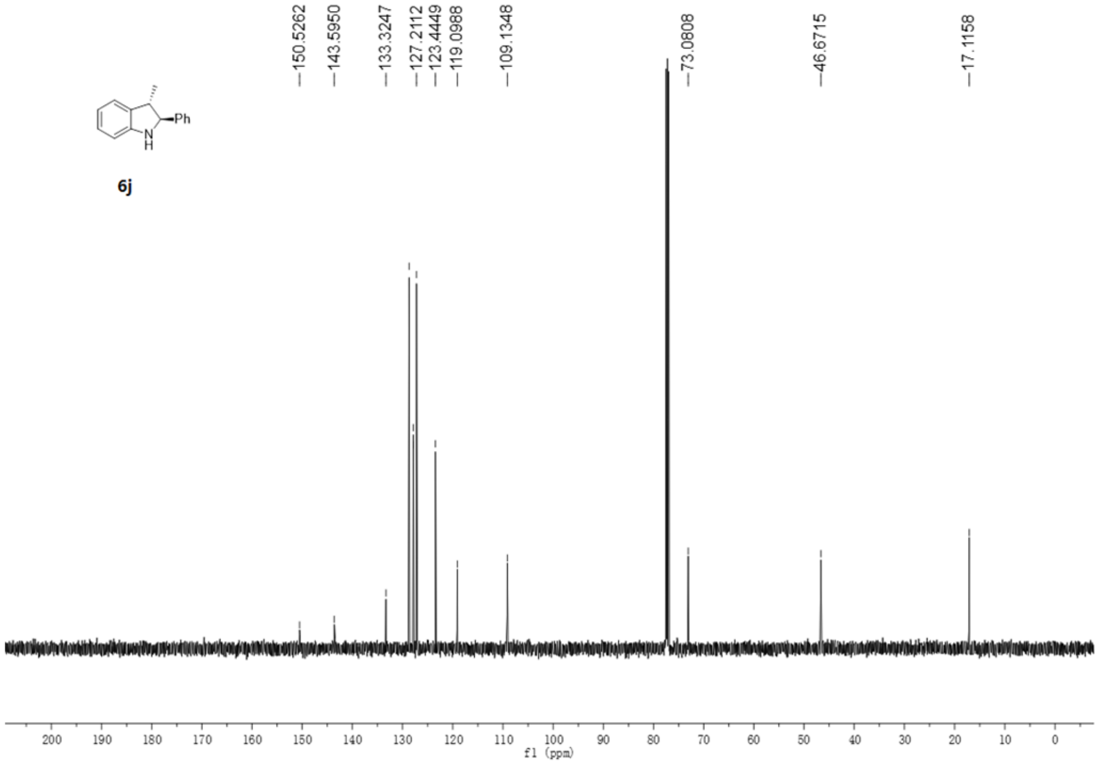

Supplementary figure 91 ^{1}H & ^{13}C NMR spectra of **6j**.

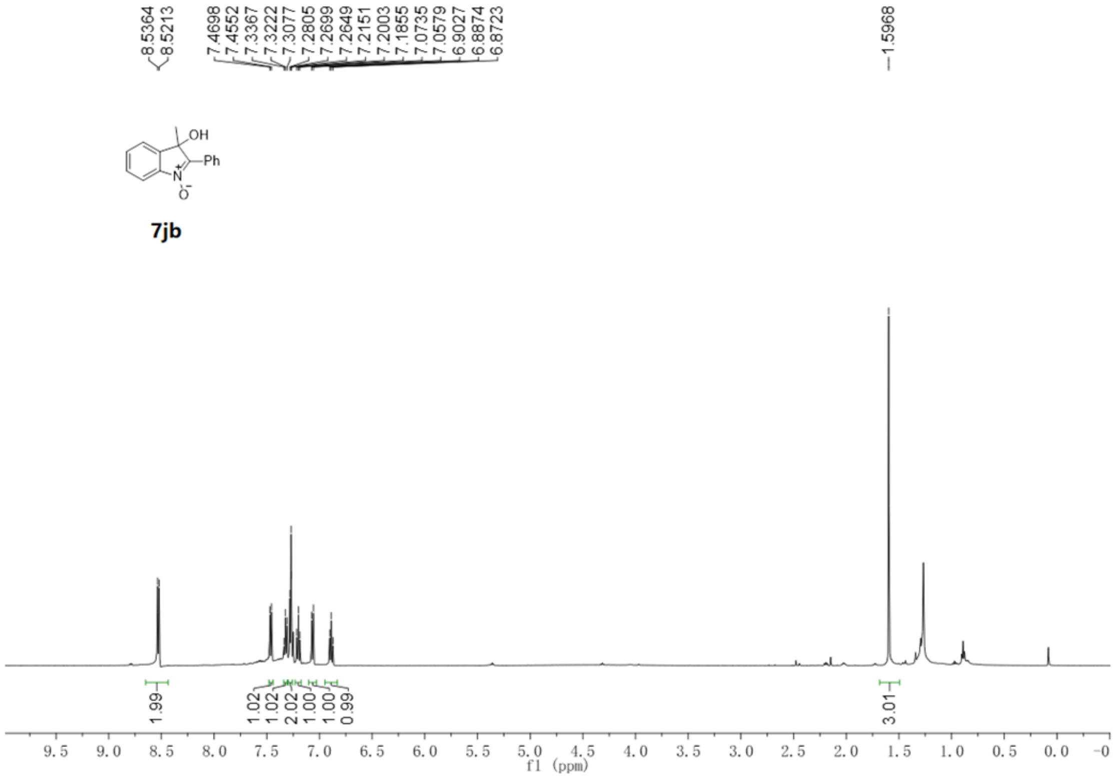

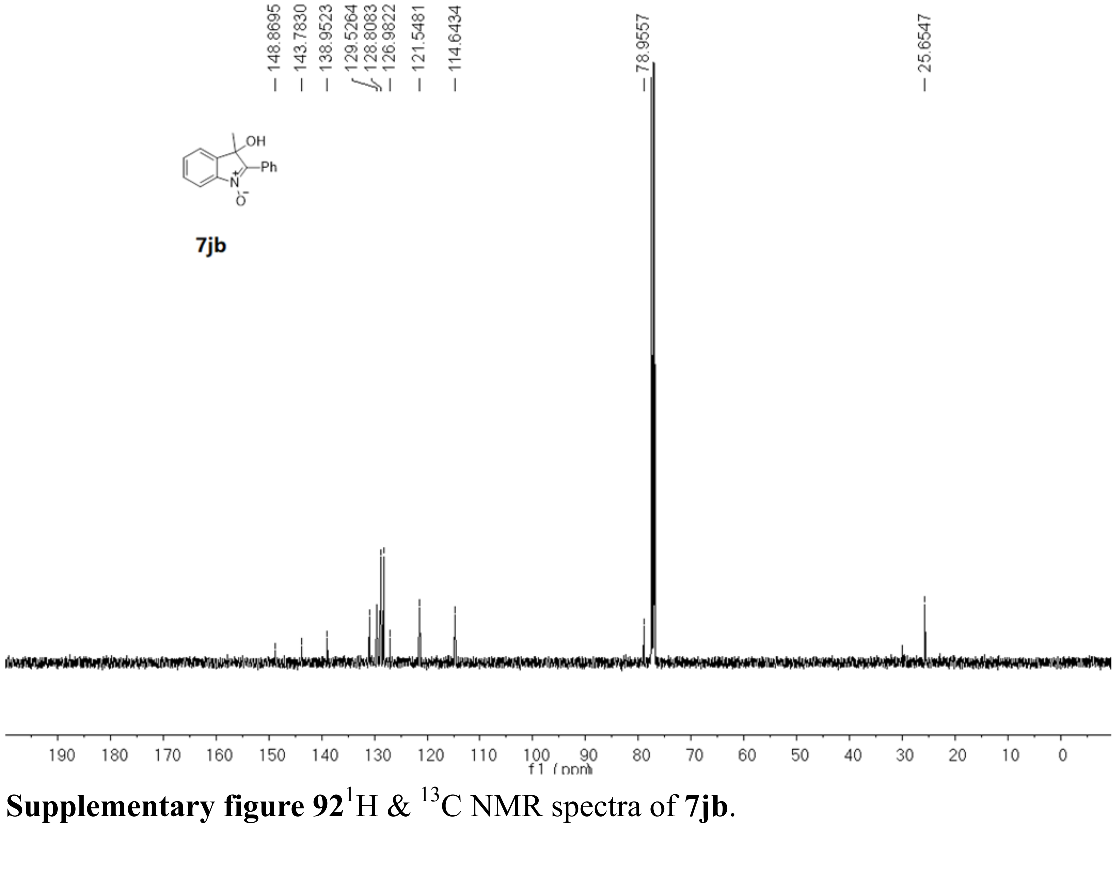

Supplementary figure 92 ^{1}H & ^{13}C NMR spectra of **7jb**.

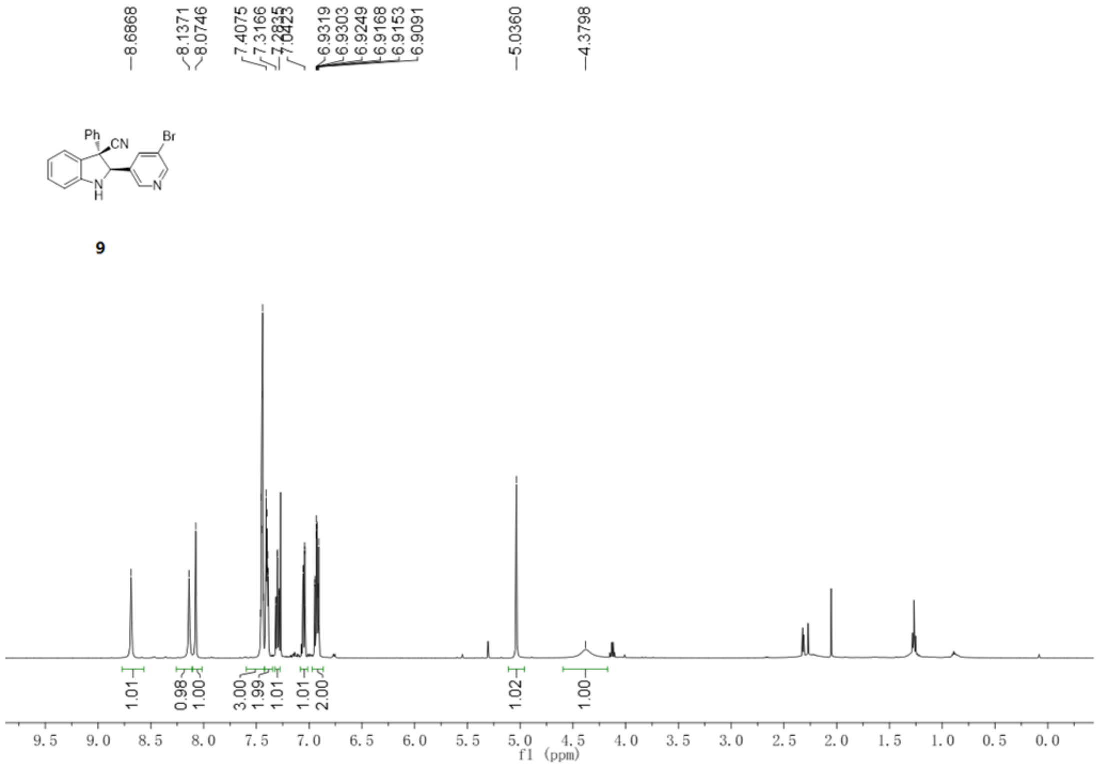

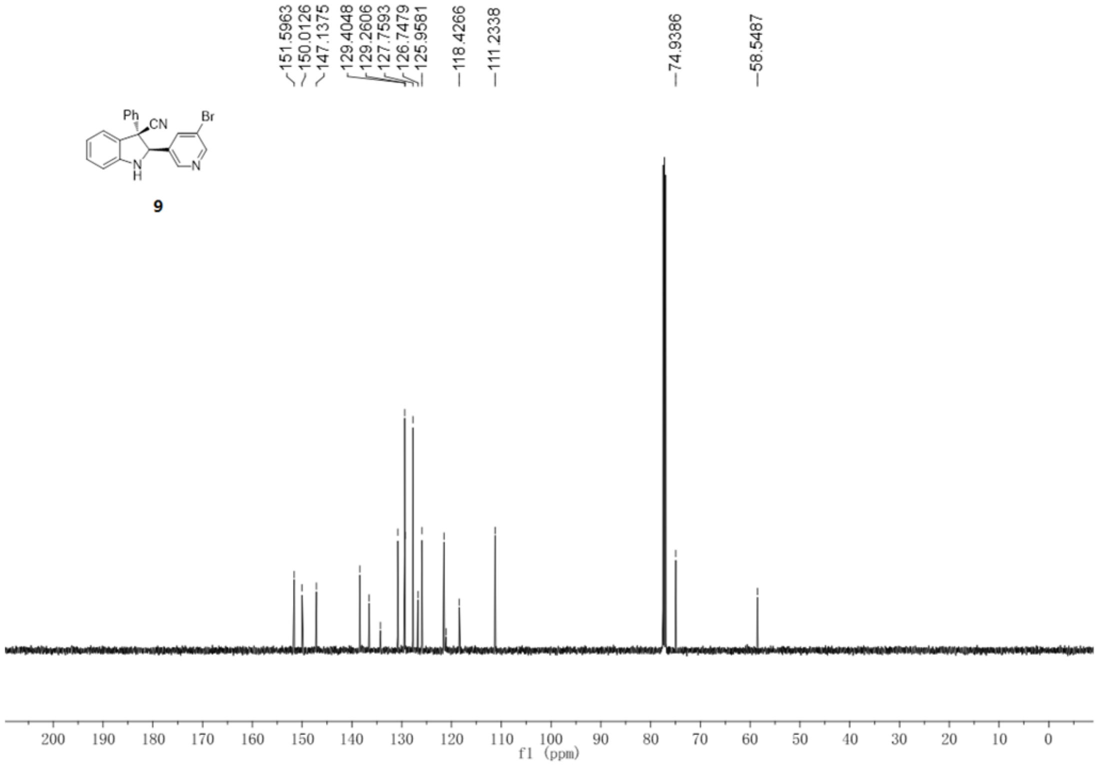

Supplementary figure 93 ^{1}H & ^{13}C NMR spectra of **7j**.

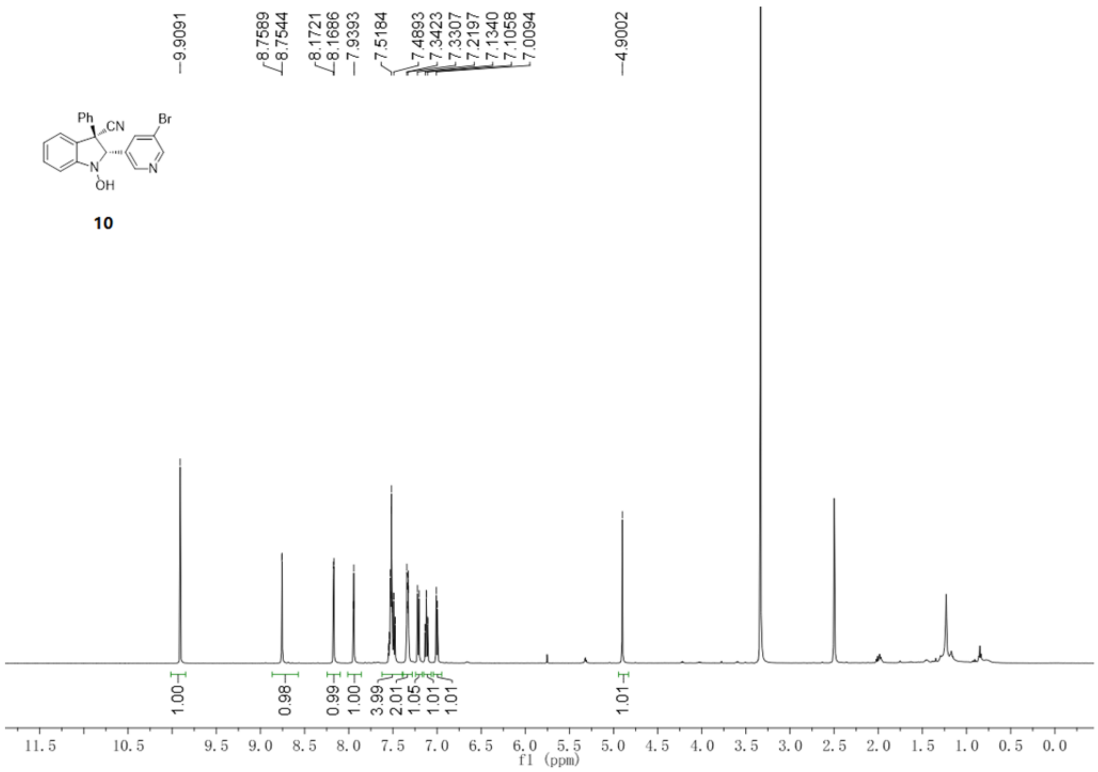

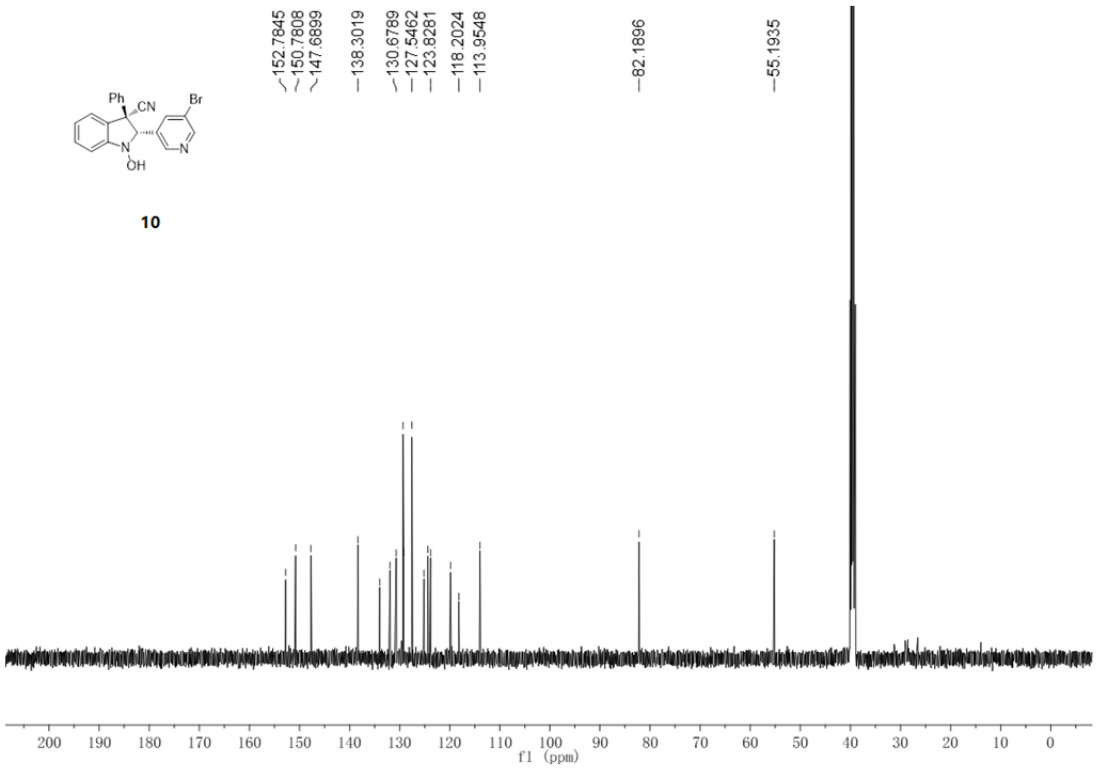

Supplementary figure 94 ^{1}H & ^{13}C NMR spectra of **10**.

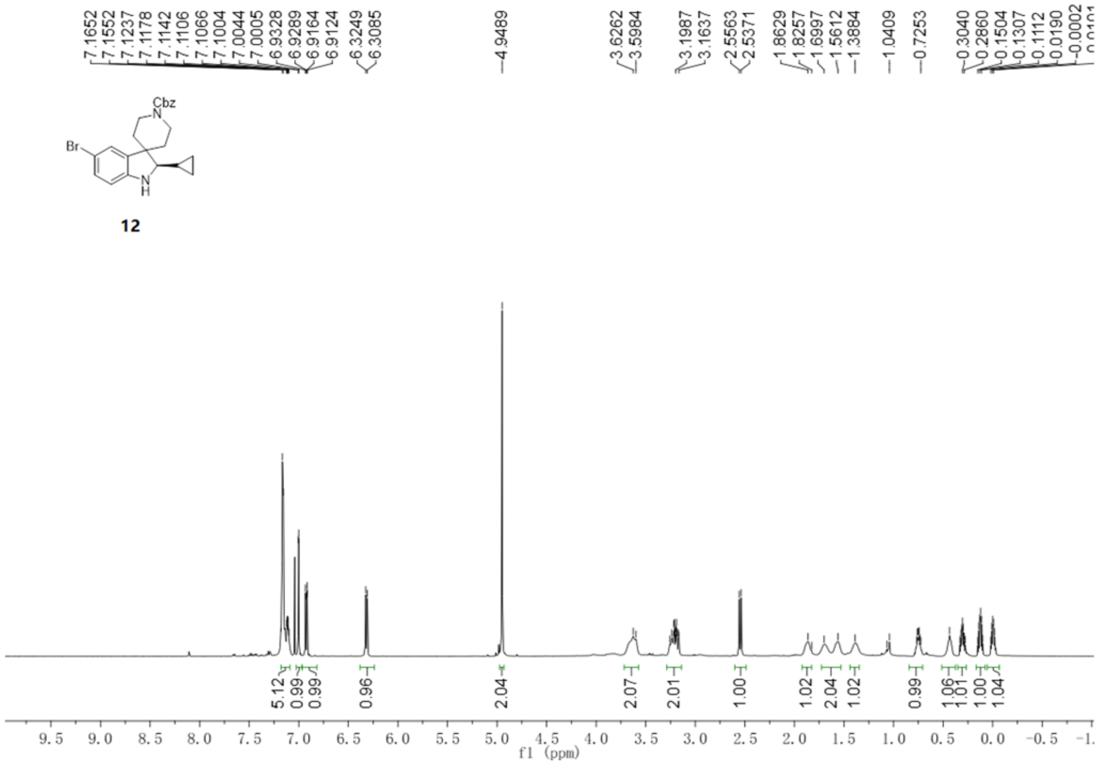

12

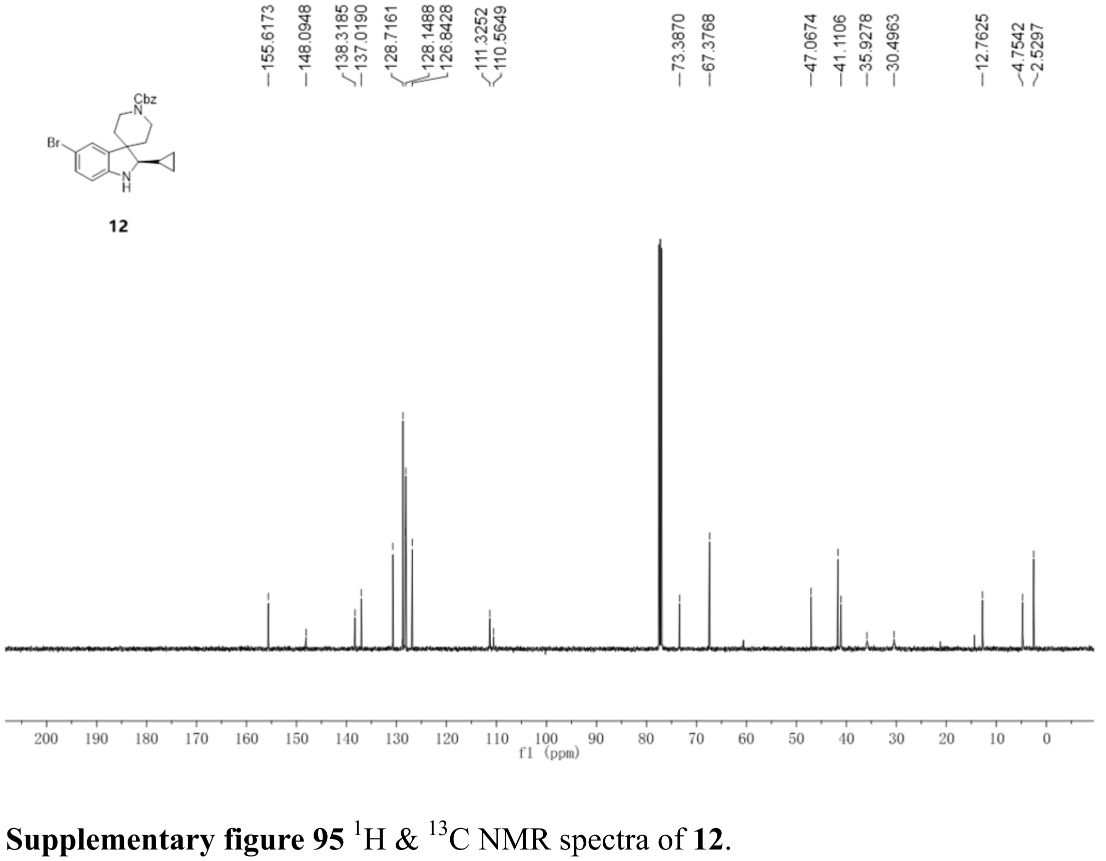

12

Supplementary figure 95 ^{1}H & ^{13}C NMR spectra of **12**.

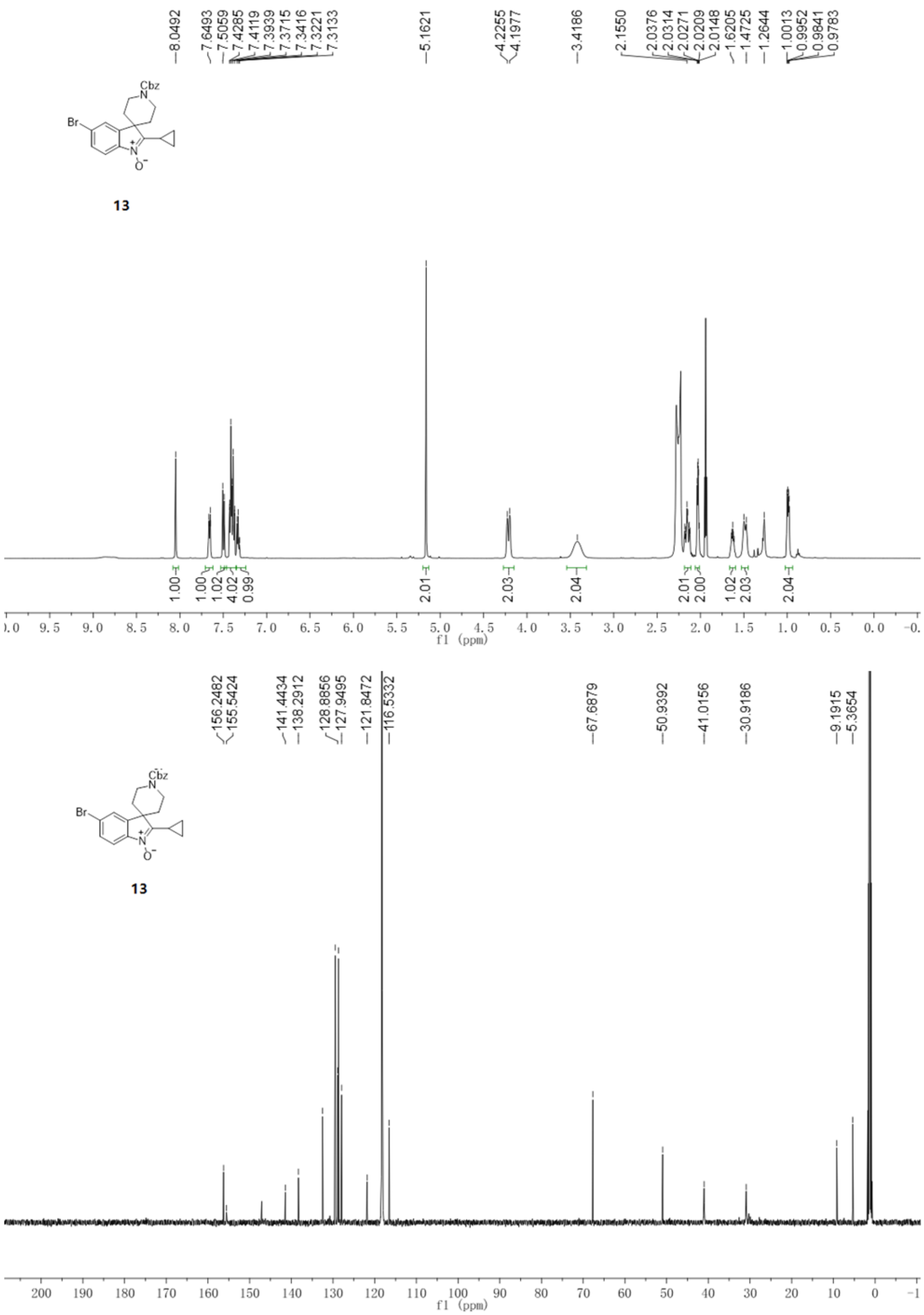

Supplementary figure 96 ^{1}H & ^{13}C NMR spectra of **13**.

HPLC traces

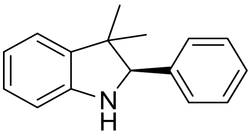

1a

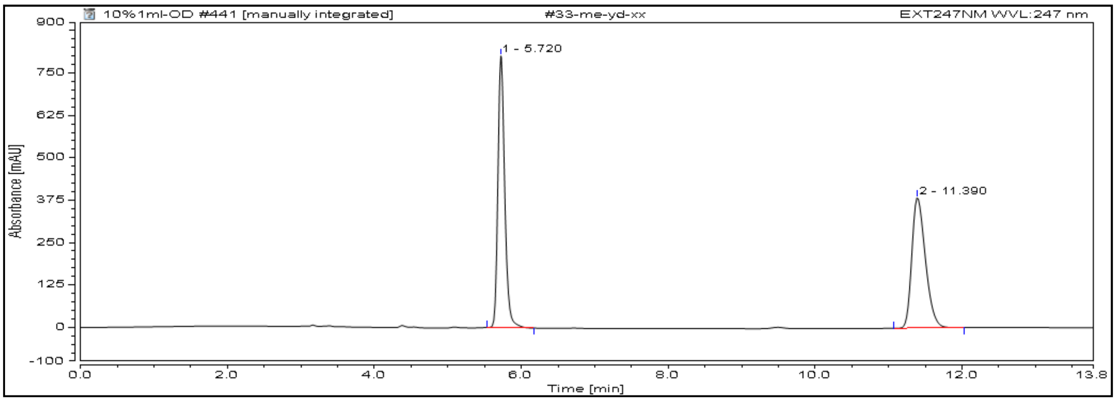

Integration Results					
No.	Peak Name	Retention Time min	Area mAU*min	Relative Area %	Amount n.a.
1		5.720	86.181	51.17	n.a.
2		11.390	82.225	48.83	n.a.
**Total:**			**168.406**	**100.00**	

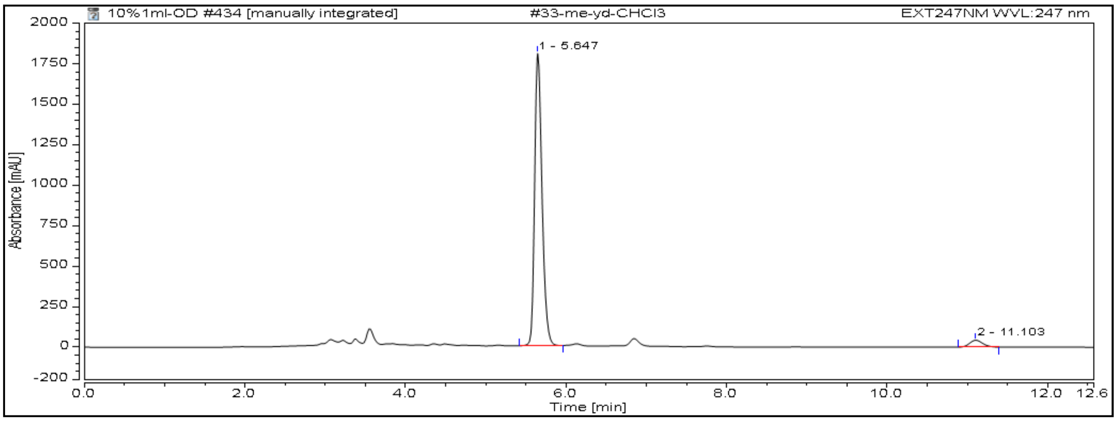

Integration Results					
No.	Peak Name	Retention Time min	Area mAU*min	Relative Area %	Amount n.a.
1		5.647	192.054	95.91	n.a.
2		11.103	8.182	4.09	n.a.
**Total:**			**200.236**	**100.00**	

Supplementary figure 97. HPLC chromatogram for **1a**.

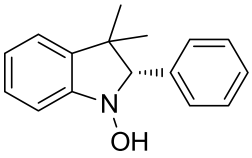

2a

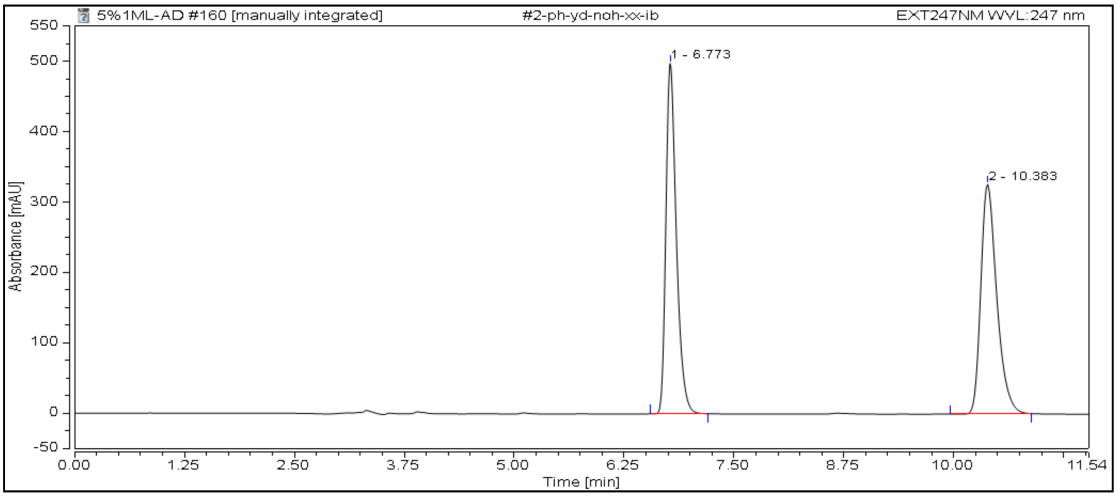

Integration Results					
No.	Peak Name	Retention Time min	Area mAU*min	Relative Area %	Amount n.a.
1		6.773	68.561	49.89	n.a.
2		10.383	68.876	50.11	n.a.
**Total:**			**137.437**	**100.00**	

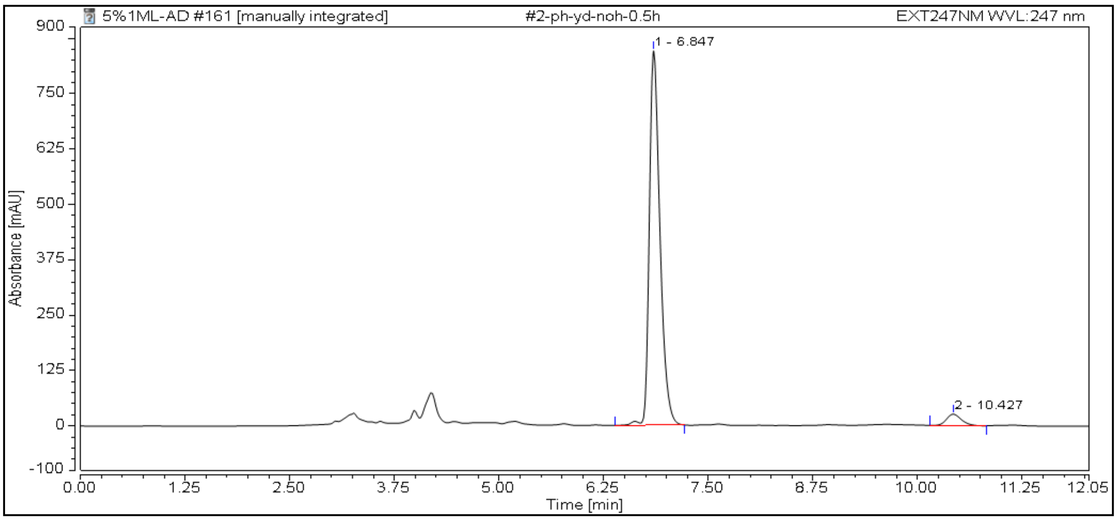

Integration Results					
No.	Peak Name	Retention Time min	Area mAU*min	Relative Area %	Amount n.a.
1		6.847	124.514	95.97	n.a.
2		10.427	5.227	4.03	n.a.
**Total:**			**129.741**	**100.00**	

Supplementary figure 98. HPLC chromatogram for **2a**

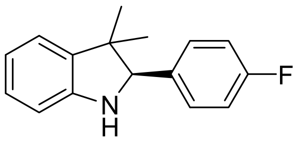

1b

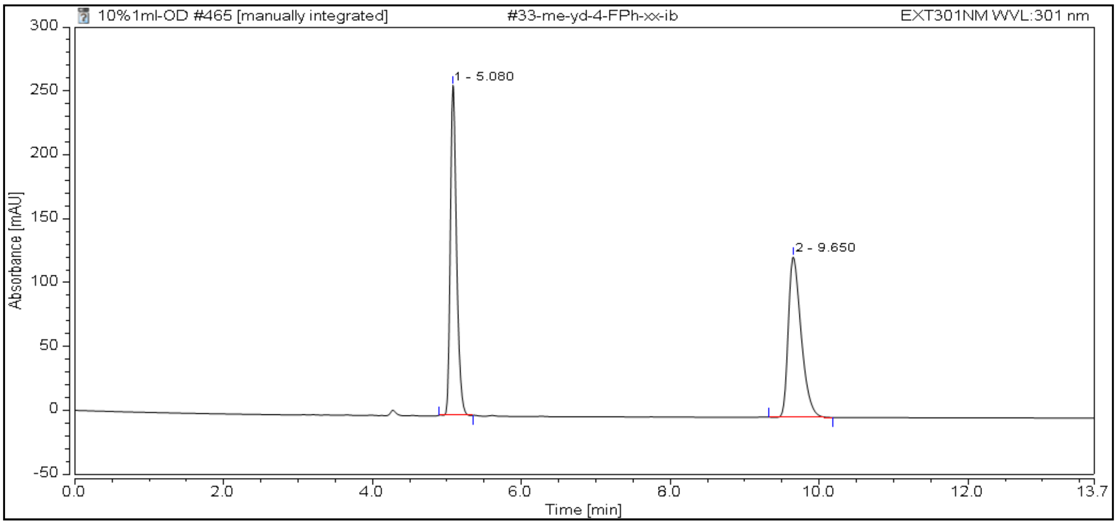

Integration Results					
No.	Peak Name	Retention Time min	Area mAU*min	Relative Area %	Amount n.a.
1		5.080	25.002	49.94	n.a.
2		9.650	25.058	50.06	n.a.
**Total:**			**50.060**	**100.00**	

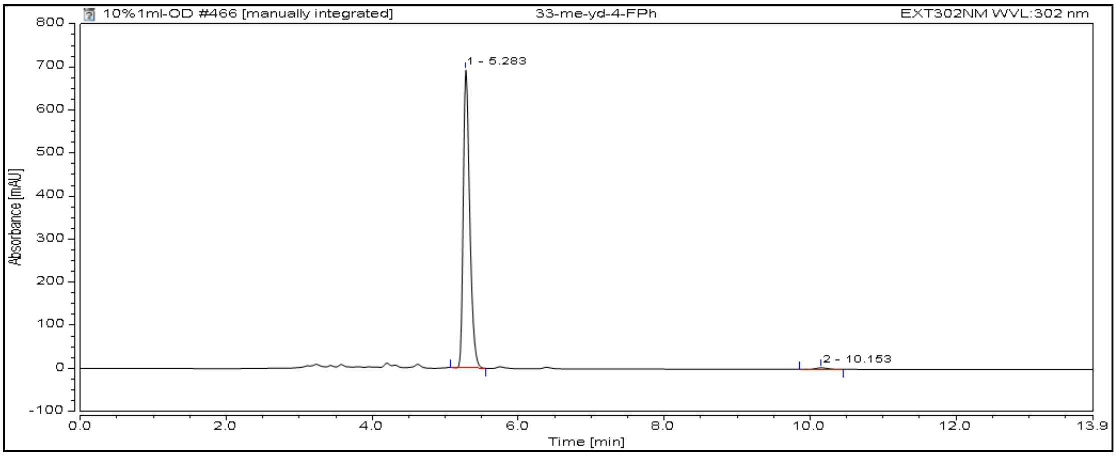

Integration Results					
No.	Peak Name	Retention Time min	Area mAU*min	Relative Area %	Amount n.a.
1		5.283	70.995	99.03	n.a.
2		10.153	0.698	0.97	n.a.
**Total:**			**71.693**	**100.00**	

Supplementary figure 99. HPLC chromatogram for **1b**

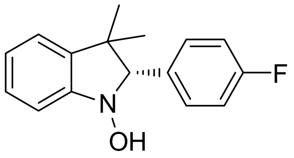

2b

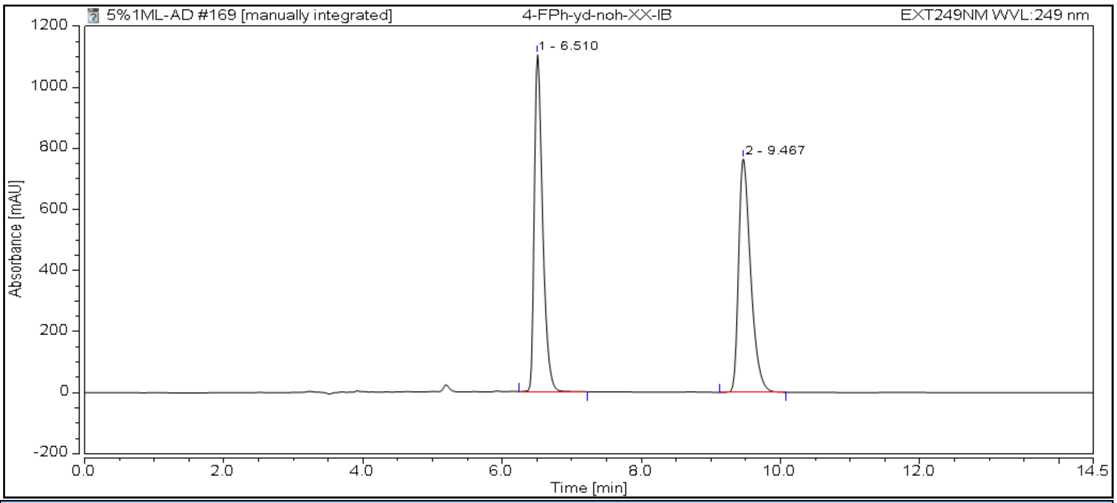

Integration Results

No.	Peak Name	Retention Time min	Area mAU*min	Relative Area %	Amount n.a.
1		6.510	153.164	49.96	n.a.
2		9.467	153.410	50.04	n.a.
**Total:**			**306.574**	**100.00**	

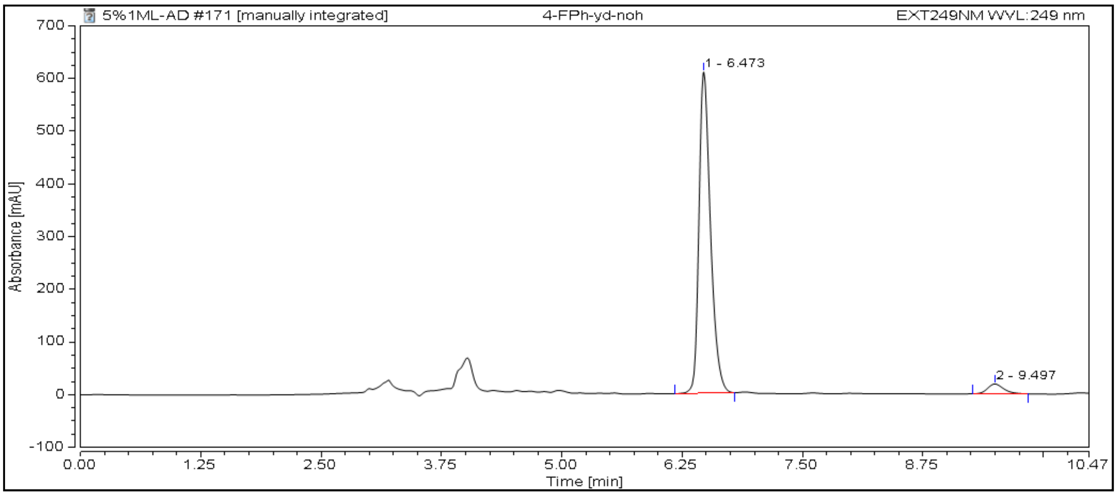

Integration Results

No.	Peak Name	Retention Time min	Area mAU*min	Relative Area %	Amount n.a.
1		6.473	84.530	95.89	n.a.
2		9.497	3.626	4.11	n.a.
**Total:**			**88.156**	**100.00**	

Supplementary figure 100. HPLC chromatogram for **2b**

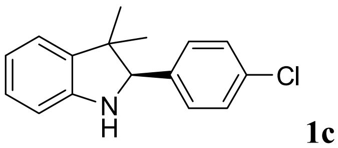

1c

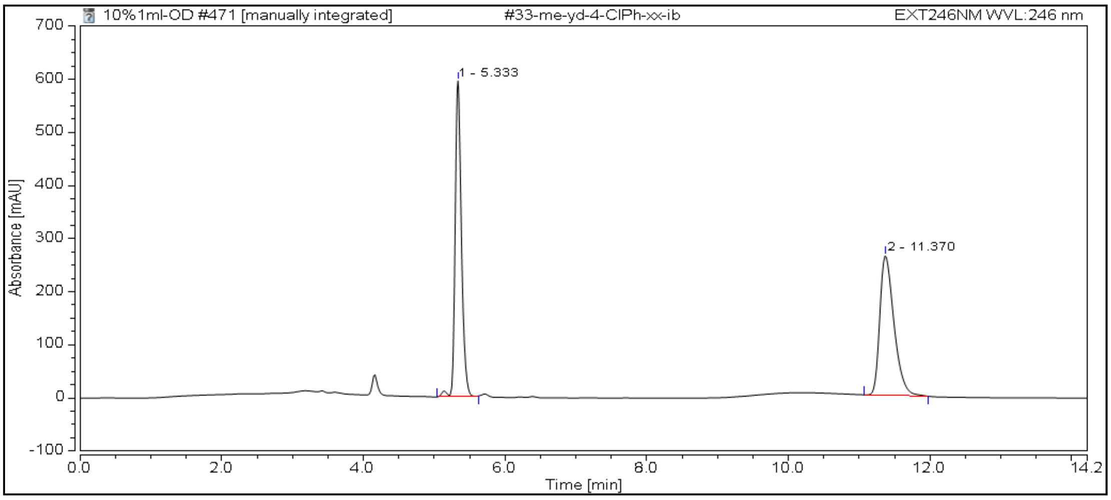

Integration Results

No.	Peak Name	Retention Time min	Area mAU*min	Relative Area %	Amount n.a.
1		5.333	60.718	49.83	n.a.
2		11.370	61.121	50.17	n.a.
**Total:**			**121.839**	**100.00**	

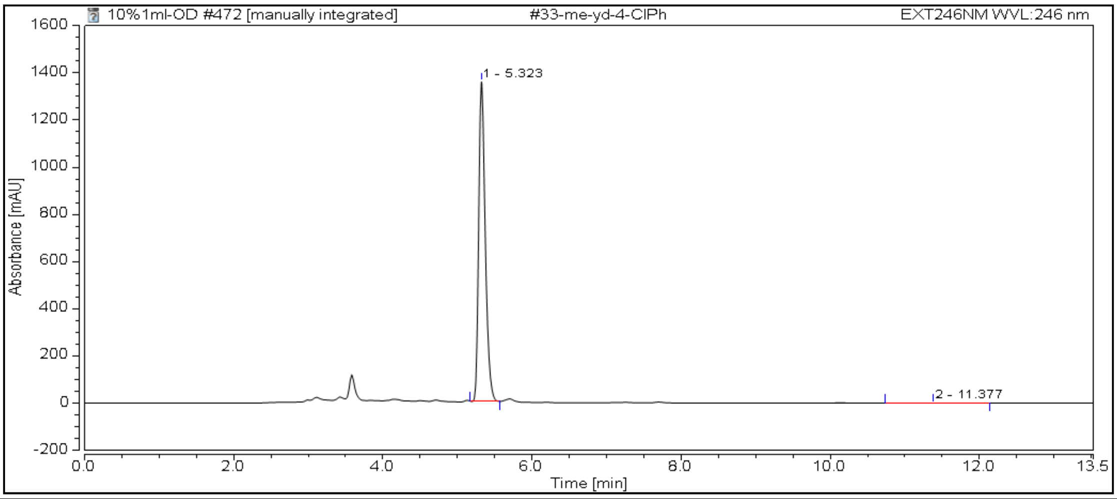

Integration Results

No.	Peak Name	Retention Time min	Area mAU*min	Relative Area %	Amount n.a.
1		5.323	137.407	99.76	n.a.
2		11.377	0.337	0.24	n.a.
**Total:**			**137.744**	**100.00**	

Supplementary figure 101. HPLC chromatogram for **1c**

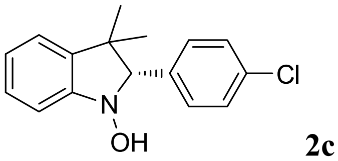

2c

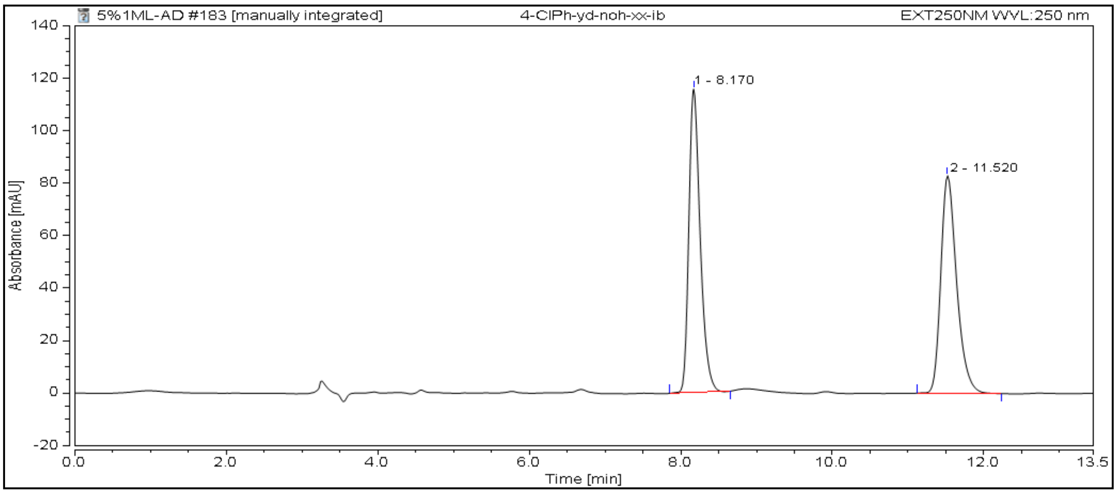

Integration Results

No.	Peak Name	Retention Time min	Area mAU*min	Relative Area %	Amount n.a.
1		8.170	19.997	49.86	n.a.
2		11.520	20.106	50.14	n.a.
**Total:**			**40.103**	**100.00**	

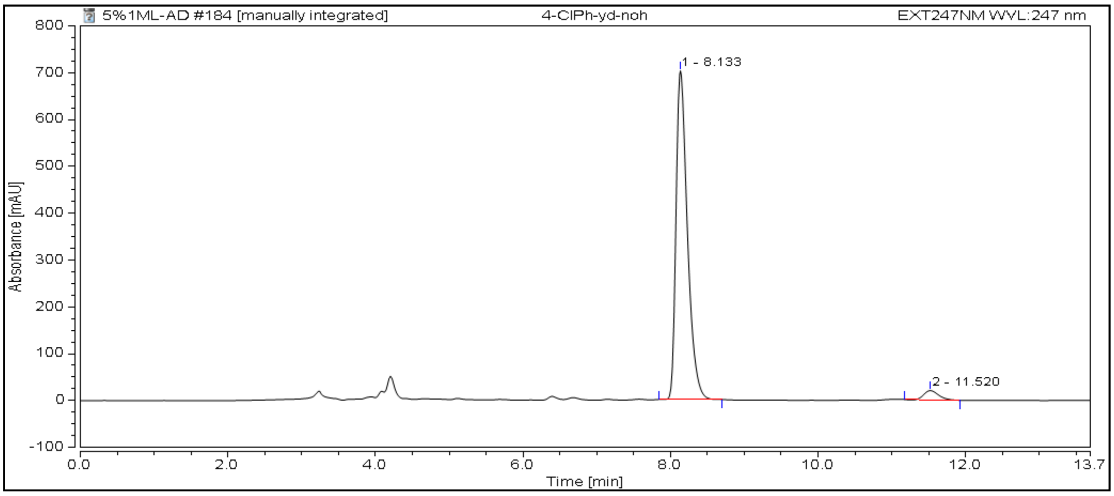

Integration Results

No.	Peak Name	Retention Time min	Area mAU*min	Relative Area %	Amount n.a.
1		8.133	125.378	96.52	n.a.
2		11.520	4.520	3.48	n.a.
**Total:**			**129.898**	**100.00**	

Supplementary figure 102. HPLC chromatogram for **2c**

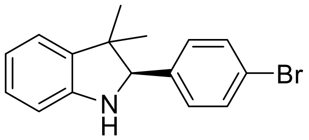

1d

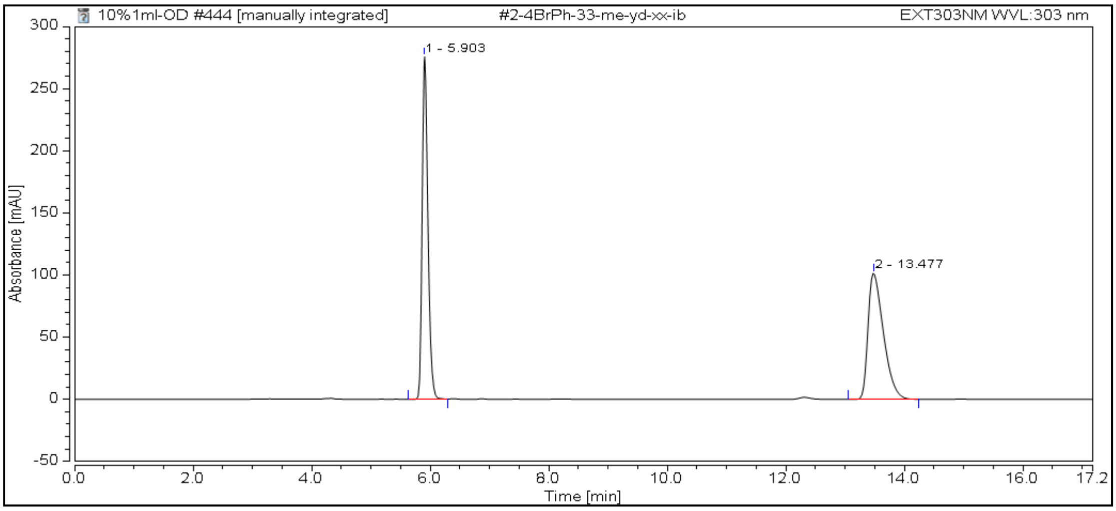

Integration Results

No.	Peak Name	Retention Time min	Area mAU*min	Relative Area %	Amount n.a.
1		5.903	30.797	50.25	n.a.
2		13.477	30.486	49.75	n.a.
**Total:**			**61.283**	**100.00**	

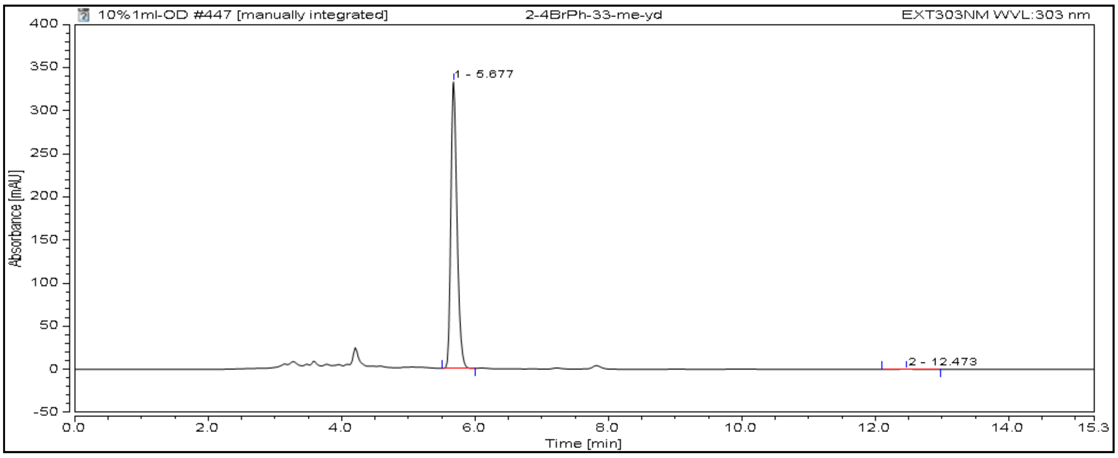

Integration Results

No.	Peak Name	Retention Time min	Area mAU*min	Relative Area %	Amount n.a.
1		5.677	35.570	99.78	n.a.
2		12.473	0.080	0.22	n.a.
**Total:**			**35.650**	**100.00**	

Supplementary figure 103. HPLC chromatogram for **1d**

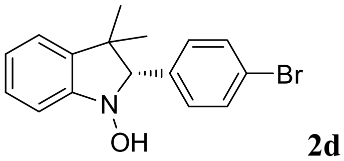

2d

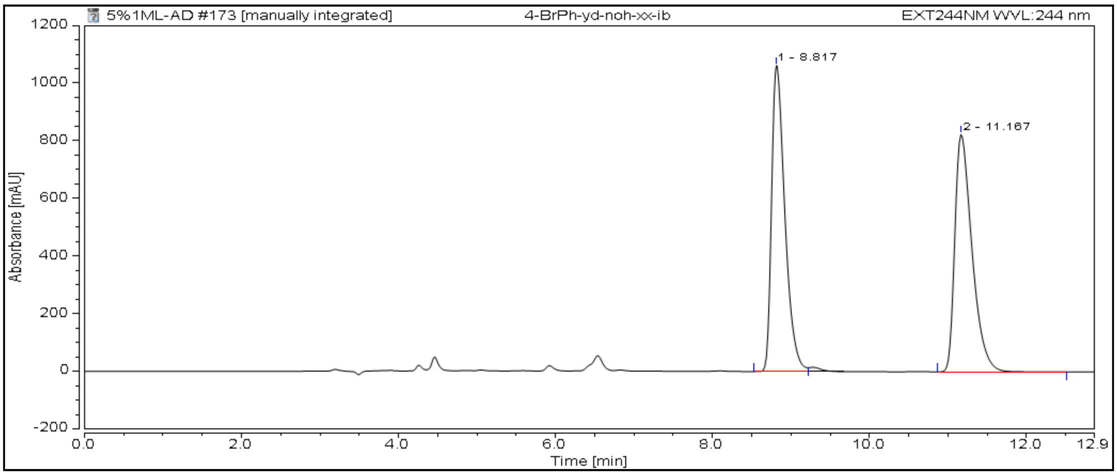

Integration Results					
No.	Peak Name	Retention Time min	Area mAU*min	Relative Area %	Amount n.a.
1		8.817	207.969	49.89	n.a.
2		11.167	208.866	50.11	n.a.
**Total:**			**416.835**	**100.00**	

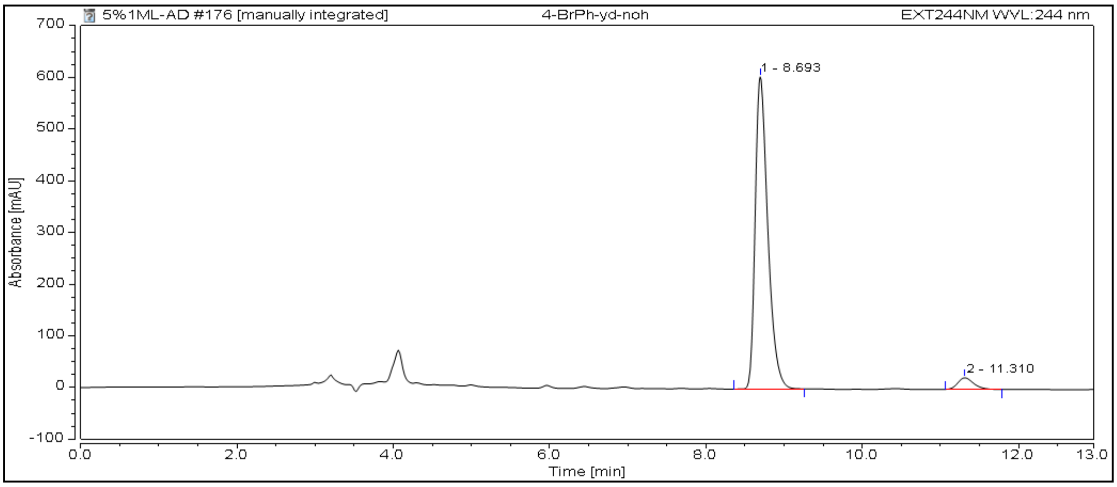

Integration Results					
No.	Peak Name	Retention Time min	Area mAU*min	Relative Area %	Amount n.a.
1		8.693	112.634	95.52	n.a.
2		11.310	5.281	4.48	n.a.
**Total:**			**117.914**	**100.00**	

Supplementary figure 104. HPLC chromatogram for **2d**

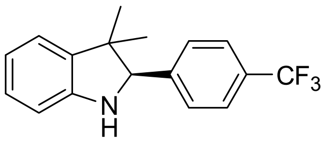

1e

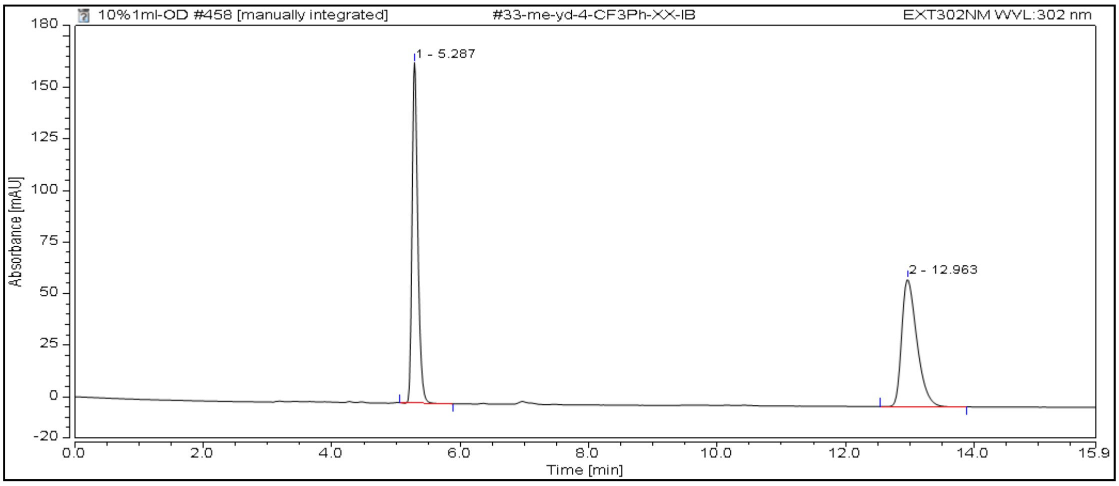

Integration Results					
No.	Peak Name	Retention Time min	Area mAU*min	Relative Area %	Amount n.a.
1		5.287	16.987	50.01	n.a.
2		12.963	16.977	49.99	n.a.
**Total:**			**33.964**	**100.00**	

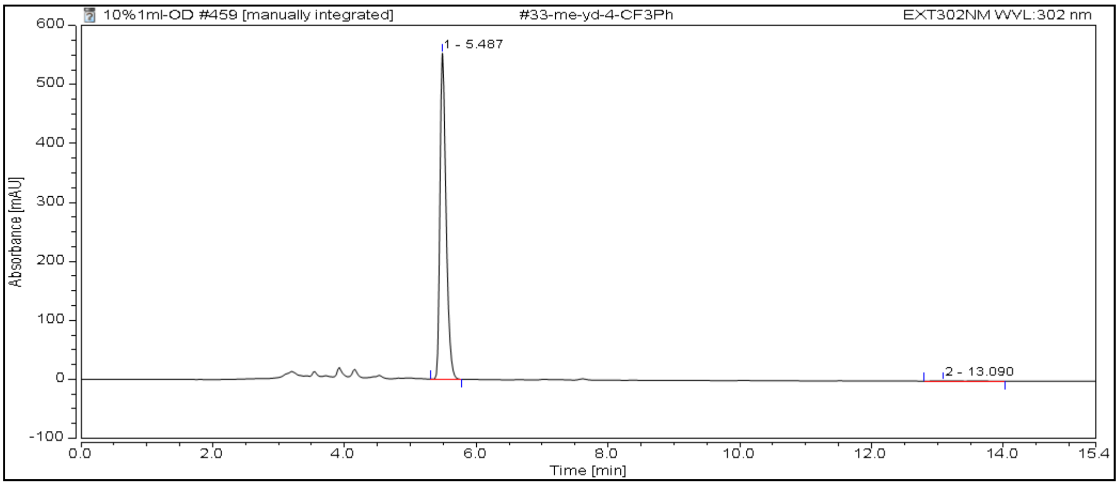

Integration Results					
No.	Peak Name	Retention Time min	Area mAU*min	Relative Area %	Amount n.a.
1		5.487	59.360	99.59	n.a.
2		13.090	0.242	0.41	n.a.
**Total:**			**59.603**	**100.00**	

Supplementary figure 105. HPLC chromatogram for **1e**

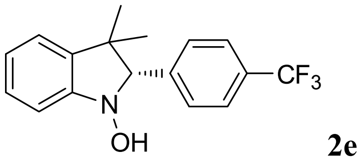

2e

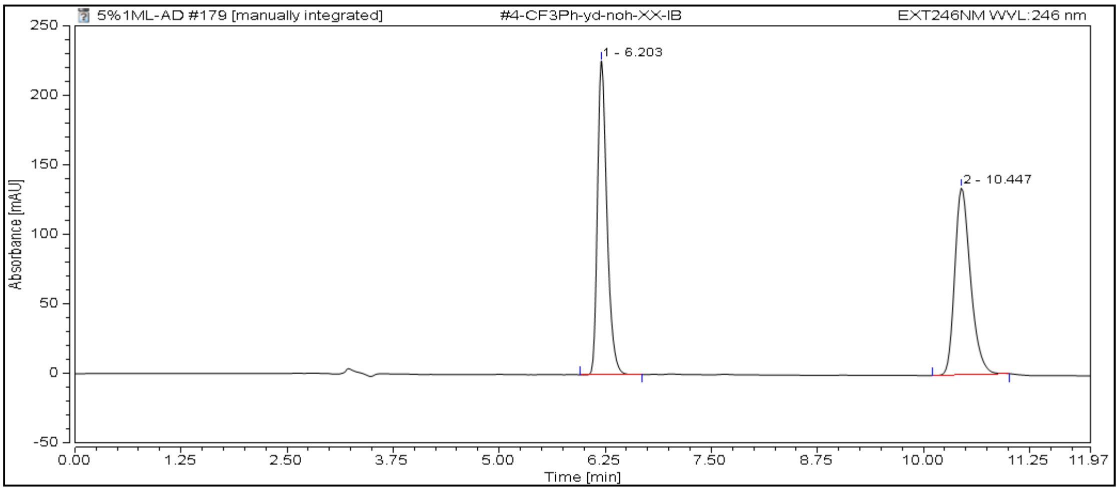

Integration Results					
No.	Peak Name	Retention Time min	Area mAU*min	Relative Area %	Amount n.a.
1		6.203	29.149	50.20	n.a.
2		10.447	28.912	49.80	n.a.
**Total:**			**58.061**	**100.00**	

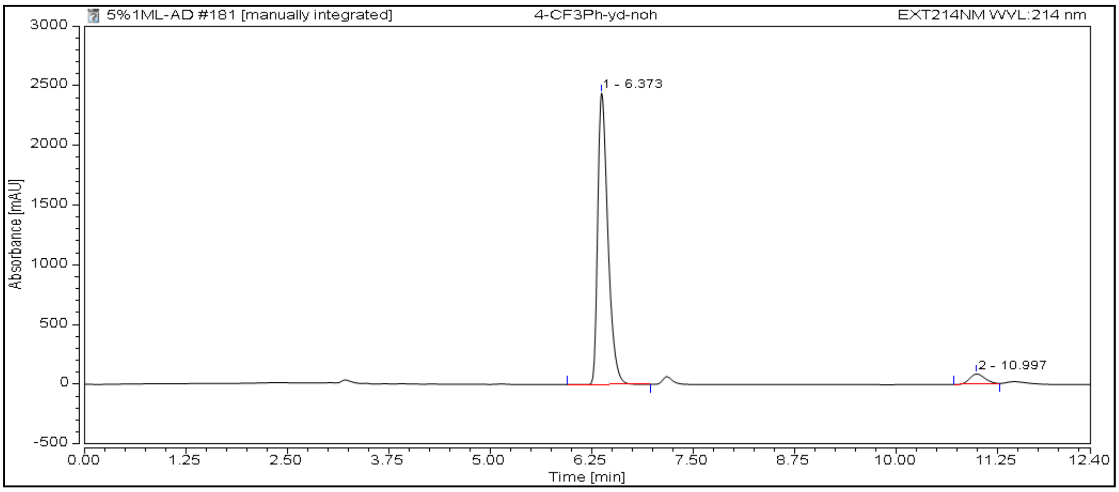

Integration Results					
No.	Peak Name	Retention Time min	Area mAU*min	Relative Area %	Amount n.a.
1		6.373	354.615	95.14	n.a.
2		10.997	18.118	4.86	n.a.
**Total:**			**372.733**	**100.00**	

Supplementary figure 106. HPLC chromatogram for **2e**

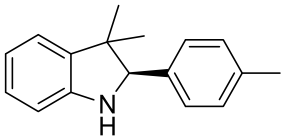

1f

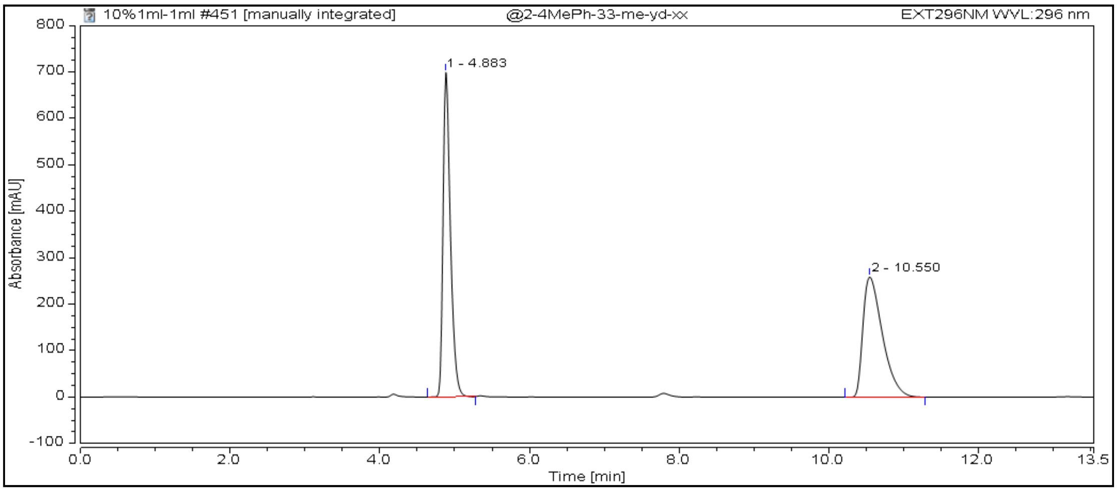

Integration Results					
No.	Peak Name	Retention Time min	Area mAU*min	Relative Area %	Amount n.a.
1		4.883	79.728	50.58	n.a.
2		10.550	77.910	49.42	n.a.
**Total:**			**157.637**	**100.00**	

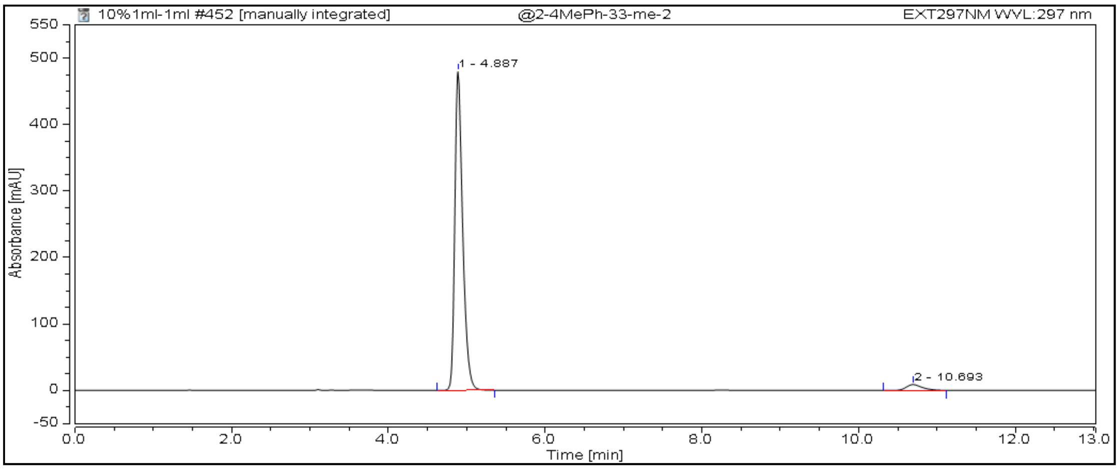

Integration Results					
No.	Peak Name	Retention Time min	Area mAU*min	Relative Area %	Amount n.a.
1		4.887	55.791	96.42	n.a.
2		10.693	2.070	3.58	n.a.
**Total:**			**57.861**	**100.00**	

Supplementary figure 107. HPLC chromatogram for **1f**

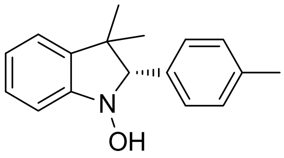

2f

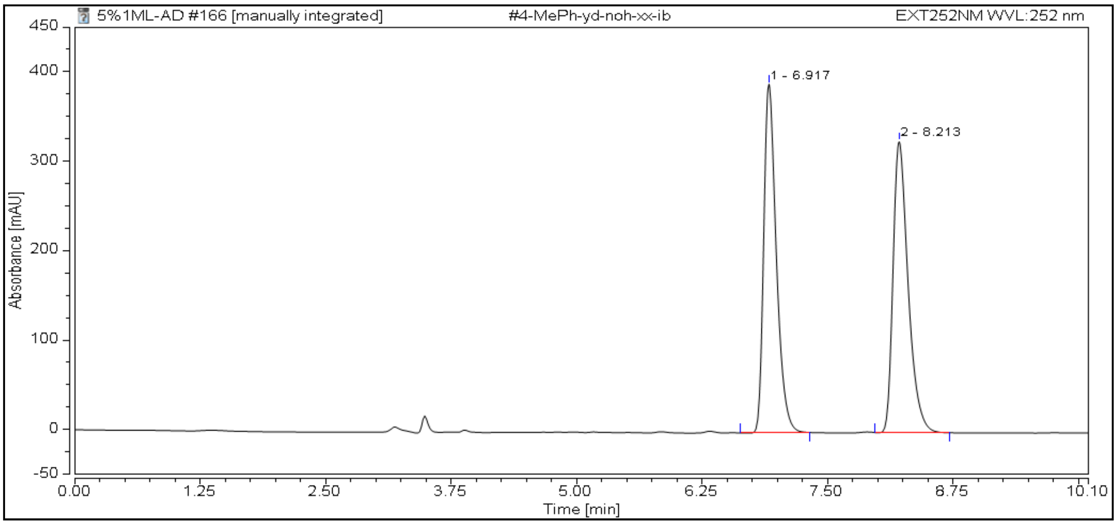

Integration Results					
No.	Peak Name	Retention Time min	Area mAU*min	Relative Area %	Amount n.a.
1		6.917	56.468	50.02	n.a.
2		8.213	56.413	49.98	n.a.
**Total:**			**112.881**	**100.00**	

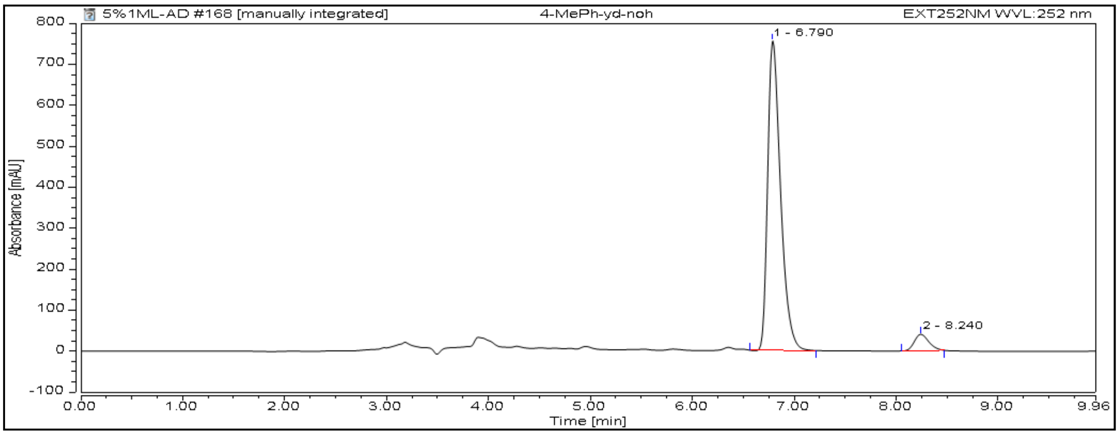

Integration Results					
No.	Peak Name	Retention Time min	Area mAU*min	Relative Area %	Amount n.a.
1		6.790	110.757	94.39	n.a.
2		8.240	6.578	5.61	n.a.
**Total:**			**117.335**	**100.00**	

Supplementary figure 108. HPLC chromatogram for **2f**

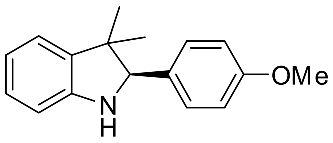

1g

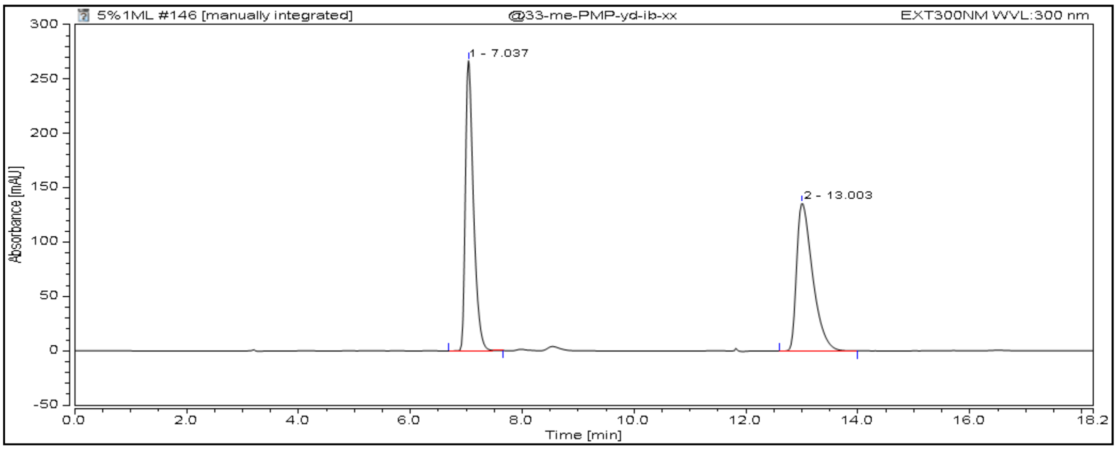

Integration Results					
No.	Peak Name	Retention Time min	Area mAU*min	Relative Area %	Amount n.a.
1		7.037	44.651	49.84	n.a.
2		13.003	44.931	50.16	n.a.
**Total:**			**89.582**	**100.00**	

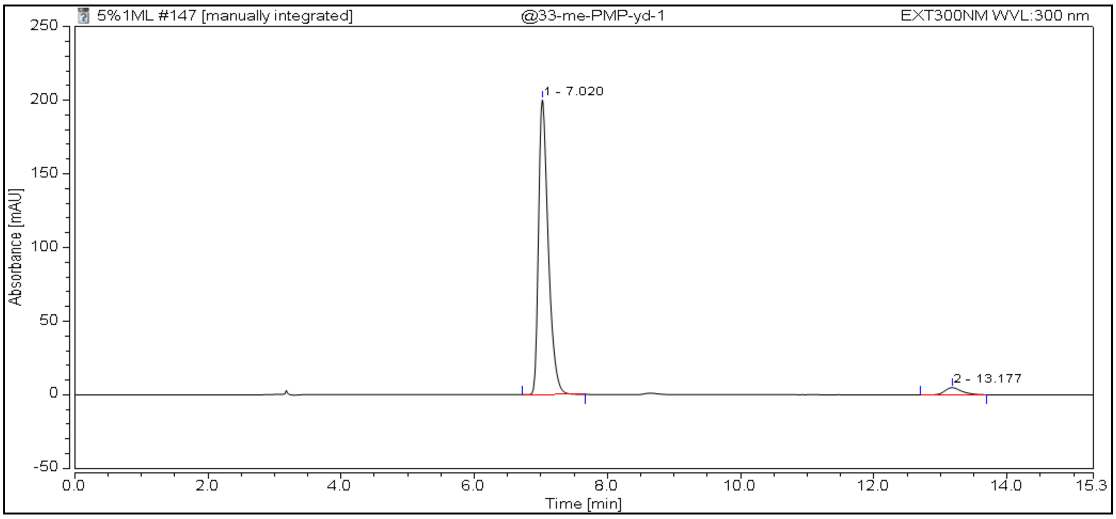

Integration Results					
No.	Peak Name	Retention Time min	Area mAU*min	Relative Area %	Amount n.a.
1		7.020	33.946	96.00	n.a.
2		13.177	1.413	4.00	n.a.
**Total:**			**35.359**	**100.00**	

Supplementary figure 109. HPLC chromatogram for **1g**

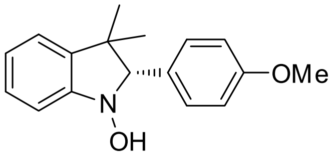

2g

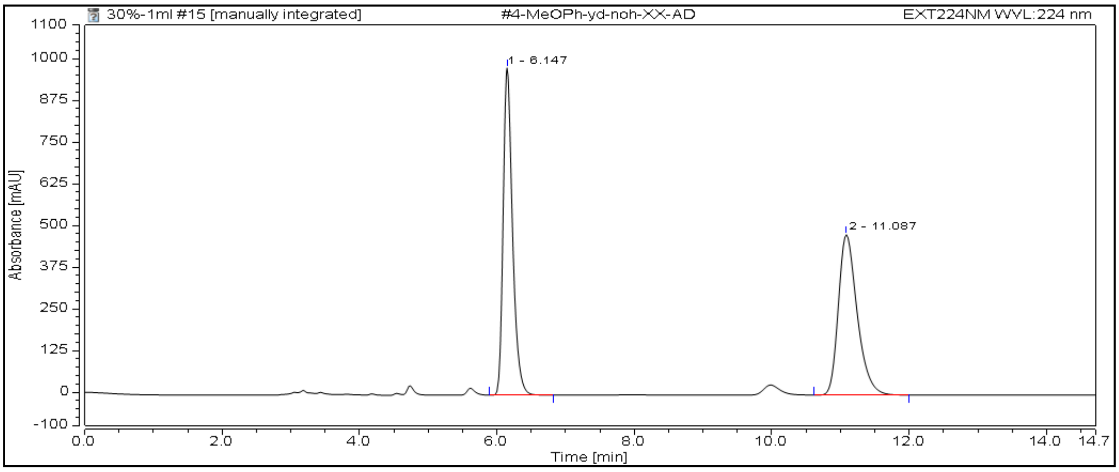

Integration Results					
No.	Peak Name	Retention Time min	Area mAU*min	Relative Area %	Amount n.a.
1		6.147	154.105	50.26	n.a.
2		11.087	152.499	49.74	n.a.
**Total:**			**306.604**	**100.00**	

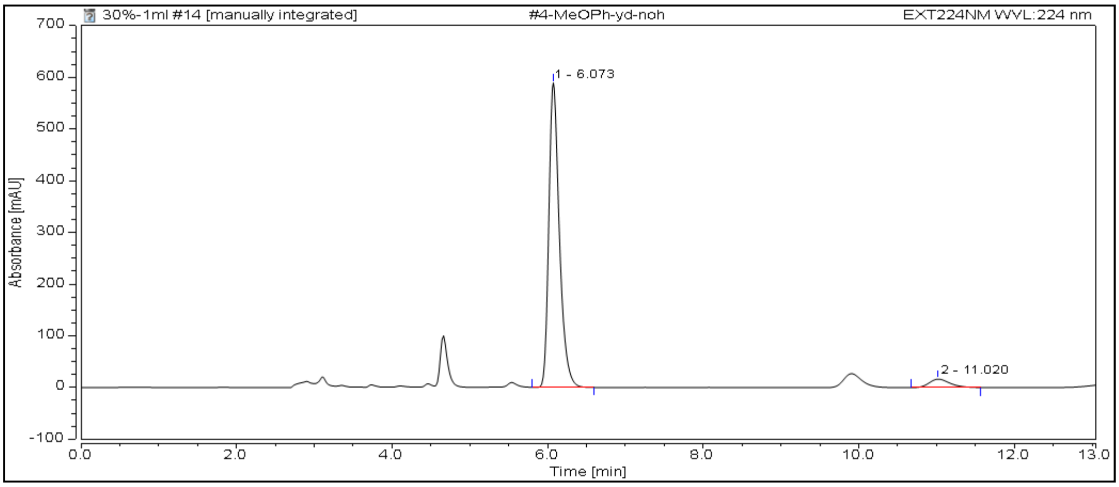

Integration Results					
No.	Peak Name	Retention Time min	Area mAU*min	Relative Area %	Amount n.a.
1		6.073	92.422	94.80	n.a.
2		11.020	5.073	5.20	n.a.
**Total:**			**97.495**	**100.00**	

Supplementary figure 110. HPLC chromatogram for **2g**

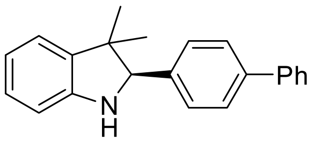

1h

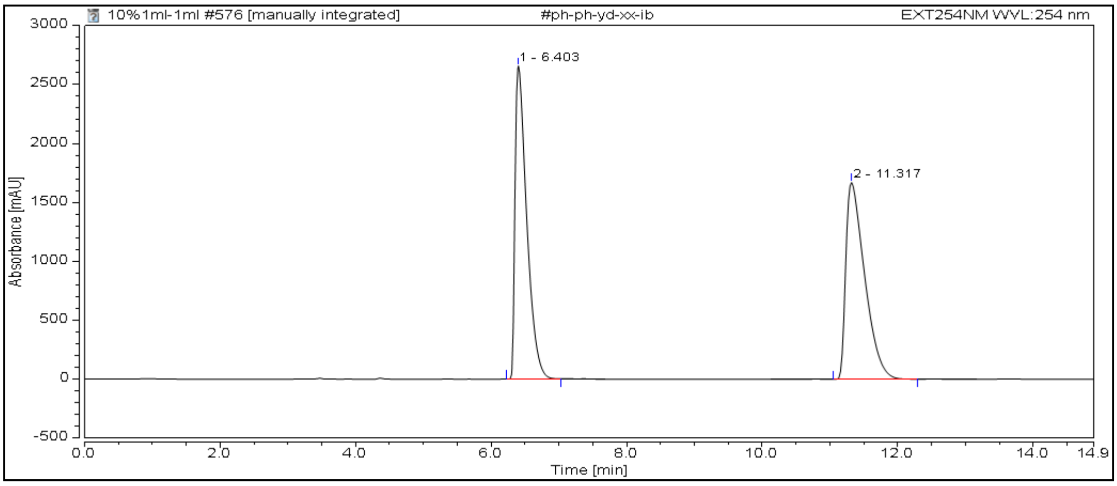

Integration Results					
No.	Peak Name	Retention Time min	Area mAU*min	Relative Area %	Amount n.a.
1		6.403	530.583	49.67	n.a.
2		11.317	537.645	50.33	n.a.
**Total:**			**1068.228**	**100.00**	

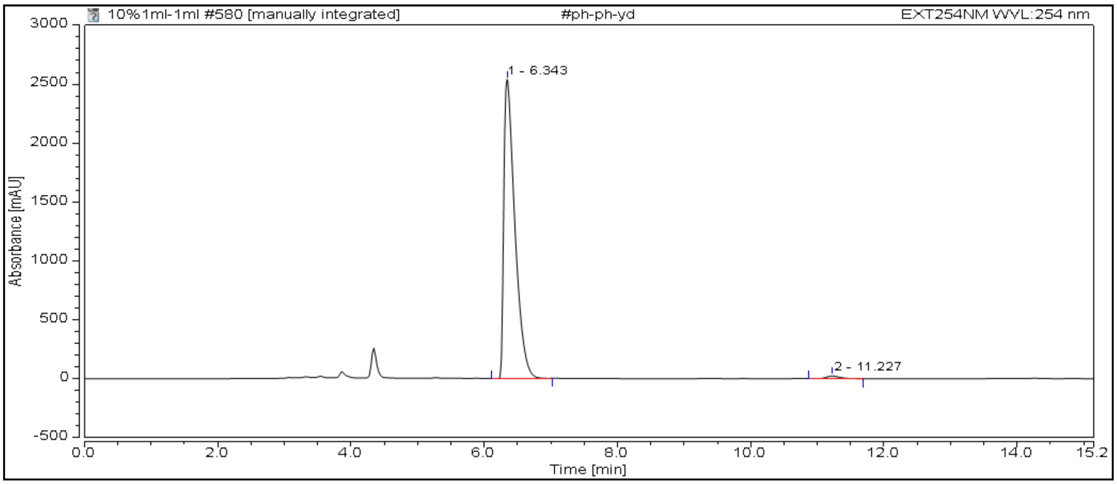

Integration Results					
No.	Peak Name	Retention Time min	Area mAU*min	Relative Area %	Amount n.a.
1		6.343	486.699	98.93	n.a.
2		11.227	5.250	1.07	n.a.
**Total:**			**491.949**	**100.00**	

Supplementary figure 111. HPLC chromatogram for **1h**

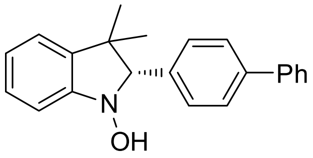

2h

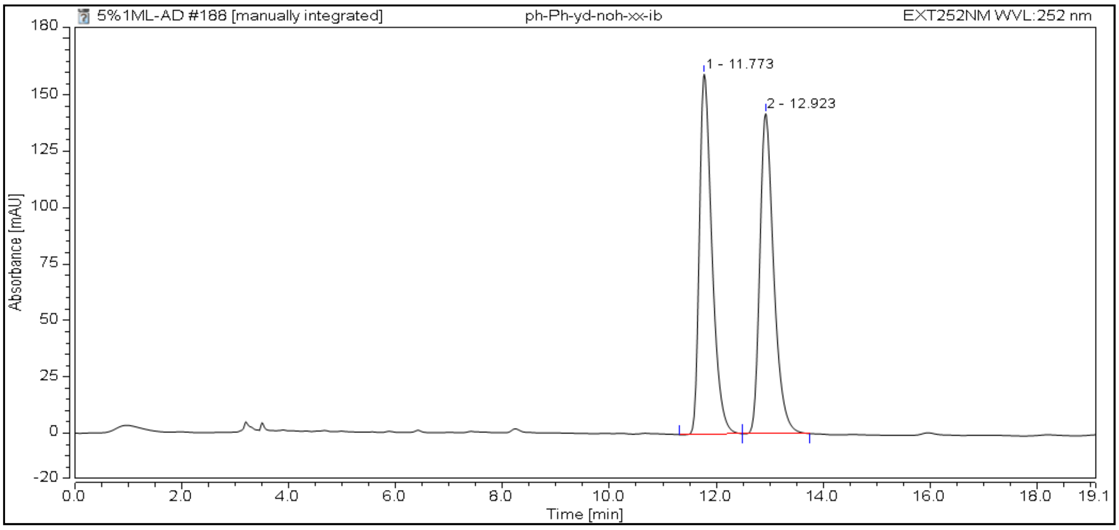

Integration Results					
No.	Peak Name	Retention Time min	Area mAU*min	Relative Area %	Amount n.a.
1		11.773	43.492	50.36	n.a.
2		12.923	42.873	49.64	n.a.
**Total:**			**86.366**	**100.00**	

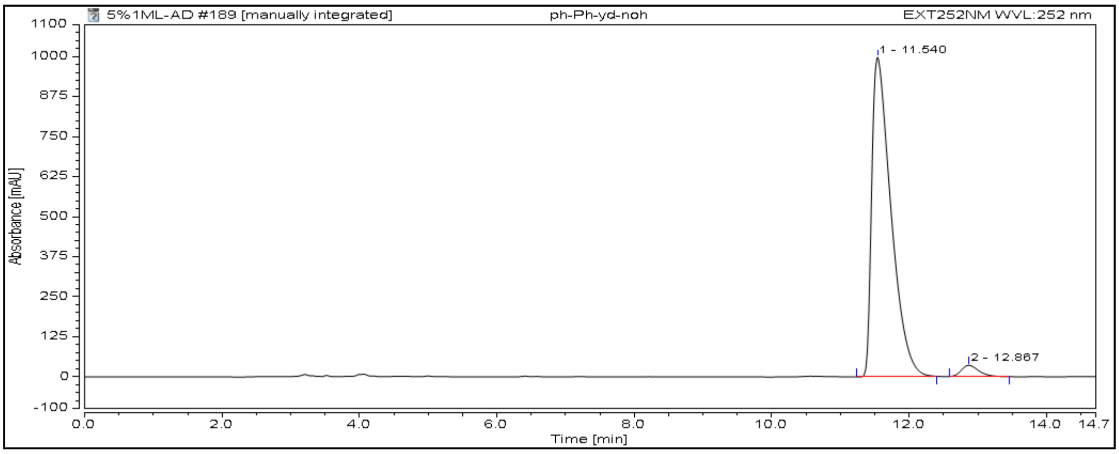

Integration Results					
No.	Peak Name	Retention Time min	Area mAU*min	Relative Area %	Amount n.a.
1		11.540	323.529	96.89	n.a.
2		12.867	10.394	3.11	n.a.
**Total:**			**333.923**	**100.00**	

Supplementary figure 112. HPLC chromatogram for **2h**

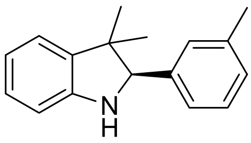

1i

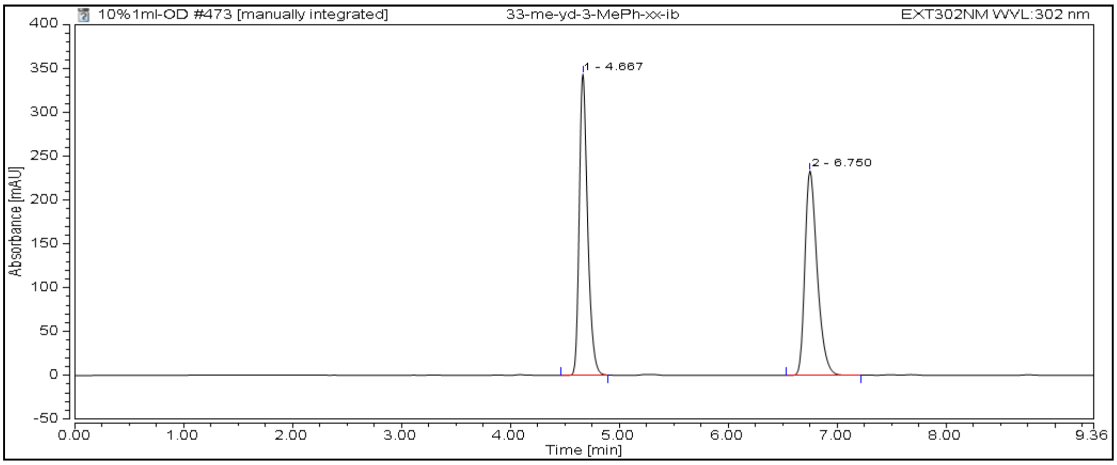

Integration Results					
No.	Peak Name	Retention Time min	Area mAU*min	Relative Area %	Amount n.a.
1		4.667	30.506	50.08	n.a.
2		6.750	30.409	49.92	n.a.
**Total:**			**60.914**	**100.00**	

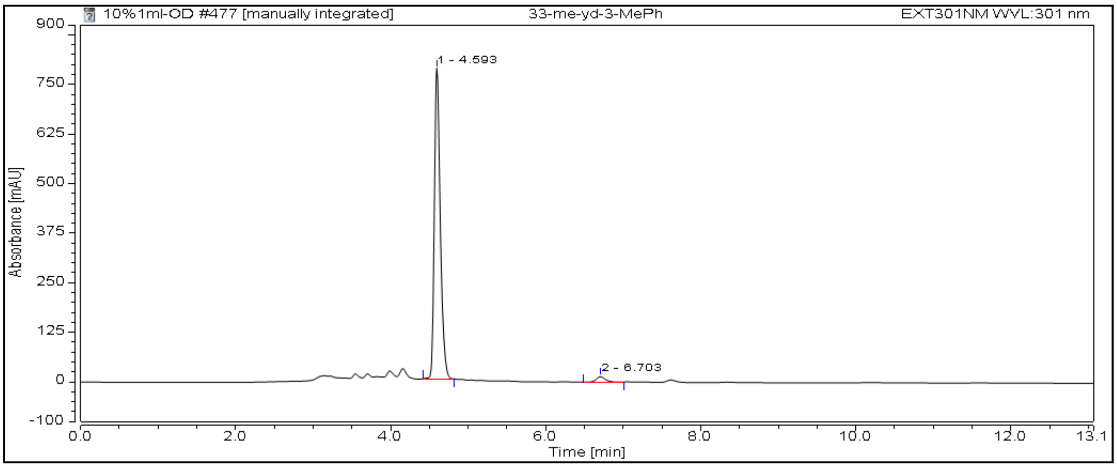

Integration Results					
No.	Peak Name	Retention Time min	Area mAU*min	Relative Area %	Amount n.a.
1		4.593	69.254	97.47	n.a.
2		6.703	1.797	2.53	n.a.
**Total:**			**71.051**	**100.00**	

Supplementary figure 113. HPLC chromatogram for **1i**

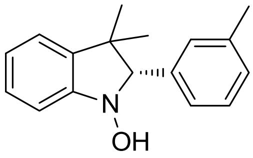

2i

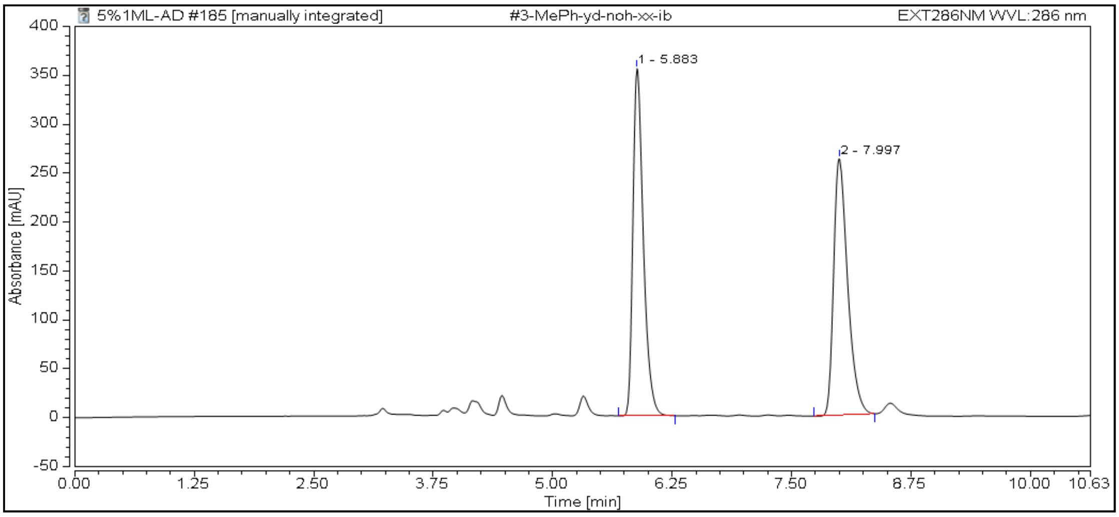

Integration Results					
No.	Peak Name	Retention Time min	Area mAU*min	Relative Area %	Amount n.a.
1		5.883	44.863	50.26	n.a.
2		7.997	44.397	49.74	n.a.
**Total:**			**89.261**	**100.00**	

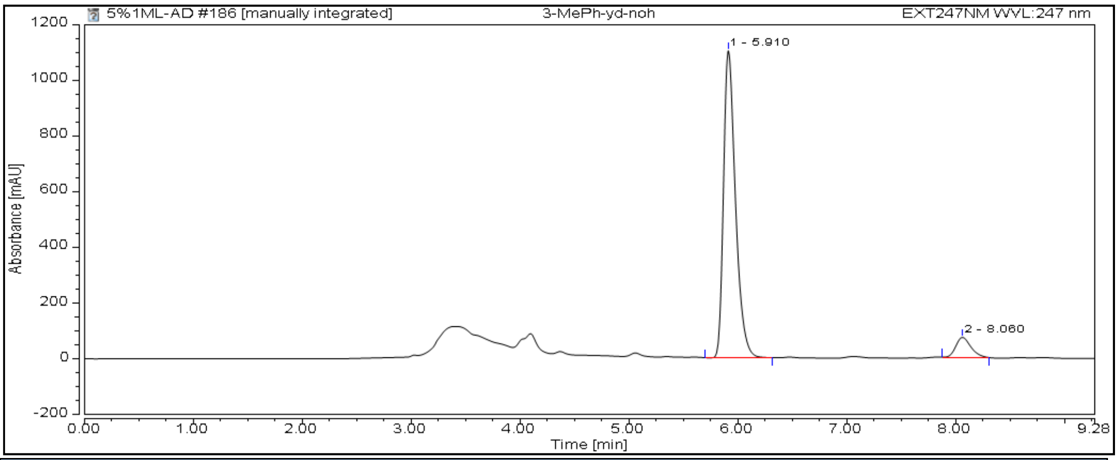

Integration Results					
No.	Peak Name	Retention Time min	Area mAU*min	Relative Area %	Amount n.a.
1		5.910	139.967	92.44	n.a.
2		8.060	11.440	7.56	n.a.
**Total:**			**151.407**	**100.00**	

Supplementary figure 114. HPLC chromatogram for **2i**

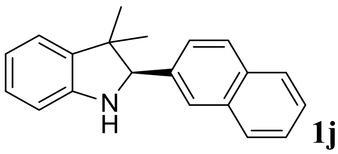

1j

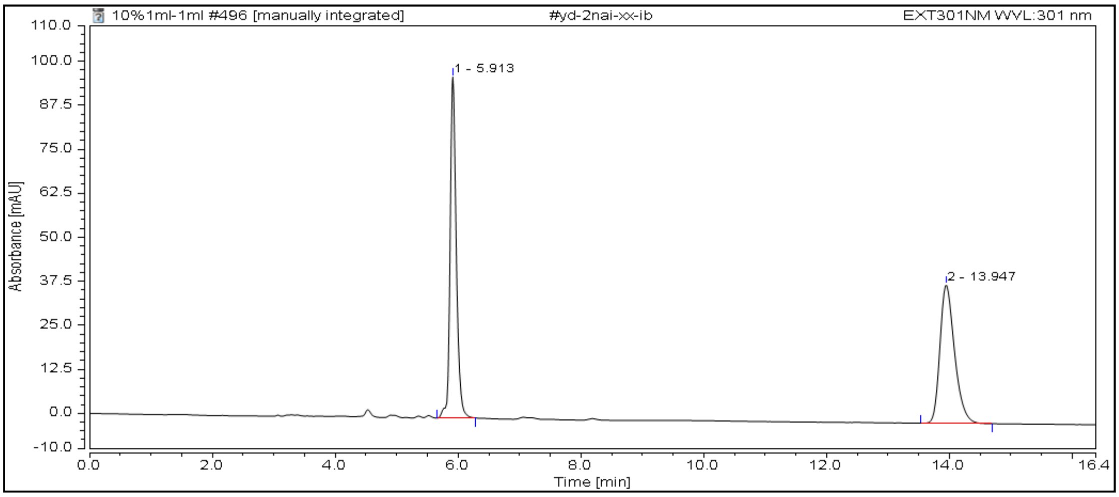

Integration Results					
No.	Peak Name	Retention Time min	Area mAU*min	Relative Area %	Amount n.a.
1		5.913	11.201	50.79	n.a.
2		13.947	10.851	49.21	n.a.
**Total:**			**22.051**	**100.00**	

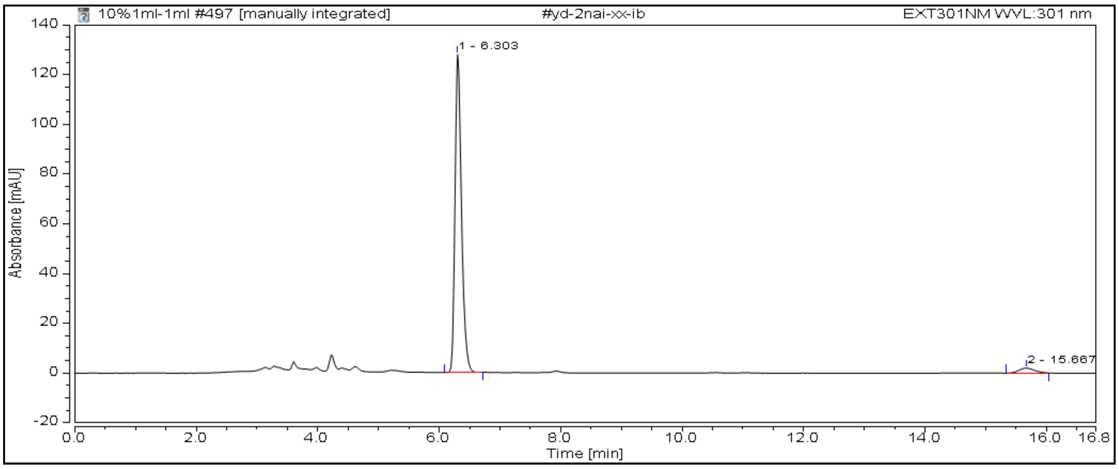

Integration Results					
No.	Peak Name	Retention Time min	Area mAU*min	Relative Area %	Amount n.a.
1		6.303	15.754	96.35	n.a.
2		15.667	0.597	3.65	n.a.
**Total:**			**16.351**	**100.00**	

Supplementary figure 115. HPLC chromatogram for **1j**

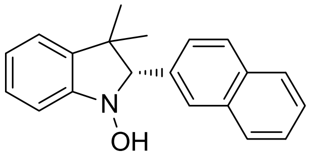

2j

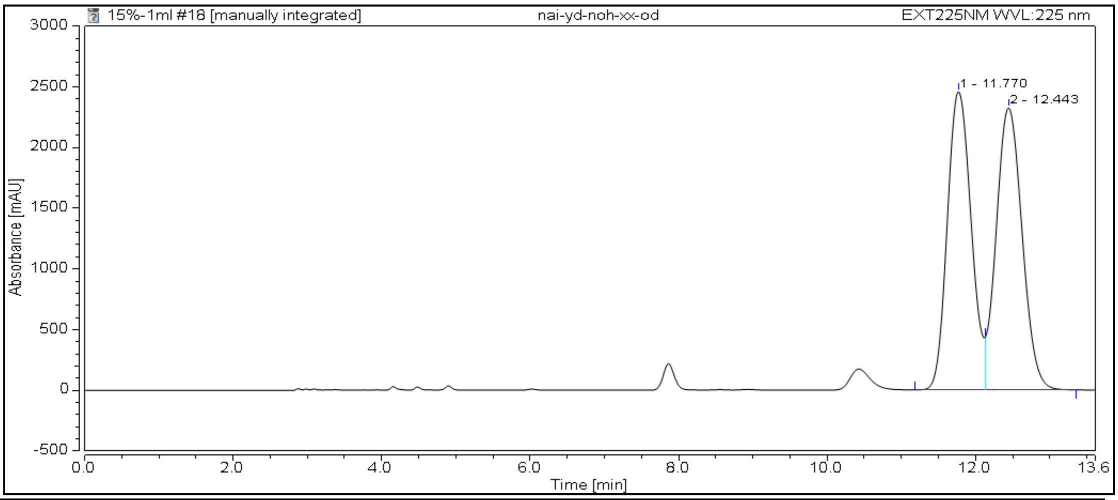

Integration Results					
No.	Peak Name	Retention Time min	Area mAU*min	Relative Area %	Amount n.a.
1		11.770	910.033	49.08	n.a.
2		12.443	944.282	50.92	n.a.
**Total:**			**1854.315**	**100.00**	

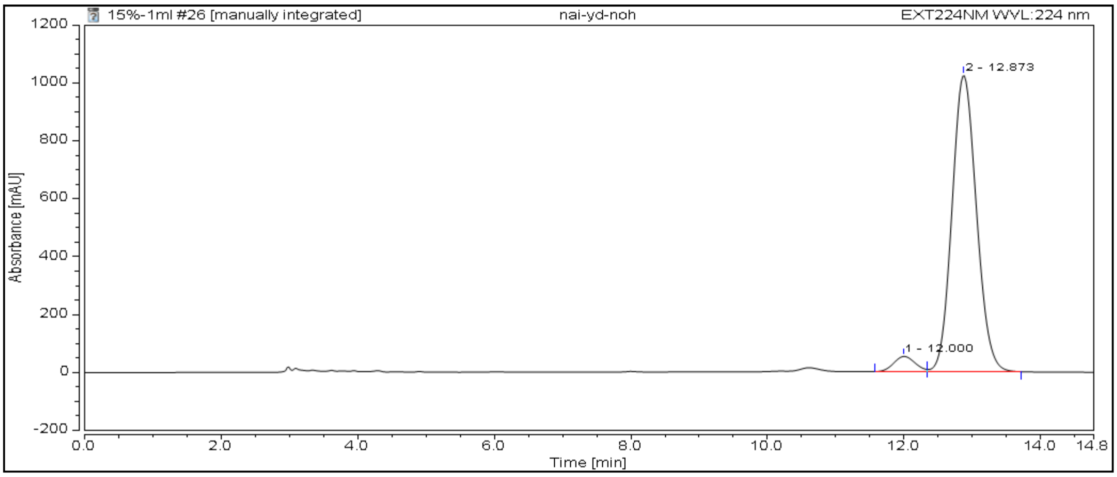

Integration Results					
No.	Peak Name	Retention Time min	Area mAU*min	Relative Area %	Amount n.a.
1		12.000	20.014	4.42	n.a.
2		12.873	432.900	95.58	n.a.
**Total:**			**452.914**	**100.00**	

Supplementary figure 116. HPLC chromatogram for **2j**

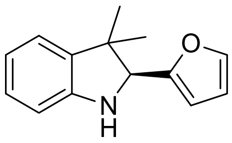

1k

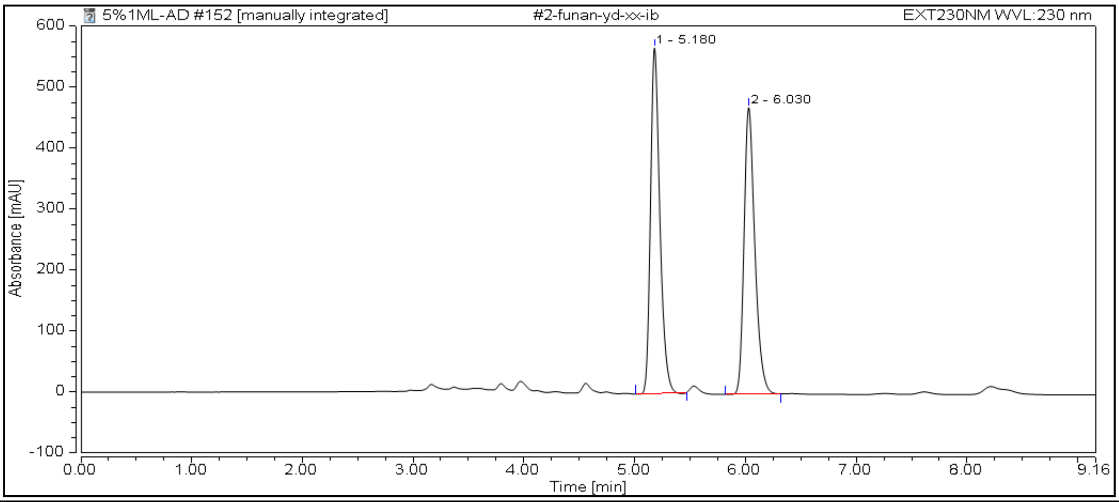

Integration Results

No.	Peak Name	Retention Time min	Area mAU*min	Relative Area %	Amount n.a.
1		5.180	55.422	50.99	n.a.
2		6.030	53.260	49.01	n.a.
**Total:**			**108.682**	**100.00**	

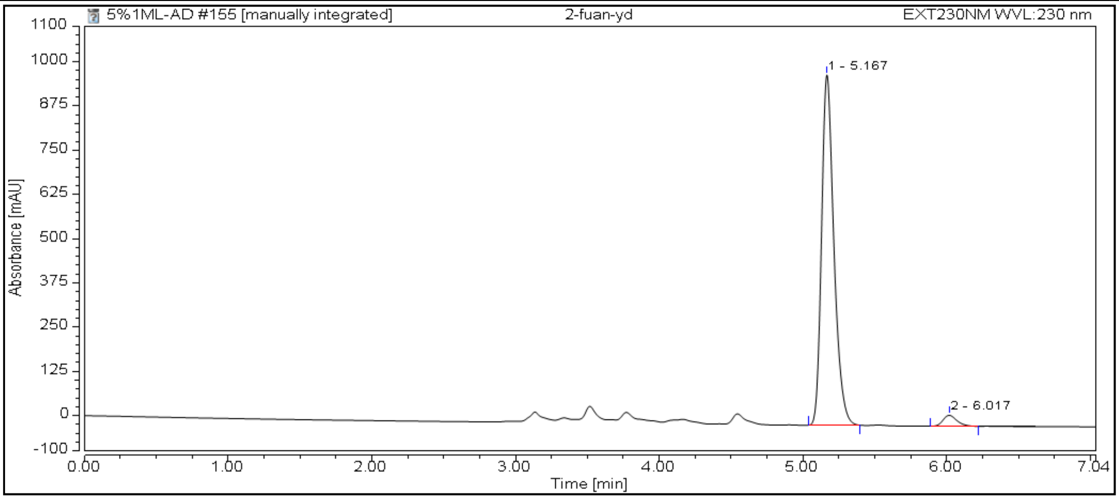

Integration Results

No.	Peak Name	Retention Time min	Area mAU*min	Relative Area %	Amount n.a.
1		5.167	98.903	96.54	n.a.
2		6.017	3.548	3.46	n.a.
**Total:**			**102.452**	**100.00**	

Supplementary figure 117. HPLC chromatogram for **1k**

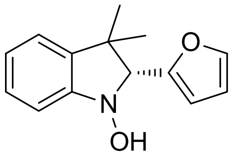

2k

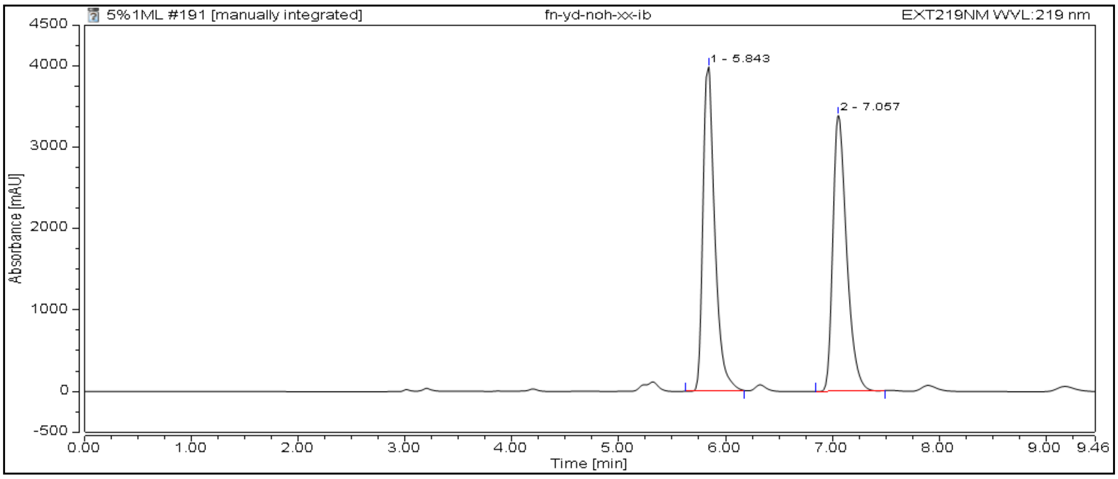

**Integration Results**					
No.	Peak Name	Retention Time min	Area mAU*min	Relative Area %	Amount n.a.
1		5.843	509.914	50.22	n.a.
2		7.057	505.436	49.78	n.a.
**Total:**			**1015.349**	**100.00**	

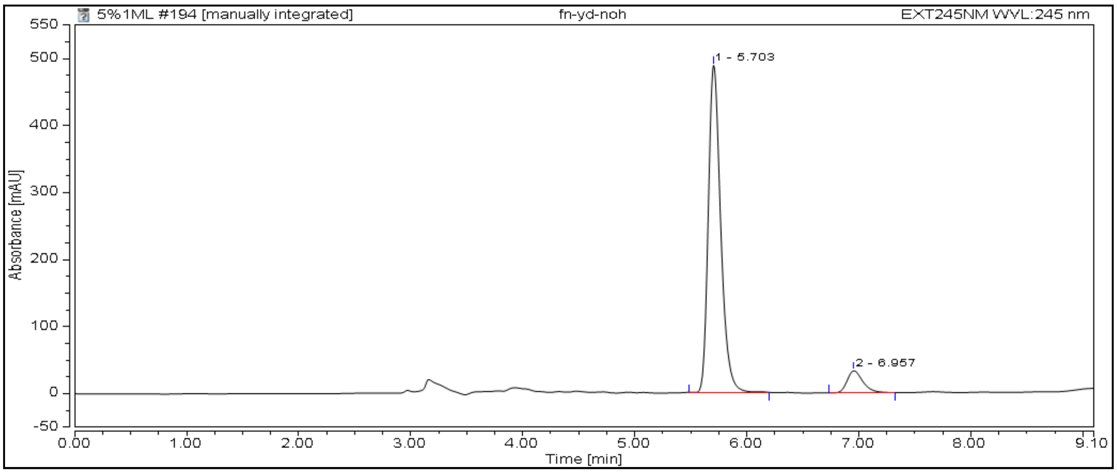

**Integration Results**					
No.	Peak Name	Retention Time min	Area mAU*min	Relative Area %	Amount n.a.
1		5.703	62.461	92.07	n.a.
2		6.957	5.376	7.93	n.a.
**Total:**			**67.837**	**100.00**	

Supplementary figure 118. HPLC chromatogram for **2k**

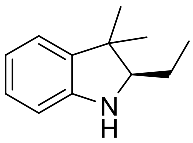

1l

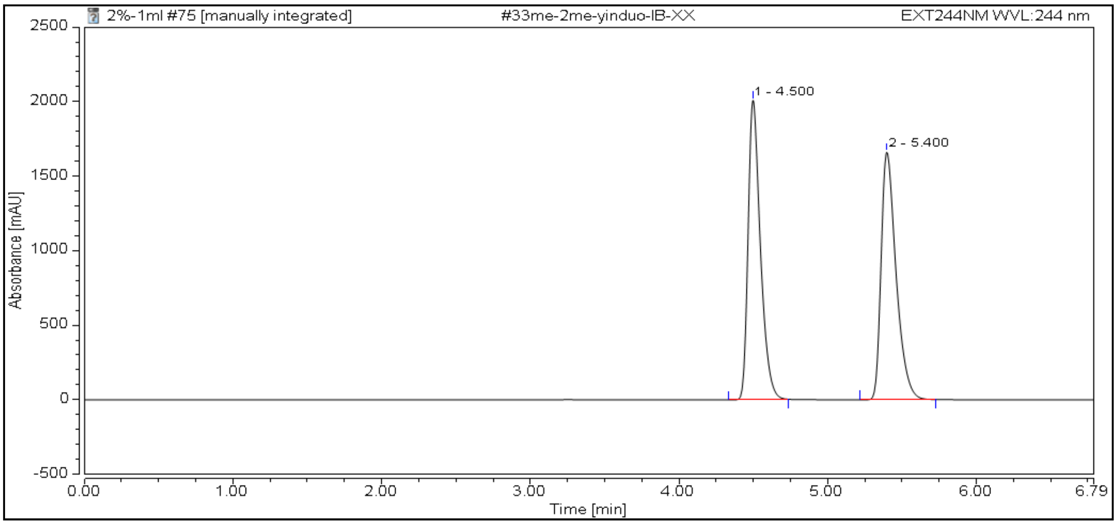

Integration Results					
No.	Peak Name	Retention Time min	Area mAU*min	Relative Area %	Amount n.a.
1		4.500	192.155	49.85	n.a.
2		5.400	193.349	50.15	n.a.
**Total:**			**385.504**	**100.00**	

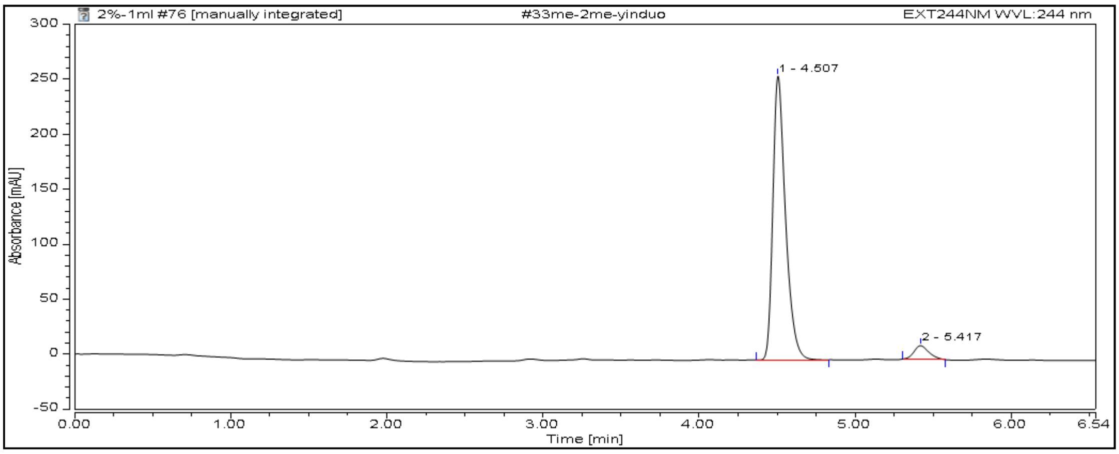

Integration Results					
No.	Peak Name	Retention Time min	Area mAU*min	Relative Area %	Amount n.a.
1		4.507	24.152	94.62	n.a.
2		5.417	1.375	5.38	n.a.
**Total:**			**25.527**	**100.00**	

Supplementary figure 119. HPLC chromatogram for **1l**

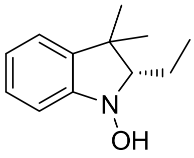

2l

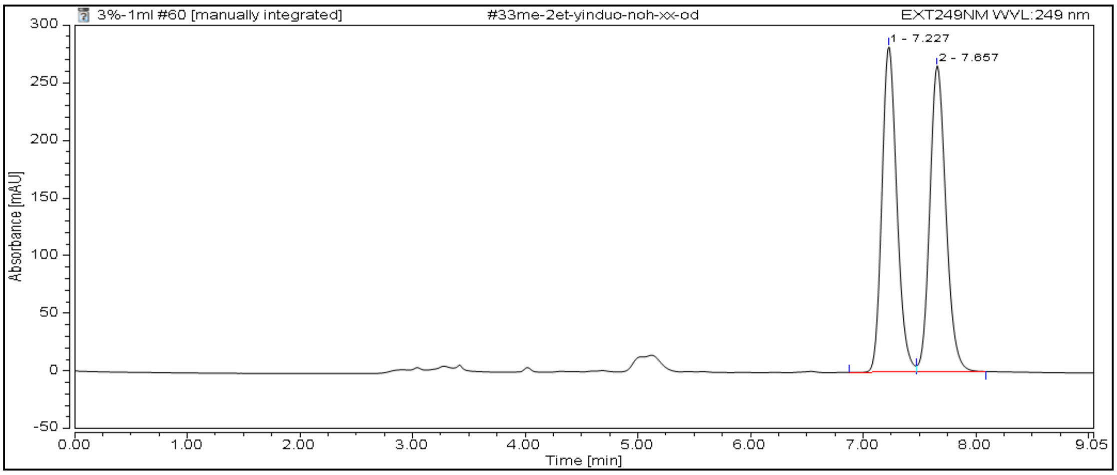

Integration Results					
No.	Peak Name	Retention Time min	Area mAU*min	Relative Area %	Amount n.a.
1		7.227	43.390	49.64	n.a.
2		7.657	44.022	50.36	n.a.
**Total:**			**87.412**	**100.00**	

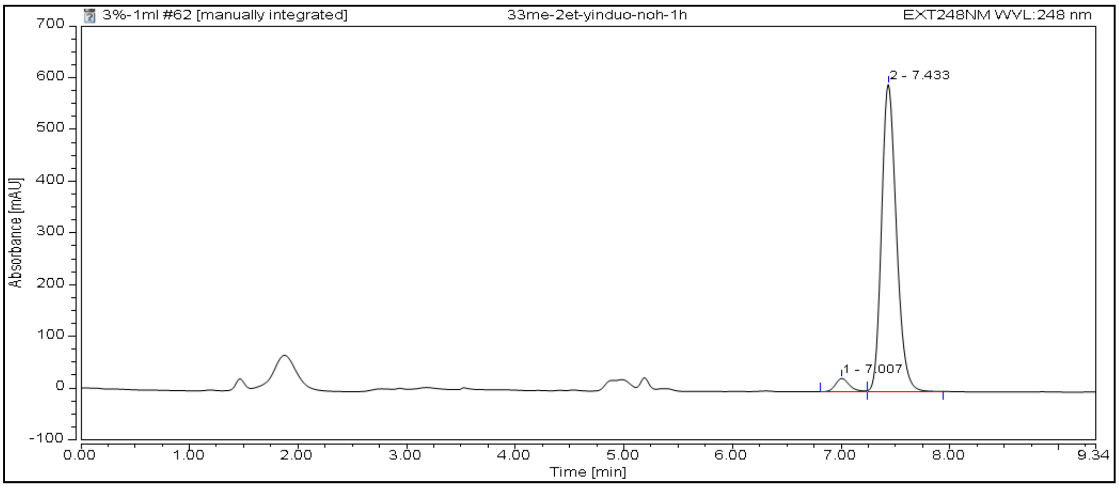

Integration Results					
No.	Peak Name	Retention Time min	Area mAU*min	Relative Area %	Amount n.a.
1		7.007	3.855	3.94	n.a.
2		7.433	94.063	96.06	n.a.
**Total:**			**97.918**	**100.00**	

Supplementary figure 120. HPLC chromatogram for **2l**

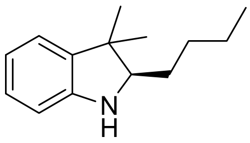

1m

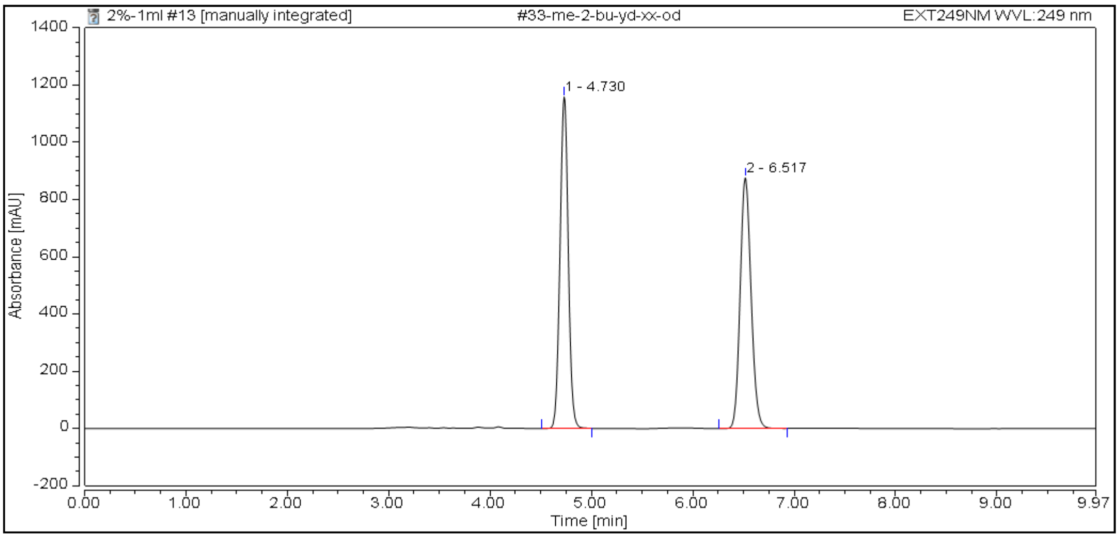

**Integration Results**					
No.	Peak Name	Retention Time min	Area mAU*min	Relative Area %	Amount n.a.
1		4.730	109.374	50.01	n.a.
2		6.517	109.312	49.99	n.a.
**Total:**			**218.686**	**100.00**	

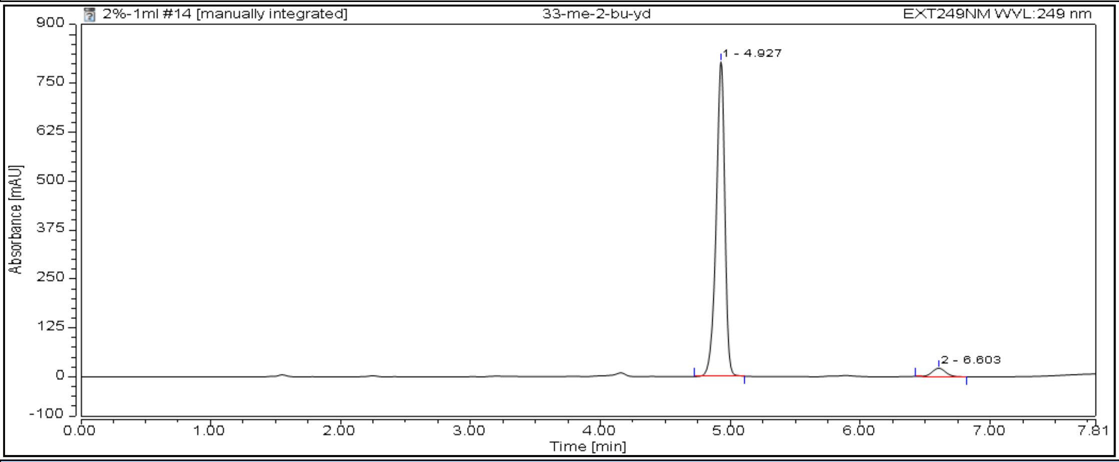

**Integration Results**					
No.	Peak Name	Retention Time min	Area mAU*min	Relative Area %	Amount n.a.
1		4.927	61.998	96.08	n.a.
2		6.603	2.532	3.92	n.a.
**Total:**			**64.529**	**100.00**	

Supplementary figure 121. HPLC chromatogram for **1m**

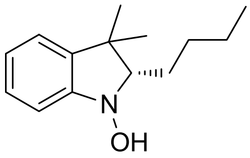

2m

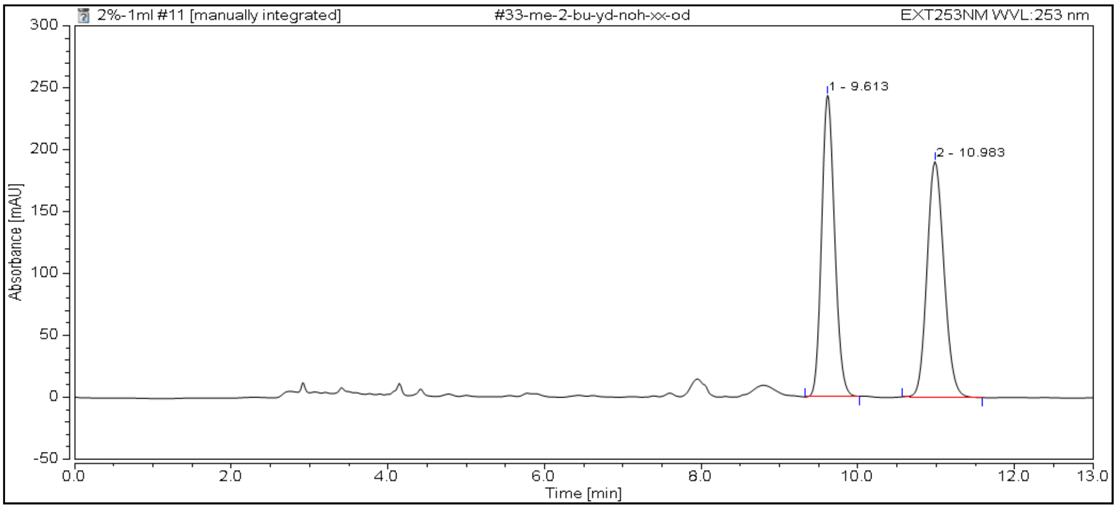

Integration Results					
No.	Peak Name	Retention Time min	Area mAU*min	Relative Area %	Amount n.a.
1		9.613	46.912	49.47	n.a.
2		10.983	47.920	50.53	n.a.
**Total:**			**94.831**	**100.00**	

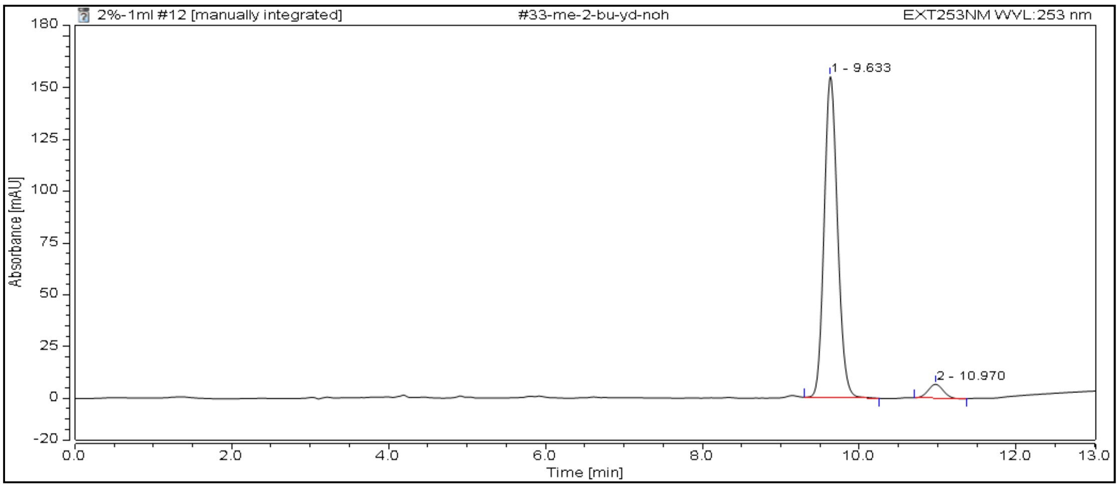

Integration Results					
No.	Peak Name	Retention Time min	Area mAU*min	Relative Area %	Amount n.a.
1		9.633	30.696	95.43	n.a.
2		10.970	1.471	4.57	n.a.
**Total:**			**32.167**	**100.00**	

Supplementary figure 122. HPLC chromatogram for **2m**

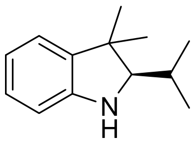

1n

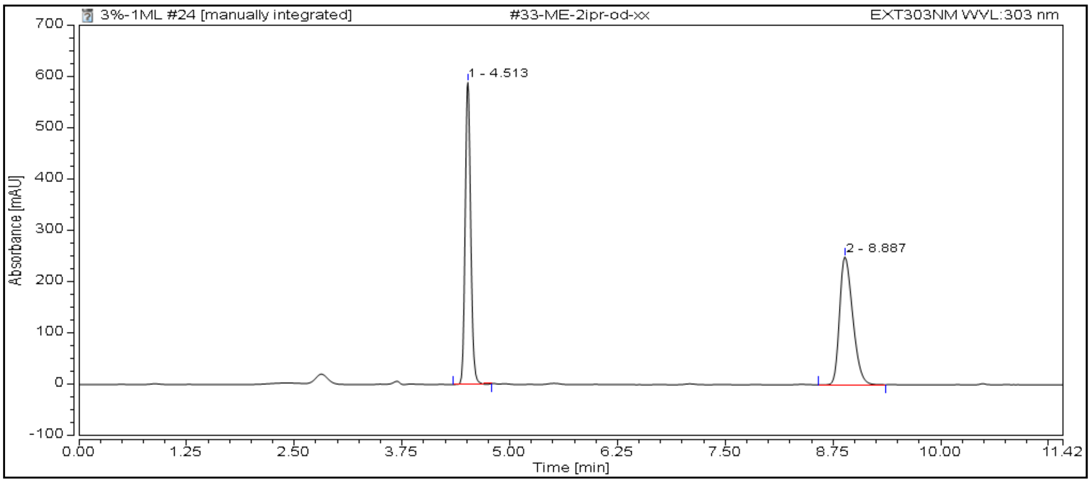

Integration Results					
No.	Peak Name	Retention Time min	Area mAU*min	Relative Area %	Amount n.a.
1		4.513	43.557	49.74	n.a.
2		8.887	44.005	50.26	n.a.
**Total:**			**87.562**	**100.00**	

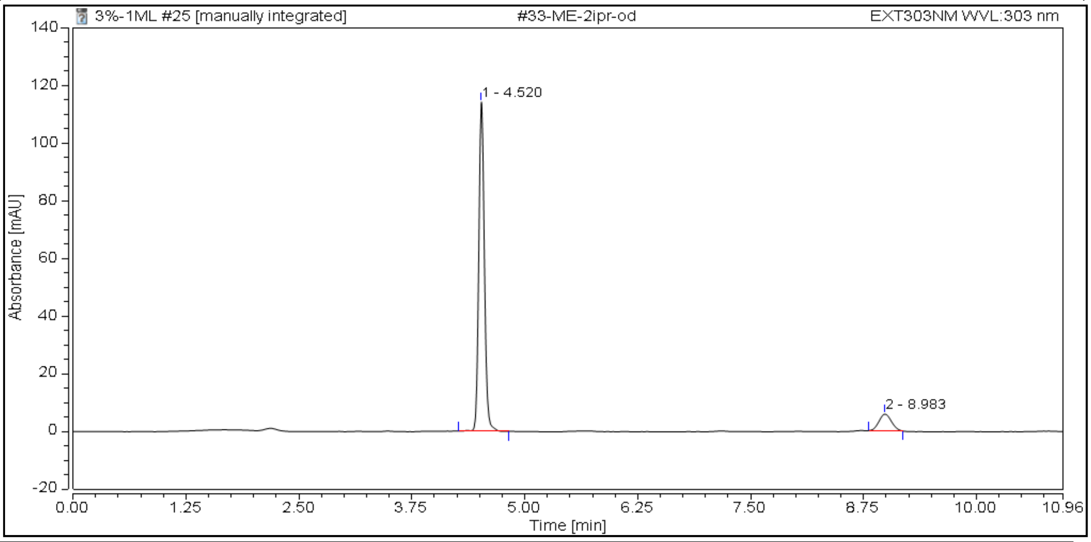

Integration Results					
No.	Peak Name	Retention Time min	Area mAU*min	Relative Area %	Amount n.a.
1		4.520	8.216	89.84	n.a.
2		8.983	0.929	10.16	n.a.
**Total:**			**9.145**	**100.00**	

Supplementary figure 123. HPLC chromatogram for **1n**

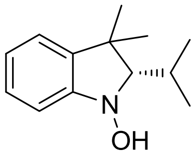

2n

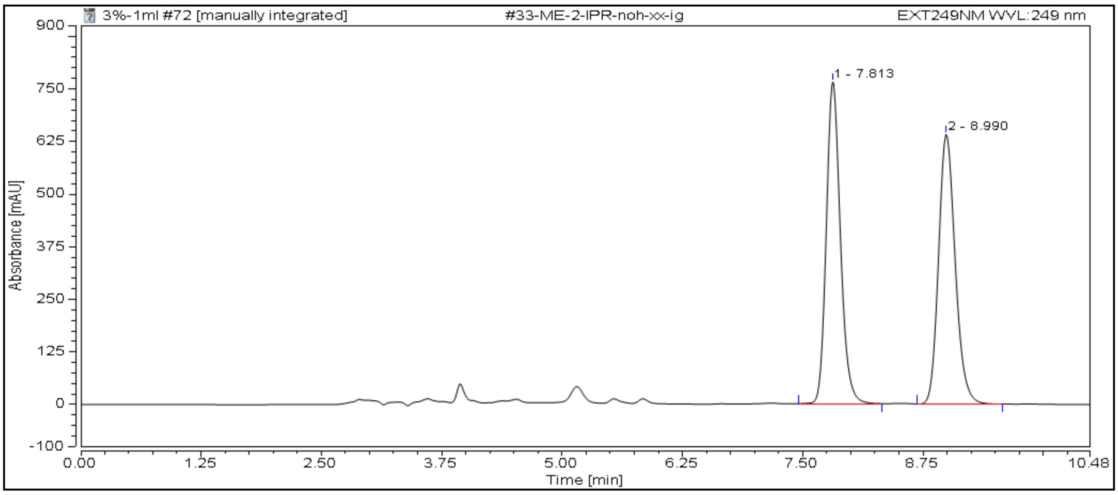

Integration Results					
No.	Peak Name	Retention Time min	Area mAU*min	Relative Area %	Amount n.a.
1		7.813	129.229	50.22	n.a.
2		8.990	128.075	49.78	n.a.
**Total:**			**257.304**	**100.00**	

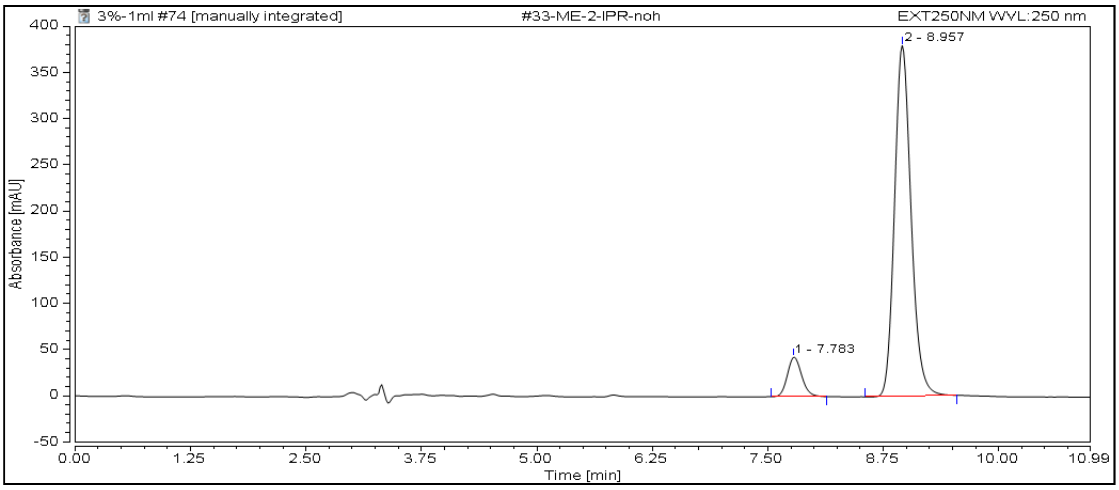

Integration Results					
No.	Peak Name	Retention Time min	Area mAU*min	Relative Area %	Amount n.a.
1		7.783	7.702	8.90	n.a.
2		8.957	78.865	91.10	n.a.
**Total:**			**86.568**	**100.00**	

Supplementary figure 124. HPLC chromatogram for **2n**

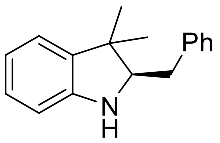

1o

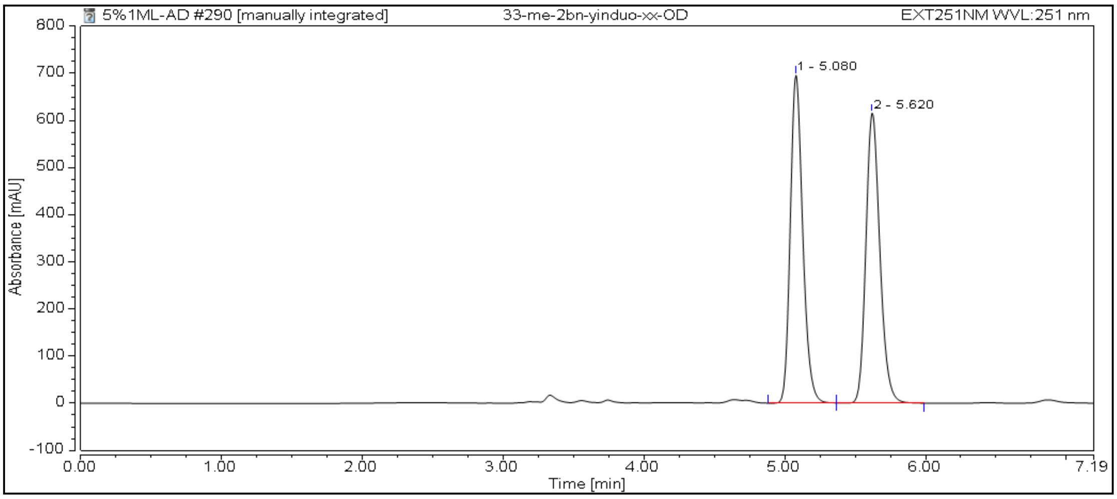

No.	Peak Name	Retention Time min	Area mAU*min	Relative Area %	Amount n.a.
1		5.080	71.055	50.16	n.a.
2		5.620	70.588	49.84	n.a.
**Total:**			**141.644**	**100.00**	

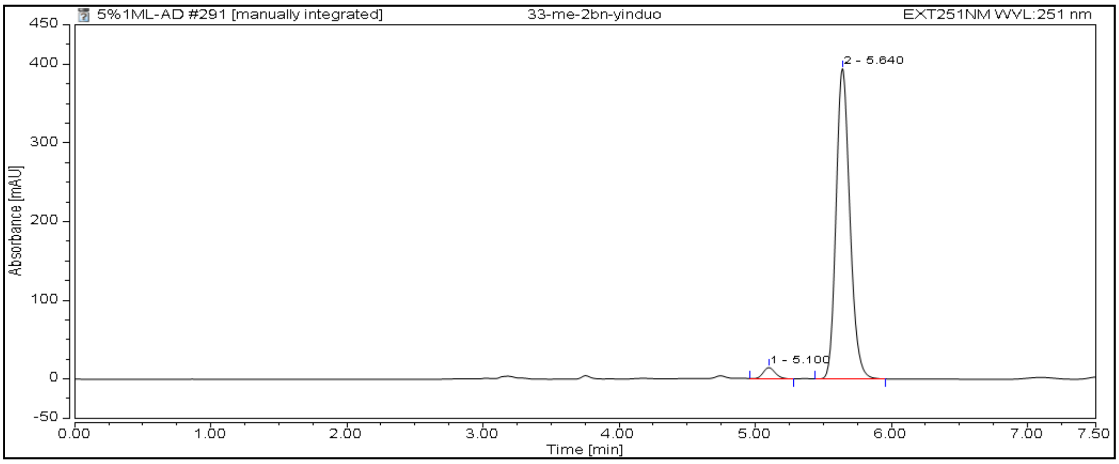

No.	Peak Name	Retention Time min	Area mAU*min	Relative Area %	Amount n.a.
1		5.100	1.418	2.97	n.a.
2		5.640	46.326	97.03	n.a.
**Total:**			**47.744**	**100.00**	

Supplementary figure 125. HPLC chromatogram for **1o**

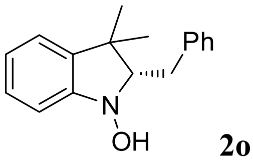

2o

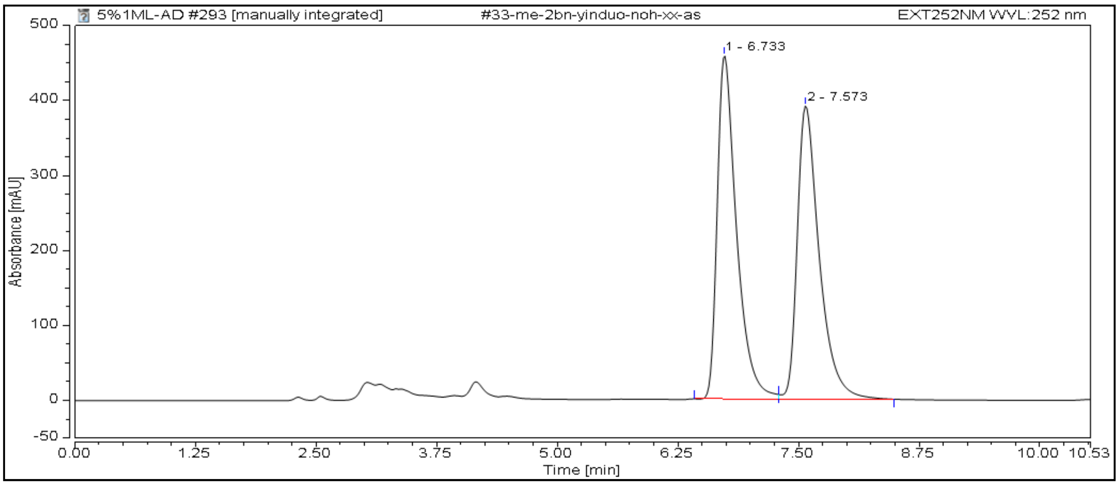

Integration Results					
No.	Peak Name	Retention Time min	Area mAU*min	Relative Area %	Amount n.a.
1		6.733	106.243	50.74	n.a.
2		7.573	103.157	49.26	n.a.
**Total:**			**209.401**	**100.00**	

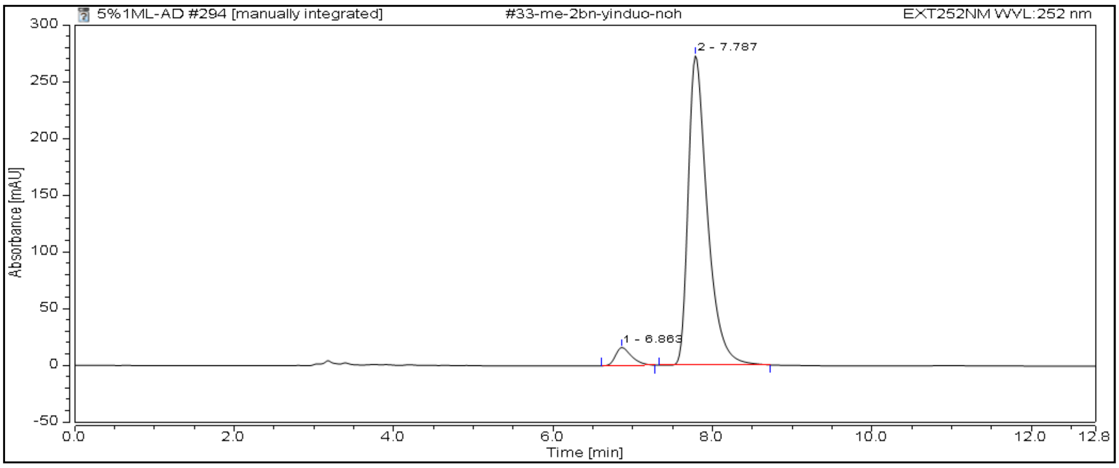

Integration Results					
No.	Peak Name	Retention Time min	Area mAU*min	Relative Area %	Amount n.a.
1		6.863	3.706	4.66	n.a.
2		7.787	75.910	95.34	n.a.
**Total:**			**79.616**	**100.00**	

Supplementary figure 126. HPLC chromatogram for **2o**

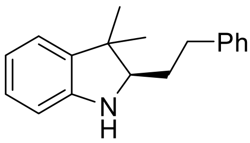

1p

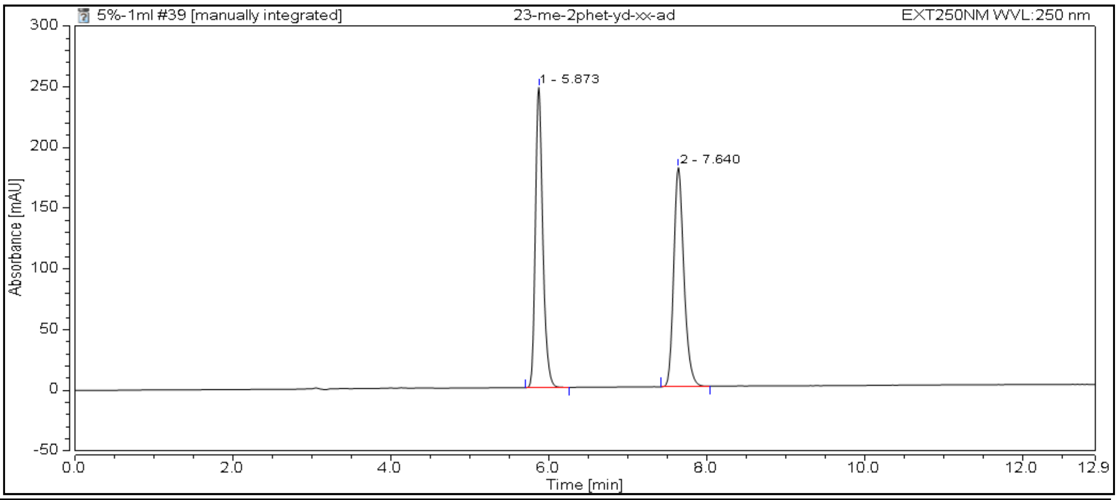

Integration Results

No.	Peak Name	Retention Time min	Area mAU*min	Relative Area %	Amount n.a.
1		5.873	26.838	49.94	n.a.
2		7.640	26.906	50.06	n.a.
**Total:**			**53.744**	**100.00**	

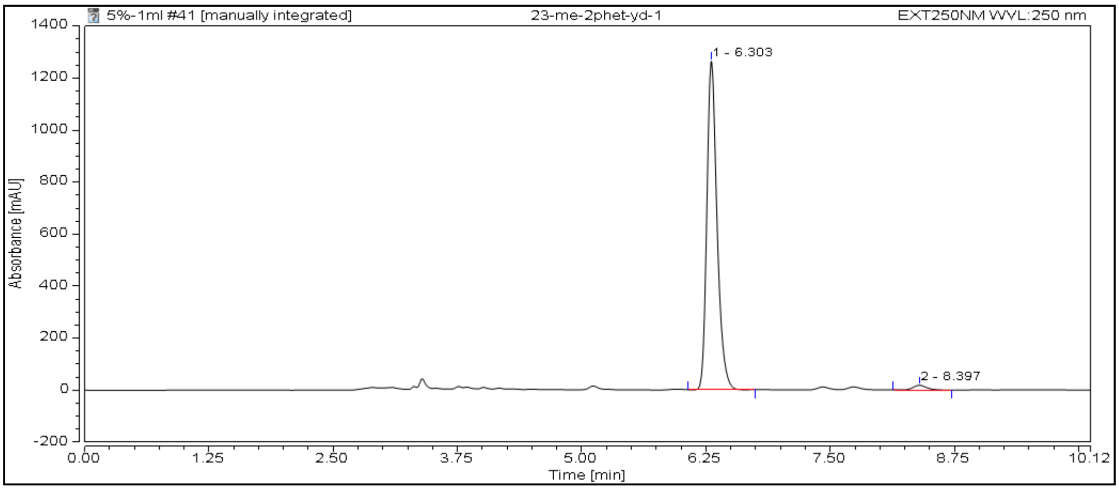

Integration Results

No.	Peak Name	Retention Time min	Area mAU*min	Relative Area %	Amount n.a.
1		6.303	150.159	98.11	n.a.
2		8.397	2.887	1.89	n.a.
**Total:**			**153.046**	**100.00**	

Supplementary figure 127. HPLC chromatogram for **1p**

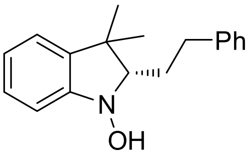

2p

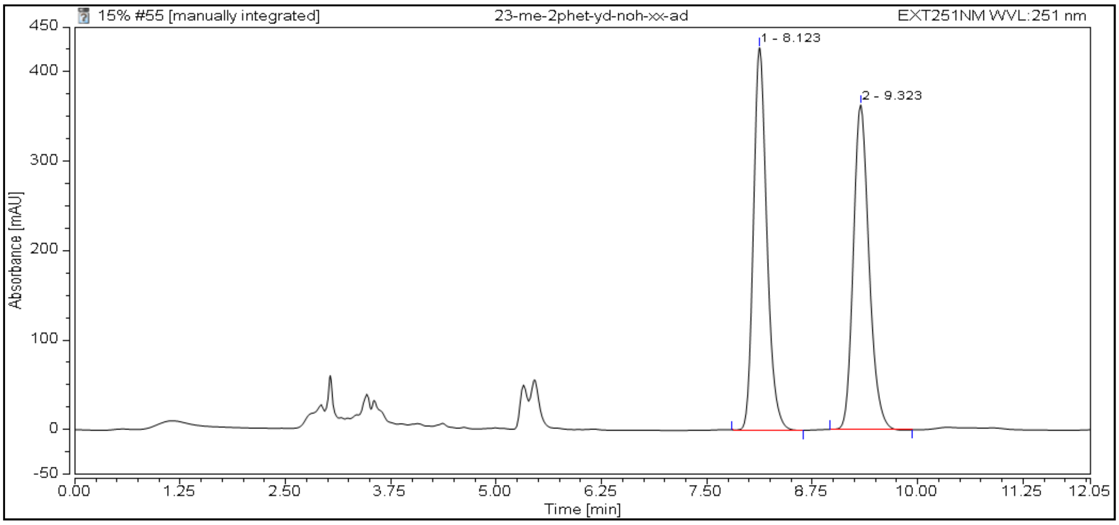

Integration Results					
No.	Peak Name	Retention Time min	Area mAU*min	Relative Area %	Amount n.a.
1		8.123	78.624	50.10	n.a.
2		9.323	78.297	49.90	n.a.
**Total:**			**156.921**	**100.00**	

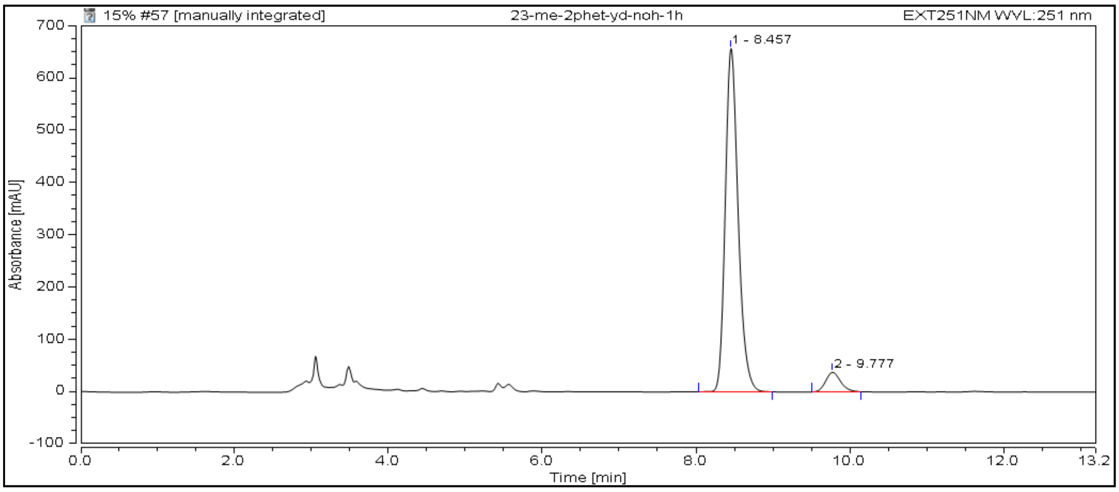

Integration Results					
No.	Peak Name	Retention Time min	Area mAU*min	Relative Area %	Amount n.a.
1		8.457	126.982	93.80	n.a.
2		9.777	8.390	6.20	n.a.
**Total:**			**135.372**	**100.00**	

Supplementary figure 128. HPLC chromatogram for **2p**

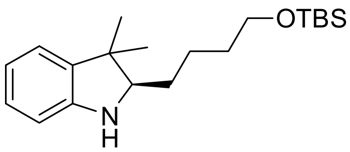

1q

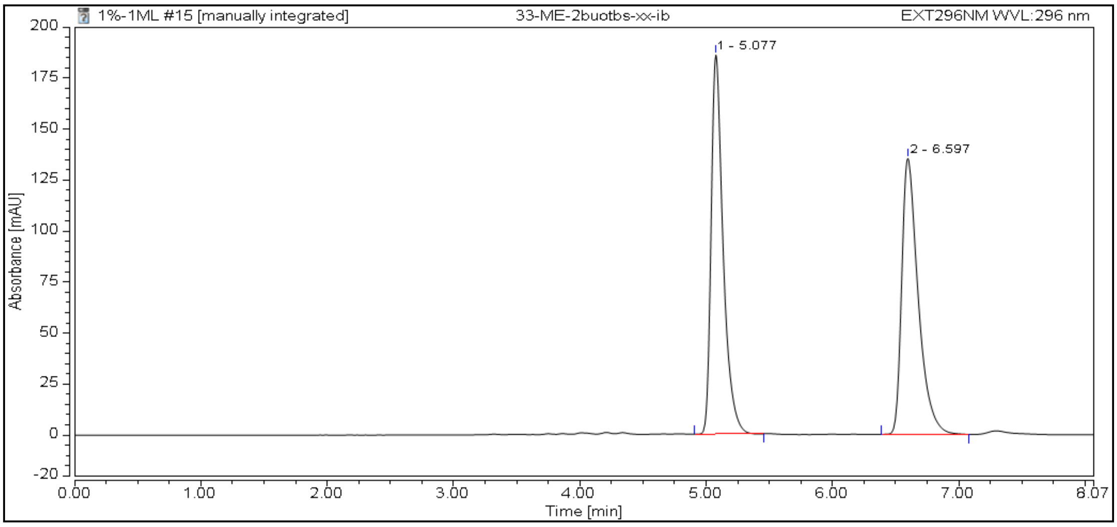

Integration Results

No.	Peak Name	Retention Time min	Area mAU*min	Relative Area %	Amount n.a.
1		5.077	20.400	49.97	n.a.
2		6.597	20.424	50.03	n.a.
**Total:**			**40.824**	**100.00**	

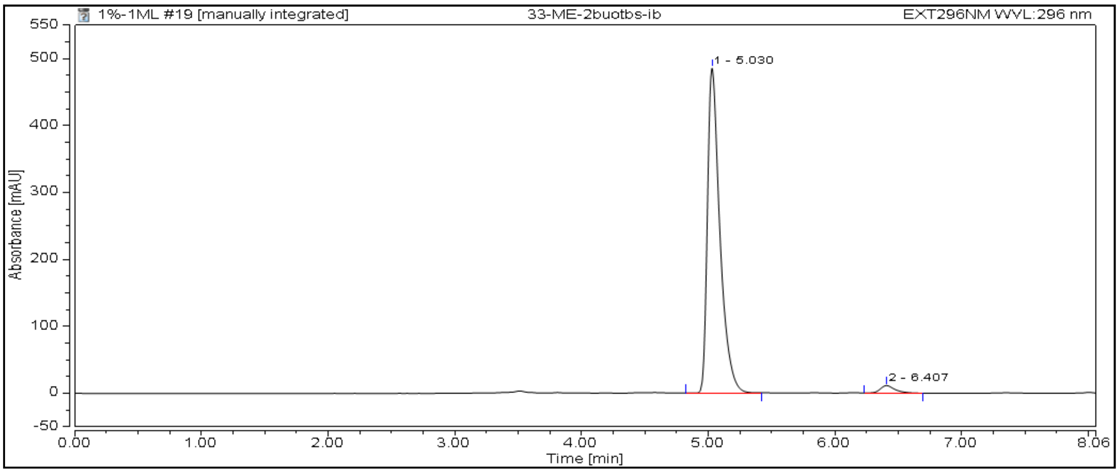

Integration Results

No.	Peak Name	Retention Time min	Area mAU*min	Relative Area %	Amount n.a.
1		5.030	55.236	97.42	n.a.
2		6.407	1.464	2.58	n.a.
**Total:**			**56.700**	**100.00**	

Supplementary figure 129. HPLC chromatogram for **1q**

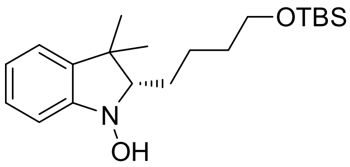

2q

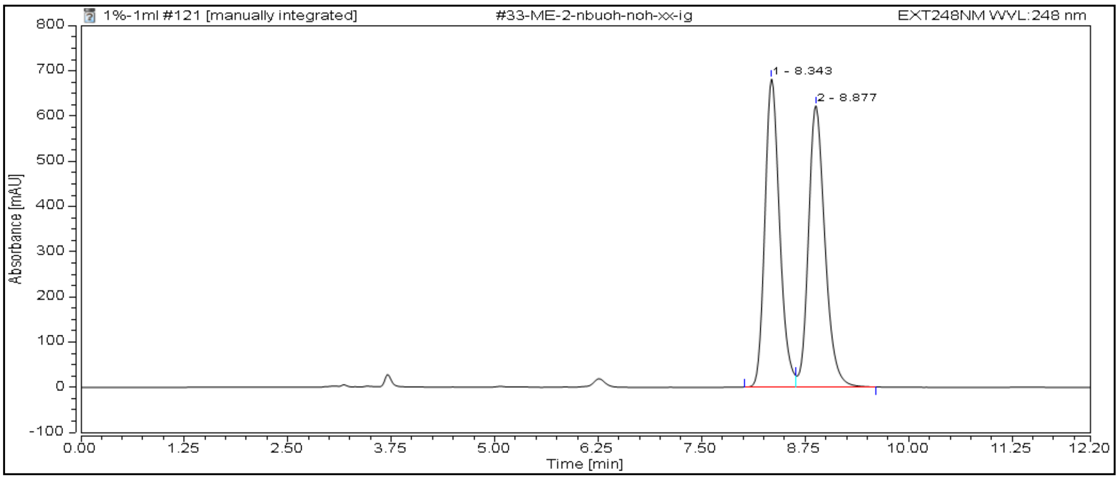

Integration Results					
No.	Peak Name	Retention Time min	Area mAU*min	Relative Area %	Amount n.a.
1		8.343	140.254	49.41	n.a.
2		8.877	143.592	50.59	n.a.
**Total:**			**283.846**	**100.00**	

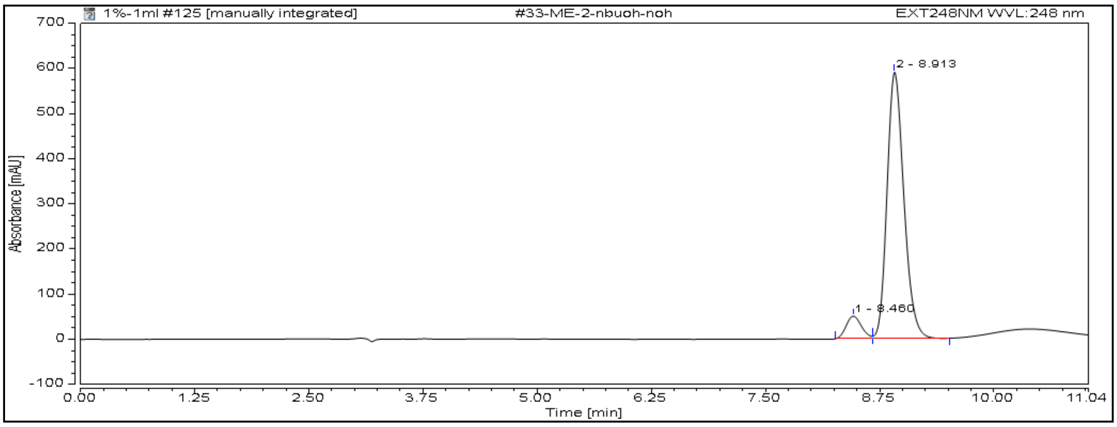

Integration Results					
No.	Peak Name	Retention Time min	Area mAU*min	Relative Area %	Amount n.a.
1		8.460	9.149	6.60	n.a.
2		8.913	129.428	93.40	n.a.
**Total:**			**138.577**	**100.00**	

Supplementary figure 130. HPLC chromatogram for **2q**

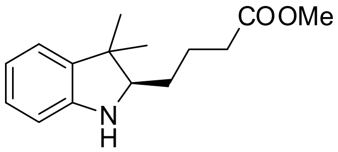

1r

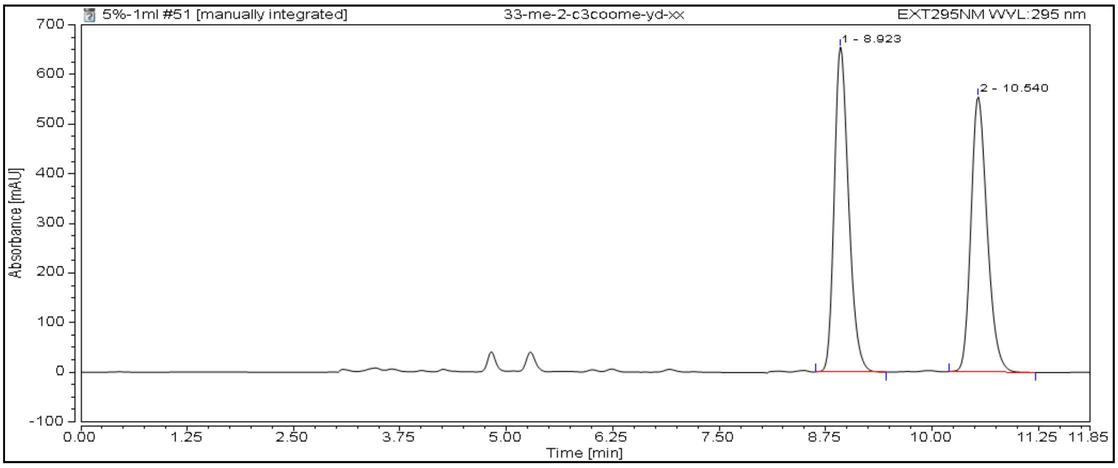

Integration Results					
No.	Peak Name	Retention Time<br>min	Area<br>mAU*min	Relative Area<br>%	Amount<br>n.a.
1		8.923	128.106	51.17	n.a.
2		10.540	122.246	48.83	n.a.
**Total:**			**250.352**	**100.00**	

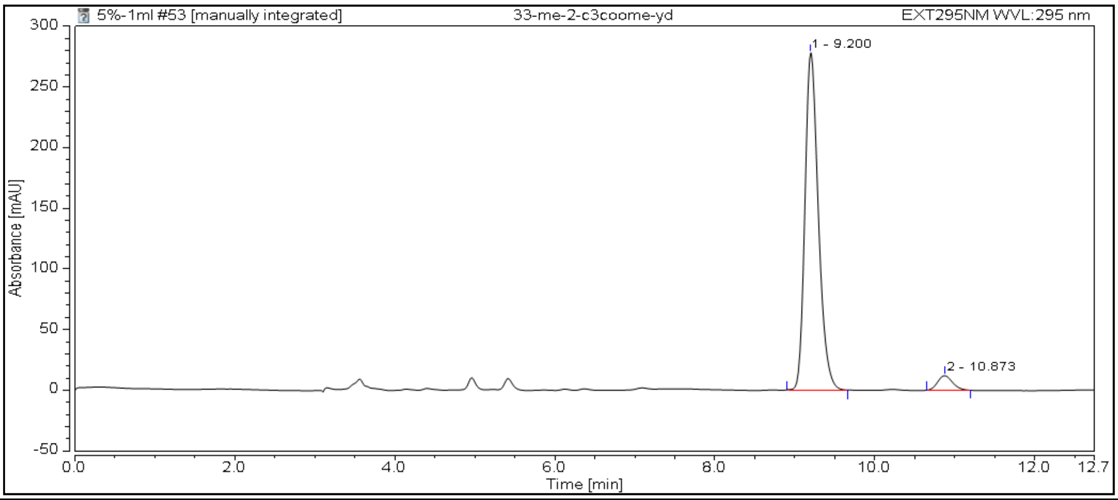

Integration Results					
No.	Peak Name	Retention<br>Time<br>min	Area<br>mAU*min	Relative<br>Area<br>%	Amount<br>n.a.
1		9.200	53.965	95.38	n.a.
2		10.873	2.616	4.62	n.a.
**Total:**			**56.581**	**100.00**	

Supplementary figure 131. HPLC chromatogram for **1r**

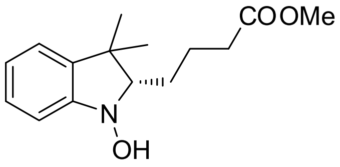

2r

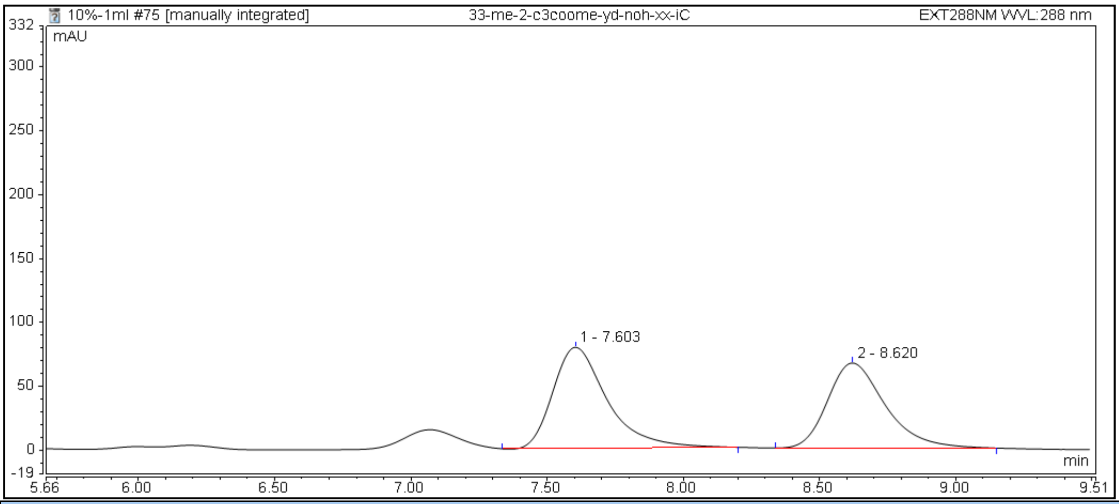

Integration Results

No.	Peak Name	Retention Time<br>min	Area<br>mAU*min	Relative Area<br>%	Amount<br>n.a.
1		7.603	18.248	52.76	n.a.
2		8.620	16.338	47.24	n.a.
**Total:**			**34.586**	**100.00**	

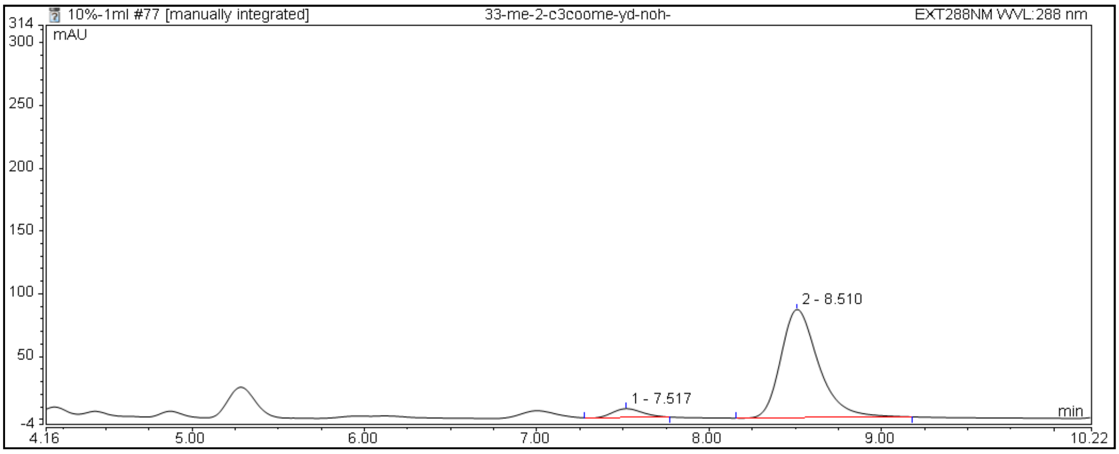

Integration Results

No.	Peak Name	Retention Time<br>min	Area<br>mAU*min	Relative Area<br>%	Amount<br>n.a.
1		7.517	1.400	6.03	n.a.
2		8.510	21.820	93.97	n.a.
**Total:**			**23.220**	**100.00**	

Supplementary figure 132. HPLC chromatogram for **2r**

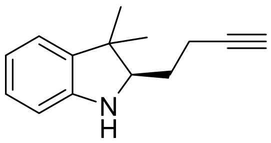

1s

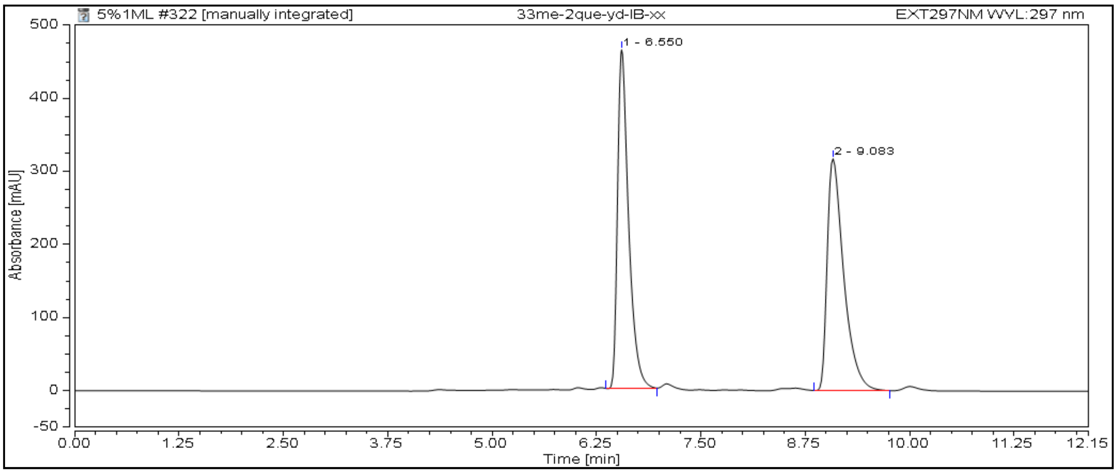

Integration Results					
No.	Peak Name	Retention Time<br>min	Area<br>mAU*min	Relative Area<br>%	Amount<br>n.a.
1		6.550	71.040	49.36	n.a.
2		9.083	72.882	50.64	n.a.
**Total:**			**143.922**	**100.00**	

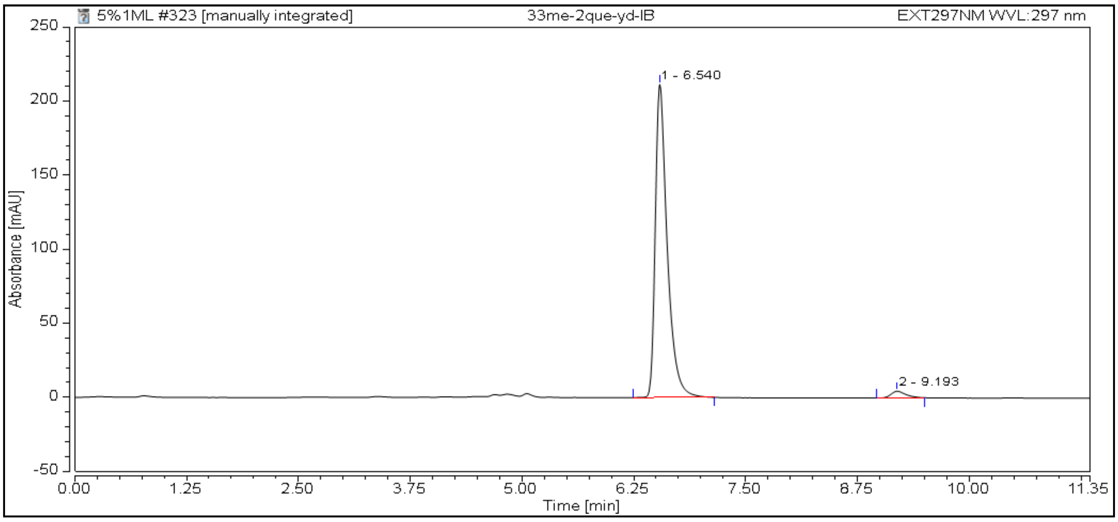

Integration Results					
No.	Peak Name	Retention Time<br>min	Area<br>mAU*min	Relative Area<br>%	Amount<br>n.a.
1		6.540	32.671	97.43	n.a.
2		9.193	0.863	2.57	n.a.
**Total:**			**33.533**	**100.00**	

Supplementary figure 133. HPLC chromatogram for **1s**

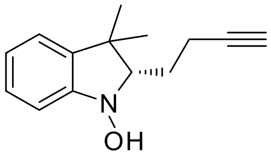

2s

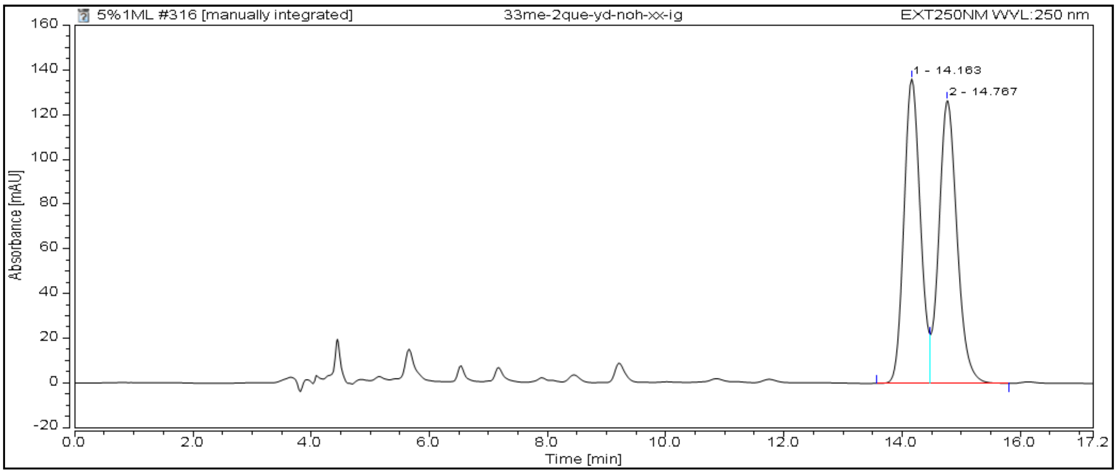

Integration Results					
No.	Peak Name	Retention Time<br>min	Area<br>mAU*min	Relative Area<br>%	Amount<br>n.a.
1		14.163	43.698	49.28	n.a.
2		14.767	44.974	50.72	n.a.
**Total:**			**88.672**	**100.00**	

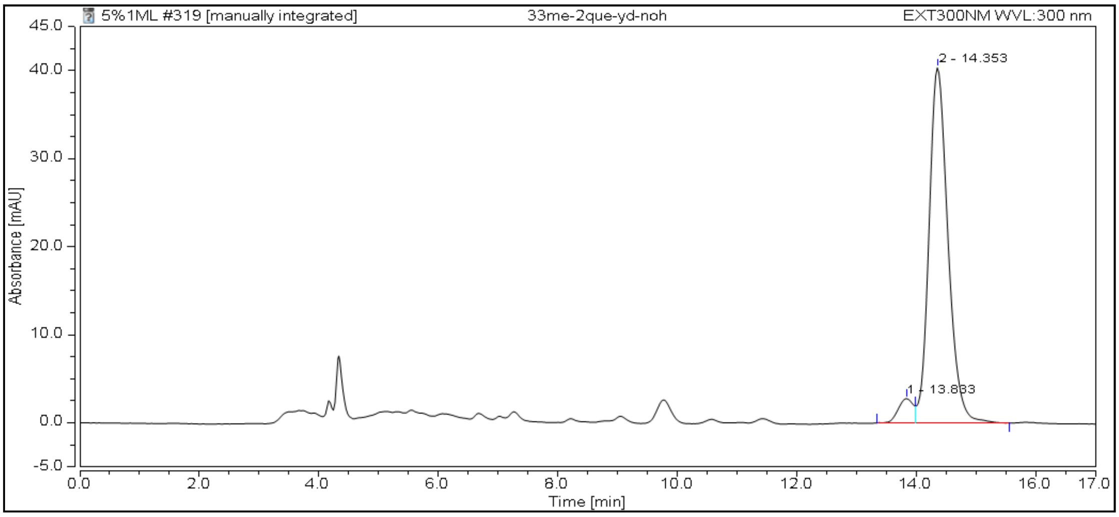

Integration Results					
No.	Peak Name	Retention Time<br>min	Area<br>mAU*min	Relative<br>Area<br>%	Amount<br>n.a.
1		13.833	0.795	5.07	n.a.
2		14.353	14.886	94.93	n.a.
**Total:**			**15.681**	**100.00**	

Supplementary figure 134. HPLC chromatogram for **2s**

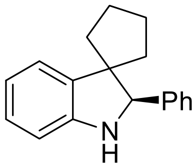

1t

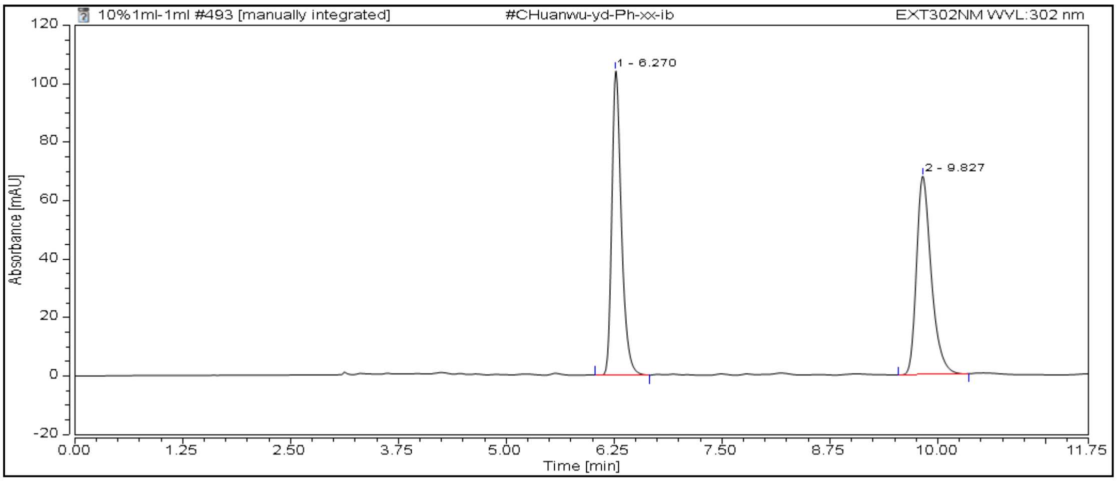

Integration Results					
No.	Peak Name	Retention Time min	Area mAU*min	Relative Area %	Amount n.a.
1		6.270	13.373	49.90	n.a.
2		9.827	13.427	50.10	n.a.
**Total:**			**26.800**	**100.00**	

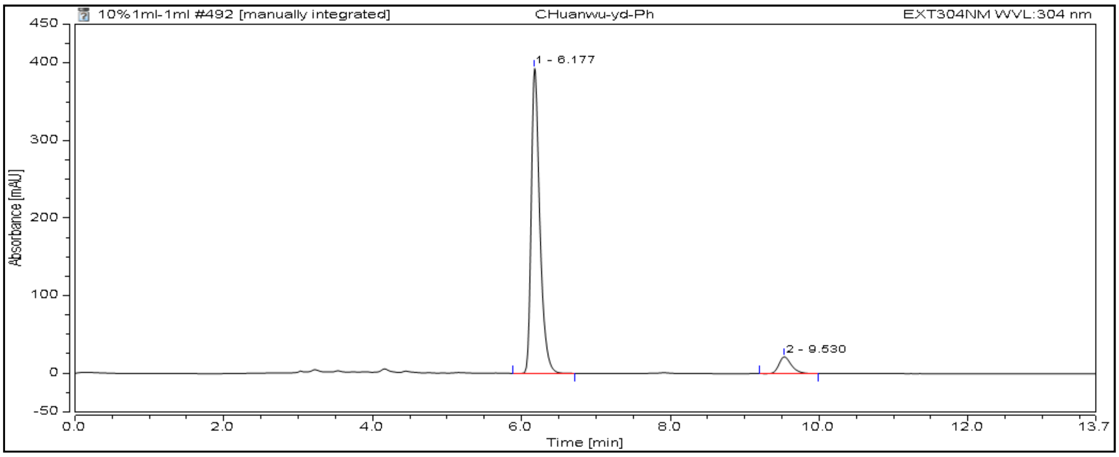

Integration Results					
No.	Peak Name	Retention Time min	Area mAU*min	Relative Area %	Amount n.a.
1		6.177	52.718	92.63	n.a.
2		9.530	4.192	7.37	n.a.
**Total:**			**56.910**	**100.00**	

Supplementary figure 135. HPLC chromatogram for **1r**

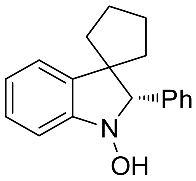

2t

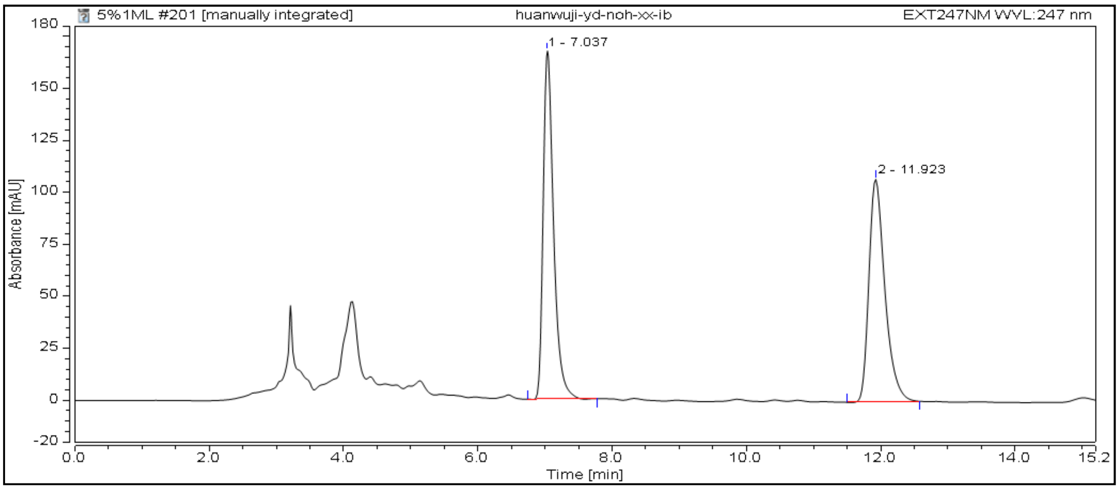

Integration Results					
No.	Peak Name	Retention Time min	Area mAU*min	Relative Area %	Amount n.a.
1		7.037	29.917	50.42	n.a.
2		11.923	29.414	49.58	n.a.
**Total:**			**59.330**	**100.00**	

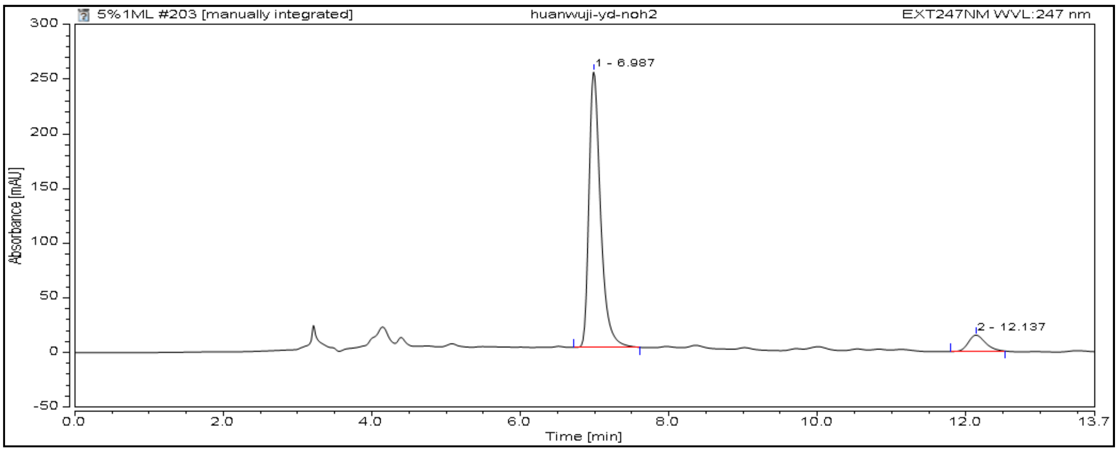

Integration Results					
No.	Peak Name	Retention Time min	Area mAU*min	Relative Area %	Amount n.a.
1		6.987	45.497	92.03	n.a.
2		12.137	3.942	7.97	n.a.
**Total:**			**49.439**	**100.00**	

Supplementary figure 136. HPLC chromatogram for **2r**

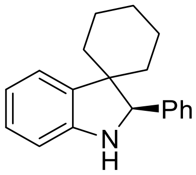

1u

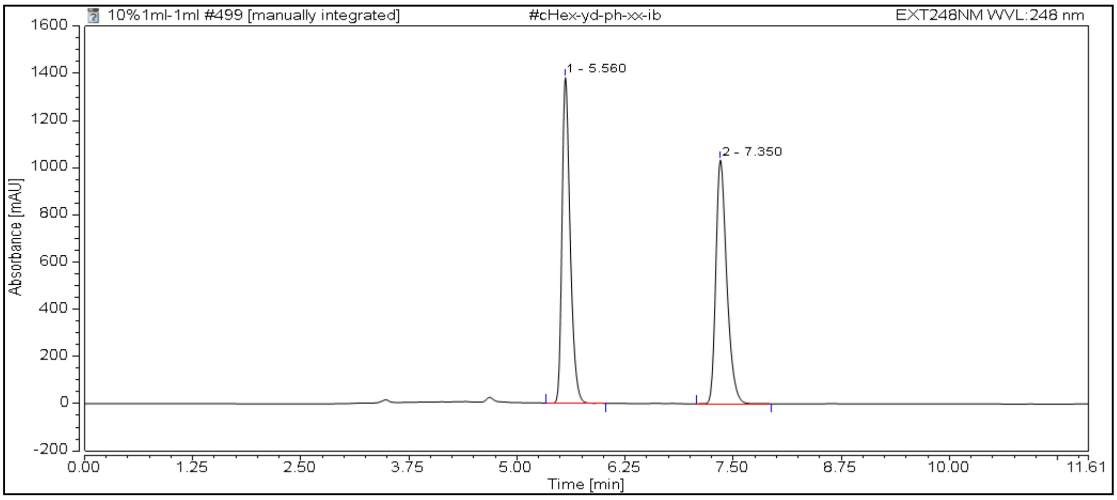

Integration Results					
No.	Peak Name	Retention Time min	Area mAU*min	Relative Area %	Amount n.a.
1		5.560	150.750	49.91	n.a.
2		7.350	151.290	50.09	n.a.
**Total:**			**302.040**	**100.00**	

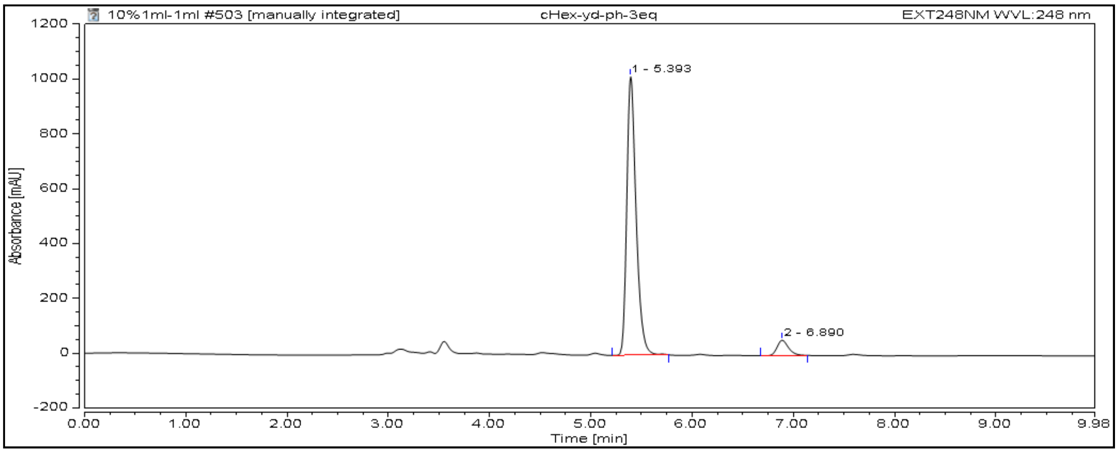

Integration Results					
No.	Peak Name	Retention Time min	Area mAU*min	Relative Area %	Amount n.a.
1		5.393	108.466	93.72	n.a.
2		6.890	7.270	6.28	n.a.
**Total:**			**115.735**	**100.00**	

Supplementary figure 137. HPLC chromatogram for **1s**

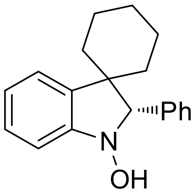

2u

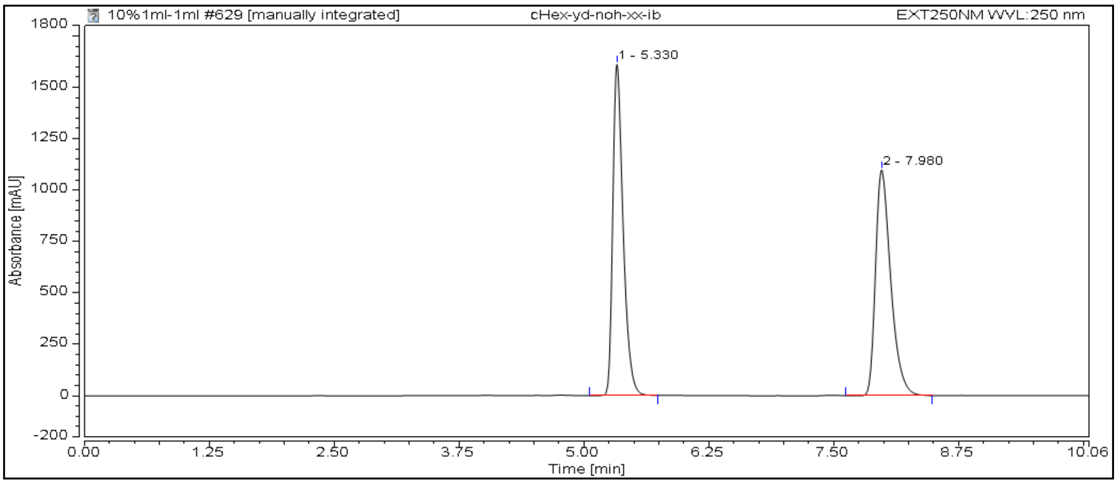

Integration Results					
No.	Peak Name	Retention Time min	Area mAU*min	Relative Area %	Amount n.a.
1		5.330	192.663	49.93	n.a.
2		7.980	193.219	50.07	n.a.
**Total:**			**385.882**	**100.00**	

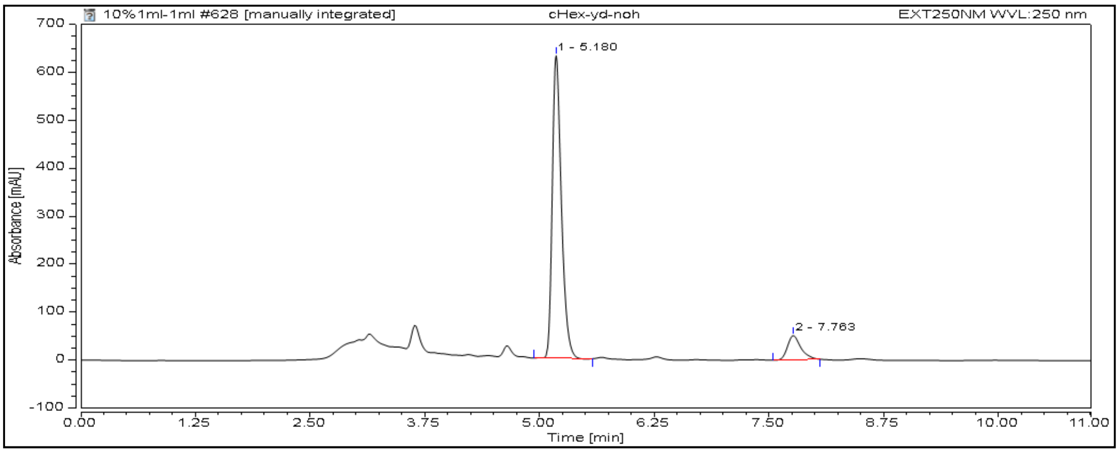

Integration Results					
No.	Peak Name	Retention Time min	Area mAU*min	Relative Area %	Amount n.a.
1		5.180	76.115	89.49	n.a.
2		7.763	8.935	10.51	n.a.
**Total:**			**85.049**	**100.00**	

Supplementary figure 138. HPLC chromatogram for **2s**

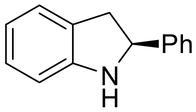

1v

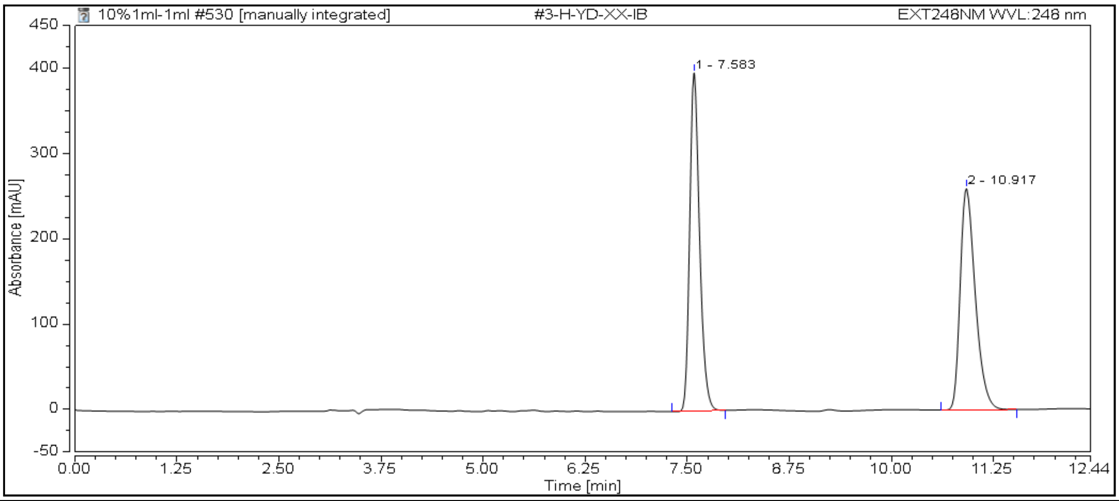

Integration Results

No.	Peak Name	Retention Time min	Area mAU*min	Relative Area %	Amount n.a.
1		7.583	55.475	49.94	n.a.
2		10.917	55.613	50.06	n.a.
**Total:**			**111.087**	**100.00**	

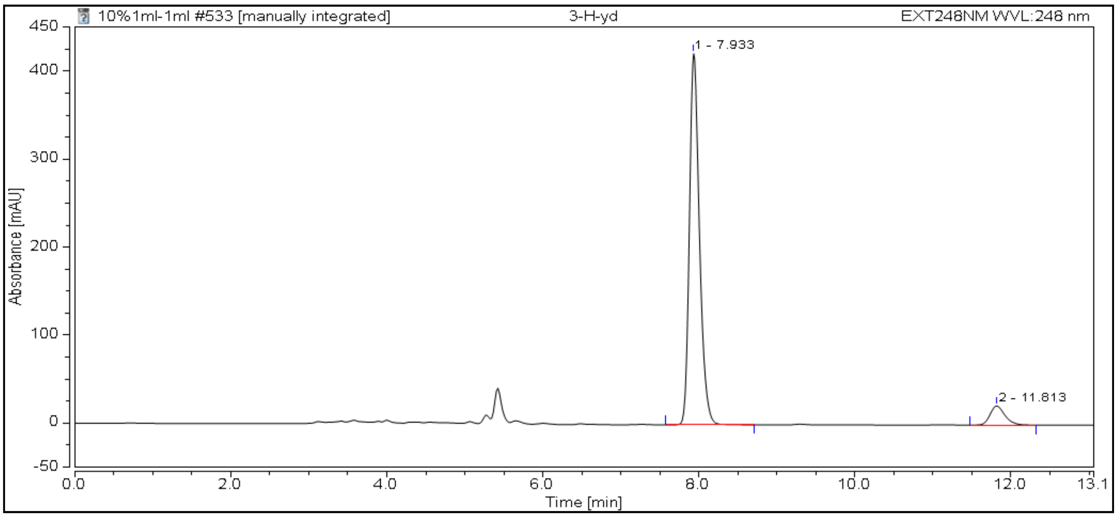

Integration Results

No.	Peak Name	Retention Time min	Area mAU*min	Relative Area %	Amount n.a.
1		7.933	62.778	92.94	n.a.
2		11.813	4.772	7.06	n.a.
**Total:**			**67.550**	**100.00**	

Supplementary figure 139. HPLC chromatogram for **1t**

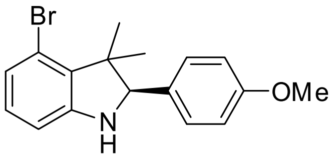

4a

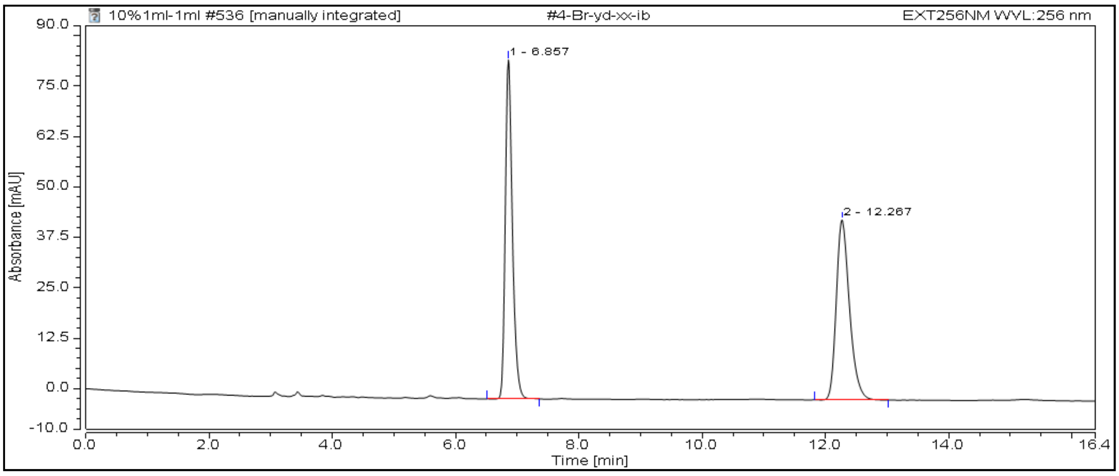

Integration Results

No.	Peak Name	Retention Time min	Area mAU*min	Relative Area %	Amount n.a.
1		6.857	11.219	50.28	n.a.
2		12.267	11.095	49.72	n.a.
**Total:**			**22.314**	**100.00**	

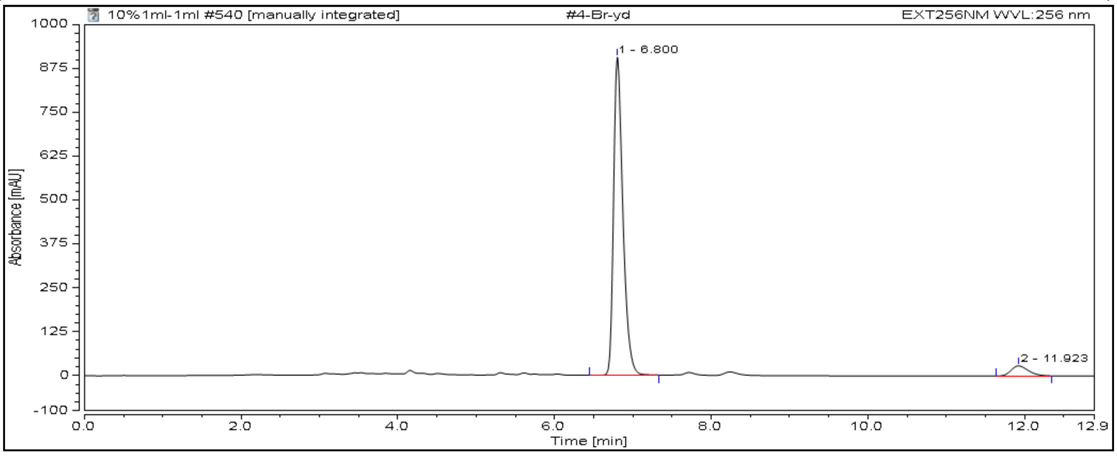

Integration Results

No.	Peak Name	Retention Time min	Area mAU*min	Relative Area %	Amount n.a.
1		6.800	124.746	94.43	n.a.
2		11.923	7.357	5.57	n.a.
**Total:**			**132.104**	**100.00**	

Supplementary figure 140. HPLC chromatogram for **4a**

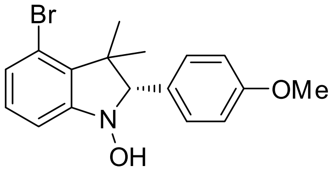

5a

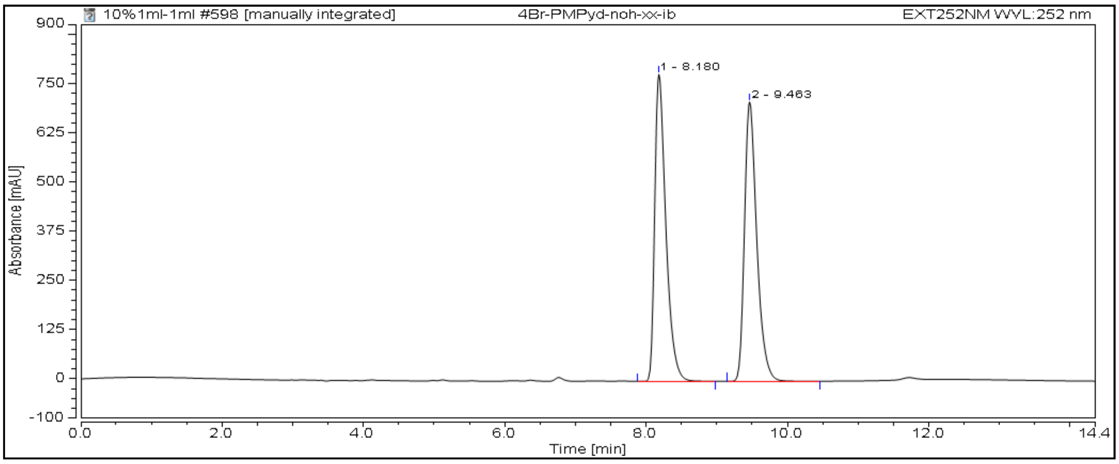

Integration Results					
No.	Peak Name	Retention Time min	Area mAU*min	Relative Area %	Amount n.a.
1		8.180	142.428	49.98	n.a.
2		9.463	142.549	50.02	n.a.
**Total:**			**284.977**	**100.00**	

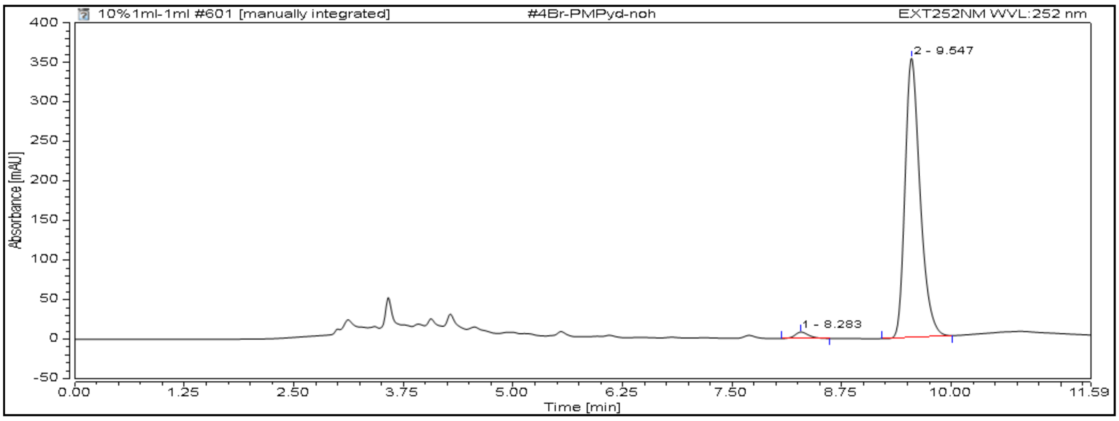

Integration Results					
No.	Peak Name	Retention Time min	Area mAU*min	Relative Area %	Amount n.a.
1		8.283	1.474	2.05	n.a.
2		9.547	70.345	97.95	n.a.
**Total:**			**71.819**	**100.00**	

Supplementary figure 141. HPLC chromatogram for **5a**

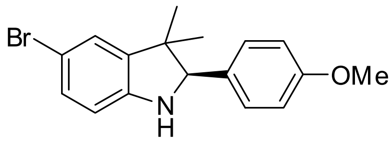

4b

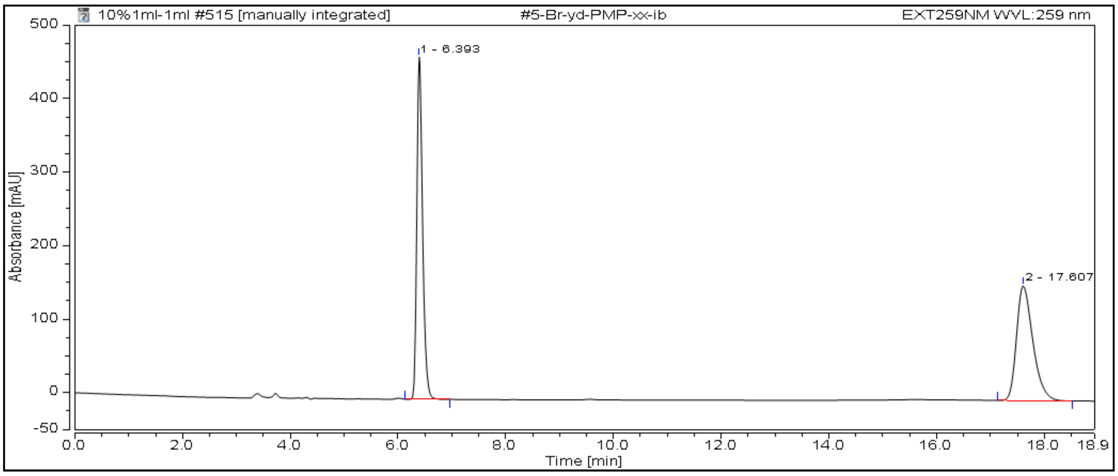

Integration Results					
No.	Peak Name	Retention Time min	Area mAU*min	Relative Area %	Amount n.a.
1		6.393	57.630	51.25	n.a.
2		17.607	54.816	48.75	n.a.
**Total:**			**112.446**	**100.00**	

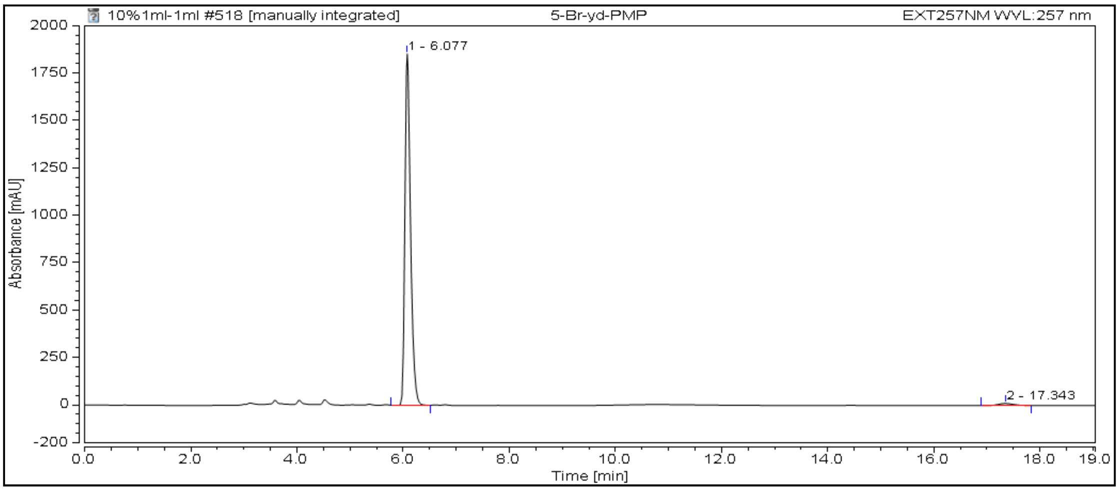

Integration Results					
No.	Peak Name	Retention Time min	Area mAU*min	Relative Area %	Amount n.a.
1		6.077	227.010	98.35	n.a.
2		17.343	3.799	1.65	n.a.
**Total:**			**230.809**	**100.00**	

Supplementary figure 142. HPLC chromatogram for **4b**

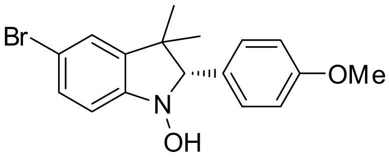

5b

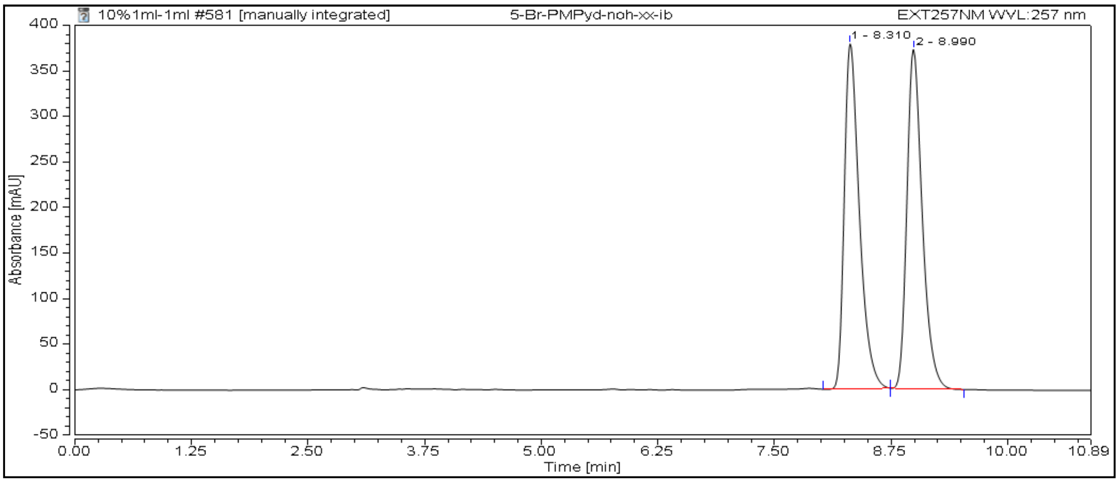

Integration Results					
No.	Peak Name	Retention Time min	Area mAU*min	Relative Area %	Amount n.a.
1		8.310	71.036	50.00	n.a.
2		8.990	71.041	50.00	n.a.
**Total:**			**142.077**	**100.00**	

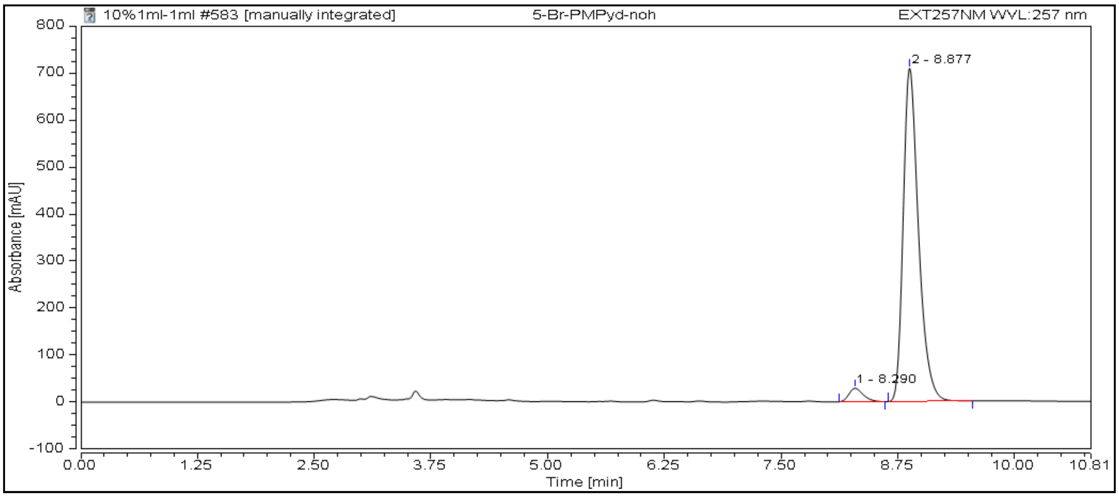

Integration Results					
No.	Peak Name	Retention Time min	Area mAU*min	Relative Area %	Amount n.a.
1		8.290	5.175	3.70	n.a.
2		8.877	134.544	96.30	n.a.
**Total:**			**139.719**	**100.00**	

Supplementary figure 143. HPLC chromatogram for **5b**

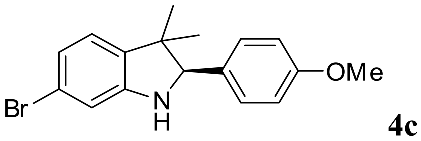

4c

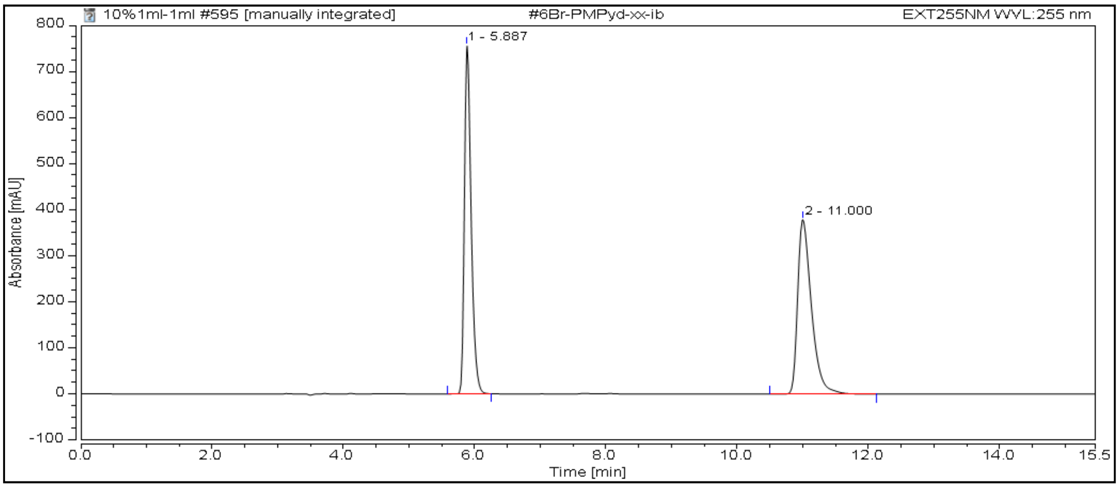

Integration Results					
No.	Peak Name	Retention Time min	Area mAU*min	Relative Area %	Amount n.a.
1		5.887	89.748	49.50	n.a.
2		11.000	91.572	50.50	n.a.
**Total:**			**181.320**	**100.00**	

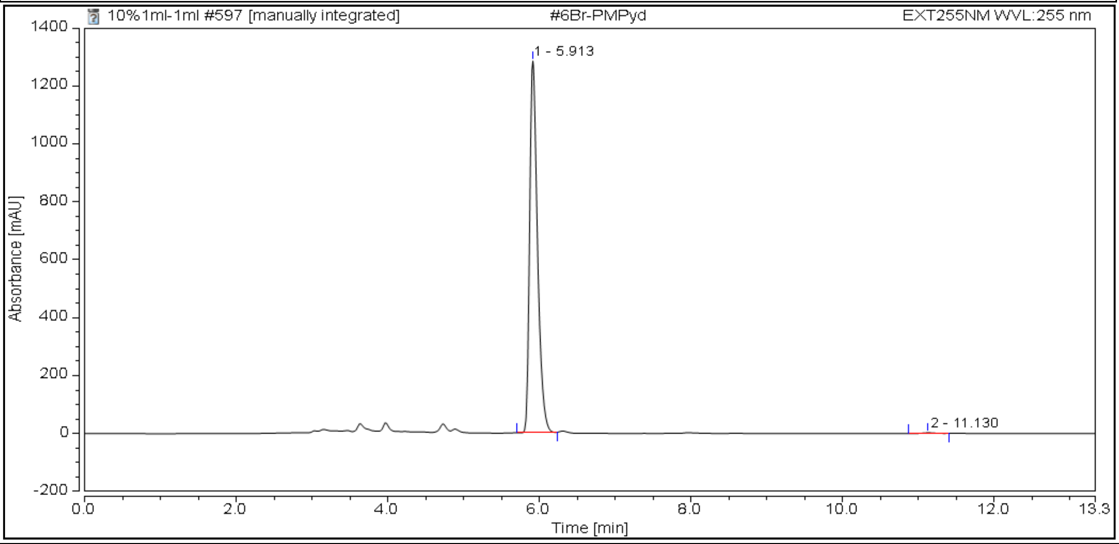

Integration Results					
No.	Peak Name	Retention Time min	Area mAU*min	Relative Area %	Amount n.a.
1		5.913	153.192	99.74	n.a.
2		11.130	0.401	0.26	n.a.
**Total:**			**153.593**	**100.00**	

Supplementary figure 144. HPLC chromatogram for **4c**

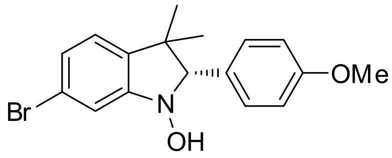

5c

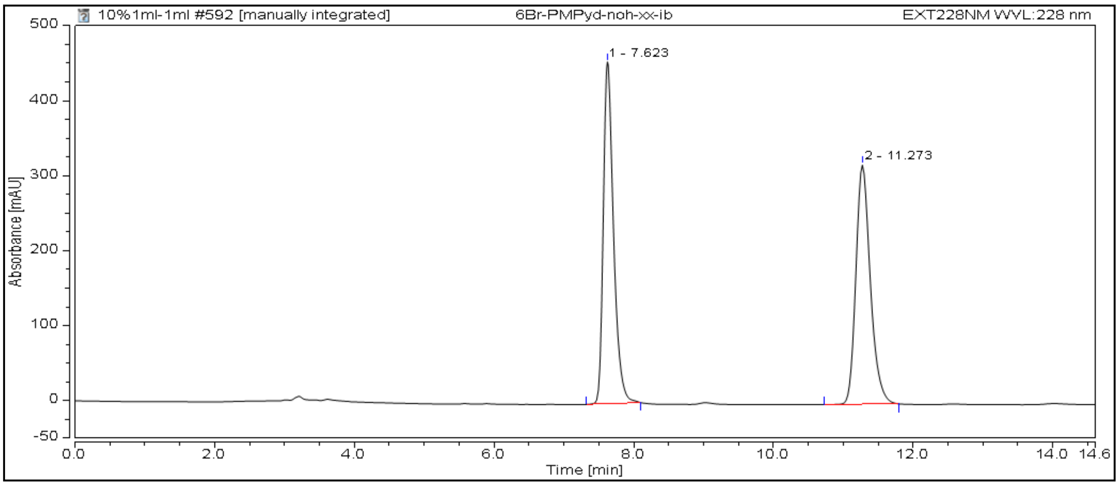

Integration Results					
No.	Peak Name	Retention Time min	Area mAU*min	Relative Area %	Amount n.a.
1		7.623	77.013	50.21	n.a.
2		11.273	76.368	49.79	n.a.
**Total:**			**153.381**	**100.00**	

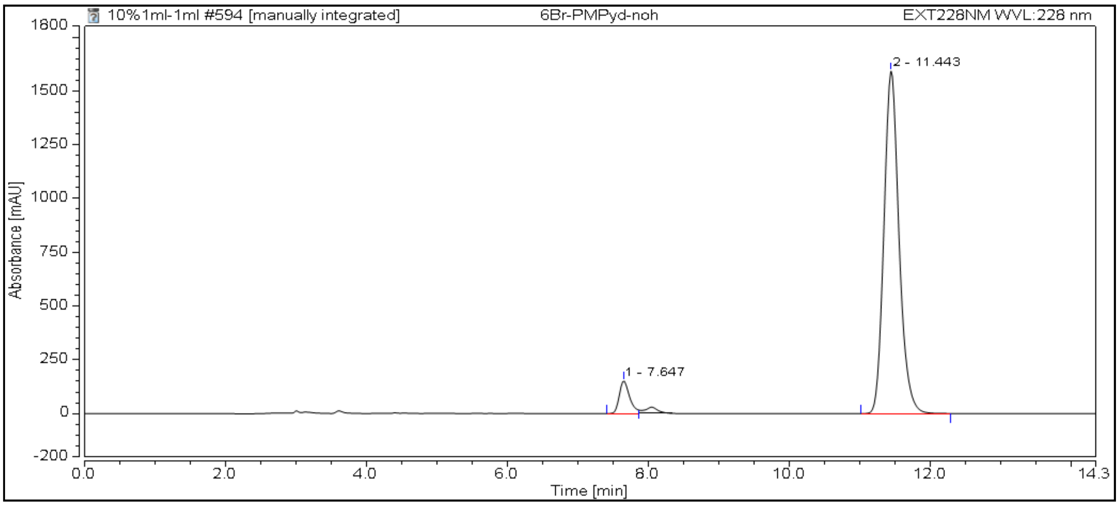

Integration Results					
No.	Peak Name	Retention Time min	Area mAU*min	Relative Area %	Amount n.a.
1		7.647	25.747	6.11	n.a.
2		11.443	395.579	93.89	n.a.
**Total:**			**421.326**	**100.00**	

Supplementary figure 145. HPLC chromatogram for **5c**

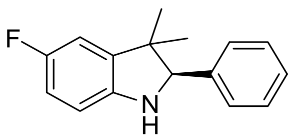

4d

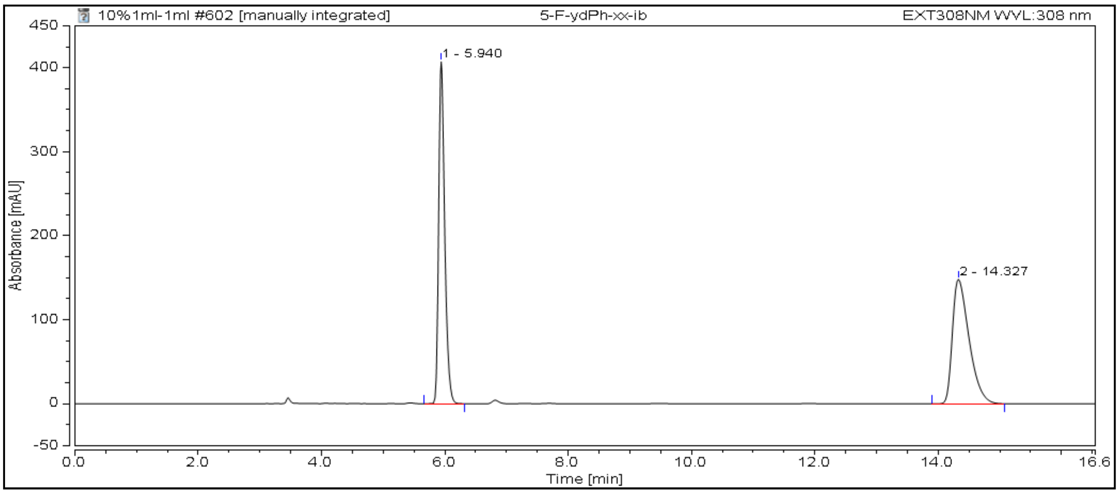

Integration Results					
No.	Peak Name	Retention Time min	Area mAU*min	Relative Area %	Amount n.a.
1		5.940	46.316	50.00	n.a.
2		14.327	46.317	50.00	n.a.
**Total:**			**92.634**	**100.00**	

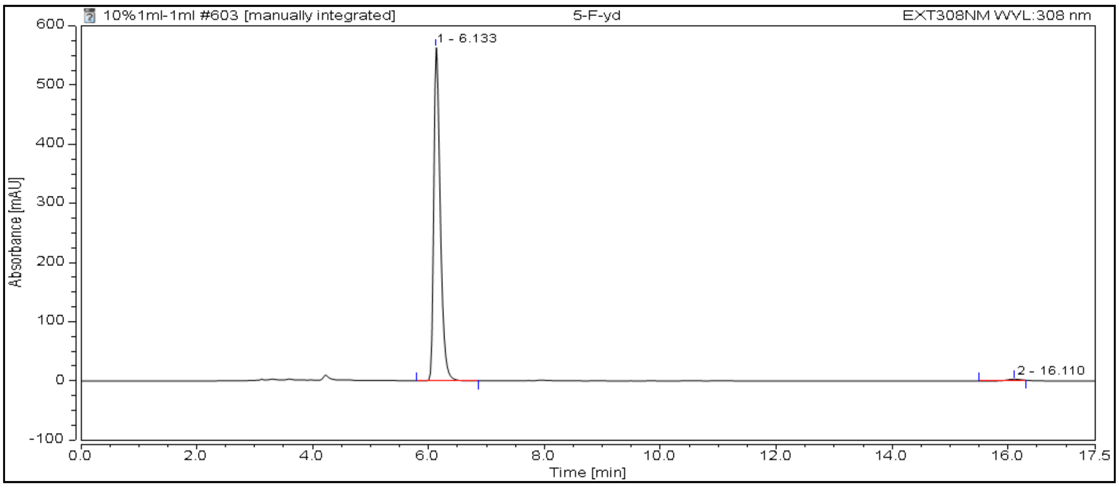

Integration Results					
No.	Peak Name	Retention Time min	Area mAU*min	Relative Area %	Amount n.a.
1		6.133	73.045	99.44	n.a.
2		16.110	0.410	0.56	n.a.
**Total:**			**73.454**	**100.00**	

Supplementary figure 146. HPLC chromatogram for **4d**

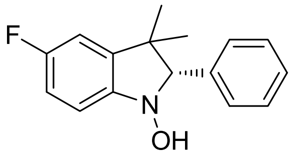

5d

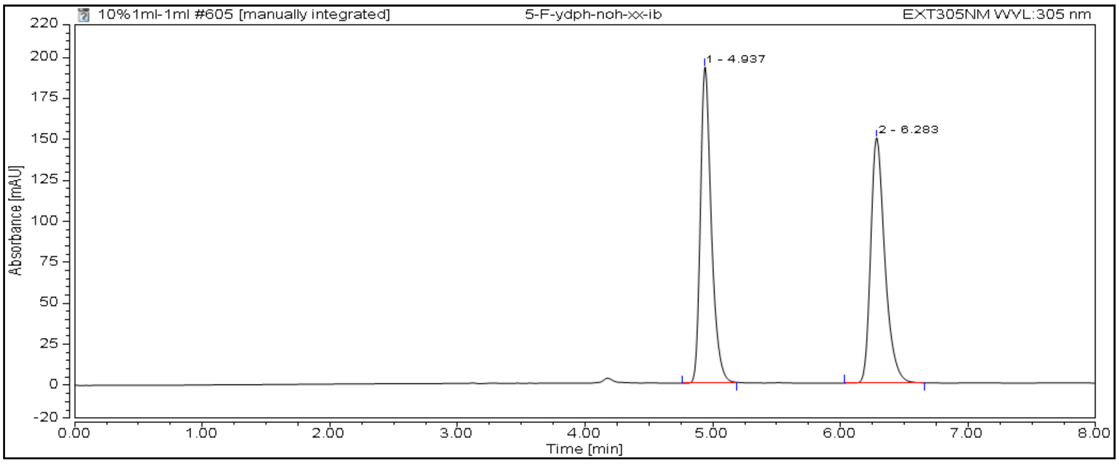

Integration Results					
No.	Peak Name	Retention Time min	Area mAU*min	Relative Area %	Amount n.a.
1		4.937	19.051	49.78	n.a.
2		6.283	19.217	50.22	n.a.
**Total:**			**38.268**	**100.00**	

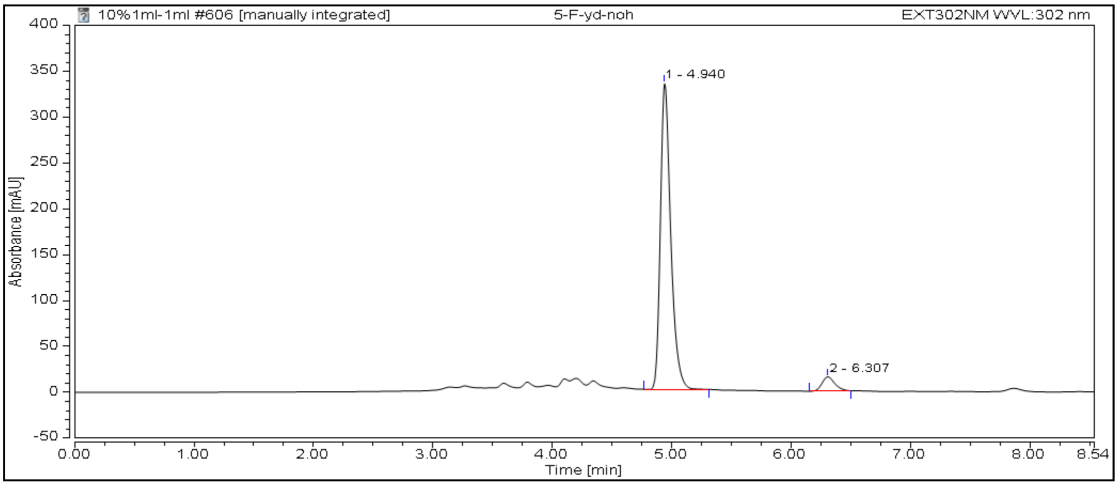

Integration Results					
No.	Peak Name	Retention Time min	Area mAU*min	Relative Area %	Amount n.a.
1		4.940	33.571	94.85	n.a.
2		6.307	1.821	5.15	n.a.
**Total:**			**35.393**	**100.00**	

Supplementary figure 147. HPLC chromatogram for **5d**

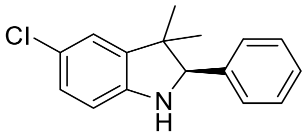

4e

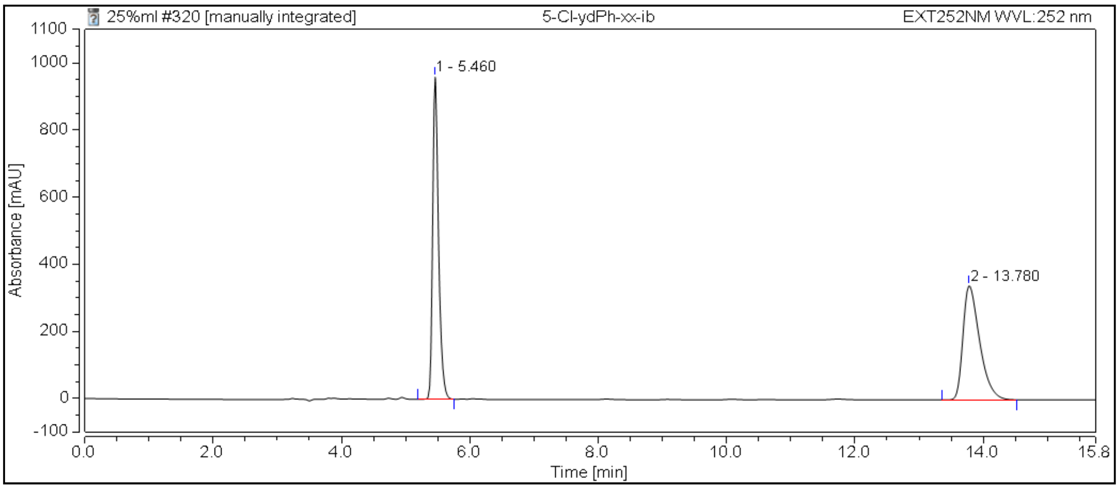

Integration Results					
No.	Peak Name	Retention Time min	Area mAU*min	Relative Area %	Amount n.a.
1		5.460	101.810	49.94	n.a.
2		13.780	102.041	50.06	n.a.
**Total:**			**203.851**	**100.00**	

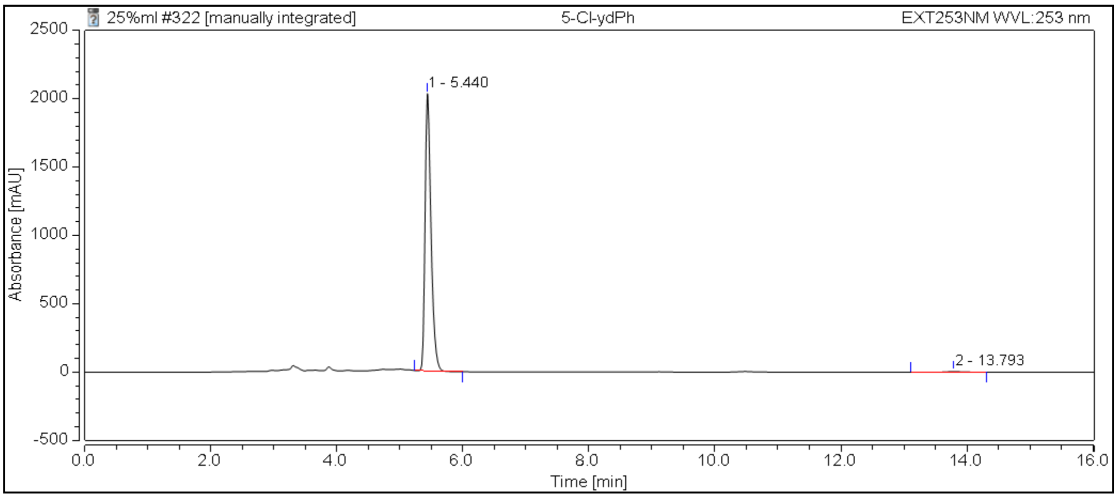

Integration Results					
No.	Peak Name	Retention Time min	Area mAU*min	Relative Area %	Amount n.a.
1		5.440	220.105	99.46	n.a.
2		13.793	1.189	0.54	n.a.
**Total:**			**221.295**	**100.00**	

Supplementary figure 148. HPLC chromatogram for **4e**

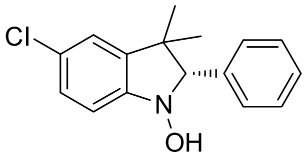

5e

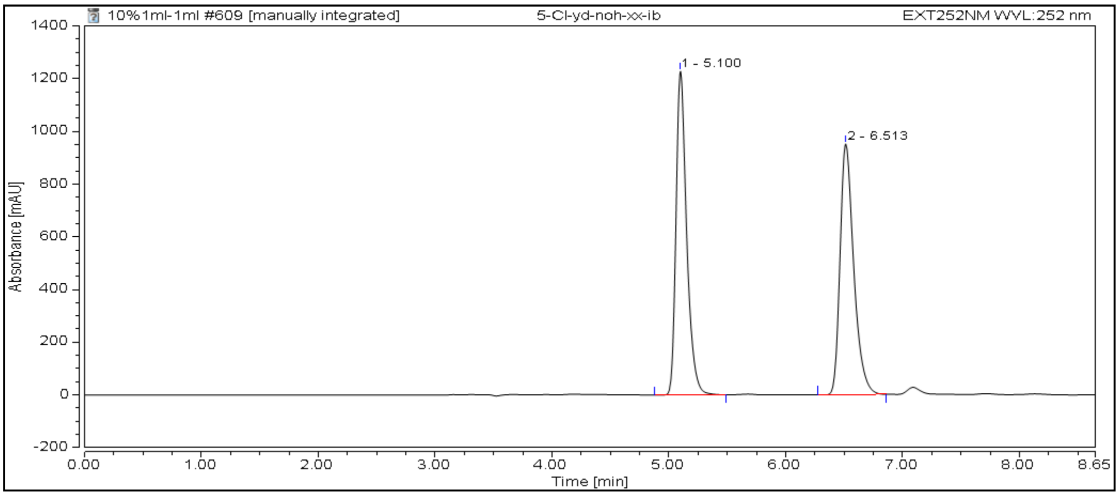

Integration Results					
No.	Peak Name	Retention Time min	Area mAU*min	Relative Area %	Amount n.a.
1		5.100	129.318	50.09	n.a.
2		6.513	128.842	49.91	n.a.
**Total:**			**258.159**	**100.00**	

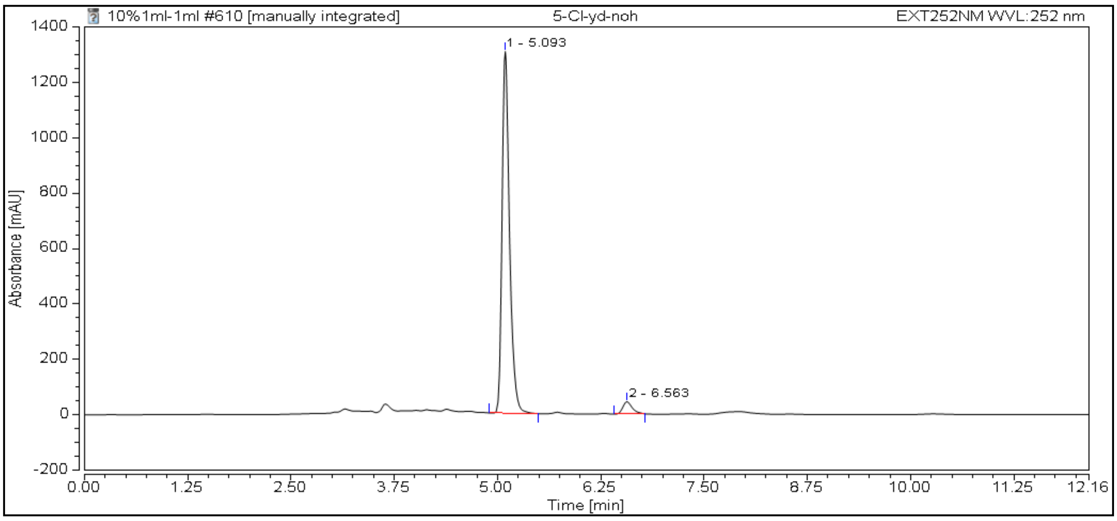

Integration Results					
No.	Peak Name	Retention Time min	Area mAU*min	Relative Area %	Amount n.a.
1		5.093	136.683	95.85	n.a.
2		6.563	5.921	4.15	n.a.
**Total:**			**142.605**	**100.00**	

Supplementary figure 149. HPLC chromatogram for **5e**

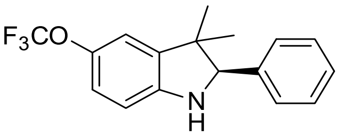

4f

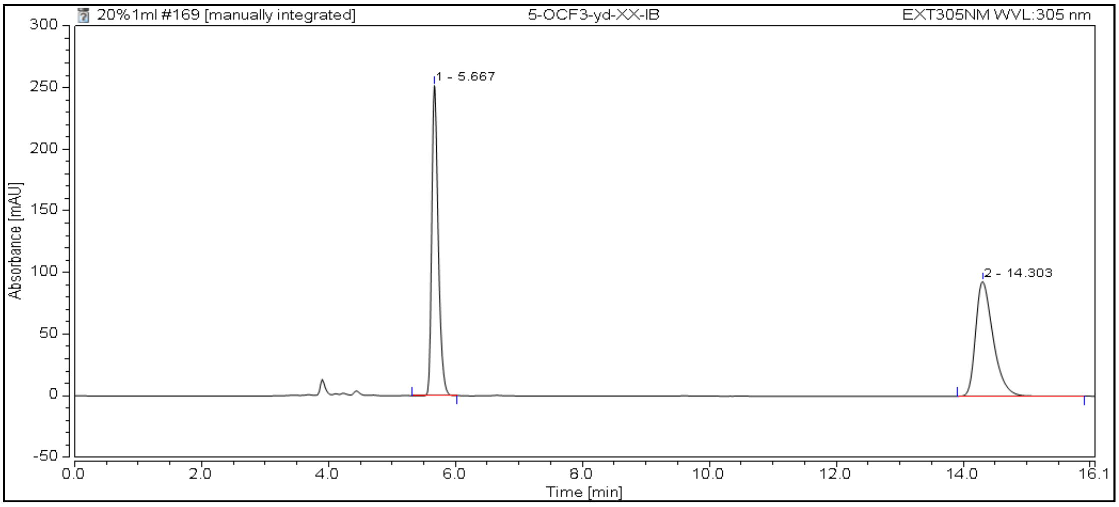

Integration Results

No.	Peak Name	Retention Time min	Area mAU*min	Relative Area %	Amount n.a.
1		5.667	29.631	49.88	n.a.
2		14.303	29.771	50.12	n.a.
**Total:**			**59.402**	**100.00**	

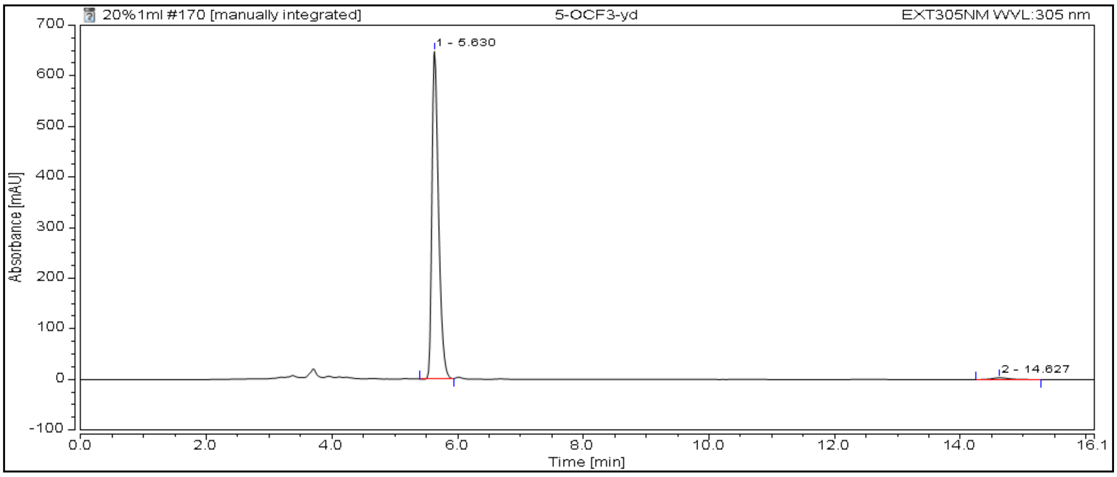

Integration Results

No.	Peak Name	Retention Time min	Area mAU*min	Relative Area %	Amount n.a.
1		5.630	78.806	98.65	n.a.
2		14.627	1.080	1.35	n.a.
**Total:**			**79.886**	**100.00**	

Supplementary figure 150. HPLC chromatogram for **4f**

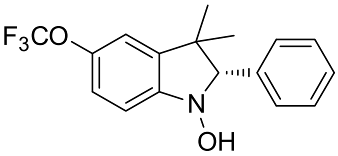

5f

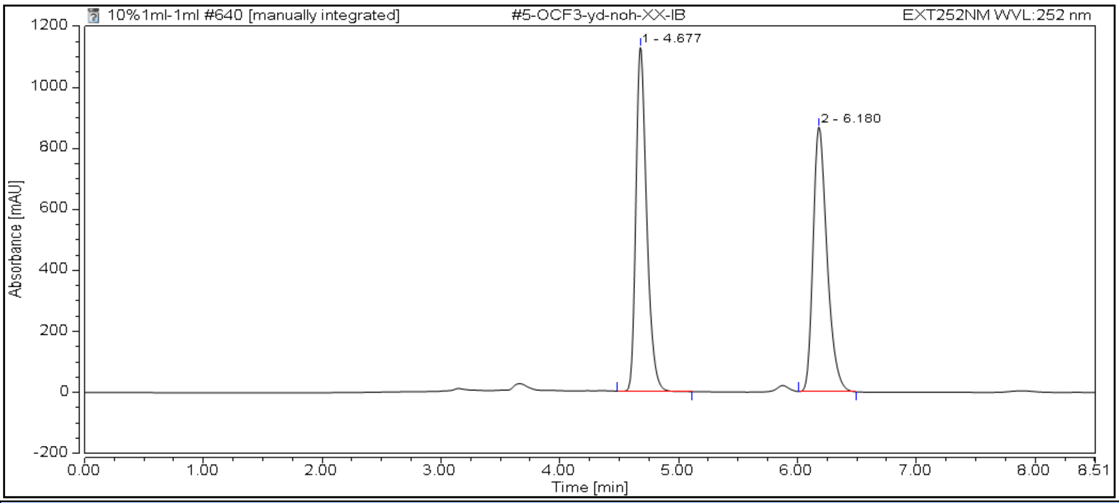

**Integration Results**					
No.	Peak Name	Retention Time min	Area mAU*min	Relative Area %	Amount n.a.
1		4.677	118.428	50.06	n.a.
2		6.180	118.157	49.94	n.a.
**Total:**			**236.585**	**100.00**	

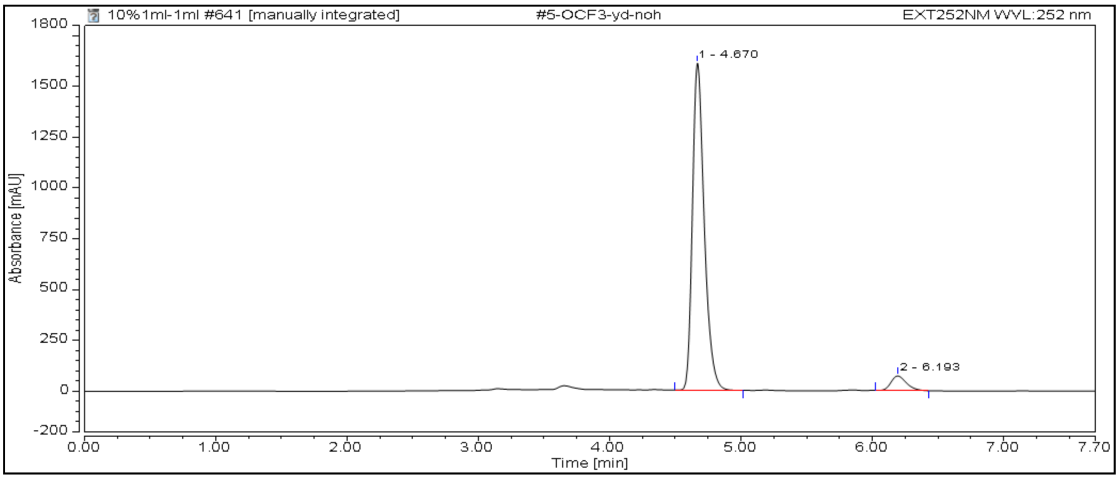

**Integration Results**					
No.	Peak Name	Retention Time min	Area mAU*min	Relative Area %	Amount n.a.
1		4.670	167.651	94.52	n.a.
2		6.193	9.715	5.48	n.a.
**Total:**			**177.366**	**100.00**	

Supplementary figure 151. HPLC chromatogram for **5f**

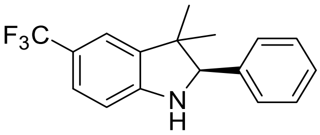

4g

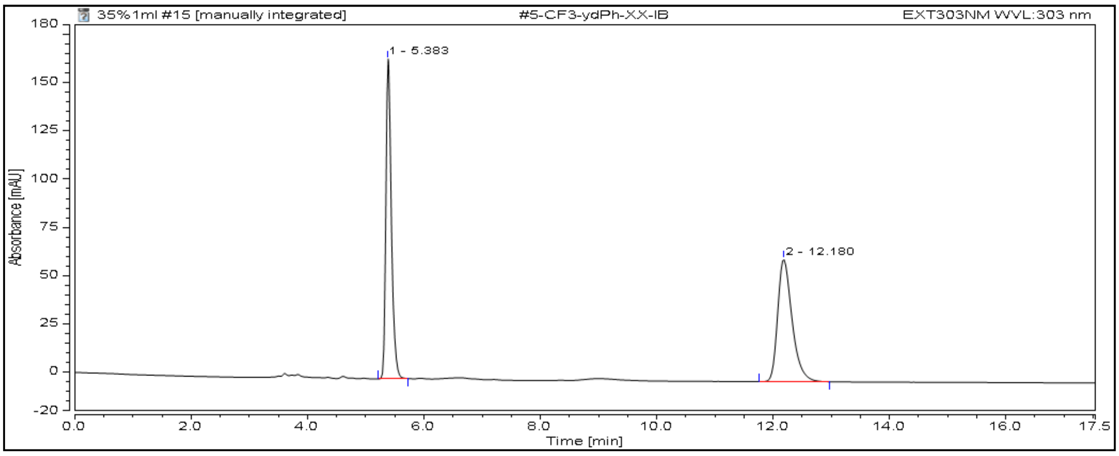

Integration Results					
No.	Peak Name	Retention Time min	Area mAU*min	Relative Area %	Amount n.a.
1		5.383	18.026	49.84	n.a.
2		12.180	18.144	50.16	n.a.
**Total:**			**36.169**	**100.00**	

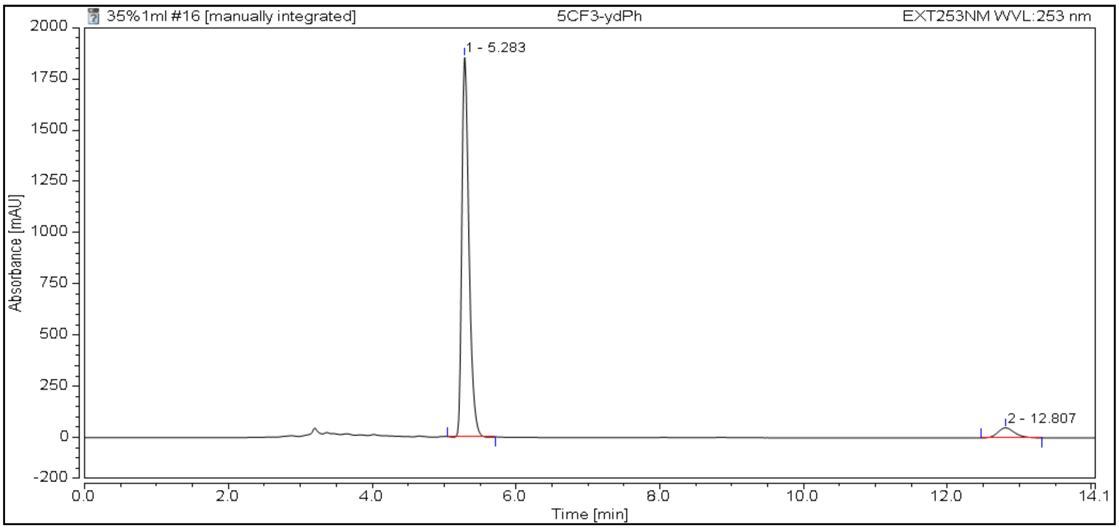

Integration Results					
No.	Peak Name	Retention Time min	Area mAU*min	Relative Area %	Amount n.a.
1		5.283	212.255	94.07	n.a.
2		12.807	13.389	5.93	n.a.
**Total:**			**225.644**	**100.00**	

Supplementary figure 152. HPLC chromatogram for **4g**

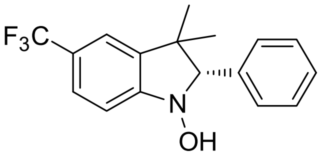

5g

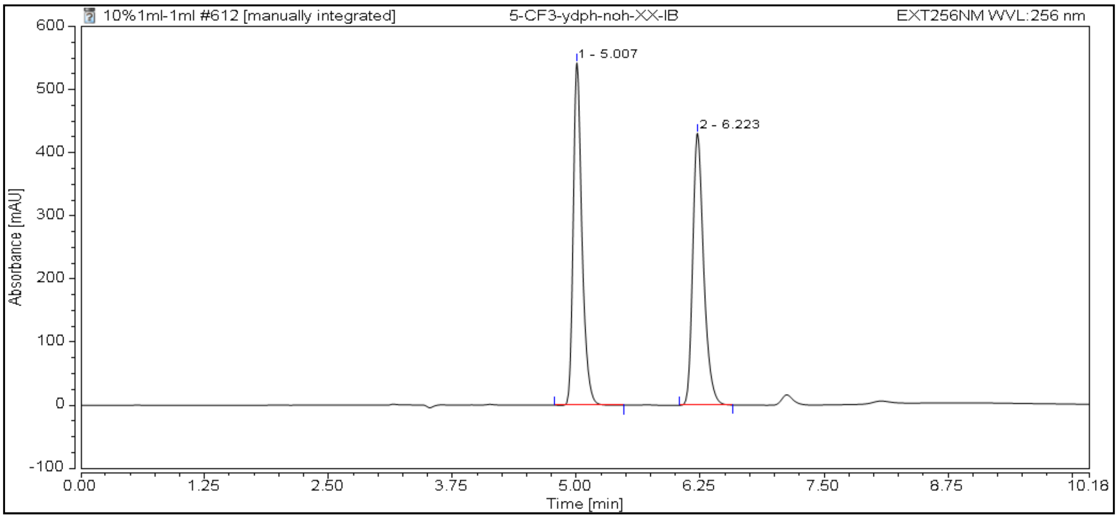

**Integration Results**					
No.	Peak Name	Retention Time min	Area mAU*min	Relative Area %	Amount n.a.
1		5.007	55.291	50.00	n.a.
2		6.223	55.286	50.00	n.a.
**Total:**			**110.577**	**100.00**	

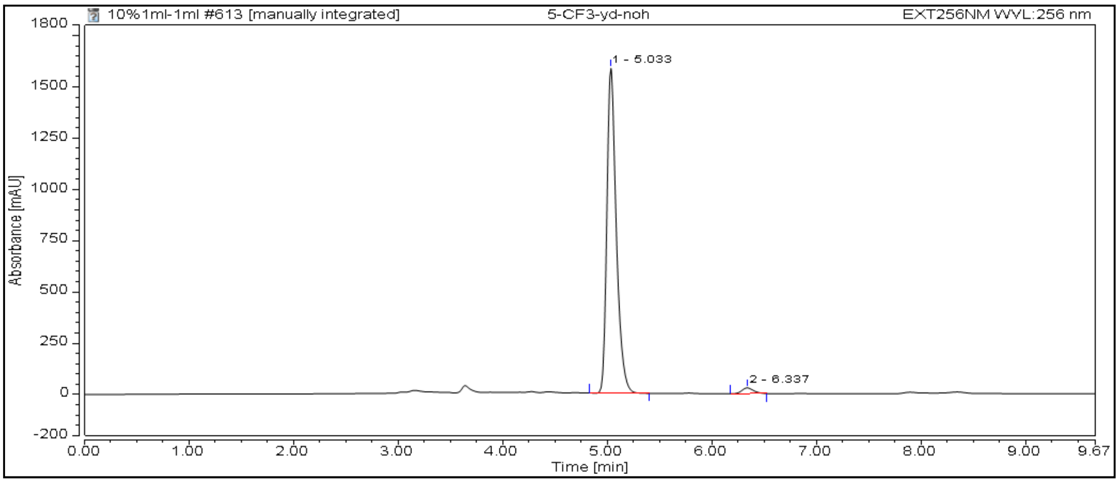

**Integration Results**					
No.	Peak Name	Retention Time min	Area mAU*min	Relative Area %	Amount n.a.
1		5.033	165.311	97.86	n.a.
2		6.337	3.612	2.14	n.a.
**Total:**			**168.922**	**100.00**	

Supplementary figure 153. HPLC chromatogram for **5g**

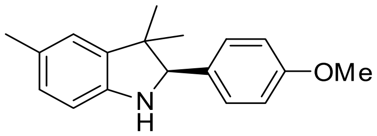

4h

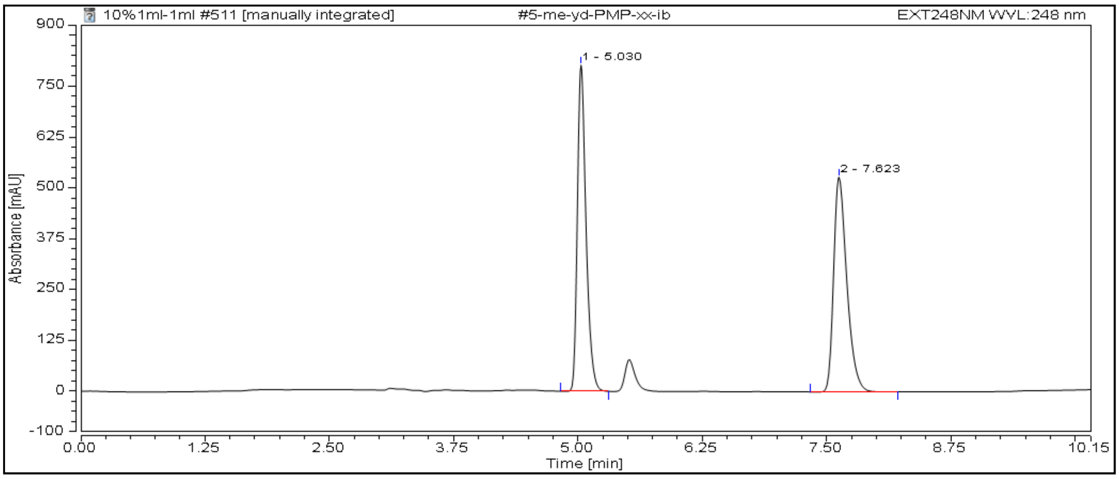

**Integration Results**					
No.	Peak Name	Retention Time min	Area mAU*min	Relative Area %	Amount n.a.
1		5.030	80.829	49.89	n.a.
2		7.623	81.188	50.11	n.a.
**Total:**			**162.016**	**100.00**	

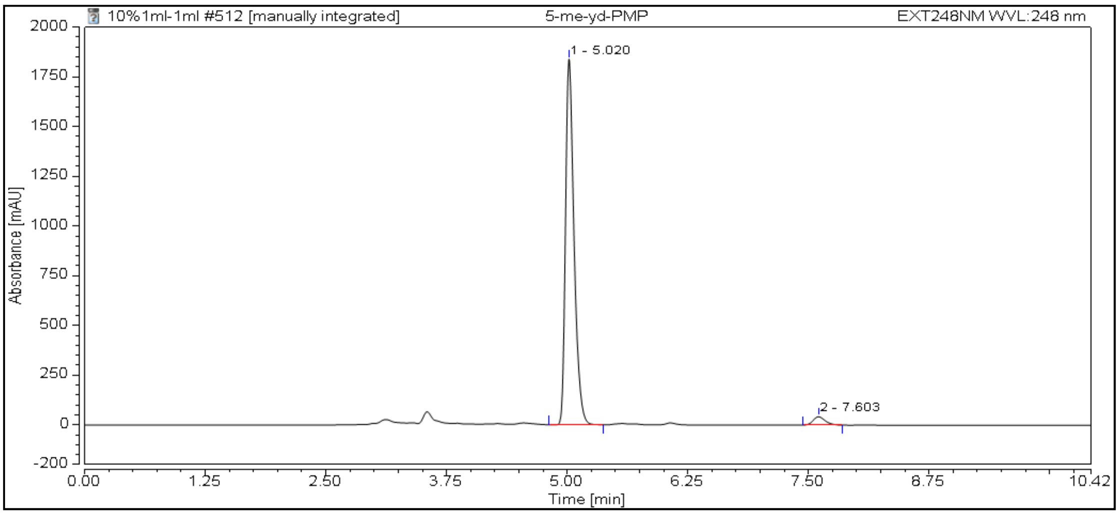

**Integration Results**					
No.	Peak Name	Retention Time min	Area mAU*min	Relative Area %	Amount n.a.
1		5.020	187.848	96.85	n.a.
2		7.603	6.112	3.15	n.a.
**Total:**			**193.960**	**100.00**	

Supplementary figure 154. HPLC chromatogram for **4h**

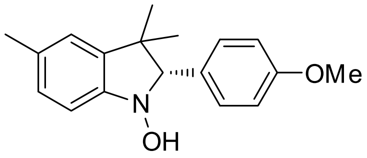

5h

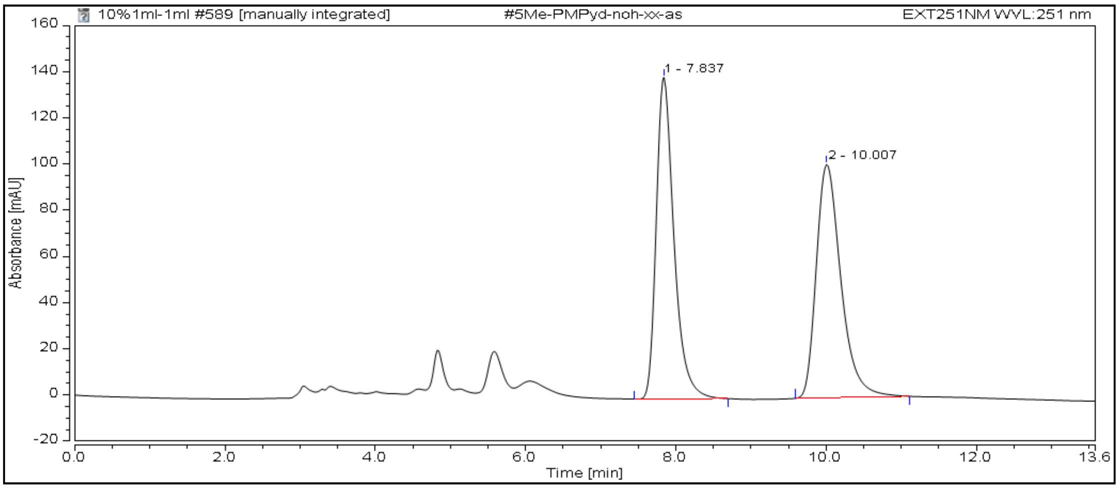

Integration Results					
No.	Peak Name	Retention Time min	Area mAU*min	Relative Area %	Amount n.a.
1		7.837	37.978	49.80	n.a.
2		10.007	38.286	50.20	n.a.
**Total:**			**76.264**	**100.00**	

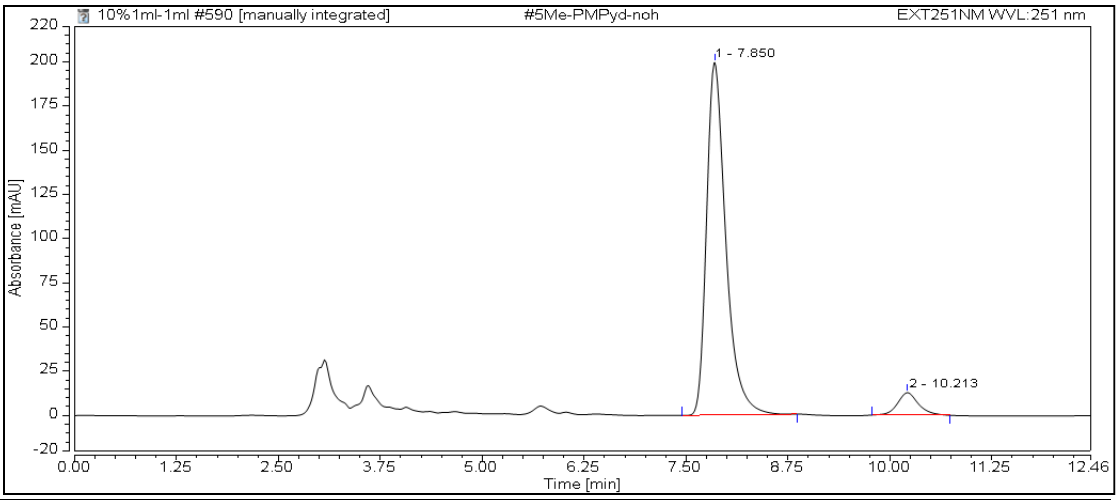

Integration Results					
No.	Peak Name	Retention Time min	Area mAU*min	Relative Area %	Amount n.a.
1		7.850	54.648	93.52	n.a.
2		10.213	3.785	6.48	n.a.
**Total:**			**58.433**	**100.00**	

Supplementary figure 155. HPLC chromatogram for **5h**

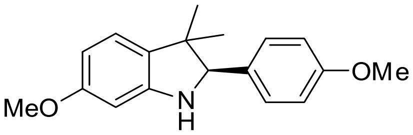

4i

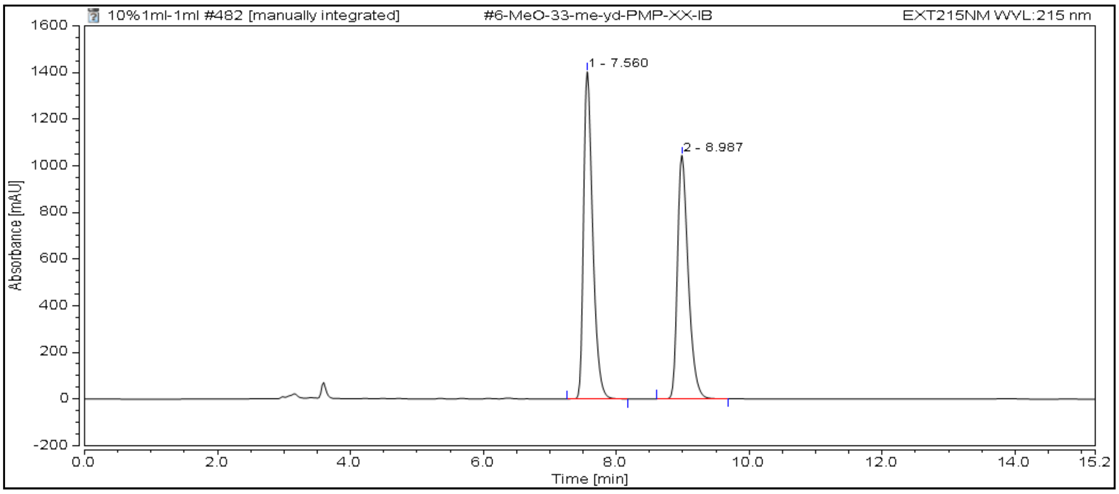

Integration Results

No.	Peak Name	Retention Time min	Area mAU*min	Relative Area %	Amount n.a.
1		7.560	223.138	53.25	n.a.
2		8.987	195.914	46.75	n.a.
**Total:**			**419.052**	**100.00**	

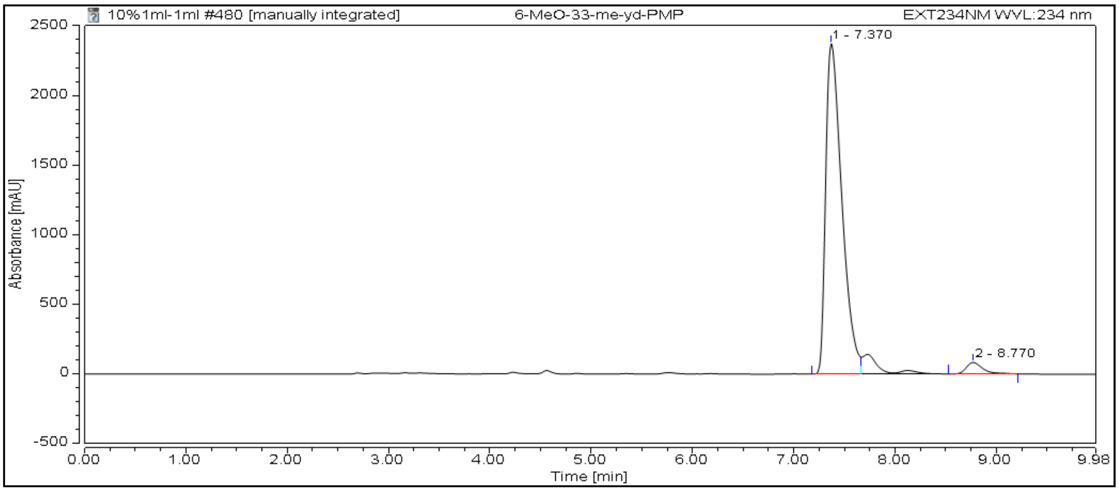

Integration Results

No.	Peak Name	Retention Time min	Area mAU*min	Relative Area %	Amount n.a.
1		7.370	438.812	96.55	n.a.
2		8.770	15.669	3.45	n.a.
**Total:**			**454.481**	**100.00**	

Supplementary figure 156. HPLC chromatogram for **4i**

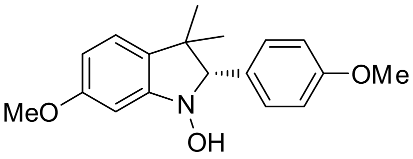

5i

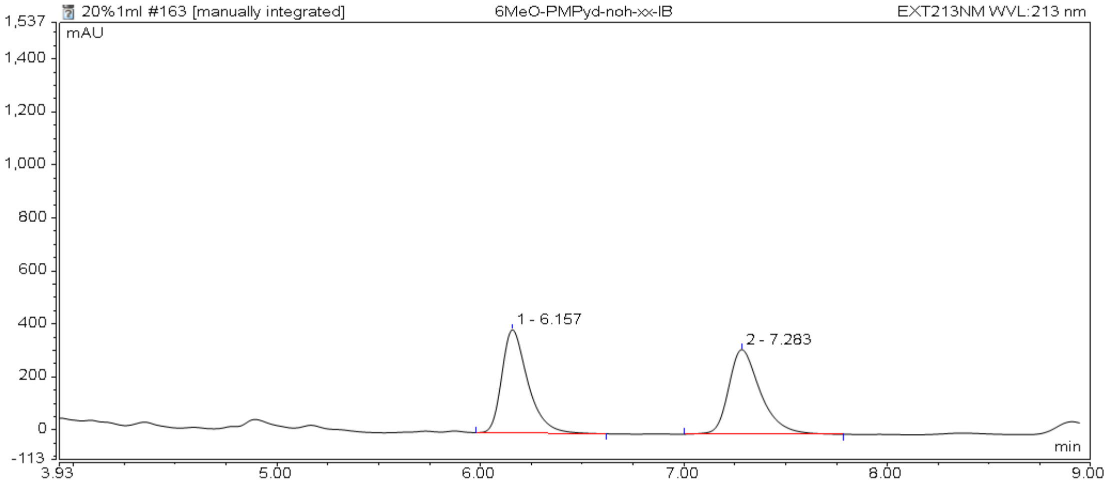

Integration Results					
No.	Peak Name	Retention Time min	Area mAU*min	Relative Area %	Amount n.a.
1		6.157	57.852	49.95	n.a.
2		7.283	57.979	50.05	n.a.
**Total:**			**115.831**	**100.00**	

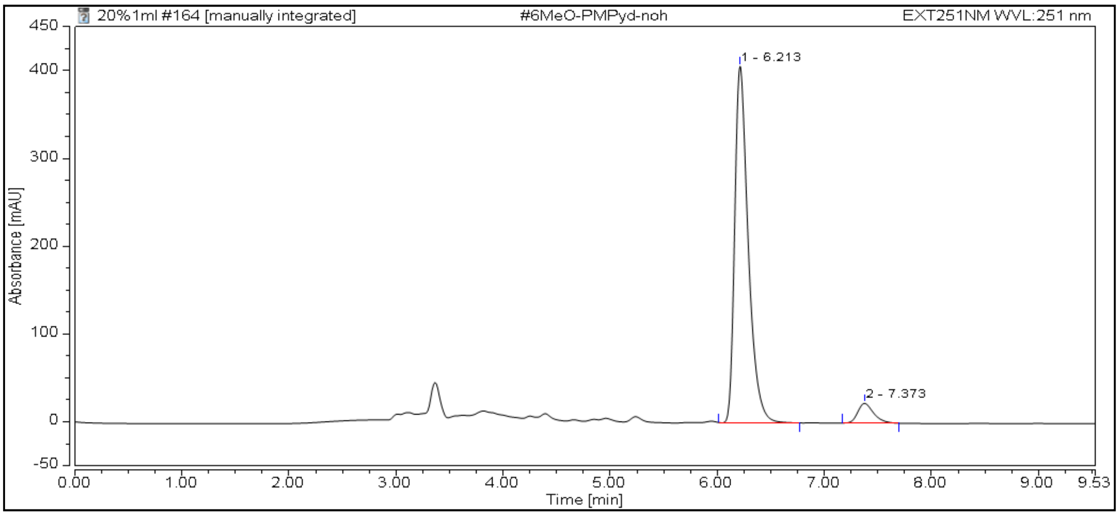

Integration Results					
No.	Peak Name	Retention Time min	Area mAU*min	Relative Area %	Amount n.a.
1		6.213	59.328	93.90	n.a.
2		7.373	3.854	6.10	n.a.
**Total:**			**63.182**	**100.00**	

Supplementary figure 157. HPLC chromatogram for **5i**

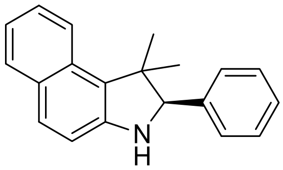

4j

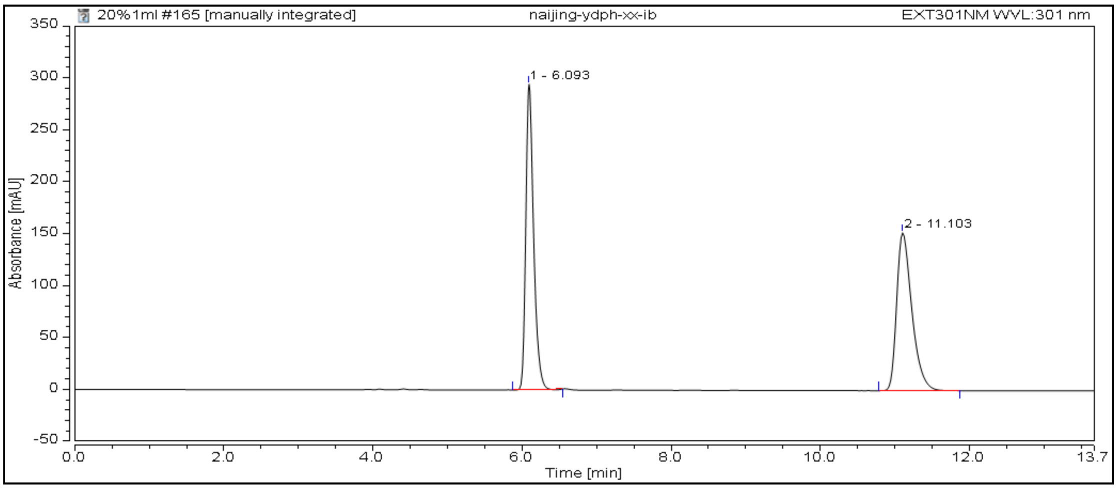

Integration Results

No.	Peak Name	Retention Time min	Area mAU*min	Relative Area %	Amount n.a.
1		6.093	35.546	49.79	n.a.
2		11.103	35.840	50.21	n.a.
**Total:**			**71.386**	**100.00**	

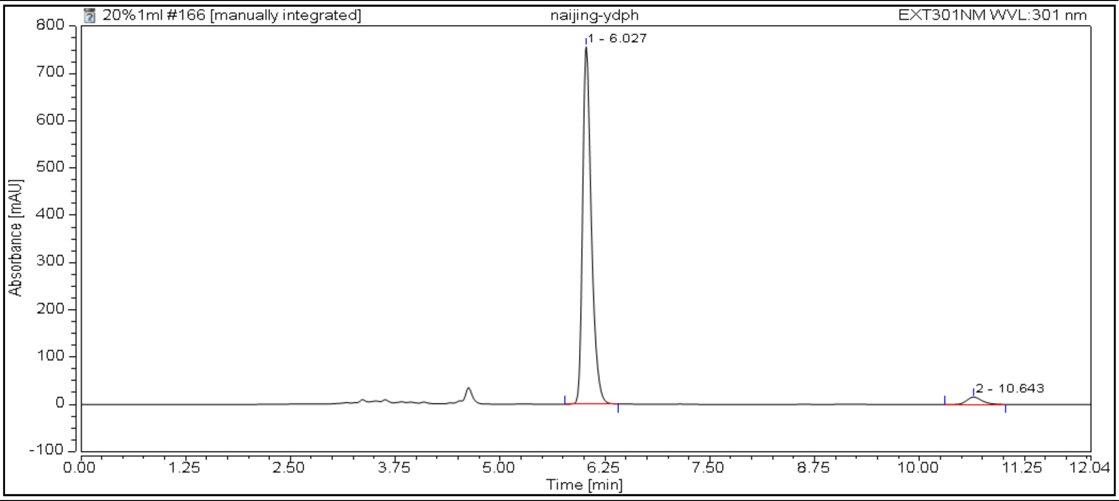

Integration Results

No.	Peak Name	Retention Time min	Area mAU*min	Relative Area %	Amount n.a.
1		6.027	91.432	96.53	n.a.
2		10.643	3.285	3.47	n.a.
**Total:**			**94.717**	**100.00**	

Supplementary figure 158. HPLC chromatogram for **4j**

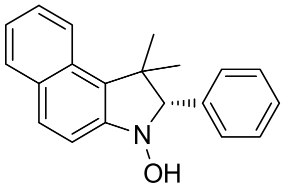

5j

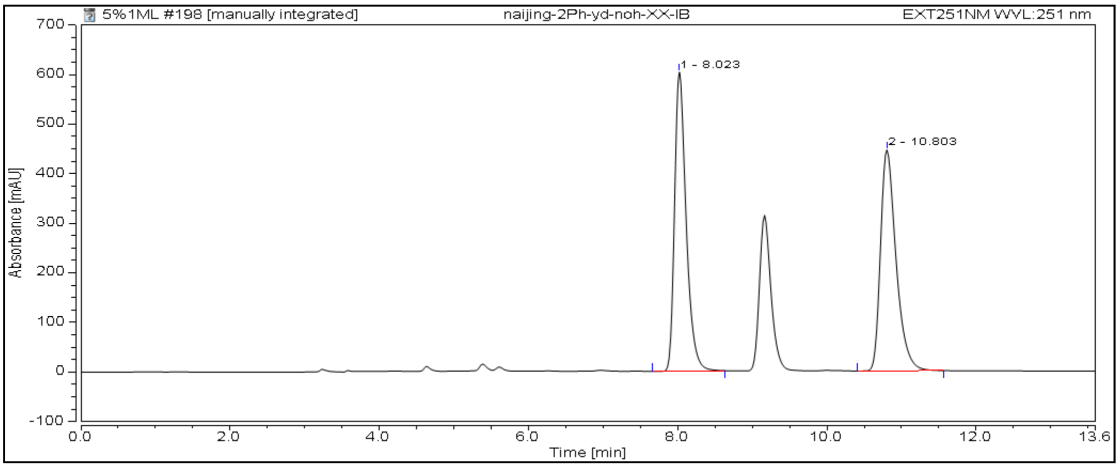

Integration Results					
No.	Peak Name	Retention Time min	Area mAU*min	Relative Area %	Amount n.a.
1		8.023	108.151	50.19	n.a.
2		10.803	107.335	49.81	n.a.
**Total:**			**215.487**	**100.00**	

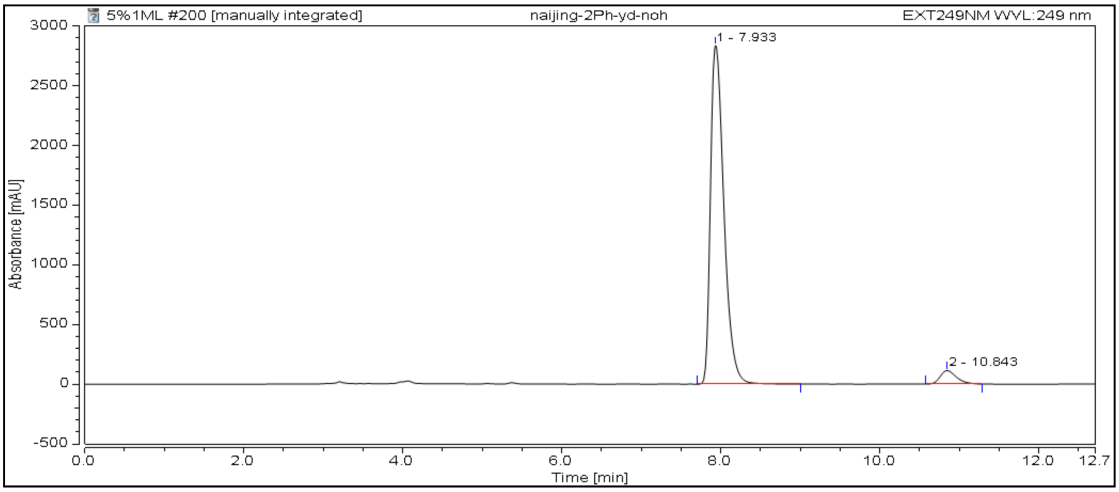

Integration Results					
No.	Peak Name	Retention Time min	Area mAU*min	Relative Area %	Amount n.a.
1		7.933	555.370	95.40	n.a.
2		10.843	26.787	4.60	n.a.
**Total:**			**582.157**	**100.00**	

Supplementary figure 159. HPLC chromatogram for **5j**

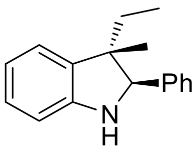

6a

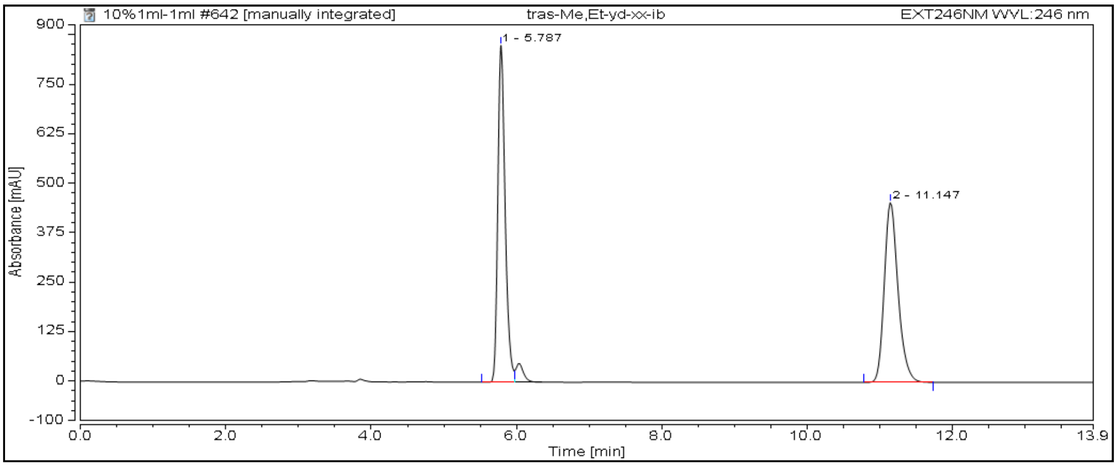

Integration Results

No.	Peak Name	Retention Time min	Area mAU*min	Relative Area %	Amount n.a.
1		5.787	99.388	49.92	n.a.
2		11.147	99.721	50.08	n.a.
**Total:**			**199.109**	**100.00**	

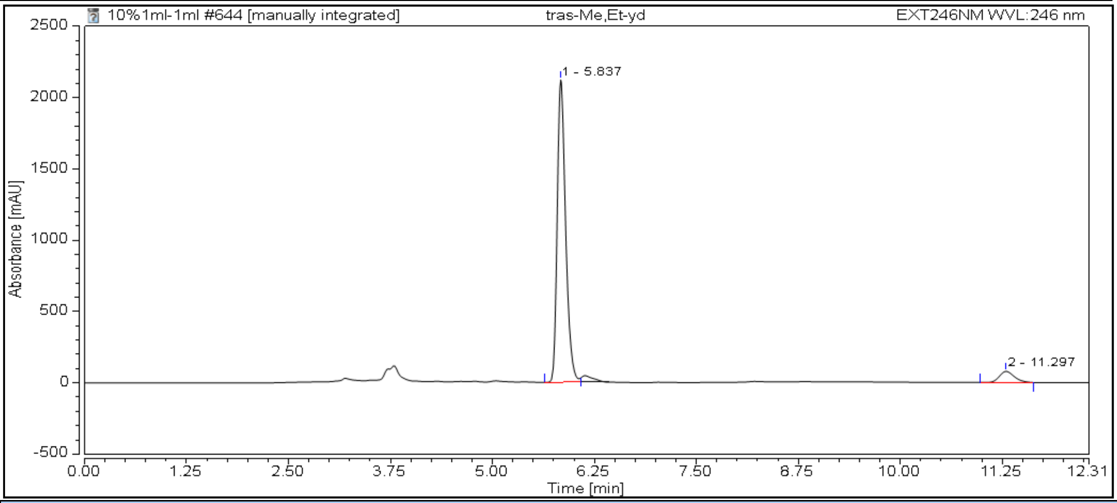

Integration Results

No.	Peak Name	Retention Time min	Area mAU*min	Relative Area %	Amount n.a.
1		5.837	253.152	93.85	n.a.
2		11.297	16.602	6.15	n.a.
**Total:**			**269.754**	**100.00**	

Supplementary figure 160. HPLC chromatogram for **6a**

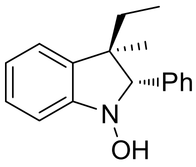

7a

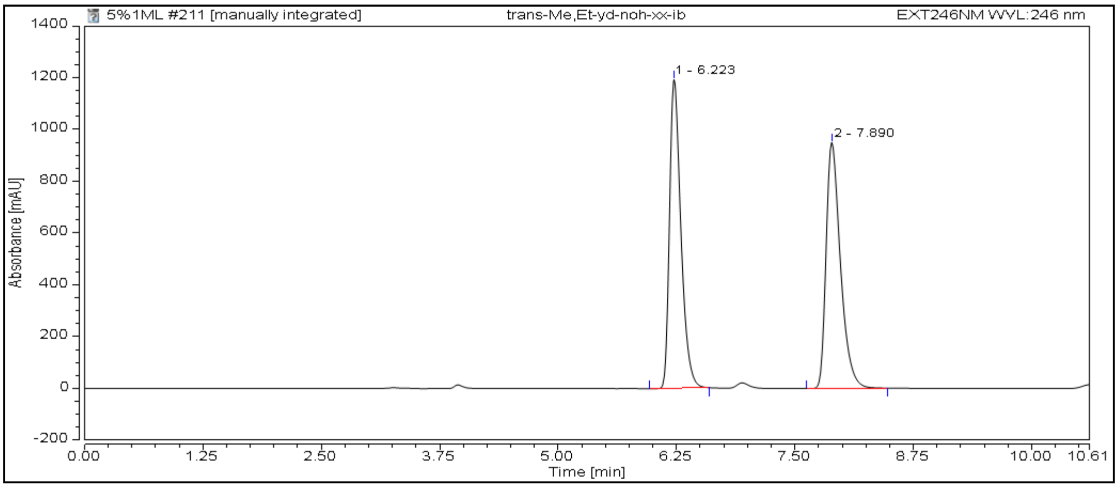

Integration Results					
No.	Peak Name	Retention Time min	Area mAU*min	Relative Area %	Amount n.a.
1		6.223	165.639	49.87	n.a.
2		7.890	166.491	50.13	n.a.
**Total:**			**332.130**	**100.00**	

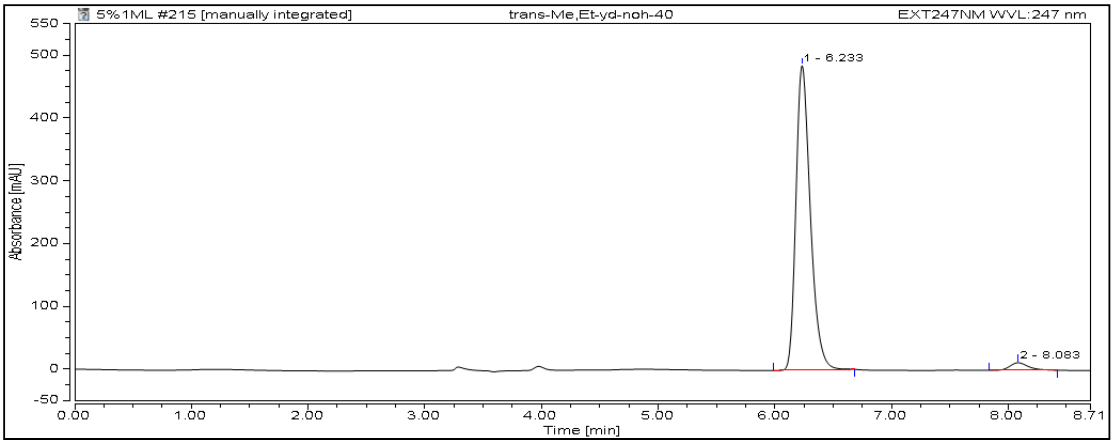

Integration Results					
No.	Peak Name	Retention Time min	Area mAU*min	Relative Area %	Amount n.a.
1		6.233	69.445	96.41	n.a.
2		8.083	2.586	3.59	n.a.
**Total:**			**71.628**	**100.00**	

Supplementary figure 161. HPLC chromatogram for **7a**

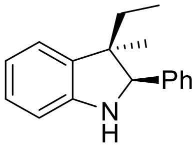

6b

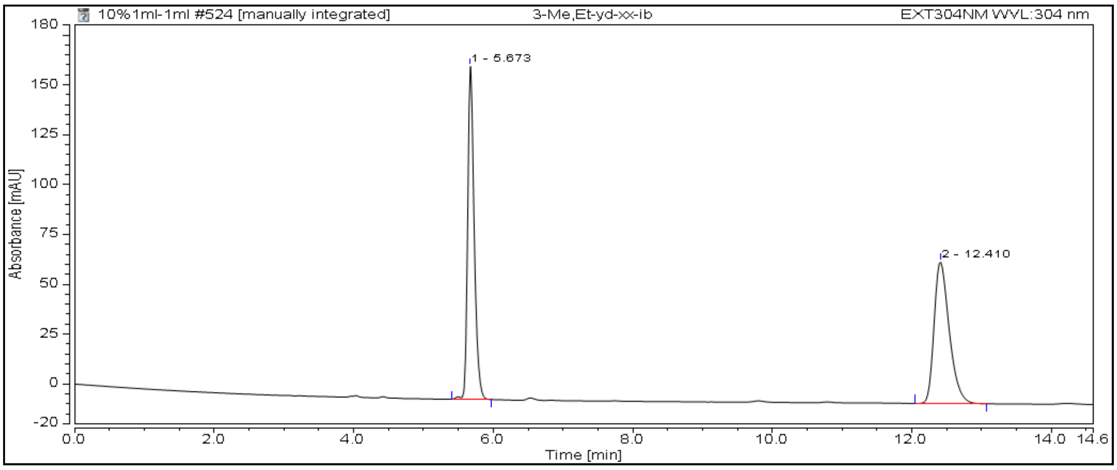

Integration Results					
No.	Peak Name	Retention Time min	Area mAU*min	Relative Area %	Amount n.a.
1		5.673	18.083	50.28	n.a.
2		12.410	17.883	49.72	n.a.
**Total:**			**35.966**	**100.00**	

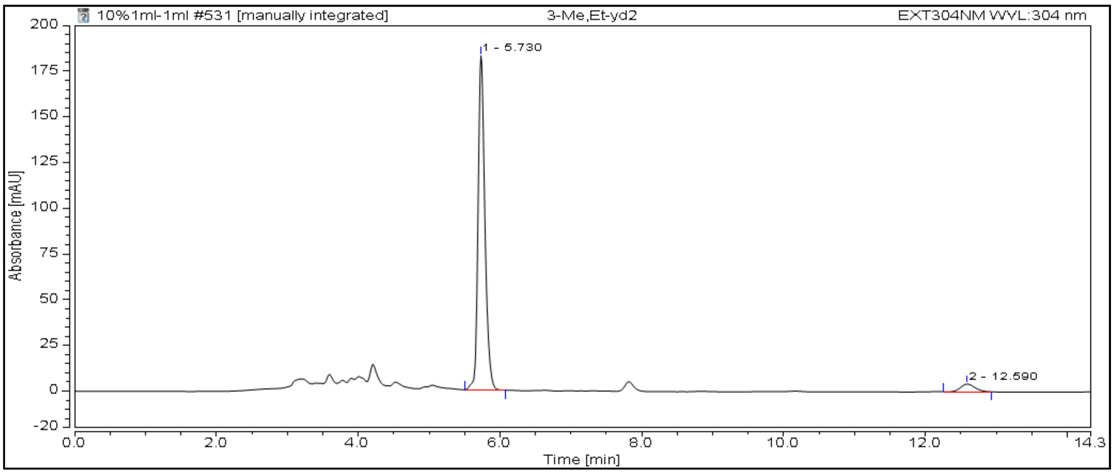

Integration Results					
No.	Peak Name	Retention Time min	Area mAU*min	Relative Area %	Amount n.a.
1		5.730	20.467	95.40	n.a.
2		12.590	0.986	4.60	n.a.
**Total:**			**21.454**	**100.00**	

Supplementary figure 162. HPLC chromatogram for **6b**

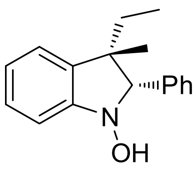

7b

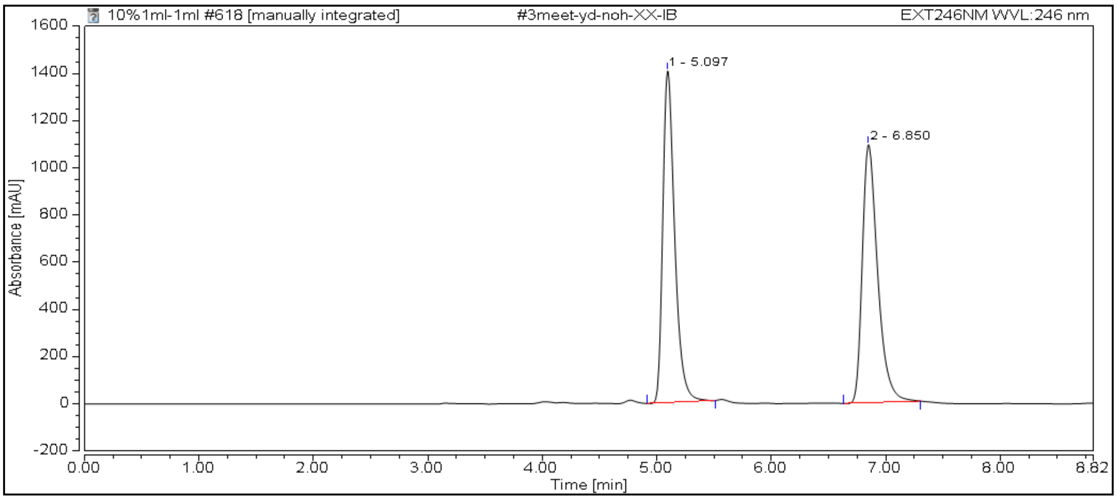

**Integration Results**					
No.	Peak Name	Retention Time min	Area mAU*min	Relative Area %	Amount n.a.
1		5.097	168.855	49.86	n.a.
2		6.850	169.784	50.14	n.a.
**Total:**			**338.639**	**100.00**	

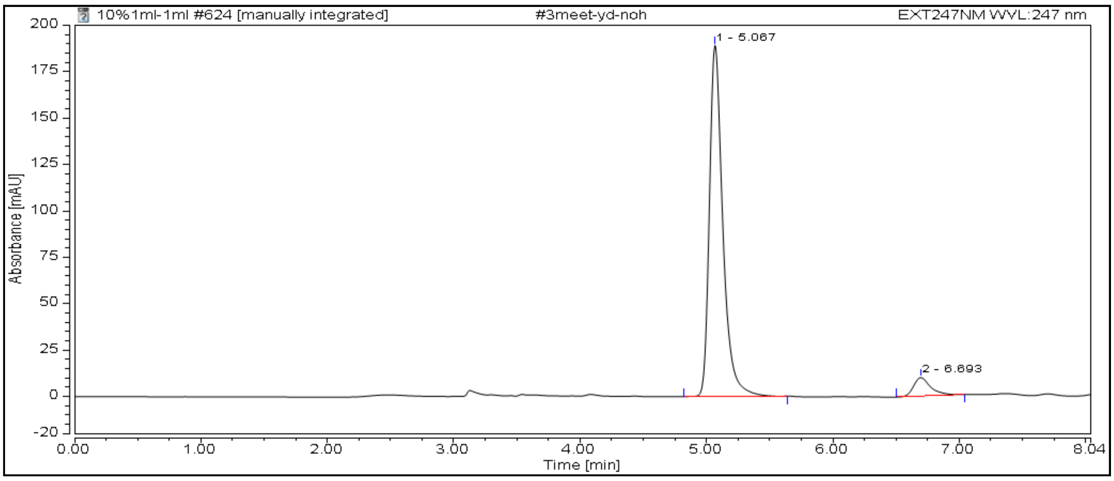

**Integration Results**					
No.	Peak Name	Retention Time min	Area mAU*min	Relative Area %	Amount n.a.
1		5.067	23.219	93.66	n.a.
2		6.693	1.572	6.34	n.a.
**Total:**			**24.792**	**100.00**	

Supplementary figure 163. HPLC chromatogram for **7b**

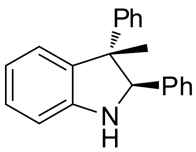

6c

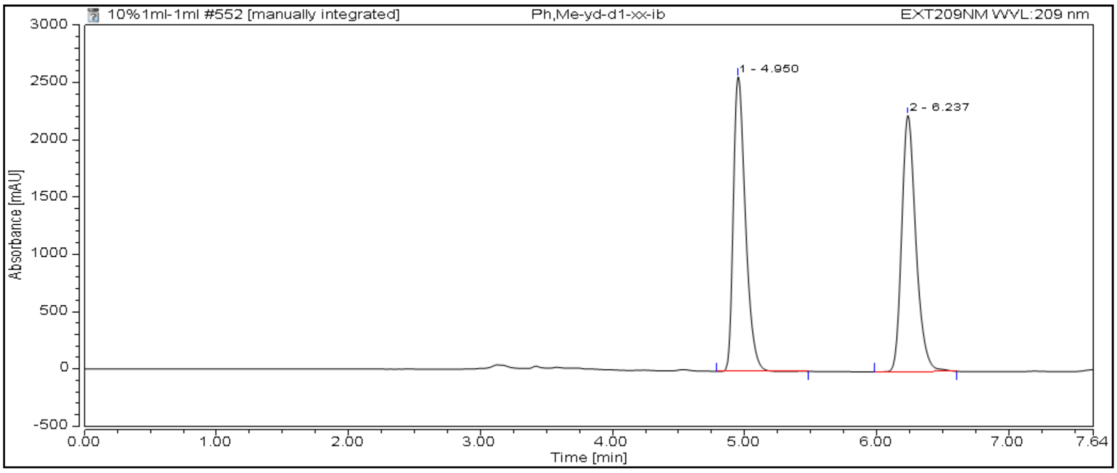

Integration Results					
No.	Peak Name	Retention Time min	Area mAU*min	Relative Area %	Amount n.a.
1		4.950	272.661	49.44	n.a.
2		6.237	278.789	50.56	n.a.
**Total:**			**551.450**	**100.00**	

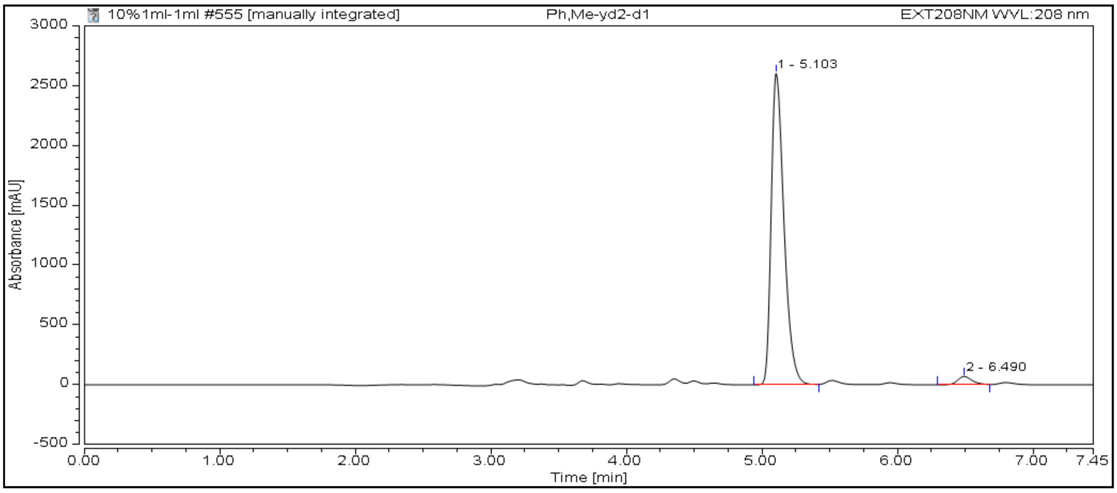

Integration Results					
No.	Peak Name	Retention Time min	Area mAU*min	Relative Area %	Amount n.a.
1		5.103	283.195	97.15	n.a.
2		6.490	8.296	2.85	n.a.
**Total:**			**291.491**	**100.00**	

Supplementary figure 164. HPLC chromatogram for **6c**

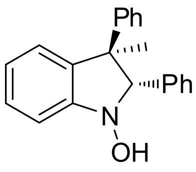

7c

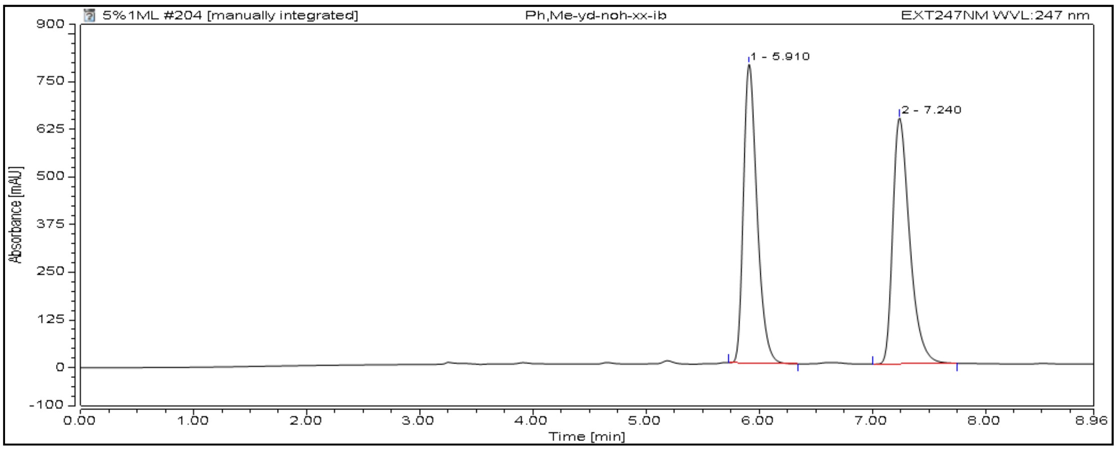

Integration Results					
No.	Peak Name	Retention Time min	Area mAU*min	Relative Area %	Amount n.a.
1		5.910	108.965	49.41	n.a.
2		7.240	111.568	50.59	n.a.
**Total:**			**220.533**	**100.00**	

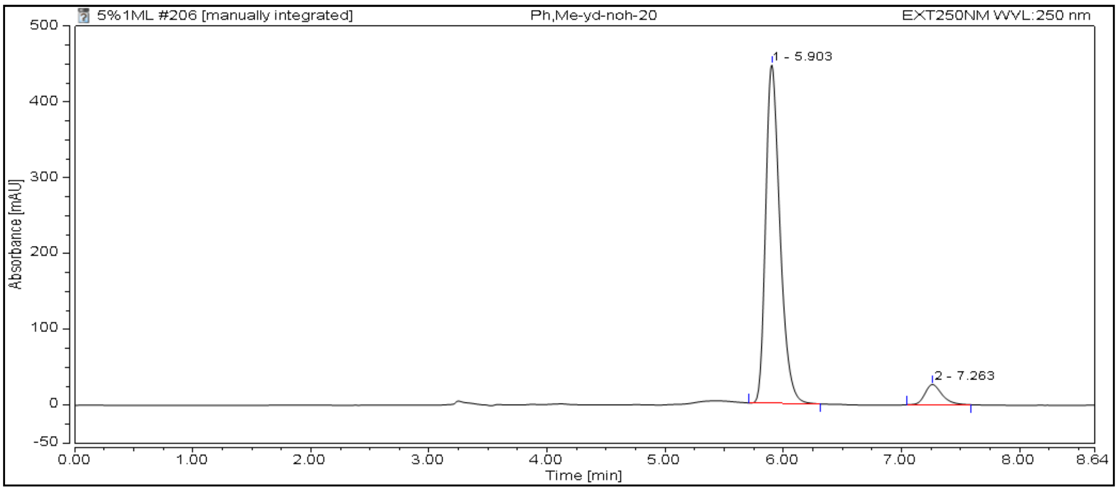

Integration Results					
No.	Peak Name	Retention Time min	Area mAU*min	Relative Area %	Amount n.a.
1		5.903	62.732	93.35	n.a.
2		7.263	4.471	6.65	n.a.
**Total:**			**67.204**	**100.00**	

Supplementary figure 165. HPLC chromatogram for **7c**

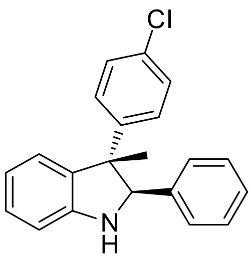

6d

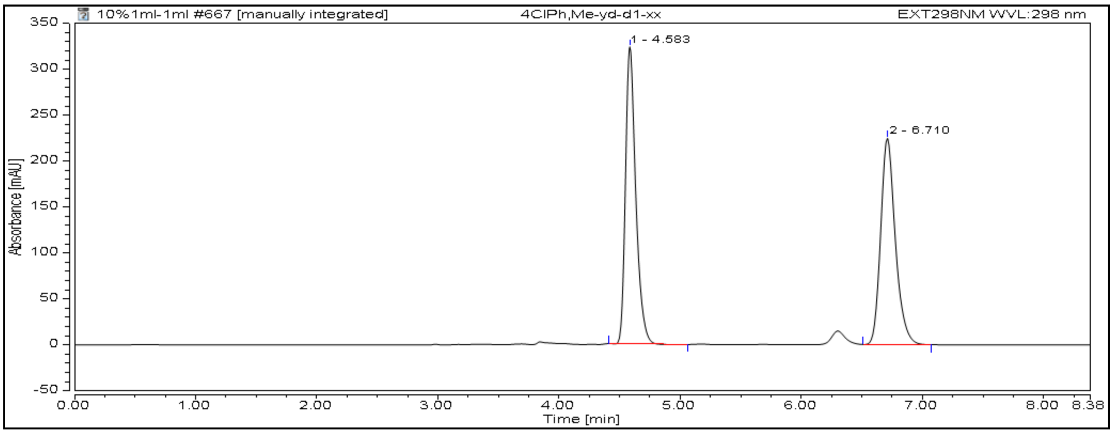

Integration Results					
No.	Peak Name	Retention Time min	Area mAU*min	Relative Area %	Amount n.a.
1		4.583	31.618	50.10	n.a.
2		6.710	31.491	49.90	n.a.
**Total:**			**63.110**	**100.00**	

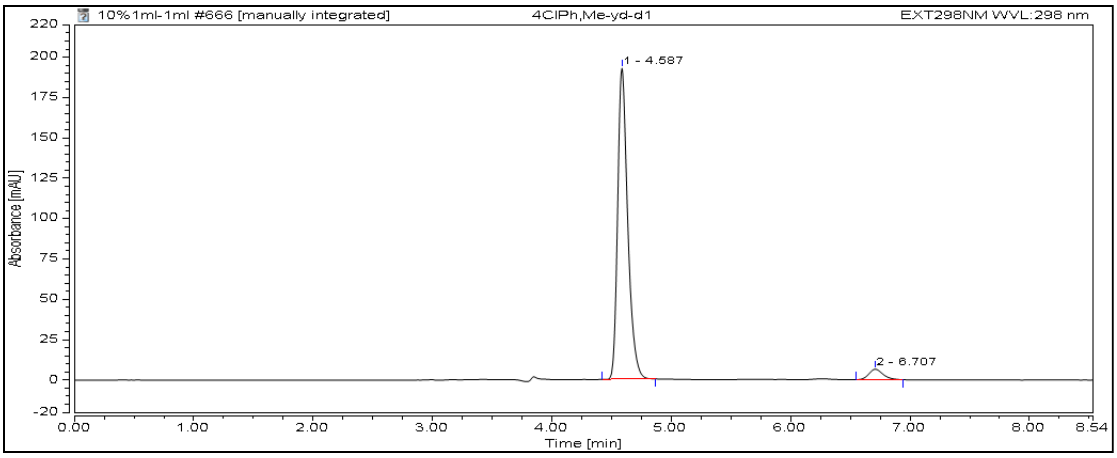

Integration Results					
No.	Peak Name	Retention Time min	Area mAU*min	Relative Area %	Amount n.a.
1		4.587	18.813	95.45	n.a.
2		6.707	0.897	4.55	n.a.
**Total:**			**19.710**	**100.00**	

Supplementary figure 166. HPLC chromatogram for **6d**

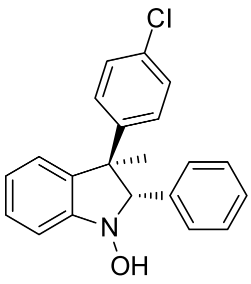

7d

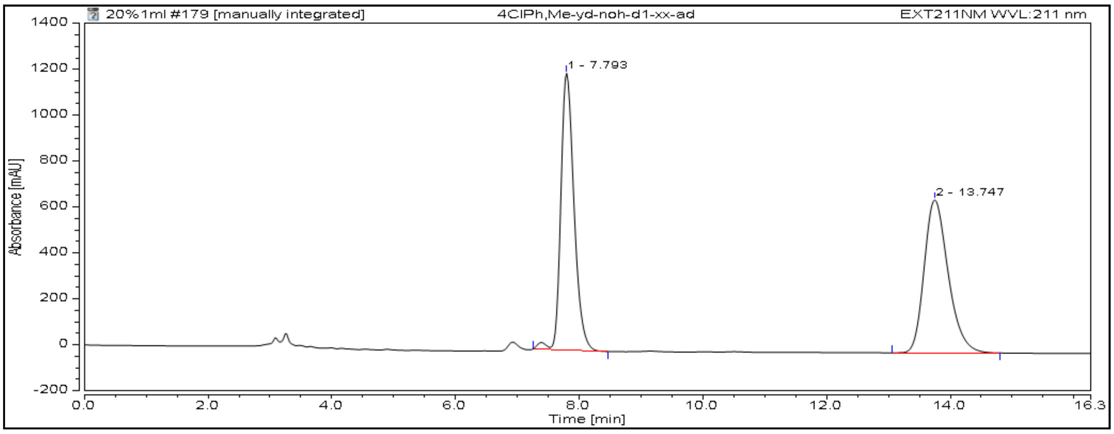

Integration Results

No.	Peak Name	Retention Time min	Area mAU*min	Relative Area %	Amount n.a.
1		7.793	299.606	50.02	n.a.
2		13.747	299.366	49.98	n.a.
**Total:**			**598.972**	**100.00**	

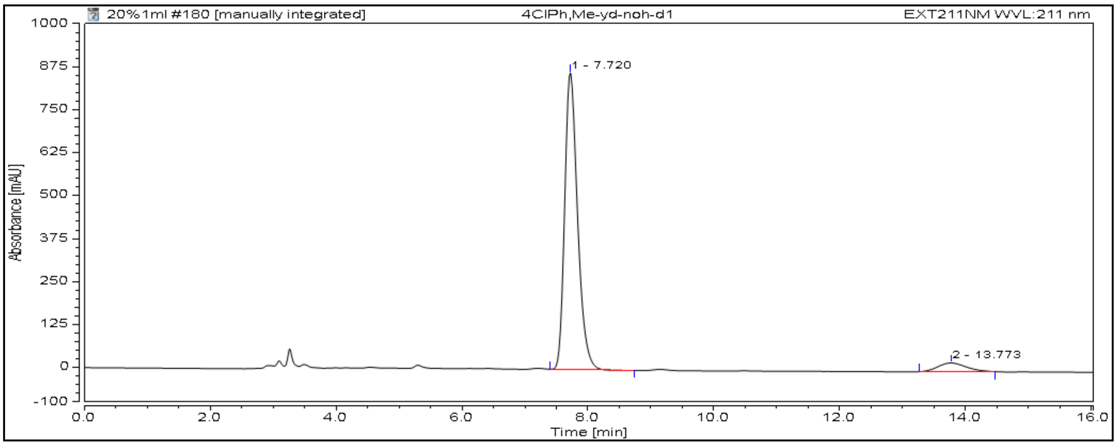

Integration Results

No.	Peak Name	Retention Time min	Area mAU*min	Relative Area %	Amount n.a.
1		7.720	204.297	93.95	n.a.
2		13.773	13.152	6.05	n.a.
**Total:**			**217.449**	**100.00**	

Supplementary figure 167. HPLC chromatogram for **7d**

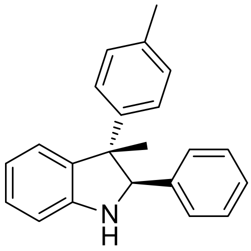

6e

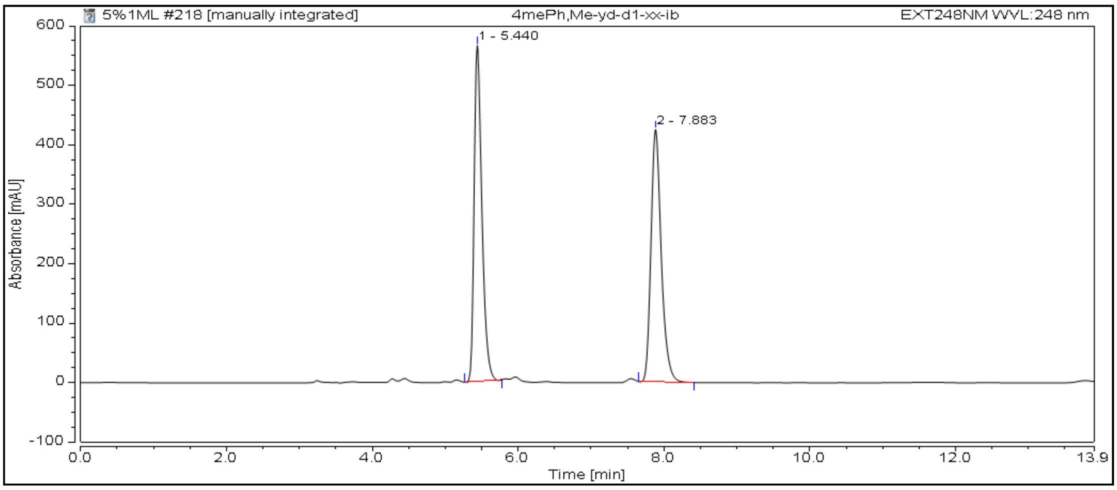

Integration Results					
No.	Peak Name	Retention Time min	Area mAU*min	Relative Area %	Amount n.a.
1		5.440	69.443	50.16	n.a.
2		7.883	69.008	49.84	n.a.
**Total:**			**138.451**	**100.00**	

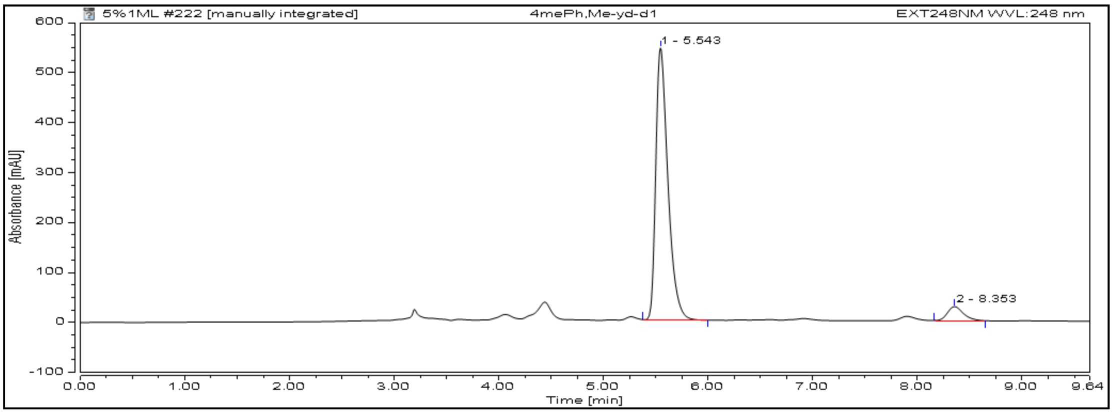

Integration Results					
No.	Peak Name	Retention Time min	Area mAU*min	Relative Area %	Amount n.a.
1		5.543	73.047	93.67	n.a.
2		8.353	4.940	6.33	n.a.
**Total:**			**77.988**	**100.00**	

Supplementary figure 168. HPLC chromatogram for **6e**

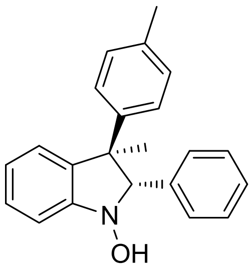

7e

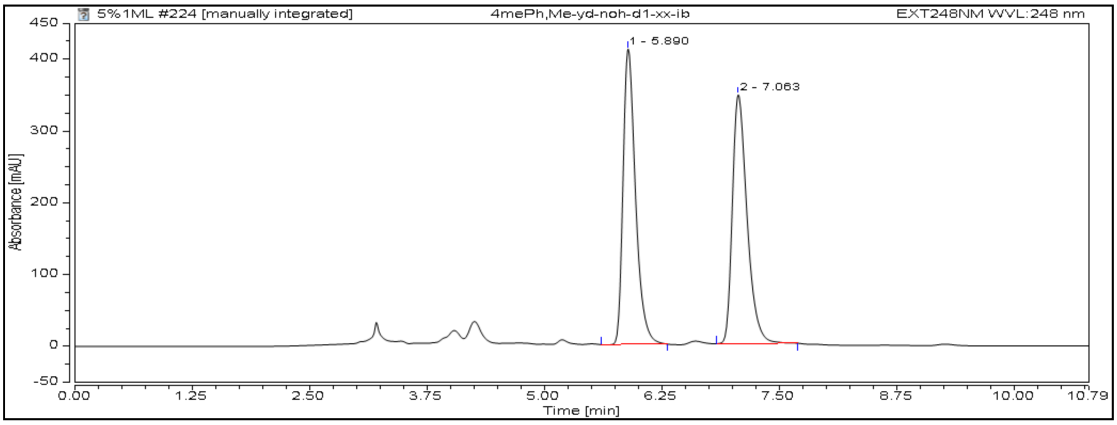

Integration Results					
No.	Peak Name	Retention Time min	Area mAU*min	Relative Area %	Amount n.a.
1		5.890	63.293	50.23	n.a.
2		7.063	62.708	49.77	n.a.
**Total:**			**126.001**	**100.00**	

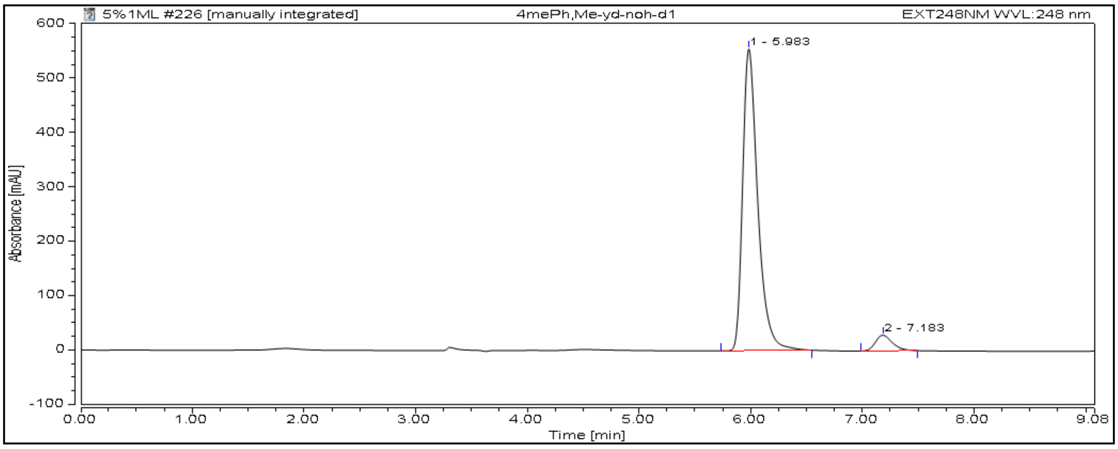

Integration Results					
No.	Peak Name	Retention Time min	Area mAU*min	Relative Area %	Amount n.a.
1		5.983	86.859	94.47	n.a.
2		7.183	5.086	5.53	n.a.
**Total:**			**91.944**	**100.00**	

Supplementary figure 169. HPLC chromatogram for **7e**

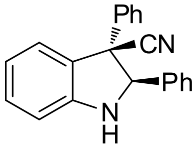

6f

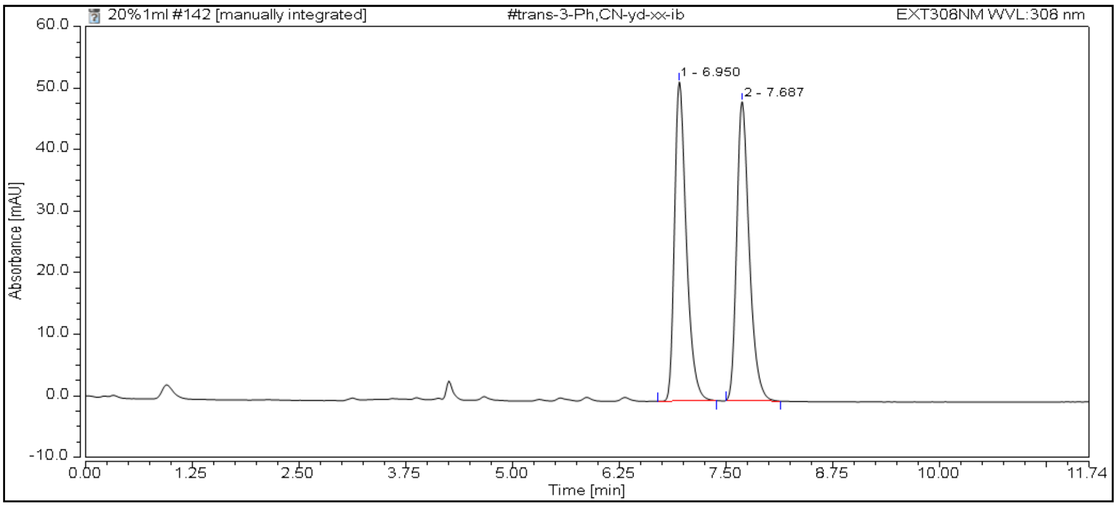

**Integration Results**					
No.	Peak Name	Retention Time min	Area mAU*min	Relative Area %	Amount n.a.
1		6.950	8.309	50.05	n.a.
2		7.687	8.294	49.95	n.a.
**Total:**			**16.603**	**100.00**	

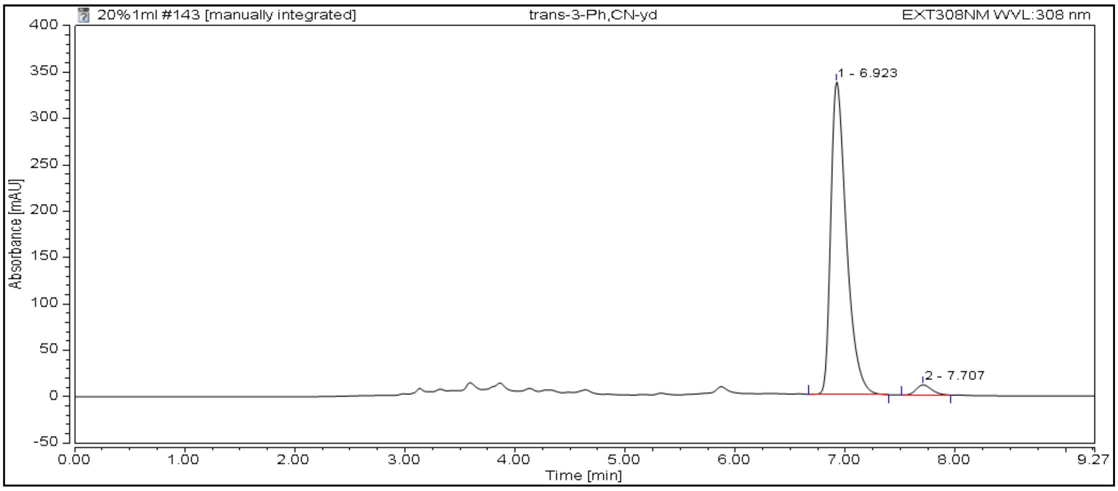

**Integration Results**					
No.	Peak Name	Retention Time min	Area mAU*min	Relative Area %	Amount n.a.
1		6.923	55.459	96.89	n.a.
2		7.707	1.778	3.11	n.a.
**Total:**			**57.237**	**100.00**	

Supplementary figure 170. HPLC chromatogram for **6f**

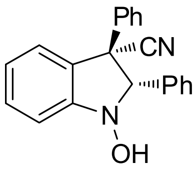

7f

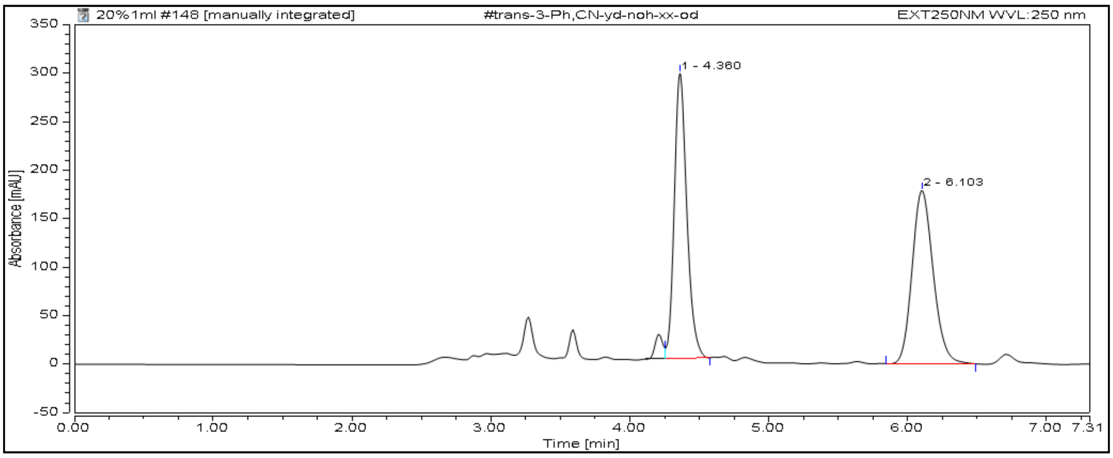

Integration Results

No.	Peak Name	Retention Time min	Area mAU*min	Relative Area %	Amount n.a.
1		4.360	30.708	49.49	n.a.
2		6.103	31.336	50.51	n.a.
**Total:**			**62.045**	**100.00**	

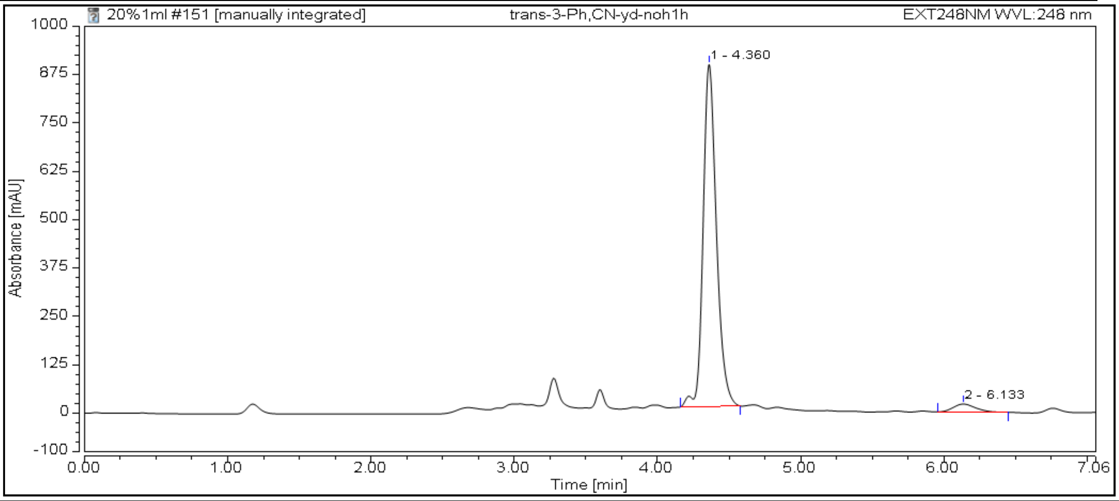

Integration Results

No.	Peak Name	Retention Time min	Area mAU*min	Relative Area %	Amount n.a.
1		4.360	95.224	96.20	n.a.
2		6.133	3.764	3.80	n.a.
**Total:**			**98.988**	**100.00**	

Supplementary figure 171. HPLC chromatogram for **7f**

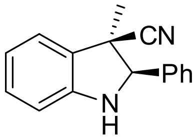

6g

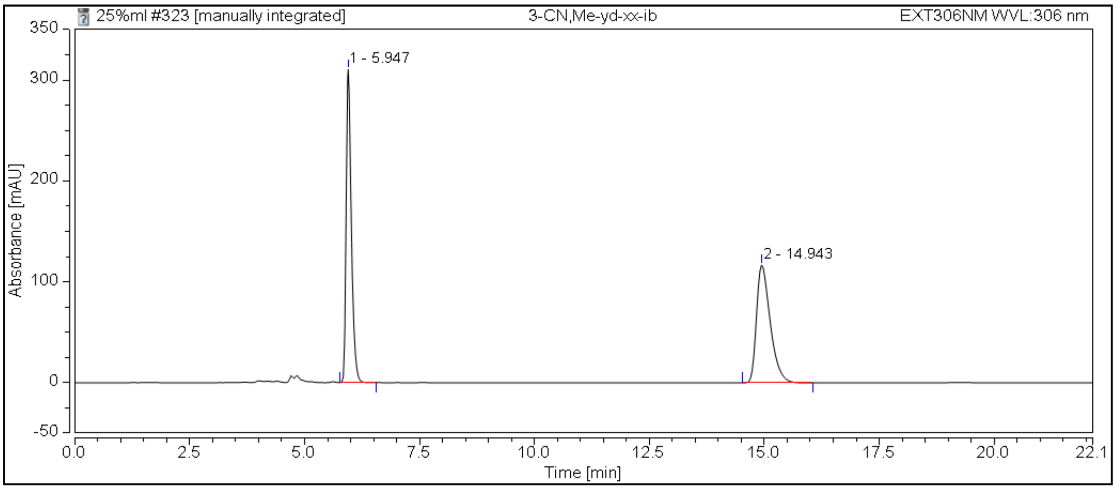

Integration Results					
No.	Peak Name	Retention Time min	Area mAU*min	Relative Area %	Amount n.a.
1		5.947	40.385	49.94	n.a.
2		14.943	40.490	50.06	n.a.
**Total:**			**80.874**	**100.00**	

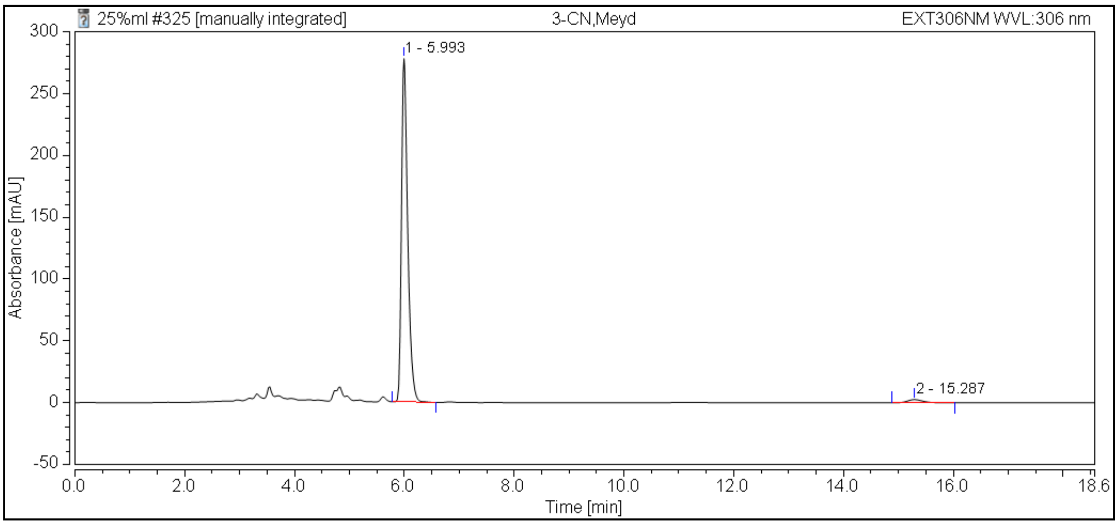

Integration Results					
No.	Peak Name	Retention Time min	Area mAU*min	Relative Area %	Amount n.a.
1		5.993	36.465	97.65	n.a.
2		15.287	0.878	2.35	n.a.
**Total:**			**37.343**	**100.00**	

Supplementary figure 172. HPLC chromatogram for **6g**

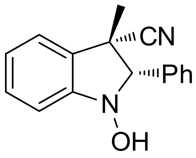

7g

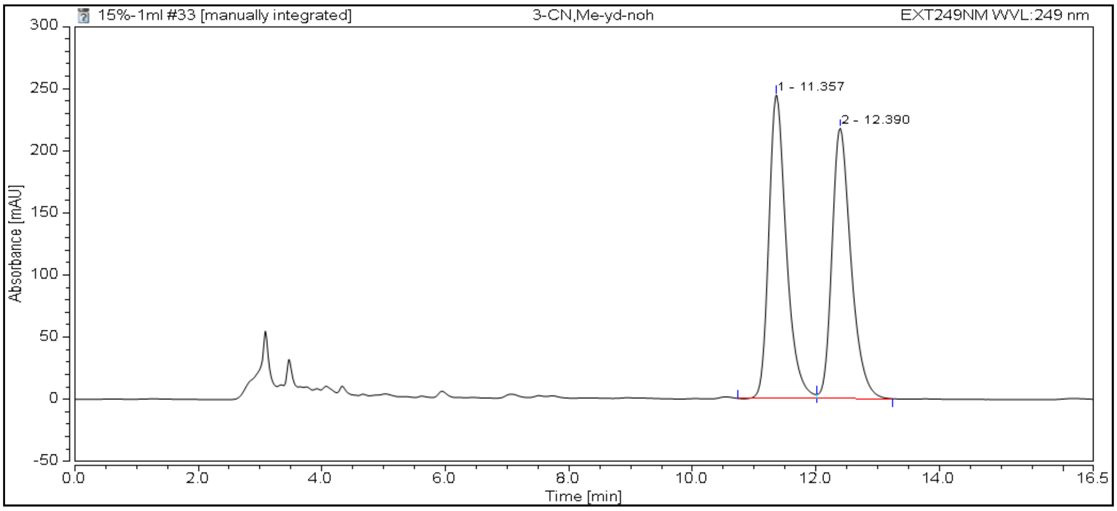

Integration Results					
No.	Peak Name	Retention Time min	Area mAU*min	Relative Area %	Amount n.a.
1		11.357	80.228	50.77	n.a.
2		12.390	77.784	49.23	n.a.
**Total:**			**158.013**	**100.00**	

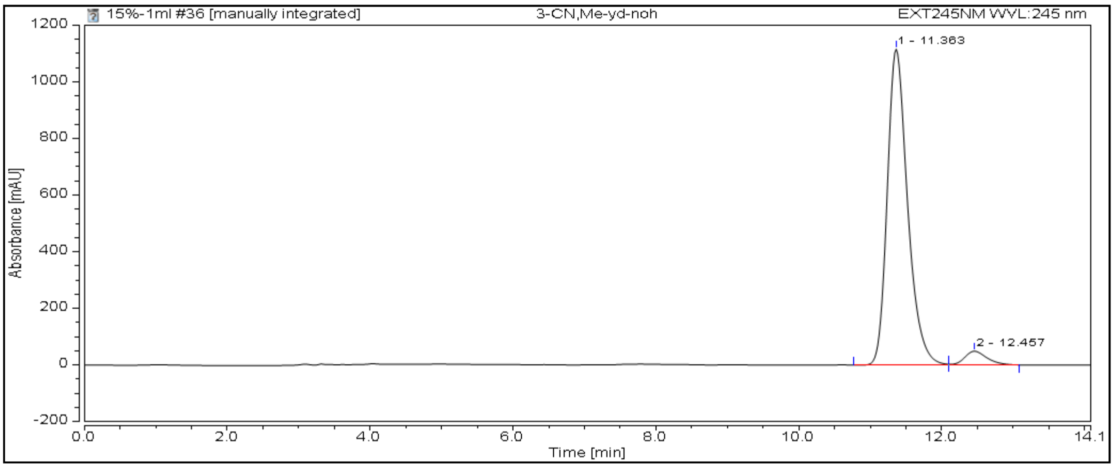

Integration Results					
No.	Peak Name	Retention Time min	Area mAU*min	Relative Area %	Amount n.a.
1		11.363	367.203	95.40	n.a.
2		12.457	17.696	4.60	n.a.
**Total:**			**384.899**	**100.00**	

Supplementary figure 173. HPLC chromatogram for **7g**

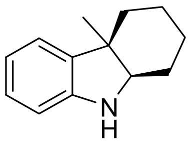

6h

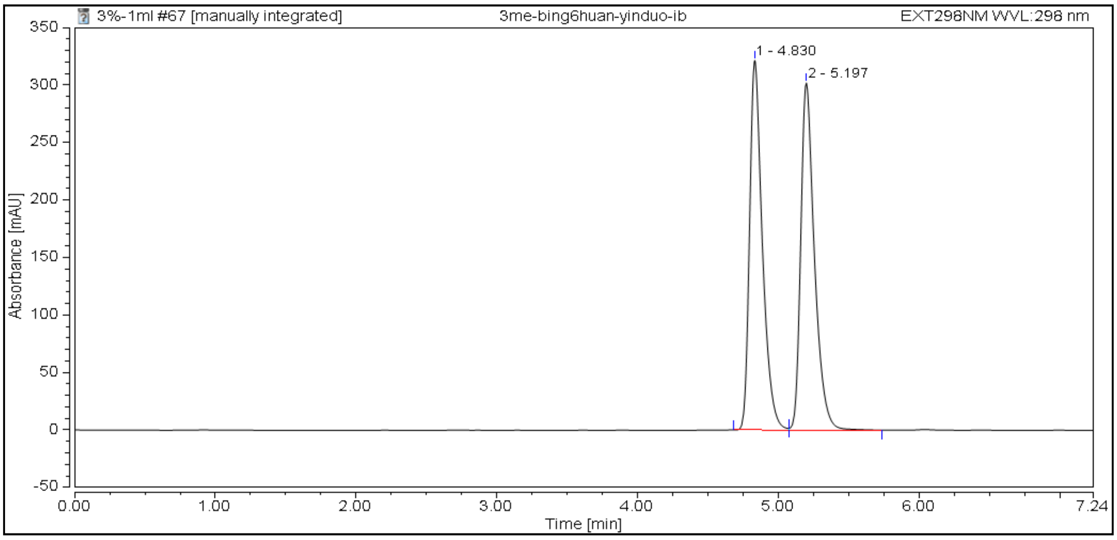

Integration Results

No.	Peak Name	Retention Time min	Area mAU*min	Relative Area %	Amount n.a.
1		4.830	33.755	49.77	n.a.
2		5.197	34.069	50.23	n.a.
**Total:**			**67.825**	**100.00**	

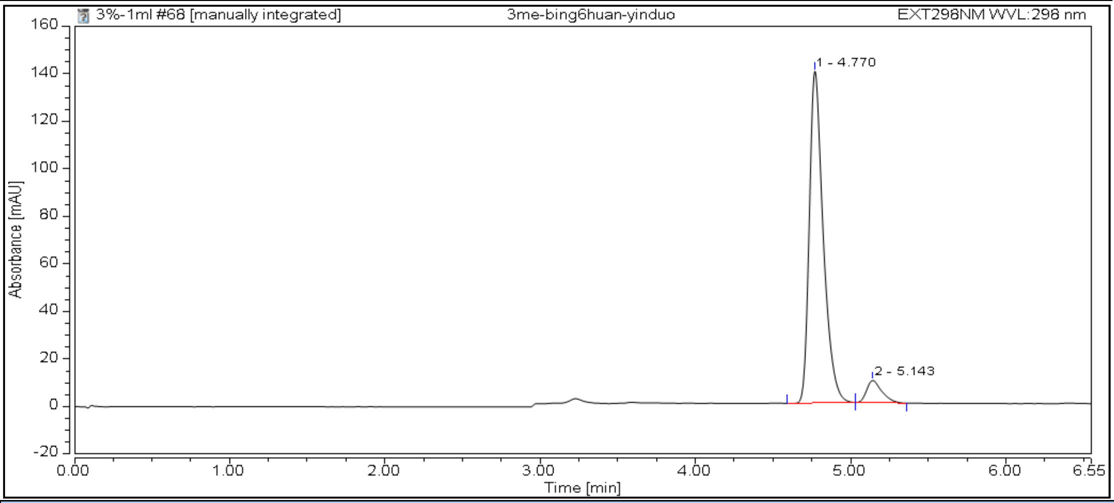

Integration Results

No.	Peak Name	Retention Time min	Area mAU*min	Relative Area %	Amount n.a.
1		4.770	14.507	93.15	n.a.
2		5.143	1.067	6.85	n.a.
**Total:**			**15.574**	**100.00**	

Supplementary figure 174. HPLC chromatogram for **6h**

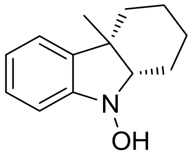

7h

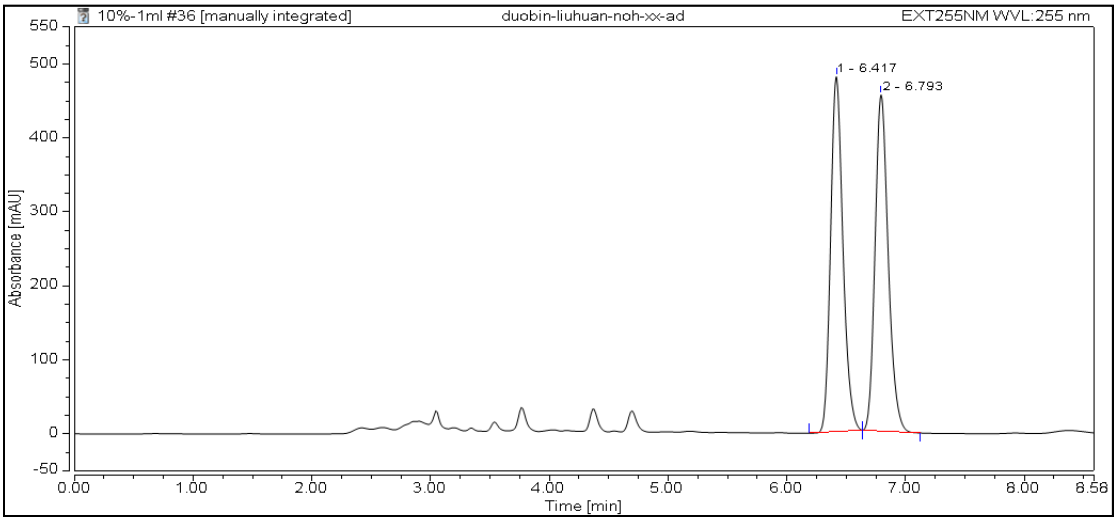

Integration Results					
No.	Peak Name	Retention Time min	Area mAU*min	Relative Area %	Amount n.a.
1		6.417	57.679	49.74	n.a.
2		6.793	58.287	50.26	n.a.
**Total:**			**115.966**	**100.00**	

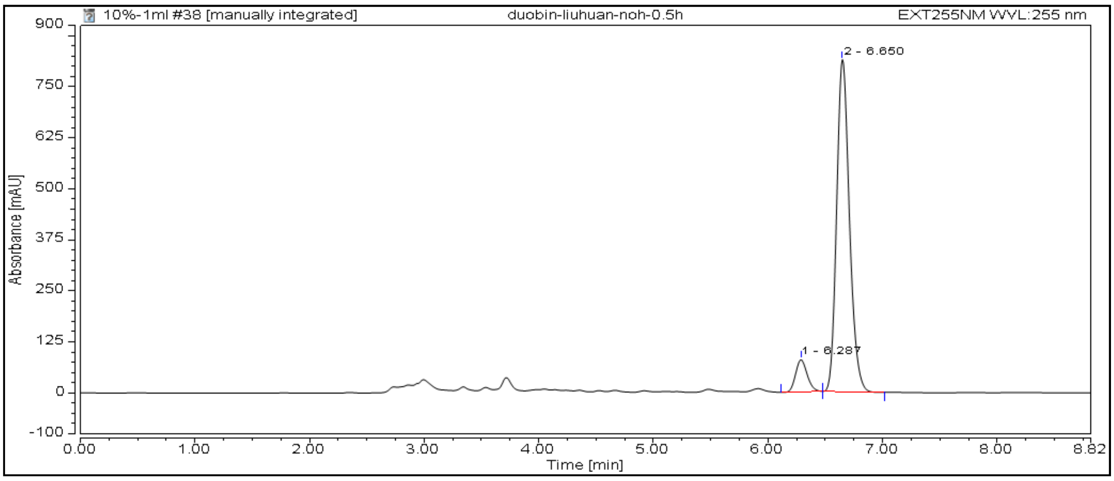

Integration Results					
No.	Peak Name	Retention Time min	Area mAU*min	Relative Area %	Amount n.a.
1		6.287	9.222	8.07	n.a.
2		6.650	105.114	91.93	n.a.
**Total:**			**114.337**	**100.00**	

Supplementary figure 175. HPLC chromatogram for **7h**

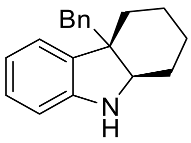

6i

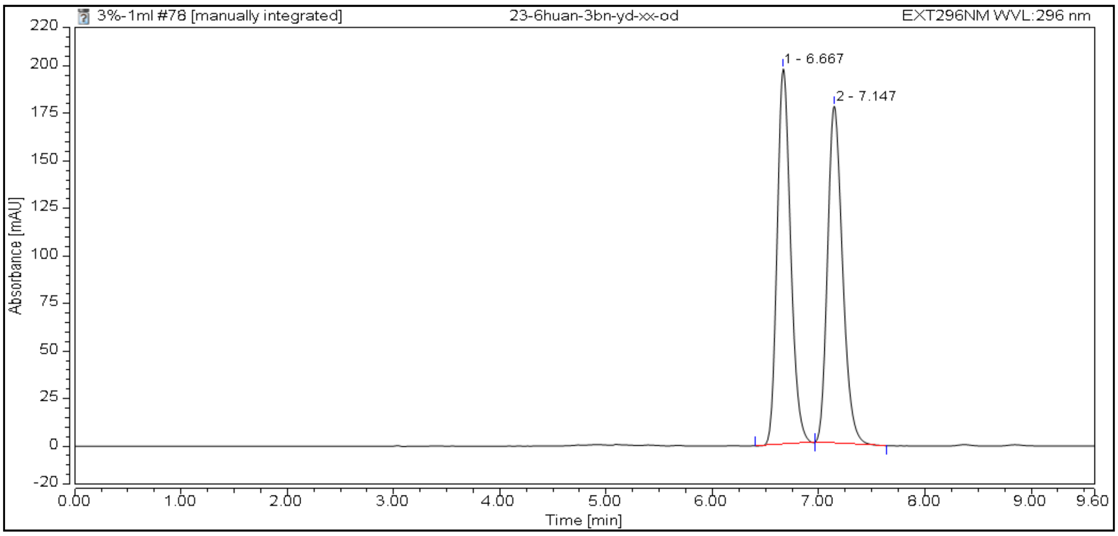

Integration Results

No.	Peak Name	Retention Time min	Area mAU*min	Relative Area %	Amount n.a.
1		6.667	28.895	49.99	n.a.
2		7.147	28.911	50.01	n.a.
**Total:**			**57.806**	**100.00**	

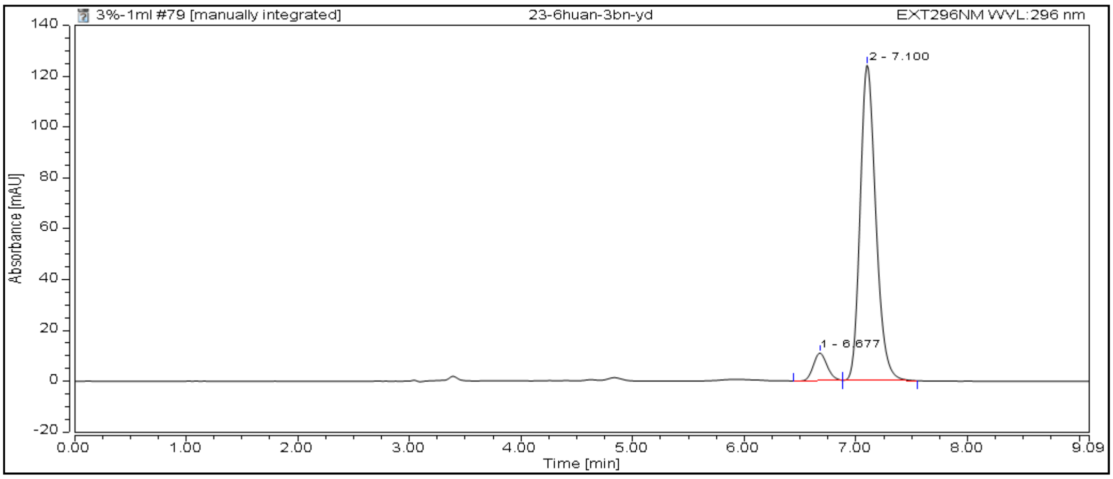

Integration Results

No.	Peak Name	Retention Time min	Area mAU*min	Relative Area %	Amount n.a.
1		6.677	1.538	7.08	n.a.
2		7.100	20.195	92.92	n.a.
**Total:**			**21.733**	**100.00**	

Supplementary figure 176. HPLC chromatogram for **6i**

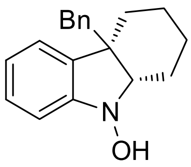

7i

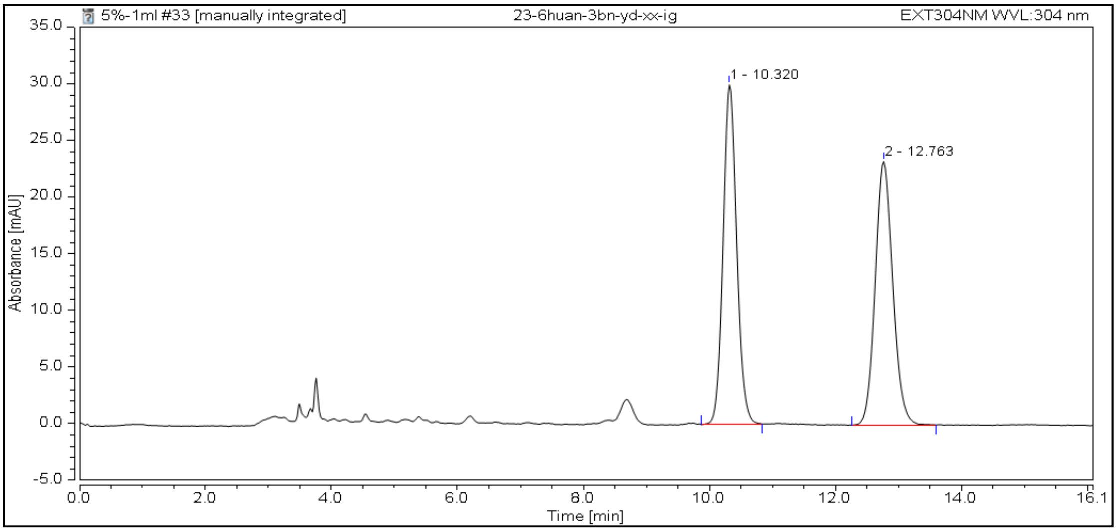

Integration Results					
No.	Peak Name	Retention Time min	Area mAU*min	Relative Area %	Amount n.a.
1		10.320	7.519	49.86	n.a.
2		12.763	7.561	50.14	n.a.
**Total:**			**15.079**	**100.00**	

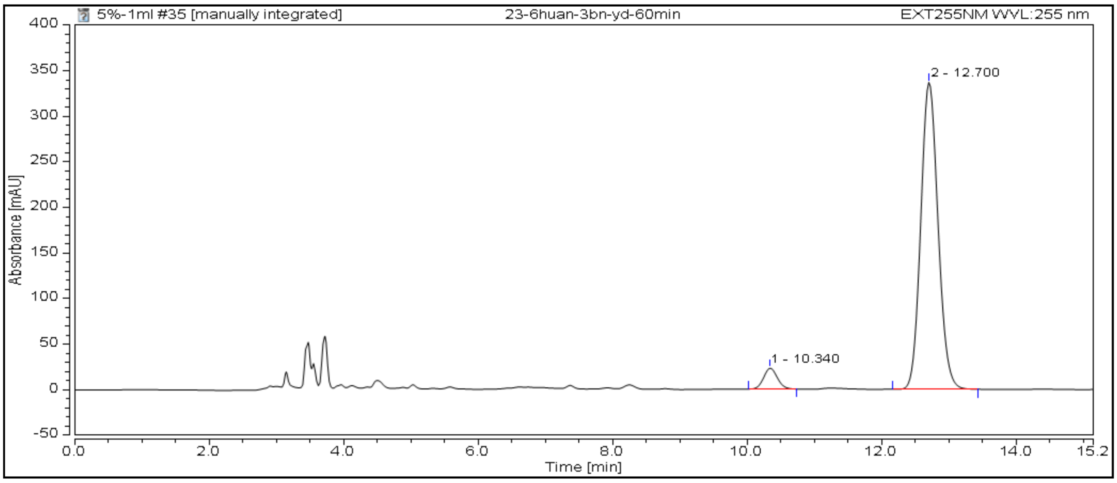

Integration Results					
No.	Peak Name	Retention Time min	Area mAU*min	Relative Area %	Amount n.a.
1		10.340	5.400	5.12	n.a.
2		12.700	100.097	94.88	n.a.
**Total:**			**105.497**	**100.00**	

Supplementary figure 177. HPLC chromatogram for **7i**

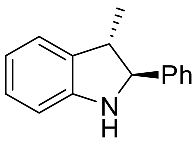

6j

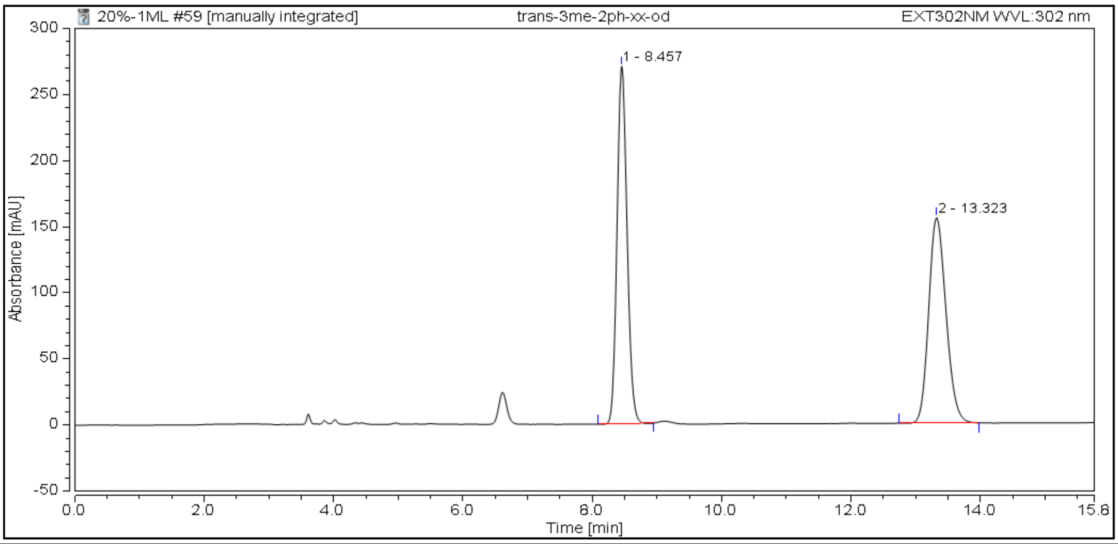

Integration Results					
No.	Peak Name	Retention Time min	Area mAU*min	Relative Area %	Amount n.a.
1		8.457	48.270	50.09	n.a.
2		13.323	48.097	49.91	n.a.
**Total:**			**96.368**	**100.00**	

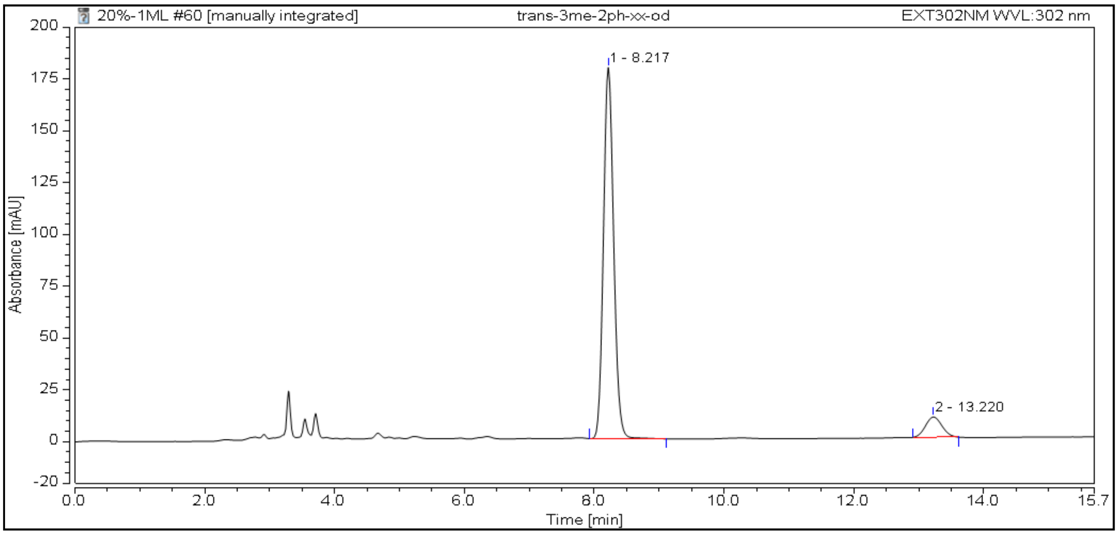

Integration Results					
No.	Peak Name	Retention Time min	Area mAU*min	Relative Area %	Amount n.a.
1		8.217	32.623	91.87	n.a.
2		13.220	2.886	8.13	n.a.
**Total:**			**35.508**	**100.00**	

Supplementary figure 178. HPLC chromatogram for **6j**

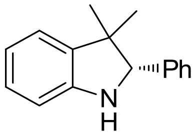

(***S***)-**1a**

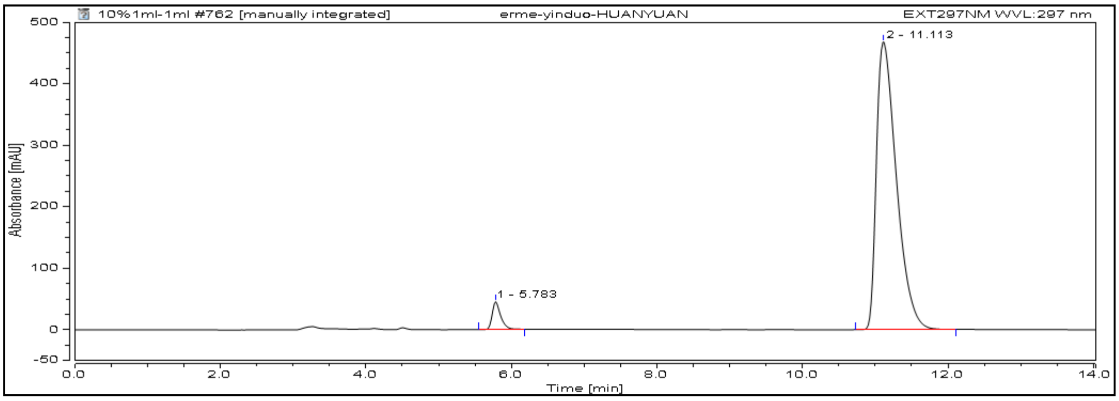

Integration Results					
No.	Peak Name	Retention Time<br>min	Area<br>mAU*min	Relative Area<br>%	Amount<br>n.a.
1		5.783	6.029	3.90	n.a.
2		11.113	148.578	96.10	n.a.
**Total:**			**154.607**	**100.00**	

Supplementary figure 179. HPLC chromatogram for (***S***)-**1a**

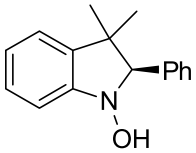

(***R***)-**2a**

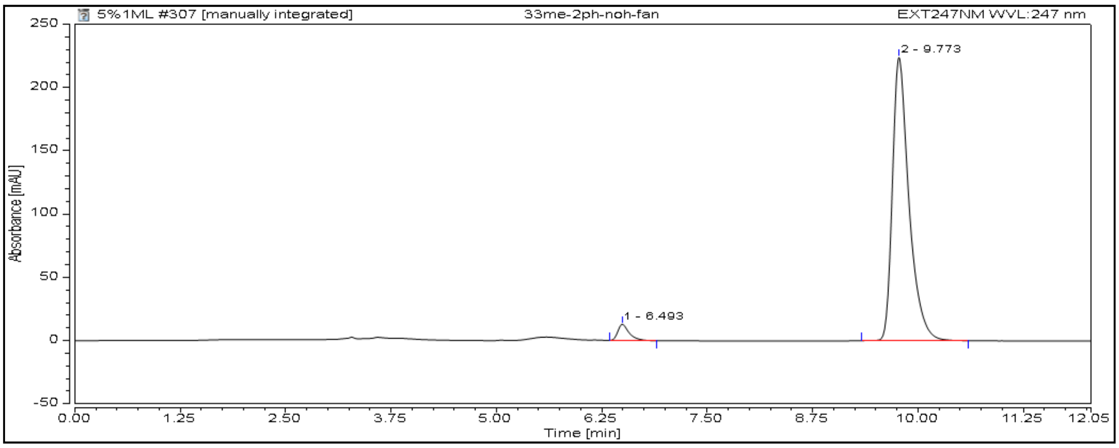

Integration Results					
No.	Peak Name	Retention Time<br>min	Area<br>mAU*min	Relative Area<br>%	Amount<br>n.a.
1		6.493	2.228	4.19	n.a.
2		9.773	50.931	95.81	n.a.
**Total:**			**52.931**	**100.00**	

Supplementary figure 180. HPLC chromatogram for (***R***)-**2a**

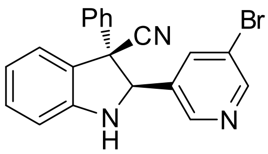

9

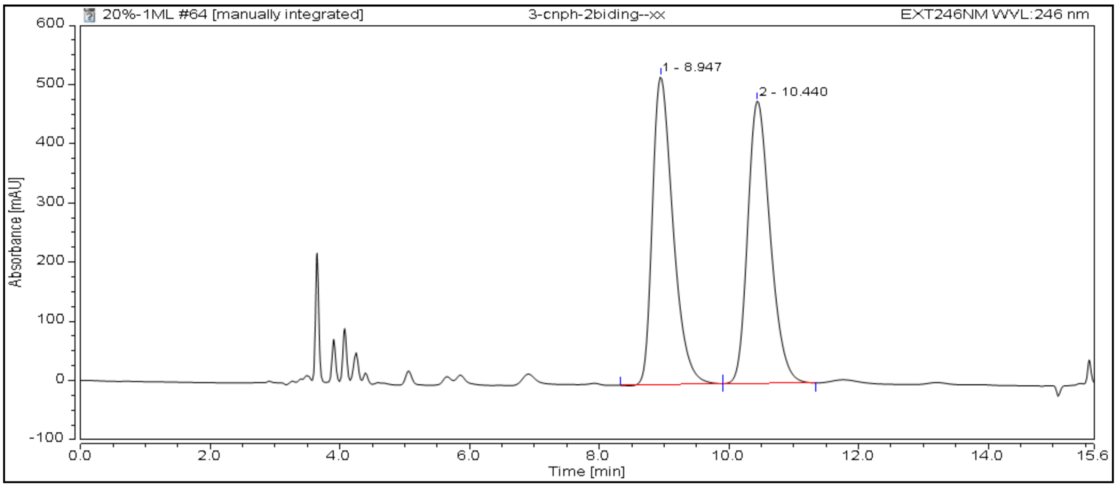

**Integration Results**					
No.	Peak Name	Retention Time<br>min	Area<br>mAU*min	Relative Area<br>%	Amount<br>n.a.
1		8.947	191.344	49.91	n.a.
2		10.440	192.002	50.09	n.a.
**Total:**			**383.346**	**100.00**	

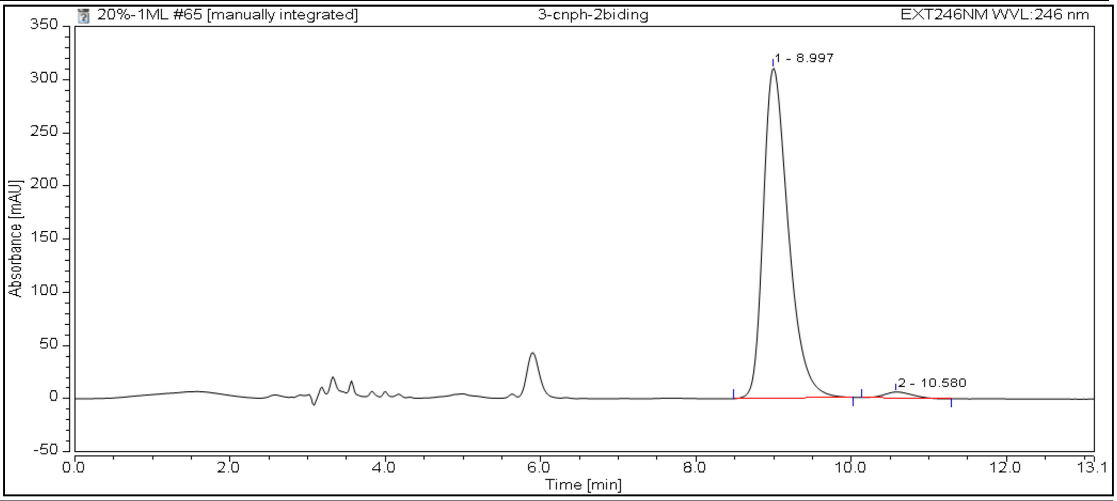

**Integration Results**					
No.	Peak Name	Retention Time<br>min	Area<br>mAU*min	Relative Area<br>%	Amount<br>n.a.
1		8.997	116.029	98.06	n.a.
2		10.580	2.299	1.94	n.a.
**Total:**			**118.328**	**100.00**	

Supplementary figure 181. HPLC chromatogram for **9**

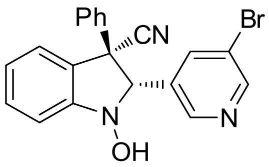

10

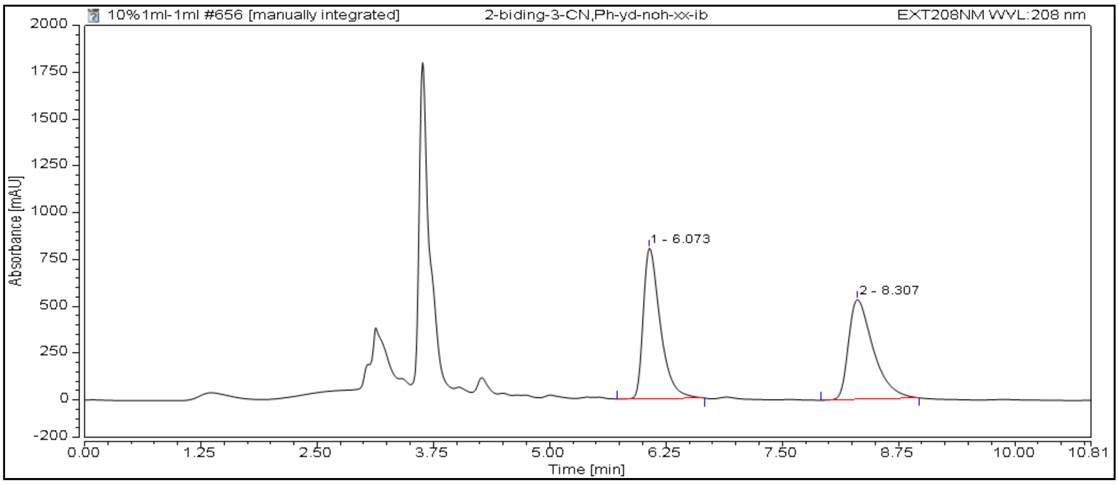

Integration Results					
No.	Peak Name	Retention Time min	Area mAU*min	Relative Area %	Amount n.a.
1		6.073	170.717	50.98	n.a.
2		8.307	164.121	49.02	n.a.
**Total:**			**334.837**	**100.00**	

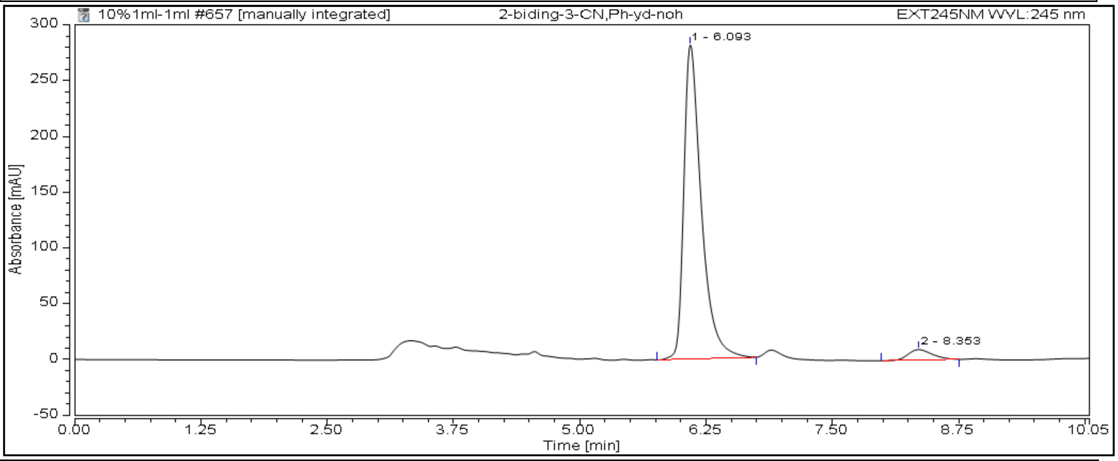

Integration Results					
No.	Peak Name	Retention Time min	Area mAU*min	Relative Area %	Amount n.a.
1		6.093	56.584	95.61	n.a.
2		8.353	2.595	4.39	n.a.
**Total:**			**59.179**	**100.00**	

Supplementary figure 182. HPLC chromatogram for **10**

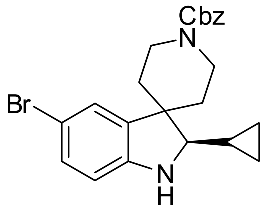

12

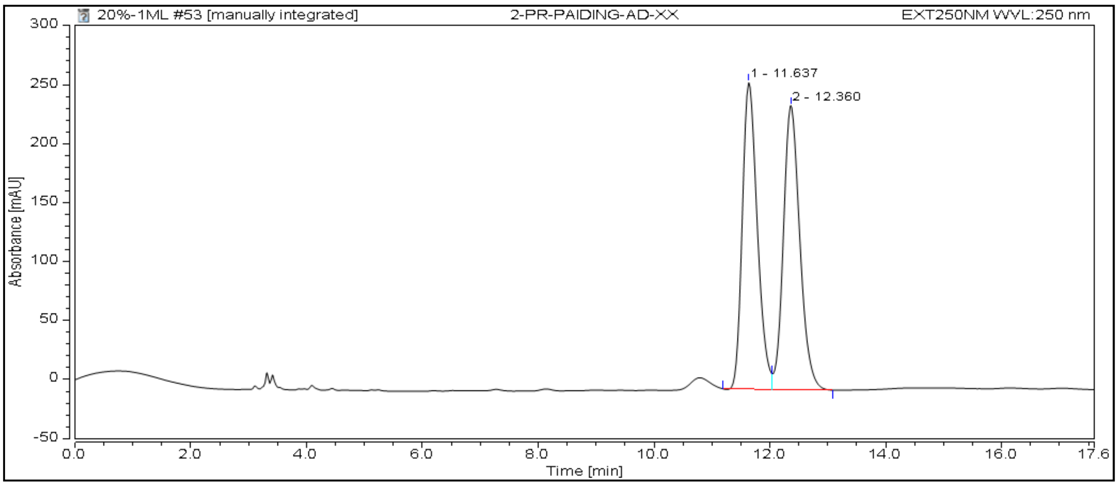

Integration Results					
No.	Peak Name	Retention Time min	Area mAU*min	Relative Area %	Amount n.a.
1		11.637	77.934	49.56	n.a.
2		12.360	79.321	50.44	n.a.
**Total:**			**157.255**	**100.00**	

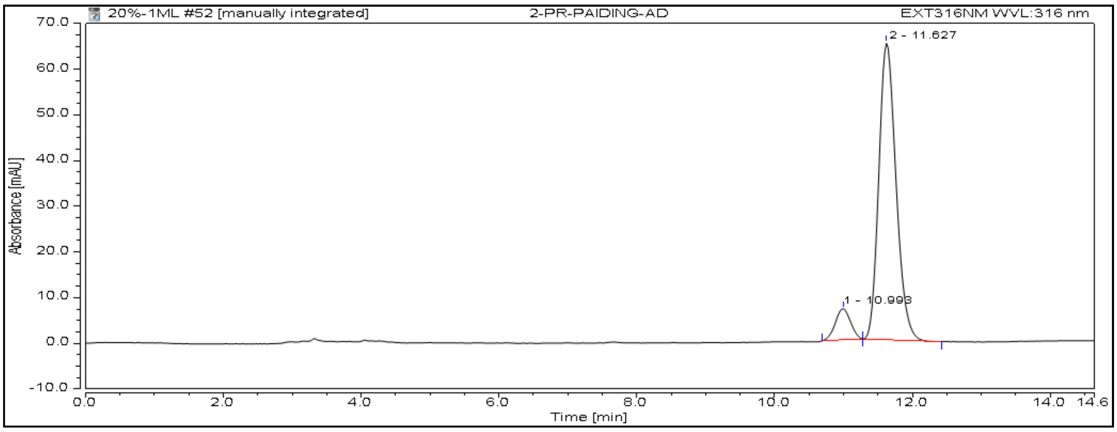

Integration Results					
No.	Peak Name	Retention Time min	Area mAU*min	Relative Area %	Amount n.a.
1		10.993	1.754	8.53	n.a.
2		11.627	18.822	91.47	n.a.
**Total:**			**20.577**	**100.00**	

Supplementary figure 183. HPLC chromatogram for **12**

Supplementary References

1. Saito, K.; Shibata, Y.; Yamanaka, M.; Akiyama, T. Chiral phosphoric acid-catalyzed oxidative kinetic resolution of indolines based on transfer hydrogenation to imines. *J. Am. Chem. Soc.* **135**, 11740 (2013).
2. McComas, C. C.; Gilbert, E. J.; Van Vranken. D. L. Stereochemistry of 3-alkylindole dimerization: acyclic δ_1,δ_1‘-tryptophan dimers. *J. Org. Chem.* **62**, 8600 (1997).
3. Lin A.; Yang J.; Hashim M. *N*-Indolyltriethylborate: a useful reagent for synthesis of C3-quaternary indolenines. *Org. Lett.* **15**, 1950 (2013).
4. Belokon, Y. N.; Caveda-Cepas, S.; Green, B.; Ikonnikov, N. S.; Khrustalev, V. N.; Larichev, V. S.; Moscalenko, M. A.; North, M.; Orizu, C.; Tararov, V. I.; Tasinazzo, M.; Timofeeva, G. I.; Yashkina, L. V. The asymmetric addition of trimethylsilyl cyanide to aldehydes catalyzed by chiral (salen)Titanium complexes. *J. Am. Chem. Soc.* **121**, 3968– 3973 (1999).
5. Matsumoto, K.; Sawada, Y.; Saito, B.; Sakai, K.; Katsuki, T. Construction of pseudo-heterochiral and homochiral di-μ-oxotitanium(Schiff base) dimers and enantioselective epoxidation using aqueous hydrogen peroxide. *Angew. Chem., Int. Ed.* **44**, 4935 (2005).
6. Lackner, A. D.; Samant, A. V.; Toste, F. D.Single-operation deracemization of 3H-indolines and tetrahydroquinolines enabled by phase separation. *J. Am. Chem. Soc.* **135**, 14090 (2013).
7. Ma, W.; Zhang, J.; Xu, C.; Chen, F.; He, Y.-M.; Fan, Q.-H. Highly enantioselective direct synthesis of endocyclic vicinal diamines through chiral Ru(diamine)-catalyzed hydrogenation of 2,2′-bisquinoline derivatives. *Angew. Chem., Int. Ed.* **55**, 12891 (2016).
8. Sharma, K.; Wolstenhulme, J. R.; Painter, P. P.; Yeo, D.; Grande-Carmona, F.; Johnston, C. P.; Tantillo, D. J.; Smith, M. D. Cation-controlled enantioselective and diastereoselective synthesis of indolines: an autoinductive phase-transfer initiated 5-*endo-trig* process. *J. Am. Chem. Soc.* **137**, 13414 (2015).
9. Gerken, P. A.; Wolstenhulme, J. R.; Tumber, A.; Hatch, S. B.; Zhang, Y.; Müller, S.; Chandler, S. A.; Mair, B.; Li, F.; Nijman, S. M. B.; Konietzny, R.; Szommer, T.; Yapp, C.; Fedorov, O.; Benesch, J. L. P.; Vedadi, M.; Kessler, B. M.; Kawamura, A.; Brennan, P. E.; Smith, M. D. Discovery of a highly selective cell-active inhibitor of the histone lysine demethylases KDM2/7. *Angew. Chem., Int. Ed.* **56**, 15555 (2017).
10. Panknin, O.; Bäurle, S.; Ring, S.; Schwede, W.; Schmees, N.; Nowak-Reppel, K.; Langer, G. Spiro[indolin-3,4,-piperidine] derivatives as gnrh receptor anatagonists. PCT Int. Appl. WO 2015091315A1 (2015).
